# Unique Flexibility in Energy Metabolism Allows Mycobacteria to Combat Starvation and Hypoxia

**DOI:** 10.1371/journal.pone.0008614

**Published:** 2010-01-07

**Authors:** Michael Berney, Gregory M. Cook

**Affiliations:** Department of Microbiology and Immunology, Otago School of Medical Sciences, University of Otago, Dunedin, New Zealand; University of California Merced, United States of America

## Abstract

Mycobacteria are a group of obligate aerobes that require oxygen for growth, but paradoxically have the ability to survive and metabolize under hypoxia. The mechanisms responsible for this metabolic plasticity are unknown. Here, we report on the adaptation of *Mycobacterium smegmatis* to slow growth rate and hypoxia using carbon-limited continuous culture. When *M. smegmatis* is switched from a 4.6 h to a 69 h doubling time at a constant oxygen saturation of 50%, the cells respond through the down regulation of respiratory chain components and the F_1_F_o_-ATP synthase, consistent with the cells lower demand for energy at a reduced growth rate. This was paralleled by an up regulation of molecular machinery that allowed more efficient energy generation (i.e. Complex I) and the use of alternative electron donors (e.g. hydrogenases and primary dehydrogenases) to maintain the flow of reducing equivalents to the electron transport chain during conditions of severe energy limitation. A hydrogenase mutant showed a 40% reduction in growth yield highlighting the importance of this enzyme in adaptation to low energy supply. Slow growing cells at 50% oxygen saturation subjected to hypoxia (0.6% oxygen saturation) responded by switching on oxygen scavenging cytochrome *bd*, proton-translocating cytochrome *bc_1_-aa_3_* supercomplex, another putative hydrogenase, and by substituting NAD^+^-dependent enzymes with ferredoxin-dependent enzymes thus highlighting a new pattern of mycobacterial adaptation to hypoxia. The expression of ferredoxins and a hydrogenase provides a potential conduit for disposing of and transferring electrons in the absence of exogenous electron acceptors. The use of ferredoxin-dependent enzymes would allow the cell to maintain a high carbon flux through its central carbon metabolism independent of the NAD^+^/NADH ratio. These data demonstrate the remarkable metabolic plasticity of the mycobacterial cell and provide a new framework for understanding their ability to survive under low energy conditions and hypoxia.

## Introduction

Microorganisms show a remarkable metabolic flexibility that allows them to adapt to various environmental changes (e.g. nutrient starvation, oxygen deprivation and various exogenous stress conditions). A crucial feature in the adaptation of any bacterium to alternative energy sources and changing environmental parameters is the balance of oxidative and reductive reactions in the metabolic scheme that are dependent on electron donor and acceptor availability. Mycobacteria are a group of obligate aerobes that require oxygen for growth, but paradoxically have the extraordinary ability to survive and metabolize under hypoxia suggesting a high degree of metabolic plasticity. The mechanisms responsible for this metabolic flexibility are unknown.

The paucity of basic knowledge in this area reflects the limitations of available models to study the adaptation of mycobacteria to slow growth rate and/or hypoxia. A conventional approach has been to put mycobacterial cells into a low metabolic state using the following stimuli; e.g. low oxygen (Wayne model) [1], nutrient starvation [2] and extended stationary phase [3], [4]. These approaches have provided valuable information, but frequently have multiple factors changing throughout the experiment, and the mycobacteria often fail to grow and metabolize.

A powerful method to study gene expression in response to changing environmental conditions, where the growth rate can be maintained at a constant value, is continuous culture. This also allows the researcher to change the growth rate (i.e. dilution rate) while maintaining other factors (e.g. oxygen tension) at a constant value. For example, carbon-limited continuous culture was used to study the effect of growth rate on gene expression in *Mycobacterium bovis* BCG [5]. Slowing the growth rate of *M. bovis* BCG from a 23 h to a 69 h doubling time at 70–100% oxygen saturation has been shown to precipitate a transcriptional response more akin to growth of *M. tuberculosis* in macrophages compared to other *in vitro* models. However, in the study by Beste *et al*. [5], the growth rate varied by only 3-fold whereas the change in growth rate during the transition to hypoxia or nutrient limitation is likely to be much greater than this value. Bacon *et al.* [6] report on the response of *M. tuberculosis* to lowering oxygen saturation (20% and 1%) at a constant mean generation time of 24 h in glycerol-limited continuous culture. However, no study has yet been performed that dissects both the response to carbon starvation and oxygen limitation simultaneously.

The aim of the current study was to determine the molecular response of *M. smegmatis* to changing growth rate (15-fold difference) and decreasing oxygen supply (50% to 0.6% oxygen saturation) using carbon-limited continuous culture. We rationalized that this would be a valid model to elucidate the metabolism and energetics of *M. smegmatis* growth in slowly metabolizing cells and in cells confronted with oxygen limitation.

## Results

### Establishing Carbon-Limited Continuous Culture of *M. smegmatis*

**Figure 1:**
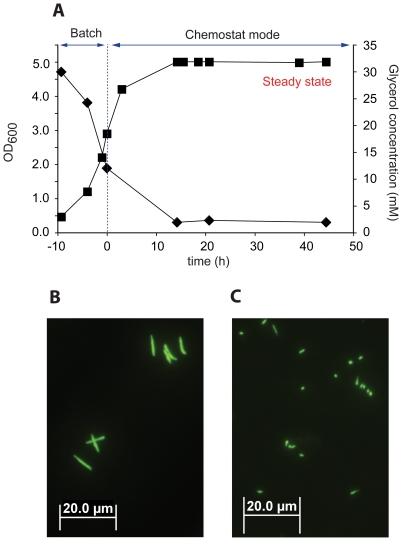
Growth of *M. smegmatis* in continuous culture. A: Growth curve of *M. smegmatis* based on OD_600_ (solid squares) and residual glycerol concentration (solid diamonds) measured during chemostat cultivation at a dilution rate of 0.15 h^−1^. B&C: Fluorescence microscopy pictures of *Mycobacterium smegmatis* grown in continuous cultivation at a dilution rate of (B) 0.15 h^−1^ (fast) and (C) 0.01 h^−1^ (slow) and stained with SYBRGreen I.

*M. smegmatis* was grown in carbon-limited continuous culture at dilution rates of either 0.15 h^−1^ (fast) or 0.01 h^−1^ (slow) that corresponded to doubling times of 4.6 h and 69 h respectively. A doubling time of 69 h has been used in other continuous culture studies with mycobacteria (e.g. *M. bovis* BCG) to simulate growth rates akin to those *in vivo* [5]. Oxygen supply was maintained at 50%, 2.5% or 0.6% oxygen saturation and these values were chosen to be comparable with the oxygen tensions observed in the Wayne model [1] i.e. during exponential growth phase (50%), beginning of NRP1 (2.5%) and advanced NRP1 (0.6%), respectively. Oxygen saturation values of 70–100% [7] and 20–50% [6] have been used in other continuous culture studies with mycobacteria. Steady state growth was confirmed in all cultures by monitoring the optical density of the culture as well as oxygen consumption. Carbon limitation was tested by pulsing glycerol into the culture vessel and showing an increase in OD_600_, as well as respiration rate, and by measuring residual glycerol (Figure 1). The steady-state optical density was significantly lower at low dilution rate (OD_600_ = 4.2±0.1) compared to high dilution rate (5.1±0.2). The number of viable cells was higher at low dilution rate than at high dilution rate indicating a difference in cell volume (Table 1). This was confirmed by fluorescence microscopy, which revealed a 7-fold difference in cell length between slow and fast growing cells (Figure 1B and 1C). The growth yields on glycerol and the magnitude of the proton-motive force were comparable at both dilution rates suggesting the cells were energetically equivalent (Table 1).

**Table 1:** Bulk parameters measured at steady state in continuous culture experiments with *M. smegmatis*.

Dilution rate (h^−1^)	OD_600_	CFU (*10^9^)/ml	Yield glycerol (g biomass/g glycerol)	Membrane potential (mV)	ZΔpH (mV)^a^	ΔµH^+^ (mV)
0.01	4.2±0.1	1.5±0.6	0.56±0.04	75±5	55±7	130±12
0.15	5.1±0.2	0.7±0.3	0.62±0.01	64±8	48±5	111±13

a Extracellular pH was 6.6.

When the oxygen saturation was lowered from 50% to 2.5%, the oxygen demand decreased (stirring rate) and the culture maintained growth at a 69 hours doubling time. At 0.6% oxygen saturation, the doubling time was greater (86 hours) indicating that the chemostat culture was oxygen-limited.

### Differential Gene Expression in Response to Slow Growth Rate and Low Oxygen Concentration

A total of 1294 genes of *M. smegmatis* were differentially expressed (p-value<0.05, expression ratio >2 or<0.5) at low dilution rate compared to high dilution rate. Of these, 691 genes were upregulated and 603 were downregulated. For a complete list of genes, see Dataset S1. At 2.5% oxygen saturation and a dilution rate of 69 h, 28 genes were downregulated and 91 upregulated. For a complete list of genes, see Dataset S1. Most prominently, two gene clusters (*msmeg_3932-3955* and *msmeg_5241-5246*) with homology to DosR-regulated genes in *M. tuberculosis* were upregulated. Both clusters contain a copy of the gene *devR,* the homologous gene to *dosR* in *M. tuberculosis*. At 0.6% oxygen saturation, the expression ratio of these gene clusters increased and additional genes were differentially regulated. In total, we found 870 genes differentially expressed (p-value<0.05) at 0.6% oxygen saturation compared to 50% oxygen saturation, 429 genes above a 2-fold expression ratio and 441 genes below 0.5-fold threshold level. For a complete list of genes, see Dataset S1.

To check the quality of our microarray data, we used quantitative RT-PCR to validate expression ratios for selected genes (Figure S3). Figure S3 demonstrates that real-time PCR results correlated well with the microarray results.

### Energy Metabolism

#### Energy-limiting conditions

**Figure 2:**
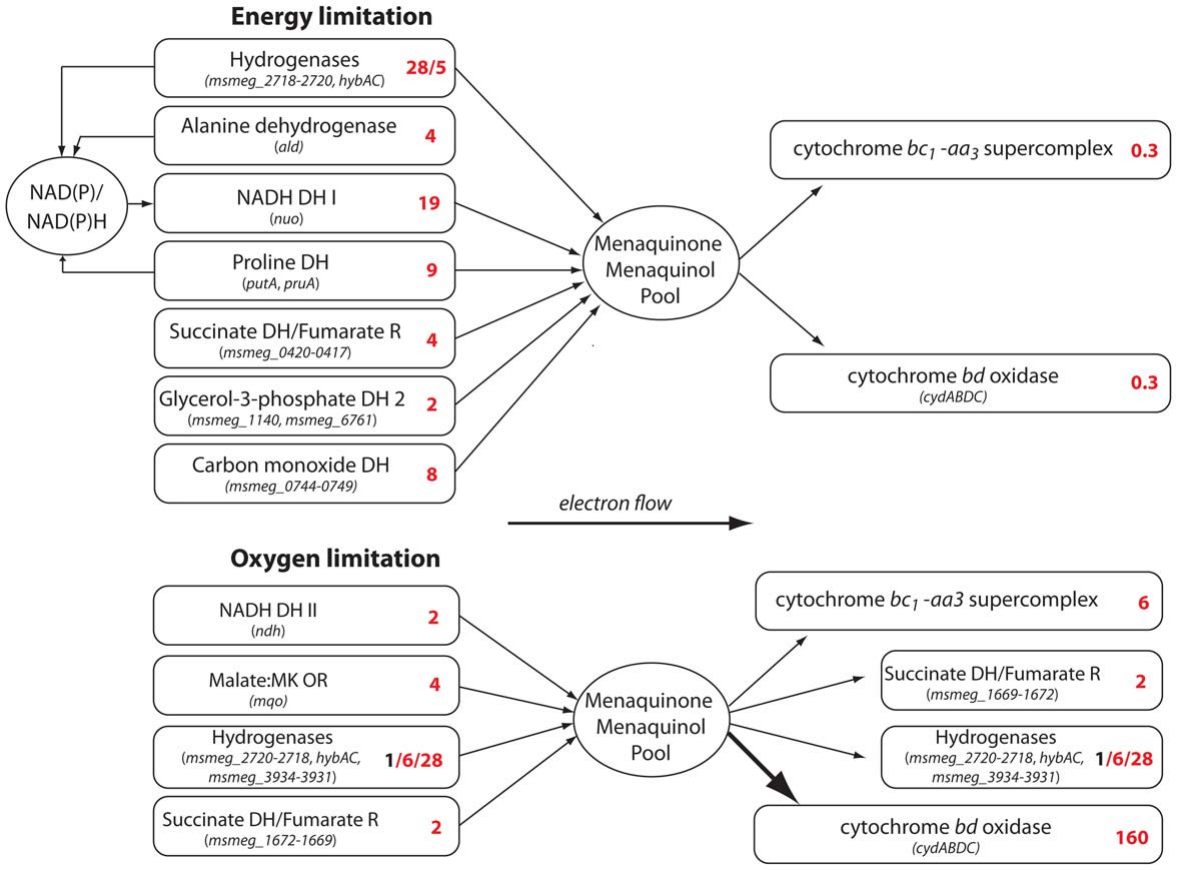
Proposed scheme of enzymes preferentially used in the respiratory chain of *M. smegmatis* under energy- and oxygen-limiting conditions. Data shown is based on gene expression ratios (indicated as red numbers next to gene name). See Table 2, Table S1 and Dataset S1 for detailed expression data.

In response to low dilution rate at 50% oxygen saturation, 128 genes involved in energy metabolism were upregulated and 62 downregulated. The majority of the downregulated genes encode components of the oxidative phosphorylation machinery. These included the gene clusters for the F_1_F_O_-ATP synthase, succinate dehydrogenase (*msmeg_1669-1672*), cytochrome *bc_1_-aa_3_* supercomplex, and cytochrome *bd* (Table S1). Strikingly, strong upregulation of gene clusters encoding for two Ni-Fe-hydrogenases (*msmeg_2262-2264 and msmeg_2720-2713)*, NADH-quinone oxidoreductase (*nuo*), propane monoxygenase, carbon-monoxide dehydrogenase and a succinate dehydrogenase (*msmeg_0417- 0419*) were observed. Furthermore, several primary dehydrogenases proposed to be electron donors to the electron transport chain were upregulated at slow growth rate. These included proline dehydrogenase, alanine dehydrogenase, glycerol-3-phosphate dehydrogenase 2, and a putative pyruvate dehydrogenase (Figure 2). The data for genes with a purported role in energy metabolism is summarized in Table S1 and specifically for hydrogenases in Table 2. Additionally, we found 17 out of 32 cytochrome P450 enzymes, several alcohol and aldehyde dehydrogenases were all upregulated (Dataset S1).

**Table 2:** Expression ratios of gene clusters encoding for hydrogenases and assembly genes in *M. smegmatis* at different growth rates and oxygen concentrations.

Gene ID	Gene name	Slow/Fast^1^	Hypoxia^2^ 2.5% O_2_	Hypoxia^2^ 0.6% O_2_	Description
MSMEG_2262	*hybA*	**5.4**	**4.1**	**5.7**	hydrogenase-2, small subunit
MSMEG_2263	*hybC*	**5.4**	3.7	**6.2**	hydrogenase-2, large subunit
MSMEG_2264		**2.3**	3.5	**4.4**	peptidase M52, hydrogen uptake protein
MSMEG_2271	*hypB*	**1.5**	**2.2**	2.3	hydrogenase accessory protein
MSMEG_2272	*hypA*	**1.9**	**2.8**	1.8	hydrogenase nickel insertion protein HypA
MSMEG_2273	*hypF*	**2.0**	**2.0**	3.0	[NiFe] hydrogenase maturation protein HypF
MSMEG_2274	*hypC*	**1.8**	2.1	**2.5**	hydrogenase assembly chaperone HypC/HupF
MSMEG_2275	*hypD*	**1.8**	**3.0**	5.2	hydrogenase expression/formation protein HypD
MSMEG_2276	*hypE*	**2.0**	**2.0**	2.0	hydrogenase expression/formation protein HypE
MSMEG_2702	*hypD*	**3.6**	1.4	**2.4**	hydrogenase expression/formation protein HypD
MSMEG_2703	*hypC*	**7.0**	1.5	**3.5**	hydrogenase assembly chaperone HypC/HupF
MSMEG_2705	*hypE*	**4.9**	1.2	3.7	hydrogenase expression/formation protein HypE
MSMEG_2711	*hypF*	7.0	1.6	11.2	[NiFe] hydrogenase maturation protein HypF
MSMEG_2712	*hypC*	**15.1**	1.5	**12.1**	hydrogenase assembly chaperone HypC/HupF
MSMEG_2713		**14.5**	1.4	**8.9**	peptidase M52, hydrogen uptake protein
MSMEG_2718		**15.4**	0.9	**3.5**	iron-sulfur cluster-binding protein, Rieske family
MSMEG_2719		**18.2**	1.1	**6.6**	hydrogen:quinone oxidoreductase
MSMEG_2720		**27.8**	1.2	3.1	NADH ubiquinone oxidoreductase, 20 kda subunit
MSMEG_2721	*hypB*	**7.9**	1.0	1.0	hydrogenase accessory protein HypB
MSMEG_2722	*hypA*	**1.5**	1.2	0.8	hydrogenase nickel insertion protein HypA
MSMEG_3927		1.4	**5.2**	**18.6**	peptidase M52, hydrogen uptake protein
MSMEG_3928		**2.6**	**2.2**	**7.6**	[NiFe] hydrogenase, alpha subunit, putative
MSMEG_3929		1.0	**8.2**	**26.9**	[NiFe] hydrogenase, delta subunit, putative
MSMEG_3930		1.2	**10.0**	**28.0**	[NiFe] hydrogenase, gamma subunit, putative
MSMEG_3931		1.0	**16.7**	67.3	[NiFe] hydrogenase, beta subunit, putative

Expression ratios with a p-value<0.05 are indicated with bold numbers.

1 Gene expression ratios of slow growing (0.01 h^−1^) versus fast growing (0.15 h^−1^) chemostat cultures at 50% oxygen saturation.

2 Gene expression ratios of slow growing (0.01 h^−1^, 50% oxygen saturation) versus slow growing cultures at either 2.5% or 0.6% oxygen saturation.

#### Oxygen-limiting conditions

**Figure 3:**
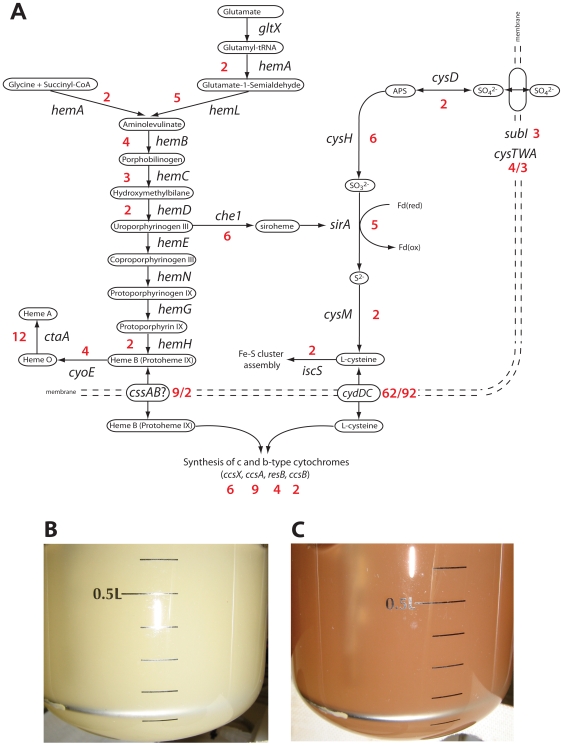
Effect of oxygen limitation on *M. smegmatis* growing at slow growth rate. (A) Diagram of the upregulated heme, cysteine and cytochrome c biosynthesis pathways in *M. smegmatis* under oxygen-limited growth. Numbers in red represent gene expression ratios (0.6% oxygen saturation/50% oxygen saturation) above the 2-fold threshold. B&C: Comparison of *M. smegmatis* mc^2^155 wild type growing in continuous culture at a dilution rate of 0.01 h^−1^ (69 h doubling time) with 50% oxygen saturation (B) or 0.6% oxygen saturation (C).

When the oxygen saturation was lowered to 2.5%, 8 genes involved in energy metabolism were upregulated. These included, two Ni-Fe-hydrogenase gene clusters [*msmeg_2262-2264* (Hyd1) *and msmeg_3931-3927* (Hyd3)], soluble pyridine nucleotide transhydrogenase *sthA* and a conserved hypothetical protein. In contrast, at an oxygen saturation of 0.6% we observed a distinct gene expression pattern (Table S1). The high-affinity cytochrome *bd* oxidase was upregulated >50-fold, along with heme and cysteine biosynthesis pathways (Figure 3A), and genes annotated or with similarity to known cytochrome *c* biogenesis proteins (*msmeg_0971-0974*). Furthermore, cysteine desulfurase (*msmeg_2347*), a protein involved in Fe-S cluster assembly [8] was upregulated 2-fold. Unexpectedly, given the low oxygen saturation, cytochrome *bc_1_-aa_3_* complex was upregulated (5-fold), along with the cytochrome *aa_3_* controlling protein *msmeg_3117* (12-fold). Nearly all dehydrogenases that were upregulated during slow growth showed very low mRNA levels under hypoxia (Table S1). The only exceptions were the hydrogenases, which showed a 2- to 5-fold increase in expression (Table 2). This indicates that *M. smegmatis* makes a distinct transcriptional switch from energy starvation to oxygen limitation and that hydrogenases would appear to play an important role in this adaptation. Furthermore, oxygen-limited growth of *M. smegmatis* was accompanied by a change in the coloration of the cells from a light yellow pigmentation to a red pigmentation (Figure 3B and C).

### TCA and Glyoxylate Shunt Pathways

#### Energy limiting conditions

The majority of genes of the TCA cycle and glycolysis/gluconeogenesis did not show a change in gene expression at low growth rate. The two putative succinate dehydrogenase operons [*msmeg_0420-0417* (Sdh1) and *msmeg_1672-1669* (Sdh2)] showed opposite expression ratios. While Sdh1 was upregulated 4-fold, Sdh2 was downregulated 3-fold like most other genes of the respiratory chain. Succinate dehydrogenase plays an important role in respiration and the TCA cycle. Both enzyme complexes have close homologues in *M. tuberculosis* (*Rv0250-Rv0247, Rv3316-Rv3319*). Additionally, three genes annotated as subunits of pyruvate dehydrogenase (*msmeg_4710-4712*) were strongly upregulated (up to 11-fold) in response to slow growth rate. The genome of *M. smegmatis* encodes two isocitrate lyase enzymes and both are homologous to those found in *M. tuberculosis* CDC1551. At low dilution rate, one isocitrate lyase (*msmeg_3706*) was upregulated while the second copy *msmeg_0911* was downregulated (Dataset S1).

#### Oxygen-limiting conditions

**Figure 4:**
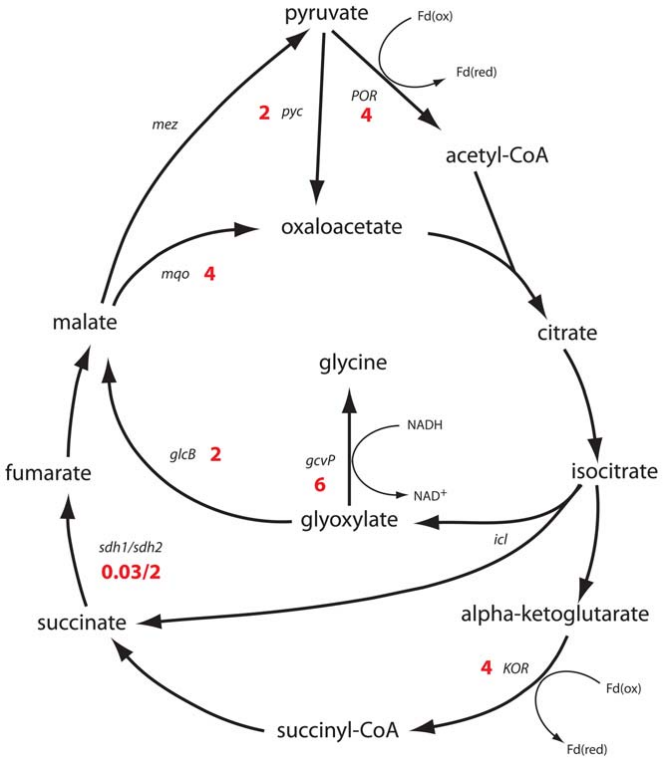
Expression of genes encoding enzymes of the TCA cycle and glyoxylate shunt during growth of *M. smegmatis* under microaerobic conditions. Annotation: *sdh1/sdh2* – succinate dehydrogenase (*msmeg_0420-0417*/*msmeg_1672-1669*), *pyc* – pyruvate carboxylase, *POR* – pyruvate:ferredoxin oxidoreductase, *mqo* – malate:quinone oxidoreductase, *mez* – malic enzyme, *gcvP* - glycine dehydrogenase, *glcB* – malate synthase, *icl* – isocitrate lyase, *KOR* – alpha-ketoglutarate:ferredoxin oxidoreductase.

Under oxygen-limiting conditions (0.6% oxygen saturation), several enzymes of the TCA cycle and glyoxylate shunt were differentially regulated compared to cultures grown at 50% oxygen saturation (Figure 4). Genes for malate synthase were upregulated as well as glycine dehydrogenase, which converts glyoxylate to glycine and recycles NADH to NAD^+^. Furthermore, *M. smegmatis* does not harbour a NAD^+^-dependent malate dehydrogenase, but instead expresses a malate:quinone oxidoreductase (MQO), which feeds electrons directly into the menaquinone pool. MQO was upregulated 4-fold under hypoxia (Figure 4). Additionally, a putative pyruvate:ferredoxin oxidoreductase or α-ketoglutarate:ferredoxin oxidoreductase (4-fold p = 0.02), pyruvate carboxylase (2.5-fold, p = 0.02), succinate dehydrogenase (Sdh2) (2-fold p = 0.02) and phosphoenolpyruvate synthase (23-fold, p = 0.08) were upregulated (Dataset S1). Genes downregulated under these conditions included malic enzyme, succinate dehydrogenase (Sdh1), pyruvate kinase, putative pyruvate dehydrogenase and succinic semialdehyde dehydrogenase (Dataset S1).

#### Regulation and signal transduction

The transcriptional response to 2.5% oxygen saturation at slow growth rate was manifested predominantly in the upregulation of two gene clusters with 31 genes (*msmeg_3926*-*msmeg_3955*) and 6 genes (*msmeg_5241*-*msmeg_5246*) respectively. Both clusters are near the two-component regulatory system DevR/DevS, of which *M. smegmatis* harbors two copies (see Dataset S1). Most of these genes have homologues in *M. tuberculosis* and are part of the Dos regulon [9]. Exceptions to this are the 5 genes encoding a Ni-Fe hydrogenase, a cation-transporting P-type ATPase and the gene encoding for phosphoenolpyruvate synthase. The expression pattern of these genes was similar at 0.6% oxygen saturation although a few genes showed increased expression (Dataset S1). Three two-component regulatory systems (i.e. *msmeg_0854/msmeg_0856*, *msmeg_4968/msmeg_4969* and *msmeg_6236/msmeg_6238*) were upregulated at slow growth rate while *senX3/regX3* and *msmeg_5487/msmeg_5488* (MprA) were upregulated under hypoxia.

The *M. smegmatis* genome is annotated to have 29 sigma factors and 4 anti-sigma factors. At slow growth rate, the expression ratio of 17 sigma factors and 3 anti-sigma factors was significantly different from 1 (p<0.05) (Dataset S1). All but one sigma factor (*sigF*) and one anti-sigma factor were upregulated. The genes *sigG, sigH, sigL, sigJ, sigE* (all with homologues in *M. tuberculosis*) and *msmeg_1970* were upregulated more than 2-fold (Dataset S1). Sigma factor genes *sigB*, *sigE, sigD, sigH* and *sigJ* showed increased mRNA levels under oxygen limiting conditions, while *sigG* and *sigL* levels were decreased. One out of 6 WhiB transcription factors, i.e. *msmeg_6199*, showed a >4-fold induction in response to slow growth. The homologue in *M. tuberculosis* is WhiB4 and is believed to be involved in redox signaling [10]. When the oxygen saturation was lowered to 0.6%, the same gene was downregulated 20-fold. Additionally, *msmeg_1919* (WhiB1) responded to hypoxia with an 8-fold upregulation (Dataset S1).

### Hydrogenase Gene Clusters in *M. smegmatis* and Characterization of a Hydrogenase Mutant

**Figure 5:**
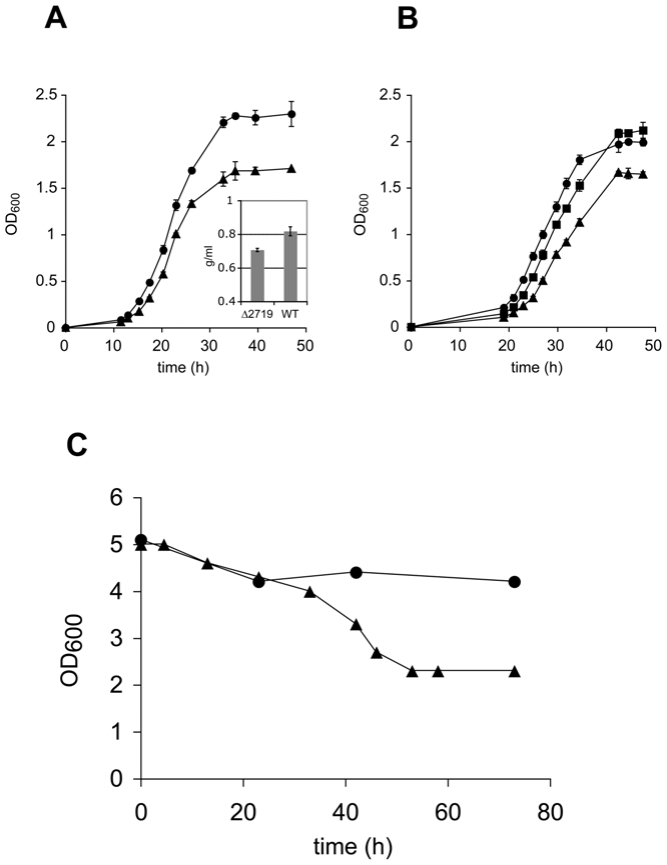
Comparative analysis of wild-type *M. smegmatis* mc^2^155 with an isogenic hydrogenase mutant (Δ2719). A. Growth curve of *M. smegmatis* mc^2^155 wild-type (solid circles) versus strain Δ2719 (solid triangles) in LB Tween80. Insert: Biomass of the two strains based on dry weight measurements. Error bars represent standard deviations from 3 biological replicates. B. Complementation experiment: Growth curve of *M. smegmatis* mc^2^155 mutant strain Δ2719_pOLYG (solid triangles), strain wt_pOLYG (solid circles) and the complemented mutant strain Δ2719_pOLYGHyd2 (solid squares) in LB Tween80. C. Optical density of wild-type strain (solid circles) and mutant strain Δ2719 (solid triangles) measured during chemostat runs at a dilution rate of 0.01 h^−1^. Time zero represents start of chemostat mode.

There are three different putative hydrogenase gene clusters (complexes) in the genome of *M. smegmatis*, two of which showed strong upregulation under energy limiting conditions and all three were upregulated under hypoxia (Table 2). All three enzyme complexes are predicted to have a Ni-Fe center (or Ni-Fe-Se) and to be soluble proteins (PFAM and THMMH analysis). According to these predictions, their cofactors could be either NADH, ferredoxin or coenzyme F_420_. A phylogenetic analysis on the catalytic subunit of each of the three enzymes revealed that they share homology with hydrogenases found in cyanobacteria (*msmseg_2263*), other soil microorganisms (*msmeg_2719*), and pathogenic microorganisms (*msmeg_3928*) (Figure S1). To elucidate the role of these hydrogenases in response to slow growth rate (i.e. energy limitation), we constructed a markerless mutation in *msmeg_2719,* a gene encoding a putative hydrogen:quinone oxidoreductase that was the most strongly upregulated of the three hydrogenases in response to growth rate (see Table 2). The gene disruption in *msmeg_2719* was confirmed via Southern hybridization (Figure S2). When grown in LBT medium (batch growth), the mutant exhibited a 20% reduction in biomass compared to the isogenic wild-type parent strain (Figure 5A). Complementation of *msmeg_2719* with the operon *msmeg_2720*-*msmeg_2718*, under control of its native promoter, restored growth behavior to wild type levels (Figure 5B). The difference in growth yield was significantly greater (40%) when grown in continuous culture at a dilution rate of 69 h (50% oxygen saturation) demonstrating the essential role of this hydrogenase in the adaptation of *M. smegmatis* to energy limitation. No H_2_ gas was detected in the culture headspace from both batch and continuous culture using gas chromatography suggesting that *M. smegmatis* did not produce detectable levels of H_2_ using the culture conditions employed in this study.

## Discussion

### Adaptation of *M. smegmatis* to Low Growth Rate Activates the Expression of Alternative Primary Dehydrogenases and Hydrogenases for Energy Generation and Unique Pathways for Scavenging Carbon

The results of this study demonstrate that *M. smegmatis* represents an important model to dissect the physiological response mycobacteria exhibit during adaptation to low energy conditions and hypoxia. As mycobacteria make the transition from fast to slow growth rate, the cell adjusts its metabolic machinery to match physiological demands for energy to drive catabolic and anabolic reactions in the cell. This effect was manifested by a downregulation of respiratory chain components commensurate with decreased rates of oxygen consumption. Despite the lower rates of respiration, the membrane potential was maintained at the same magnitude as fast growing cells. This suggests that energy-limited cells switch to more energy-efficient mechanisms for membrane potential generation and this was supported by the upregulation of the proton-translocating NADH:menaquinone dehydrogenase complex I. To power the respiratory chain under energy-limited conditions, mycobacteria switch to alternative primary dehydrogenases to provide reducing equivalents for respiration. For example, succinate dehydrogenase, alanine dehydrogenase, Ni-Fe-hydrogenases and proline dehydrogenase were all upregulated in response to slow growth rate (Figure 2).

The physiological adaptation of *M. smegmatis* to energy limitation included the induction of enzymes to transport sugars and degrade a wide array of natural and xenobiotic compounds. However, apart from glycerol, Tween80 and sodium-glutamate (0.05%), there were no additional carbon sources present in the growth medium. The observed derepression of alternative catabolic functions in the absence of the respective inducers (substrates) presumably is advantageous in many natural growth situations [11]. Two putative succinate dehydrogenases were the only TCA cycle enzymes that showed induction/repression under energy limiting conditions. The role of succinate dehydrogenase and the reason for the presence of two putative Sdh enzymes with different membrane-spanning regions in mycobacterial species is still unclear [12]. *M. tuberculosis* contains close homologues of both Sdh enzymes and expresses an additional fumarate reductase. Succinate dehydrogenase (Sdh) and fumarate reductase (Frd) are enzymatically and structurally similar proteins. In mycobacteria the membrane-bound electron acceptor for Sdh is menaquinone, indicating that the conversion of fumarate to succinate is a thermodynamically favourable reaction [12]. The *M. smegmatis* genome does not encode a designated fumarate reductase, thus we hypothesize that at least one of the annotated Sdh enzymes could function as a fumarate reductase suggesting that fumarate could be used as an electron acceptor in the absence of oxygen.

### Adaptation to Hypoxia by *M. smegmatis* Results in the Upregulation of Oxygen Scavenging Oxidases, Low Redox Potential Electron Carriers and Hydrogenases

All mycobacteria species are obligate aerobes and are therefore faced with a number of energetic challenges as the cells encounter hypoxic conditions (e.g. recycling of reducing equivalents). Our data indicates that *M. smegmatis* adopts three strategies to metabolize under hypoxia. Firstly, the cell induces the high-affinity (oxygen scavenging) cytochrome *bd* oxidase and machinery needed for its assembly and synthesis (i.e. porphyrin/heme and cysteine biosynthesis). Secondly, it switches to NAD^+^/NADH-independent enzymes, especially those with ferredoxins as a redox partner. Thirdly, soluble hydrogenases are upregulated to either oxidize hydrogen or produce hydrogen, or both activities operate in concert with other energetic partners.

The high-affinity cytochrome *bd* oxidase is able to scavenge oxygen under microaerobic conditions and by operating in concert with menaquinone oxidoreductases (*ndh2*) and the proton-translocating cytochrome *bc_1_-aa_3_* supercomplex would result in the formation of an electrical potential at low oxygen tensions. During *M. tuberculosis* infection of macrophages, the cytochrome *bc_1_-aa_3_* complex is downregulated, but this is based on a relative comparison with broth cultures [13] and therefore this most likely reflects the change in growth rate and not oxygen *per se*. Our results also show a downregulation of the terminal oxidases in response to slow growth rate, but an upregulation (relative to aerobic slow growth) when switched to hypoxia. Upregulation of cytochrome *bd* in mycobacteria has been observed *in vitro* [14] and *in vivo* [15], however the designated role and the regulator(s) of this enzyme are still unknown. In *Streptomyces coelicor*, a transcriptional repressor of cytochrome *bd* was identified and named Rex [16]. This NADH/NAD^+^ redox sensor regulates the expression of *cydABDC* as well as the *hemACDB* operon [16]. No homologue of this protein has been found in mycobacterial species, however the simultaneous upregulation of both operons observed in our study could point to a common regulatory protein.

A novel, but yet unexplained observation is the red pigmentation observed in hypoxic cultures (Figure 3A&B). This could be due to the increased expression of heme containing cytochromes, which is supported by our expression data showing strong upregulation of heme/porphyrin, cytochromes and cysteine biosynthesis pathways as well as a potential heme transporter (CssAB) [17] (Figure 3A). It has been suggested that the presence of porphyrins is the main reason for the slightly red color in Mycobacteria, because the absence of porphyrins in iron-deficient grown cells has been reported to account for their very pale appearance [18].

An unanswered question in the physiology and metabolism of mycobacteria under hypoxia is how cells recycle reducing equivalents (e.g. NADH) in the lack of exogenous electron acceptors and maintain metabolic flux through the TCA cycle. NADH-producing enzymes commonly found in the TCA cycle of bacteria (e.g. α-ketoglutarate dehydrogenase and malate dehydrogenase) are absent in the genome of *M. smegmatis*. Instead, these enzymes are replaced with a malate:quinone oxidoreductase (MQO) and a putative α-ketoglutarate:ferredoxin oxidoreductase (KOR) and/or pyruvate:ferredoxin oxidoreductase (POR), which reduce menaquinone or ferredoxin, respectively. It has been suggested that MQO in *C. glutamicum* allows cells to attain a higher TCA cycle flux independently of the NADH/NAD^+^ ratio [19]. In *E. coli* MQO is regulated by the oxygen limitation sensor ArcBA [20]. The gene *msmeg_4645* is annotated as KOR and its neighbouring gene, *msmeg_4646*, encodes a protein with a POR family domain. This raises the possibility that this gene pair encodes for an enzyme that is able to metabolize both α-ketoglutarate and pyruvate under hypoxia. Support for this idea comes from a recent report demonstrating that *M. tuberculosis* does indeed express an “anaerobic type” α-ketoglutarate:ferredoxin oxidoreductase (KOR) (*Rv2454c/Rv2455c*) and a mutant defective in this enzyme is impaired for aerobic growth in the absence of CO_2_ [21]. The strategy of substituting NAD^+^-dependent with NAD^+^-independent enzymes ensures functionality of the TCA cycle under hypoxia and this appears to apply to other metabolic pathways. We observed the induction of several ferredoxin-reducing and -oxidizing enzymes: Ni-Fe hydrogenases, sulfite reductase, ferredoxin–NADP^+^ reductase, several cytochrome P450s (CYP) and for ferredoxin itself. This indicates that under hypoxic conditions, *M. smegmatis* is dependent on the alternative electron carrier ferredoxin, a feature only reported for anaerobic bacteria. Recently, it was shown that a ferredoxin-dependent CYP is essential in *M. tuberculosis* [22]. Notwithstanding this, NADH recycling remains important as demonstrated by the induction of glycine dehydrogenase, an enzyme that produces NAD^+^ [23], [24].

*M. smegmatis* has assembled a large arsenal of tools to degrade complex substrates and to adapt to large fluctuations in energy and oxygen supply. This metabolic plasticity allows the bacterium to survive in diverse environmental compartments, such as different soils, water distribution systems [25] and under human skin [26]. *M. smegmatis* harbors gene clusters encoding for three putative soluble hydrogenases that are differentially regulated in response to growth rate and hypoxia. Hydrogenases catalyze the reversible reaction: 2H^+^ + 2e^−^ ⇔ H_2_ and represent the ideal mechanism to deal with fluctuations in energy and oxygen supply [27]. Under energy limiting conditions, hydrogenases can scavenge hydrogen as an energy source and under hypoxia, hydrogen can be produced as a redox sink and ultimately re-utilized by hydrogenases when the intracellular carriers become less reduced as observed for many fermentative organisms [28]. To validate this proposal, we disrupted *msmeg_2719* and this mutant exhibited a significantly reduced growth yield compared to the wild type, indicating an essential role of hydrogenases in the adaptation of Mycobacteria to low energy environments. It has been shown that *M. smegmatis,* among other mycobacterial species, can oxidize molecular hydrogen in the presence of carbon monoxide implying that they indeed express a functional hydrogenase [29]. However, in our experiment no additional hydrogen was added so the only hydrogen available could be scavenged from air or produced by the cells themselves. We could not detect H_2_ gas production in either batch or continuous growth and this data would support the notion that hydrogen is rapidly recycled in *M. smegmatis*, as it has been observed in *Salmonella typhimurium* [30].

*M. tuberculosis* harbours a single gene cluster for a putative membrane-bound hydrogenase complex and this complex has been shown to be upregulated during *M. tuberculosis* infection of human macrophage-like cells [31]. Moreover, the transcription of the first genes of the *hycP/hycQ* - containing operon was shown to be upregulated during anaerobic adaptation in several studies and is thought to be part of the DevR/DevS regulon [4], [6], [9], [32], [33]. Boshoff & Barry (2005) hypothesize that the *hyc* operon in *M. tuberculosis* is regulated by other signals, in addition to low oxygen tension, because it was upregulated in resting as well as activated murine bone marrow macrophages [13]. Both *hycP* and *hycQ* (encoding for a possible hydrogenase components) were shown to be essential for optimal growth [34]. The putative hydrogenase gene cluster of *M. tuberculosis* shows homology to components of hydrogenase 4 and 3 complex of *Escherichia coli*, the latter of which can catalyze hydrogen evolution at acidic pH [35]. It is tempting to argue that the acidic, hypoxic and lipid-rich environment in the macrophages might require the expression of a hydrogenase to help with the recycling of reducing equivalents under these conditions. Alternatively, hydrogen could serve as an additional means of energy in the host, a much better energy source than host lipids. The use of hydrogen as an electron donor for respiration in the host by pathogenic bacteria is not unprecedented [36], [37], [38], but has been overlooked in the pathogenesis of mycobacteria.

In summary, we propose that mycobacteria adapt to slow growth rate and hypoxia using a battery of strategies that involve the use of alternative primary dehydrogenases and hydrogenases to maintain the flow of reducing equivalents to the electron transport chain during conditions of energy limitation. When the cell is additionally confronted with hypoxia, mycobacteria adapt further by switching to hydrogenases as a potential conduit for disposing of electrons in the absence of exogenous electron acceptors, and unique electron carriers (ferredoxins) that are more akin to those employed by anaerobic bacteria. These data demonstrate the incredible metabolic diversity that mycobacteria possess and provides a framework for understanding their ability to survive for prolonged periods of time under low energy conditions and hypoxia. The regulators and signaling pathways required for this extraordinary adaptive ability remains to be uncovered, but are clearly unique.

## Materials and Methods

### Strains and Media

*Mycobacterium smegmatis* strain mc^2^155 [39] was used in all experiments. For batch culture experiments, either Middlebrook 7H9 medium (Difco) or Luria-Bertani broth (diluted 3×) both supplemented with 0.05% (w/v) TWEEN 80 was used. For continuous cultivation, Middlebrook 7H9 was supplemented with Na_2_HPO_4_ (1.25 gL^−1^) and KH_2_PO_4_ (0.5 gL^−1^) to buffer the growth medium at pH 6.6±0.2 for high bacterial concentrations (OD_600_≈5). The medium contained 27 mM glycerol, 0.1% antifoam and 0.4% TWEEN 80.

### DNA Manipulation and Cloning of Constructs

All molecular biology techniques were carried out according to standard procedures [40]. Restriction or DNA modifying enzymes and other molecular biology reagents were obtained from Roche Diagnostics or New England Biolabs. Genomic DNA of *M. smegmatis* was isolated as described previously [41]. All primer sequences are listed in Table S2. To create a markerless deletion of *msmeg_2719*, a 1058 bp fragment flanking *msmeg_2719* on the left, including 208 bp coding sequence, was amplified with primers Hyd2.1 and Hyd2.2 and a 985 bp fragment flanking *msmeg_2719* on the right, including 136 bp coding sequence, was amplified with primers Hyd2.3 and Hyd2.4. The two products were fused by PCR-overlap extension [42], cloned into the SpeI site of the pPR23-derived [43] vector pX33 [41], creating pHyd2KO, and transformed into *M. smegmatis* mc2155. Deletion of *msmeg_2719* was carried out using the two-step method for integration and excision of the plasmid as described previously [44]. Correct integration and excision were confirmed by Southern hybridization analysis (Figure S2) as described previously [41]. The deletion resulted in loss of 82% of the *msmeg_2719* coding sequence, creating strain Δ2719. For complementation of the *msmeg_2719* deletion, the operon *msmeg_2720-2718*, including 72 bp downstream and 413 bp upstream DNA, was PCR amplified with primers cHyd2f and cHyd2r and cloned as a HindIII fragment into the integrative *E. coli*/mycobacteria shuttle vector pOLYG [45], creating plasmid pOLYHyd2. This construct was transformed into the Δ2719 strain to yield strain Δ2719_pOLYHyd2. Empty pOLYG plasmid was transformed into the wild type as well as the Δ2719 strain to yield strain wt_pOLYG and Δ2719_pOLYG respectively.

### Batch and Continuous Culture

Prior to each experiment, bacteria were inoculated from a cryo-culture (−80°C) onto a LBT plate and incubated for 3 days at 37°C. A single colony was picked from the plate and inoculated into the 5 ml batch medium (LBT or 7H9) in a 30 ml glass universal and incubated at 37°C on a rotary shaker at 200 rpm. At an OD_600_ between 0.3 − 0.5, a sample was withdrawn to inoculate the bioreactor (700 ml) or batch culture (500 ml Erlenmeyer flask containing 50 LBT medium) to a starting OD_600_ of 0.005. The flasks were incubated at 37°C on a rotary shaker at 200 rpm. Before inoculation, the culture medium in the bioreactor was stirred at 320 rpm at 37°C for at least 10 minutes to fully saturate the water with oxygen. This corresponded approximately to an O_2_ concentration of 6.9 mg/L. The oxygen probe was then set to 100%. After inoculation, the culture was left in batch mode maintaining 50% oxygen saturation until OD_600_ reached around 80% of steady-state OD_600_ and then switched to chemostat mode either at a dilution rate of 0.15 h^−1^ or 0.01 h^−1^ corresponding to doubling times of 4.6 h and 69 h respectively. The chemostat was then left running for at least two volume changes before cell harvest. Glycerol concentration was measured according to Garland & Randle [46].

Cells were harvested into cold glycerol saline [47]. In short, 50 ml of culture was extracted with a syringe. Four centrifugation tubes filled with 20 ml of cold glycerol saline (3:2) (−20°C) were then mixed with 10 ml of culture by vigorous shaking and immediately placed into the centrifuge. Samples were centrifuged for 20 min at 27,200 g at −20°C in a Beckman J2-21M/E with a JA-20 rotor. After centrifugation, the supernatant was removed, the cell pellet resuspended in glycerol saline (1∶1) (−20°C) transferred to a 1.5 ml Eppendorf tube and snap frozen in a dry ice ethanol bath. The samples were stored at −80°C until further use.

For the analysis of gene expression at low oxygen concentration, five individual chemostats were performed at the low dilution rate (D = 0.01 h^−1^) with 2.5% oxygen saturation and another five with 0.6% oxygen saturation. These chemostats were first brought into steady state at 50% oxygen saturation (as described above) before lowering the oxygen concentration slowly over two consecutive days. All other parameters were left unchanged. At an oxygen saturation of 0.6% the chemostat was truly oxygen-limited. The growth rate of the culture was 0.008±0.0005 h^−1^.

### Fluorescence Microscopy

Bacterial cell samples were withdrawn from the chemostat and 100 µl stained with 1 µl of SYBRGreen I stain. After 15 min incubation the sample was analyzed by epifluorescence microscopy (Olympus BX51) equipped with a 100× magnification lens and a bandpass filter at 520±30 nm.

### RNA Extraction

Total RNA was extracted using TRIZOL® reagent (Invitrogen) according to the manufacturer's instructions. Cell lysis was achieved by three cycles of bead-beating in a Mini-Beadbeater (Biospec) at 5,000 rpm for 30 sec. DNA was removed from the RNA preparation by treatment with 2 U RNase-free DNase using the TURBO DNA-*free* kit (Ambion), according to the manufacturer's instructions. RNA concentrations were determined using a NanoDrop® ND-1000 spectrophotometer and the quality of RNA checked on a 1.2% agarose gel.

### Quantitative Real-Time PCR

cDNA was synthesized from 1 µg of RNA for each sample with the SuperScript III reverse transcriptase kit (Invitrogen). After cDNA synthesis, real-time PCR was conducted according to the manufacturer with Platinum®SYBR®Green qPCR SuperMix-UDG with ROX (Invitrogen). Primers (Invitrogen) for 10 genes (Table S3) were designed with the publicly available Primer 3 software. Primer optimization was performed. The real-time PCR reactions were conducted in ABI Prism®7500 (Applied Biosystems) and the results were normalized to the gene *sigA* (*msmeg_2758*) as endogenous control (Figure S3).

### Microarray Analysis

Microarray analysis was performed using arrays provided by the Pathogen Functional Genomics Research Center (PFGRC) funded by the National Institute of Allergy and Infectious Diseases using protocols SOP# M007 and M008 from The Institute of Genomic Research (TIGR) [48]. Ten µg of total RNA was used to create cDNA labeled with aminoallyl dUTP (Sigma). Fluorescent Cy3 and Cy5 dyes (GE Healthcare) were then covalently attached to the aminoallyl tags. Each pair of differentially labeled probes was resuspended in 60 µl of hybridization buffer (500 µl formamide, 250 µl 20× SSC, 5 µl 10% SDS, 245 µl ultrapure water) and hybridized to the microarray slide overnight in a 42°C water bath. Slides were then washed in increasingly stringent wash conditions (3 times 5 min 1× SSC 0.1% SDS, 3 times 5 min 0.1× SSC 0.1% SDS, 3 times 5 min 0.1× SSC and a final dip in 0.22 µm-filtered MQ water). Arrays were scanned in a Genepix 4000A scanner and spots quantified with TIGR Spotfinder. The data was then processed in TIGR MIDAS. Two color tiff images of the microarrays were visually inspected for defects and obvious spatial biases. Prior to normalization pin-tip (block) intensity box plots were used to detect slides exhibiting spatial and/or pin biases, with post-normalization intensity box plots and MA plots used to assess the effectiveness of normalization in correcting these biases. RI plots were also used to identify outlier slides and for assessing the effectiveness of the normalization process, and Z-score histograms were used to look for slides with abnormal intensity distributions. Slides that did not pass the quality control process were rejected, and repeat hybridizations were performed. Post normalization (Total Intensity and LOWESS) the in-slide replicate spots were averaged before expression ratios were calculated. The results from 5 biological replicates including two dye swaps were then subjected to a t-test without false discovery correction in TIGR MeV software. The analysis was used as a ranking method. For a general overview, genes with expression ratios of >2 and<0.5 and a p-value<0.05 were used for data interpretation. In a second step data was extracted for all genes involved in energy metabolism. In all tables, genes with a p-value of<0.05 are marked with a grey background. Expression ratios and p-values for all genes are listed in Dataset S1. All data has been deposited at Gene Expression Omnibus (GEO, NCBI) with the accession number GSE17520.

### Gas Chromatography

Gaseous end products were analyzed by gas chromatography by using a HP 5890 Series II gas chromatograph (Hewlett Packard Co., California, USA) equipped with a thermal conductivity detector and Carbosphere 80/100 column (Alltech, Illinois, USA). Nitrogen was used as the carrier gas at a pressure of 50 psi and a flow rate of 30 ml/min. Oven and detector temperature were 150°C and 180°C respectively.

### Phylogenetic Analysis

For phylogenetic analysis, the amino acid sequences of the proteins of interest were compared to the database of MicrobesOnline (http://www.microbesonline.org/) using FastBLAST. For proteins with more than 40% identity, the amino acid sequence was downloaded and all the sequences of interest were aligned using the Clustal W method in MegAlign (DNASTAR, Lasergene) software. The alignment was then transferred to TreeView X software and depicted as a Phylogram.

## Supporting Information

Dataset S1 (0.96 MB PDF)

Table S1 Expression ratios of genes involved in energy metabolism. Expression ratios with a p-value<0.05 are indicated with bold numbers.
(0.22 MB DOC)

Table S2 Primer sequences used for quantitative RT-PCR.
(0.05 MB DOC)

Table S3 Primer sequences used for deletion of msmeg_2719 and complementation.
(0.03 MB DOC)

Figure S1 Phylogenetic trees of the large subunits of the three hydrogenases found in the genome of *M. smegmatis* mc2155. Proteins with >40% identity are depicted.
(3.47 MB TIF)

Figure S2 Construction of an unmarked msmeg_2719 deletion mutant of *M. smegmatis* mc2155. A. Schematic diagram of the two-step approach for deletion of msmeg_2719. The knockout construct consisted of two fragments flanking msmeg_2719 on the left (LF) and right (RF) in PX33. Integration of the vector (thick black line) into the chromosome (thin black line) via the left flank (Int LF) or right flank (Int RF) and subsequent deletion of msmeg_2719 (KO) are shown. Restriction sites of SmaI (S) and fragment sizes as detected in Southern hybridization are indicated. Drawing not to scale. WT, wild-type. B. Southern hybridization analysis of the integration event. Left panel, 1. crossover: SmaI-digests of genomic DNA of wild-type mc2155 (lane 1) and a candidate colony (lane 2) were probed with radiolabeled left flank PCR product of the deletion construct. Right panel, 2. crossover: Southern hybridization of msmeg_2719 deletion. Analysis of msmeg_2719 deletion strain (line 1) and wild-type mc2155 (lane 2) was performed as in the left panel. Molecular masses are indicated in kb. M, marker.
(3.30 MB TIF)

Figure S3 Validation of *M. smegmatis* gene expression ratios (slow growth rate at 50% oxygen saturation versus fast growth rate at 50% oxygen saturation) by quantitative RT-PCR (qPCR) (grey bars) compared to microarray results (black bars). For qPCR the expression of each gene was normalized to the expression of sigA. Error bars represent standard deviations of gene expression ratios from three biological replicates for each condition.
(5.48 MB TIF)
