# Supplementary material for: Unique Flexibility in Energy Metabolism Allows Mycobacteria to Combat Starvation and Hypoxia
Source: PLoS One. 2010 Jan 7;5(1):e8614. doi: 10.1371/journal.pone.0008614 (PMC2799521; doi:10.1371/journal.pone.0008614)
Supplement: Dataset S1 — (0.96 MB PDF) [file pone.0008614.s001.pdf]

Summary all arrays

Locus	GeneN	GeneS	Slow vs. Fast growth<br>Condition A: T_d=69h, 50% air<br>Condition B: T_d=4.6h, 50% air<br>Expression ratio Condition A/B	p value	Slow growth 2.5% air<br>Condition A: T_d=69h, 2.5% air<br>Condition B: T_d=69h, 50% air<br>Expression ratio Condition A/B	p value	Slow growth 0.6% air<br>Condition A: T_d=69h, 0.6% air<br>Condition B: T_d=69h, 50% air<br>Expression ratio Condition A/B	p value
MSMEG_0001	DNA polymerase III, beta subunit	*dnaN*	0.89	5.69E-02	0.99	3.63E-01	1.09	3.47E-01
MSMEG_0002	6-phosphogluconate dehydrogenase, decarboxylating		0.83	6.10E-02	0.91	2.34E-01	1.20	1.35E-01
MSMEG_0003	DNA replication and repair protein RecF		0.86	2.19E-02	0.91	2.52E-01	1.76	1.05E-01
MSMEG_0004	hypothetical protein		0.97	5.01E-01	0.95	5.63E-01	1.59	1.52E-02
MSMEG_0005	DNA gyrase, B subunit	*gyrB*	0.78	1.48E-03	0.91	1.03E-01	0.91	3.67E-01
MSMEG_0006	DNA gyrase, A subunit	*gyrA*	0.46	2.13E-05	1.03	7.05E-01	2.75	1.15E-04
MSMEG_0007	conserved hypothetical protein		0.67	1.17E-04	0.86	6.82E-02	1.65	9.43E-02
MSMEG_0009	hypothetical protein		1.47	6.20E-04	0.82	9.65E-02	1.31	2.28E-01
MSMEG_0011	FAD-binding 9, siderophore-interacting		0.84	6.47E-02	1.16	3.21E-01	1.08	7.28E-01
MSMEG_0012	ferric enterobactin transport system permease protein FepD		0.54	3.02E-02	1.75	3.00E-01	1.72	1.46E-01
MSMEG_0013	ferric enterobactin transport system permease protein FepG; this gene contains a frame shift which is not the result of sequencing error; identified by match to protein family HMM		0.60	5.94E-03	1.28	1.47E-01	1.43	1.66E-01
MSMEG_0014	Formyl transferase		1.29	1.65E-01	0.60	3.95E-02	0.88	2.75E-01
MSMEG_0015	ferric enterobactin transport ATP-binding protein FepC		1.40	1.86E-01	0.69	1.15E-02	1.11	5.31E-01
MSMEG_0016	conserved domain protein		0.90	8.95E-02	0.83	6.38E-02	0.89	3.07E-01
MSMEG_0017	ABC transporter, permease/ATP-binding protein		0.75	8.80E-02	1.34	8.29E-02	1.19	4.33E-01
MSMEG_0018	ABC transporter, permease/ATP-binding protein		0.84	4.30E-02	1.21	1.61E-01	1.10	6.70E-01
MSMEG_0019	amino acid adenylation		0.88	1.60E-01	1.03	8.26E-01	0.91	1.77E-01
MSMEG_0020	Periplasmic binding protein		0.60	3.51E-04	0.77	7.20E-03	0.11	1.11E-06
MSMEG_0021	aspartate 1-decarboxylase	*panD*	0.77	7.30E-03	1.00	9.87E-01	0.39	6.85E-03
MSMEG_0022	L-ornithine 5-monooxygenase		0.53	2.09E-03	1.05	6.91E-01	0.32	2.30E-03
MSMEG_0023	conserved hypothetical protein		1.07	4.72E-01	0.96	6.98E-01	1.21	1.91E-01
MSMEG_0024	peptidyl-prolyl cis-trans isomerase B		0.50	1.62E-04	0.76	8.13E-02	2.34	4.86E-02
MSMEG_0025	conserved hypothetical protein		1.02	3.70E-01	0.99	7.78E-01	1.03	4.86E-01
MSMEG_0026	putative membrane protein		0.89	4.07E-02	1.02	4.70E-01	1.18	9.58E-03

MSMEG_0027	conserved hypothetical protein		1.02	8.67E-01	1.11	6.71E-02	0.94	3.92E-01
MSMEG_0028	serine-threonine protein kinase		0.85	1.29E-01	0.94	3.58E-01	1.24	1.86E-01
MSMEG_0029	anthranilate synthase component 2		0.96	8.09E-03	0.80	6.65E-02	0.66	9.58E-03
MSMEG_0030	serine/threonine protein kinase PknA	*pknA*	0.69	2.37E-03	1.30	9.47E-02	1.22	4.04E-02
MSMEG_0031	Penicillin binding protein transpeptidase domain protein		1.02	5.74E-01	1.15	1.64E-01	1.25	9.69E-03
MSMEG_0032	cell cycle protein, FtsW/RodA/SpoVE family protein		0.79	1.78E-02	1.28	5.71E-02	1.25	2.90E-01
MSMEG_0033	protein phosphatase 2C		0.70	3.60E-04	1.00	9.84E-01	1.31	3.13E-01
MSMEG_0034	FHA domain protein		0.79	5.52E-02	1.20	1.25E-01	1.13	2.67E-01
MSMEG_0035	FHA domain protein		1.21	3.51E-02	1.13	1.02E-01	0.56	9.21E-04
MSMEG_0036	hypothetical protein		1.12	3.93E-01	2.39	8.81E-02	1.14	4.67E-01
MSMEG_0038	putative glucose-methanol-choline oxidoreductase; this region contains one or more premature stops and/or frameshifts which are not the result of sequencing error		1.15	1.58E-01	0.86	3.26E-01	1.35	4.65E-01
MSMEG_0039	small membrane hydrophobic protein		1.87	1.59E-01	1.76	1.55E-01	0.96	6.23E-01
MSMEG_0040	conserved hypothetical protein		3.65	1.67E-01	3.48	1.75E-01	1.02	8.28E-01
MSMEG_0041	conserved hypothetical protein		3.97	2.61E-01	4.19	2.23E-01	1.07	3.50E-01
MSMEG_0042	TetR-family protein transcriptional regulator		0.95	3.63E-01	0.91	1.93E-01	1.86	4.29E-01
MSMEG_0043	extradiol ring-cleavage dioxygenase, class III enzyme, subunit B		1.08	3.63E-01	3.27	9.68E-02	2.17	7.93E-03
MSMEG_0044	hypothetical protein		0.95	6.16E-01	1.06	6.39E-01	2.89	1.72E-04
MSMEG_0045	hypothetical protein		0.82	2.75E-03	0.93	5.77E-01	2.01	9.08E-03
MSMEG_0046	conserved hypothetical protein		0.82	1.51E-02	0.92	1.83E-01	1.14	1.80E-01
MSMEG_0047	hypothetical protein		0.57	1.66E-04	5.42	9.75E-02	0.75	6.00E-04
MSMEG_0048	pyridoxamine 5'-phosphate oxidase family		0.57	4.34E-03	1.02	8.36E-01	0.74	2.80E-01
MSMEG_0049	pirin domain protein		0.98	8.79E-01	8.70	1.11E-01	1.19	6.51E-01
MSMEG_0050	conserved hypothetical protein		1.04	5.11E-01	1.16	4.68E-01	0.90	4.96E-01
MSMEG_0051	transcription factor WhiB family protein		1.81	8.75E-02	0.62	8.86E-02	0.81	5.75E-01
MSMEG_0052	conserved hypothetical protein		0.94	3.12E-01	0.83	4.13E-02	0.90	5.06E-01
MSMEG_0053	conserved hypothetical protein		0.68	4.13E-03	0.82	6.21E-02	0.64	3.70E-03
MSMEG_0053	conserved hypothetical protein		1.09	3.53E-01	1.21	1.40E-01	0.82	9.28E-02
MSMEG_0055	hypothetical protein		0.83	3.68E-02	1.05	2.34E-01	0.65	6.89E-02
MSMEG_0056	hypothetical protein		1.04	7.60E-01	1.17	5.69E-02	0.66	2.98E-02
MSMEG_0057	conserved hypothetical protein		0.85	3.75E-02	0.95	3.57E-01	0.75	1.22E-01
MSMEG_0058	conserved hypothetical protein		0.84	2.40E-03	0.89	8.78E-02	1.12	4.69E-01
MSMEG_0059	ATPase, AAA family protein		0.94	4.09E-01	0.87	1.01E-01	0.94	7.68E-01

MSMEG_0060	conserved hypothetical protein	0.70	5.51E-04	0.99	8.67E-01	0.78	3.81E-02
MSMEG_0061	ftsk/spoiiie family protein	0.85	1.54E-02	0.92	1.64E-01	0.63	6.18E-03
MSMEG_0062	ftsk/spoiiie family protein	1.32	9.24E-03	0.92	4.01E-01	0.49	4.73E-03
MSMEG_0063	PE family protein	0.87	1.36E-01	1.42	1.63E-01	0.30	3.68E-04
MSMEG_0064	ppe family protein	0.79	2.47E-02	1.18	2.64E-01	0.48	2.64E-03
MSMEG_0065	conserved hypothetical protein	0.68	5.11E-05	1.10	2.41E-01	0.27	7.73E-04
MSMEG_0066	early secretory antigenic target, 6 kDa	1.01	9.17E-01	1.22	1.82E-01	0.35	1.71E-04
MSMEG_0067	conserved hypothetical protein	1.00	9.78E-01	1.11	3.99E-01	0.26	7.24E-05
MSMEG_0068	probable conserved transmembrane protein	0.74	1.24E-02	0.93	4.73E-01	0.79	4.14E-01
MSMEG_0069	translation initiation factor IF-2 protein	0.83	8.95E-02	1.13	3.28E-01	0.95	4.22E-01
MSMEG_0070	hypothetical protein	1.08	4.09E-01	0.89	1.27E-02	1.14	7.11E-01
MSMEG_0071	conserved hypothetical protein	0.76	1.29E-02	0.78	7.90E-03	0.74	2.42E-02
MSMEG_0071	conserved hypothetical protein	1.02	5.37E-01	1.03	6.86E-01	1.29	3.28E-01
MSMEG_0076	antigen MTB48	0.97	6.25E-01	0.79	1.11E-02	0.49	6.79E-03
MSMEG_0077	hypothetical protein	1.22	1.59E-02	0.79	1.29E-01	0.45	3.60E-05
MSMEG_0078	hypothetical protein	0.89	1.18E-01	0.82	1.15E-01	0.54	1.26E-02
MSMEG_0079	hypothetical protein	0.63	6.53E-03	1.10	1.70E-01	0.87	2.54E-01
MSMEG_0080	conserved hypothetical protein	0.52	8.52E-04	1.12	2.84E-01	0.87	4.31E-01
MSMEG_0081	conserved hypothetical protein	0.76	9.71E-03	1.20	3.89E-02	0.51	1.33E-02
MSMEG_0082	conserved hypothetical protein	0.52	2.82E-04	1.02	5.62E-01	1.51	6.38E-02
MSMEG_0083	membrane-anchored mycosin mycp1	0.73	6.70E-03	1.16	2.23E-01	0.58	6.29E-05
MSMEG_0084	phosphocarrier protein hpr	1.00	9.46E-01	0.86	2.23E-01	0.87	7.27E-01
MSMEG_0085	PTS system, Fru family protein, IIABC components	1.10	4.01E-01	1.34	1.63E-01	0.70	1.24E-01
MSMEG_0086	1-phosphofructokinase	0.96	7.45E-01	3.73	3.22E-01	0.87	2.85E-01
MSMEG_0087	glucitol operon repressor	1.31	2.25E-01	1.14	1.92E-01	1.18	7.18E-01
MSMEG_0088	phosphoenolpyruvate-protein phosphotransferase *ptsI*	1.16	3.09E-01	1.06	4.96E-01	0.73	1.15E-01
MSMEG_0089	chromosome condensation protein	2.26	4.71E-02	1.80	1.23E-01	1.05	6.23E-01
MSMEG_0090	conserved hypothetical protein	1.22	1.32E-02	2.38	6.94E-02	0.87	3.73E-01
MSMEG_0091	putative transcriptional regulator	1.25	3.31E-02	1.30	4.87E-02	1.08	4.15E-01
MSMEG_0092	probable transcriptional regulatory protein	1.67	3.58E-02	1.00	9.83E-01	0.26	1.95E-04
MSMEG_0093	methyltransferase	0.28	2.63E-05	1.27	8.33E-02	4.88	7.64E-02
MSMEG_0094	hypothetical protein	1.14	4.48E-01	6.49	1.25E-01	0.94	7.65E-01
MSMEG_0095	methyltransferase	0.35	1.17E-04	1.08	6.50E-01	4.28	4.35E-02
MSMEG_0096	peroxisomal hydratase-dehydrogenase-epimerase	0.43	1.21E-03	0.84	1.88E-01	1.57	7.38E-02
MSMEG_0097	oxidoreductase, zinc-binding dehydrogenase family protein	0.54	2.06E-03	1.05	6.94E-01	2.00	7.78E-02
MSMEG_0098	methyltransferase	0.62	1.32E-03	0.99	9.24E-01	1.74	1.24E-01

MSMEG_0099	conserved hypothetical protein		1.05	5.24E-01	1.21	2.53E-01	1.00	9.39E-01
MSMEG_0100	phosphotyrosine protein phosphatase ptpb		0.53	1.15E-04	0.87	1.52E-01	1.97	8.77E-03
MSMEG_0101	conserved hypothetical protein		1.29	4.71E-02	0.79	8.24E-04	1.20	2.67E-01
MSMEG_0102	putative acyl-CoA dehydrogenase		0.63	1.60E-03	1.02	6.88E-01	1.21	1.04E-01
MSMEG_0103	major facilitator family protein transporter		0.82	3.30E-01	1.12	4.81E-01	1.21	4.17E-01
MSMEG_0104	transcriptional regulator, LysR family protein		0.81	6.66E-02	1.19	1.58E-01	1.07	2.82E-01
MSMEG_0105	DNA-binding response regulator, LuxR family protein		1.35	1.28E-02	1.19	3.59E-02	0.66	6.63E-02
MSMEG_0106	sensory histidine kinase		0.99	9.42E-01	1.22	2.51E-01	0.94	2.05E-01
MSMEG_0107	cell envelope-related function transcriptional attenuator common domain		1.29	1.37E-02	1.01	8.79E-01	1.11	3.22E-02
MSMEG_0108	acyl-CoA dehydrogenase		0.43	1.10E-04	0.85	1.30E-01	0.95	2.02E-01
MSMEG_0109	NAD(P) transhydrogenase, beta subunit	*pntB*	0.49	8.86E-06	0.91	1.96E-01	0.83	3.61E-01
MSMEG_0110	NAD(P) transhydrogenase, alpha subunit	*pntA*	0.51	7.94E-05	0.91	4.29E-01	0.73	3.75E-02
MSMEG_0111	putative magnesium transport protein		0.64	1.24E-03	0.91	9.36E-02	0.97	9.20E-01
MSMEG_0112	hypothetical protein		1.19	2.86E-01	1.17	5.56E-01	1.34	3.90E-01
MSMEG_0113	taurine transport system permease protein TauC		0.65	4.29E-03	8.15	2.41E-01	3.59	2.68E-01
MSMEG_0114	extracellular solute-binding protein, family protein 3		0.45	4.75E-03	9.54	2.24E-01	10.07	1.21E-01
MSMEG_0115	haloacid dehalogenase, type II	*dehII*	1.92	4.99E-01	1.21	8.84E-02	1.09	2.28E-01
MSMEG_0116	taurine import ATP-binding protein TauB		0.90	4.31E-02	17.62	3.31E-01	10.61	2.05E-01
MSMEG_0117	hydrolase		0.96	6.85E-01	1.79	2.12E-01	0.89	2.38E-01
MSMEG_0118	conserved hypothetical protein		1.19	4.14E-01	1.08	2.51E-01	1.25	1.34E-01
MSMEG_0119	conserved hypothetical protein		1.66	1.05E-03	1.13	2.51E-01	0.85	3.07E-01
MSMEG_0120	probable transcriptional regulatory protein		1.13	7.99E-02	1.18	8.15E-02	1.96	1.31E-02
MSMEG_0121	rhamnolipids biosynthesis 3-oxoacyl-[acyl-carrier-protein] reductase		1.16	3.14E-02	1.01	9.35E-01	2.43	2.87E-02
MSMEG_0122	putative periplasmic solute-binding protein		2.48	2.95E-01	1.14	1.42E-01	0.85	2.36E-01
MSMEG_0123	hypothetical protein		0.96	7.76E-01	1.04	7.28E-01	0.94	4.09E-01
MSMEG_0124	transcriptional regulator, GntR family protein		0.97	8.33E-01	1.09	4.50E-01	1.35	1.38E-01
MSMEG_0125	integral membrane protein		1.42	2.34E-01	1.40	9.48E-02	1.10	4.14E-01
MSMEG_0126	mandelate racemase/muconate lactonizing		0.37	1.49E-04	1.60	3.40E-02	1.23	3.44E-01
MSMEG_0127	oxidoreductase, zinc-binding dehydrogenase family protein		1.20	1.21E-01	0.82	9.35E-02	1.65	6.59E-02
MSMEG_0128	thioesterase superfamily protein		1.34	1.46E-01	0.93	4.03E-02	1.31	3.10E-01
MSMEG_0129	cyclase/dehydrase family protein		0.61	4.67E-04	1.04	5.40E-01	1.06	7.51E-01
MSMEG_0130	transcriptional regulator, GntR family protein		1.02	4.90E-01	1.15	2.72E-01	0.79	1.57E-01
MSMEG_0131	AMP-binding enzyme, putative		1.05	6.20E-01	0.80	4.04E-03	0.15	5.88E-06

MSMEG_0132	conserved hypothetical protein		0.90	4.18E-01	0.92	4.49E-01	0.21	3.43E-05
MSMEG_0133	ABC-transporter integral membrane protein		1.06	7.35E-01	0.79	1.87E-02	0.17	2.09E-05
MSMEG_0134	virulence factor Mce family protein		0.79	6.71E-02	1.08	2.61E-02	0.21	2.35E-06
MSMEG_0135	virulence factor Mce family protein		0.70	1.78E-03	1.08	3.81E-01	0.23	4.71E-06
MSMEG_0136	virulence factor Mce family protein, putative		0.98	6.38E-01	1.07	8.05E-01	0.46	7.00E-03
MSMEG_0137	virulence factor mce family protein		0.83	8.36E-03	1.12	2.10E-01	0.22	7.20E-06
MSMEG_0138	virulence factor Mce family protein		0.72	8.98E-04	0.95	5.54E-01	0.22	3.69E-05
MSMEG_0139	mce-family protein mce1f		0.70	2.90E-03	0.92	3.35E-01	0.24	1.80E-04
MSMEG_0140	probable conserved mce associated membrane protein		0.82	4.34E-02	0.82	4.30E-02	0.82	1.06E-01
MSMEG_0141	probable conserved mce associated transmembrane protein		0.56	1.60E-04	0.95	4.33E-01	0.78	8.90E-02
MSMEG_0142	conserved hypothetical protein		0.53	5.51E-04	1.28	7.96E-02	1.09	7.36E-01
MSMEG_0143	probable conserved mce associated membrane protein		0.82	5.48E-03	1.20	3.67E-01	0.82	9.00E-02
MSMEG_0144	HNH endonuclease		1.00	9.95E-01	1.22	1.31E-01	1.66	3.34E-01
MSMEG_0145	conserved hypothetical protein		0.69	1.48E-01	1.04	8.23E-01	1.28	4.46E-01
MSMEG_0146	mosc domain protein		0.94	2.46E-01	0.78	8.23E-02	0.78	1.04E-02
MSMEG_0147	C-5 sterol desaturase		1.11	2.23E-02	0.74	9.39E-03	0.79	2.04E-01
MSMEG_0148	transcriptional regulator, TetR family protein		1.71	6.09E-02	0.79	7.11E-02	1.08	5.12E-01
MSMEG_0149	ThiC family protein		1.72	5.43E-02	1.03	7.88E-01	1.02	8.89E-01
MSMEG_0150	NAD(P) transhydrogenase beta subunit		1.67	1.45E-02	0.82	5.33E-04	0.87	1.92E-01
MSMEG_0151	PntAB protein		2.08	3.43E-02	0.81	7.16E-02	1.03	9.54E-01
MSMEG_0152	Alanine dehydrogenase/pyridine nucleotide transhydrogenase		1.66	3.18E-02	0.90	5.29E-01	0.75	1.08E-01
MSMEG_0153	2-dehydropantoate 2-reductase	*panE*	1.46	1.19E-02	1.09	5.40E-01	0.93	8.95E-01
MSMEG_0154	pyruvate kinase	*pyk*	1.51	4.40E-02	0.79	3.83E-02	0.27	2.22E-04
MSMEG_0155	transcriptional regulator		1.76	5.15E-02	0.87	2.80E-01	0.73	1.68E-01
MSMEG_0156	transcriptional regulator, LysR family protein		1.32	3.64E-02	0.89	2.54E-02	0.94	8.45E-01
MSMEG_0157	oxalyl-CoA decarboxylase		2.04	1.54E-01	0.97	8.35E-01	0.52	2.79E-02
MSMEG_0158	formyl-coenzyme A transferase		1.29	3.56E-01	0.80	3.59E-02	0.28	2.74E-03
MSMEG_0159	formate dehydrogenase, gamma subunit		0.62	2.14E-03	1.26	2.70E-02	0.99	9.15E-01
MSMEG_0160	formate dehydrogenase, beta subunit		1.46	2.36E-01	1.12	4.05E-01	1.05	7.10E-01
MSMEG_0161	formate dehydrogenase, alpha subunit		1.84	7.74E-02	1.22	4.31E-01	1.28	2.04E-01
MSMEG_0162	NAD-dependent formate dehydrogenase delta subunit		1.64	1.27E-01	0.98	9.38E-01	0.76	1.02E-01
MSMEG_0163	conserved hypothetical protein		1.46	2.94E-02	1.08	4.57E-01	0.91	1.52E-01
MSMEG_0164	transmembrane transport protein		1.19	1.41E-01	1.16	2.89E-01	0.90	5.44E-01

MSMEG_0165	formate dehydrogenase family protein accessory protein FdhD	*fdhD*	1.07	9.45E-02	1.08	7.43E-01	0.88	2.82E-01
MSMEG_0166	GntR-family protein transcriptional regulator		1.18	4.59E-03	0.94	3.38E-01	0.74	5.64E-03
MSMEG_0167	transmembrane transport protein		8.89	1.62E-02	0.82	2.91E-01	0.07	1.84E-07
MSMEG_0168	formyl-coenzyme A transferase		4.99	3.23E-02	0.77	1.06E-01	0.45	2.23E-02
MSMEG_0169	hypothetical protein		1.39	2.04E-02	0.57	1.75E-02	1.15	3.52E-01
MSMEG_0170	transmembrane transport protein		5.40	2.29E-04	0.67	2.02E-03	0.20	2.17E-05
MSMEG_0171	histone deacetylase superfamily protein		2.29	1.05E-02	0.66	5.90E-03	0.62	1.84E-02
MSMEG_0172	probable conserved transmembrane protein, putative		0.33	6.21E-04	0.72	3.61E-02	20.58	3.50E-02
MSMEG_0173	hypothetical protein		0.68	4.96E-02	0.98	8.10E-01	1.00	8.99E-01
MSMEG_0174	putative inner membrane protein		0.61	4.43E-03	0.88	6.55E-02	1.37	2.46E-01
MSMEG_0175	FAD dependent oxidoreductase, putative		0.96	7.57E-01	1.07	6.54E-01	1.08	5.21E-01
MSMEG_0176	glutamine ABC transporter periplasmic-binding protein		0.84	2.08E-01	0.95	1.69E-01	1.07	5.03E-01
MSMEG_0177	ABC polar amino acid family protein transporter, inner membrane subunit		1.08	8.38E-02	0.93	4.08E-01	1.06	1.11E-01
MSMEG_0178	ATP-binding protein		1.36	4.51E-01	1.20	3.39E-01	1.01	8.76E-01
MSMEG_0179	transcriptional regulator, GntR family protein		1.06	2.51E-01	0.90	6.96E-02	10.56	3.74E-01
MSMEG_0180	putative HTH-type transcriptional regulator		1.32	1.15E-02	1.21	3.62E-01	0.93	3.55E-01
MSMEG_0181	alpha-ketoglutarate-dependent taurine		0.97	7.35E-01	0.93	4.04E-01	1.22	5.38E-01
MSMEG_0182	epoxide hydrolase 1		1.81	1.12E-01	0.99	9.76E-01	1.51	2.41E-01
MSMEG_0183	conserved hypothetical protein		1.04	7.19E-01	1.17	1.02E-01	1.33	1.63E-01
MSMEG_0184	transferase		3.97	2.41E-02	0.60	2.07E-03	0.93	3.38E-01
MSMEG_0185	MmpL6 protein		1.07	4.80E-01	1.25	3.07E-01	1.10	3.11E-01
MSMEG_0186	MmpS2 protein		0.75	2.45E-02	2.93	7.37E-02	0.89	4.34E-01
MSMEG_0187	acetyltransferase		1.13	1.30E-01	0.79	6.49E-03	2.26	1.98E-01
MSMEG_0188	MarR-family protein transcriptional regulator		0.86	7.27E-02	1.05	7.70E-01	0.64	9.45E-02
MSMEG_0189	conserved hypothetical protein		1.72	5.60E-02	1.24	4.79E-01	1.01	7.25E-01
MSMEG_0190	integral membrane transport protein		1.37	2.60E-01	1.50	1.56E-01	0.97	3.85E-01
MSMEG_0191	BadF/BadG/BcrA/BcrD ATPase family protein		0.93	5.44E-01	0.97	7.11E-01	1.11	2.70E-01
MSMEG_0192	transcriptional regulator, RpiR family protein		1.74	9.70E-03	0.92	4.76E-01	0.95	2.33E-01
MSMEG_0193	putative glucokinase regulator		2.19	1.40E-01	0.89	2.04E-01	0.94	6.53E-01
MSMEG_0194	serine esterase, cutinase family protein		0.89	3.39E-01	1.03	6.63E-01	3.44	1.94E-02
MSMEG_0195	steroid monooxygenase		1.89	1.95E-02	0.91	2.44E-01	0.52	6.19E-03
MSMEG_0196	putative dehydrogenase		1.86	4.86E-02	0.87	6.47E-02	2.59	1.91E-01
MSMEG_0197	nudix hydrolase		1.09	3.87E-01	1.06	7.34E-01	2.12	3.43E-01
MSMEG_0198	acyl-CoA dehydrogenase, C- domain protein		1.22	4.79E-01	1.02	8.67E-01	1.10	3.43E-01

MSMEG_0199	aminoglycoside phosphotransferase; this gene contains a frame shift which is not the result of sequencing error; identified by match to protein family HMM PF03881		1.57	4.50E-02	0.75	1.19E-02	1.18	2.50E-01
MSMEG_0200	hypothetical protein		6.01	9.37E-04	1.24	2.64E-01	0.78	3.02E-01
MSMEG_0201	hypothetical protein		0.99	8.80E-01	1.00	9.89E-01	1.07	1.96E-01
MSMEG_0204	hypothetical protein		3.02	2.86E-01	1.62	2.50E-01	0.92	3.96E-01
MSMEG_0205	tetracenomycin polyketide synthesis hydroxylase TcmH		1.00	9.87E-01	1.05	9.16E-01	1.06	6.38E-01
MSMEG_0206	acyltransferase 3		2.03	2.33E-01	1.17	8.21E-02	2.80	1.01E-01
MSMEG_0207	MarR-family protein transcriptional regulator		1.01	9.03E-01	0.94	4.23E-01	1.00	9.82E-01
MSMEG_0208	ribonuclease		1.13	4.75E-01	1.06	6.69E-01	0.81	2.32E-01
MSMEG_0209	ribonuclease inhibitor		0.96	2.48E-01	0.97	8.46E-01	1.09	6.73E-01
MSMEG_0210	LprO protein		0.65	4.80E-02	1.40	4.05E-01	1.24	5.73E-01
MSMEG_0211	ABC transporter, ATP-binding protein		1.54	1.87E-03	0.84	1.16E-01	1.04	2.88E-01
MSMEG_0212	lyase		1.57	3.50E-04	0.82	2.41E-02	0.98	8.49E-01
MSMEG_0213	transcriptional regulator		1.56	5.95E-04	0.78	5.87E-02	0.81	5.69E-02
MSMEG_0214	L-sorbosone dehydrogenase		0.74	6.56E-02	0.84	7.98E-02	1.16	3.59E-01
MSMEG_0215	YhhW family protein		0.95	1.73E-01	0.95	1.92E-02	1.10	3.04E-01
MSMEG_0216	3-hydroxyacyl-CoA dehydrogenase		0.75	6.33E-03	1.09	5.36E-01	2.08	2.80E-03
MSMEG_0217	alcohol dehydrogenase B		0.80	6.61E-02	0.98	7.96E-01	0.90	2.04E-01
MSMEG_0218	3-demethylubiquinone-9 3-methyltransferase		2.42	3.10E-02	1.20	1.85E-01	0.71	1.39E-01
MSMEG_0219	RNA polymerase sigma-70 factor, family protein		3.44	9.07E-04	0.95	3.64E-01	0.34	2.39E-04
MSMEG_0220	monoglyceride lipase		0.41	1.94E-03	0.99	8.29E-01	0.79	3.26E-02
MSMEG_0221	secreted protein		0.46	1.09E-04	0.94	4.64E-01	0.68	1.45E-02
MSMEG_0222	conserved hypothetical protein		0.65	1.07E-02	0.90	3.25E-01	0.86	2.29E-01
MSMEG_0223	conserved hypothetical protein		0.81	6.17E-01	1.08	4.34E-01	1.03	1.98E-01
MSMEG_0224	O-methyltransferase MdmC		1.02	9.20E-01	0.84	6.64E-02	1.08	6.11E-01
MSMEG_0225	MmpL4 protein		0.62	8.81E-04	0.64	4.94E-03	1.09	5.53E-01
MSMEG_0226	MmpS5 protein		0.67	9.14E-04	0.73	1.98E-02	1.38	1.18E-01
MSMEG_0227	transcriptional regulator, TetR family protein		1.61	1.04E-02	0.58	8.02E-03	0.99	9.48E-01
MSMEG_0228	adenylate and Guanylate cyclase catalytic domain protein		1.39	2.21E-01	1.13	3.98E-01	1.16	3.07E-01
MSMEG_0229	dihydroxy-acid dehydratase	*ilvD*	0.67	7.30E-04	0.80	1.83E-01	1.53	1.28E-01
MSMEG_0230	conserved hypothetical protein		1.50	3.79E-03	0.83	1.75E-02	0.62	1.07E-02
MSMEG_0231	conserved hypothetical protein		1.45	9.05E-03	0.86	4.83E-02	2.38	1.08E-01
MSMEG_0232	sugar transporter family protein		0.58	6.01E-03	0.89	3.24E-01	1.14	6.21E-02
MSMEG_0233	lipoprotein Lpps		1.64	4.57E-04	0.82	1.07E-01	0.79	9.57E-04

MSMEG_0234	metallopeptidase		0.99	8.37E-01	1.06	4.28E-01	0.70	1.30E-02
MSMEG_0235	probable conserved membrane protein		0.38	1.97E-04	0.89	2.41E-01	0.99	6.44E-01
MSMEG_0236	putative conserved transmembrane protein		0.75	4.69E-02	0.97	6.22E-01	1.25	1.77E-01
MSMEG_0238	O-acetylhomoserine/O-acetylserine sulfhydrylase		0.47	3.56E-04	0.99	9.51E-01	0.72	1.57E-01
MSMEG_0239	O-acetylhomoserine/O-acetylserine sulfhydrylase		0.44	4.75E-05	0.94	7.34E-01	0.59	7.46E-02
MSMEG_0240	conserved hypothetical protein		2.33	1.48E-04	0.66	2.35E-02	0.55	3.26E-03
MSMEG_0241	MmpL11 protein		0.87	6.97E-02	0.86	8.03E-03	1.55	2.82E-02
MSMEG_0242	conserved hypothetical protein		0.80	1.48E-01	0.69	4.44E-03	1.14	3.57E-01
MSMEG_0243	conserved hypothetical protein		1.12	2.74E-01	0.83	2.55E-01	0.44	7.84E-04
MSMEG_0244	two component response transcriptional regulatory protein prra		0.71	1.83E-03	0.89	2.43E-01	0.62	2.08E-02
MSMEG_0245	probable conserved transmembrane protein		1.93	2.41E-04	0.72	4.93E-02	1.24	4.95E-02
MSMEG_0246	sensor-type histidine kinase PrrB		0.36	7.06E-06	1.09	3.16E-01	1.21	3.31E-01
MSMEG_0247	secreted peptidase		2.16	1.85E-04	0.67	9.79E-03	1.23	1.36E-01
MSMEG_0248	conserved hypothetical protein		3.18	1.48E-04	0.94	5.65E-01	1.55	1.15E-01
MSMEG_0249	integral membrane protein		1.09	7.96E-01	0.78	1.84E-02	1.14	5.62E-01
MSMEG_0250	membrane protein, MmpL family protein		1.10	3.45E-01	0.86	2.41E-01	0.93	7.04E-01
MSMEG_0251	conserved hypothetical protein		1.73	1.11E-02	0.80	4.34E-02	0.91	5.24E-01
MSMEG_0252	tRNA (guanine-N(7)-)-methyltransferase	*trmB*	1.33	1.00E-02	0.96	5.35E-01	0.67	3.82E-02
MSMEG_0253	conserved hypothetical protein		1.27	4.94E-02	0.76	1.59E-02	1.34	5.86E-02
MSMEG_0254	conserved hypothetical protein		0.92	6.98E-02	0.87	1.42E-01	1.45	1.42E-01
MSMEG_0255	phosphoenolpyruvate carboxykinase		1.15	3.82E-02	0.69	5.43E-03	0.93	7.18E-01
MSMEG_0256	conserved hypothetical protein		0.94	6.97E-01	0.99	9.77E-01	1.04	4.34E-01
MSMEG_0257	acyl-CoA synthase		2.02	1.33E-02	0.81	6.00E-02	0.33	3.17E-03
MSMEG_0258	oxidoreductase, short chain dehydrogenase/reductase family protein		1.64	1.25E-01	1.11	4.03E-01	0.90	4.12E-01
MSMEG_0259	enoyl-CoA hydratase/isomerase		1.67	1.29E-02	0.95	8.36E-01	0.90	1.93E-01
MSMEG_0260	DNA-binding protein		1.52	1.00E-03	0.92	3.14E-01	0.93	7.17E-01
MSMEG_0261	p40 protein		0.56	5.15E-04	1.03	7.13E-01	2.57	4.44E-03
MSMEG_0262	conserved hypothetical protein		0.41	8.43E-05	1.28	8.57E-02	2.45	3.80E-02
MSMEG_0263	amidohydrolase 3		0.43	1.99E-06	0.92	3.77E-01	1.65	3.77E-01
MSMEG_0264	transmembrane transport protein		0.47	9.45E-03	0.94	2.48E-01	1.14	2.85E-01
MSMEG_0265	uracil DNA glycosylase superfamily protein		1.27	2.09E-01	1.11	2.98E-01	1.30	2.23E-01
MSMEG_0266	arginine decarboxylase		1.24	4.13E-01	1.44	2.34E-02	0.81	9.73E-02
MSMEG_0267	esterase		0.98	9.50E-01	1.71	7.50E-02	0.81	6.01E-02
MSMEG_0268	transcriptional regulator, GntR family protein		1.02	5.73E-01	0.99	8.94E-01	1.18	2.85E-01
MSMEG_0269	3-oxoacyl-[acyl-carrier-protein] reductase		0.64	9.93E-05	1.01	9.64E-01	1.01	9.69E-01
MSMEG_0270	aminoglycoside phosphotransferase		0.75	1.55E-01	0.95	5.17E-01	1.03	9.12E-01

MSMEG_0271	conserved hypothetical protein		1.45	2.32E-01	0.78	2.98E-02	0.93	4.32E-01
MSMEG_0272	propanediol utilization protein PduA		1.42	5.67E-02	1.04	7.94E-01	0.99	7.06E-01
MSMEG_0273	ethanolamine utilization protein EutN		1.62	5.20E-02	1.01	8.83E-01	1.21	5.26E-01
MSMEG_0274	hypothetical protein		0.98	8.08E-01	1.03	8.60E-01	1.14	3.28E-01
MSMEG_0275	bacterial microcompartments protein family protein		2.11	2.92E-01	1.11	1.56E-01	1.01	5.96E-01
MSMEG_0276	aldehyde-alcohol dehydrogenase		0.99	9.07E-01	0.94	6.30E-01	0.81	1.56E-01
MSMEG_0277	putative aminotransferase		2.34	4.87E-02	1.03	7.93E-01	1.02	7.84E-01
MSMEG_0278	hypothetical protein		0.94	6.47E-01	1.14	2.37E-01	0.89	2.57E-01
MSMEG_0279	amino acid permease		1.82	5.70E-02	1.73	1.68E-01	1.00	9.15E-01
MSMEG_0280	alpha/beta hydrolase fold		1.31	3.95E-01	1.39	8.18E-03	0.74	1.06E-01
MSMEG_0281	choline dehydrogenase		2.17	3.36E-02	0.93	6.02E-01	0.54	5.35E-02
MSMEG_0282	conserved hypothetical protein		1.49	1.24E-01	0.72	1.02E-01	0.43	3.40E-04
MSMEG_0283	3-oxoacyl-[acyl-carrier-protein] reductase		0.55	3.63E-03	1.02	8.59E-01	1.05	4.93E-01
MSMEG_0284	ribosyldihydronicotinamide dehydrogenase (quinone)		1.71	2.38E-03	0.80	9.66E-02	0.81	2.75E-01
MSMEG_0285	transcriptional regulator TetR family protein		1.47	1.86E-01	0.82	3.92E-02	0.99	7.27E-01
MSMEG_0286	GntR-family protein transcriptional regulator		1.37	2.98E-01	1.17	3.23E-01	1.63	1.41E-01
MSMEG_0287	conserved hypothetical protein		1.23	3.50E-01	0.96	9.18E-02	1.28	2.32E-01
MSMEG_0288	FAD dependent oxidoreductase, putative		1.21	3.69E-01	1.02	8.56E-01	1.02	6.42E-01
MSMEG_0289	alpha/beta hydrolase fold family protein		1.17	4.10E-01	0.83	1.47E-01	1.10	5.67E-01
MSMEG_0290	acyltransferase, ws/dgat/mgat subfamily protein		0.73	1.27E-03	1.29	2.68E-02	0.95	2.75E-01
MSMEG_0291	dioxygenase, TauD/TfdA family protein		1.10	5.78E-01	0.96	7.75E-01	1.01	8.82E-01
MSMEG_0292	conserved hypothetical protein		1.22	3.15E-01	0.98	9.01E-01	1.18	5.95E-01
MSMEG_0293	Rieske [2Fe-2S] domain protein		1.47	6.56E-03	1.05	5.16E-01	1.04	7.48E-01
MSMEG_0294	3-oxoacyl-(acyl-carrier-protein) reductase	*fabG*	1.90	8.76E-02	0.88	3.50E-01	1.62	1.70E-01
MSMEG_0295	oxidoreductase, FAD-binding		1.06	6.09E-01	1.01	7.48E-01	1.14	5.79E-01
MSMEG_0296	transcriptional regulator, MarR family protein		2.10	1.17E-02	0.86	3.04E-01	0.92	5.06E-01
MSMEG_0297	amidohydrolase		0.92	5.03E-01	1.03	8.54E-01	1.27	4.50E-01
MSMEG_0298	conserved hypothetical protein		1.50	1.04E-01	1.14	4.66E-01	1.20	3.24E-01
MSMEG_0299	Rieske [2Fe-2S] domain protein		1.46	6.93E-02	1.40	4.24E-02	1.31	3.49E-01
MSMEG_0300	amidohydrolase family protein		2.28	6.78E-02	0.93	4.59E-01	0.97	5.23E-01
MSMEG_0301	oxidoreductase, FAD-binding		2.22	5.67E-03	0.74	8.25E-03	1.73	1.06E-02
MSMEG_0302	peptidase, S9A/B/C families		0.84	1.71E-01	0.78	1.93E-02	1.44	3.96E-01
MSMEG_0303	alcohol dehydrogenase, zinc-containing		1.87	5.70E-02	1.30	3.33E-01	1.02	8.18E-01
MSMEG_0304	acyl-CoA synthase		0.86	2.50E-01	0.83	9.68E-02	1.28	2.61E-01
MSMEG_0305	acyltransferase domain protein		1.77	4.37E-02	0.92	4.31E-01	0.91	3.25E-01
MSMEG_0306	arylamine N-acetyltransferase		1.76	6.59E-02	1.32	2.78E-01	1.02	2.69E-01

MSMEG_0307	transcriptional regulator, AraC family protein		2.47	1.54E-01	1.24	3.16E-01	0.86	2.77E-01
MSMEG_0308	dihydrofolate reductase		1.11	5.07E-01	1.07	5.14E-01	0.55	8.00E-03
MSMEG_0309	aldehyde dehydrogenase family protein		2.26	2.23E-03	0.87	1.07E-01	0.36	5.28E-05
MSMEG_0310	SAM-dependent methyltransferase; this gene contains a frame shift which is not the result of sequencing error		0.89	6.69E-01	1.00	9.60E-01	1.20	2.23E-01
MSMEG_0311	glycosyltransferase		0.58	2.02E-03	1.01	8.81E-01	1.52	5.71E-02
MSMEG_0312	2-dehydro-3-deoxyphosphogluconate aldolase/4-hydroxy-2-oxoglutarate aldolase	*eda*	0.68	5.57E-04	1.16	2.39E-01	1.55	1.70E-01
MSMEG_0313	phosphogluconate dehydratase	*edd*	0.69	4.66E-02	1.08	5.56E-01	4.32	3.26E-01
MSMEG_0314	glucose-6-phosphate 1-dehydrogenase	*zwf*	0.43	1.50E-03	1.76	1.96E-02	1.28	5.64E-01
MSMEG_0315	probable conserved transmembrane protein		0.50	7.80E-03	0.98	7.98E-01	1.77	1.52E-01
MSMEG_0316	putative NagC regulator		0.98	7.50E-01	0.79	7.18E-02	1.23	1.55E-01
MSMEG_0317	conserved hypothetical protein		0.56	1.39E-04	0.95	4.84E-01	0.82	1.03E-01
MSMEG_0318	AMP-dependent synthetase and ligase		1.61	3.65E-02	1.01	8.51E-01	0.52	4.05E-04
MSMEG_0319	acyltransferase		0.94	7.33E-01	0.85	9.79E-02	0.96	5.96E-01
MSMEG_0320	putative phosphotriesterase		1.63	8.98E-03	1.19	3.33E-02	1.29	7.20E-01
MSMEG_0321	transcriptional regulator		1.29	3.49E-02	0.77	1.94E-02	1.15	4.09E-02
MSMEG_0322	glyoxylase family protein; this gene contains a frame shift which is not the result of sequencing error		1.26	1.99E-01	0.82	3.01E-01	1.01	3.15E-01
MSMEG_0323	acyl-CoA dehydrogenase, short-chain specific, putative		1.58	3.48E-03	1.96	4.05E-01	0.97	5.58E-01
MSMEG_0324	conserved hypothetical protein		0.95	6.87E-01	1.24	8.22E-02	1.60	4.02E-01
MSMEG_0325	conserved hypothetical protein		1.28	2.33E-01	0.94	5.30E-01	1.02	2.67E-01
MSMEG_0326	AMP-dependent synthetase and ligase		1.10	2.84E-01	1.08	4.93E-01	1.36	2.21E-01
MSMEG_0327	aldehyde dehydrogenase		1.04	8.22E-01	1.01	9.20E-01	0.91	2.64E-01
MSMEG_0328	hypothetical protein		2.08	2.35E-01	1.22	4.77E-01	1.24	3.40E-01
MSMEG_0329	FMN oxidoreductase		1.53	1.40E-01	0.73	3.05E-03	1.08	3.23E-01
MSMEG_0330	transcriptional regulator, LuxR family protein		6.43	6.68E-02	1.07	6.16E-01	0.07	5.35E-06
MSMEG_0331	transcriptional regulator, LuxR family protein		5.60	9.49E-03	1.15	4.65E-01	0.05	2.09E-07
MSMEG_0332	2-nitropropane dioxygenase, NPD		1.12	5.12E-01	0.99	8.75E-01	0.96	1.52E-01
MSMEG_0333	Carboxyl transferase domain protein		0.69	3.23E-02	0.98	7.60E-01	0.80	7.86E-02
MSMEG_0334	acetyl-/propionyl-coenzyme A carboxylase alpha chain		0.76	1.07E-01	0.98	7.91E-01	0.88	1.35E-01
MSMEG_0335	enoyl-CoA hydratase/isomerase		0.75	1.86E-01	1.00	9.56E-01	0.82	1.34E-01
MSMEG_0336	conserved hypothetical protein		1.00	9.83E-01	1.62	1.15E-01	0.80	1.80E-01
MSMEG_0337	conserved hypothetical protein		0.66	7.22E-04	1.16	2.50E-01	0.75	3.60E-02

MSMEG_0338	acyl-CoA dehydrogenase fadE12	0.73	1.43E-03	1.16	2.11E-01	0.94	3.22E-01
MSMEG_0339	FMN-dependent monooxygenase	0.87	5.51E-03	0.94	2.44E-01	0.99	7.30E-01
MSMEG_0340	enoyl-CoA hydratase/isomerase family protein	1.19	1.25E-01	0.94	3.58E-01	1.27	4.27E-01
MSMEG_0341	conserved hypothetical protein	2.27	9.26E-02	0.93	4.73E-01	1.18	5.85E-01
MSMEG_0342	hypothetical protein	1.42	2.87E-01	0.96	8.72E-01	0.98	7.71E-01
MSMEG_0343	TetR-family protein transcriptional regulator	0.99	9.03E-01	0.86	3.30E-01	1.54	2.38E-01
MSMEG_0344	conserved hypothetical protein	1.12	3.74E-01	1.10	1.47E-01	1.26	3.90E-01
MSMEG_0345	conserved hypothetical protein	0.98	8.55E-01	1.11	5.96E-01	1.06	6.09E-01
MSMEG_0346	virulence factor	0.90	6.18E-01	1.29	3.59E-01	1.54	1.60E-01
MSMEG_0347	virulence factor Mce family protein	1.97	1.81E-01	0.77	9.43E-02	1.01	8.73E-01
MSMEG_0348	mce-family protein mce3c	1.35	2.28E-01	0.86	2.46E-01	0.91	3.79E-01
MSMEG_0349	virulence factor mce family protein	1.44	3.46E-01	0.94	7.93E-01	1.20	1.82E-01
MSMEG_0350	virulence factor Mce family protein	1.44	2.33E-01	0.95	7.97E-01	1.01	8.94E-01
MSMEG_0351	virulence factor mce family protein	1.29	3.08E-01	0.94	7.75E-01	1.24	2.49E-01
MSMEG_0352	conserved hypothetical protein	1.23	4.92E-01	0.86	5.22E-01	1.13	3.42E-01
MSMEG_0353	conserved hypothetical protein	1.50	2.32E-01	0.75	1.06E-01	1.11	4.25E-01
MSMEG_0354	conserved hypothetical protein	1.33	1.19E-01	0.90	5.84E-01	1.16	4.60E-01
MSMEG_0355	conserved hypothetical protein	1.29	1.91E-01	0.75	1.40E-01	0.95	8.17E-01
MSMEG_0356	acetyltransferase	2.02	2.01E-03	0.78	6.09E-03	0.90	3.88E-01
MSMEG_0357	transmembrane transport protein	2.37	7.88E-02	0.93	3.84E-01	1.21	1.39E-01
MSMEG_0358	ribonucleoside-diphosphate reductase, beta subunit	4.77	3.27E-03	1.03	8.17E-01	0.11	2.24E-06
MSMEG_0359	conserved hypothetical protein	0.58	7.89E-02	0.81	5.31E-02	1.34	1.03E-01
MSMEG_0360	conserved hypothetical protein	0.91	3.52E-02	1.06	6.15E-01	1.23	2.06E-01
MSMEG_0361	Glycosyl hydrolase family protein 3	0.73	7.09E-04	0.80	5.30E-02	0.58	4.29E-02
MSMEG_0362	amidohydrolase 2	1.12	3.78E-01	1.02	5.98E-01	1.37	1.15E-01
MSMEG_0363	TetR-family protein regulatory protein	1.26	1.99E-02	0.84	1.15E-01	1.12	6.67E-01
MSMEG_0364	hypothetical protein	1.53	1.86E-02	1.07	2.80E-01	1.19	4.88E-01
MSMEG_0365	conserved hypothetical protein	1.22	9.45E-02	1.32	4.70E-02	1.57	3.27E-01
MSMEG_0366	hypothetical protein	1.39	2.66E-01	0.99	9.56E-01	0.45	1.31E-04
MSMEG_0367	O-demethylpuromycin-O-methyltransferase	0.66	3.62E-03	1.07	4.06E-01	0.95	3.73E-01
MSMEG_0368	hypothetical protein	0.66	5.40E-03	0.87	5.60E-01	1.55	1.40E-01
MSMEG_0369	hypothetical protein	0.68	7.41E-03	1.01	8.81E-01	2.00	6.21E-03
MSMEG_0370	conserved hypothetical protein	0.59	1.37E-03	1.00	9.97E-01	2.31	2.74E-02
MSMEG_0371	MaoC like domain protein	0.67	1.58E-02	1.03	8.00E-01	1.53	2.22E-01
MSMEG_0372	oxidoreductase, short chain dehydrogenase/reductase family protein	0.83	3.79E-01	0.90	3.97E-01	2.27	2.22E-01
MSMEG_0373	3-ketoacyl-CoA thiolase	1.29	9.50E-02	0.99	9.39E-01	1.43	2.44E-01

MSMEG_0374	glycolate oxidase subunit		1.11	4.51E-01	1.13	4.98E-01	1.02	8.32E-01
MSMEG_0375	phospholipase D family protein		1.13	1.52E-04	1.80	7.67E-02	1.60	3.91E-02
MSMEG_0376	transcriptional regulator, AraC family protein		0.83	2.56E-01	1.08	4.69E-01	1.23	3.57E-01
MSMEG_0377	nitrile hydratase		1.61	1.08E-01	1.27	2.34E-01	1.01	3.81E-01
MSMEG_0378	nitrile hydratase, alpha subunit	*nthA*	1.12	6.43E-02	1.11	5.50E-01	1.04	3.71E-01
MSMEG_0379	nitrile hydratase activator P14k		1.01	8.02E-01	0.92	4.28E-01	1.00	9.43E-01
MSMEG_0380	MmpS4 protein		0.61	1.57E-03	0.97	6.86E-01	0.48	3.54E-03
MSMEG_0381	Mmp14a protein		0.68	3.70E-02	1.01	8.72E-01	0.55	4.36E-02
MSMEG_0382	putative transport protein		0.60	6.85E-03	1.02	7.61E-01	0.70	7.72E-02
MSMEG_0383	conserved hypothetical protein		1.82	1.13E-02	0.77	7.08E-02	0.74	9.65E-02
MSMEG_0384	glucose-1-phosphate thymidylyltransferase	*rfbA*	0.98	5.87E-01	0.92	1.52E-01	0.71	2.71E-02
MSMEG_0385	hypothetical glycosyl transferase		1.07	4.20E-01	1.03	3.61E-01	0.65	3.98E-02
MSMEG_0386	NAD dependent epimerase/dehydratase family protein		0.57	4.35E-03	0.87	2.04E-01	0.68	7.73E-02
MSMEG_0387	Rmt2 protein		0.53	8.69E-05	0.87	2.23E-02	1.03	6.13E-01
MSMEG_0388	Macrocin-O-methyltransferase	*tylF*	0.68	4.87E-04	1.00	9.77E-01	0.39	2.82E-03
MSMEG_0389	hypothetical glycosyl transferase		0.41	7.46E-07	0.90	8.71E-02	0.67	1.94E-01
MSMEG_0390	putative acyltransferase		0.39	2.44E-04	1.09	2.56E-01	0.86	4.26E-01
MSMEG_0391	Rmt3 protein		0.54	5.54E-03	0.80	2.95E-01	1.11	6.22E-01
MSMEG_0392	hypothetical glycosyl transferase		0.82	4.56E-02	0.96	6.41E-01	0.88	9.63E-02
MSMEG_0393	Fmt protein		1.35	2.42E-04	0.88	3.81E-01	0.43	3.01E-04
MSMEG_0394	hypothetical protein		0.57	6.84E-04	0.81	6.26E-02	1.01	9.75E-01
MSMEG_0395	hypothetical protein		0.94	3.85E-01	1.12	1.84E-01	1.17	3.39E-01
MSMEG_0396	hypothetical protein		1.01	9.51E-01	0.71	3.38E-01	1.36	6.04E-02
MSMEG_0399	conserved domain protein		1.35	9.39E-03	1.20	3.72E-02	1.04	4.56E-01
MSMEG_0400	peptide synthetase		0.75	4.69E-04	1.09	1.91E-01	0.70	2.88E-03
MSMEG_0401	putative non-ribosomal peptide synthase		0.79	1.84E-01	1.02	7.75E-01	1.89	2.78E-01
MSMEG_0402	linear gramicidin synthetase subunit D		0.71	1.18E-03	1.18	1.00E-01	1.52	1.41E-02
MSMEG_0403	integral membrane protein		0.53	1.86E-04	0.99	8.80E-01	0.93	1.19E-01
MSMEG_0404	sigma associated protein		0.57	5.18E-04	0.91	4.33E-02	0.88	2.65E-01
MSMEG_0405	extra cytoplasmic sigma factor		0.93	5.99E-01	1.04	5.34E-01	2.27	2.39E-02
MSMEG_0406	acyl-coA-dehydrogenase		1.55	2.31E-02	2.21	7.73E-02	3.70	1.42E-01
MSMEG_0407	conserved hypothetical protein		1.88	6.75E-02	0.99	8.38E-01	0.49	2.68E-02
MSMEG_0408	type I modular polyketide synthase		0.77	6.45E-02	0.97	6.79E-01	1.01	9.70E-01
MSMEG_0409	Condensation domain protein		0.38	6.52E-06	1.13	2.62E-02	1.53	1.48E-01
MSMEG_0410	MmpL protein		0.50	8.43E-05	1.03	4.62E-01	1.87	1.01E-01
MSMEG_0411	acyl-CoA synthase		0.49	3.08E-05	0.92	3.04E-01	1.77	1.21E-01
MSMEG_0412	conserved hypothetical protein		0.95	4.09E-01	0.88	2.62E-03	0.84	3.81E-01

MSMEG_0413	conserved hypothetical protein		1.00	9.65E-01	1.02	5.86E-01	9.97	3.55E-01
MSMEG_0414	oxidoreductase, 2OG-Fe(II) oxygenase family protein		1.60	4.70E-03	0.92	5.43E-01	1.17	1.33E-01
MSMEG_0415	NADH-fmn oxidoreductase		1.55	1.18E-01	0.83	3.28E-01	0.78	3.41E-02
MSMEG_0416	hypothetical protein		5.93	1.87E-04	0.88	3.16E-01	0.86	4.42E-01
MSMEG_0417	succinate dehydrogenase		3.96	2.78E-02	0.93	5.35E-01	0.14	1.10E-05
MSMEG_0418	succinate dehydrogenase flavoprotein subunit		4.37	8.16E-02	0.74	4.39E-02	0.09	5.66E-06
MSMEG_0419	integral membrane protein		4.41	5.29E-02	1.07	5.24E-01	0.03	2.62E-09
MSMEG_0420	conserved hypothetical protein		9.91	4.10E-02	1.31	6.10E-02	0.02	8.25E-10
MSMEG_0421	hypothetical protein		0.91	9.02E-02	1.10	5.11E-01	8.79	3.88E-01
MSMEG_0422	transferase		1.02	7.38E-01	0.88	1.78E-01	1.04	8.09E-01
MSMEG_0423	conserved hypothetical protein		1.23	6.50E-02	1.14	1.76E-01	0.73	1.24E-01
MSMEG_0424	Hsp20/alpha crystallin family protein		5.15	8.19E-03	1.98	2.43E-02	1.53	2.30E-01
MSMEG_0425	putative membrane protein DcsA		1.06	2.69E-01	1.22	1.47E-01	0.95	3.99E-01
MSMEG_0426	transcriptional regulator, GntR family protein		1.86	3.87E-01	0.96	7.03E-01	1.03	5.71E-01
MSMEG_0427	nitrite reductase [NAD(P)H], large subunit	*nirB*	0.94	7.74E-01	1.21	3.46E-01	1.28	4.54E-01
MSMEG_0428	nitrite reductase [NAD(P)H] small subunit		0.82	6.03E-01	1.39	4.08E-01	0.95	5.08E-01
MSMEG_0429	putative ferric uptake regulator		0.64	3.05E-03	1.55	2.36E-01	1.47	3.64E-01
MSMEG_0431	secreted protein		0.33	1.68E-02	0.94	6.01E-01	0.90	1.89E-01
MSMEG_0432	uroporphyrinogen-III synthetase		0.37	7.67E-03	1.14	7.31E-02	0.99	5.61E-01
MSMEG_0433	nitrite extrusion protein		1.23	4.10E-01	1.37	2.97E-02	1.14	3.24E-01
MSMEG_0434	aminoglycoside 2'-N-acetyltransferase (AAC(2')-Id)		2.57	9.60E-04	0.65	4.53E-03	1.61	3.06E-02
MSMEG_0435	allophanate hydrolase subunit 2		2.58	2.20E-02	1.07	4.95E-01	0.81	3.12E-01
MSMEG_0436	allophanate hydrolase subunit 1		2.47	1.02E-02	1.51	1.30E-03	0.70	3.85E-02
MSMEG_0437	conserved hypothetical integral membrane protein		0.56	1.37E-03	0.85	3.22E-02	1.00	9.79E-01
MSMEG_0438	Periplasmic binding protein		0.80	1.40E-01	0.98	8.29E-01	1.59	6.39E-02
MSMEG_0439	conserved hypothetical protein		0.60	3.01E-02	2.88	1.57E-01	1.27	3.23E-01
MSMEG_0440	hypothetical protein		0.80	1.34E-02	1.34	3.73E-01	0.76	2.55E-01
MSMEG_0441	conserved hypothetical protein, putative		1.06	2.89E-01	0.85	5.58E-02	0.52	3.74E-03
MSMEG_0442	tetracyclin repressor, C- all-alpha domain protein		1.94	3.95E-02	0.86	5.04E-02	1.49	2.21E-01
MSMEG_0443	hydrolase, carbon-nitrogen family protein		1.13	6.13E-01	1.09	3.51E-01	1.32	4.81E-01
MSMEG_0444	agmatine deiminase		0.70	2.92E-02	1.02	8.70E-01	1.36	5.12E-01
MSMEG_0445	amidohydrolase family protein		0.77	1.35E-01	0.96	3.59E-01	0.90	4.02E-01
MSMEG_0446	putrescine importer		0.81	8.33E-02	0.83	4.63E-02	1.10	4.52E-01
MSMEG_0447	conserved hypothetical protein		1.26	2.53E-01	2.80	1.40E-01	1.24	5.25E-01
MSMEG_0448	transcriptional regulator, MarR family protein		1.53	3.48E-02	0.98	8.12E-01	0.74	1.46E-01

MSMEG_0449	transporter, major facilitator family protein, putative		0.75	1.58E-01	2.11	1.49E-01	0.90	2.03E-01
MSMEG_0450	hypothetical protein		1.24	2.85E-01	1.63	1.52E-01	1.31	4.70E-01
MSMEG_0451	oxidoreductase, FAD-linked		2.08	1.20E-01	2.11	4.33E-02	0.21	1.62E-04
MSMEG_0452	inner membrane permease YgbN		2.92	1.04E-01	0.85	6.26E-02	0.21	9.71E-05
MSMEG_0453	shikimate kinase	*aroK*	1.74	1.70E-02	0.99	8.72E-01	1.27	6.28E-01
MSMEG_0454	GntR-family protein transcriptional regulator		0.99	8.67E-01	0.97	8.49E-01	3.23	3.46E-01
MSMEG_0455	aldehyde dehydrogenase		1.40	1.15E-02	0.93	1.68E-01	0.36	3.38E-05
MSMEG_0456	DNA gyrase subunit A		0.35	3.06E-04	0.81	1.44E-01	1.79	1.28E-01
MSMEG_0457	DNA topoisomerase IV subunit B		0.40	1.43E-04	0.91	6.60E-01	4.43	1.27E-01
MSMEG_0458	two-component system sensor kinase		1.18	1.27E-01	0.96	6.62E-01	1.40	2.99E-01
MSMEG_0459	two-component system response regulator		1.14	2.58E-01	0.83	1.11E-01	0.91	1.36E-01
MSMEG_0460	alpha/beta hydrolase fold		0.91	3.46E-01	0.89	1.52E-01	1.09	4.95E-01
MSMEG_0461	CinZ protein		1.69	1.01E-04	1.12	8.49E-02	0.87	5.01E-01
MSMEG_0462	MmpS4 protein		0.64	3.44E-02	1.32	1.08E-01	1.14	2.43E-01
MSMEG_0463	MmpL4 protein		0.76	1.69E-02	1.43	3.88E-02	1.04	1.99E-01
MSMEG_0464	phosphomethylpyrimidine kinase		1.03	1.91E-01	0.96	4.94E-01	0.85	5.98E-02
MSMEG_0465	transcriptional regulator, TetR family protein		1.94	3.26E-02	0.74	3.51E-02	1.41	1.94E-01
MSMEG_0466	hypothetical protein		0.77	1.23E-01	1.05	3.26E-01	1.42	7.66E-02
MSMEG_0467	membrane transport protein		5.31	3.22E-01	3.80	4.57E-02	13.23	1.95E-01
MSMEG_0468	integral membrane protein		0.55	2.17E-02	0.99	9.57E-01	1.89	3.69E-01
MSMEG_0469	transcriptional regulatory protein PadR-		1.55	7.96E-03	1.00	9.89E-01	1.14	2.72E-01
MSMEG_0470	para-nitrobenzyl esterase		7.62	1.68E-03	0.66	1.07E-01	1.45	2.83E-01
MSMEG_0471	transcriptional regulator LysR family protein		1.06	4.34E-01	1.02	6.64E-01	1.05	2.82E-01
MSMEG_0472	putative lipoprotein		10.73	1.04E-03	0.49	1.54E-02	0.64	6.73E-03
MSMEG_0473	transcriptional regulator, LuxR family protein		0.66	3.38E-03	0.91	2.66E-01	0.95	7.83E-01
MSMEG_0474	glucosamine--fructose-6-phosphate aminotransferase (isomerizing)		1.30	1.80E-01	1.08	2.69E-01	1.33	1.61E-01
MSMEG_0475	nucleotide-sugar dehydrogenase		1.44	2.14E-01	1.25	9.30E-02	0.96	4.52E-01
MSMEG_0476	chitin synthase		1.17	4.98E-01	1.02	8.57E-01	1.11	2.98E-01
MSMEG_0477	hypothetical protein		0.89	4.71E-01	1.26	1.82E-01	0.84	1.35E-01
MSMEG_0478	secreted protein, putative		1.26	1.49E-01	0.94	5.65E-01	1.10	3.71E-01
MSMEG_0479	hypothetical protein		1.17	1.75E-01	1.09	5.90E-01	1.63	3.69E-01
MSMEG_0480	transcriptional regulator, GntR family protein		0.67	1.99E-03	1.01	9.05E-01	1.21	5.40E-01
MSMEG_0481	FAD dependent oxidoreductase		1.35	3.80E-01	0.94	5.77E-01	0.87	1.74E-01
MSMEG_0482	dihydroxy-acid dehydratase		1.11	5.00E-01	0.96	6.56E-01	1.01	6.23E-01
MSMEG_0483	shikimate transporter		1.09	3.98E-01	1.06	6.70E-01	1.02	8.06E-01
MSMEG_0484	formamidase		0.90	5.30E-01	0.88	3.79E-01	0.87	4.66E-01

MSMEG_0485	amidase	1.36	3.07E-01	1.21	4.89E-01	1.08	1.77E-01
MSMEG_0486	putative ABC transporter, periplasmic protein	1.18	1.59E-01	0.74	1.05E-01	0.99	6.56E-01
MSMEG_0487	ABC transporter permease	1.93	2.31E-01	0.99	9.36E-01	0.98	5.76E-01
MSMEG_0488	ABC transporter ATP-binding protein	1.18	2.70E-01	1.19	1.98E-01	1.05	7.01E-01
MSMEG_0489	racemase	1.14	4.85E-01	1.28	1.34E-01	0.95	4.06E-01
MSMEG_0490	enoyl-CoA hydratase	2.03	1.56E-01	0.95	4.42E-01	1.24	1.25E-01
MSMEG_0491	transcriptional regulator, LacI family protein	1.54	2.14E-01	0.97	9.41E-01	1.68	2.08E-01
MSMEG_0492	conserved hypothetical protein	1.02	8.10E-01	0.92	1.07E-01	1.17	3.84E-01
MSMEG_0493	hypothetical protein	5.48	2.56E-03	1.91	9.74E-02	1.39	4.03E-01
MSMEG_0494	hypothetical protein	2.55	1.36E-02	1.37	1.92E-02	1.07	5.75E-01
MSMEG_0495	glycerol dehydratase reactivase chain A, putative; this gene contains a frame shift which is not the result of sequencing error	1.30	8.25E-02	1.11	1.11E-01	1.25	2.34E-01
MSMEG_0496	coenzyme B12-dependent glycerol dehydrogenase small subunit	1.04	8.47E-01	1.16	3.11E-01	1.08	7.56E-01
MSMEG_0497	glycerol dehydratase large subunit	1.07	5.89E-01	0.95	1.02E-01	0.97	6.57E-01
MSMEG_0498	hypothetical protein	1.19	1.32E-01	1.17	2.87E-01	1.54	3.64E-01
MSMEG_0499	amino acid permease-associated region	1.30	3.44E-01	0.97	7.51E-01	1.10	1.96E-01
MSMEG_0500	regulator of polyketide synthase expression, putative	0.71	7.19E-02	0.99	8.02E-01	2.67	1.10E-01
MSMEG_0501	glucosamine-6-phosphate deaminase 1	0.55	7.78E-03	0.90	1.62E-01	0.97	6.95E-01
MSMEG_0502	glucosidase	1.13	5.70E-01	0.72	1.02E-01	0.87	3.73E-01
MSMEG_0503	DeoR-family protein transcriptional regulator	0.98	8.57E-01	0.90	2.29E-01	0.99	8.34E-01
MSMEG_0504	carbohydrate kinase	1.24	3.37E-01	1.07	5.93E-01	0.83	1.23E-01
MSMEG_0505	probable sugar ABC transporter, substrate-binding protein, putative	1.10	5.50E-01	0.76	1.87E-02	0.49	9.37E-03
MSMEG_0506	ABC transporter, permease protein	1.01	9.66E-01	1.01	9.63E-01	0.96	2.89E-02
MSMEG_0507	probable sugar ABC transporter, permease protein	0.99	8.66E-01	0.76	5.19E-02	0.98	5.83E-01
MSMEG_0508	glycerol-phosphate porter	1.85	1.81E-01	0.97	8.17E-01	0.81	1.26E-01
MSMEG_0509	transcriptional regulatory protein	0.95	6.50E-01	1.13	3.54E-01	0.78	7.70E-02
MSMEG_0510	D-tagatose-bisphosphate aldolase, class II, non-catalytic subunit	2.56	1.42E-01	1.17	2.75E-01	1.24	2.59E-01
MSMEG_0511	putative sugar isomerase, AgaS family protein	1.47	2.01E-01	1.20	6.19E-02	1.02	3.47E-01
MSMEG_0512	BadF/BadG/BcrA/BcrD ATPase family protein	1.47	1.59E-01	1.04	6.05E-01	1.02	4.50E-01
MSMEG_0513	integral membrane protein	1.54	5.00E-02	0.98	8.51E-01	0.95	4.11E-01
MSMEG_0514	alpha-galactosidase	1.13	3.46E-01	1.10	4.58E-01	2.06	3.35E-01
MSMEG_0515	probable sugar transporter sugar binding lipoprotein	1.27	3.97E-01	0.99	8.90E-01	0.91	3.67E-01

MSMEG_0516	sugar transport system	1.09	5.43E-01	0.98	8.79E-01	0.93	5.65E-01
MSMEG_0517	sugar binding-protein dependent transporter system permease	1.35	2.64E-01	1.56	1.56E-01	0.95	5.81E-01
MSMEG_0518	ABC transporter, nucleotide binding/ATPase protein, sn-Glycerol-3-phosphate	1.77	9.34E-02	0.87	1.72E-01	2.05	3.63E-01
MSMEG_0519	conserved hypothetical protein	1.69	2.62E-04	1.24	2.75E-01	1.18	2.96E-01
MSMEG_0520	porin	1.78	6.32E-03	0.99	9.54E-01	0.44	4.93E-02
MSMEG_0521	conserved hypothetical protein; this gene contains a frame shift which is not the result of sequencing error	1.25	3.33E-02	1.28	2.27E-01	1.48	1.17E-01
MSMEG_0521	conserved hypothetical protein; this gene contains a frame shift which is not the result of sequencing error	1.04	7.55E-01	1.13	4.27E-01	1.00	9.91E-01
MSMEG_0522	pp24 protein	1.33	2.78E-01	1.25	3.10E-01	1.01	5.74E-01
MSMEG_0523	DNA-binding protein	1.31	1.22E-01	0.94	7.80E-01	0.92	2.71E-01
MSMEG_0524	short chain dehydrogenase	1.39	1.11E-01	1.02	6.16E-01	1.24	3.94E-01
MSMEG_0525	hypothetical protein	1.16	2.45E-01	1.28	6.15E-02	0.88	3.05E-01
MSMEG_0526	oxidoreductase, GMC family protein	1.20	3.64E-01	1.08	3.60E-01	1.38	7.67E-02
MSMEG_0527	2-hydroxycyclohexnecarboxyl-CoA dehydrogenase	1.30	5.11E-03	1.22	6.32E-02	0.91	6.32E-01
MSMEG_0528	conserved hypothetical protein	1.96	6.50E-02	1.20	4.02E-01	1.09	5.67E-01
MSMEG_0529	probable serine/threonine-protein kinase PknK	4.34	7.81E-03	0.62	1.83E-02	2.96	3.06E-01
MSMEG_0530	short chain dehydrogenase	2.66	3.58E-03	0.52	1.57E-02	2.42	2.13E-01
MSMEG_0531	putative acyl-CoA dehydrogenase	0.36	2.54E-05	0.85	1.61E-01	1.41	1.76E-01
MSMEG_0532	transcriptional regulator, TetR family protein	0.47	1.17E-04	1.16	3.62E-02	1.52	2.71E-01
MSMEG_0533	gp236 protein	8.36	2.54E-02	0.44	1.21E-02	0.73	2.13E-01
MSMEG_0534	permease, major facilitator family protein	1.23	1.72E-01	1.15	6.83E-01	1.47	2.79E-01
MSMEG_0535	GntR-family protein transcriptional regulator	0.56	1.64E-03	1.29	1.97E-01	1.55	1.92E-01
MSMEG_0536	intracellular protease, PfpI family protein	3.81	3.56E-02	1.62	1.98E-01	0.43	8.49E-04
MSMEG_0537	conserved hypothetical protein	3.62	2.02E-03	0.74	1.16E-01	0.72	1.85E-01
MSMEG_0537	conserved hypothetical protein	2.62	7.13E-03	0.87	4.07E-01	0.86	5.35E-01
MSMEG_0538	regulatory protein, MarR	2.29	1.04E-03	0.97	6.22E-01	1.11	7.11E-01
MSMEG_0539	transcriptional regulator, Crp/Fnr family protein	0.93	5.59E-01	0.70	8.75E-03	0.30	1.78E-04
MSMEG_0540	transcriptional regulator, putative	1.26	3.40E-02	0.73	1.50E-02	1.38	2.36E-01
MSMEG_0541	hypothetical protein	1.71	4.23E-03	0.98	5.21E-01	0.34	1.99E-06
MSMEG_0542	antar domain protein	1.22	1.07E-01	1.02	7.88E-01	0.55	4.69E-03
MSMEG_0543	conserved hypothetical protein	1.65	4.04E-03	1.10	6.84E-01	0.69	1.29E-01
MSMEG_0544	transcriptional regulator	1.50	1.25E-02	0.81	8.03E-02	1.21	1.30E-01

MSMEG_0545	transcriptional regulator, LuxR family protein		1.68	8.89E-02	0.87	9.75E-02	0.90	2.47E-01
MSMEG_0546	conserved hypothetical protein		0.56	1.33E-02	1.36	2.51E-01	0.87	3.51E-01
MSMEG_0547	ISMsm5, transposase		1.09	3.19E-01	0.88	1.43E-01	0.54	2.64E-05
MSMEG_0548	chromosome replication initiation inhibitor protein		0.96	7.89E-01	0.98	6.19E-01	4.40	3.10E-02
MSMEG_0549	ABC transporter, permease protein		0.69	3.04E-03	1.07	8.26E-01	3.99	1.65E-01
MSMEG_0550	sulfonate binding protein		0.69	1.55E-02	1.29	3.72E-01	1.73	2.89E-01
MSMEG_0551	ABC nitrate/sulfonate/bicarbonate transporter, ATPase subunit		0.73	6.29E-02	1.47	2.07E-01	3.27	1.88E-01
MSMEG_0552	conserved hypothetical protein		0.39	3.72E-05	1.10	3.14E-01	0.94	6.06E-01
MSMEG_0553	probable sugar ABC transporter, substrate-binding protein, putative		0.80	5.55E-02	4.56	1.18E-01	1.45	1.77E-01
MSMEG_0554	ABC transporter permease protein, putative		0.95	7.19E-01	1.24	2.21E-01	1.05	3.88E-01
MSMEG_0555	ABC transporter permease protein		1.14	1.16E-01	1.48	5.04E-02	1.00	9.68E-01
MSMEG_0556	ABC transporter, nucleotide binding/ATPase protein		1.22	2.29E-01	1.17	2.22E-01	1.01	9.47E-01
MSMEG_0557	hypothetical protein		2.37	2.11E-04	0.71	7.90E-02	1.03	8.46E-01
MSMEG_0558	conserved hypothetical protein		2.50	4.85E-03	0.92	7.20E-01	1.46	2.37E-01
MSMEG_0559	conserved domain protein		1.32	2.81E-02	0.74	3.69E-01	1.35	3.17E-01
MSMEG_0560	dihydrodipicolinate reductase, N-terminus domain protein	*dapB*	1.57	8.91E-02	1.13	3.35E-01	0.78	2.01E-01
MSMEG_0561	putative TetR family protein receptor protein		0.67	4.93E-03	0.84	1.70E-01	1.47	4.38E-01
MSMEG_0562	conserved hypothetical protein		0.74	5.21E-02	0.91	2.62E-01	1.08	1.38E-01
MSMEG_0563	hypothetical protein		0.57	1.01E-03	1.31	1.63E-01	1.00	9.76E-01
MSMEG_0564	xanthine/uracil permease		0.42	1.12E-04	0.94	6.65E-01	1.36	1.28E-01
MSMEG_0565	putative glycosyl transferases group 1		0.91	6.43E-01	1.14	2.93E-01	1.52	1.42E-01
MSMEG_0566	aliphatic amidase		0.65	1.06E-02	1.29	2.60E-02	1.06	4.02E-01
MSMEG_0567	selenophosphate synthetase		0.21	5.06E-04	1.46	4.81E-01	0.87	5.72E-01
MSMEG_0568	radical SAM domain protein		0.24	1.34E-04	1.59	2.76E-01	1.73	4.45E-01
MSMEG_0569	flavoprotein involved in K+ transport		0.09	1.18E-05	4.04	3.49E-01	0.65	8.11E-02
MSMEG_0570	conserved hypothetical protein		0.12	1.02E-05	1.31	1.16E-01	0.87	1.41E-01
MSMEG_0571	hydrolase, carbon-nitrogen family protein		0.11	1.16E-05	1.78	2.54E-01	0.64	1.38E-01
MSMEG_0572	conserved hypothetical protein		0.09	7.34E-06	3.02	2.75E-01	0.55	7.60E-02
MSMEG_0573	putative ECF sigma factor RpoE1		1.18	1.13E-01	1.00	9.78E-01	2.01	2.83E-04
MSMEG_0574	putative ECF sigma factor RpoE1		2.25	3.47E-02	0.92	5.64E-01	1.04	4.58E-01
MSMEG_0575	MmpS1 protein		1.57	1.90E-02	1.03	7.70E-01	0.90	3.10E-01
MSMEG_0576	MmpL4 protein		1.07	6.53E-01	0.97	6.79E-01	1.27	4.48E-01
MSMEG_0577	major facilitator superfamily protein, putative		1.06	7.00E-01	1.43	2.00E-02	1.28	3.71E-01

MSMEG_0578	acyl-CoA dehydrogenase, middle domain protein		1.26	2.21E-01	1.69	4.89E-02	1.07	4.16E-01
MSMEG_0579	helix-turn-helix, Fis-type		0.72	1.07E-01	1.52	2.16E-01	1.10	3.09E-01
MSMEG_0580	conserved hypothetical protein		0.81	1.20E-01	0.86	1.21E-01	0.98	7.22E-01
MSMEG_0581	4-aminobutyrate transaminase	*gabT*	0.94	5.62E-01	1.04	8.39E-01	1.12	5.10E-01
MSMEG_0582	succinic semialdehyde dehydrogenase		0.87	7.01E-02	0.96	9.29E-02	1.06	5.44E-01
MSMEG_0583	probable membrane protein		6.94	1.30E-01	0.85	2.15E-02	0.85	1.81E-01
MSMEG_0584	hypothetical protein		1.61	9.10E-02	1.09	7.03E-01	0.46	5.53E-03
MSMEG_0585	L-carnitine dehydratase/bile acid-inducible protein F		1.64	1.46E-02	0.92	3.20E-01	0.98	1.46E-01
MSMEG_0586	stas domain, putative		1.26	2.70E-01	1.05	8.10E-01	0.46	1.90E-03
MSMEG_0587	L-rhamnose 1-epimerase	*rhaU*	1.31	1.67E-02	1.39	1.16E-01	1.70	4.74E-02
MSMEG_0588	putative rhamnose tranport protein, MFS family protein		1.24	2.97E-01	1.02	8.37E-01	0.95	6.93E-01
MSMEG_0589	L-rhamnose isomerase	*rhaI*	1.42	1.61E-01	1.06	4.05E-01	0.91	3.38E-01
MSMEG_0590	rhamnulose-1-phosphate aldolase/alcohol dehydrogenase	*rhaD*	1.59	1.26E-02	0.83	2.25E-01	1.07	7.52E-01
MSMEG_0591	rhamnulokinase	*rhaB*	0.97	6.37E-01	1.04	7.94E-01	1.12	3.89E-01
MSMEG_0592	putative rhamnose catabolism operon transcriptional regulator		0.77	9.34E-02	0.84	5.74E-02	1.84	3.69E-01
MSMEG_0593	conserved hypothetical protein		2.13	4.86E-03	0.87	3.30E-01	1.37	3.15E-01
MSMEG_0594	iron-sulfur cluster binding protein		1.13	7.90E-03	0.87	6.65E-03	1.59	1.70E-02
MSMEG_0595	glycolate oxidase		2.11	2.86E-03	0.84	2.72E-02	0.95	3.98E-01
MSMEG_0596	bacterial regulatory protein, GntR family protein		1.04	7.97E-01	1.13	6.48E-01	1.06	3.68E-01
MSMEG_0597	conserved hypothetical protein		2.08	2.28E-06	1.48	1.52E-01	0.88	1.68E-01
MSMEG_0598	hypothetical protein		1.17	4.52E-01	0.85	2.93E-01	1.00	5.85E-01
MSMEG_0599	acyl-CoA synthase		1.15	1.76E-01	0.90	3.06E-01	0.18	6.45E-06
MSMEG_0600	dehydrogenase		0.75	1.86E-01	1.27	6.37E-02	0.87	1.82E-01
MSMEG_0601	conserved hypothetical protein		2.26	6.27E-03	2.09	1.49E-01	1.27	2.12E-01
MSMEG_0602	hypothetical protein		4.82	1.24E-03	1.64	1.19E-01	0.74	1.30E-01
MSMEG_0603	putative acyl-CoA dehydrogenase		4.14	1.27E-02	0.76	5.34E-02	0.16	5.67E-06
MSMEG_0604	glyoxylate reductase		1.71	7.24E-02	0.80	5.93E-02	1.12	4.48E-01
MSMEG_0605	conserved hypothetical protein		2.30	6.83E-02	0.68	5.44E-02	3.95	3.76E-01
MSMEG_0606	transcriptional regulator, TetR family protein		1.00	9.73E-01	1.01	9.02E-01	1.06	5.41E-01
MSMEG_0607	methyltransferase, putative, family protein		0.93	5.10E-01	0.93	1.04E-01	0.95	5.39E-01
MSMEG_0608	glyoxalase family protein		1.29	6.55E-03	1.08	1.10E-01	0.63	6.34E-03
MSMEG_0609	helix-turn-helix domain protein		1.33	9.64E-02	0.91	1.57E-01	1.01	9.04E-01
MSMEG_0610	beta-lactamase		1.30	2.01E-01	1.14	5.57E-02	1.12	6.20E-01
MSMEG_0611	para-nitrobenzyl esterase		1.25	2.52E-01	1.22	6.43E-02	1.05	1.11E-01

MSMEG_0612	transcriptional regulator, TetR family protein		1.38	1.12E-01	1.00	9.97E-01	0.95	2.57E-01
MSMEG_0613	conserved hypothetical protein		1.82	8.96E-04	0.74	1.09E-02	0.61	3.24E-02
MSMEG_0614	methyltransferase		1.03	9.49E-01	0.78	1.96E-02	2.24	1.79E-02
MSMEG_0615	ATPase, AAA family protein		0.33	3.71E-05	1.27	1.37E-01	2.48	1.84E-01
MSMEG_0616	conserved hypothetical protein		0.37	2.52E-03	1.43	7.32E-02	2.22	2.99E-01
MSMEG_0617	ftsk/spoiiie family protein		0.30	4.94E-04	0.96	3.24E-01	3.26	2.19E-01
MSMEG_0618	pe family protein		0.38	1.41E-03	1.11	4.76E-01	1.92	2.08E-01
MSMEG_0619	ppe family protein		0.44	4.96E-03	0.95	6.92E-01	2.08	1.73E-01
MSMEG_0620	pe family protein		0.46	9.35E-04	1.08	3.60E-01	0.91	6.76E-01
MSMEG_0621	low molecular weight protein antigen 7		0.50	3.72E-03	0.98	9.12E-01	1.20	4.92E-01
MSMEG_0622	putative DNA-binding protein		0.35	6.65E-04	1.13	4.16E-01	1.54	2.73E-01
MSMEG_0623	secretion protein Snm4	*snm*	0.33	2.19E-04	1.06	7.25E-01	2.40	1.18E-01
MSMEG_0624	subtilase family protein		0.32	1.13E-04	1.07	6.30E-01	2.47	1.85E-01
MSMEG_0625	conserved hypothetical protein		0.94	6.87E-01	0.73	3.47E-02	1.09	5.34E-01
MSMEG_0626	conserved hypothetical protein		0.28	1.20E-04	1.28	2.61E-01	3.28	1.44E-01
MSMEG_0627	glycosyl transferase, family protein 39		0.87	5.42E-01	0.74	1.74E-01	0.87	3.21E-01
MSMEG_0628	amidohydrolase family protein		0.34	6.23E-04	1.05	7.42E-01	1.24	4.62E-01
MSMEG_0629	trans-aconitate 2-methyltransferase		0.91	3.69E-01	1.00	9.93E-01	1.02	3.59E-01
MSMEG_0630	conserved hypothetical protein		0.93	1.05E-01	1.02	8.84E-01	1.02	7.38E-01
MSMEG_0631	sulfatase family protein, authentic frameshift; this gene contains a frame shift which is not the result of sequencing error; identified by match to protein family HMM PF00884		1.15	1.95E-01	1.41	4.45E-01	3.38	2.95E-01
MSMEG_0632	conserved hypothetical protein		1.03	7.36E-01	0.75	7.44E-05	1.91	2.39E-03
MSMEG_0633	PAP2 superfamily protein		0.89	8.66E-02	1.01	8.76E-01	1.15	1.32E-01
MSMEG_0634	PAP2 superfamily protein		0.75	1.90E-02	1.07	3.17E-01	1.14	4.21E-01
MSMEG_0635	putative conserved exported protein		1.11	7.24E-01	0.88	4.68E-01	0.97	9.36E-01
MSMEG_0636	conserved hypothetical protein		0.65	9.68E-03	1.26	9.10E-02	0.65	1.16E-02
MSMEG_0637	iron-sulfur binding oxidoreductase		1.45	4.50E-01	1.17	9.17E-03	0.89	2.56E-01
MSMEG_0638	conserved hypothetical proline and threonine rich protein		0.84	3.68E-01	0.85	3.26E-01	1.70	2.05E-01
MSMEG_0639	oligopeptide transport ATP-binding protein AppF		0.47	1.79E-04	1.08	5.25E-01	0.60	1.15E-02
MSMEG_0640	oligopeptide transport ATP-binding protein OppD		0.99	9.07E-01	1.13	2.34E-01	0.48	2.19E-04
MSMEG_0641	binding-protein-dependent transport systems inner membrane component		0.68	5.85E-02	1.06	7.19E-01	0.32	4.13E-05
MSMEG_0642	hypothetical ABC transporter permease protein YliD		1.05	6.07E-01	1.65	9.39E-02	0.32	3.20E-05

MSMEG_0643	extracellular solute-binding protein, family protein 5, putative		0.85	2.80E-01	1.01	9.22E-01	0.30	4.44E-05
MSMEG_0644	cupin domain protein		0.26	8.41E-06	1.22	1.45E-01	0.97	5.69E-01
MSMEG_0645	putative beta-1,3-glucanase		0.42	8.54E-06	0.88	1.75E-01	0.52	2.58E-02
MSMEG_0646	putative transporter		1.10	2.72E-01	1.09	6.53E-01	0.85	7.78E-02
MSMEG_0647	phosphonate ABC transporter, ATP-binding protein	*phnC*	1.07	5.67E-01	0.87	3.59E-01	1.07	2.80E-01
MSMEG_0648	hypothetical protein		1.23	3.45E-02	1.14	1.70E-01	1.30	1.47E-01
MSMEG_0649	phosphonate-binding periplasmic protein		1.61	1.02E-01	0.93	7.48E-01	1.06	1.92E-01
MSMEG_0650	GntR-family protein transcriptional regulator		1.32	2.49E-01	1.30	1.11E-02	0.93	2.42E-01
MSMEG_0651	putative conserved exported protein		2.04	7.53E-02	1.28	5.13E-01	0.70	7.20E-02
MSMEG_0652	hypothetical protein		1.09	5.59E-01	1.01	8.45E-01	1.72	5.73E-02
MSMEG_0653	hypothetical protein; this gene contains a frame shift which is not the result of sequencing error; identified by Glimmer2; putative		1.01	9.31E-01	1.19	2.05E-01	0.95	4.19E-01
MSMEG_0654	conserved hypothetical protein		2.24	6.63E-02	0.82	6.15E-02	0.99	7.91E-01
MSMEG_0655	glucose 1-dehydrogenase, putative		1.14	3.08E-01	0.96	6.94E-01	1.09	4.83E-01
MSMEG_0656	transcriptional regulator, TetR family protein		1.67	1.35E-03	0.76	3.02E-02	1.05	7.64E-01
MSMEG_0657	Rieske 2Fe-2S domain protein		4.14	1.17E-02	0.69	3.24E-02	0.96	8.13E-01
MSMEG_0658	polyamine ABC-transporter, inner membrane subunit		1.22	2.74E-01	0.95	7.29E-01	0.92	1.84E-01
MSMEG_0659	polyamine ABC transporter permease protein		2.15	1.30E-02	0.91	2.44E-01	0.96	3.43E-01
MSMEG_0660	Bacterial extracellular solute-binding protein		7.82	1.89E-04	0.88	1.92E-01	0.30	5.83E-04
MSMEG_0661	glutamate-1-semialdehyde 2,1-aminomutase		4.99	2.13E-04	0.76	4.65E-02	0.83	8.16E-02
MSMEG_0662	putrescine transport ATP-binding protein PotG		2.72	1.50E-04	1.16	5.61E-02	0.91	1.45E-01
MSMEG_0663	TetR-family protein transcriptional regulator		1.37	5.10E-03	0.89	7.93E-02	1.51	1.29E-04
MSMEG_0664	FAD dependent oxidoreductase		4.31	9.16E-04	0.86	2.05E-02	0.78	2.97E-01
MSMEG_0665	sarcosine oxidase subunit beta, putative		3.40	3.43E-05	0.76	1.79E-02	0.46	1.11E-03
MSMEG_0666	acetyl-CoA synthetase, putative		1.22	1.83E-01	1.04	6.35E-01	1.15	2.36E-01
MSMEG_0667	butyryl-CoA dehydrogenase		1.68	9.96E-03	0.79	5.06E-02	1.06	8.42E-01
MSMEG_0668	integral membrane protein		1.56	3.79E-02	0.77	1.55E-02	0.90	4.50E-01
MSMEG_0669	hypothetical protein		1.23	2.01E-01	1.09	3.87E-01	0.73	5.44E-02
MSMEG_0670	FAD dependent oxidoreductase		0.72	6.63E-02	1.39	5.13E-02	1.12	4.77E-01
MSMEG_0671	S-(hydroxymethyl)glutathione dehydrogenase		1.23	6.24E-01	2.63	2.85E-02	0.59	6.17E-02
MSMEG_0672	conserved hypothetical protein		1.70	3.22E-01	2.75	2.54E-02	0.30	6.62E-05
MSMEG_0673	hypothetical protein		1.04	7.09E-01	0.75	1.72E-01	1.27	2.47E-01
MSMEG_0674	ErfK/YbiS/YcfS/YnhG family protein		1.61	8.24E-02	0.63	2.65E-02	1.01	9.56E-01
MSMEG_0675	putative cytochrome P450 144		2.06	1.34E-02	0.79	6.76E-02	1.02	3.71E-01

MSMEG_0676	putative transcriptional regulatory protein		1.08	4.80E-01	0.99	9.41E-01	2.23	6.59E-02
MSMEG_0678	deoxycytidine triphosphate deaminase	*dcd*	0.57	5.71E-04	0.94	7.54E-01	1.13	6.17E-01
MSMEG_0679	conserved hypothetical protein		1.68	3.55E-03	0.80	1.65E-02	1.24	4.38E-01
MSMEG_0680	UDP-glucose 6-dehydrogenase		1.17	4.01E-01	2.68	1.29E-01	1.04	2.24E-01
MSMEG_0681	P450 heme-thiolate protein		1.76	6.57E-03	0.88	1.06E-01	0.75	1.01E-01
MSMEG_0682	conserved hypothetical protein, putative		2.51	1.72E-02	1.05	4.94E-01	0.25	8.96E-05
MSMEG_0683	conserved hypothetical protein		1.50	1.09E-03	0.75	2.31E-02	0.56	1.09E-02
MSMEG_0684	aldehyde oxidase and xanthine dehydrogenase, molybdopterin binding		1.05	8.96E-01	1.91	8.53E-03	0.81	2.06E-01
MSMEG_0685	oxidoreductase, molybdopterin-binding subunit		1.32	4.47E-01	2.34	5.25E-02	0.58	1.36E-01
MSMEG_0686	oxidoreductase		1.01	9.03E-01	0.84	2.14E-02	0.64	2.43E-04
MSMEG_0687	conserved hypothetical protein		0.42	2.00E-04	0.97	6.18E-01	1.31	5.81E-01
MSMEG_0688	aspartate aminotransferase		0.63	2.15E-03	1.02	8.66E-01	0.90	2.70E-01
MSMEG_0689	hypothetical protein		3.68	1.03E-03	0.35	1.50E-04	0.48	6.10E-02
MSMEG_0690	iron-sulfur cluster-binding protein		0.54	1.96E-04	0.91	2.94E-01	3.69	7.97E-03
MSMEG_0691	putative transcriptional regulatory protein		0.54	4.31E-04	0.87	1.09E-01	2.05	1.69E-02
MSMEG_0692	conserved hypothetical protein		0.50	3.76E-04	0.94	3.43E-01	1.52	5.68E-02
MSMEG_0693	conserved hypothetical protein		2.07	8.30E-06	0.58	8.50E-03	1.16	2.57E-01
MSMEG_0694	conserved hypothetical protein; this gene contains a frame shift which is not the result of sequencing error		2.28	5.88E-05	0.44	6.69E-03	0.70	1.28E-01
MSMEG_0695	isoniazid inductible protein IniA		1.31	7.11E-03	0.59	7.47E-03	1.15	3.75E-01
MSMEG_0696	alanine-rich protein		1.55	2.48E-01	1.43	6.44E-02	0.54	3.59E-02
MSMEG_0697	integral membrane protein		1.77	4.47E-01	2.18	5.40E-03	0.52	5.85E-02
MSMEG_0698	isoniazid inductible protein IniC		0.69	1.40E-02	0.96	5.49E-01	1.48	7.96E-02
MSMEG_0699	conserved hypothetical proline rich protein		2.32	1.28E-02	0.61	2.51E-03	3.42	8.74E-02
MSMEG_0700	hypothetical protein		0.63	3.40E-02	1.21	1.86E-01	0.99	9.65E-01
MSMEG_0701	hypothetical protein		1.10	5.30E-01	1.15	3.54E-01	1.10	4.84E-01
MSMEG_0702	monooxygenase		1.09	2.26E-01	0.69	3.14E-02	0.97	5.51E-01
MSMEG_0703	conserved hypothetical protein		2.03	2.21E-03	0.74	2.07E-02	1.48	1.41E-01
MSMEG_0704	LpqJ protein		0.99	9.79E-01	0.73	3.95E-02	2.54	8.86E-02
MSMEG_0705	putative permease of the major facilitator superfamily protein		1.23	6.23E-02	1.56	4.52E-01	1.73	3.96E-01
MSMEG_0706	4-carboxy-4-hydroxy-2-oxoadipate aldolase		0.88	5.33E-01	0.93	5.44E-01	1.06	6.92E-01
MSMEG_0707	conserved hypothetical protein		1.26	3.65E-01	1.10	5.07E-01	1.19	1.94E-01
MSMEG_0708	transcriptional regulator, LysR family protein		0.89	2.39E-01	0.91	1.70E-01	1.27	5.10E-01
MSMEG_0709	chaperone protein DnaK	*dnaK*	1.35	4.73E-02	2.33	8.73E-02	1.40	1.70E-01
MSMEG_0710	co-chaperone GrpE	*grpE*	1.38	5.79E-02	1.36	1.91E-01	1.40	1.80E-01

MSMEG_0711	chaperone protein DnaJ	*dnaJ*	1.19	1.46E-02	1.60	1.52E-01	1.77	3.80E-02
MSMEG_0712	transcriptional regulator, TetR family protein		0.53	5.83E-04	0.99	9.33E-01	1.19	5.37E-01
MSMEG_0713	transcriptional regulator, MerR family protein		0.94	4.23E-01	3.08	1.35E-01	1.70	5.85E-02
MSMEG_0714	acyl-CoA dehydrogenase		1.51	2.28E-01	1.03	7.51E-01	0.52	2.28E-03
MSMEG_0715	3-oxoacyl-[acyl-carrier-protein] reductase		1.43	4.83E-02	1.11	5.44E-01	1.06	3.75E-01
MSMEG_0716	major facilitator superfamily protein		1.97	1.12E-02	5.00	1.09E-01	0.88	6.25E-01
MSMEG_0717	aldose 1-epimerase subfamily protein		0.93	2.82E-01	0.97	8.64E-01	0.56	8.55E-04
MSMEG_0718	acetyl-CoA synthetase		1.14	2.62E-01	4.08	6.34E-02	0.96	7.52E-01
MSMEG_0719	flavohemoprotein		0.57	6.55E-03	0.96	7.10E-01	1.74	4.51E-02
MSMEG_0720	integral membrane protein		1.28	3.11E-02	0.85	7.38E-02	1.22	2.71E-01
MSMEG_0721	RemQ protein; this gene contains a frame shift which is not the result of sequencing error		0.90	3.07E-01	2.64	9.73E-02	0.84	1.85E-01
MSMEG_0721	RemQ protein; this gene contains a frame shift which is not the result of sequencing error		1.10	4.84E-01	1.96	1.63E-01	0.99	6.46E-01
MSMEG_0722	conserved hypothetical protein		1.30	2.16E-01	1.04	7.94E-01	1.04	1.62E-01
MSMEG_0723	conserved hypothetical protein		0.93	2.99E-01	0.97	7.21E-01	1.25	4.47E-02
MSMEG_0725	IS1137, transposase orfB		0.68	9.44E-02	1.82	2.38E-01	1.22	4.31E-01
MSMEG_0726	hypothetical protein		0.91	2.59E-01	1.08	4.35E-01	0.51	4.99E-02
MSMEG_0727	hypothetical protein		0.39	1.54E-04	1.05	7.87E-01	0.94	2.69E-01
MSMEG_0728	hypothetical protein		1.39	8.03E-02	0.93	4.49E-01	0.49	2.80E-02
MSMEG_0729	conserved hypothetical protein		0.69	3.46E-02	1.01	9.60E-01	1.13	2.72E-01
MSMEG_0730	oleandomycin glycosyltransferase		0.79	1.56E-01	1.08	6.95E-01	1.07	4.92E-01
MSMEG_0731	conserved hypothetical protein		1.03	8.19E-01	1.17	4.72E-01	1.31	4.94E-01
MSMEG_0732	chaperone ClpB		2.36	2.25E-03	5.78	9.70E-02	1.39	1.70E-02
MSMEG_0733	dihydrodipicolinate reductase, N-terminus domain protein	*dapB*	0.90	8.67E-02	1.44	1.81E-01	0.63	4.41E-02
MSMEG_0734	Rieske [2Fe-2S] domain protein		1.08	5.26E-01	1.36	4.22E-02	0.64	8.11E-02
MSMEG_0735	putative transcriptional regulator		0.89	4.27E-01	15.15	7.79E-02	0.97	6.98E-01
MSMEG_0736	conserved hypothetical protein		0.84	2.32E-02	0.97	7.49E-01	0.96	6.96E-01
MSMEG_0737	dehydrogenase		0.76	1.61E-02	2.19	1.16E-01	1.53	2.65E-03
MSMEG_0738	conserved hypothetical protein		0.30	2.62E-05	4.15	1.01E-01	1.97	1.65E-02
MSMEG_0739	spou rRNA methylase family protein		0.36	2.37E-05	5.52	1.01E-01	2.26	8.68E-03
MSMEG_0740	glycosyl hydrolase family protein 76		0.78	5.36E-03	2.21	1.21E-01	1.11	8.30E-02
MSMEG_0741	conserved hypothetical protein		0.93	3.71E-01	2.01	1.73E-01	1.05	8.41E-01
MSMEG_0742	probable transcriptional regulatory protein		1.51	4.43E-03	0.78	7.27E-03	1.61	3.30E-02
MSMEG_0743	xanthine dehydrogenase		1.78	1.82E-02	1.43	1.25E-01	1.02	8.42E-01
MSMEG_0744	carbon monoxide dehydrogenase medium chain		8.47	1.50E-02	2.40	6.61E-02	0.15	4.17E-05
MSMEG_0745	[2Fe-2S] binding domain protein		3.44	5.02E-04	1.89	8.01E-02	0.33	6.18E-04

MSMEG_0746	carbon-monoxide dehydrogenase, large subunit		6.92	5.79E-03	2.10	6.51E-02	0.21	2.40E-06
MSMEG_0747	carbon monoxide dehydrogenase F protein		5.77	1.14E-02	1.89	1.29E-01	0.53	7.52E-03
MSMEG_0748	ATPase associated with various cellular activities, AAA_5		2.32	1.30E-02	3.10	9.54E-02	0.85	1.76E-01
MSMEG_0749	carbon monoxide dehydrogenase subunit G (CoxG) family protein		1.98	4.35E-03	1.65	8.09E-02	0.92	4.06E-01
MSMEG_0750	membrane protein		0.76	7.60E-03	0.95	1.00E-01	1.11	4.97E-01
MSMEG_0751	conserved hypothetical protein		1.22	4.81E-01	2.86	1.58E-01	0.97	5.55E-01
MSMEG_0752	fructose-bisphosphate aldolase, class II	*fbaA*	1.40	7.97E-03	1.29	1.58E-01	0.83	3.20E-02
MSMEG_0753	conserved hypothetical protein		0.89	5.13E-02	0.84	7.11E-02	0.55	8.90E-04
MSMEG_0754	conserved hypothetical protein		1.05	1.50E-01	0.98	7.12E-01	4.95	3.35E-01
MSMEG_0755	cobalt-zinc-cadmium resistance protein		3.31	3.40E-03	0.75	4.79E-02	0.76	1.33E-01
MSMEG_0756	peptidase, M50B family protein		0.60	3.06E-03	0.93	6.17E-01	0.84	3.90E-01
MSMEG_0757	hypothetical protein		0.81	1.87E-01	5.38	1.26E-01	2.01	1.83E-01
MSMEG_0758	conserved hypothetical protein		0.65	7.68E-02	1.14	4.36E-01	1.34	2.67E-01
MSMEG_0759	adenylosuccinate synthetase	*purA*	1.05	6.09E-01	0.78	2.05E-04	1.10	4.10E-02
MSMEG_0760	thioesterase family protein		0.67	6.66E-06	1.37	1.35E-02	21.39	3.54E-01
MSMEG_0761	conserved domain protein		2.61	1.20E-04	0.65	9.37E-03	0.91	2.83E-01
MSMEG_0762	cytochrome P450 FAS1		2.92	8.16E-03	0.70	4.50E-03	0.81	2.58E-01
MSMEG_0763	antibiotic transporter		0.83	3.46E-01	1.25	5.17E-02	1.32	2.01E-01
MSMEG_0764	Na+/H+ antiporter		0.46	4.48E-04	1.21	3.86E-02	1.05	7.95E-01
MSMEG_0765	zinc finger, UBP-type		1.03	7.19E-01	1.09	2.98E-01	0.79	3.04E-01
MSMEG_0766	phosphoribosylglycinamide formyltransferase 2	*purT*	0.74	1.82E-02	0.99	9.64E-01	1.96	1.23E-02
MSMEG_0767	hypothetical protein		0.48	3.60E-04	1.41	3.21E-02	2.08	8.00E-02
MSMEG_0768	conserved hypothetical protein		0.61	1.08E-03	1.02	8.44E-01	2.07	1.81E-02
MSMEG_0769	O-succinylhomoserine sulfhydrylase	*metZ*	0.51	5.70E-05	1.11	2.64E-01	2.24	1.77E-03
MSMEG_0770	hypothetical protein		0.43	1.19E-05	1.15	5.00E-01	2.06	4.02E-02
MSMEG_0771	hypothetical oxidoreductase YqjQ		1.68	2.30E-03	0.84	1.36E-01	0.81	1.85E-01
MSMEG_0772	phytase		1.18	9.55E-02	1.09	4.31E-01	1.21	1.35E-01
MSMEG_0773	acetyltransferase		3.12	1.20E-02	1.70	1.35E-01	1.20	5.09E-01
MSMEG_0774	flavin-dependent oxidoreductase; this gene contains a frame shift which is not the result of sequencing error		3.54	1.02E-02	1.40	2.95E-01	0.89	1.71E-01
MSMEG_0775	metallo-beta-lactamase superfamily protein		1.34	4.67E-02	0.96	6.33E-01	0.50	7.90E-04
MSMEG_0776	conserved hypothetical protein		1.05	5.69E-01	1.15	2.28E-01	5.13	2.98E-02
MSMEG_0777	F420-dependent glucose-6-phosphate dehydrogenase		0.95	3.25E-01	0.97	7.48E-01	0.73	7.47E-03
MSMEG_0778	putative transcriptional regulator		0.93	2.70E-01	1.05	4.88E-02	1.03	7.36E-01

MSMEG_0779	short-chain dehydrogenase/reductase SDR		0.18	7.98E-05	0.92	5.57E-01	0.81	8.60E-02
MSMEG_0780	phosphotransferase enzyme family protein		1.01	9.17E-01	0.92	2.05E-01	1.07	8.42E-01
MSMEG_0781	amino acid permease		0.22	1.07E-04	0.98	7.62E-01	1.00	9.77E-01
MSMEG_0782	aminotransferase class-III		0.36	1.07E-02	1.55	1.88E-01	1.10	4.82E-01
MSMEG_0783	phosphate acetyltransferase	*pta*	0.89	1.95E-01	1.22	5.62E-02	2.98	1.94E-03
MSMEG_0784	acetate kinase	*ackA*	0.68	8.33E-03	1.34	1.08E-01	2.32	1.49E-01
MSMEG_0785	conserved hypothetical protein		0.75	4.57E-02	1.40	3.38E-01	1.01	9.40E-01
MSMEG_0786	serine/threonine protein kinase		1.91	2.10E-02	0.85	3.65E-02	3.93	1.46E-04
MSMEG_0787	Bacterial extracellular solute-binding protein, family protein 3		1.57	6.17E-02	0.70	9.48E-03	5.10	1.76E-02
MSMEG_0788	putative conserved membrane protein		1.96	6.81E-03	0.85	2.77E-02	2.85	2.39E-02
MSMEG_0789	thiamine-phosphate pyrophosphorylase	*thiE*	0.48	5.85E-04	0.87	2.20E-01	0.75	2.01E-01
MSMEG_0790	hydrolase, NUDIX family protein		1.07	2.43E-01	0.80	9.14E-02	0.99	9.65E-01
MSMEG_0791	glycine oxidase ThiO	*thiO*	1.19	3.09E-02	0.66	1.90E-02	1.17	2.98E-01
MSMEG_0792	thiamine biosynthesis protein ThiS	*thiS*	1.21	5.52E-03	0.65	4.62E-03	0.85	3.20E-01
MSMEG_0793	thiazole biosynthesis protein ThiG	*thiG*	1.01	8.35E-01	0.78	1.64E-02	1.03	8.69E-01
MSMEG_0794	conserved hypothetical protein		0.55	9.63E-04	0.95	5.76E-01	1.28	1.43E-01
MSMEG_0795	ABC transporter ATP-binding protein		0.80	1.18E-01	0.78	9.72E-02	1.95	9.60E-03
MSMEG_0796	ABC transporter integral membrane subunit		0.96	3.44E-01	1.01	9.16E-01	1.11	5.39E-01
MSMEG_0797	conserved hypothetical protein; this gene contains a frame shift which is not the result of sequencing error		0.85	6.80E-02	1.22	3.92E-02	1.26	1.56E-01
MSMEG_0797	conserved hypothetical protein; this gene contains a frame shift which is not the result of sequencing error		1.27	4.64E-01	0.96	6.06E-01	0.94	6.97E-01
MSMEG_0798	hypothetical protein		1.14	4.50E-01	0.99	8.95E-01	1.24	5.36E-01
MSMEG_0799	hypothetical protein		1.18	2.37E-02	0.74	3.21E-03	0.33	1.31E-04
MSMEG_0800	hypothetical protein		0.90	1.11E-01	0.79	6.38E-02	1.15	5.36E-01
MSMEG_0803	hypothetical protein		1.92	5.12E-02	0.61	4.37E-02	1.44	1.97E-01
MSMEG_0803	hypothetical protein		0.95	5.96E-01	1.10	4.36E-01	0.94	6.89E-01
MSMEG_0804	hypothetical protein		1.13	2.16E-01	1.09	3.56E-01	1.23	3.49E-01
MSMEG_0805	conserved hypothetical protein		0.62	6.92E-04	1.07	5.90E-01	1.11	1.77E-01
MSMEG_0806	hydrolase		0.68	5.70E-02	0.95	8.32E-01	1.29	6.13E-03
MSMEG_0807	leupeptin-inactivating enzyme 1		0.38	9.23E-04	1.13	1.78E-01	2.01	2.24E-02
MSMEG_0808	possible chalcone synthase Pks10		1.74	2.73E-01	1.20	2.83E-01	1.01	9.48E-01
MSMEG_0809	isoprenylcysteine carboxyl methyltransferase		1.19	2.21E-01	2.18	2.92E-01	1.09	5.82E-01
MSMEG_0810	putative pyrimidine permease RutG		0.74	5.79E-03	0.86	1.54E-01	0.89	6.47E-01
MSMEG_0811	oxidoreductase		1.11	5.50E-01	1.31	2.03E-01	0.98	8.01E-01

MSMEG_0812	amino acid transporter		1.18	2.93E-01	0.94	5.54E-01	1.10	4.39E-01
MSMEG_0813	conserved hypothetical protein		1.53	2.64E-02	0.91	3.80E-01	1.93	1.12E-01
MSMEG_0814	membrane protein		1.08	4.75E-01	0.79	6.49E-02	1.45	5.53E-02
MSMEG_0815	transcriptional regulator, TetR family protein		1.66	4.44E-04	0.96	6.14E-01	0.99	9.11E-01
MSMEG_0816	flavin-binding monooxygenase		2.93	4.60E-04	0.71	2.16E-02	1.02	7.68E-01
MSMEG_0817	LysR-family protein transcriptional regulator		1.17	5.62E-01	1.11	2.58E-01	0.94	2.67E-01
MSMEG_0818	transporter, major facilitator family protein		1.80	1.14E-02	0.85	1.35E-01	1.31	4.16E-01
MSMEG_0819	N-carbamoyl-L-amino acid amidohydrolase		1.09	6.50E-01	0.95	7.71E-01	0.94	2.84E-01
MSMEG_0820	conserved hypothetical protein		11.59	6.16E-02	0.56	3.35E-02	0.92	7.48E-01
MSMEG_0821	glutamyl-tRNA(Gln) amidotransferase subunit A		1.30	1.98E-01	0.95	6.85E-01	1.00	9.48E-01
MSMEG_0822	cytochrome P450		5.76	3.95E-02	0.65	8.07E-02	0.59	9.76E-02
MSMEG_0823	conserved hypothetical protein		0.66	2.74E-03	0.97	9.21E-01	1.29	4.39E-01
MSMEG_0824	conserved hypothetical protein		0.65	1.01E-03	0.85	8.26E-02	0.56	3.89E-04
MSMEG_0825	phosphomethylpyrimidine kinase	*thiD*	0.60	2.88E-03	0.63	2.57E-03	0.48	2.52E-03
MSMEG_0826	thiamine biosynthesis protein ThiC	*thiC*	0.81	7.90E-02	0.72	2.07E-02	0.52	5.72E-03
MSMEG_0827	major facilitator superfamily protein MFS_1		1.09	8.09E-01	1.91	1.61E-01	1.18	4.27E-01
MSMEG_0828	immunogenic protein MPT63		2.52	6.71E-03	0.83	1.70E-01	0.36	5.58E-03
MSMEG_0829	exodeoxyribonuclease III	*xth*	0.47	5.59E-03	0.92	5.03E-01	1.26	9.52E-02
MSMEG_0830	acetyltransferase, gnat family protein		0.47	5.97E-03	1.15	6.11E-01	1.36	3.80E-01
MSMEG_0831	short chain dehydrogenase		1.26	2.39E-01	1.34	2.13E-01	1.23	1.01E-01
MSMEG_0832	peptide deformylase	*def*	0.44	1.59E-04	1.05	4.55E-01	1.13	4.07E-01
MSMEG_0833	conserved hypothetical protein		0.86	2.53E-01	1.12	4.03E-01	1.27	3.29E-01
MSMEG_0834	tuberculin related peptide		0.59	1.83E-03	0.96	5.40E-01	1.58	1.19E-02
MSMEG_0835	copper/zinc superoxide dismutase	*sodC*	0.43	1.91E-06	0.83	7.13E-02	1.22	4.26E-01
MSMEG_0836	carboxylate-amine ligase		0.30	2.19E-05	1.29	1.91E-02	2.46	6.58E-02
MSMEG_0837	hypothetical protein		0.74	3.62E-01	1.08	4.58E-01	1.38	2.72E-01
MSMEG_0838	AsnC-family protein transcriptional regulator		0.63	9.49E-06	0.90	3.66E-01	1.38	2.40E-01
MSMEG_0839	ATP-dependent protease La (LON) domain subfamily protein		1.68	7.83E-03	0.88	3.01E-01	0.96	7.14E-01
MSMEG_0840	hypothetical protein		0.42	1.19E-05	1.13	1.87E-01	0.47	3.39E-05
MSMEG_0841	hypothetical protein; this gene contains a frame shift which is not the result of sequencing error; identified by Glimmer2; putative		0.72	5.99E-03	1.01	7.97E-01	0.47	1.62E-04
MSMEG_0842	hypothetical protein		0.73	1.74E-03	1.20	2.01E-01	0.46	1.54E-02
MSMEG_0843	multisubunit Na+/H+ antiporter, G subunit		0.71	8.06E-05	0.80	3.17E-02	0.92	3.79E-01
MSMEG_0844	multiple resistance and pH regulation protein F		0.63	4.29E-06	0.93	5.61E-01	0.80	2.57E-01
MSMEG_0845	multisubunit Na+/H+ antiporter		0.61	1.79E-04	0.86	5.10E-02	1.19	2.99E-01

MSMEG_0846	NADH-ubiquinone oxidoreductase/multisubunit na+/h+ antiporter, d subunit		0.46	2.78E-07	0.70	4.25E-02	1.66	1.20E-01
MSMEG_0847	NADH-ubiquinone/plastoquinone oxidoreductase chain 4l family protein		0.60	6.05E-05	0.88	1.96E-01	1.19	9.76E-02
MSMEG_0848	NADH ubiquinone oxidoreductase subunit 5		0.65	2.95E-03	0.95	6.18E-01	1.08	4.19E-01
MSMEG_0849	oxygenase		0.94	4.07E-01	1.15	4.17E-01	1.07	5.00E-01
MSMEG_0850	conserved hypothetical protein		1.08	4.93E-01	1.29	2.79E-01	0.91	4.48E-01
MSMEG_0851	Citrate transporter		6.63	3.87E-01	1.04	3.34E-01	1.04	6.69E-01
MSMEG_0852	CBS domain protein		1.77	2.67E-02	1.03	8.87E-01	1.09	3.62E-01
MSMEG_0853	conserved hypothetical protein		3.03	3.24E-03	1.37	3.58E-01	1.40	6.80E-02
MSMEG_0854	two-component system sensor kinase		3.82	3.40E-03	0.89	2.16E-01	1.04	7.88E-01
MSMEG_0855	conserved hypothetical protein		1.14	3.34E-01	0.80	9.27E-02	1.03	6.02E-01
MSMEG_0856	DNA-binding response regulator, LuxR family protein		3.17	4.96E-04	0.96	6.71E-01	1.13	1.02E-01
MSMEG_0857	conserved hypothetical protein		0.48	2.85E-03	0.80	2.12E-02	0.93	4.66E-01
MSMEG_0858	cell division control protein Cdc48		0.70	5.52E-03	1.11	2.43E-01	2.16	8.70E-02
MSMEG_0859	transcriptional regulator, TetR family protein		0.93	4.64E-01	0.77	1.30E-02	1.33	1.29E-01
MSMEG_0860	CDP-diacylglycerol--serine O-phosphatidyltransferase		0.67	2.08E-04	0.83	8.61E-02	1.26	1.29E-01
MSMEG_0861	phosphatidylserine decarboxylase	*psd*	0.64	6.65E-04	1.04	6.72E-01	1.57	1.82E-02
MSMEG_0862	molybdopterin biosynthesis protein MoeA		0.59	8.25E-05	0.85	8.64E-02	0.83	1.76E-01
MSMEG_0863	short chain dehydrogenase		0.75	2.41E-03	0.94	2.59E-01	0.57	2.98E-03
MSMEG_0864	bacterial membrane flanked domain family		0.56	1.52E-05	0.90	2.84E-01	0.95	6.73E-01
MSMEG_0865	bacterial membrane flanked domain family		1.06	6.28E-01	0.93	5.43E-01	1.00	9.63E-01
MSMEG_0866	DNA or RNA helicase of superfamily protein II		0.79	2.31E-02	0.79	1.31E-01	2.20	3.77E-02
MSMEG_0867	hydrolase		0.80	8.40E-03	0.93	4.86E-01	1.18	1.35E-01
MSMEG_0868	hypothetical protein		1.22	1.60E-01	1.19	1.65E-01	1.05	3.01E-01
MSMEG_0869	hypothetical protein		1.01	8.88E-01	1.15	3.15E-01	1.00	9.38E-01
MSMEG_0870	oxidoreductase		2.27	2.46E-01	1.02	8.80E-01	1.06	3.57E-01
MSMEG_0871	putative aldehyde or xanthine dehydrogenase, molybdopterin binding subunit protein		2.54	3.88E-01	0.89	5.78E-01	1.15	2.17E-02
MSMEG_0872	twin-arginine translocation pathway signal		1.77	2.35E-01	0.89	4.05E-01	1.01	3.92E-02
MSMEG_0873	hypothetical protein		1.12	2.09E-01	1.05	6.13E-01	1.00	8.69E-01
MSMEG_0874	transcriptional regulator, GntR family protein		0.77	1.83E-02	0.96	5.25E-01	1.44	2.39E-01
MSMEG_0875	putative sialic acid transporter		1.49	1.36E-03	0.64	1.63E-04	1.57	4.26E-01
MSMEG_0876	short chain dehydrogenase		0.83	7.31E-02	0.84	3.45E-01	0.58	8.34E-03
MSMEG_0877	dihydrodipicolinate synthase		1.20	1.49E-01	0.70	5.13E-03	1.38	1.59E-01
MSMEG_0878	transcriptional regulator		1.51	3.34E-02	0.75	1.64E-01	0.77	5.40E-02

MSMEG_0879	hypothetical protein		0.85	2.19E-01	0.76	7.47E-02	0.99	8.81E-01
MSMEG_0880	chaperonin GroL	*groL*	0.62	7.12E-04	0.84	4.52E-02	0.41	2.52E-05
MSMEG_0881	conserved hypothetical protein		0.57	7.99E-04	0.87	5.26E-02	1.19	3.75E-01
MSMEG_0882	conserved hypothetical protein		2.04	7.14E-05	1.09	1.16E-01	0.42	1.06E-03
MSMEG_0883	amidohydrolase family protein		1.65	3.16E-03	0.91	6.38E-02	0.87	4.45E-01
MSMEG_0884	glyoxalase family protein		0.91	5.92E-01	0.92	3.48E-01	1.37	3.72E-01
MSMEG_0885	conserved hypothetical protein		2.22	4.33E-02	0.90	4.69E-01	1.03	8.60E-01
MSMEG_0886	serine/threonine-protein kinase PknD		2.08	6.93E-04	1.19	6.28E-01	0.82	8.72E-03
MSMEG_0887	conserved hypothetical protein		1.47	2.77E-03	0.74	2.52E-02	1.15	5.69E-01
MSMEG_0888	conserved hypothetical protein		0.85	7.99E-02	1.09	3.63E-01	1.91	2.83E-02
MSMEG_0889	succinic semialdehyde dehydrogenase		1.63	1.47E-02	0.89	1.93E-01	0.16	1.95E-05
MSMEG_0890	HD domain protein		0.78	1.69E-03	0.90	2.10E-01	1.08	5.71E-01
MSMEG_0891	polyphosphate kinase 2		0.67	4.40E-02	0.96	8.20E-01	0.95	3.46E-01
MSMEG_0892	conserved hypothetical protein		0.61	1.83E-03	0.84	8.45E-02	1.08	9.60E-02
MSMEG_0893	hypothetical protein		1.23	3.18E-02	1.01	8.72E-01	1.08	4.37E-01
MSMEG_0894	dihydrodipicolinate reductase		1.79	7.57E-03	1.07	6.66E-01	0.50	6.74E-02
MSMEG_0895	bacterial regulatory protein, GntR family protein		1.23	5.37E-04	0.77	2.51E-02	0.92	2.28E-01
MSMEG_0896	integral membrane transport protein		1.25	1.89E-02	0.85	3.43E-03	0.97	8.30E-01
MSMEG_0897	conserved hypothetical protein		1.27	2.27E-02	0.80	5.49E-02	1.39	1.06E-01
MSMEG_0898	tagatose-1,6-bisphosphate aldolase GatY		1.22	2.12E-02	0.84	2.92E-01	1.41	6.72E-02
MSMEG_0899	prolyl oligopeptidase family protein		0.64	1.18E-04	0.81	8.69E-02	1.60	5.34E-02
MSMEG_0900	eptc-inducible aldehyde dehydrogenase		2.83	3.24E-03	1.07	6.45E-01	0.20	1.42E-04
MSMEG_0901	conserved hypothetical protein		1.45	2.32E-02	0.90	1.52E-01	0.30	2.95E-04
MSMEG_0902	cyclopropane-fatty-acyl-phospholipid synthase 1		0.94	3.47E-01	1.19	2.45E-01	1.20	6.18E-01
MSMEG_0903	dihydrolipoamide dehydrogenase	*lpdA*	0.45	5.17E-04	1.14	1.54E-02	0.71	5.19E-03
MSMEG_0904	probable conserved membrane protein		0.27	4.02E-06	1.06	5.95E-01	1.55	3.04E-01
MSMEG_0905	4-carboxymuconolactone decarboxylase domain protein	*pcaC*	1.22	2.47E-02	0.78	1.35E-02	0.73	6.56E-02
MSMEG_0906	DNA-binding protein		0.53	2.40E-05	0.93	2.06E-02	0.98	8.70E-01
MSMEG_0907	conserved hypothetical protein		0.83	2.87E-01	1.00	9.71E-01	1.01	6.62E-01
MSMEG_0908	hypothetical protein		0.86	6.78E-02	1.09	2.71E-01	0.95	5.06E-01
MSMEG_0909	acyl-ACP thioesterase superfamily protein		1.07	3.22E-01	0.89	4.15E-01	1.01	9.48E-01
MSMEG_0910	hypothetical protein		0.99	9.03E-01	1.11	5.82E-01	1.53	4.43E-01
MSMEG_0911	isocitrate lyase	*aceA*	0.25	4.75E-05	1.36	3.54E-01	1.29	1.26E-01
MSMEG_0912	3-hydroxybutyryl-CoA dehydrogenase		0.50	1.00E-03	0.80	5.02E-03	0.78	4.84E-01
MSMEG_0913	methoxy mycolic acid synthase 1		0.83	2.40E-03	0.74	6.65E-02	0.85	1.99E-01
MSMEG_0914	conserved hypothetical protein		0.73	3.84E-02	1.06	2.86E-01	1.00	9.14E-01
MSMEG_0915	polyphosphate kinase 2 superfamily protein		1.15	2.02E-01	0.67	7.15E-02	1.81	6.90E-02

MSMEG_0916	transcriptional regulator, TetR family protein		2.11	6.16E-04	0.89	1.48E-01	0.82	2.00E-02
MSMEG_0917	conserved hypothetical protein		1.53	4.34E-02	0.93	5.63E-01	0.90	6.55E-01
MSMEG 0918	transcriptional regulator, XRE family protein		1.84	5.83E-02	0.80	1.12E-01	1.36	5.43E-01
MSMEG 0919	heparin-binding hemagglutinin		1.78	1.07E-02	0.93	4.50E-01	1.91	2.95E-01
MSMEG_0920	conserved hypothetical protein		1.43	5.57E-03	0.94	2.67E-01	0.77	3.84E-04
MSMEG_0921	conserved hypothetical protein		0.72	2.59E-03	0.92	2.69E-01	1.29	2.31E-02
MSMEG 0922	deoxyribose-phosphate aldolase	*deoC*	0.66	6.42E-03	0.81	1.79E-01	1.94	1.01E-02
MSMEG 0923	conserved hypothetical protein		0.50	3.86E-05	0.81	1.02E-01	1.20	3.40E-01
MSMEG_0924	hydrolase, carbon-nitrogen family protein		1.19	6.12E-02	0.71	3.05E-02	1.43	3.11E-01
MSMEG_0925	conserved hypothetical protein		0.83	1.05E-02	1.22	2.46E-02	1.10	1.02E-01
MSMEG 0926	conserved hypothetical protein		1.02	7.01E-01	0.87	3.57E-01	1.49	1.28E-01
MSMEG 0927	conserved hypothetical protein		0.68	5.42E-03	1.16	2.04E-01	1.05	8.22E-01
MSMEG_0928	UDP-N-acetylenolpyruvoylglucosamine	*murB*	0.30	5.99E-06	0.97	5.35E-01	1.23	2.36E-01
MSMEG 0929	ErfK/YbiS/YcfS/YnhG family protein		0.76	1.48E-02	0.77	3.92E-02	1.32	2.43E-01
MSMEG 0930	serine 3-dehydrogenase		0.79	7.94E-02	0.96	7.60E-01	1.36	2.12E-02
MSMEG_0931	hypothetical protein		1.26	7.84E-02	0.82	7.32E-02	0.98	7.11E-01
MSMEG_0932	ROK family protein		0.69	1.06E-02	1.70	1.75E-01	12.21	1.67E-01
MSMEG 0933	conserved hypothetical protein		1.28	2.58E-03	1.15	5.47E-01	1.65	5.78E-03
MSMEG 0934	conserved hypothetical protein		0.87	1.63E-02	0.81	5.77E-02	1.63	1.15E-01
MSMEG_0935	2,3-bisphosphoglycerate-dependent phosphoglycerate mutase		0.60	6.08E-04	1.03	6.91E-01	0.82	3.63E-01
MSMEG_0936	sensor histidine kinase SenX3	*senX3*	1.50	4.80E-02	1.00	9.46E-01	2.04	6.57E-03
MSMEG 0937	DNA-binding response regulator RegX3	*regX3*	1.41	8.29E-03	0.89	1.67E-01	1.84	4.29E-03
MSMEG 0938	conserved hypothetical protein		0.84	3.43E-03	0.87	8.53E-02	1.56	5.77E-04
MSMEG_0939	Ppx/GppA phosphatase family protein		1.00	9.53E-01	0.90	4.57E-02	1.23	1.72E-01
MSMEG_0940	conserved hypothetical protein		0.78	1.70E-03	0.80	1.57E-02	1.47	1.29E-02
MSMEG 0941	AP endonuclease, family protein 2		0.25	3.61E-06	1.00	9.23E-01	3.24	4.68E-04
MSMEG 0942	conserved hypothetical protein		0.36	8.77E-05	1.02	7.60E-01	1.76	7.35E-02
MSMEG_0943	pyrroline-5-carboxylate reductase	*proC*	0.35	9.08E-05	0.98	8.20E-01	2.00	2.34E-01
MSMEG 0944	DNA binding domain, excisionase family protein		1.38	1.21E-03	0.64	1.49E-02	0.71	4.18E-02
MSMEG 0945	conserved domain protein		0.80	1.41E-02	0.86	6.24E-02	1.31	4.27E-01
MSMEG_0946	NAD dependent epimerase/dehydratase family protein		0.97	3.54E-01	0.97	4.49E-01	0.83	9.39E-03
MSMEG_0947	acyltransferase		1.15	4.22E-01	0.81	3.60E-02	0.94	6.95E-01
MSMEG 0948	conserved hypothetical protein		0.56	7.20E-05	1.05	6.12E-01	1.39	6.35E-02
MSMEG_0949	HAD-superfamily protein subfamily protein IB hydrolase, TIGR01490		0.85	3.03E-02	0.83	3.33E-03	2.06	9.47E-03
MSMEG_0950	hypothetical protein		0.80	2.28E-01	1.28	1.77E-01	3.30	3.85E-01

MSMEG_0951	glutaredoxin 2		0.57	9.69E-05	0.99	8.18E-01	1.73	1.58E-02
MSMEG_0952	glutamyl-tRNA reductase	*hemA*	1.08	1.18E-01	1.03	6.16E-01	2.03	1.14E-03
MSMEG_0953	porphobilinogen deaminase	*hemC*	0.49	3.82E-04	0.87	2.99E-02	2.58	1.08E-02
MSMEG_0954	uroporphyrinogen-III synthase	*hemD*	0.49	2.06E-04	0.86	5.07E-02	2.34	2.96E-02
MSMEG_0955	hypothetical protein		0.99	8.85E-01	0.96	8.62E-01	1.50	3.35E-01
MSMEG_0956	delta-aminolevulinic acid dehydratase	*hemB*	0.46	3.51E-06	0.81	5.50E-02	3.75	1.67E-02
MSMEG_0957	hypothetical protein		0.37	1.16E-06	0.99	8.53E-01	4.45	2.09E-02
MSMEG_0958	hypothetical protein		0.96	6.84E-01	0.78	4.71E-03	1.86	7.24E-02
MSMEG_0959	putative conserved transmembrane protein		0.78	1.11E-02	0.92	4.26E-01	1.19	3.87E-01
MSMEG_0960	putative transmembrane protein		0.66	7.66E-04	1.02	3.83E-01	1.23	4.69E-01
MSMEG_0961	conserved hypothetical protein; this gene contains a frame shift which is not the result of sequencing error		5.71	1.91E-03	0.33	1.08E-03	0.61	7.60E-02
MSMEG_0962	TetR-family protein transcriptional regulator		2.64	8.34E-04	0.48	3.12E-03	1.07	7.90E-01
MSMEG_0963	hypothetical protein		1.22	3.29E-01	1.40	1.73E-03	0.82	2.31E-01
MSMEG_0964	pyridoxamine 5'-phosphate oxidase family		1.00	9.85E-01	1.06	5.36E-01	0.71	1.07E-03
MSMEG_0965	porin		0.61	2.12E-04	1.14	1.91E-01	0.70	3.12E-02
MSMEG_0966	conserved hypothetical protein		1.24	1.98E-01	0.80	3.95E-02	1.06	5.12E-01
MSMEG_0967	conserved hypothetical protein		1.66	2.01E-02	0.63	5.18E-03	0.88	2.19E-01
MSMEG_0968	cytochrome P450		2.52	8.70E-03	0.97	7.85E-01	0.39	3.36E-03
MSMEG_0969	glutamate-1-semialdehyde-2,1-aminomutase	*hemL*	0.44	4.72E-05	0.84	1.66E-01	4.55	1.35E-02
MSMEG_0970	phosphoglycerate mutase family protein		0.30	1.28E-06	0.85	4.11E-02	6.06	2.06E-02
MSMEG_0971	conserved hypothetical protein		0.35	3.22E-06	1.11	5.23E-01	5.73	9.17E-03
MSMEG_0972	cytochrome C biogenesis protein transmembrane region		0.41	5.75E-05	0.99	9.33E-01	8.66	3.74E-02
MSMEG_0973	conserved membrane protein		0.60	3.80E-04	1.05	3.46E-01	4.03	2.30E-02
MSMEG_0974	cytochrome c-type biogenesis protein CcsB	*ccsB*	0.41	1.55E-04	0.85	5.06E-02	2.03	4.30E-02
MSMEG_0975	conserved hypothetical protein		1.04	6.26E-01	0.92	3.24E-01	1.45	3.06E-03
MSMEG_0976	conserved hypothetical protein		3.43	2.85E-03	0.74	1.87E-02	1.26	4.57E-02
MSMEG_0977	conserved hypothetical protein		1.07	4.37E-01	0.90	2.70E-01	1.65	1.41E-02
MSMEG_0978	aminotransferase, class V superfamily protein		0.42	4.35E-05	0.92	2.35E-01	2.46	1.15E-01
MSMEG_0979	keratin associated protein		0.81	4.46E-02	0.79	7.40E-03	1.54	3.01E-01
MSMEG_0980	signal transduction histidine kinase		1.91	1.91E-01	1.59	2.78E-01	2.08	3.96E-01
MSMEG_0981	two-component system regulator		1.80	9.12E-02	0.94	6.29E-01	1.15	3.86E-01
MSMEG_0982	immunogenic protein		2.14	3.52E-03	1.23	1.49E-01	0.45	8.53E-04
MSMEG_0983	two-component system response regulator		1.32	1.66E-02	0.84	1.97E-01	0.57	8.17E-03
MSMEG_0984	trap transporter, 4tm/12tm fusion protein		1.66	3.53E-03	1.14	3.74E-01	0.65	8.59E-02
MSMEG_0985	sugar transporter family protein		0.32	3.62E-05	1.02	8.83E-01	1.87	1.74E-01

MSMEG_0986	RemM protein		0.76	3.49E-04	0.93	4.17E-01	0.54	7.05E-04
MSMEG_0987	hypothetical protein		2.79	1.98E-03	0.71	5.73E-03	0.66	3.47E-03
MSMEG_0988	1,4-dihydroxy-2-naphthoate octaprenyltransferase	*menA*	0.95	8.01E-01	0.77	2.01E-01	1.13	1.76E-01
MSMEG_0989	conserved hypothetical protein		1.19	2.18E-01	0.78	5.83E-02	1.10	4.89E-01
MSMEG_0990	methylthioadenosine phosphorylase	*mtaP*	1.13	3.27E-01	0.94	6.31E-01	1.52	4.60E-03
MSMEG_0991	NAD-dependent epimerase/dehydratase		1.01	6.97E-01	1.28	5.05E-02	1.31	2.07E-01
MSMEG_0992	2-nitropropane dioxygenase, NPD		1.40	9.50E-02	0.96	4.11E-01	1.22	3.15E-01
MSMEG_0993	histidine kinase		1.40	9.63E-04	1.07	2.85E-01	0.74	1.20E-01
MSMEG_0994	DNA-binding response regulator ResD	*resD*	1.23	2.78E-02	0.79	8.10E-02	0.96	6.54E-01
MSMEG_0995	glycosyl transferase, family protein 2		3.95	2.89E-04	0.65	9.21E-03	1.04	3.63E-01
MSMEG_0996	conserved hypothetical protein		3.58	1.75E-02	0.87	1.58E-01	1.44	1.48E-01
MSMEG_0997	conserved hypothetical protein		1.88	2.20E-02	1.59	4.35E-01	1.26	7.41E-02
MSMEG_0998	oxidoreductase, molybdopterin binding		1.38	1.95E-01	0.96	3.45E-01	1.07	2.29E-01
MSMEG_0999	integral membrane protein		0.92	4.65E-01	0.80	6.70E-02	0.84	2.69E-01
MSMEG_1000	hypothetical protein		1.70	2.04E-02	0.98	5.15E-01	1.00	8.69E-01
MSMEG_1001	acetyltransferase, gnat family protein, putative		0.76	3.54E-02	1.29	7.98E-02	1.33	5.95E-01
MSMEG_1004	This is the transposed copy of the gene fragment that was disrupted by an IS1096 element elsewhere in the chromosome.; conserved hypothetical protein, interruption-N; identified by similarity to OMNI:NTL02MT01721		0.99	9.66E-01	1.06	7.10E-01	0.88	7.26E-01
MSMEG_1060	putative Lsr2 protein		1.72	6.51E-04	0.71	1.09E-02	0.84	1.18E-01
MSMEG_1061	phosphohydrolase		1.21	1.19E-01	0.89	1.14E-01	0.47	2.48E-05
MSMEG_1062	O-succinylbenzoic acid--CoA ligase		0.93	1.88E-01	0.88	3.27E-01	2.05	2.46E-02
MSMEG_1063	conserved hypothetical protein		1.06	5.55E-01	0.88	3.18E-01	1.96	1.68E-02
MSMEG_1064	phosphate/sulphate permease		1.17	1.34E-01	0.71	1.48E-02	2.53	9.40E-03
MSMEG_1065	conserved hypothetical protein		0.76	6.68E-03	0.86	2.01E-02	3.04	4.83E-03
MSMEG_1066	conserved hypothetical protein		1.05	7.49E-01	0.86	4.52E-02	3.33	1.99E-03
MSMEG_1067	conserved hypothetical protein		1.65	4.73E-04	0.84	1.93E-02	1.08	2.95E-01
MSMEG_1068	regulatory protein		1.09	4.33E-01	0.89	1.17E-01	1.83	6.30E-02
MSMEG_1069	amino acid permease-associated region		1.18	2.38E-01	0.88	6.66E-02	1.22	4.36E-01
MSMEG_1070	3-oxoacyl-[acyl-carrier-protein] reductase		1.73	2.77E-02	1.02	8.27E-01	1.11	3.20E-01
MSMEG_1071	3-oxoacyl-[acyl-carrier-protein] reductase		1.47	1.38E-02	1.02	7.92E-01	0.96	4.20E-01
MSMEG_1072	agmatinase	*speB*	1.14	3.37E-01	1.02	7.86E-01	1.02	8.71E-01
MSMEG_1073	oxidoreductase, short-chain dehydrogenase/reductase family protein		0.54	1.09E-03	0.96	4.62E-01	0.97	8.75E-01
MSMEG_1074	polysaccharide deacetylase		0.92	2.09E-01	1.00	9.62E-01	1.10	7.48E-01
MSMEG_1075	naphthoate synthase	*menB*	0.76	1.38E-01	0.96	6.41E-01	1.33	5.19E-02

MSMEG_1076	conserved hypothetical protein		1.24	6.61E-01	3.17	4.86E-02	0.48	1.67E-02
MSMEG_1077	conserved hypothetical protein		1.40	1.66E-03	0.82	1.59E-01	1.33	4.65E-02
MSMEG_1078	hydrolase		1.14	5.64E-01	1.03	8.96E-01	1.17	4.00E-01
MSMEG_1079	hypothetical protein		0.97	7.90E-01	1.49	2.24E-01	1.67	3.90E-01
MSMEG_1080	large subunit of N,N-dimethylformamidase		0.96	6.06E-01	1.05	8.04E-01	1.23	3.49E-01
MSMEG_1081	hypothetical protein		1.13	1.77E-01	1.01	9.37E-01	0.88	5.33E-01
MSMEG_1082	putative response regulator		0.17	7.44E-05	0.91	6.72E-01	1.11	5.30E-01
MSMEG_1083	hypothetical protein		0.84	1.85E-01	1.11	3.93E-01	0.91	5.18E-01
MSMEG_1084	peptide/opine/nickel uptake family protein ABC transporter, periplasmic substrate-binding protein, putative		0.98	9.02E-01	1.10	3.86E-01	1.45	3.74E-01
MSMEG_1085	dipeptide transport system permease protein DppB		1.10	5.27E-01	1.06	5.97E-01	0.93	6.55E-01
MSMEG_1086	ABC transporter permease protein		1.01	9.71E-01	1.00	9.95E-01	0.82	9.11E-02
MSMEG_1087	oligopeptide ABC transporter ATP-binding protein; this gene contains a frame shift which is not the result of sequencing error; identified by match to protein family HMM PF00005		1.41	3.88E-01	0.81	2.92E-01	1.04	2.35E-01
MSMEG_1087	oligopeptide ABC transporter ATP-binding protein; this gene contains a frame shift which is not the result of sequencing error; identified by match to protein family HMM PF00005		0.94	8.16E-01	1.16	4.14E-01	2.33	4.04E-01
MSMEG_1088	glutamyl-tRNA(Gln)/aspartyl-tRNA(Asn) amidotransferase, A subunit	*gatA*	1.72	3.57E-01	1.08	6.24E-01	0.79	1.75E-01
MSMEG_1089	hypothetical protein		1.57	9.17E-02	0.99	9.26E-01	0.61	1.04E-02
MSMEG_1090	amidase		1.46	1.74E-01	0.74	1.37E-01	0.38	1.57E-05
MSMEG_1091	urease accessory protein UreE 1		1.17	4.91E-01	1.11	5.93E-01	0.99	3.54E-01
MSMEG_1092	urease accessory protein UreF		1.08	6.59E-01	1.08	5.41E-01	0.99	9.07E-01
MSMEG_1093	urease gamma/beta subunit		1.37	1.78E-01	0.88	2.64E-01	1.01	8.54E-01
MSMEG_1094	urease, alpha subunit	*ureC*	1.34	3.60E-01	0.96	3.53E-01	1.09	2.11E-01
MSMEG_1095	urease accessory protein UreG	*ureG*	1.10	3.29E-01	0.99	8.12E-01	1.06	1.84E-01
MSMEG_1096	urease accessory protein UreD, putative		1.01	9.18E-01	1.47	1.03E-01	1.06	6.61E-01
MSMEG_1097	glycosyl transferase, group 2 family protein		1.09	8.51E-01	2.22	2.50E-02	0.33	2.29E-03
MSMEG_1098	acyl-CoA synthase		1.74	1.71E-02	0.95	7.24E-01	0.75	6.74E-02
MSMEG_1099	hypothetical protein		1.15	4.83E-01	0.99	9.42E-01	0.96	6.37E-01
MSMEG_1100	dgpf protein		2.25	4.57E-03	0.87	7.50E-02	0.93	1.12E-01
MSMEG_1101	hypothetical protein		2.26	7.22E-02	0.62	5.79E-03	1.05	8.73E-01
MSMEG_1102	twin-arginine translocation pathway signal		0.94	6.70E-01	1.17	2.56E-01	0.86	4.60E-01

MSMEG_1103	O-succinylbenzoate synthase	0.90	1.74E-01	0.90	4.65E-01	1.46	1.34E-01
MSMEG_1104	hypothetical protein	9.11	1.04E-01	0.47	2.57E-02	1.21	5.90E-01
MSMEG_1105	phthiodiolone ketoreductase; this gene contains a frame shift which is not the result of sequencing error	9.14	3.82E-02	0.56	2.97E-02	1.60	1.35E-01
MSMEG_1105	phthiodiolone ketoreductase; this gene contains a frame shift which is not the result of sequencing error	1.02	8.53E-01	1.00	9.71E-01	0.95	8.84E-01
MSMEG_1106	transcriptional regulator, AraC family protein	2.20	1.91E-02	1.29	4.13E-01	0.91	5.30E-01
MSMEG_1107	isonitrile hydratase, putative	1.55	1.37E-02	1.20	6.24E-01	1.02	9.26E-01
MSMEG_1108	hydrolase, alpha/beta fold family protein	0.57	7.61E-05	0.91	2.79E-02	0.87	4.49E-01
MSMEG_1109	2-oxoglutarate decarboxylase	0.81	4.68E-02	0.79	1.12E-02	1.53	2.49E-04
MSMEG_1110	probable conserved transmembrane protein	0.43	1.65E-04	1.15	1.62E-01	1.07	5.12E-01
MSMEG_1111	conserved hypothetical protein	0.97	6.23E-01	0.91	6.75E-01	1.15	5.39E-01
MSMEG_1112	aconitate hydratase, putative	1.58	2.34E-01	2.40	2.14E-02	0.55	5.88E-02
MSMEG_1113	glycosyl transferase	1.23	1.62E-01	1.03	8.95E-01	0.98	7.31E-01
MSMEG_1114	short chain dehydrogenase	3.94	1.82E-03	0.70	6.55E-02	0.84	4.09E-01
MSMEG_1115	menaquinone biosynthesis methyltransferase UbiE	0.54	6.28E-04	1.19	4.84E-01	1.19	1.37E-01
MSMEG_1116	gamma-glutamylisopropylamide synthetase	1.34	4.80E-02	0.92	1.86E-01	0.90	1.22E-01
MSMEG_1117	transcriptional regulator	1.31	1.16E-02	0.83	1.78E-02	0.87	9.27E-02
MSMEG_1118	amino acid permease	0.60	1.15E-02	1.09	3.55E-01	1.10	4.97E-01
MSMEG_1119	glutamine amidotransferase, class I	1.16	5.82E-01	0.72	1.03E-01	1.51	3.59E-01
MSMEG_1120	nitrilase/cyanide hydratase and apolipoprotein N-acyltransferase	0.79	5.34E-03	1.95	2.58E-01	1.01	8.79E-01
MSMEG_1121	conserved hypothetical protein	0.46	1.47E-05	1.22	1.01E-01	11.63	3.74E-01
MSMEG_1122	hypothetical protein	0.87	3.98E-02	0.81	7.44E-02	0.89	2.00E-01
MSMEG_1123	cobalamin synthesis protein	0.72	2.01E-02	1.23	3.16E-01	1.20	5.10E-01
MSMEG_1124	putative ferredoxin FdxA	1.85	3.12E-02	0.88	1.90E-01	1.42	2.60E-01
MSMEG_1125	regulatory protein, ArsR	1.13	7.94E-02	1.32	2.88E-01	1.09	3.99E-01
MSMEG_1126	hypothetical protein	3.03	2.57E-01	0.88	1.81E-02	1.03	8.80E-01
MSMEG_1127	probable conserved transmembrane protein	1.80	1.23E-03	1.10	5.66E-01	1.73	4.00E-01
MSMEG_1128	conserved hypothetical protein	4.71	1.06E-03	0.69	2.56E-02	0.97	7.12E-01
MSMEG_1129	D-amino-acid dehydrogenase	1.51	1.62E-01	1.00	9.96E-01	1.13	4.84E-01
MSMEG_1130	hypothetical protein	1.57	2.25E-01	0.96	3.50E-01	1.44	1.24E-01
MSMEG_1131	tryptophan-rich sensory protein	0.88	7.30E-01	1.24	1.42E-01	14.60	3.56E-01
MSMEG_1132	FAD binding domain, putative	1.09	6.45E-01	0.91	2.76E-01	1.19	2.25E-01

MSMEG_1133	bifunctional short chain isoprenyl diphosphate synthase	0.75	7.68E-04	0.67	8.50E-03	1.21	4.49E-01
MSMEG_1134	putative protease HtpX	1.13	1.38E-01	1.50	1.22E-01	1.14	2.13E-01
MSMEG_1135	hypothetical protein	1.16	1.38E-01	1.44	3.14E-01	1.01	9.10E-01
MSMEG_1136	conserved hypothetical protein	0.99	9.03E-01	1.05	4.79E-01	0.41	4.81E-04
MSMEG_1137	amino acid permease-associated region	1.17	5.05E-01	0.94	4.84E-01	1.07	7.80E-01
MSMEG_1138	alcohol dehydrogenase 1	2.33	1.18E-02	0.86	1.88E-01	0.97	6.38E-01
MSMEG_1139	transcription regulator	1.48	1.74E-01	0.91	1.45E-01	1.10	2.79E-01
MSMEG_1140	glycerol-3-phosphate dehydrogenase 2 [NAD(P)+] (NAD(P)H-dependent glycerol-3-phosphate dehydrogenase 2)	2.19	9.84E-03	1.08	3.36E-01	0.40	1.89E-02
MSMEG_1141	ABC-transporter integral membrane protein	1.39	3.77E-01	0.89	3.48E-01	0.84	4.15E-01
MSMEG_1142	ABC-transporter integral membrane protein	1.05	8.39E-01	1.12	4.59E-01	0.85	2.24E-01
MSMEG_1143	mce related protein	1.22	4.37E-02	0.97	8.13E-01	0.68	9.82E-02
MSMEG_1144	virulence factor Mce family protein	1.09	6.56E-01	0.91	8.84E-02	0.64	9.44E-02
MSMEG_1145	virulence factor Mce family protein	0.95	5.18E-01	1.01	7.76E-01	0.78	2.16E-01
MSMEG_1146	virulence factor Mce family protein	1.24	6.36E-02	1.10	1.02E-01	0.88	2.48E-01
MSMEG_1147	mce related protein	0.98	7.71E-01	0.92	2.82E-01	0.61	1.84E-02
MSMEG_1148	mce related protein	1.06	5.37E-01	1.04	6.94E-01	0.84	1.92E-01
MSMEG_1149	conserved hypothetical protein	1.12	3.55E-02	0.92	1.63E-01	0.71	9.01E-02
MSMEG_1150	conserved hypothetical protein	1.10	1.76E-01	0.88	6.09E-02	0.89	2.12E-01
MSMEG_1151	DNA-binding protein	0.53	1.56E-02	1.09	4.64E-01	1.09	5.48E-01
MSMEG_1152	citrate-proton symporter	1.10	4.35E-01	0.73	8.26E-03	0.63	5.36E-02
MSMEG_1153	FAD dependent oxidoreductase	0.63	4.05E-02	1.31	2.22E-01	0.97	8.27E-01
MSMEG_1154	formyl-coenzyme A transferase	0.57	1.27E-02	1.03	8.26E-01	1.08	6.76E-01
MSMEG_1155	carnitinyl-CoA dehydratase	0.46	3.05E-04	1.86	3.96E-01	1.00	9.90E-01
MSMEG_1156	dihydrodipicolinate synthetase	0.43	8.38E-04	1.01	9.49E-01	0.89	1.95E-01
MSMEG_1157	short chain dehydrogenase	0.63	1.57E-02	1.38	1.53E-01	1.03	6.37E-01
MSMEG_1158	5-oxovalerate dehydrogenase	1.43	6.54E-01	0.97	7.22E-01	1.38	5.44E-01
MSMEG_1159	ABC transporter component A	0.99	9.15E-01	1.35	3.73E-01	0.96	6.59E-01
MSMEG_1160	ABC-type nitrate/sulfonate/bicarbonate transport system ATPase component	1.14	3.49E-01	1.04	5.13E-01	2.18	1.97E-01
MSMEG_1161	taurine transport system permease protein TauC	1.48	2.59E-01	0.99	8.86E-01	0.98	5.72E-01
MSMEG_1162	nitrilotriacetate monooxygenase component A	1.43	3.46E-01	1.36	8.89E-02	1.03	5.15E-01
MSMEG_1163	hypothetical protein	1.42	2.50E-01	1.28	2.32E-01	1.00	7.36E-01
MSMEG_1164	putative transporter; this region contains one or more premature stops and/or frameshifts which are not the result of sequencing error	1.91	1.14E-01	1.30	1.26E-02	1.08	7.71E-02

MSMEG_1164	putative transporter; this region contains one or more premature stops and/or frameshifts which are not the result of sequencing error		0.89	2.25E-01	0.89	2.59E-01	1.77	1.69E-01
MSMEG_1164	putative transporter; this region contains one or more premature stops and/or frameshifts which are not the result of sequencing error		1.08	4.84E-01	1.03	6.39E-01	1.02	3.97E-01
MSMEG_1165	conserved hypothetical protein		1.32	1.98E-02	0.96	6.42E-01	1.34	6.09E-02
MSMEG_1167	cytochrome P450		6.21	5.89E-04	0.92	5.68E-01	0.85	7.11E-01
MSMEG_1168	putative transcriptional regulator		1.49	3.75E-02	0.95	4.45E-01	1.12	3.72E-01
MSMEG_1169	integral membrane transport protein		0.96	6.68E-01	1.16	9.31E-02	3.05	3.50E-02
MSMEG_1170	conserved domain protein		1.00	9.80E-01	1.30	1.89E-01	1.06	2.43E-01
MSMEG_1171	Low molecular weight phosphotyrosine protein phosphatase		0.83	2.20E-01	1.06	4.81E-01	1.10	4.86E-01
MSMEG_1172	arsenical-resistance protein	*arsB*	0.58	8.68E-03	1.01	8.26E-01	0.87	1.02E-01
MSMEG_1173	transcriptional regulator, ArsR family protein		1.04	5.90E-01	0.91	4.18E-02	0.81	5.31E-02
MSMEG_1174	cadmium inducible protein cadi		2.76	2.17E-05	0.90	3.61E-01	0.63	1.68E-02
MSMEG_1175	putative transcriptional regulatory protein		1.06	5.43E-01	0.78	1.47E-02	1.17	4.20E-01
MSMEG_1176	hypothetical protein		0.39	1.10E-03	1.05	5.27E-01	1.11	3.26E-01
MSMEG_1177	cytosine/purines/uracil/thiamine/allantoin permease family protein		0.91	4.80E-01	1.18	2.49E-01	1.13	4.77E-01
MSMEG_1178	transcriptional regulator		0.90	3.75E-01	1.29	5.29E-02	1.19	4.89E-01
MSMEG_1179	urocanate hydratase	*hutU*	1.01	9.39E-01	1.07	4.67E-01	1.51	1.32E-01
MSMEG_1180	N-carbamoyl-L-amino acid hydrolase		0.88	5.70E-01	0.96	5.91E-01	1.02	7.84E-01
MSMEG_1181	formiminoglutamate deiminase	*hutF*	1.01	9.55E-01	0.98	4.78E-01	1.22	3.30E-01
MSMEG_1182	imidazolonepropionase	*hutI*	0.97	8.30E-01	0.86	9.61E-02	0.98	8.84E-01
MSMEG_1183	histidine ammonia-lyase	*hutH*	0.94	7.64E-01	0.96	4.85E-01	1.20	4.50E-01
MSMEG_1184	serine esterase, cutinase family protein		1.64	1.46E-02	1.43	1.55E-02	0.10	3.78E-08
MSMEG_1185	transcriptional regulator, AsnC family protein		0.86	2.17E-01	1.16	2.57E-01	1.08	4.57E-01
MSMEG_1186	putative permease		1.32	9.34E-02	0.91	9.05E-02	0.93	4.85E-01
MSMEG_1187	N-acyl-D-aspartate deacylase		2.98	5.04E-02	0.98	9.45E-01	1.47	3.09E-01
MSMEG_1188	conserved hypothetical protein		1.31	9.01E-03	0.88	2.66E-02	1.67	2.59E-02
MSMEG_1189	conserved hypothetical protein		1.52	1.80E-01	0.77	5.14E-02	0.73	1.41E-02
MSMEG_1190	serine/threonine-protein kinase PknD		1.67	4.33E-01	1.15	3.34E-01	0.96	2.13E-01
MSMEG_1191	hypothetical protein		0.45	2.51E-05	1.04	6.62E-01	0.99	8.43E-01
MSMEG_1192	M23 peptidase domain protein		2.28	2.09E-03	0.88	4.34E-01	0.97	5.49E-01
MSMEG_1193	TROVE domain protein		1.63	1.03E-01	1.64	2.26E-02	1.02	8.12E-01
MSMEG_1194	hypothetical protein		1.60	9.89E-04	1.20	2.65E-02	0.50	3.26E-02
MSMEG_1195	conserved hypothetical protein		1.21	1.97E-02	0.78	5.84E-02	1.63	4.49E-02

MSMEG_1196	SNF2 domain protein		1.79	3.69E-03	1.74	2.73E-01	1.13	1.98E-01
MSMEG_1197	transcriptional regulator, LuxR family protein		1.56	8.07E-02	1.21	5.73E-02	1.15	3.83E-01
MSMEG_1198	hypothetical protein		1.45	2.38E-02	1.10	2.98E-01	1.14	1.79E-01
MSMEG_1199	conserved hypothetical protein		0.67	6.75E-02	1.12	6.33E-01	1.20	4.73E-01
MSMEG_1200	serine/threonine-protein kinase PknD		1.48	2.95E-02	0.74	2.15E-02	0.87	2.04E-01
MSMEG_1201	hypothetical protein		0.66	1.42E-03	0.89	3.66E-01	1.07	4.48E-01
MSMEG_1202	hypothetical protein		3.53	4.51E-03	1.55	1.65E-01	0.53	1.03E-03
MSMEG_1203	methoxy mycolic acid synthase 1		7.31	6.46E-04	0.80	1.65E-01	0.57	9.67E-04
MSMEG_1204	3-oxoacyl-[acyl-carrier-protein] synthase 2		2.87	6.66E-05	0.95	4.48E-01	1.09	4.63E-01
MSMEG_1205	cyclopropane-fatty-acyl-phospholipid synthase 1		6.40	1.01E-02	0.83	2.43E-01	0.51	1.27E-03
MSMEG_1206	hypothetical protein		2.11	1.23E-01	1.02	9.36E-01	0.89	1.95E-01
MSMEG_1207	glycosyltransferase, group I		2.49	7.04E-02	0.84	3.23E-01	1.09	1.15E-01
MSMEG_1208	glycosyltransferase, group I		1.56	7.55E-03	0.82	3.54E-02	1.16	2.63E-01
MSMEG_1209	glycosidase		1.89	2.15E-01	0.97	3.25E-01	1.38	2.86E-01
MSMEG_1210	conserved hypothetical protein		0.74	8.05E-02	0.99	9.46E-01	0.97	7.92E-01
MSMEG_1211	Fatty acid desaturase		1.49	3.10E-02	0.76	6.28E-03	0.82	3.14E-02
MSMEG_1212	hypothetical protein		14.66	1.30E-02	2.67	1.02E-01	0.15	2.71E-05
MSMEG_1213	cytochrome P450 monooxygenase		1.90	2.99E-03	1.03	5.86E-01	0.84	2.86E-01
MSMEG_1214	oxidoreductase		1.17	2.06E-01	0.97	7.30E-01	1.01	8.70E-01
MSMEG_1215	serine/threonine-protein kinase PknE, putative		1.10	3.54E-01	0.99	9.65E-01	1.11	7.22E-01
MSMEG_1216	ABC-type transport system periplasmic substrate-binding protein		2.19	2.43E-02	1.31	1.01E-01	0.25	8.92E-06
MSMEG_1217	ABC-type transport system ATP-binding protein I		1.31	3.63E-01	1.20	9.89E-02	0.82	4.53E-01
MSMEG_1218	ABC-type transport system ATP-binding protein II		1.43	2.54E-02	1.33	7.45E-02	0.68	1.00E-01
MSMEG_1219	ABC-type transport system permease protein II		1.19	3.14E-01	1.11	1.78E-01	0.85	2.10E-01
MSMEG_1220	ABC-type transport system permease protein I		1.89	1.54E-02	1.09	1.74E-01	0.28	1.47E-05
MSMEG_1221	rifampin ADP-ribosyl transferase		1.27	2.67E-01	1.19	3.32E-01	0.97	3.21E-01
MSMEG_1222	ISMsm6, transposase		1.32	3.81E-01	1.12	3.22E-01	1.03	8.23E-01
MSMEG_1223	hypothetical protein		2.22	9.07E-04	0.72	1.06E-02	0.97	6.85E-01
MSMEG_1224	rifampin ADP-ribosyl transferase		1.27	1.28E-01	1.01	5.53E-01	0.71	5.81E-02
MSMEG_1225	conserved hypothetical protein		0.92	7.53E-01	0.79	8.14E-02	1.99	1.50E-01
MSMEG_1226	sulfatase-modifying factor 1		0.48	4.88E-04	1.06	6.15E-01	1.36	4.84E-01
MSMEG_1227	transcriptional regulator, GntR family protein		0.38	5.40E-05	1.06	5.54E-01	2.67	3.93E-02
MSMEG_1228	sulfatase		0.61	9.80E-03	1.08	6.85E-01	0.98	5.13E-01
MSMEG_1229	DNA gyrase, B subunit	*gyrB*	1.05	5.74E-01	1.09	1.54E-02	1.00	9.65E-01
MSMEG_1230	hypothetical protein		1.71	2.74E-01	1.37	1.19E-01	3.03	3.96E-01

MSMEG_1231	inner membrane protein YidH		1.43	1.43E-01	1.01	7.93E-01	0.43	4.33E-03
MSMEG_1232	ABC transporter substrate-binding protein		1.05	8.87E-01	1.16	2.98E-01	1.11	4.54E-01
MSMEG_1233	ABC transporter permease protein; this gene contains a frame shift which is not the result of sequencing error		1.67	2.16E-01	0.96	7.98E-01	1.07	2.45E-01
MSMEG_1234	taurine import ATP-binding protein TauB		0.94	4.52E-01	0.97	5.18E-01	0.89	4.59E-01
MSMEG_1235	sulfate permease		2.73	2.78E-01	0.94	6.96E-01	3.73	3.63E-01
MSMEG_1236	Mpr protein		1.89	2.21E-02	0.94	7.34E-01	1.06	2.02E-01
MSMEG_1237	hypothetical protein		1.94	9.08E-02	0.87	3.67E-01	1.55	1.44E-01
MSMEG_1238	type III restriction enzyme, res subunit		1.17	2.55E-01	0.83	4.16E-02	0.50	6.65E-03
MSMEG_1240	conserved hypothetical protein		1.21	3.02E-02	0.95	6.34E-01	1.29	2.50E-01
MSMEG_1241	conserved hypothetical protein		1.37	4.01E-01	1.06	7.19E-01	1.14	3.28E-01
MSMEG_1242	cysteine desulfurase IscS	*iscS*	0.61	8.31E-03	1.05	4.89E-01	1.29	1.22E-01
MSMEG_1243	conserved hypothetical protein		0.77	2.16E-01	1.04	7.93E-01	1.32	3.19E-01
MSMEG_1244	conserved hypothetical protein		0.60	7.16E-03	1.07	4.59E-01	1.40	3.33E-01
MSMEG_1245	phosphoadenosine phosphosulfate reductase		0.77	2.28E-02	1.20	1.92E-01	0.81	2.78E-01
MSMEG_1246	conserved hypothetical protein		0.51	6.98E-04	1.49	1.47E-02	0.90	6.99E-02
MSMEG_1247	conserved hypothetical protein		0.29	6.70E-05	1.45	1.43E-01	0.89	2.56E-01
MSMEG_1248	hypothetical protein		0.40	2.67E-04	1.35	2.00E-01	0.96	3.69E-01
MSMEG_1249	ISMsm7, transposase orfB		0.51	2.64E-03	1.20	1.21E-01	0.98	9.00E-01
MSMEG_1250	ISMsm7, transposase orfA		0.57	3.51E-03	1.20	2.10E-01	1.11	4.34E-01
MSMEG_1251	conserved hypothetical protein		0.97	8.13E-01	1.04	8.52E-01	1.03	8.93E-01
MSMEG_1252	conserved hypothetical protein		1.34	3.48E-02	0.95	5.28E-01	0.60	3.90E-02
MSMEG_1253	conserved hypothetical protein		1.45	2.09E-02	1.13	3.37E-01	0.32	6.19E-03
MSMEG_1254	DEAD/DEAH box helicase		1.35	7.55E-03	1.11	2.11E-01	0.26	2.59E-03
MSMEG_1255	UvrD/Rep helicase		0.55	1.07E-04	1.21	1.49E-01	0.47	4.90E-03
MSMEG_1256	hypothetical protein		0.65	2.58E-03	1.03	6.90E-01	0.98	7.88E-01
MSMEG_1259	hypothetical protein		1.20	5.42E-01	0.62	7.09E-02	1.62	2.35E-02
MSMEG_1260	hypothetical protein		0.49	6.87E-05	1.05	5.45E-01	0.66	1.67E-01
MSMEG_1261	hypothetical protein		0.58	4.06E-04	1.10	4.52E-01	0.62	7.12E-02
MSMEG_1262	conserved hypothetical protein		0.39	6.75E-04	1.55	1.16E-01	1.72	2.25E-01
MSMEG_1263	conserved hypothetical protein		2.57	8.13E-02	0.73	8.28E-02	1.03	7.82E-01
MSMEG_1264	prophage Lp1 protein 5		1.41	2.74E-01	0.78	4.68E-02	0.96	5.17E-01
MSMEG_1265	conserved hypothetical protein		0.38	9.29E-04	1.13	6.61E-01	0.61	2.02E-01
MSMEG_1266	ankyrin-repeat containing protein		1.26	5.42E-01	1.06	6.80E-01	1.13	5.86E-01

MSMEG_1267	putative ribosylglycohydrolase; this gene contains a frame shift which is not the result of sequencing error; identified by match to protein family HMM PF00782		0.92	6.45E-01	1.25	4.87E-01	1.00	7.47E-01
MSMEG_1268	nudix hydrolase		1.54	1.45E-02	0.60	1.89E-02	1.19	5.11E-01
MSMEG_1269	Ser/Thr protein phosphatase family protein		0.97	7.87E-01	0.91	2.18E-01	1.35	1.39E-01
MSMEG_1270	hypothetical protein		0.74	2.76E-02	1.13	4.15E-01	1.25	2.54E-01
MSMEG_1271	hypothetical protein		1.10	6.29E-01	1.21	2.53E-01	1.39	2.34E-01
MSMEG_1272	putative ribosylglycohydrolase		0.98	9.41E-01	0.77	7.39E-02	1.03	4.65E-01
MSMEG_1273	conserved hypothetical protein		0.59	9.95E-03	1.12	1.89E-01	0.95	4.57E-01
MSMEG_1274	gluconolactonase		2.43	1.77E-01	0.94	5.12E-01	1.52	2.15E-01
MSMEG_1275	HNH nuclease, putative		1.13	1.21E-01	1.04	6.81E-01	0.88	4.93E-01
MSMEG_1276	hypothetical protein		1.07	5.89E-01	1.01	9.60E-01	1.73	2.78E-01
MSMEG_1277	hypothetical protein		0.99	8.94E-01	0.74	1.95E-02	1.16	5.51E-01
MSMEG_1278	death-on-curing protein		0.71	4.74E-02	0.92	3.06E-01	1.03	2.88E-01
MSMEG_1279	conserved hypothetical protein		0.88	1.09E-01	0.72	2.22E-02	1.37	2.21E-01
MSMEG_1280	hypothetical cytosolic protein		1.01	9.12E-01	0.92	2.81E-01	1.08	2.19E-01
MSMEG_1281	conserved hypothetical protein		1.41	5.71E-03	0.99	9.23E-01	2.45	1.43E-01
MSMEG_1282	hypothetical cytosolic protein		1.43	4.05E-02	0.86	1.67E-01	0.84	1.32E-01
MSMEG_1283	probable ribbon-helix-helix transcription factor, family protein		1.15	2.87E-01	0.84	3.38E-02	0.74	3.90E-03
MSMEG_1284	PIN domain protein		1.16	1.40E-01	0.90	1.51E-01	0.89	1.84E-01
MSMEG_1285	tetratricopeptide repeat family protein		0.82	1.82E-02	0.95	3.87E-01	0.97	8.70E-01
MSMEG_1286	sulfatase family protein		2.84	2.12E-03	0.83	1.94E-02	0.28	9.37E-06
MSMEG_1287	cyclase/dehydrase superfamily protein		3.29	2.68E-04	0.81	4.04E-02	0.31	6.19E-05
MSMEG_1288	conserved hypothetical protein		1.05	8.40E-01	1.34	2.89E-01	0.94	3.65E-01
MSMEG_1289	conserved hypothetical protein		1.03	8.18E-01	0.97	8.13E-01	1.03	7.76E-01
MSMEG_1290	dihydroxy-acid dehydratase	*ilvD*	1.08	4.53E-01	1.06	4.40E-01	1.21	4.19E-01
MSMEG_1291	putative oxidoreductase		1.23	2.97E-02	0.80	7.64E-02	2.60	1.53E-02
MSMEG_1292	FAD binding domain in molybdopterin dehydrogenase protein		1.07	3.74E-01	0.83	9.24E-02	1.71	6.02E-02
MSMEG_1293	xanthine/uracil permeases family protein		0.45	2.05E-03	1.29	4.05E-01	0.65	2.20E-02
MSMEG_1294	allantoicase		0.48	1.51E-03	1.43	1.21E-01	0.90	2.74E-01
MSMEG_1295	transthyretin		0.52	6.45E-04	1.16	2.90E-01	0.73	2.31E-01
MSMEG_1296	uricase		0.67	1.97E-02	1.32	1.19E-01	0.79	3.44E-02
MSMEG_1297	hydroxydechloroatrazine ethylaminohydrolase		0.66	1.40E-02	1.27	3.62E-01	1.01	9.70E-01
MSMEG_1298	guanine deaminase		0.86	4.46E-01	0.98	9.27E-01	1.00	7.30E-01

MSMEG_1299	oxidoreductase, 2OG-Fe(II) oxygenase family protein		1.05	7.25E-01	0.82	4.25E-01	0.84	6.47E-02
MSMEG_1300	hypothetical protein		3.75	2.08E-02	1.05	8.38E-01	0.56	7.51E-02
MSMEG_1301	NanT3		1.59	2.03E-01	1.21	2.24E-01	0.91	2.24E-01
MSMEG_1302	alkylphosphonate uptake protein		0.92	2.63E-01	0.91	4.07E-01	0.93	4.09E-01
MSMEG_1303	putative transcriptional regulator		2.24	4.48E-02	1.79	7.66E-02	1.00	9.31E-01
MSMEG_1304	rhizopine catabolism protein		1.17	2.81E-03	1.15	1.92E-01	1.13	1.58E-01
MSMEG_1305	TetR family protein regulatory protein		1.03	7.35E-01	0.97	7.58E-01	1.01	8.94E-01
MSMEG_1306	aldehyde dehydrogenase (NAD) family protein		1.69	1.69E-02	0.94	4.34E-01	0.72	1.16E-01
MSMEG_1307	EthD protein		2.44	5.55E-02	0.87	4.72E-01	0.95	1.21E-01
MSMEG_1308	peptidase S15		2.74	1.24E-01	0.96	2.92E-01	1.18	3.84E-01
MSMEG_1309	conserved hypothetical protein		1.19	6.16E-02	0.91	2.55E-01	1.08	6.23E-01
MSMEG_1311	oligopeptide transporter, OPT family protein		2.06	8.24E-03	0.96	5.67E-01	0.67	3.89E-02
MSMEG_1312	hypothetical protein		2.25	2.04E-01	0.85	9.21E-02	0.96	2.46E-01
MSMEG_1313	secreted protein		0.72	3.19E-02	1.34	9.44E-02	1.11	2.86E-01
MSMEG_1314	hypothetical protein		1.42	2.46E-03	0.91	3.06E-01	1.88	4.71E-01
MSMEG_1315	transporter, small conductance mechanosensitive ion channel (MscS) family protein		0.82	3.74E-01	1.80	1.03E-01	0.75	5.85E-02
MSMEG_1316	peptide chain release factor 3	*prfC*	0.64	2.88E-04	0.75	2.29E-03	2.12	1.25E-01
MSMEG_1317	transcriptional regulator		1.03	8.18E-01	0.76	8.39E-02	1.37	6.04E-02
MSMEG_1318	polysaccharide deacetylase family protein		0.58	1.29E-02	0.86	2.20E-01	0.98	7.86E-01
MSMEG_1319	Asp/Glu racemase		0.70	3.37E-02	1.05	5.49E-01	1.39	3.61E-01
MSMEG_1320	major facilitator superfamily protein MFS_1		0.36	1.30E-03	1.29	1.03E-01	1.10	4.05E-01
MSMEG_1321	5-carboxymethyl-2-hydroxymuconate delta-isomerase		1.31	1.15E-01	1.31	3.86E-01	1.82	1.75E-01
MSMEG_1322	ErfK/YbiS/YcfS/YnhG family protein		0.53	3.78E-03	0.80	5.14E-02	1.63	5.36E-03
MSMEG_1323	hypothetical protein		0.86	3.35E-01	0.95	5.20E-01	1.14	4.46E-01
MSMEG_1324	hypothetical protein		1.14	2.69E-01	1.35	4.27E-01	1.16	5.11E-01
MSMEG_1325	exodeoxyribonuclease V, alpha subunit	*recD*	0.83	1.07E-01	1.52	3.27E-01	1.22	1.48E-01
MSMEG_1326	hypothetical protein		1.46	5.22E-02	0.82	7.25E-02	1.03	8.90E-01
MSMEG_1327	exodeoxyribonuclease V, beta subunit	*recB*	1.06	7.26E-01	0.96	5.67E-01	1.62	1.51E-01
MSMEG_1328	exodeoxyribonuclease V, gamma subunit	*recC*	0.81	2.42E-01	1.00	9.66E-01	1.86	9.67E-02
MSMEG_1329	major facilitator superfamily protein MFS_1, putative		0.88	1.15E-01	0.90	4.20E-01	1.50	2.43E-01
MSMEG_1330	MarR-family protein transcriptional regulator		0.83	1.67E-02	0.93	3.63E-01	1.10	5.32E-01
MSMEG_1331	enoyl-CoA hydratase		0.74	1.87E-02	0.99	9.48E-01	1.32	8.60E-02
MSMEG_1332	conserved hypothetical protein		1.37	6.98E-03	0.90	6.64E-02	0.59	1.85E-02
MSMEG_1333	conserved hypothetical protein		3.19	5.21E-02	0.63	1.19E-01	1.14	6.98E-01

MSMEG_1334	metallo-beta-lactamase family protein		0.48	2.11E-04	0.93	1.56E-01	1.01	7.41E-01
MSMEG_1335	transcriptional regulator, BadM/Rrf2 family protein		1.00	9.91E-01	1.15	2.37E-01	1.41	1.36E-01
MSMEG_1336	flavohemoprotein, putative		0.50	3.58E-03	0.97	7.87E-01	1.33	3.03E-02
MSMEG_1339	ribosomal protein L33	*rpmG*	0.49	5.83E-04	0.97	8.20E-01	1.12	5.21E-01
MSMEG_1340	conserved hypothetical protein		0.44	5.42E-05	0.94	5.82E-01	1.20	3.69E-01
MSMEG_1341	MaoC family protein		0.40	4.97E-05	0.98	7.23E-01	1.22	2.75E-01
MSMEG_1342	conserved hypothetical protein		0.45	3.66E-06	0.91	3.42E-01	1.21	1.88E-01
MSMEG_1344	translocase		0.84	7.81E-02	1.09	5.85E-01	1.31	5.96E-02
MSMEG_1345	transcription antitermination protein NusG		0.64	1.44E-04	0.77	3.49E-02	1.04	5.35E-01
MSMEG_1346	ribosomal protein L11	*rplK*	0.61	9.44E-05	0.84	7.51E-02	1.89	3.99E-02
MSMEG_1347	ribosomal protein L1	*rplA*	0.54	4.71E-04	0.81	5.01E-02	1.02	7.58E-01
MSMEG_1348	RNA polymerase ECF-subfamily protein sigma factor		1.97	2.85E-01	1.06	2.37E-01	1.68	3.82E-01
MSMEG_1349	dgpf domain family protein		0.41	2.32E-04	1.35	2.75E-01	1.79	3.89E-01
MSMEG_1350	cyclopropane-fatty-acyl-phospholipid synthase 1		0.68	3.36E-04	0.95	1.26E-01	0.76	8.52E-02
MSMEG_1351	cyclopropane-fatty-acyl-phospholipid synthase 1		0.95	5.11E-01	0.76	4.44E-03	0.46	6.46E-04
MSMEG_1352	hydrolase, alpha/beta fold family protein		1.22	6.16E-03	0.74	1.30E-02	1.18	1.90E-01
MSMEG_1353	ABC1 family protein		1.84	4.78E-03	0.80	1.64E-02	1.06	5.92E-01
MSMEG_1354	conserved hypothetical protein; this gene contains a frame shift which is not the result of sequencing error		6.86	1.70E-02	0.87	2.50E-01	0.07	1.39E-06
MSMEG_1355	transcriptional regulator, TetR family protein		1.46	5.23E-02	0.91	4.70E-01	0.64	5.98E-02
MSMEG_1356	conserved hypothetical protein		1.47	2.26E-02	0.82	1.30E-01	0.97	8.54E-01
MSMEG_1357	conserved hypothetical protein		1.57	5.79E-03	0.97	6.23E-01	0.79	9.53E-02
MSMEG_1358	conserved hypothetical protein		2.04	4.12E-02	1.79	2.96E-03	0.76	1.30E-01
MSMEG_1359	DeoR-family protein transcriptional regulator		1.03	8.58E-01	1.14	1.47E-01	1.02	9.25E-01
MSMEG_1360	endonuclease/exonuclease/phosphatase		1.27	5.44E-03	0.87	7.02E-02	1.27	3.93E-01
MSMEG_1361	alpha-mannosidase		1.69	2.13E-02	1.21	1.89E-01	0.93	3.69E-01
MSMEG_1362	conserved hypothetical protein		1.43	7.16E-02	1.14	2.39E-01	0.78	7.24E-02
MSMEG_1363	glucokinase		0.87	1.09E-01	0.95	7.80E-01	1.09	5.28E-01
MSMEG_1364	50S ribosomal protein L10		0.65	6.43E-04	0.95	5.53E-01	0.72	1.85E-02
MSMEG_1365	ribosomal protein L7/L12	*rplL*	0.90	1.16E-01	0.70	1.04E-01	0.47	9.21E-03
MSMEG_1366	ABC transporter, ATP-binding protein		1.38	5.75E-02	0.82	1.24E-01	0.22	9.70E-06
MSMEG_1367	DNA-directed RNA polymerase, beta subunit	*rpoB*	1.33	2.86E-03	0.88	3.40E-01	1.82	1.83E-02
MSMEG_1368	DNA-directed RNA polymerase, beta' subunit	*rpoC*	0.96	5.92E-01	0.86	6.58E-02	4.44	4.50E-04
MSMEG_1369	LacI-family protein transcriptional regulator		0.82	4.84E-02	0.79	2.31E-01	1.61	3.35E-01
MSMEG_1370	2-deoxy-scyllo-inosamine dehydrogenase		3.67	1.16E-03	0.77	1.77E-01	0.69	5.99E-02

MSMEG_1371	conserved hypothetical protein		2.93	4.55E-03	0.71	4.18E-02	1.12	3.76E-02
MSMEG_1372	ABC transporter ATP-binding protein		3.93	1.31E-03	0.80	1.79E-01	0.33	1.17E-02
MSMEG_1373	ribose/xylose/arabinose/galactoside ABC-type transport systems, permease components		3.68	3.25E-04	0.92	3.62E-01	0.06	1.38E-07
MSMEG_1374	ribose ABC transporter, periplasmic binding protein		3.36	2.63E-03	0.82	1.34E-01	0.14	3.31E-05
MSMEG_1375	tagatose-1,6-bisphosphate aldolase GatY		1.22	5.77E-02	0.89	5.64E-02	1.04	8.59E-01
MSMEG_1376	putative xylulose kinase		1.18	2.30E-01	0.94	5.68E-01	1.00	9.37E-01
MSMEG_1377	DeoR-family protein transcriptional regulator		1.20	3.72E-01	0.85	7.30E-02	1.16	3.77E-01
MSMEG_1378	1-phosphofructokinase		1.16	3.76E-01	0.86	2.17E-01	1.68	1.71E-01
MSMEG_1379	conserved hypothetical protein		1.38	1.56E-02	1.22	4.53E-02	0.69	1.15E-01
MSMEG_1380	transcriptional regulator		1.08	3.61E-01	0.96	2.77E-01	1.01	9.63E-01
MSMEG_1381	MmpS5 protein		0.51	5.12E-02	0.88	5.03E-01	1.05	2.05E-01
MSMEG_1382	MmpL5 protein		0.68	2.86E-02	0.94	5.49E-01	6.72	3.68E-01
MSMEG_1383	endonuclease IV		0.88	1.72E-02	0.98	7.67E-01	0.95	6.26E-01
MSMEG_1384	conserved hypothetical protein		1.12	5.79E-01	0.83	3.51E-01	3.75	4.65E-01
MSMEG_1385	PAP2 superfamily protein		0.97	6.35E-01	0.74	3.72E-02	1.13	7.26E-01
MSMEG_1386	hypothetical protein		8.23	3.61E-02	0.41	1.83E-02	0.92	7.60E-01
MSMEG_1387	putative acyl-CoA dehydrogenase		1.78	1.78E-05	0.98	8.34E-01	1.11	4.73E-01
MSMEG_1388	enoyl-CoA hydratase		1.98	1.81E-03	1.12	4.88E-01	1.57	4.32E-02
MSMEG_1389	conserved hypothetical protein		1.44	1.58E-02	0.83	3.50E-01	2.56	6.00E-02
MSMEG_1390	enoyl-CoA hydratase		2.15	1.39E-02	0.94	6.63E-01	1.45	1.58E-01
MSMEG_1391	transcriptional regulator, LysR family protein		0.60	3.17E-03	1.06	4.07E-01	1.97	4.24E-01
MSMEG_1392	alcohol dehydrogenase, class IV		1.06	2.68E-01	1.64	3.88E-01	1.50	3.43E-01
MSMEG_1393	conserved hypothetical protein		0.68	7.26E-04	0.96	2.56E-01	0.82	6.91E-02
MSMEG_1394	probable conserved transmembrane protein		0.58	4.59E-04	1.09	2.56E-01	0.72	1.81E-02
MSMEG_1395	lipoprotein, putative		1.01	9.07E-01	0.96	5.39E-01	0.84	3.82E-02
MSMEG_1396	LprB protein		0.60	1.89E-03	1.00	9.31E-01	1.48	2.06E-01
MSMEG_1397	transcriptional regulator, TetR family protein		0.58	1.08E-04	1.13	5.64E-02	1.26	4.56E-01
MSMEG_1398	ribosomal protein S12	*rpsL*	0.51	4.58E-05	1.04	6.33E-01	1.04	8.10E-01
MSMEG_1399	ribosomal protein S7	*rpsG*	0.34	4.58E-06	0.89	1.92E-01	1.09	6.44E-01
MSMEG_1400	elongation factor G; this gene contains a frame shift which is not the result of sequencing error; identified by match to protein family HMM PF00009; match to protein family HMM TIGR00231		0.36	5.32E-07	1.21	8.31E-02	0.79	4.49E-02

MSMEG_1400	elongation factor G; this gene contains a frame shift which is not the result of sequencing error; identified by match to protein family HMM PF00009; match to protein family HMM TIGR00231		0.32	5.37E-06	0.92	2.98E-01	1.11	3.29E-01
MSMEG_1401	translation elongation factor Tu	*tuf*	0.76	3.50E-03	0.95	5.96E-01	0.77	3.19E-04
MSMEG_1402	hypothetical protein		0.50	3.41E-04	0.80	5.85E-02	0.75	7.87E-02
MSMEG_1403	cutinase superfamily protein		0.89	1.75E-01	0.71	8.16E-02	1.28	1.17E-01
MSMEG_1406	conserved hypothetical protein		0.87	6.27E-01	0.92	7.05E-01	1.08	5.76E-01
MSMEG_1407	Na(+)/H(+) antiporter; this gene contains a frame shift which is not the result of sequencing error		1.54	4.29E-02	1.28	8.39E-02	1.13	1.12E-01
MSMEG_1407	Na(+)/H(+) antiporter; this gene contains a frame shift which is not the result of sequencing error		0.91	4.05E-01	1.05	3.64E-01	0.98	8.49E-01
MSMEG_1408	arginine/ornithine antiporter; this region contains one or more premature stops and/or frameshifts which are not the result of sequencing error		1.14	2.33E-02	0.86	1.07E-01	1.26	1.66E-02
MSMEG_1408	arginine/ornithine antiporter; this region contains one or more premature stops and/or frameshifts which are not the result of sequencing error		0.87	8.85E-02	2.04	2.80E-01	1.12	1.48E-01
MSMEG_1408	arginine/ornithine antiporter; this region contains one or more premature stops and/or frameshifts which are not the result of sequencing error		1.01	8.44E-01	1.09	3.71E-01	0.92	4.67E-01
MSMEG_1409	arginine deiminase	*arcA*	1.21	1.15E-01	1.46	1.71E-01	1.10	3.46E-01
MSMEG_1410	carveol dehydrogenase		1.52	2.15E-02	0.86	2.37E-01	0.41	1.52E-03
MSMEG_1411	universal stress protein family protein		2.19	7.23E-02	1.25	4.46E-01	0.93	1.47E-02
MSMEG_1412	amino acid permease		1.79	3.90E-03	1.09	6.85E-01	0.74	2.52E-01
MSMEG_1413	ornithine--oxo-acid transaminase	*rocD*	2.09	1.32E-01	1.33	2.52E-01	0.64	2.60E-03
MSMEG_1414	Amidinotransferase		1.70	1.23E-01	1.31	2.66E-01	0.69	1.90E-01
MSMEG_1415	AsnC-family protein transcriptional regulator		0.82	3.42E-01	1.32	3.99E-01	0.91	4.15E-01
MSMEG_1416	Pyridine nucleotide-disulphide oxidoreductase		0.46	2.38E-04	1.09	2.96E-01	0.87	5.85E-01
MSMEG_1417	glyoxalase family protein		1.36	2.59E-02	1.25	1.14E-01	0.65	2.78E-02
MSMEG_1418	RNA polymerase ECF-type sigma factor		1.20	1.83E-01	1.33	2.78E-02	0.69	2.93E-03
MSMEG_1419	conserved hypothetical protein		0.99	9.41E-01	1.10	4.69E-01	0.86	1.55E-01
MSMEG_1420	probable transcriptional regulatory protein		0.82	2.46E-01	0.90	3.19E-01	1.10	4.89E-01
MSMEG_1421	hypothetical protein		1.19	3.82E-01	1.26	1.09E-01	0.42	1.65E-02
MSMEG_1422	conserved hypothetical protein		1.12	5.53E-01	1.33	8.43E-03	0.32	5.89E-04
MSMEG_1423	radical SAM domain protein		1.03	8.56E-01	1.62	2.26E-02	0.57	5.12E-02
MSMEG_1424	FMN-dependent dehydrogenase		1.23	3.20E-01	1.23	9.48E-02	0.42	3.05E-03

MSMEG_1425	creatininase subfamily protein		2.33	9.72E-03	1.33	1.93E-01	0.49	2.25E-05
MSMEG_1426	probable membrane sugar transferase		2.06	1.30E-02	0.94	5.71E-01	0.61	8.59E-03
MSMEG_1427	transmembrane efflux protein		2.57	5.21E-02	1.09	7.57E-01	0.99	7.48E-01
MSMEG_1428	glucose-methanol-choline oxidoreductase		0.68	5.15E-03	0.89	2.40E-01	1.46	7.21E-02
MSMEG_1429	cytochrome P450-terp		1.43	6.89E-02	0.84	2.62E-01	0.55	6.62E-03
MSMEG_1430	transcriptional regulator, TetR family protein		0.76	7.80E-02	0.91	6.84E-01	0.88	4.44E-01
MSMEG_1431	cytochrome P450-terp		0.91	2.25E-01	1.14	4.81E-01	1.09	1.56E-01
MSMEG_1432	acetylcholinesterase		0.60	3.84E-03	1.06	4.23E-01	0.99	9.36E-01
MSMEG_1433	thioesterase		1.00	9.73E-01	1.02	7.76E-01	1.06	8.42E-01
MSMEG_1434	conserved hypothetical protein		0.54	3.64E-06	1.11	3.78E-01	0.97	5.30E-01
MSMEG_1435	ribosomal protein S10	*rpsJ*	0.74	1.43E-04	0.81	2.06E-02	0.65	1.94E-04
MSMEG_1436	ribosomal protein L3	*rplC*	0.42	6.05E-05	0.87	1.59E-01	0.66	2.46E-04
MSMEG_1437	ribosomal protein L4/L1 family protein	*rplD*	0.58	6.23E-05	0.78	6.05E-03	0.67	1.06E-04
MSMEG_1438	ribosomal protein L23	*rplW*	0.37	4.06E-06	0.78	4.39E-02	0.34	7.93E-05
MSMEG_1439	ribosomal protein L2	*rplB*	0.45	1.22E-04	0.98	9.00E-01	0.60	2.26E-03
MSMEG_1440	ribosomal protein S19	*rpsS*	0.43	8.24E-05	1.00	9.85E-01	0.78	1.61E-02
MSMEG_1441	50S ribosomal protein L22		0.36	7.43E-05	0.96	5.98E-01	0.97	8.51E-01
MSMEG_1442	ribosomal protein S3	*rpsC*	0.44	2.26E-05	0.82	8.00E-02	0.85	7.79E-02
MSMEG_1443	ribosomal protein L16	*rplP*	0.45	6.98E-05	0.90	2.52E-01	0.55	2.91E-03
MSMEG_1444	ribosomal protein L29	*rpmC*	0.40	5.45E-05	0.97	8.23E-01	0.47	4.58E-03
MSMEG_1445	30S ribosomal protein S17		0.43	2.41E-04	0.87	2.80E-01	0.61	5.75E-03
MSMEG_1446	NTP pyrophosphohydrolase		0.29	3.41E-06	1.00	9.99E-01	0.98	4.95E-01
MSMEG_1447	transcriptional regulator, MerR family protein		0.61	1.13E-02	1.09	6.20E-02	1.62	4.67E-01
MSMEG_1448	integral membrane transporter		0.57	3.15E-02	0.92	2.40E-01	1.43	3.42E-01
MSMEG_1449	fasciclin domain protein; this gene contains a frame shift which is not the result of sequencing error; identified by match to protein family HMM PF02469		1.56	1.37E-01	0.96	6.53E-01	1.13	6.51E-01
MSMEG_1450	hypothetical protein		1.24	8.14E-02	1.18	4.82E-01	0.96	6.06E-01
MSMEG_1451	arylsulfatase		0.99	8.99E-01	1.40	1.48E-02	1.01	8.88E-01
MSMEG_1452	sulfatase-modifying factor 1		1.04	8.15E-01	1.09	2.83E-01	1.06	6.81E-01
MSMEG_1453	Citrate transporter		1.45	3.50E-03	0.92	5.26E-01	1.13	4.91E-01
MSMEG_1454	hypothetical protein		0.97	7.08E-01	1.14	4.40E-01	1.33	3.99E-01
MSMEG_1455	putative multidrug resistance protein		1.03	8.20E-01	1.73	4.40E-01	0.98	8.60E-01
MSMEG_1456	hypothetical protein		1.27	2.00E-01	1.01	9.07E-01	0.88	4.28E-01
MSMEG_1457	conserved hypothetical protein		0.84	1.34E-01	1.22	1.70E-01	0.95	2.49E-01
MSMEG_1458	tena/thi-4 family protein		1.44	2.65E-01	0.92	5.14E-01	0.99	4.55E-01
MSMEG_1459	Rieske [2Fe-2S] domain protein		0.92	9.16E-03	0.87	2.40E-01	0.91	4.84E-01

MSMEG_1460	Pyridine nucleotide-disulphide oxidoreductase		0.99	9.51E-01	1.31	3.15E-01	1.00	7.51E-01
MSMEG_1461	5-oxovalerate dehydrogenase		1.20	5.72E-01	1.24	6.74E-02	1.06	3.34E-01
MSMEG_1462	ferredoxin		1.01	9.30E-01	1.17	2.31E-01	1.10	5.67E-01
MSMEG_1463	gamma-glutamyltranspeptidase		1.55	3.36E-01	1.09	4.04E-01	1.44	2.50E-01
MSMEG_1464	translation initiation inhibitor		0.97	7.80E-01	1.07	1.96E-01	1.02	4.37E-01
MSMEG_1465	ribosomal protein L14	*rplN*	0.51	1.04E-05	0.95	4.17E-01	0.83	8.13E-02
MSMEG_1466	ribosomal protein L24	*rplX*	0.70	6.15E-03	0.78	6.29E-03	0.73	1.16E-03
MSMEG_1467	50S ribosomal protein L5		0.35	1.65E-06	0.97	7.18E-01	0.57	9.14E-06
MSMEG_1468	ribosomal protein S14p/S29e	*rpsN*	0.44	1.59E-05	0.94	4.87E-01	0.65	1.66E-03
MSMEG_1469	ribosomal protein S8	*rpsH*	0.53	5.82E-05	0.93	4.78E-01	0.75	6.79E-03
MSMEG_1470	50S ribosomal protein L6		0.64	3.39E-04	0.91	1.57E-01	0.68	6.20E-03
MSMEG_1471	ribosomal protein L18	*rplR*	0.55	4.08E-05	0.79	2.82E-02	0.78	1.34E-02
MSMEG_1472	ribosomal protein S5	*rpsE*	0.57	3.28E-05	0.89	1.95E-01	0.64	3.86E-04
MSMEG_1473	ribosomal protein L30	*rpmD*	0.54	7.73E-05	0.87	8.87E-02	0.75	1.75E-02
MSMEG_1474	ribosomal protein L15	*rplO*	0.64	4.51E-04	0.88	1.83E-01	0.69	4.19E-02
MSMEG_1475	conserved hypothetical protein		1.36	1.51E-02	0.76	8.30E-03	1.25	1.87E-01
MSMEG_1476	signal peptide peptidase SppA, 67K type	*sppA*	0.65	1.30E-04	1.03	5.52E-01	0.94	6.72E-01
MSMEG_1477	major facilitator superfamily protein		0.47	2.09E-02	1.06	6.79E-01	0.84	3.30E-01
MSMEG_1478	transporter, LysE family protein		0.59	6.79E-03	0.92	1.61E-01	1.08	6.00E-01
MSMEG_1479	methyltransferase, putative, family protein		0.77	6.66E-02	0.79	1.86E-02	0.88	5.19E-01
MSMEG_1480	methyltransferase		0.75	5.23E-02	0.82	8.03E-03	1.40	2.96E-01
MSMEG_1481	methyltransferase		1.00	9.82E-01	1.01	8.72E-01	1.27	4.15E-01
MSMEG_1482	methyltransferase		1.06	5.85E-01	0.80	3.96E-02	0.81	2.06E-01
MSMEG_1483	preprotein translocase, SecY subunit		0.43	1.95E-05	0.85	9.02E-02	1.46	8.34E-02
MSMEG_1484	adenylate kinase		0.51	1.55E-05	0.76	2.31E-02	1.30	2.08E-02
MSMEG_1485	methionine aminopeptidase, type I	*map*	0.45	4.69E-08	0.67	5.10E-02	1.14	4.87E-01
MSMEG_1486	RNA polymerase sigma-70 factor		4.40	5.69E-05	0.97	2.76E-01	0.59	4.55E-03
MSMEG_1487	conserved hypothetical protein		3.91	9.01E-05	0.91	2.74E-01	0.59	2.11E-02
MSMEG_1488	acetolactate synthase		1.49	7.06E-02	0.95	6.92E-01	0.86	2.44E-01
MSMEG_1489	conserved hypothetical protein		1.98	7.13E-02	0.95	6.56E-01	1.80	4.19E-01
MSMEG_1490	3-oxoacyl-ACP synthase III		1.99	9.04E-02	1.03	7.34E-01	1.06	1.77E-01
MSMEG_1491	histidinol-phosphate aminotransferase 2		1.69	1.65E-01	0.87	2.10E-01	0.94	6.75E-01
MSMEG_1492	transcriptional regulator, MarR family protein		1.09	3.46E-01	0.82	4.99E-02	0.82	2.23E-01
MSMEG_1493	sensor histidine kinase		1.81	2.89E-02	1.20	1.71E-02	0.83	1.65E-01
MSMEG_1493	sensor histidine kinase		1.36	3.07E-02	0.98	5.23E-01	1.35	2.22E-01
MSMEG_1494	transcriptional regulatory protein DegU		1.54	6.81E-02	0.89	1.06E-01	1.46	3.67E-01
MSMEG_1495	HAD-superfamily protein hydrolase subfamily protein IA, variant 3, putative		24.55	4.69E-03	0.91	5.38E-01	0.20	1.53E-04

MSMEG_1496	3-hydroxyisobutyrate dehydrogenase	*mmsB*	1.76	6.72E-04	1.17	5.90E-02	0.63	5.39E-02
MSMEG_1497	acyl-CoA dehydrogenase family protein member		3.24	5.17E-03	0.84	2.42E-01	0.37	5.84E-04
MSMEG_1498	methylmalonate-semialdehyde dehydrogenase	*mmsA*	3.28	1.88E-04	0.87	9.87E-02	0.33	2.17E-04
MSMEG_1499	oxygenase		0.90	3.90E-01	1.18	5.24E-01	0.88	2.96E-01
MSMEG_1500	TetR family protein transcriptional regulatory protein		1.48	2.61E-01	0.87	1.76E-01	1.11	2.13E-01
MSMEG_1501	methyltransferase, putative, family protein		6.44	8.27E-03	1.42	2.60E-01	0.86	4.69E-01
MSMEG_1502	ABC transporter, ATP-binding protein		5.14	8.09E-03	1.22	4.72E-01	1.03	8.38E-01
MSMEG_1503	probable antibiotic-transport integral membrane leucine and valine rich protein abc transporter		3.80	9.21E-03	1.11	6.61E-01	1.23	4.67E-01
MSMEG_1504	probable antibiotic-transport integral membrane leucine and alanine and valine rich protein abc transporter		4.00	6.40E-03	1.08	8.22E-01	1.08	8.49E-01
MSMEG_1505	integral membrane protein		0.99	9.81E-01	1.47	1.72E-01	1.00	7.99E-01
MSMEG_1506	transcriptional regulator, TetR family protein		1.02	8.18E-01	1.19	2.41E-01	1.24	5.77E-02
MSMEG_1507	D-aminopeptidase		0.89	2.68E-01	0.99	9.42E-01	1.04	2.48E-01
MSMEG_1508	amino acid permease-associated region		1.20	3.49E-02	0.95	7.86E-01	1.08	6.48E-01
MSMEG_1509	TetR-family protein transcriptional regulator		1.08	7.00E-01	2.56	1.81E-01	1.26	4.96E-01
MSMEG_1510	dTDP-4-dehydrorhamnose 3,5-epimerase		0.61	2.42E-03	1.35	1.77E-01	0.87	2.90E-01
MSMEG_1511	putative oxidoreductase		0.96	8.88E-01	1.28	1.16E-01	1.45	2.91E-01
MSMEG_1512	dTDP-glucose 4,6-dehydratase	*rfbB*	0.84	6.03E-02	1.39	9.50E-02	0.67	1.23E-02
MSMEG_1513	conserved hypothetical protein		1.24	9.96E-03	1.00	9.91E-01	1.27	9.85E-02
MSMEG_1514	conserved hypothetical protein		1.36	3.03E-02	1.25	3.34E-01	1.35	5.93E-02
MSMEG_1515	two-component sensor histidine kinase		1.63	1.04E-02	1.63	6.35E-02	1.37	5.31E-04
MSMEG_1516	thioredoxin reductase		1.28	4.95E-02	1.26	5.34E-02	1.99	3.86E-04
MSMEG_1517	spfh domain/band 7 family protein, putative		1.03	7.87E-01	1.13	3.07E-01	0.39	7.05E-03
MSMEG_1518	hypothetical protein		0.86	7.43E-03	0.91	8.01E-02	0.49	6.48E-04
MSMEG_1519	translation initiation factor IF-1	*infA*	0.70	1.54E-03	0.96	3.99E-01	2.21	7.58E-03
MSMEG_1520	ribosomal protein L36	*rpmJ*	0.50	1.67E-03	1.02	7.67E-01	2.07	8.05E-02
MSMEG_1521	ribosomal protein S13p/S18e	*rpsM*	0.84	2.85E-02	0.85	2.52E-02	0.73	1.48E-02
MSMEG_1522	ribosomal protein S11	*rpsK*	0.94	2.98E-01	0.78	4.37E-04	1.31	3.18E-02
MSMEG_1523	ribosomal protein S4	*rpsD*	0.84	1.57E-01	0.78	2.50E-02	0.78	2.15E-02
MSMEG_1524	DNA-directed RNA polymerase, alpha subunit	*rpoA*	0.56	2.71E-05	0.94	4.07E-01	1.81	6.60E-03
MSMEG_1525	50S ribosomal protein L17		0.33	8.68E-07	1.16	2.37E-01	1.47	4.70E-02
MSMEG_1526	probable cutinase		1.52	7.02E-04	1.04	6.52E-01	1.06	7.30E-01
MSMEG_1527	tRNA pseudouridine synthase A	*truA*	0.89	5.21E-01	1.02	7.93E-01	1.43	2.52E-01
MSMEG_1528	cutinase		1.13	1.74E-01	0.86	2.79E-02	1.06	8.39E-01
MSMEG_1529	serine esterase, cutinase family protein		0.86	4.62E-02	0.84	5.23E-02	0.94	5.38E-01

MSMEG_1530	integral membrane protein		0.73	6.90E-03	1.42	6.30E-02	2.61	1.68E-01
MSMEG_1531	conserved hypothetical protein		0.66	1.52E-02	1.02	7.36E-01	1.45	1.13E-02
MSMEG_1532	antibiotic biosynthesis monooxygenase		1.05	6.07E-01	0.89	4.14E-01	1.61	1.27E-01
MSMEG_1533	subtilase family protein		2.56	2.44E-01	1.05	7.83E-01	0.87	3.28E-01
MSMEG_1534	probable conserved membrane protein		1.07	5.33E-01	1.57	2.63E-01	0.88	2.47E-01
MSMEG_1535	conserved hypothetical protein		1.26	3.74E-01	1.16	2.27E-01	1.05	6.04E-01
MSMEG_1536	ftsk/spoiiie family protein		1.24	1.65E-01	0.89	3.83E-01	1.05	6.92E-01
MSMEG_1537	conserved hypothetical protein		1.39	2.45E-01	1.03	8.57E-01	0.77	2.78E-02
MSMEG_1538	conserved hypothetical protein		1.65	9.98E-02	1.04	8.14E-01	0.87	3.69E-01
MSMEG_1539	conserved hypothetical protein		2.12	1.03E-01	1.05	5.56E-01	0.66	9.36E-02
MSMEG_1540	ATP-dependent RNA helicase		0.55	4.20E-03	1.15	2.12E-01	1.74	1.25E-01
MSMEG_1541	hypothetical protein		1.45	1.54E-01	1.17	3.94E-01	1.18	4.28E-01
MSMEG_1542	transcriptional regulator		2.25	7.35E-04	0.74	3.14E-02	0.97	8.77E-01
MSMEG_1543	eptc-inducible aldehyde dehydrogenase		2.43	2.65E-03	0.84	2.45E-01	0.10	1.66E-06
MSMEG_1544	PduO protein		1.56	2.47E-02	0.82	2.58E-01	0.71	8.13E-02
MSMEG_1545	conserved hypothetical protein		1.21	2.16E-01	0.91	3.50E-01	1.34	2.27E-01
MSMEG_1546	coenzyme B12-dependent glycerol dehydrogenase small subunit		3.09	2.60E-03	0.76	1.72E-02	0.21	8.04E-05
MSMEG_1547	glycerol dehydratase large subunit		2.74	2.11E-03	0.68	1.82E-02	0.25	3.12E-05
MSMEG_1548	propanediol utilization: dehydratase, medium subunit		2.11	1.27E-03	0.73	1.34E-02	0.28	8.01E-05
MSMEG_1549	glycerol dehydratase reactivation factor large subunit; this gene contains a frame shift which is not the result of sequencing error		1.14	3.72E-01	0.86	6.51E-02	0.86	3.82E-01
MSMEG_1550	PduH protein		0.70	1.64E-02	1.00	9.69E-01	0.91	1.36E-01
MSMEG_1551	hypothetical protein		1.42	4.27E-01	0.60	1.84E-02	1.23	1.76E-01
MSMEG_1552	ethanolamine permease	*eat*	1.98	4.98E-04	0.80	3.11E-02	0.71	1.74E-01
MSMEG_1553	ethanolamine ammonia-lyase, large subunit	*eutB*	2.09	1.67E-04	0.97	7.92E-01	1.45	1.98E-01
MSMEG_1554	ethanolamine ammonia-lyase, light chain	*eutC*	1.99	4.25E-03	1.04	7.99E-01	1.50	1.42E-01
MSMEG_1555	conserved hypothetical protein, authentic frameshift; this gene contains a frame shift which is not the result of sequencing error; identified by similarity to RF:NP_854757.1		1.10	3.61E-01	1.13	2.25E-01	0.99	9.44E-01
MSMEG_1556	ribosomal protein L13	*rplM*	0.52	1.44E-04	1.06	5.95E-01	0.88	1.10E-01
MSMEG_1557	ribosomal protein S9	*rpsI*	0.82	4.59E-03	0.96	6.99E-01	0.67	1.59E-02
MSMEG_1558	conserved hypothetical protein		1.99	1.09E-02	0.98	9.18E-01	0.49	1.56E-02
MSMEG_1559	phosphoglucosamine mutase	*glmM*	1.20	5.77E-03	0.77	3.42E-02	1.11	5.23E-02
MSMEG_1560	conserved hypothetical protein		0.97	8.57E-01	0.98	8.66E-01	0.97	6.20E-01

MSMEG_1561	LysR-family protein trancsriptional regulator		2.01	1.54E-02	0.81	7.91E-02	1.24	2.57E-01
MSMEG_1562	conserved hypothetical alanine and proline rich protein; this gene contains a frame shift which is not the result of sequencing error		1.46	1.86E-01	0.87	1.24E-01	0.83	3.79E-02
MSMEG_1562	conserved hypothetical alanine and proline rich protein; this gene contains a frame shift which is not the result of sequencing error		0.86	2.84E-01	1.12	4.74E-01	1.20	3.45E-01
MSMEG_1563	short-chain dehydrogenase/reductase SDR		2.08	5.02E-02	0.86	3.04E-01	1.24	1.51E-01
MSMEG_1564	lignostilbene-alpha,beta-dioxygenase		1.53	3.45E-02	1.66	1.52E-01	0.92	2.42E-01
MSMEG_1565	transcriptional regulator, TetR family protein		1.04	5.51E-01	1.03	7.32E-01	0.90	7.05E-01
MSMEG_1566	oxidoreductase		1.81	3.72E-03	1.08	3.74E-01	2.33	9.49E-02
MSMEG_1567	conserved hypothetical protein		0.53	3.33E-04	0.95	2.33E-01	1.05	8.23E-01
MSMEG_1568	glucosamine--fructose-6-phosphate aminotransferase, isomerizing	*glmS*	0.66	1.89E-03	0.84	5.64E-02	1.76	3.21E-02
MSMEG_1569	hypothetical protein		0.62	5.06E-04	1.22	1.10E-01	0.91	6.43E-01
MSMEG_1570	carboxylesterase		1.34	5.08E-02	0.76	1.24E-01	1.42	3.71E-01
MSMEG_1571	integral membrane protein		1.23	1.50E-01	1.11	2.01E-01	1.01	8.53E-01
MSMEG_1572	transcriptional regulator, GntR family protein		1.09	4.88E-01	1.13	6.16E-01	0.98	7.86E-01
MSMEG_1573	carbohydrate kinase family protein		1.27	7.87E-02	0.77	7.30E-03	2.58	1.69E-02
MSMEG_1574	glutamate decarboxylase		0.84	5.83E-02	0.78	8.92E-03	0.96	5.45E-01
MSMEG_1575	alanine racemase	*alr*	0.65	3.61E-03	1.02	8.25E-01	3.55	1.05E-03
MSMEG_1576	alpha/beta hydrolase fold		0.67	1.73E-03	0.94	4.61E-01	3.88	1.44E-01
MSMEG_1577	conserved hypothetical protein		0.52	3.50E-03	0.93	4.16E-01	2.96	2.93E-02
MSMEG_1578	peptidase M22, glycoprotease		0.72	3.55E-02	0.96	7.57E-01	3.21	3.29E-02
MSMEG_1579	ribosomal-protein-alanine acetyltransferase	*rimI*	0.70	8.53E-04	1.10	2.00E-01	2.21	9.98E-03
MSMEG_1580	O-sialoglycoprotein endopeptidase		0.63	1.38E-03	0.96	7.36E-01	2.70	5.94E-03
MSMEG_1581	conserved hypothetical protein		0.44	4.05E-04	1.57	1.06E-01	1.89	5.37E-02
MSMEG_1582	chaperonin GroS	*groS*	0.95	2.47E-01	0.60	2.92E-03	0.45	1.04E-04
MSMEG_1583	chaperonin GroL	*groL*	0.77	1.01E-01	0.74	3.36E-02	0.49	1.17E-02
MSMEG_1584	hypothetical protein		0.49	3.18E-04	0.93	3.99E-01	0.79	1.66E-02
MSMEG_1585	cupin domain protein		1.81	1.33E-01	1.08	4.17E-01	0.85	2.93E-01
MSMEG_1586	beta-lactamase		1.89	1.81E-01	1.60	2.25E-01	0.98	4.00E-01
MSMEG_1587	alpha/beta hydrolase fold		1.14	3.90E-02	1.54	1.19E-01	1.31	2.59E-01
MSMEG_1588	conserved hypothetical protein		2.32	2.96E-03	1.47	9.83E-02	0.68	1.16E-01
MSMEG_1589	hypothetical protein		0.94	4.58E-01	2.42	1.50E-01	1.31	4.14E-01
MSMEG_1590	hypothetical protein		1.08	2.93E-01	0.97	7.50E-01	1.08	1.89E-01
MSMEG_1591	TnpC protein		1.94	1.19E-03	0.76	2.48E-02	1.59	1.34E-01
MSMEG_1591	TnpC protein		1.45	3.46E-02	0.83	7.33E-02	1.26	2.06E-01

MSMEG_1592	hypothetical protein		1.71	6.49E-03	1.15	2.39E-01	0.53	1.00E-03
MSMEG_1593	hypothetical protein		1.98	3.92E-01	1.99	1.06E-01	0.90	2.98E-01
MSMEG_1594	enoyl-CoA hydratase		2.05	1.89E-04	0.96	5.29E-01	0.88	3.56E-01
MSMEG_1595	putative oxidoreductase		1.32	3.31E-01	2.83	6.10E-02	1.00	8.61E-01
MSMEG_1596	transcriptional regulator		0.95	5.66E-01	0.92	5.52E-01	0.90	2.31E-01
MSMEG_1597	Transcription factor WhiB		0.59	5.53E-02	1.22	4.03E-01	1.89	4.15E-01
MSMEG_1598	conserved hypothetical protein		0.42	5.63E-03	1.09	5.40E-01	1.20	2.34E-01
MSMEG_1599	RNA polymerase sigma-70 factor		1.63	1.14E-03	0.80	4.89E-02	2.42	1.58E-02
MSMEG_1600	conserved hypothetical protein		1.03	6.31E-01	0.78	3.21E-02	1.69	1.05E-01
MSMEG_1601	conserved hypothetical protein		1.72	6.27E-03	1.18	2.04E-01	1.32	8.46E-03
MSMEG_1602	inosine-5'-monophosphate dehydrogenase	*guaB*	1.33	5.99E-04	0.89	1.74E-01	1.24	5.90E-02
MSMEG_1603	IMP dehydrogenase family protein		0.83	1.09E-01	0.73	9.98E-03	1.47	7.92E-03
MSMEG_1604	FAD dependent oxidoreductase		1.00	9.87E-01	1.36	4.25E-02	2.12	1.20E-02
MSMEG_1605	phosphate transport system regulatory protein PhoU	*phoU*	1.40	3.98E-01	2.14	2.59E-03	0.67	1.35E-01
MSMEG_1606	benzoylformate decarboxylase		1.78	3.68E-01	4.84	3.82E-01	1.38	1.14E-01
MSMEG_1607	putative tautomerase		0.58	1.28E-02	1.01	6.05E-01	1.15	2.12E-01
MSMEG_1608	glycosyl transferase		1.03	7.99E-01	1.70	3.88E-02	1.05	4.78E-01
MSMEG_1609	beta-phosphoglucomutase hydrolase; this gene contains a frame shift which is not the result of sequencing error; identified by match to protein family HMM PF00702; match to protein family HMM TIGR01509; match to protein family HMM TIGR02009		1.44	2.86E-02	1.61	8.88E-02	0.86	2.95E-01
MSMEG_1610	glutamine-hydrolyzing GMP synthase		0.64	4.17E-05	0.73	2.49E-02	2.09	6.39E-03
MSMEG_1611	transcriptional regulator, TetR family protein, putative		0.33	3.87E-04	1.02	6.55E-01	2.60	5.86E-02
MSMEG_1612	extracellular solute-binding protein, family protein 3		0.72	8.63E-02	1.30	3.25E-01	1.06	7.12E-01
MSMEG_1613	ABC polar amino acid transporter, inner membrane subunit		1.22	4.35E-01	0.81	4.15E-02	0.97	5.14E-01
MSMEG_1614	glutamine transport ATP-binding protein GlnQ		0.98	7.75E-01	0.85	3.51E-01	1.15	3.17E-01
MSMEG_1615	gamma-glutamyltranspeptidase	*ggt*	1.92	3.60E-01	1.15	6.51E-01	1.25	3.89E-01
MSMEG_1616	hypothetical protein; this gene contains a frame shift which is not the result of sequencing error; identified by Glimmer2; putative		1.06	5.81E-01	2.13	1.75E-01	0.86	1.80E-01
MSMEG_1617	kumamolisin		1.20	1.98E-01	1.04	7.89E-01	0.21	4.36E-07
MSMEG_1618	hypothetical protein		0.93	3.82E-01	0.95	5.53E-01	0.28	2.17E-05

MSMEG_1619	hypothetical protein	2.18	3.87E-01	2.66	7.18E-02	1.07	5.37E-01
MSMEG_1620	conserved hypothetical protein	1.90	1.65E-01	2.63	1.34E-01	1.40	2.40E-01
MSMEG_1621	pyrimidine-specific ribonucleoside hydrolase RihA	0.93	2.38E-01	0.93	1.34E-01	1.44	6.55E-02
MSMEG_1622	putative DNA repair polymerase	1.95	1.42E-01	1.67	1.56E-01	2.25	2.69E-01
MSMEG_1623	short chain dehydrogenase	2.09	3.12E-03	0.90	2.98E-01	1.03	8.04E-01
MSMEG_1624	universal stress protein family protein	3.56	3.53E-02	1.50	2.46E-02	0.40	1.58E-02
MSMEG_1625	amino acid transporter, putative	3.21	6.93E-02	1.34	1.47E-01	0.73	1.19E-01
MSMEG_1626	putative DNA-binding protein	2.26	4.29E-02	0.98	8.22E-01	0.82	3.07E-01
MSMEG_1627	hypothetical protein	0.93	4.45E-01	0.80	3.31E-02	1.61	2.03E-01
MSMEG_1628	oxidoreductase, short chain dehydrogenase/reductase family protein	1.07	7.48E-01	1.28	2.46E-01	1.01	9.78E-01
MSMEG_1629	hypothetical protein	0.83	3.16E-03	0.76	1.10E-01	0.43	5.21E-03
MSMEG_1630	2,5-dichloro-2,5-cyclohexadiene-1,4-diol dehydrogenase	1.39	3.28E-01	1.14	4.98E-01	1.05	5.52E-01
MSMEG_1631	DNA-binding protein	1.44	2.21E-03	1.04	7.86E-01	1.16	7.30E-02
MSMEG_1632	hypothetical protein	0.65	7.16E-03	0.89	3.21E-01	1.04	1.48E-01
MSMEG_1633	DNA polymerase III, alpha subunit, putative	0.95	6.90E-01	1.77	3.18E-01	1.15	3.72E-01
MSMEG_1634	probable forkhead-associated protein	0.34	8.61E-06	0.88	3.83E-01	0.74	3.10E-01
MSMEG_1635	nitroreductase family protein	0.55	6.82E-06	0.97	3.11E-01	0.77	1.85E-01
MSMEG_1636	RNA methyltransferase, TrmH family protein, group 2	0.58	2.17E-03	0.98	7.02E-01	1.27	2.11E-01
MSMEG_1637	periplasmic sensor signal transduction histidine kinase	2.70	1.31E-05	0.79	3.53E-02	0.36	7.37E-04
MSMEG_1638	Roadblock/LC7 domain protein	1.90	6.95E-04	0.67	2.21E-02	0.49	6.96E-04
MSMEG_1639	conserved hypothetical protein	1.17	1.36E-01	0.82	1.11E-01	0.56	3.82E-03
MSMEG_1640	ATP/GTP-binding protein	1.08	2.38E-01	0.71	1.40E-02	0.70	1.61E-02
MSMEG_1641	conserved hypothetical protein	0.30	4.06E-07	0.84	2.02E-01	1.08	6.50E-01
MSMEG_1642	ABC transporter, ATP-binding protein	0.58	2.31E-05	1.08	2.98E-01	1.73	1.55E-01
MSMEG_1643	hypothetical protein	0.37	6.11E-05	0.90	2.00E-01	1.62	1.72E-01
MSMEG_1644	hypothetical protein	0.75	7.61E-03	0.99	8.85E-01	0.90	3.35E-01
MSMEG_1645	NADH:flavin oxidoreductase/nadh oxidase	0.80	8.40E-02	0.72	3.51E-02	1.04	6.23E-01
MSMEG_1646	ribosomal RNA adenine dimethylase family protein	1.02	9.13E-01	1.24	1.15E-01	0.99	9.04E-01
MSMEG_1647	tetrahydrofolate dehydrogenase/cyclohydrolase FolD	0.86	4.24E-02	0.85	8.35E-02	1.13	2.44E-01
MSMEG_1648	putative transcriptional regulator	1.31	3.31E-01	1.82	9.30E-02	0.90	3.09E-01
MSMEG_1649	FAD dependent oxidoreductase	1.06	6.02E-01	1.14	3.60E-01	0.87	3.48E-01

MSMEG_1650	methyltransferase type 11		0.73	7.95E-04	0.87	4.81E-01	0.87	3.58E-01
MSMEG_1651	homoserine O-acetyltransferase	*metX*	0.97	5.62E-01	0.80	2.67E-01	0.76	1.08E-01
MSMEG_1652	O-acetylhomoserine sulfhydrylase		0.76	1.20E-02	0.70	7.80E-02	0.69	1.94E-02
MSMEG_1653	hypothetical protein		0.98	7.42E-01	1.17	2.89E-01	1.19	7.30E-02
MSMEG_1654	isocitrate dehydrogenase, NADP-dependent		0.39	1.08E-05	1.05	6.76E-01	1.58	5.96E-02
MSMEG_1655	hydrolase, alpha/beta fold family protein, putative		2.78	6.46E-04	0.82	9.43E-02	0.77	1.78E-01
MSMEG_1656	exodeoxyribonuclease III	*xth*	0.45	3.49E-03	1.07	6.48E-01	1.65	2.60E-01
MSMEG_1657	tryptophanyl-tRNA synthetase	*trpS*	0.51	4.90E-04	0.87	6.97E-02	1.60	1.47E-01
MSMEG_1658	ribonuclease, putative		0.55	4.08E-05	1.18	3.57E-01	0.98	8.68E-01
MSMEG_1659	multidrug resistance protein, SMR family protein		0.67	4.67E-03	0.95	6.10E-01	1.38	1.56E-02
MSMEG_1660	oxidoreductase, 2-nitropropane dioxygenase family protein		0.56	7.31E-03	1.16	1.66E-01	3.98	3.20E-01
MSMEG_1661	D-alanyl-D-alanine carboxypeptidase		1.28	1.21E-01	0.96	7.91E-01	1.36	4.36E-01
MSMEG_1662	taurine-pyruvate aminotransferase		1.18	1.13E-01	0.71	2.41E-02	0.44	1.95E-04
MSMEG_1663	transcriptional regulator, AsnC family protein		1.46	2.64E-02	0.87	2.22E-01	0.34	5.40E-05
MSMEG_1664	hypothetical protein		1.58	1.46E-02	0.73	4.29E-02	0.80	1.92E-01
MSMEG_1665	aldehyde dehydrogenase (NAD) family protein		0.99	8.76E-01	0.72	8.14E-03	0.75	6.55E-02
MSMEG_1666	RNA polymerase sigma-70 factor		2.00	7.04E-04	0.89	2.01E-01	1.62	1.21E-03
MSMEG_1667	IS110 family transposase, truncation; identified by similarity to OMNI:MT3430		1.42	7.80E-02	0.77	8.87E-03	0.86	2.97E-01
MSMEG_1668	pyridoxamine 5'-phosphate oxidase family		1.79	3.94E-02	1.04	6.79E-01	1.00	9.89E-01
MSMEG_1669	succinate dehydrogenase, iron-sulfur protein	*sdhB*	0.27	3.89E-07	0.86	1.13E-01	2.46	1.20E-01
MSMEG_1670	succinate dehydrogenase, flavoprotein subunit	*sdhA*	0.31	1.06E-05	1.05	5.74E-01	2.02	2.10E-02
MSMEG_1671	succinate dehydrogenase hydrophobic membrane anchor protein SdhD		0.39	2.83E-06	0.92	1.41E-01	2.09	3.75E-02
MSMEG_1672	succinate dehydrogenase, cytochrome b556 subunit	*sdhC*	0.39	1.55E-05	0.87	6.73E-02	1.79	6.51E-02
MSMEG_1673	cytidine deaminase	*cdd*	0.63	2.45E-03	0.92	6.17E-01	1.70	1.95E-01
MSMEG_1674	hypothetical protein		1.10	5.40E-01	0.88	2.00E-01	1.62	2.68E-01
MSMEG_1675	pyrimidine-nucleoside phosphorylase		0.85	5.58E-03	0.89	3.73E-01	2.48	4.53E-03
MSMEG_1676	adenosine deaminase	*add*	1.14	4.32E-01	0.76	2.51E-01	0.96	4.45E-01
MSMEG_1677	aspartate ammonia-lyase	*aspA*	2.32	2.89E-03	1.11	5.13E-01	0.12	6.83E-06
MSMEG_1678	transcriptional regulator, LysR family protein		1.13	2.35E-01	0.92	2.12E-01	1.20	1.74E-01
MSMEG_1679	AmiB		2.50	9.51E-04	1.61	9.49E-03	0.16	9.11E-05
MSMEG_1680	conserved hypothetical protein		1.49	1.06E-02	1.04	6.27E-01	0.14	1.95E-05
MSMEG_1681	endoribonuclease L-PSP superfamily protein		2.31	1.55E-03	1.01	9.57E-01	0.41	2.62E-02
MSMEG_1682	flavin-containing monooxygenase FMO		2.43	4.68E-04	1.06	5.56E-01	0.16	4.13E-05

MSMEG_1683	cytosine/purine/uracil/thiamine/allantoin permease family protein		2.45	1.28E-04	1.23	1.14E-01	0.14	1.76E-08
MSMEG_1684	conserved hypothetical protein		0.40	5.92E-07	1.15	4.32E-02	0.73	6.65E-02
MSMEG_1685	hypothetical protein		1.18	4.25E-01	0.98	7.59E-01	1.15	3.37E-01
MSMEG_1686	NPL/P60-family protein secreted protein		1.08	3.37E-01	1.02	7.74E-01	1.14	5.06E-01
MSMEG_1687	hypothetical protein		1.12	6.75E-02	1.28	2.34E-01	0.87	1.84E-01
MSMEG_1688	cupin domain protein		1.62	4.24E-02	1.02	8.01E-01	1.17	5.10E-01
MSMEG_1689	3-oxoacyl-[acyl-carrier-protein] reductase		1.04	4.72E-01	1.05	5.42E-01	1.33	8.98E-02
MSMEG_1690	putative ECF sigma factor RpoE1		1.28	5.51E-02	1.03	4.45E-01	1.47	3.84E-01
MSMEG_1691	transcriptional regulatory protein		1.20	1.03E-01	1.06	6.27E-01	1.16	3.48E-01
MSMEG_1692	ECF-family protein RNA polymerase sigma		1.13	3.19E-01	1.11	4.99E-02	1.02	8.73E-01
MSMEG_1693	succinate dehydrogenase [ubiquinone] flavoprotein subunit		1.28	7.19E-02	1.32	1.36E-01	1.02	7.89E-01
MSMEG_1694	uracil phosphoribosyltransferase	*upp*	0.64	5.73E-03	1.05	6.01E-01	0.85	1.26E-01
MSMEG_1695	phosphoglucomutase/phosphomannomutase		1.28	1.87E-01	1.10	3.14E-01	0.85	1.61E-01
MSMEG_1696	regulatory protein, MarR		0.90	5.08E-01	1.90	2.65E-01	0.97	4.75E-01
MSMEG_1697	hypothetical protein		1.41	3.43E-01	1.42	1.23E-01	1.03	1.59E-01
MSMEG_1698	putative ammonia monooxygenase superfamily protein		0.69	3.97E-02	18.97	2.00E-01	1.85	4.15E-01
MSMEG_1699	pentachlorophenol 4-monooxygenase		1.43	1.88E-01	1.66	1.70E-01	1.06	7.46E-01
MSMEG_1700	TetR-family protein transcriptional regulator		0.84	1.44E-01	5.09	1.35E-01	1.02	9.06E-01
MSMEG_1701	purine nucleotide phosphorylase		0.67	1.51E-02	0.82	4.10E-02	0.94	2.90E-01
MSMEG_1702	amidohydrolase		1.67	1.95E-04	1.51	9.89E-02	2.34	9.87E-03
MSMEG_1703	amidohydrolase		1.10	5.40E-01	1.14	5.31E-01	2.13	2.19E-02
MSMEG_1704	ABC transporter		1.07	8.22E-01	1.05	8.13E-01	0.27	2.74E-03
MSMEG_1705	D-xylose transport ATP-binding protein XylG		1.11	7.22E-01	1.20	4.75E-01	0.15	2.90E-06
MSMEG_1706	xylose transport system permease protein XylH		0.74	2.93E-01	1.23	4.90E-01	0.53	8.00E-03
MSMEG_1707	phosphatase YfbT		0.60	1.05E-02	1.07	6.63E-01	0.94	2.97E-01
MSMEG_1708	ribose operon repressor, putative		0.68	4.99E-02	1.06	6.53E-01	1.17	3.40E-01
MSMEG_1709	inner membrane ABC transporter permease protein YjfF		1.03	8.59E-01	0.97	7.87E-01	0.70	1.15E-01
MSMEG_1710	ribose transport system permease protein RbsC		0.62	9.26E-03	1.17	3.87E-01	0.63	5.16E-02
MSMEG_1711	ATP binding protein of ABC transporter		0.80	1.62E-01	0.92	4.61E-01	0.09	8.41E-06
MSMEG_1712	ABC transporter periplasmic-binding protein YtfQ		0.99	9.47E-01	0.86	3.75E-01	0.12	2.86E-05
MSMEG_1713	L-ribulokinase	*araB*	0.40	4.93E-03	1.03	8.68E-01	0.86	1.11E-01
MSMEG_1714	L-ribulose-5-phosphate 4-epimerase UlaF		0.54	2.97E-02	1.16	3.79E-01	0.56	2.02E-02
MSMEG_1715	L-arabinose isomerase	*araA*	0.40	3.71E-03	0.97	8.92E-01	1.04	6.39E-01

MSMEG_1716	IS3 family protein element, transposase orfA	0.22	2.47E-06	1.47	7.11E-02	1.04	5.13E-01
MSMEG_1717	ISMsm8, transposase	0.77	2.32E-01	1.05	7.52E-01	0.94	4.30E-01
MSMEG_1718	This gene is disrupted by an IS element.; IS3 family element, transposase orfB, interruption-N; identified by similarity to GP:15619023	0.71	3.10E-03	1.00	9.93E-01	0.88	3.74E-01
MSMEG_1719	This gene is disrupted by an IS element.; IS3 family element, transposase orfB, interruption-C; identified by similarity to GP:15619023	0.79	3.19E-01	1.62	1.66E-01	1.01	9.65E-01
MSMEG_1721	IS1137, transposase orfB	0.62	2.20E-03	2.07	1.02E-01	1.41	2.88E-01
MSMEG_1722	hypothetical protein	0.75	5.04E-02	1.38	6.75E-03	0.78	1.90E-01
MSMEG_1723	hypothetical protein	0.76	2.69E-02	1.09	4.89E-01	1.41	3.49E-01
MSMEG_1724	conserved hypothetical protein	1.42	8.65E-03	1.23	1.45E-01	0.39	1.70E-04
MSMEG_1729	This gene is disrupted by an ISMsm1 element.; ISMsm5, transposase, interruption-N; identified by similarity to GB:CAD18826.1	0.95	1.15E-01	0.92	2.79E-01	0.48	2.21E-05
MSMEG_1731	IS6120, transposase	0.88	8.71E-03	0.77	1.99E-02	1.52	2.23E-01
MSMEG_1731	IS6120, transposase	1.26	3.27E-02	0.88	1.03E-01	13.61	3.47E-01
MSMEG_1731	IS6120, transposase	0.76	3.77E-02	1.11	1.42E-01	1.01	9.21E-01
MSMEG_1732	Transposase IS116/IS110/IS902 family protein	1.18	5.21E-01	1.50	3.43E-01	1.01	9.39E-01
MSMEG_1733	conserved hypothetical protein	1.00	9.86E-01	1.13	2.72E-01	1.17	4.51E-01
MSMEG_1734	conserved hypothetical protein	0.53	5.36E-04	1.11	9.54E-02	0.72	5.87E-03
MSMEG_1735	dihydrolipoamide dehydrogenase	0.51	1.92E-04	0.97	8.20E-01	0.87	3.16E-01
MSMEG_1736	glycerol-3-phosphate dehydrogenase 2	1.16	1.01E-01	0.97	5.83E-01	1.11	5.50E-01
MSMEG_1737	RNA pseudouridine synthase family protein	0.43	2.99E-04	1.05	5.81E-01	1.24	7.15E-02
MSMEG_1738	probable conserved transmembrane protein	3.16	1.73E-01	0.52	1.33E-02	28.81	1.10E-01
MSMEG_1739	enoyl-CoA hydratase/isomerase family protein	0.47	2.15E-06	1.16	3.32E-01	1.41	1.64E-01
MSMEG_1740	dehydrogenase/reductase SDR family protein member 4	0.71	4.63E-02	0.94	4.97E-01	0.92	8.06E-01
MSMEG_1741	TetR-family protein transcriptional regulator	0.51	1.04E-04	1.40	1.54E-01	1.05	6.49E-01
MSMEG_1742	oxidoreductase	0.53	1.86E-02	0.95	6.73E-01	1.28	3.70E-01
MSMEG_1743	Fatty acid desaturase	0.45	3.81E-03	1.12	1.72E-01	0.98	3.71E-01
MSMEG_1744	hypothetical protein	0.73	4.28E-02	1.89	2.82E-01	1.40	3.65E-01
MSMEG_1745	transcriptional regulator	0.81	5.89E-02	1.22	1.96E-01	1.07	1.73E-01
MSMEG_1746	6-hydroxy-D-nicotine oxidase	0.98	8.95E-01	1.16	1.53E-01	3.79	3.64E-01
MSMEG_1747	RNA polymerase sigma-70 factor	1.01	9.73E-01	1.00	9.79E-01	0.98	9.31E-01
MSMEG_1749	putative monooxygenase	4.18	6.31E-03	1.55	2.78E-01	0.68	1.26E-01
MSMEG_1750	hypothetical protein	0.82	3.76E-02	0.91	2.63E-01	1.14	2.95E-01
MSMEG_1750	hypothetical protein	1.15	4.39E-01	1.04	6.77E-01	1.08	5.46E-01

MSMEG_1751	norsolorinic acid reductase		1.05	7.01E-01	1.46	2.08E-01	1.16	2.34E-01
MSMEG_1752	hypothetical protein		1.12	3.93E-01	1.54	1.58E-01	1.08	3.61E-01
MSMEG_1753	MTA/SAH nucleosidase	*mtnN*	0.47	5.43E-04	1.08	4.52E-01	1.31	7.43E-02
MSMEG_1754	conserved hypothetical protein		0.67	3.51E-03	1.14	2.88E-01	1.15	6.45E-01
MSMEG_1755	anti-sigm factor, ChrR		2.91	1.11E-02	0.67	8.57E-03	0.89	9.87E-02
MSMEG_1756	endonuclease VIII and dna n-glycosylase with an ap lyase activity		1.99	2.04E-02	0.94	1.94E-01	1.39	3.11E-01
MSMEG_1757	DEAD/DEAH box helicase		2.20	2.71E-03	1.01	9.56E-01	1.89	2.18E-01
MSMEG_1758	hypothetical protein		1.28	4.94E-01	2.14	3.49E-02	0.59	7.19E-02
MSMEG_1759	hypothetical protein		3.89	1.75E-02	1.76	4.86E-02	0.76	1.58E-01
MSMEG_1760	short-chain dehydrogenase/reductase SDR		1.05	8.22E-01	0.80	1.37E-02	0.93	4.60E-01
MSMEG_1761	integral membrane protein		1.34	1.13E-01	1.11	5.11E-01	0.91	6.83E-01
MSMEG_1762	piperideine-6-carboxylic acid dehydrogenase		0.66	2.92E-02	1.79	1.63E-01	0.80	1.02E-01
MSMEG_1763	leucine-responsive regulatory protein		1.12	2.35E-01	0.92	4.60E-02	0.86	2.74E-01
MSMEG_1764	L-lysine-epsilon aminotransferase		0.64	9.26E-02	2.35	1.15E-01	0.81	2.92E-01
MSMEG_1765	restriction endonuclease family protein		0.67	1.74E-02	0.93	5.86E-01	0.87	1.51E-01
MSMEG_1766	conserved hypothetical protein		1.51	4.65E-01	2.17	4.27E-02	0.54	3.12E-02
MSMEG_1767	conserved hypothetical protein		1.10	6.81E-01	2.27	2.96E-03	0.60	2.70E-02
MSMEG_1768	conserved hypothetical protein		1.02	9.59E-01	3.23	4.39E-02	0.73	3.35E-01
MSMEG_1769	UsfY protein		0.66	1.61E-02	2.14	1.65E-02	0.63	1.13E-01
MSMEG_1770	conserved hypothetical protein		1.59	4.63E-01	3.09	5.73E-02	0.68	7.34E-02
MSMEG_1771	methylase, putative		1.96	3.35E-01	2.81	1.88E-02	0.25	2.01E-04
MSMEG_1772	conserved hypothetical protein		1.32	5.73E-01	1.97	2.72E-02	0.28	1.41E-04
MSMEG_1773	conserved hypothetical protein		2.32	8.22E-02	1.71	1.47E-01	0.27	3.93E-05
MSMEG_1774	conserved hypothetical protein		3.95	1.80E-01	1.90	6.25E-02	0.43	4.62E-04
MSMEG_1775	cytochrome P450 monooxygenase		1.01	9.55E-01	1.67	3.69E-02	0.70	7.53E-02
MSMEG_1776	ribonuclease Z		1.99	1.43E-02	1.08	5.35E-01	1.42	3.54E-01
MSMEG_1777	UsfY protein		1.78	1.67E-01	2.36	2.01E-02	0.27	9.85E-05
MSMEG_1778	conserved hypothetical protein		1.29	6.31E-02	1.58	4.58E-02	0.97	7.75E-01
MSMEG_1779	hypothetical protein		1.15	6.04E-01	1.20	3.88E-02	1.43	3.60E-01
MSMEG_1780	hypothetical protein		1.11	4.24E-01	1.38	1.19E-01	1.11	3.51E-01
MSMEG_1781	hypothetical protein		1.00	9.99E-01	1.30	1.35E-01	1.00	9.68E-01
MSMEG_1782	oxidoreductase, short chain dehydrogenase/reductase family protein		2.19	2.97E-01	2.38	4.87E-02	0.34	3.10E-03
MSMEG_1783	hypothetical protein		1.18	6.80E-01	2.62	3.80E-02	0.58	2.21E-01
MSMEG_1784	type I topoisomerase		2.42	2.36E-01	1.72	1.86E-01	1.09	6.59E-01
MSMEG_1785	hypothetical protein		1.23	1.99E-01	1.44	2.30E-01	1.10	3.12E-01
MSMEG_1786	stas domain, putative		5.30	1.04E-03	1.44	1.05E-01	0.45	1.00E-03

MSMEG_1787	RsbW protein		1.17	3.55E-01	1.36	1.04E-01	0.70	1.04E-01
MSMEG_1788	conserved hypothetical protein		3.02	2.05E-01	3.54	3.01E-02	0.25	4.04E-04
MSMEG_1789	conserved hypothetical protein		3.14	1.65E-01	2.74	4.05E-02	0.20	1.78E-05
MSMEG_1790	conserved hypothetical protein		3.37	1.95E-01	2.95	2.85E-02	0.15	3.14E-07
MSMEG_1791	UsfY protein		5.88	3.75E-03	1.14	6.04E-01	0.60	2.07E-04
MSMEG_1792	conserved hypothetical protein		1.39	3.26E-01	1.40	1.66E-01	0.92	1.27E-01
MSMEG_1793	membrane protein		1.01	8.34E-01	0.95	3.48E-01	0.88	1.74E-01
MSMEG_1794	dehydrogenase		1.37	2.50E-01	1.48	3.91E-02	0.73	1.62E-01
MSMEG_1795	2-deoxy-scyllo-inosamine dehydrogenase		1.39	5.71E-02	0.91	1.34E-01	1.26	3.72E-01
MSMEG_1796	membrane protein		1.39	2.66E-01	0.71	2.56E-03	1.54	3.82E-01
MSMEG_1797	salicylate esterase		1.59	1.13E-01	0.98	8.97E-01	1.11	2.79E-01
MSMEG_1798	major facilitator superfamily protein		1.16	3.63E-01	0.90	5.75E-01	0.93	4.30E-01
MSMEG_1799	transcriptional regulator		1.01	8.07E-01	0.75	4.09E-03	0.82	9.45E-02
MSMEG_1800	hypothetical protein		1.04	4.37E-01	1.07	1.71E-01	1.01	9.17E-01
MSMEG_1801	hypothetical protein		1.20	1.94E-01	1.41	1.89E-01	1.03	6.65E-01
MSMEG_1802	ChaB protein		1.61	2.21E-01	2.04	2.01E-02	0.39	3.51E-04
MSMEG_1803	RsbW protein		1.20	2.79E-02	1.23	1.60E-01	0.51	3.74E-04
MSMEG_1804	RNA polymerase sigma-F factor		0.56	1.08E-04	1.11	6.70E-02	0.77	1.11E-01
MSMEG_1805	conserved hypothetical protein		0.24	1.04E-05	0.68	1.76E-02	3.56	2.85E-02
MSMEG_1806	conserved hypothetical protein		1.05	7.93E-01	1.08	3.47E-01	1.56	9.97E-02
MSMEG_1807	acetyl-/propionyl-coenzyme A carboxylase alpha chain		0.45	4.27E-04	0.76	6.45E-02	2.00	9.12E-02
MSMEG_1808	Fe-S metabolism associated SufE		0.64	5.57E-04	0.89	1.57E-01	1.58	1.18E-03
MSMEG_1809	putative thiosulfate sulfurtransferase		0.94	1.40E-01	1.05	2.67E-01	1.48	4.49E-03
MSMEG_1810	hypothetical protein		2.56	8.17E-03	0.79	2.68E-01	0.17	4.15E-06
MSMEG_1811	septum formation protein Maf	*maf*	0.14	5.88E-08	1.04	8.24E-01	4.21	6.25E-02
MSMEG_1812	conserved hypothetical protein		0.30	4.66E-06	0.79	1.44E-01	3.03	7.00E-02
MSMEG_1813	propionyl-CoA carboxylase beta chain		0.36	2.22E-05	0.92	3.89E-01	2.48	2.51E-02
MSMEG_1814	hypothetical protein		1.03	6.98E-01	0.96	7.38E-01	1.40	4.34E-01
MSMEG_1815	hypothetical protein		0.96	7.75E-01	1.00	9.23E-01	1.04	5.10E-01
MSMEG_1816	hypothetical protein; this region contains one or more premature stops and/or frameshifts which are not the result of sequencing error; identified by Glimmer2; putative		1.32	2.19E-01	0.84	3.01E-01	1.09	5.63E-02
MSMEG_1816	hypothetical protein; this region contains one or more premature stops and/or frameshifts which are not the result of sequencing error; identified by Glimmer2; putative		0.95	6.78E-01	1.24	3.18E-01	0.94	4.66E-01

MSMEG_1817	probable conserved transmembrane protein		0.56	1.48E-05	1.11	1.55E-01	1.19	3.19E-01
MSMEG_1818	Bacterial membrane flanked domain protein		0.61	1.44E-02	1.16	2.16E-02	1.04	1.50E-01
MSMEG_1819	phosphoribosylaminoimidazole carboxylase, ATPase subunit	*purK*	0.63	2.42E-04	0.87	1.89E-02	1.75	4.63E-02
MSMEG_1820	phosphoribosylaminoimidazole carboxylase, catalytic subunit	*purE*	0.65	1.27E-03	0.80	3.72E-02	1.76	1.83E-02
MSMEG_1821	acyl-CoA dehydrogenase		0.98	9.14E-01	1.17	3.32E-01	3.49	9.25E-03
MSMEG_1822	biotin-[acetyl-CoA-carboxylase] ligase		0.53	6.10E-04	1.05	6.16E-01	3.96	3.09E-02
MSMEG_1823	conserved hypothetical protein		0.88	1.56E-01	1.39	8.55E-02	0.99	9.21E-01
MSMEG_1824	transcriptional regulator, LytR family protein		0.67	7.90E-03	1.13	2.52E-01	1.53	7.53E-02
MSMEG_1825	dTDP-4-dehydrorhamnose reductase	*rfbD*	0.67	2.47E-04	1.06	1.16E-01	1.73	9.23E-03
MSMEG_1826	dTDP-RhA:a-D-GlcNAc-diphosphoryl polyprenol, a-3-L-rhamnosyl transferase		0.38	2.67E-05	1.08	3.67E-01	2.08	2.77E-02
MSMEG_1827	hydrolase, nudix family protein, putative		0.54	1.74E-03	1.02	8.48E-01	0.95	4.38E-01
MSMEG_1828	Nucleotidyl transferase; this gene contains a frame shift which is not the result of sequencing error; identified by match to protein family HMM PF00132; match to protein family HMM PF00483		0.29	6.37E-06	0.83	1.49E-01	2.06	6.46E-02
MSMEG_1829	F420-0:gamma-glutamyl ligase		0.76	2.50E-02	0.93	2.20E-01	1.51	3.84E-02
MSMEG_1830	lppg:fo 2-phospho-l-lactate transferase	*cofD*	0.80	4.15E-03	0.94	4.38E-01	1.02	8.43E-01
MSMEG_1831	Transcription factor WhiB		1.74	1.30E-02	0.63	1.39E-02	1.46	3.91E-01
MSMEG_1832	conserved hypothetical protein		0.62	9.02E-04	1.12	2.16E-01	1.12	4.36E-01
MSMEG_1833	conserved hypothetical protein		1.02	9.23E-01	0.86	3.46E-01	0.84	4.22E-01
MSMEG_1834	phosphomannomutase/phosphoglucomutase		0.81	5.31E-02	0.80	6.24E-02	0.68	2.53E-03
MSMEG_1835	TobH protein		1.09	1.11E-03	0.81	6.20E-02	1.33	6.00E-02
MSMEG_1836	mannose-6-phosphate isomerase, class I	*manA*	0.84	1.08E-02	0.77	4.30E-02	1.50	1.17E-01
MSMEG_1837	secreted protein		0.87	3.29E-01	0.98	7.64E-01	0.47	5.51E-03
MSMEG_1838	cationic amino acid transporter		1.49	3.23E-03	1.19	9.55E-03	0.32	3.43E-05
MSMEG_1839	alkane 1-monooxygenase		1.45	1.61E-02	0.86	2.16E-01	0.76	6.35E-02
MSMEG_1840	rubredoxin		1.70	2.90E-02	0.98	8.37E-01	0.89	2.66E-01
MSMEG_1841	rubredoxin		1.60	2.00E-03	0.87	1.67E-01	0.70	1.05E-01
MSMEG_1842	transcriptional regulator, TetR family protein		0.85	1.98E-02	1.06	2.50E-01	1.04	4.66E-01

MSMEG_1843	adenosylhomocysteinase; this gene contains a frame shift which is not the result of sequencing error; identified by match to protein family HMM PF00670; match to protein family HMM PF02254; match to protein family HMM PF02826; match to protein family HMM PF05221; match to protein family HMM TIGR00936	*ahcY*	0.55	7.94E-05	1.12	3.28E-01	1.29	3.32E-01
MSMEG_1843	adenosylhomocysteinase; this gene contains a frame shift which is not the result of sequencing error; identified by match to protein family HMM PF00670; match to protein family HMM PF02254; match to protein family HMM PF02826; match to protein family HMM PF05221; match to protein family HMM TIGR00936	*ahcY*	0.48	9.07E-05	0.95	5.76E-01	1.08	7.32E-01
MSMEG_1844	conserved hypothetical protein		1.16	1.90E-01	1.09	5.51E-01	1.46	4.33E-01
MSMEG_1845	YghA protein		0.79	3.60E-01	0.80	1.68E-01	1.49	4.15E-02
MSMEG_1846	polysulphide reductase, NrfD		1.18	5.55E-01	1.08	3.52E-01	1.15	4.66E-01
MSMEG_1847	4Fe-4S ferredoxin, iron-sulfur binding protein		1.05	5.99E-01	1.46	5.32E-02	1.15	4.03E-01
MSMEG_1848	formate dehydrogenase-O, major subunit; this gene contains a frame shift which is not the result of sequencing error; identified by match to protein family HMM PF00384; match to protein family HMM PF04879		1.16	2.99E-01	0.87	3.27E-02	1.14	2.07E-01
MSMEG_1848	formate dehydrogenase-O, major subunit; this gene contains a frame shift which is not the result of sequencing error; identified by match to protein family HMM PF00384; match to protein family HMM PF04879		1.00	9.70E-01	1.34	3.00E-01	0.89	2.98E-01
MSMEG_1849	selenocysteine-specific translation elongation factor	*selB*	0.74	1.25E-03	1.05	6.78E-01	1.62	6.69E-02
MSMEG_1850	L-seryl-tRNA selenium transferase	*selA*	0.82	1.63E-02	1.12	8.74E-02	1.11	1.62E-02
MSMEG_1852	selenide, water dikinase	*selD*	1.37	3.06E-01	0.75	5.58E-03	0.98	9.34E-01

MSMEG_1853	Na+/H+ antiporter NhaA; this gene contains a frame shift which is not the result of sequencing error; identified by match to protein family HMM PF06965		1.19	2.11E-01	1.22	2.10E-02	0.90	7.90E-02
MSMEG_1853	Na+/H+ antiporter NhaA; this gene contains a frame shift which is not the result of sequencing error; identified by match to protein family HMM PF06965		0.97	6.76E-01	0.91	3.48E-01	1.10	2.58E-01
MSMEG_1854	valosin containing protein-1		0.89	1.44E-01	1.34	1.41E-01	0.72	1.39E-01
MSMEG_1855	tetratricopeptide repeat protein, putative		1.03	7.56E-01	1.04	6.53E-01	0.89	1.32E-01
MSMEG_1856	hypothetical protein		0.84	2.48E-01	1.17	3.32E-01	0.93	5.52E-01
MSMEG_1859	hypothetical protein		0.91	1.10E-01	1.21	9.35E-02	1.11	5.57E-01
MSMEG_1860	hypothetical protein		1.17	3.65E-01	1.32	1.65E-01	1.12	6.68E-02
MSMEG_1861	hypothetical protein		0.39	2.83E-04	1.43	1.77E-01	1.21	2.55E-01
MSMEG_1862	transposase		0.40	7.02E-06	1.21	7.43E-02	1.86	3.51E-01
MSMEG_1862	transposase		0.55	7.83E-03	0.89	2.73E-01	0.98	8.31E-01
MSMEG_1863	putative transposase		0.62	1.26E-04	1.26	1.81E-02	0.76	3.97E-02
MSMEG_1863	putative transposase		0.52	3.33E-04	1.06	5.63E-01	0.93	3.70E-01
MSMEG_1864	transposase		1.12	4.39E-01	1.21	3.94E-01	1.00	6.02E-01
MSMEG_1866	transposase B		0.64	2.43E-02	1.30	4.44E-01	1.11	3.60E-01
MSMEG_1867	transposase		0.59	8.06E-04	0.98	5.69E-01	0.79	5.03E-02
MSMEG_1868	hypothetical protein		0.58	9.96E-03	1.08	4.36E-01	0.38	1.51E-04
MSMEG_1869	hypothetical protein		0.82	1.56E-01	1.13	2.67E-01	0.72	1.52E-01
MSMEG_1870	conserved hypothetical protein		0.78	2.63E-01	1.21	4.02E-01	0.91	3.41E-01
MSMEG_1871	conserved hypothetical protein		0.57	1.77E-03	1.19	3.34E-01	0.81	2.20E-01
MSMEG_1872	conserved hypothetical protein		0.59	3.78E-04	1.28	7.00E-02	0.53	3.77E-02
MSMEG_1873	thymidylate kinase		0.39	7.16E-06	1.22	2.53E-01	1.18	5.29E-01
MSMEG_1874	DNA-binding response regulator MtrA	*mtrA*	0.98	6.37E-01	1.11	7.54E-02	1.22	1.66E-01
MSMEG_1875	sensor histidine kinase MtrB		1.47	5.64E-01	2.08	2.34E-01	1.84	3.40E-01
MSMEG_1876	LpqB protein		0.67	1.80E-03	1.22	5.41E-02	2.46	4.13E-02
MSMEG_1877	conserved hypothetical protein		1.36	9.48E-02	1.07	4.57E-01	3.57	3.36E-01
MSMEG_1878	S30AE family protein		2.75	3.11E-04	1.21	8.44E-02	1.95	1.31E-02
MSMEG_1879	hypothetical protein		1.20	4.65E-01	1.35	1.27E-01	1.09	4.08E-01
MSMEG_1880	hypothetical alanine arginine proline rich protein		1.21	1.98E-01	1.80	3.72E-01	0.92	6.41E-01
MSMEG_1881	preprotein translocase, SecA subunit	*secA*	1.05	4.50E-01	0.84	1.21E-02	0.93	1.64E-01
MSMEG_1882	acyltransferase, ws/dgat/mgat subfamily protein		0.89	1.35E-01	0.97	6.48E-01	1.08	5.67E-01
MSMEG_1883	glycine betaine transporter OpuD		1.67	4.23E-03	0.76	2.37E-02	0.73	6.31E-02
MSMEG_1884	conserved hypothetical protein		0.48	2.95E-05	0.78	2.22E-02	1.10	3.88E-01

MSMEG_1885	2Fe-2S iron-sulfur cluster binding domain protein		0.58	1.63E-02	1.31	5.82E-01	0.96	8.96E-01
MSMEG_1886	Fatty acid desaturase		0.54	2.53E-02	2.09	9.96E-03	0.29	2.43E-04
MSMEG_1887	hypothetical protein		2.98	4.17E-04	0.58	1.36E-02	0.72	1.21E-01
MSMEG_1888	methyltransferase		1.37	5.77E-02	0.72	1.55E-02	1.64	1.24E-01
MSMEG_1889	putative ribosome small subunit-dependent GTPase RsgA		0.62	1.94E-03	1.13	6.46E-03	1.37	4.30E-01
MSMEG_1890	3-phosphoshikimate 1-carboxyvinyltransferase	*aroA*	0.64	5.24E-04	1.00	9.62E-01	1.68	3.33E-02
MSMEG_1891	conserved hypothetical protein		1.46	7.23E-04	1.01	9.05E-01	1.31	2.69E-01
MSMEG_1892	transferase		0.64	4.44E-02	0.89	9.47E-02	1.28	2.41E-02
MSMEG_1893	UbiE/COQ5 methyltransferase		1.39	1.57E-02	0.75	3.26E-02	0.92	6.15E-01
MSMEG_1894	short chain dehydrogenase		1.51	8.66E-03	1.04	6.90E-01	0.48	4.12E-04
MSMEG_1895	HTH-type transcriptional regulator AlsR		0.95	5.86E-01	1.29	3.55E-01	0.97	2.61E-01
MSMEG_1896	beta-ketothiolase		3.22	3.24E-02	0.84	4.19E-01	1.14	3.22E-01
MSMEG_1897	3-oxoadipate enol-lactonase	*pcaD*	2.56	3.50E-02	0.82	6.87E-02	0.83	1.94E-01
MSMEG_1898	succinyl-CoA:3-ketoacid-coenzyme A transferase 1, precursor		3.90	1.45E-02	0.75	1.71E-04	0.81	3.00E-01
MSMEG_1899	succinyl-CoA:3-ketoacid-coenzyme A transferase subunit B		3.26	2.19E-02	0.66	3.38E-03	1.04	3.19E-01
MSMEG_1900	D-alanyl-D-alanine carboxypeptidase family protein		0.70	1.26E-02	0.81	1.83E-02	1.27	2.81E-01
MSMEG_1900	D-alanyl-D-alanine carboxypeptidase family protein		0.77	5.30E-02	1.05	6.34E-01	0.93	5.46E-01
MSMEG_1901	DNA-binding HTH domain containing protein, putative		1.38	1.01E-04	1.23	4.18E-02	1.48	9.98E-02
MSMEG_1902	transcriptional regulator		1.25	1.60E-03	0.86	2.37E-01	1.06	5.00E-01
MSMEG_1903	caib/baif family protein		3.40	8.21E-04	0.71	6.00E-03	1.00	9.65E-01
MSMEG_1904	acyl-CoA dehydrogenase		4.95	1.32E-02	0.77	7.65E-02	0.92	4.85E-01
MSMEG_1905	acyl-CoA dehydrogenase protein		3.59	1.24E-02	0.82	1.05E-01	1.01	9.25E-01
MSMEG_1906	toluate 1,2-dioxygenase electron transfer component		5.86	1.40E-01	1.22	7.77E-02	0.59	1.48E-02
MSMEG_1907	toluate 1,2-dioxygenase beta subunit		5.14	5.26E-02	1.59	8.89E-02	0.31	2.27E-03
MSMEG_1908	benzoate 1,2-dioxygenase alpha subunit		4.99	4.56E-02	1.31	1.00E-02	1.14	8.15E-01
MSMEG_1909	putative HTH-type transcriptional regulator YnfL		2.56	1.31E-02	0.91	4.60E-01	0.88	1.43E-01
MSMEG_1910	muconate cycloisomerase		3.01	1.37E-02	0.71	7.16E-03	1.04	8.77E-01
MSMEG_1911	catechol 1,2-dioxygenase	*catA*	2.94	6.26E-04	0.98	8.99E-01	0.25	1.64E-04
MSMEG_1912	muconolactone delta-isomerase 1		2.02	1.01E-03	1.02	7.94E-01	0.72	1.74E-01
MSMEG_1913	YbaK/ebsC protein	*ybaK*	0.46	1.61E-03	1.30	2.56E-01	1.59	1.44E-01
MSMEG_1914	RNA polymerase sigma-70 factor, family protein		1.85	9.56E-04	1.11	2.83E-01	0.63	1.89E-02

MSMEG_1915	anti-sigma factor, family protein		1.10	9.68E-02	0.96	7.76E-01	1.79	1.94E-02
MSMEG_1916	hypothetical protein		1.24	1.39E-01	0.72	1.30E-01	1.73	1.52E-01
MSMEG_1917	conserved domain protein		0.43	2.36E-06	1.45	9.53E-02	0.83	2.59E-01
MSMEG_1918	sensor histidine kinase		0.65	6.89E-02	2.22	9.92E-02	1.13	1.57E-01
MSMEG_1919	Transcription factor WhiB		0.59	3.38E-03	0.62	3.87E-02	8.33	4.48E-03
MSMEG_1920	diacylglycerol kinase, catalytic region		0.33	1.34E-04	0.98	7.08E-01	2.48	2.64E-03
MSMEG_1921	SEC-C motif domain protein		0.68	1.72E-02	1.04	7.33E-01	1.78	3.64E-01
MSMEG_1922	3-dehydroquinate dehydratase, type II	*aroQ*	0.46	4.72E-04	1.33	2.06E-01	1.62	2.26E-02
MSMEG_1923	membrane protein		0.51	4.33E-04	1.47	1.11E-01	1.42	8.98E-02
MSMEG_1924	N-acetyltransferase Ats1		0.65	4.25E-04	0.97	2.50E-01	2.99	3.52E-01
MSMEG_1925	isochorismate synthase DhbC		0.45	2.03E-05	1.09	2.19E-01	1.27	3.84E-01
MSMEG_1926	phosphoglycerate mutase family protein		0.86	3.79E-02	0.77	1.43E-02	2.03	1.29E-02
MSMEG_1927	cobyrinic Acid a,c-diamide synthase		1.18	2.22E-01	0.89	1.11E-01	0.69	2.08E-04
MSMEG_1928	regulatory protein		1.67	1.06E-01	1.03	6.07E-01	1.65	2.06E-02
MSMEG_1929	conserved hypothetical protein		0.49	5.16E-04	1.06	2.52E-01	3.13	7.79E-02
MSMEG_1930	DEAD/DEAH box helicase		2.00	7.39E-03	0.97	7.50E-01	1.15	3.56E-01
MSMEG_1931	conserved hypothetical protein		1.05	4.30E-01	0.81	2.88E-02	0.91	6.83E-01
MSMEG_1932	MmpS3 protein		1.49	1.02E-01	0.74	2.80E-01	1.27	5.19E-01
MSMEG_1933	hypothetical protein		0.93	4.57E-01	0.84	1.18E-02	0.93	7.96E-01
MSMEG_1934	ATP-binding protein		1.21	5.49E-03	0.86	1.54E-01	1.03	7.70E-01
MSMEG_1935	TetR-family protein transcriptional regulator		1.00	9.67E-01	0.81	1.27E-01	0.96	4.02E-01
MSMEG_1936	conserved hypothetical protein		1.13	3.75E-01	0.75	2.42E-02	0.97	9.15E-01
MSMEG_1937	molybdopterin biosynthesis protein MoeB		1.35	5.14E-03	1.11	3.54E-01	1.19	1.02E-01
MSMEG_1938	conserved hypothetical protein		0.71	3.52E-03	2.46	4.95E-02	1.13	3.00E-01
MSMEG_1939	6-O-methylguanine DNA methyltransferase, DNA binding domain subfamily protein		1.24	1.80E-01	1.07	3.39E-01	1.64	1.02E-01
MSMEG_1940	hydrolase, alpha/beta fold family protein		2.65	3.30E-01	1.46	5.00E-01	1.15	4.46E-01
MSMEG_1941	helicase, UvrD/Rep family protein		0.98	9.09E-01	1.63	2.24E-01	2.94	7.16E-02
MSMEG_1942	integral membrane protein		1.38	8.95E-02	0.95	3.60E-01	1.69	5.07E-02
MSMEG_1943	ATP-dependent DNA helicase		0.61	6.83E-02	1.33	3.03E-01	2.19	4.15E-02
MSMEG_1944	membrane protein		1.77	3.46E-03	1.01	9.45E-01	0.72	8.61E-04
MSMEG_1945	ion channel membrane protein		0.50	2.36E-05	1.10	7.05E-02	1.52	5.62E-02
MSMEG_1946	NADH pyrophosphatase		0.56	2.24E-04	1.03	5.11E-01	1.12	4.91E-01
MSMEG_1947	conserved hypothetical protein		1.18	1.67E-01	0.92	6.00E-01	1.69	5.99E-02
MSMEG_1948	hypothetical protein		1.30	2.23E-01	0.97	8.95E-01	0.94	4.59E-01
MSMEG_1949	hypothetical protein		0.94	3.02E-01	1.50	1.05E-01	1.05	2.75E-01
MSMEG_1950	conserved hypothetical protein		2.50	3.34E-01	1.64	4.59E-02	1.17	6.12E-01
MSMEG_1951	conserved domain protein		1.49	5.00E-01	2.30	5.65E-02	0.90	5.53E-01

MSMEG_1952	ATP-dependent DNA helicase		0.73	6.09E-02	0.94	5.82E-01	1.49	1.76E-01
MSMEG_1953	transcription factor WhiB		0.78	4.40E-01	1.65	3.40E-01	1.54	2.10E-01
MSMEG_1954	ABC1 family protein		0.94	4.41E-01	0.78	4.55E-03	0.59	9.60E-03
MSMEG_1955	conserved hypothetical protein		0.40	8.39E-04	1.18	4.19E-01	1.39	1.35E-01
MSMEG_1956	hypothetical protein		0.61	9.09E-04	1.47	9.99E-02	0.99	3.26E-01
MSMEG_1957	conserved hypothetical protein		1.21	1.66E-02	0.96	5.47E-01	1.44	2.58E-02
MSMEG_1958	PDZ domain family protein		0.50	3.26E-04	1.18	9.94E-02	0.93	4.61E-01
MSMEG_1959	putative membrane protein		1.05	6.10E-01	0.82	9.21E-02	0.58	6.80E-03
MSMEG_1960	hypothetical protein		1.20	1.91E-01	1.05	3.95E-01	0.74	2.47E-02
MSMEG_1961	conserved hypothetical protein		1.06	5.89E-01	1.12	4.65E-01	0.65	6.20E-02
MSMEG_1962	hypothetical protein		1.00	9.86E-01	1.05	6.99E-01	0.86	3.36E-01
MSMEG_1963	putative transcriptional regulatory protein		0.70	3.22E-04	1.02	7.52E-01	1.14	3.16E-01
MSMEG_1964	mitomycin radical oxidase		0.81	3.59E-01	1.10	6.49E-01	1.19	3.93E-01
MSMEG_1966	hypothetical protein		6.06	1.10E-02	0.60	8.62E-03	8.28	1.26E-01
MSMEG_1967	TetR-family protein transcriptional regulator		0.84	5.75E-03	1.03	9.08E-01	1.55	1.79E-01
MSMEG_1968	putative oxidoreductase		0.84	1.62E-02	1.21	6.40E-02	1.43	6.20E-03
MSMEG_1969	MerR-family protein transcriptional regulator		1.91	1.71E-02	0.76	1.43E-01	1.42	1.54E-01
MSMEG_1970	sigma factor		13.23	7.25E-03	1.10	2.01E-01	0.87	3.07E-01
MSMEG_1971	propane monooxygenase hydroxylase large subunit		50.66	3.66E-02	1.17	3.27E-01	0.08	3.09E-06
MSMEG_1972	methane monooxygenase component C		30.92	2.32E-02	1.92	1.26E-01	0.07	4.55E-06
MSMEG_1973	propane monooxygenase hydroxylase small subunit; this gene contains a frame shift which is not the result of sequencing error		22.81	1.05E-02	1.62	6.75E-02	0.04	2.51E-07
MSMEG_1973	propane monooxygenase hydroxylase small subunit; this gene contains a frame shift which is not the result of sequencing error		40.06	4.09E-02	1.45	7.56E-02	0.07	6.17E-06
MSMEG_1974	propane monooxygenase coupling protein		24.98	2.67E-02	1.57	2.82E-01	0.04	3.52E-07
MSMEG_1975	amidohydrolase 2		37.86	2.13E-02	1.72	1.19E-01	0.03	4.84E-08
MSMEG_1976	conserved hypothetical protein		20.54	2.07E-02	1.22	1.74E-01	0.07	7.48E-06
MSMEG_1977	alcohol dehydrogenase		17.87	1.93E-02	1.30	2.16E-01	0.08	1.39E-05
MSMEG_1978	chaperonin GroL	*groL*	20.61	1.28E-02	1.21	2.99E-01	0.07	4.57E-06
MSMEG_1979	antibiotic biosynthesis monooxygenase		17.86	3.64E-02	1.50	1.14E-01	0.14	7.17E-05
MSMEG_1980	monooxygenase		1.92	8.93E-03	0.81	7.13E-02	0.57	4.47E-03
MSMEG_1981	conserved hypothetical protein		1.90	1.18E-03	0.76	4.18E-02	0.54	1.89E-03
MSMEG_1982	acyl-CoA synthase		1.08	7.80E-01	1.17	2.67E-01	0.88	3.60E-01
MSMEG_1983	major facilitator family protein transporter		3.10	7.71E-02	2.07	4.48E-01	1.40	1.78E-01
MSMEG_1984	haloacetate dehalogenase H-1		9.82	4.61E-03	0.80	1.50E-01	0.90	1.45E-01

MSMEG_1985	transcriptional regulator, LysR family protein		3.36	3.39E-03	0.79	3.76E-02	1.20	4.58E-01
MSMEG_1986	tartrate dehydrogenase		11.95	2.29E-03	0.59	3.41E-02	0.92	8.18E-01
MSMEG_1987	conserved hypothetical protein		1.38	3.33E-01	0.71	1.97E-01	2.36	1.28E-01
MSMEG_1988	conserved hypothetical protein		1.05	8.62E-01	0.73	1.04E-01	1.03	5.85E-01
MSMEG_1989	phenoxybenzoate dioxygenase beta subunit		0.82	3.59E-01	0.77	5.55E-02	1.05	5.62E-01
MSMEG_1990	conserved hypothetical protein		1.32	2.10E-01	0.83	5.70E-01	1.03	5.33E-01
MSMEG_1991	isovaleryl-CoA dehydrogenase		0.87	3.85E-01	1.07	3.10E-01	0.97	2.58E-01
MSMEG_1992	L-carnitine dehydratase/bile acid-inducible protein F		0.96	7.90E-01	1.12	7.06E-01	1.04	6.58E-01
MSMEG_1993	MaoC like domain protein		0.76	4.47E-02	1.14	3.92E-02	1.00	9.06E-01
MSMEG_1994	HpcH/HpaI aldolase		0.69	9.32E-03	1.18	1.00E-01	0.96	4.65E-01
MSMEG_1995	transcriptional regulator MdcY family protein		0.82	2.27E-02	0.97	6.60E-01	0.57	2.18E-03
MSMEG_1996	N5,N10- methylenetetrahydromethanopterin reductase-related protein		1.48	3.05E-03	0.92	2.11E-01	0.74	1.08E-02
MSMEG_1997	N-acyl-D-glutamate amidohydrolase		1.25	6.27E-02	1.26	4.32E-02	0.56	2.99E-03
MSMEG_1998	hydrolase, alpha/beta fold family protein		1.17	3.02E-01	1.12	3.86E-01	0.65	4.32E-04
MSMEG_1999	hypothetical protein		0.72	7.30E-02	0.71	2.27E-02	4.41	5.11E-02
MSMEG_2000	conserved hypothetical protein		1.41	3.17E-01	1.22	4.60E-01	0.78	7.72E-02
MSMEG_2001	sugar transporter		1.03	8.75E-01	1.07	5.59E-01	1.23	4.18E-01
MSMEG_2004	arabinose-proton symporter		1.13	3.64E-01	1.04	6.70E-01	0.92	2.93E-01
MSMEG_2005	sugar phosphate isomerase/epimerase		1.24	2.11E-01	1.09	3.65E-01	1.05	5.30E-01
MSMEG_2006	2,3-dihydroxybiphenyl 1,2-dioxygenase		3.57	5.96E-03	0.90	4.55E-01	0.73	1.55E-01
MSMEG_2007	putative HpcE protein		3.51	5.78E-03	0.78	1.33E-01	0.83	4.25E-01
MSMEG_2008	hypothetical protein		1.09	7.78E-01	1.08	5.13E-01	0.94	4.40E-01
MSMEG_2009	regulatory protein GntR, HTH:GntR, C-		1.41	3.43E-02	0.92	2.21E-01	1.03	7.53E-01
MSMEG_2010	stress responsive A/B Barrel Domain superfamily protein		0.30	2.10E-04	0.87	4.39E-01	2.10	1.49E-01
MSMEG_2011	transcriptional regulator, LacI family protein		0.95	7.66E-01	1.04	7.62E-01	1.10	4.00E-01
MSMEG_2012	histidinol dehydrogenase	*hisD*	0.76	1.78E-01	1.53	2.21E-01	0.97	4.35E-01
MSMEG_2013	oxidoreductase, short chain dehydrogenase/reductase family protein		1.20	4.32E-01	1.20	3.52E-01	0.99	3.12E-01
MSMEG_2014	molybdenum import ATP-binding protein ModC		0.28	1.50E-03	2.16	5.00E-02	3.94	1.16E-02
MSMEG_2015	molybdate ABC transporter, permease protein	*modB*	0.10	8.87E-07	3.49	6.45E-03	3.59	1.80E-02
MSMEG_2016	molybdate ABC transporter, periplasmic molybdate-binding protein	*modA*	0.29	4.28E-05	1.93	1.73E-02	1.57	3.46E-02
MSMEG_2017	transcriptional regulator, MerR family protein		0.27	6.86E-06	2.25	2.99E-03	1.83	3.94E-03

MSMEG_2018	cytochrome P450 107B1; this gene contains a frame shift which is not the result of sequencing error	1.01	8.99E-01	1.29	3.72E-01	1.20	3.90E-01
MSMEG_2019	short-chain dehydrogenase/reductase SDR	0.59	5.24E-05	0.89	7.69E-02	1.08	6.74E-01
MSMEG_2020	hyi family protein, authentic frameshift; this gene contains a frame shift which is not the result of sequencing error; identified by similarity to RF:NP_457306.1	0.79	4.35E-03	1.07	5.98E-01	0.83	2.22E-01
MSMEG_2021	lipopolysaccharide biosynthesis acyltransferase, M	0.57	1.00E-03	1.05	4.16E-01	1.16	3.42E-01
MSMEG_2022	GntT protein, putative	1.79	1.42E-01	0.82	7.14E-02	0.98	8.76E-01
MSMEG_2023	CAIB/BAIF family protein	1.63	1.44E-01	0.95	6.03E-01	1.17	1.90E-01
MSMEG_2024	hydroxymethylglutaryl-CoA lyase	1.79	4.95E-02	1.09	4.35E-01	0.92	1.49E-01
MSMEG_2025	putative IclR family protein transcriptional regulator	0.98	8.24E-01	1.24	2.67E-01	0.75	7.41E-02
MSMEG_2026	short chain dehydrogenase	0.58	2.00E-04	0.89	2.34E-01	1.30	1.44E-03
MSMEG_2027	conserved hypothetical protein TIGR00026	2.84	5.92E-03	1.41	3.75E-01	0.59	5.26E-02
MSMEG_2028	chloramphenicol resistance protein	0.90	3.85E-01	1.49	7.20E-02	1.06	1.94E-01
MSMEG_2029	3-ketoacyl-ACP/CoA redutase	0.92	6.60E-01	1.07	6.38E-01	0.98	3.75E-01
MSMEG_2030	transcriptional regulator, TetR family protein	1.29	6.81E-02	0.95	6.84E-01	1.44	1.24E-01
MSMEG_2031	hypothetical protein	1.33	2.27E-01	1.09	5.03E-01	1.17	3.48E-01
MSMEG_2032	conserved hypothetical protein	1.54	7.71E-03	0.84	1.27E-01	1.50	7.63E-02
MSMEG_2033	oxidoreductase, zinc-binding dehydrogenase family protein	0.51	4.32E-04	1.05	5.26E-01	3.38	1.47E-02
MSMEG_2034	aryl-alcohol dehydrogenase	1.27	2.80E-01	0.92	4.90E-01	0.99	7.28E-01
MSMEG_2035	amine oxidase [flavin-containing] B	0.95	7.53E-01	1.39	6.67E-02	0.64	1.74E-02
MSMEG_2036	hydrolase, alpha/beta fold family protein	1.60	6.06E-02	1.01	9.68E-01	0.98	8.95E-01
MSMEG_2037	conserved hypothetical protein	0.41	1.72E-04	1.04	7.36E-01	1.00	9.86E-01
MSMEG_2038	monooxygenase, flavin-binding family protein	1.00	9.82E-01	1.27	8.37E-02	0.58	7.51E-06
MSMEG_2039	putative transcriptional regulator	1.43	3.43E-02	0.87	3.34E-01	0.98	8.25E-01
MSMEG_2040	hypothetical protein	0.89	7.14E-02	1.25	2.85E-01	1.81	4.02E-01
MSMEG_2041	conserved hypothetical protein; this gene contains a frame shift which is not the result of sequencing error	1.03	7.32E-01	0.97	7.28E-01	0.55	3.22E-02
MSMEG_2042	phosphotransferase enzyme family protein	1.18	5.02E-03	1.01	8.42E-01	0.53	2.07E-02
MSMEG_2043	transcriptional regulator, TetR family protein	1.06	8.53E-01	2.08	1.87E-01	1.81	2.31E-01
MSMEG_2044	integral membrane transport protein	0.63	2.05E-02	1.36	4.06E-01	5.28	1.00E-02
MSMEG_2045	monoxygenase	0.50	1.18E-05	1.30	2.86E-01	2.37	2.85E-03

MSMEG_2046	GlcNAc-PI de-N-acetylase family protein		0.64	2.80E-02	1.07	1.43E-01	2.13	9.88E-02
MSMEG_2047	probable enoyl-CoA hydratase		0.80	9.60E-02	1.61	3.45E-01	0.81	1.10E-01
MSMEG_2048	alcohol dehydrogenase		0.95	4.50E-01	0.91	1.70E-01	1.31	3.08E-01
MSMEG_2049	putative transcriptional regulator, TetR family protein		1.24	1.72E-01	1.22	2.43E-01	0.93	5.89E-01
MSMEG_2050	NADH-quinone oxidoreductase, N subunit	*nuoN*	3.39	6.76E-02	0.97	7.03E-01	0.45	3.76E-02
MSMEG_2051	NADH-quinone oxidoreductase, M subunit	*nuoM*	4.27	2.18E-02	0.99	9.26E-01	0.19	7.95E-04
MSMEG_2052	NADH-quinone oxidoreductase, L subunit	*nuoL*	3.46	3.07E-02	0.84	7.15E-02	0.50	6.62E-02
MSMEG_2053	NADH-quinone oxidoreductase, k subunit	*nuoK*	4.95	1.88E-02	0.85	7.70E-02	0.12	7.78E-05
MSMEG_2054	NADH dehydrogenase subunit j		4.47	9.87E-02	0.86	1.61E-01	0.19	1.17E-03
MSMEG_2055	NADH-quinone oxidoreductase, I subunit	*nuoI*	2.36	1.57E-01	1.04	4.84E-01	0.73	2.17E-01
MSMEG_2056	NADH-quinone oxidoreductase, H subunit	*nuoH*	6.08	1.03E-01	1.02	8.19E-01	0.24	5.72E-03
MSMEG_2057	NADH-quinone oxidoreductase, G subunit	*nuoG*	5.15	3.28E-02	1.01	8.95E-01	0.10	2.58E-05
MSMEG_2058	NADH-quinone oxidoreductase, F subunit	*nuoF*	19.12	3.96E-02	0.77	4.34E-01	0.10	1.82E-05
MSMEG_2059	NADH-quinone oxidoreductase chain e		4.18	3.31E-02	1.10	5.31E-01	0.22	7.38E-04
MSMEG_2060	NADH-quinone oxidoreducatase, D subunit	*nuoD*	4.90	1.09E-01	0.68	2.51E-02	0.50	7.77E-02
MSMEG_2061	NADH-quinone oxidoreductase chain c		3.14	5.33E-02	1.06	5.27E-01	0.16	1.67E-05
MSMEG_2062	NADH-quinone oxidoreductase, B subunit	*nuoB*	7.69	4.44E-02	0.76	6.14E-02	0.16	3.22E-04
MSMEG_2063	NADH-quinone oxidoreductase, a subunit	*nuoA*	6.64	7.99E-02	0.91	6.22E-01	0.75	3.65E-01
MSMEG_2064	two-component system response regulator		3.97	3.26E-03	0.76	6.79E-02	0.59	2.33E-03
MSMEG_2065	YceI like family protein		1.57	3.25E-02	1.02	7.17E-01	0.95	5.66E-01
MSMEG_2066	conserved hypothetical protein		1.28	1.88E-01	0.91	3.36E-01	1.43	1.79E-01
MSMEG_2067	methyltransferase type 12		1.31	5.52E-02	1.13	3.57E-01	1.09	3.38E-01
MSMEG_2068	hypothetical protein		1.22	4.27E-02	0.87	1.93E-01	1.93	2.58E-01
MSMEG_2069	phosphotransferase enzyme family protein		1.22	7.85E-02	0.79	8.06E-02	1.88	6.71E-02
MSMEG_2070	acyl-CoA dehydrogenase family protein		1.52	1.80E-02	0.84	1.32E-01	1.11	4.98E-01
MSMEG_2071	transcriptional regulator, TetR family protein		1.50	1.77E-01	1.16	6.43E-01	1.10	6.96E-01
MSMEG_2072	3-hydroxy-3-methylglutaryl-CoA lyase		1.40	6.06E-02	0.72	1.39E-02	1.60	1.53E-01
MSMEG_2073	CAIB/BAIF family protein		0.89	4.65E-02	0.94	1.04E-02	1.14	6.56E-01
MSMEG_2074	conserved hypothetical protein		0.66	3.47E-03	0.97	3.79E-01	0.64	1.54E-01
MSMEG_2075	homogentisate 1,2-dioxygenase	*hmgA*	1.33	2.43E-03	0.94	2.93E-01	0.48	1.39E-02
MSMEG_2076	conserved hypothetical protein		1.08	4.15E-01	1.20	4.37E-01	1.36	7.03E-02
MSMEG_2077	acyl-CoA dehydrogenase, C- domain protein		0.83	1.13E-01	0.98	7.68E-01	1.06	6.35E-01
MSMEG_2078	antigen 85-C		2.47	9.30E-04	0.73	2.54E-02	0.60	4.13E-02
MSMEG_2079	alcohol dehydrogenase		1.08	5.65E-01	0.72	2.75E-04	1.88	3.76E-03
MSMEG_2080	putative acyl-CoA dehydrogenase		1.33	1.38E-01	0.71	1.27E-01	2.87	6.25E-02
MSMEG_2081	putative acyl-CoA dehydrogenase		1.43	8.36E-02	0.77	1.25E-01	2.20	9.21E-02
MSMEG_2082	hypothetical protein		1.22	2.80E-01	0.44	4.44E-03	1.03	6.00E-01

MSMEG_2083	inositol monophosphatase	*hisN*	0.55	1.49E-04	1.26	5.29E-01	1.37	3.13E-01
MSMEG_2084	conserved hypothetical protein		1.15	1.43E-01	0.90	1.71E-01	1.82	8.23E-03
MSMEG_2085	NADPH-ferredoxin reductase fpra; this gene contains a frame shift which is not the result of sequencing error; identified by match to protein family HMM PF07992		1.89	3.90E-04	0.90	7.98E-02	0.82	8.62E-02
MSMEG_2086	peptide chain release factor 2	*prfB*	0.70	3.08E-03	1.08	3.17E-01	1.50	8.10E-02
MSMEG_2087	transporter, small conductance mechanosensitive ion channel (MscS) family protein		0.62	1.03E-03	0.81	5.36E-02	1.95	4.50E-02
MSMEG_2088	hypothetical proline-rich protein		0.70	1.24E-03	0.78	1.28E-02	2.40	3.09E-02
MSMEG_2089	cell division ATP-binding protein FtsE	*ftsE*	0.54	4.12E-04	0.73	6.00E-03	1.58	9.18E-02
MSMEG_2090	putative cell division protein FtsX		0.47	1.89E-04	0.95	4.68E-01	1.34	1.87E-01
MSMEG_2091	SsrA-binding protein	*smpB*	0.52	1.29E-04	0.96	6.60E-01	1.50	3.61E-01
MSMEG_2092	D-aminopeptidase		1.23	3.66E-02	1.08	2.05E-01	0.31	5.22E-06
MSMEG_2094	56kDa selenium binding protein		1.23	1.78E-01	0.94	4.26E-01	1.08	4.33E-01
MSMEG_2095	serine esterase, cutinase family protein		1.69	4.40E-03	1.08	3.23E-01	0.46	1.11E-05
MSMEG_2096	integral membrane protein		1.00	9.78E-01	1.07	3.15E-01	0.73	2.58E-02
MSMEG_2097	Acyl-coenzyme A:6-aminopenicillanic acid acyl-transferase		1.48	4.49E-01	1.28	4.27E-02	0.91	2.15E-01
MSMEG_2098	fumarylacetoacetate hydrolase family protein		0.81	4.62E-01	1.07	4.83E-01	1.38	3.90E-01
MSMEG_2099	ABC transporter, substrate binding protein [nitrate/sulfonate], putative		0.77	4.11E-01	1.73	2.22E-01	1.29	4.58E-01
MSMEG_2100	peptidase family protein M20/M25/M40		0.63	5.75E-03	1.28	2.94E-01	1.00	9.19E-01
MSMEG_2101	taurine ABC transporter, permease protein; this gene contains a frame shift which is not the result of sequencing error; identified by match to protein family HMM PF00528		0.93	2.48E-01	1.06	5.97E-01	2.79	3.57E-01
MSMEG_2102	nitrate transport ATP-binding protein NrtD		0.78	3.70E-02	0.97	7.95E-01	1.00	9.91E-01
MSMEG_2103	5,10-methylenetetrahydromethanopterin		1.64	2.36E-01	0.90	8.78E-02	1.09	3.23E-01
MSMEG_2104	GntR-family protein transcriptional regulator		1.15	4.14E-01	0.97	7.80E-01	0.95	4.04E-03
MSMEG_2105	ATP dependent DNA ligase		1.25	5.37E-02	1.08	5.20E-01	0.91	3.45E-01
MSMEG_2106	putative transcriptional regulator		0.96	6.48E-01	0.94	3.38E-01	1.33	3.38E-01
MSMEG_2107	conserved hypothetical protein		0.98	8.20E-01	1.16	1.71E-01	0.49	3.65E-03
MSMEG_2108	conserved hypothetical protein		1.27	8.81E-02	1.13	2.79E-01	1.05	6.46E-01
MSMEG_2109	conserved hypothetical protein		1.13	2.88E-01	0.91	1.20E-01	0.80	1.89E-01
MSMEG_2110	conserved hypothetical protein		1.26	6.03E-03	0.77	2.41E-02	0.52	1.64E-03
MSMEG_2111	chorismate mutase		1.18	4.46E-02	0.87	3.08E-01	0.51	1.41E-02
MSMEG_2112	secreted protein		1.13	5.85E-01	1.88	6.17E-02	0.40	9.75E-04

MSMEG_2113	hypothetical protein		0.74	5.53E-02	1.43	1.41E-01	1.24	2.91E-01
MSMEG_2114	glucose-6-phosphate isomerase, putative		0.53	3.08E-03	1.04	5.98E-01	1.19	3.53E-01
MSMEG_2115	conserved hypothetical protein		1.31	5.11E-01	2.18	2.58E-02	0.48	7.30E-04
MSMEG_2116	PTS system, glucose-specific IIBC component		0.47	6.69E-06	1.12	7.14E-02	0.33	7.70E-04
MSMEG_2117	beta-glucoside-specific EII permease		0.54	8.28E-04	1.19	2.26E-01	0.39	3.49E-04
MSMEG_2118	glucosamine-6-phosphate isomerase	*nagB*	0.38	4.39E-05	1.02	8.45E-01	0.60	4.12E-02
MSMEG_2119	N-acetylglucosamine-6-phosphate deacetylase	*nagA*	0.38	1.38E-05	1.01	8.85E-01	0.92	5.48E-01
MSMEG_2120	tetratricopeptide repeat family protein		0.93	1.79E-01	1.12	1.51E-01	1.21	4.58E-01
MSMEG_2121	multiphosphoryl transfer protein (MTP)		0.55	3.53E-03	3.27	1.16E-01	0.91	2.65E-01
MSMEG_2122	dihydroxyacetone kinase, L subunit		0.69	1.49E-02	3.41	1.17E-01	0.83	1.10E-01
MSMEG_2123	dihydroxyacetone kinase, DhaK subunit	*dhaK*	0.69	6.83E-03	3.26	1.12E-01	0.46	2.04E-05
MSMEG_2124	glycerol uptake facilitator, MIP channel		0.51	1.37E-03	8.08	1.30E-01	1.06	3.91E-01
MSMEG_2125	glycerol operon regulatory protein		0.62	2.63E-02	1.09	1.27E-01	1.00	9.43E-01
MSMEG_2126	hypothetical protein		0.82	2.95E-01	1.09	3.78E-02	1.09	1.87E-01
MSMEG_2127	conserved hypothetical protein		1.54	1.22E-01	1.01	7.04E-01	1.09	6.50E-01
MSMEG_2128	malonyl CoA decarboxylase		0.85	6.12E-01	0.99	8.28E-01	1.30	4.78E-01
MSMEG_2129	hypothetical protein		1.38	2.91E-01	1.01	9.34E-01	1.13	4.64E-01
MSMEG_2130	putative acyl-CoA dehydrogenase		0.43	8.61E-04	1.10	6.36E-01	2.96	1.77E-01
MSMEG_2131	acyl-CoA synthase		0.24	2.61E-04	1.16	3.12E-01	3.28	8.34E-02
MSMEG_2132	acyl carrier protein Acp		0.60	4.14E-02	0.96	7.27E-01	2.13	3.74E-02
MSMEG_2133	conserved hypothetical protein, putative		0.89	2.08E-01	1.11	2.13E-01	1.56	3.76E-01
MSMEG_2134	CrcB protein	*crcB*	0.98	7.53E-01	0.92	4.25E-01	1.11	1.99E-01
MSMEG_2135	putative CrcB protein		1.11	1.99E-01	1.01	8.95E-01	1.16	3.24E-01
MSMEG_2136	phosphoglucomutase, alpha-D-glucose phosphate-specific	*pgm*	1.00	9.90E-01	0.98	7.89E-01	0.72	8.68E-03
MSMEG_2137	permease of the major facilitator superfamily protein		0.44	7.53E-05	1.07	4.35E-01	1.74	3.34E-01
MSMEG_2139	hypothetical protein		0.92	5.98E-01	0.98	7.50E-01	1.03	6.27E-01
MSMEG_2140	Fic protein family protein		0.82	1.33E-01	1.05	5.13E-01	1.02	8.92E-01
MSMEG_2141	gp35 protein		1.46	1.92E-02	1.25	3.74E-01	1.24	2.56E-01
MSMEG_2142	gp36 protein		1.67	5.31E-02	0.87	1.94E-01	0.88	2.18E-01
MSMEG_2143	hypothetical protein		1.57	9.69E-03	0.89	2.68E-01	1.15	3.11E-01
MSMEG_2144	conserved hypothetical protein		2.45	4.26E-04	1.03	7.69E-01	0.64	2.81E-03
MSMEG_2145	conserved domain protein		1.31	1.06E-01	0.88	9.59E-02	1.77	3.94E-01
MSMEG_2146	gp15 protein		1.19	3.23E-01	1.15	4.43E-01	1.11	6.30E-01
MSMEG_2147	gp16 protein; this gene contains a frame shift which is not the result of sequencing error		1.16	1.12E-01	1.16	6.28E-01	0.88	3.64E-01
MSMEG_2148	HNH endonuclease domain protein		2.04	2.36E-02	0.95	6.97E-01	1.05	3.70E-01

MSMEG_2149	hypothetical protein		2.52	3.33E-03	1.02	9.12E-01	0.78	1.28E-01
MSMEG_2150	putative transposition helper protein		1.37	1.38E-01	1.60	6.95E-02	0.97	3.82E-01
MSMEG_2150	putative transposition helper protein		0.89	5.93E-01	1.21	5.26E-01	1.29	3.87E-01
MSMEG_2151	transposase IstA protein, putative		0.70	2.13E-02	2.44	6.27E-02	0.88	2.50E-01
MSMEG_2151	transposase IstA protein, putative		0.90	5.81E-01	1.58	1.75E-01	0.99	2.64E-01
MSMEG_2152	hypothetical protein; this gene contains a frame shift which is not the result of sequencing error; identified by Glimmer2; putative		1.49	1.83E-01	1.70	9.40E-02	2.59	3.22E-01
MSMEG_2153	transcriptional regulator, TetR family protein		1.30	4.94E-02	1.50	2.05E-01	0.83	2.01E-01
MSMEG_2154	conserved hypothetical protein		1.08	7.35E-01	1.48	1.54E-01	1.48	4.23E-01
MSMEG_2155	trans-2-enoyl-CoA reductase		1.10	4.27E-01	0.89	9.38E-02	1.46	4.36E-01
MSMEG_2156	conserved hypothetical protein		1.42	3.19E-02	1.00	9.82E-01	1.04	2.84E-01
MSMEG_2157	hypothetical protein		2.06	6.46E-02	0.68	1.86E-03	1.09	6.27E-01
MSMEG_2158	hypothetical protein		0.93	6.80E-01	1.00	9.93E-01	0.98	5.09E-01
MSMEG_2159	conserved hypothetical protein		2.89	1.96E-02	0.66	1.12E-02	0.90	1.77E-01
MSMEG_2160	hypothetical protein		1.60	4.01E-02	1.62	1.39E-02	1.01	9.27E-01
MSMEG_2161	FadD9 protein; this region contains one or more premature stops and/or frameshifts which are not the result of sequencing error		1.95	7.61E-03	0.86	6.53E-02	1.33	5.07E-02
MSMEG_2161	FadD9 protein; this region contains one or more premature stops and/or frameshifts which are not the result of sequencing error		1.53	3.33E-02	1.29	9.96E-02	3.61	1.95E-01
MSMEG_2161	FadD9 protein; this region contains one or more premature stops and/or frameshifts which are not the result of sequencing error		0.92	3.21E-01	1.04	7.10E-01	1.01	9.06E-01
MSMEG_2162	MmcJ protein		0.46	2.02E-03	1.02	7.82E-01	0.83	1.37E-01
MSMEG_2163	aldehyde dehydrogenase		0.75	3.55E-03	0.93	4.46E-01	1.03	8.28E-01
MSMEG_2164	transcriptional regulator, putative		0.56	2.50E-05	0.83	2.45E-02	2.43	4.25E-02
MSMEG_2165	transketolase, N- subunit		1.26	1.90E-01	0.95	7.48E-01	1.08	4.59E-02
MSMEG_2166	transketoloase, C half		1.17	3.87E-01	0.90	2.71E-01	0.94	6.24E-01
MSMEG_2167	aminopeptidase, putative		1.18	1.80E-01	1.43	2.83E-01	1.00	9.67E-01
MSMEG_2168	hypothetical protein		0.87	2.67E-01	1.16	1.49E-01	1.17	4.76E-01
MSMEG_2169	acetyl-CoA carboxylase, carboxyl transferase, beta subunit	*accD*	1.72	1.43E-01	1.10	6.93E-01	18.25	5.77E-02
MSMEG_2170	3-hydroxyisobutyrate dehydrogenase family protein		0.91	7.93E-02	0.90	1.88E-01	2.17	1.11E-01
MSMEG_2171	L-carnitine dehydratase/bile acid-inducible protein F		3.31	1.06E-01	0.86	2.39E-01	34.20	4.86E-02

MSMEG_2172	dicarboxylate-carrier protein		5.88	4.75E-02	0.99	8.38E-01	0.08	2.33E-06
MSMEG_2173	GntR-family protein transcriptional regulator		1.43	1.76E-02	0.84	4.94E-02	1.59	6.58E-02
MSMEG_2174	superfamily protein I DNA or RNA helicase		0.71	5.43E-04	0.85	1.06E-01	1.57	2.47E-01
MSMEG_2175	nitrilotriacetate monooxygenase component A		3.53	1.04E-02	1.06	5.63E-01	0.84	2.93E-01
MSMEG_2176	conserved hypothetical protein		0.87	1.16E-01	0.94	4.26E-01	2.47	4.16E-01
MSMEG_2177	fmnh2-utilizing oxygenase		5.18	1.54E-02	1.34	2.00E-01	0.85	4.00E-01
MSMEG_2178	integral membrane protein		1.23	2.63E-01	1.66	2.77E-01	0.97	6.93E-01
MSMEG_2179	transcriptional regulatory protein		1.68	4.87E-03	0.98	8.55E-01	1.84	7.19E-02
MSMEG_2180	RemK protein		1.45	2.17E-01	1.18	3.01E-01	1.08	4.88E-01
MSMEG_2181	cell filamentation protein		0.99	9.33E-01	1.19	3.95E-01	0.99	9.48E-01
MSMEG_2182	transcriptional regulator		0.69	1.58E-03	0.94	2.45E-02	3.06	3.78E-01
MSMEG_2183	conserved hypothetical protein		1.86	7.62E-02	0.97	8.38E-01	1.02	7.74E-01
MSMEG_2184	amino acid permease		1.03	9.37E-01	1.25	2.55E-01	0.97	7.27E-01
MSMEG_2185	conserved hypothetical protein		0.80	4.41E-02	1.01	9.24E-01	0.80	4.72E-01
MSMEG_2186	conserved hypothetical protein		0.94	6.84E-01	1.17	2.50E-01	0.83	2.83E-01
MSMEG_2187	urea amidolyase		0.83	5.69E-02	0.80	1.37E-01	1.60	6.89E-02
MSMEG_2188	integral membrane protein		0.82	2.39E-01	1.69	1.89E-01	1.14	5.33E-01
MSMEG_2189	allophanate hydrolase	*atzF*	0.73	3.99E-03	0.74	9.92E-02	1.03	1.53E-01
MSMEG_2190	acyl-CoA dehydrogenase domain protein		2.07	1.53E-01	1.16	4.78E-01	1.04	5.81E-01
MSMEG_2191	acyl-CoA dehydrogenase family protein		1.69	2.53E-01	1.41	1.92E-01	1.20	4.36E-01
MSMEG_2192	conserved hypothetical protein		1.97	9.77E-03	0.82	8.19E-02	0.77	1.47E-01
MSMEG_2193	transcriptional regulator, TetR family protein		1.20	5.83E-02	0.86	1.62E-01	1.24	2.51E-02
MSMEG_2194	MerR-family protein transcriptional regulator		32.00	3.23E-02	4.51	2.53E-01	2.12	3.76E-01
MSMEG_2195	transcriptional regulator, TetR family protein		7.06	2.79E-01	0.98	8.68E-01	1.57	2.62E-02
MSMEG_2196	sensory box/response regulator		1.24	4.76E-02	1.02	5.69E-01	7.43	3.10E-01
MSMEG_2197	hypothetical protein		0.92	5.04E-01	1.01	9.52E-01	2.40	1.19E-02
MSMEG_2198	ThiF family protein		1.38	4.46E-02	1.13	3.48E-01	1.60	5.01E-02
MSMEG_2199	conserved hypothetical protein		3.09	5.57E-05	1.00	9.86E-01	0.27	4.52E-07
MSMEG_2200	formyltetrahydrofolate deformylase	*purU*	0.46	4.36E-04	1.04	5.26E-01	1.66	4.53E-03
MSMEG_2201	ZbpA protein		1.02	7.23E-01	0.99	8.81E-01	0.68	2.52E-02
MSMEG_2202	dimethylaniline monooxygenase [N-oxide-forming] 5		0.49	5.36E-05	1.16	1.85E-01	0.42	7.65E-03
MSMEG_2203	alpha/beta hydrolase		0.90	4.96E-01	1.08	3.93E-01	2.03	3.80E-01
MSMEG_2204	carveol dehydrogenase ((+)-trans-carveol dehydrogenase)		1.28	8.57E-02	0.85	1.83E-02	1.04	7.88E-01
MSMEG_2205	putative acyl-CoA dehydrogenase		1.54	3.97E-01	0.74	8.73E-02	1.24	1.31E-01
MSMEG_2206	3-oxoacyl-(acyl-carrier-protein) reductase	*fabG*	1.33	1.04E-01	0.74	5.42E-03	1.91	2.68E-01
MSMEG_2207	beta-ketothiolase		0.78	3.73E-02	0.71	6.38E-03	2.34	2.24E-01

MSMEG_2208	acyl-CoA dehydrogenase	0.80	2.49E-02	0.70	1.38E-03	2.05	1.19E-01
MSMEG_2209	transcriptional regulator, GntR family protein	0.90	3.96E-01	0.99	9.42E-01	2.21	2.24E-01
MSMEG_2210	CaiB/BaiF family protein; this gene contains a frame shift which is not the result of sequencing error	0.94	1.92E-01	0.94	2.28E-01	2.49	2.30E-01
MSMEG_2210	CaiB/BaiF family protein; this gene contains a frame shift which is not the result of sequencing error	1.19	2.44E-01	1.04	2.99E-01	1.17	2.99E-01
MSMEG_2211	DNA-binding protein	2.29	1.48E-02	0.89	3.09E-01	1.39	1.30E-01
MSMEG_2212	short chain dehydrogenase	1.00	9.75E-01	1.09	3.75E-01	0.93	3.30E-01
MSMEG_2213	O-methyltransferase	1.15	1.60E-01	1.24	1.74E-01	1.20	3.01E-01
MSMEG_2214	alcohol dehydrogenase B; this region contains one or more premature stops and/or frameshifts which are not the result of sequencing error	1.43	7.55E-02	1.03	8.23E-01	1.48	1.98E-01
MSMEG_2215	AMP-binding enzyme	1.10	4.28E-01	1.03	8.69E-01	1.01	8.06E-01
MSMEG_2216	AMP-binding enzyme	1.03	8.61E-01	1.13	3.31E-01	1.10	2.98E-01
MSMEG_2217	AMP-dependent synthetase and ligase	1.79	1.22E-01	1.11	4.29E-01	1.01	5.01E-01
MSMEG_2218	enoyl-CoA hydratase/isomerase family protein	1.49	2.78E-01	1.04	6.27E-01	1.00	9.51E-01
MSMEG_2219	enoyl-CoA hydratase/isomerase family protein	0.84	1.02E-02	0.96	5.76E-01	1.20	2.82E-01
MSMEG_2220	enoyl-CoA hydratase/isomerase	1.39	3.28E-01	0.98	7.05E-01	1.02	8.90E-01
MSMEG_2222	hypothetical protein	1.57	7.28E-02	1.12	4.84E-01	1.13	4.70E-02
MSMEG_2222	hypothetical protein	1.03	8.33E-01	0.94	7.11E-01	1.01	7.07E-01
MSMEG_2223	enoyl-CoA hydratase/isomerase family protein	0.90	1.30E-01	1.30	3.52E-01	1.62	2.41E-01
MSMEG_2224	acetyl-CoA C-acyltransferase	0.91	6.21E-01	1.01	8.48E-01	1.37	2.01E-01
MSMEG_2225	transcriptional regulator, TetR family protein	1.05	1.94E-01	1.01	8.33E-01	0.99	9.46E-01
MSMEG_2226	hypothetical protein	1.41	4.04E-04	0.93	4.38E-01	0.94	4.66E-01
MSMEG_2227	carnitinyl-CoA dehydratase	0.94	5.90E-01	1.00	9.93E-01	1.06	3.16E-01
MSMEG_2228	oxidoreductase, short chain dehydrogenase/reductase family protein	0.78	2.31E-02	1.14	4.18E-01	1.14	1.64E-01
MSMEG_2229	enoyl-CoA hydratase	1.75	1.59E-01	1.34	2.78E-02	1.02	8.68E-01
MSMEG_2230	amidohydrolase family protein	1.02	8.29E-01	1.02	8.11E-01	1.16	6.64E-01
MSMEG_2231	AMP-binding enzyme	1.11	4.19E-01	0.88	3.18E-01	1.23	3.53E-01
MSMEG_2232	amidohydrolase family protein	0.84	6.27E-02	1.21	3.70E-01	1.29	2.75E-01
MSMEG_2233	acyl-CoA dehydrogenase, C- domain protein	1.47	3.93E-01	1.27	3.61E-01	1.02	2.24E-01
MSMEG_2234	acyl-CoA dehydrogenase, middle domain protein	0.93	5.94E-01	1.20	2.89E-01	1.05	3.29E-01
MSMEG_2235	amidohydrolase family protein	1.34	1.51E-01	1.02	8.93E-01	3.37	2.39E-01
MSMEG_2236	putative thiolase	1.07	2.04E-01	0.96	4.72E-01	1.02	8.74E-01
MSMEG_2237	anaerobic dehydrogenase, typically	1.48	1.97E-01	1.22	1.32E-01	1.13	3.51E-01

MSMEG_2238	aldehyde dehydrogenase (NAD) family protein		1.32	3.12E-03	1.24	2.11E-01	1.22	3.53E-01
MSMEG_2239	transcriptional regulator, TetR family protein		0.99	8.84E-01	1.10	6.70E-01	1.05	3.57E-01
MSMEG_2240	cytochrome P450		1.02	7.26E-01	1.07	3.82E-01	1.00	9.53E-01
MSMEG_2241	medium chain acyl-CoA synthetase		0.85	3.37E-01	0.98	7.34E-01	0.78	5.94E-02
MSMEG_2242	coniferyl aldehyde dehydrogenase		1.00	9.90E-01	1.16	4.66E-01	0.87	1.61E-01
MSMEG_2243	isochorismatase family protein		0.90	3.17E-01	0.94	6.71E-01	0.94	5.20E-01
MSMEG_2244	Rieske [2Fe-2S] domain protein		2.15	3.51E-01	1.17	3.75E-01	1.30	2.90E-01
MSMEG_2245	bile-acid 7-alpha dehydratase		1.69	5.67E-05	0.90	8.13E-02	0.76	1.29E-01
MSMEG_2246	conserved hypothetical protein		1.38	1.03E-01	1.01	9.45E-01	0.88	2.95E-01
MSMEG_2247	dihydrokaempferol 4-reductase		0.88	3.87E-02	1.54	1.51E-01	0.87	3.19E-01
MSMEG_2248	two-component system sensor kinase		1.15	5.32E-01	0.92	4.34E-01	0.90	5.10E-01
MSMEG_2249	conserved hypothetical protein		0.66	2.27E-04	1.35	6.32E-02	2.08	7.33E-02
MSMEG_2250	hypothetical protein		1.24	2.10E-01	1.22	1.99E-01	1.34	2.87E-01
MSMEG_2251	nitrate/nitrite response regulator protein		1.88	1.55E-02	0.90	4.47E-01	0.82	5.27E-02
MSMEG_2252	flavin-type hydroxylase		0.53	1.47E-03	1.22	1.61E-01	1.12	3.94E-01
MSMEG_2253	hypothetical protein		1.15	5.89E-02	1.39	4.55E-02	1.07	3.23E-01
MSMEG_2254	oxalate decarboxylase OxdC, putative		1.39	3.05E-02	1.33	3.13E-02	2.55	1.39E-01
MSMEG_2255	Carboxyl transferase domain protein		0.97	6.81E-01	1.41	2.51E-01	1.03	3.09E-01
MSMEG_2256	conserved hypothetical protein		0.94	7.11E-01	1.30	7.39E-02	1.02	6.96E-01
MSMEG_2257	cytochrome P450-terp		1.11	6.91E-01	1.12	3.44E-01	1.03	5.34E-01
MSMEG_2258	conserved hypothetical protein		1.30	9.10E-02	1.16	1.53E-01	0.21	9.32E-05
MSMEG_2259	carbon starvation protein A		1.27	2.41E-01	1.02	8.85E-01	0.21	6.50E-06
MSMEG_2260	putative transcriptional regulator		1.32	2.16E-01	1.72	1.33E-01	1.03	4.82E-01
MSMEG_2261	hypothetical protein		6.08	6.11E-03	4.30	4.48E-02	4.12	2.14E-02
MSMEG_2262	hydrogenase-2, small subunit	*hybA*	5.41	3.37E-03	4.09	4.02E-02	5.69	1.89E-02
MSMEG_2263	hydrogenase-2, large subunit	*hybC*	5.43	4.76E-03	3.68	7.62E-02	6.18	1.34E-02
MSMEG_2264	peptidase M52, hydrogen uptake protein		2.27	1.87E-02	3.55	6.51E-02	4.39	5.01E-02
MSMEG_2265	hypothetical protein		1.07	7.63E-02	0.90	3.72E-01	1.00	9.78E-01
MSMEG_2266	hypothetical protein		2.57	1.70E-03	3.88	4.47E-02	6.54	5.70E-02
MSMEG_2267	tetratricopeptide repeat domain protein		3.35	3.80E-03	3.44	8.23E-02	5.20	1.29E-02
MSMEG_2268	conserved hypothetical protein		2.90	9.19E-03	3.43	6.96E-02	9.97	3.75E-02
MSMEG_2269	NHL repeat protein		2.60	2.97E-03	3.61	6.48E-02	16.20	1.46E-02
MSMEG_2270	hypothetical protein		3.65	3.67E-03	4.05	7.74E-02	14.90	7.56E-02
MSMEG_2271	hydrogenase accessory protein HypB	*hypB*	1.52	2.12E-03	2.21	2.68E-02	2.31	1.80E-01
MSMEG_2271	hydrogenase accessory protein HypB	*hypB*	1.31	1.36E-02	2.76	3.45E-02	1.56	1.89E-01
MSMEG_2272	hydrogenase nickel insertion protein HypA	*hypA*	1.88	4.36E-03	2.04	2.08E-02	1.83	1.82E-01
MSMEG_2273	[NiFe] hydrogenase maturation protein HypF	*hypF*	2.04	6.06E-03	2.06	4.02E-02	2.96	8.01E-02
MSMEG_2274	hydrogenase assembly chaperone HypC/HupF	*hypC*	1.78	5.94E-03	3.05	7.02E-02	2.48	1.71E-02

MSMEG_2275	hydrogenase expression/formation protein HypD	*hypD*	1.81	1.15E-03	1.99	4.41E-02	5.24	8.66E-02
MSMEG_2276	hydrogenase expression/formation protein HypE	*hypE*	1.95	1.64E-03	1.71	5.19E-03	1.95	1.13E-01
MSMEG_2277	DNA ligase I, ATP-dependent	*dnl1*	1.33	1.21E-01	1.00	9.83E-01	1.12	3.03E-01
MSMEG_2278	membrane-bound oxidoreductase		1.83	4.58E-02	0.81	3.63E-02	0.82	2.57E-01
MSMEG_2279	3-hydroxyacyl-CoA dehydrogenase type-2		0.60	1.52E-02	1.12	3.44E-01	0.98	3.25E-01
MSMEG_2280	pyruvate dehydrogenase		0.97	8.95E-01	1.10	4.27E-01	0.65	1.92E-03
MSMEG_2281	This gene is disrupted by an IS1096 element.; conserved hypothetical protein, interruption-C; identified by similarity to GB:BAB49125.1; match to protein family HMM PF01638		1.02	8.65E-01	1.16	3.28E-01	1.03	5.79E-01
MSMEG_2284	This gene is disrupted by an IS1096 element.; conserved hypothetical protein, interruption-N; identified by similarity to OMNI:NTL02MT01721		3.83	3.70E-05	0.61	1.33E-02	1.18	4.46E-01
MSMEG_2285	methyltransferase type 11		4.43	1.42E-03	0.94	6.15E-01	0.50	5.40E-04
MSMEG_2286	aminoglycoside phosphotransferase		1.27	1.77E-01	0.79	6.73E-03	0.70	2.33E-02
MSMEG_2286	aminoglycoside phosphotransferase		1.10	3.64E-01	0.96	3.51E-01	0.95	2.95E-01
MSMEG_2287	transport protein		0.28	3.84E-05	1.45	1.45E-01	1.34	3.60E-01
MSMEG_2287	transport protein		0.42	3.11E-03	0.98	5.84E-01	1.12	6.30E-01
MSMEG_2288	conserved hypothetical protein		1.59	1.16E-01	1.19	5.55E-02	1.50	1.11E-01
MSMEG_2288	conserved hypothetical protein		1.40	1.99E-01	0.98	7.51E-01	1.08	6.44E-01
MSMEG_2289	cytochrome p450		4.21	1.95E-04	0.77	8.01E-02	0.17	5.86E-06
MSMEG_2289	cytochrome p450		3.51	4.08E-04	0.87	4.21E-01	0.25	4.67E-05
MSMEG_2290	transcriptional regulator, TetR family protein		1.38	4.83E-03	1.09	1.90E-01	0.70	3.91E-02
MSMEG_2290	transcriptional regulator, TetR family protein		0.93	6.63E-02	0.97	5.83E-01	0.98	8.78E-01
MSMEG_2291	short chain dehydrogenase		0.87	2.09E-02	0.79	1.15E-02	1.39	4.16E-02
MSMEG_2291	short chain dehydrogenase		1.07	3.77E-01	0.88	2.41E-02	1.08	1.56E-01
MSMEG_2292	alkaline phosphatase		0.66	7.08E-03	1.41	6.16E-02	1.23	4.33E-01
MSMEG_2292	alkaline phosphatase		0.83	1.80E-01	0.95	5.03E-01	1.14	5.86E-01
MSMEG_2293	conserved hypothetical protein		0.58	6.22E-03	0.88	1.65E-01	1.09	4.75E-02
MSMEG_2293	conserved hypothetical protein		0.72	9.53E-03	1.15	1.68E-01	0.95	5.83E-01
MSMEG_2294	DNA polymerase IV	*dinB*	1.31	7.81E-02	0.92	1.27E-01	1.69	1.70E-01
MSMEG_2294	DNA polymerase IV	*dinB*	1.07	4.72E-01	1.01	7.78E-01	1.43	3.18E-01
MSMEG_2295	transcriptional regulator, TetR family protein		1.16	2.29E-01	0.87	4.12E-02	2.01	1.10E-01
MSMEG_2295	transcriptional regulator, TetR family protein		0.93	4.65E-01	0.99	8.40E-01	1.25	3.76E-01
MSMEG_2296	secreted protein		1.42	8.47E-03	4.49	1.67E-01	1.05	7.21E-01
MSMEG_2297	glutaredoxin		0.38	9.66E-03	1.86	9.21E-02	2.46	2.27E-01
MSMEG_2297	glutaredoxin		0.43	1.55E-02	2.34	1.12E-01	1.97	3.70E-01

MSMEG_2298	protein NrdI; this gene contains a frame shift which is not the result of sequencing error; identified by match to protein family HMM PF07972	0.35	4.26E-03	1.76	9.64E-02	3.02	1.29E-01
MSMEG_2298	protein NrdI; this gene contains a frame shift which is not the result of sequencing error; identified by match to protein family HMM PF07972	0.39	6.04E-03	2.59	1.56E-01	1.37	3.07E-01
MSMEG_2299	ribonucleoside-diphosphate reductase, alpha subunit	0.30	1.75E-03	2.81	1.56E-01	1.43	2.84E-01
MSMEG_2299	ribonucleoside-diphosphate reductase, alpha subunit	0.41	3.66E-03	2.19	1.76E-01	8.71	2.93E-01
MSMEG_2300	conserved hypothetical protein; this gene contains a frame shift which is not the result of sequencing error	1.26	1.87E-02	1.57	1.37E-01	0.84	1.07E-01
MSMEG_2300	conserved hypothetical protein; this gene contains a frame shift which is not the result of sequencing error	0.67	1.61E-01	1.09	2.62E-01	1.24	1.83E-01
MSMEG_2300	conserved hypothetical protein; this gene contains a frame shift which is not the result of sequencing error	2.39	2.50E-01	1.03	6.81E-01	1.06	5.36E-01
MSMEG_2301	glyoxalase/bleomycin resistance protein/dioxygenase	0.93	8.78E-03	0.86	1.47E-01	0.66	5.98E-02
MSMEG_2301	glyoxalase/bleomycin resistance protein/dioxygenase	0.79	1.49E-02	1.03	3.51E-01	0.99	9.52E-01
MSMEG_2302	conserved hypothetical protein	1.00	9.36E-01	1.93	2.35E-01	1.00	9.63E-01
MSMEG_2303	integral membrane transporter	2.26	8.64E-04	0.82	1.96E-01	0.17	6.96E-05
MSMEG_2303	integral membrane transporter	2.76	1.01E-03	0.90	4.26E-01	0.23	9.71E-05
MSMEG_2304	hypothetical protein	2.38	3.39E-04	0.78	8.89E-02	0.31	4.47E-04
MSMEG_2305	TetR-family protein transcriptional regulator	0.88	4.15E-01	1.03	8.21E-01	1.00	9.96E-01
MSMEG_2306	putative acetyltransferase	0.91	5.18E-01	0.90	3.53E-01	1.15	1.71E-01
MSMEG_2307	conserved hypothetical protein	0.47	6.22E-05	1.46	3.29E-01	0.90	1.46E-01
MSMEG_2307	conserved hypothetical protein	0.67	1.70E-02	1.04	9.06E-01	1.04	3.90E-01
MSMEG_2308	geranylgeranyl reductase	1.14	3.11E-02	0.78	1.29E-01	0.67	8.34E-03
MSMEG_2309	probable transcriptional regulatory protein	0.58	6.76E-04	1.16	1.15E-01	1.18	9.78E-02
MSMEG_2309	probable transcriptional regulatory protein	0.65	1.78E-03	1.07	4.82E-01	0.95	4.79E-01
MSMEG_2310	monooxygenase	1.40	2.13E-03	0.68	4.11E-03	4.05	7.90E-05
MSMEG_2310	monooxygenase	1.14	3.43E-01	0.76	3.26E-02	3.48	1.68E-04

MSMEG_2311	conserved hypothetical protein		0.66	1.01E-03	0.88	2.00E-01	4.05	4.10E-02
MSMEG_2311	conserved hypothetical protein		0.74	3.47E-03	1.04	6.55E-01	2.76	1.62E-01
MSMEG_2312	hypothetical protein		0.50	1.80E-02	1.50	1.65E-01	0.87	2.57E-01
MSMEG_2313	ribonucleoside-diphosphate reductase, beta subunit		0.40	1.09E-03	1.53	4.47E-02	2.01	3.65E-02
MSMEG_2313	ribonucleoside-diphosphate reductase, beta subunit		0.49	3.43E-03	2.20	1.09E-01	1.13	2.94E-01
MSMEG_2314	putative esterase family protein		0.66	6.90E-03	0.78	8.14E-02	3.03	5.51E-03
MSMEG_2314	putative esterase family protein		0.76	3.95E-02	0.86	2.10E-01	4.21	7.46E-03
MSMEG_2315	conserved hypothetical protein		1.45	1.23E-02	0.86	3.92E-02	1.48	3.86E-02
MSMEG_2315	conserved hypothetical protein		1.11	2.59E-01	1.03	4.98E-01	1.33	8.15E-02
MSMEG_2316	monooxygenase, NtaA/SnaA/SoxA family; this gene contains a frame shift which is not the result of sequencing error		0.33	8.67E-05	8.49	1.78E-01	2.10	1.09E-02
MSMEG_2316	monooxygenase, NtaA/SnaA/SoxA family; this gene contains a frame shift which is not the result of sequencing error		0.59	5.09E-03	16.04	1.82E-01	3.25	1.75E-02
MSMEG_2316	monooxygenase, NtaA/SnaA/SoxA family; this gene contains a frame shift which is not the result of sequencing error		0.85	3.35E-01	4.79	2.64E-01	1.24	2.82E-01
MSMEG_2317	alcohol dehydrogenase, zinc-containing		0.64	2.38E-04	1.07	5.47E-01	0.77	7.18E-03
MSMEG_2317	alcohol dehydrogenase, zinc-containing		0.61	2.77E-04	0.97	7.19E-01	1.20	2.10E-01
MSMEG_2318	GTP cyclohydrolase II		0.50	5.69E-06	0.88	1.81E-01	1.66	8.99E-02
MSMEG_2318	GTP cyclohydrolase II		0.64	2.59E-04	0.98	8.45E-01	0.92	5.23E-01
MSMEG_2319	Periplasmic binding protein		0.39	5.33E-06	0.90	3.26E-01	2.23	5.76E-02
MSMEG_2319	Periplasmic binding protein		0.42	2.37E-05	1.05	5.56E-01	1.40	1.43E-01
MSMEG_2320	cytochrome c oxidase, subunit I; this gene contains a frame shift which is not the result of sequencing error; identified by match to protein family HMM PF00115; match to protein family HMM TIGR02891	*ctaD*	0.34	3.53E-07	0.82	6.51E-02	4.11	3.43E-04
MSMEG_2320	cytochrome c oxidase, subunit I; this gene contains a frame shift which is not the result of sequencing error; identified by match to protein family HMM PF00115; match to protein family HMM TIGR02891	*ctaD*	0.50	7.90E-05	0.99	8.58E-01	2.94	6.77E-03
MSMEG_2321	phosphoserine phosphatase	*serB*	0.28	5.89E-06	1.06	2.72E-01	8.94	2.01E-02
MSMEG_2321	phosphoserine phosphatase	*serB*	0.29	6.81E-06	1.00	9.27E-01	6.17	2.30E-02

MSMEG_2322	hypothetical protein		0.91	1.67E-01	0.76	2.60E-02	0.89	1.32E-01
MSMEG_2322	hypothetical protein		1.04	5.53E-01	0.86	1.39E-01	1.12	3.38E-01
MSMEG_2323	GntR-family protein transcriptional regulator		1.33	2.82E-01	1.10	3.99E-01	1.29	1.60E-01
MSMEG_2324	hypothetical protein		0.99	9.25E-01	0.97	8.15E-01	1.20	3.11E-01
MSMEG_2325	integral membrane protein		1.24	6.00E-02	1.25	1.54E-01	1.26	1.19E-01
MSMEG_2325	integral membrane protein		0.95	4.78E-01	0.92	1.86E-01	1.03	8.98E-01
MSMEG_2326	ABC-type molybdenum transport system, ATPase component		0.51	9.04E-04	0.77	8.99E-02	1.91	1.67E-01
MSMEG_2326	ABC-type molybdenum transport system, ATPase component		0.69	6.62E-03	0.97	6.90E-01	1.11	6.38E-01
MSMEG_2327	nudix hydrolase		0.52	4.24E-03	1.09	4.47E-01	1.55	1.76E-01
MSMEG_2327	nudix hydrolase		0.56	5.97E-03	0.97	7.20E-01	1.14	6.53E-01
MSMEG_2328	probable enoyl-CoA hydratase		0.57	8.44E-04	1.12	2.21E-01	1.18	3.65E-01
MSMEG_2328	probable enoyl-CoA hydratase		0.67	3.17E-03	0.93	4.80E-01	1.03	6.62E-01
MSMEG_2329	methyltransferase, UbiE/COQ5 family protein		0.61	8.23E-05	0.81	3.44E-02	0.65	1.56E-03
MSMEG_2329	methyltransferase, UbiE/COQ5 family protein		0.74	3.99E-04	0.87	7.37E-02	0.74	1.04E-02
MSMEG_2330	SAM-dependent methyltransferase		0.36	1.36E-05	0.72	2.87E-02	0.75	1.60E-01
MSMEG_2330	SAM-dependent methyltransferase		0.49	2.34E-04	1.07	1.94E-01	1.58	2.04E-01
MSMEG_2331	immunogenic protein MPB64/MPT64		1.23	5.74E-02	0.93	7.99E-03	0.55	1.44E-05
MSMEG_2331	immunogenic protein MPB64/MPT64		1.04	5.33E-01	0.84	2.00E-02	0.68	6.07E-04
MSMEG_2332	amino acid carrier protein		0.68	1.01E-01	1.38	1.16E-01	0.16	2.16E-07
MSMEG_2332	amino acid carrier protein		0.74	1.62E-01	1.23	2.17E-01	0.24	4.41E-06
MSMEG_2333	conserved hypothetical protein		0.52	1.41E-05	0.80	4.54E-02	1.46	2.17E-01
MSMEG_2334	hypothetical protein		0.75	5.48E-02	1.17	2.89E-01	1.03	7.59E-01
MSMEG_2335	hexapeptide transferase family protein		0.48	1.34E-04	1.15	1.75E-01	0.86	2.98E-01
MSMEG_2336	conserved hypothetical protein; this gene contains a frame shift which is not the result of sequencing error		1.79	8.93E-02	1.27	9.81E-03	0.72	1.26E-02
MSMEG_2336	conserved hypothetical protein; this gene contains a frame shift which is not the result of sequencing error		1.57	1.85E-01	0.96	6.62E-01	0.94	1.69E-01
MSMEG_2337	isopentenyl-diphosphate delta-isomerase, type 2	*fni*	1.88	1.04E-01	1.99	2.92E-02	0.40	4.38E-04
MSMEG_2337	isopentenyl-diphosphate delta-isomerase, type 2	*fni*	1.57	1.52E-01	1.70	4.85E-02	0.52	6.05E-03
MSMEG_2340	hypothetical protein		1.05	4.81E-01	1.02	9.23E-01	0.98	3.99E-01
MSMEG_2341	conserved hypothetical protein		1.02	9.09E-01	1.16	1.96E-01	1.19	2.13E-01
MSMEG_2342	putative glycosyltransferase		1.08	3.72E-01	1.01	8.44E-01	1.08	3.25E-01
MSMEG_2343	methylesterase		1.45	5.24E-01	1.92	1.67E-02	0.63	1.21E-01
MSMEG_2344	dehydrogenase		1.59	3.30E-01	2.62	1.45E-02	0.53	5.86E-02

MSMEG_2345	lycopene cyclase; this gene contains a frame shift which is not the result of sequencing error		1.32	4.02E-01	2.80	3.81E-02	0.58	5.00E-02
MSMEG_2346	phytoene synthase		1.88	3.19E-01	2.93	1.92E-02	0.46	3.08E-03
MSMEG_2347	phytoene dehydrogenase		1.63	9.84E-02	1.51	8.83E-02	0.50	4.75E-04
MSMEG_2348	glycosyl transferase, group 1 family protein; this gene contains a frame shift which is not the result of sequencing error; identified by match to protein family HMM PF00534		0.38	2.77E-05	0.80	5.24E-02	1.53	1.33E-02
MSMEG_2348	glycosyl transferase, group 1 family protein; this gene contains a frame shift which is not the result of sequencing error; identified by match to protein family HMM PF00534		0.51	1.59E-03	1.16	3.72E-01	1.00	9.80E-01
MSMEG_2349	glycosyl hydrolase, family protein 57		0.44	6.19E-05	1.01	8.87E-01	1.61	1.72E-01
MSMEG_2350	conserved hypothetical protein		0.71	6.11E-04	0.95	6.84E-01	0.96	5.15E-01
MSMEG_2351	electron transfer flavoprotein, beta subunit	*etfB*	0.62	2.75E-04	0.94	3.06E-01	1.49	3.41E-02
MSMEG_2352	electron transfer flavoprotein, alpha subunit	*etfA*	0.56	7.13E-04	0.73	5.67E-03	0.91	5.71E-01
MSMEG_2353	secreted protein		2.46	5.91E-04	0.87	6.48E-02	1.11	5.36E-01
MSMEG_2354	oxidoreductase		1.85	1.62E-02	0.97	7.39E-01	1.25	3.16E-01
MSMEG_2355	conserved hypothetical protein		0.69	9.81E-03	1.24	2.82E-01	1.58	3.65E-01
MSMEG_2356	acyltransferase		1.04	5.09E-01	4.46	3.90E-01	1.27	2.32E-01
MSMEG_2357	cysteine desulfurase		1.44	4.52E-02	0.82	4.19E-02	2.38	4.03E-02
MSMEG_2358	tRNA (5-methylaminomethyl-2-thiouridylate)-methyltransferase	*trmU*	1.35	4.49E-02	0.76	4.20E-02	1.96	7.26E-02
MSMEG_2359	methionine synthase, vitamin-B12 independent		0.74	4.20E-03	1.02	8.92E-01	2.03	9.72E-03
MSMEG_2360	4-coumarate:CoA ligase		1.03	5.74E-02	0.91	1.85E-01	1.46	3.75E-02
MSMEG_2361	phosphoribosylglycinamide formyltransferase		0.93	4.40E-02	1.00	9.52E-01	1.17	3.65E-01
MSMEG_2362	DNA ligase, NAD-dependent	*ligA*	0.93	5.79E-01	0.77	7.99E-03	1.18	3.75E-01
MSMEG_2363	amino acid-binding ACT		0.53	2.63E-05	1.02	8.85E-01	1.08	5.70E-01
MSMEG_2364	glutamyl-tRNA(Gln) amidotransferase, C subunit	*gatC*	0.46	1.10E-04	0.91	1.76E-01	1.57	1.49E-01
MSMEG_2365	glutamyl-tRNA(Gln) amidotransferase subunit A		0.33	1.56E-05	0.94	6.34E-01	1.33	2.53E-01
MSMEG_2366	6-phosphofructokinase 1; this gene contains a frame shift which is not the result of sequencing error; identified by match to protein family HMM PF00365; match to protein family HMM TIGR02483		0.97	7.23E-01	0.85	1.06E-01	1.29	1.86E-03
MSMEG_2367	aspartyl/glutamyl-tRNA(Asn/Gln) amidotransferase subunit B		0.71	7.50E-04	0.99	8.53E-01	2.05	6.19E-03

MSMEG_2368	transcriptional regulator, TetR family protein domain protein, putative		1.49	1.82E-02	0.82	1.37E-02	0.76	1.96E-03
MSMEG_2369	LppZ protein		1.10	6.87E-03	0.86	8.02E-02	1.02	8.52E-01
MSMEG_2370	DoxX subfamily protein, putative		1.52	4.68E-03	0.84	1.45E-01	1.49	2.72E-01
MSMEG_2371	Low molecular weight protein antigen 6		0.61	6.99E-04	0.76	5.65E-02	2.35	2.41E-03
MSMEG_2372	acetolactate synthase, large subunit, biosynthetic type	*ilvB*	0.52	1.36E-03	0.96	5.89E-01	1.30	3.80E-03
MSMEG_2373	acetolactate synthase, small subunit	*ilvN*	0.77	2.06E-02	0.76	5.80E-02	0.96	5.83E-01
MSMEG_2374	ketol-acid reductoisomerase	*ilvC*	0.46	3.51E-04	0.96	5.39E-01	1.67	1.27E-01
MSMEG_2375	NAD(P)H:quinone oxidoreductase, type IV	*wrbA*	1.02	8.68E-01	1.36	1.70E-01	0.51	1.87E-02
MSMEG_2376	conserved hypothetical protein		0.92	8.48E-01	1.76	7.89E-02	0.64	9.60E-02
MSMEG_2377	P49 protein		1.08	6.32E-01	1.02	9.51E-01	0.95	4.03E-01
MSMEG_2378	D-3-phosphoglycerate dehydrogenase	*serA*	0.43	3.62E-05	1.20	7.37E-02	1.41	1.07E-01
MSMEG_2379	3-isopropylmalate dehydrogenase	*leuB*	0.45	6.38E-05	1.14	1.38E-01	1.60	5.98E-02
MSMEG_2380	sugar transporter family protein		0.60	4.78E-04	0.95	8.49E-01	1.49	2.98E-01
MSMEG_2381	conserved hypothetical protein		0.56	4.82E-03	1.07	4.00E-01	1.53	2.02E-02
MSMEG_2382	5-carboxymethyl-2-hydroxymuconate delta-isomerase		0.38	2.44E-04	0.97	6.44E-01	2.39	2.21E-01
MSMEG_2383	glutamyl-tRNA synthetase	*gltX*	0.37	3.95E-06	1.11	4.91E-02	1.48	6.36E-02
MSMEG_2386	transcriptional regulator, IclR family protein		0.60	5.01E-03	1.03	7.85E-01	1.00	8.46E-01
MSMEG_2387	3-isopropylmalate dehydratase, large subunit	*leuC*	0.81	8.93E-03	0.72	5.11E-03	1.03	7.60E-01
MSMEG_2388	3-isopropylmalate dehydratase, small subunit	*leuD*	0.60	2.15E-05	0.83	1.68E-02	1.01	9.57E-01
MSMEG_2389	DNA-binding protein HU	*hup*	2.29	1.45E-04	0.88	1.28E-01	0.54	3.51E-02
MSMEG_2390	hydrolase, NUDIX family protein		0.55	2.44E-03	1.81	2.26E-01	1.72	6.18E-03
MSMEG_2391	polyphosphate kinase	*ppk*	0.79	9.90E-02	1.09	5.19E-01	1.43	2.02E-02
MSMEG_2392	conserved hypothetical protein		0.50	5.50E-04	1.07	5.94E-01	1.30	9.29E-02
MSMEG_2393	NAD-dependent glycerol-3-phosphate dehydrogenase	*gpsA*	0.36	3.51E-05	1.38	2.04E-01	3.13	2.59E-02
MSMEG_2394	putative cystathionine gamma-synthase		0.41	9.44E-05	1.12	5.78E-01	1.98	3.38E-03
MSMEG_2395	D-alanine--D-alanine ligase		0.49	3.30E-04	0.93	1.51E-01	1.73	2.02E-01
MSMEG_2396	conserved hypothetical protein		1.40	4.64E-02	1.03	8.54E-01	1.61	4.13E-03
MSMEG_2397	transcriptional regulatory protein, AsnC family protein		0.62	1.44E-04	0.87	1.67E-01	1.53	1.61E-01
MSMEG_2398	thiamine-monophosphate kinase	*thiL*	0.80	1.60E-01	0.81	1.68E-01	1.80	2.02E-01
MSMEG_2399	uracil-DNA glycosylase	*ung*	0.54	6.35E-05	1.05	6.69E-01	1.46	1.24E-01
MSMEG_2400	ribosomal protein L28	*rpmB*	1.07	2.02E-01	0.65	1.76E-02	0.56	1.54E-03
MSMEG_2401	hypothetical protein		0.94	5.00E-01	1.04	7.48E-01	1.27	2.84E-01
MSMEG_2402	dihydroxyacetone kinase		0.80	4.58E-02	1.20	1.13E-01	2.64	2.55E-01

MSMEG_2403	ATP-dependent DNA helicase RecG	*recG*	0.84	1.37E-01	0.96	5.88E-01	1.03	5.99E-01
MSMEG_2404	extracellular deoxyribonuclease		0.54	1.11E-03	1.13	2.14E-01	0.97	8.31E-01
MSMEG_2405	MarR-family protein transcriptional regulator		1.69	7.11E-03	0.98	5.54E-01	0.78	2.31E-01
MSMEG_2406	transcriptional regulator, TetR family protein, putative		1.11	5.28E-01	1.10	3.50E-01	1.04	7.37E-01
MSMEG_2407	morphine 6-dehydrogenase		0.98	6.92E-01	0.84	5.43E-02	0.94	4.16E-01
MSMEG_2408	2,5-diketo-D-gluconic acid reductase A		0.95	8.85E-02	0.93	9.62E-02	1.33	3.50E-03
MSMEG_2409	alpha/beta hydrolase fold family protein		0.72	1.14E-02	0.99	8.39E-01	0.67	7.66E-03
MSMEG_2410	putative serine-threonine protein kinase		0.71	1.14E-03	0.77	7.15E-02	0.96	7.51E-01
MSMEG_2411	conserved integral membrane protein		0.64	6.14E-04	0.82	3.36E-02	1.73	1.33E-01
MSMEG_2412	pyruvate carboxylase	*pyc*	0.92	1.71E-01	0.72	1.62E-02	2.35	2.26E-02
MSMEG_2413	putative methyltransferase		0.33	6.89E-05	1.03	8.63E-01	2.11	1.64E-01
MSMEG_2414	pantetheine-phosphate adenylyltransferase	*coaD*	0.86	2.80E-01	1.16	5.41E-01	1.20	2.87E-01
MSMEG_2415	hemerythrin HHE cation binding region		1.41	1.71E-01	2.42	6.98E-03	0.23	2.18E-05
MSMEG_2416	conserved hypothetical protein		1.44	1.75E-01	0.90	4.98E-01	1.28	4.61E-01
MSMEG_2417	conserved hypothetical protein		0.86	1.18E-02	0.84	1.02E-03	1.44	8.18E-02
MSMEG_2418	ribonuclease III	*rnc*	1.17	1.72E-02	0.97	6.48E-01	1.65	2.93E-04
MSMEG_2419	formamidopyrimidine-DNA glycosylase	*mutM*	0.43	4.85E-04	0.97	5.82E-01	3.32	1.92E-02
MSMEG_2420	hypothetical protein		1.73	3.56E-04	0.88	1.16E-01	0.59	2.11E-03
MSMEG_2421	conserved hypothetical protein		0.72	1.65E-02	1.16	3.72E-01	1.06	7.59E-01
MSMEG_2422	acylphosphatase		2.07	1.01E-03	0.83	2.49E-02	0.80	1.09E-02
MSMEG_2423	chromosome segregation protein SMC	*smc*	0.85	3.58E-01	1.19	7.16E-02	2.69	3.24E-03
MSMEG_2424	signal recognition particle-docking protein FtsY	*ftsY*	0.56	5.78E-05	1.10	2.54E-01	1.17	8.72E-02
MSMEG_2425	ammonium transporter	*amt*	0.12	9.64E-06	3.66	2.73E-01	0.41	4.89E-03
MSMEG_2426	nitrogen regulatory protein P-II		0.12	9.20E-06	2.47	2.64E-01	0.42	2.86E-03
MSMEG_2427	protein-P-II uridylyltransferase	*glnD*	0.23	1.07E-04	1.51	2.21E-01	0.95	3.28E-01
MSMEG_2428	DNA-binding protein		2.77	1.02E-02	0.79	3.26E-02	0.98	8.03E-01
MSMEG_2429	aldo/keto reductase		1.97	7.86E-03	1.11	4.54E-01	1.12	2.54E-01
MSMEG_2430	signal recognition particle protein	*ffh*	0.84	1.15E-01	0.58	1.08E-02	1.18	4.63E-01
MSMEG_2431	amidohydrolase 3		0.51	1.33E-05	1.02	4.98E-01	1.62	9.11E-02
MSMEG_2432	D-alanyl-D-alanine carboxypeptidase		0.91	6.80E-01	0.84	1.65E-01	1.20	3.98E-01
MSMEG_2433	D-alanyl-D-alanine carboxypeptidase		0.50	6.73E-04	0.97	8.52E-01	0.82	2.23E-01
MSMEG_2434	conserved hypothetical protein		0.57	8.89E-04	0.85	1.10E-01	1.10	5.62E-01
MSMEG_2435	30S ribosomal protein S16		0.61	4.17E-04	1.02	8.39E-01	1.49	9.04E-02
MSMEG_2436	conserved hypothetical protein		0.57	2.42E-05	0.54	4.71E-03	0.99	9.51E-01
MSMEG_2437	16S rRNA processing protein RimM	*rimM*	0.18	1.34E-06	0.79	1.80E-02	1.57	1.20E-01
MSMEG_2438	tRNA (guanine-N1)-methyltransferase	*trmD*	0.36	2.03E-05	1.37	3.63E-01	1.68	2.09E-01
MSMEG_2439	LppW protein		0.72	3.24E-03	1.12	4.16E-01	1.25	3.10E-01

MSMEG_2440	ribosomal protein L19	*rplS*	0.77	1.71E-03	0.78	1.26E-02	0.90	5.04E-01
MSMEG_2441	signal peptidase I	*lepB*	0.47	8.78E-05	0.90	4.50E-01	1.51	4.03E-02
MSMEG_2442	ribonuclease HII		1.11	7.89E-02	0.88	1.16E-01	2.67	1.01E-03
MSMEG_2443	conserved hypothetical protein		1.20	1.86E-02	0.94	5.02E-01	1.48	9.59E-02
MSMEG_2444	dienelactone hydrolase family protein		1.56	1.33E-03	0.79	2.13E-02	0.73	1.65E-02
MSMEG_2445	conserved hypothetical protein		1.91	1.01E-01	0.89	3.68E-01	1.30	1.39E-01
MSMEG_2446	conserved hypothetical protein		1.96	5.28E-04	0.85	1.52E-01	0.81	1.79E-01
MSMEG_2447	regulatory protein		2.03	2.82E-03	0.79	2.35E-01	1.76	7.45E-02
MSMEG_2448	hypothetical protein		2.01	2.34E-02	0.72	5.43E-02	3.27	3.47E-01
MSMEG_2449	methylmalonate-semialdehyde dehydrogenase	*mmsA*	2.27	2.07E-03	0.86	8.91E-02	0.97	7.38E-01
MSMEG_2450	adenosylmethionine--8-amino-7-oxononanoate transaminase		1.59	1.95E-03	1.10	5.11E-01	1.25	8.61E-02
MSMEG_2451	HAD-superfamily protein hydrolase, subfamily protein IIA		0.93	4.83E-01	1.07	3.30E-01	3.45	2.72E-01
MSMEG_2452	hypothetical protein		0.74	1.51E-02	0.80	2.30E-01	1.26	4.14E-02
MSMEG_2453	transcriptional regulator, GntR family protein		1.18	3.34E-01	0.87	2.91E-01	1.31	3.60E-02
MSMEG_2454	translation initiation inhibitor		0.97	6.62E-01	0.90	5.30E-01	1.49	8.54E-02
MSMEG_2455	carbon monoxide dehydrogenase subunit G (CoxG) superfamily protein		1.05	4.10E-01	0.89	3.33E-01	1.22	4.41E-01
MSMEG_2456	5,10-methylenetetrahydromethanopterin		2.28	1.01E-03	0.79	2.26E-01	1.01	9.08E-01
MSMEG_2457	hypothetical protein		1.74	1.04E-02	0.79	2.82E-01	1.08	4.91E-01
MSMEG_2458	hypothetical protein		1.56	5.65E-03	0.79	1.10E-01	0.91	5.50E-01
MSMEG_2459	transporter ATPase		1.66	1.61E-03	0.72	5.99E-03	1.02	8.21E-01
MSMEG_2460	transporter permease 1		2.02	2.53E-01	0.76	4.33E-03	1.13	4.07E-01
MSMEG_2461	transporter permease 2, putative		1.11	3.96E-01	0.82	1.12E-01	1.52	4.59E-02
MSMEG_2462	carbon-monoxide dehydrogenase		0.96	5.11E-01	0.92	3.82E-01	1.68	9.61E-03
MSMEG_2463	nicotine dehydrogenase chain A		1.38	7.76E-02	0.87	4.99E-01	1.93	1.04E-02
MSMEG_2464	[2Fe-2S] binding domain protein		1.14	2.02E-01	1.02	7.75E-01	1.56	4.43E-02
MSMEG_2465	adenine deaminase		1.15	6.72E-01	0.97	7.93E-01	1.20	3.40E-02
MSMEG_2466	glutaryl-CoA dehydrogenase		0.99	8.12E-01	0.94	4.31E-01	2.25	2.91E-02
MSMEG_2467	transketolase, central region		1.32	1.62E-01	0.71	1.47E-02	1.40	1.91E-01
MSMEG_2468	L-carnitine dehydratase/bile acid-inducible protein F		0.43	2.25E-05	1.31	3.46E-02	1.39	1.92E-01
MSMEG_2469	AMP-dependent synthetase and ligase		0.51	9.33E-05	0.94	5.00E-01	1.10	3.95E-01
MSMEG_2470	acyl-CoA thioesterase		0.44	1.35E-02	1.16	4.94E-01	1.20	1.30E-01
MSMEG_2471	pyruvate dehydrogenase alpha subunit		3.27	4.16E-03	0.91	6.64E-01	1.33	1.89E-01
MSMEG_2472	AsnC-family protein transcriptional regulator		0.95	7.08E-01	0.81	1.84E-01	1.94	2.49E-01

MSMEG_2473	sugar transporter; this gene contains a frame shift which is not the result of sequencing error	1.24	2.24E-01	1.22	2.99E-02	1.33	1.10E-01
MSMEG_2473	sugar transporter; this gene contains a frame shift which is not the result of sequencing error	0.98	9.24E-01	0.95	8.56E-02	1.02	7.13E-01
MSMEG 2474	probable cutinase Cut3	1.01	9.31E-01	1.06	6.64E-01	0.95	2.47E-01
MSMEG 2475	putative oxidoreductase YdbC	0.49	2.17E-02	1.01	9.33E-01	0.99	9.14E-01
MSMEG_2476	MarR family protein transcriptional regulatory protein	0.89	2.87E-01	0.84	1.12E-01	1.10	4.07E-01
MSMEG_2477	cyclopentanol dehydrogenase	0.84	2.16E-01	1.01	9.61E-01	1.35	4.97E-01
MSMEG 2478	fumarylacetoacetate hydrolase family protein	1.27	1.66E-02	0.93	4.35E-01	1.68	2.21E-02
MSMEG 2479	hypothetical protein	1.18	6.47E-01	1.05	6.11E-01	0.85	2.58E-01
MSMEG_2480	transcriptional regulator, GntR family protein, putative	0.84	1.46E-01	1.00	9.90E-01	0.80	5.52E-02
MSMEG_2481	conserved hypothetical protein	0.73	7.36E-03	1.03	7.27E-01	0.82	1.36E-01
MSMEG 2482	hypothetical protein	1.42	4.35E-02	0.98	6.20E-01	0.91	3.59E-01
MSMEG 2483	transcriptional regulator, IclR family protein	1.35	7.54E-02	1.07	5.05E-01	0.89	2.02E-01
MSMEG_2484	conserved hypothetical protein	1.26	5.35E-02	0.88	6.07E-02	2.02	2.31E-02
MSMEG 2485	monooxygenase, FAD-binding	1.31	4.35E-01	0.96	4.87E-01	1.24	1.38E-01
MSMEG 2486	major facilitator superfamily protein	2.77	3.18E-02	0.74	1.50E-01	1.32	3.12E-01
MSMEG 2487	4-aminobutyrate aminotransferase	1.94	1.85E-02	1.01	9.33E-01	0.95	1.65E-01
MSMEG_2488	[NADP+] succinate-semialdehyde dehydrogenase	0.87	1.68E-01	0.69	1.32E-02	1.33	2.78E-01
MSMEG_2489	transcriptional regulator, GntR family protein, putative	1.31	1.74E-02	0.90	3.18E-01	1.11	4.22E-01
MSMEG 2490	decarboxylase	1.27	2.11E-02	0.84	1.30E-01	1.46	2.96E-02
MSMEG_2491	acetylornithine deacetylase	1.23	2.52E-01	1.03	5.75E-01	1.25	1.19E-01
MSMEG_2492	D-lactate dehydrogenase	0.85	3.17E-02	1.20	8.78E-02	2.21	2.00E-01
MSMEG 2493	aminotransferase, class I and II family protein	0.94	6.35E-01	1.16	2.78E-01	1.17	5.58E-01
MSMEG 2494	Xaa-Pro aminopeptidase	0.98	8.47E-01	1.27	8.88E-02	1.08	7.46E-01
MSMEG_2495	decarboxylase	1.20	8.37E-02	1.11	3.93E-01	1.37	3.42E-01
MSMEG_2496	NAD-binding protein	0.89	1.83E-01	1.05	3.03E-01	1.34	3.42E-01
MSMEG 2497	amidase family protein	1.06	8.97E-01	1.40	4.96E-01	1.40	5.72E-01
MSMEG 2498	conserved hypothetical protein	0.71	1.11E-03	0.94	5.11E-01	0.95	7.76E-01
MSMEG_2499	ABC transporter, membrane spanning protein, putative	1.06	4.31E-01	1.06	9.18E-02	0.85	3.99E-01
MSMEG_2500	conserved hypothetical protein	0.39	2.67E-04	0.79	3.15E-01	1.26	1.85E-01
MSMEG_2501	ABC nitrate/sulfonate/bicarbonate family protein transporter, inner membrane subunit	1.31	1.47E-02	0.95	6.87E-01	0.95	6.98E-01
MSMEG 2502	transporter ATPase	0.95	2.61E-01	0.89	4.77E-02	0.74	1.08E-01

MSMEG_2503	hypothetical protein		0.94	1.36E-01	0.96	3.86E-01	0.80	2.30E-01
MSMEG_2504	3-isopropylmalate dehydratase small subunit		1.07	4.39E-01	0.82	8.23E-02	0.78	7.44E-02
MSMEG_2505	3-isopropylmalate dehydratase large subunit		0.97	8.05E-01	1.06	2.89E-01	0.87	3.73E-01
MSMEG_2506	carboxyvinyl-carboxyphosphonate phosphorylmutase		1.34	1.41E-01	0.99	9.29E-01	0.65	4.70E-02
MSMEG_2507	IclR-family protein transcriptional regulator		2.02	5.44E-03	0.94	6.68E-01	0.79	1.25E-01
MSMEG_2508	conserved hypothetical protein		1.11	2.57E-01	1.15	2.24E-01	1.07	3.07E-01
MSMEG_2509	Mg-chelatase subunits D/I family protein, ComM subfamily protein		1.30	2.15E-01	1.08	5.18E-01	0.91	4.09E-01
MSMEG_2510	smf family protein; this gene contains a frame shift which is not the result of sequencing error; identified by match to protein family HMM PF02481		1.29	1.57E-01	1.93	1.87E-01	1.97	2.94E-01
MSMEG_2510	smf family protein; this gene contains a frame shift which is not the result of sequencing error; identified by match to protein family HMM PF02481		1.03	7.30E-01	1.19	2.74E-01	0.95	2.99E-01
MSMEG_2511	siderophore utilization protein		0.75	2.43E-02	0.80	5.29E-02	0.76	2.87E-01
MSMEG_2512	lactate 2-monooxygenase		0.48	5.23E-05	1.14	2.58E-01	1.20	1.41E-01
MSMEG_2513	hypothetical protein		13.27	5.19E-02	0.25	1.27E-02	0.69	2.31E-01
MSMEG_2514	conserved hypothetical protein; this gene contains a frame shift which is not the result of sequencing error		2.27	7.75E-02	0.85	2.15E-01	1.44	3.90E-01
MSMEG_2514	conserved hypothetical protein; this gene contains a frame shift which is not the result of sequencing error		1.60	3.21E-01	1.03	8.64E-01	1.01	9.22E-01
MSMEG_2515	tyrosine recombinase XerC	*xerC*	1.36	5.38E-01	0.97	7.09E-01	1.80	4.52E-02
MSMEG_2516	putative oxidoreductase		0.79	6.22E-02	0.78	3.88E-02	1.47	2.68E-01
MSMEG_2517	hydrolase, alpha/beta fold family protein, putative		0.74	1.47E-01	0.91	1.46E-01	1.53	7.95E-02
MSMEG_2518	peptidase M23B		1.45	2.61E-02	1.19	1.40E-01	1.38	1.57E-01
MSMEG_2519	ribosomal protein S2	*rpsB*	0.43	5.65E-06	1.10	3.07E-01	1.44	2.80E-02
MSMEG_2520	translation elongation factor Ts	*tsf*	0.29	9.64E-08	1.09	2.71E-01	1.73	4.00E-03
MSMEG_2521	amidase		0.54	1.49E-02	0.98	8.97E-01	3.48	1.65E-01
MSMEG_2522	efflux ABC transporter, permease protein		1.13	6.22E-01	1.47	2.53E-02	0.30	4.51E-03
MSMEG_2523	efflux ABC transporter, permease protein,		0.27	1.88E-03	0.85	2.92E-01	0.99	9.76E-01
MSMEG_2524	ABC transporter, ATP-binding protein		0.10	9.02E-06	1.12	5.99E-01	0.72	1.29E-01
MSMEG_2525	amino acid permease superfamily protein		0.32	1.86E-02	5.21	2.94E-01	0.75	2.29E-01
MSMEG_2526	copper methylamine oxidase		0.15	5.03E-05	3.60	3.41E-01	0.65	1.08E-01

MSMEG_2527	hypothetical protein		2.03	1.43E-02	1.41	4.14E-01	1.05	7.79E-01
MSMEG_2528	glycerate kinase		0.82	1.32E-02	0.82	5.69E-02	0.94	6.74E-01
MSMEG_2529	glyoxylate reductase		0.63	8.25E-05	0.98	6.81E-01	1.06	6.56E-01
MSMEG_2530	4-carboxymuconolactone decarboxylase domain protein	*pcaC*	0.64	6.08E-05	1.07	3.03E-01	1.29	7.97E-02
MSMEG_2531	GntR family protein transcriptional regulator		0.76	2.49E-02	1.33	2.79E-01	0.96	5.28E-01
MSMEG_2532	dehydroquinase dehydratase, type II	*aroQ*	1.23	6.26E-03	0.78	2.07E-02	0.47	2.09E-04
MSMEG_2533	hypothetical protein		0.98	8.60E-01	0.95	5.23E-01	0.78	2.25E-01
MSMEG_2534	putative carboxylesterase protein		0.90	1.39E-01	1.01	9.52E-01	0.65	3.12E-02
MSMEG_2535	dehydrogenase/reductase SDR family protein member 10		0.68	9.96E-04	0.97	6.05E-01	0.70	8.52E-02
MSMEG_2536	3-oxoacyl-[acyl-carrier-protein] reductase		0.93	2.94E-01	0.91	1.46E-01	0.52	4.80E-02
MSMEG_2537	transporter protein		0.78	3.80E-02	0.93	2.52E-01	0.66	5.72E-02
MSMEG_2538	MarR-family protein transcriptional regulator		0.54	1.69E-03	1.03	6.23E-01	1.23	2.64E-01
MSMEG_2539	thiopurine S-methyltransferase (tpmt) superfamily protein		1.66	3.71E-02	1.01	6.86E-01	0.46	2.33E-03
MSMEG_2540	uridylate kinase	*pyrH*	0.68	5.43E-04	0.85	2.86E-02	1.31	4.62E-02
MSMEG_2541	ribosome recycling factor	*frr*	0.68	2.73E-03	0.91	1.29E-01	1.23	4.07E-02
MSMEG_2542	integral membrane protein DUF6		0.76	1.15E-02	1.86	2.80E-01	1.16	4.14E-01
MSMEG_2543	phosphatidate cytidylyltransferase	*cdsA*	0.54	5.94E-06	0.96	3.72E-01	1.94	8.21E-02
MSMEG_2544	transcriptional regulator, LysR family protein		0.99	9.05E-01	0.86	5.70E-02	0.95	8.26E-01
MSMEG_2545	radical SAM enzyme, Cfr family protein		1.09	3.98E-01	0.80	6.94E-02	1.28	1.66E-02
MSMEG_2546	transcriptional Regulator, GntR family protein		0.58	6.13E-05	1.08	2.97E-01	1.09	3.14E-01
MSMEG_2547	transcriptional regulator		1.66	4.66E-01	1.13	2.35E-01	1.29	2.24E-01
MSMEG_2548	3-oxoacyl-[acyl-carrier-protein] reductase		1.20	1.23E-01	0.96	8.01E-01	0.87	1.08E-01
MSMEG_2549	major facilitator superfamily protein		1.48	1.51E-01	0.98	8.39E-01	1.10	5.64E-01
MSMEG_2550	glyoxylate reductase		1.46	1.61E-01	0.99	9.31E-01	1.33	5.11E-01
MSMEG_2551	hypothetical protein		1.05	7.96E-01	0.93	1.38E-01	1.01	8.89E-01
MSMEG_2552	aldehyde dehydrogenase (NAD) family protein		0.93	5.92E-01	0.85	1.35E-01	1.03	2.37E-01
MSMEG_2553	transcriptional regulator, TetR family protein		1.30	3.42E-02	0.91	1.85E-01	1.08	5.64E-01
MSMEG_2554	phosphotransferase enzyme family protein		0.60	4.35E-03	1.25	3.16E-01	1.03	1.59E-01
MSMEG_2555	hypothetical protein		0.96	6.91E-01	1.17	6.06E-01	1.08	4.96E-01
MSMEG_2556	beta-glucosidase		1.61	1.09E-01	0.83	1.38E-01	1.27	8.42E-02
MSMEG_2557	transcriptional regulator, TetR family protein		0.98	4.01E-01	1.28	1.89E-01	1.10	4.07E-01
MSMEG_2558	NikQ protein		2.30	2.09E-02	1.20	8.84E-02	1.67	8.38E-02
MSMEG_2559	conserved domain protein		1.22	1.51E-01	1.07	3.22E-01	0.97	4.59E-01
MSMEG_2560	conserved hypothetical protein		1.20	5.49E-01	1.38	3.20E-01	1.60	3.87E-01
MSMEG_2561	gluconolactonase		1.06	6.67E-01	1.14	3.13E-01	1.17	2.11E-01

MSMEG_2562	conserved hypothetical protein		1.21	4.95E-01	1.40	1.82E-01	1.35	3.80E-01
MSMEG_2563	cytochrome P450 superfamily protein		1.67	6.35E-03	0.85	1.77E-01	1.40	1.66E-01
MSMEG_2564	oxidoreductase, zinc-binding dehydrogenase family protein		1.01	8.86E-01	1.14	2.36E-01	1.13	1.97E-01
MSMEG_2565	4-hydroxy-2-oxovalerate aldolase		1.21	6.05E-01	1.02	7.57E-01	0.82	1.89E-01
MSMEG_2566	3-alpha-(or 20-beta)-hydroxysteroid dehydrogenase		0.95	2.82E-01	0.88	9.14E-02	0.98	8.78E-01
MSMEG_2567	probable monooxygenase		1.18	2.92E-01	1.04	7.42E-01	1.26	5.25E-01
MSMEG_2568	LipW protein		1.12	1.59E-01	0.95	4.80E-01	1.29	4.39E-01
MSMEG_2569	oxidoreductase, 2OG-Fe(II) oxygenase family protein		0.55	2.91E-02	1.15	5.70E-01	1.09	3.96E-01
MSMEG_2570	xanthine/uracil permease		0.63	1.58E-01	0.85	1.89E-01	1.18	5.73E-01
MSMEG_2571	cupin 2, conserved barrel		0.91	1.73E-01	0.98	6.08E-01	0.96	8.40E-01
MSMEG_2572	conserved hypothetical protein		1.29	1.98E-02	0.98	7.41E-01	1.02	8.28E-01
MSMEG_2573	conserved hypothetical protein		1.30	1.95E-01	1.16	9.10E-03	0.94	3.81E-01
MSMEG_2574	conserved hypothetical protein		1.17	2.21E-01	0.97	8.36E-01	1.03	7.84E-01
MSMEG_2575	conserved hypothetical protein		0.69	4.24E-03	0.69	3.99E-02	1.55	2.57E-02
MSMEG_2576	deoxyribodipyrimidine photo-lyase		1.49	2.84E-02	1.15	3.28E-01	1.39	1.78E-01
MSMEG_2577	hypothetical protein		1.02	6.69E-01	0.98	8.06E-01	1.47	1.81E-01
MSMEG_2578	1-deoxy-D-xylulose 5-phosphate reductoisomerase	*dxr*	1.22	1.74E-02	0.84	3.78E-02	2.09	7.29E-04
MSMEG_2579	zinc metalloprotease		0.87	6.35E-03	0.81	2.49E-02	1.87	9.19E-02
MSMEG_2580	4-hydroxy-3-methylbut-2-en-1-yl diphosphate synthase	*ispG*	1.28	2.52E-03	0.79	2.17E-02	1.64	6.04E-02
MSMEG_2581	acetyltransferase		1.75	7.11E-04	0.74	2.16E-02	0.96	8.22E-01
MSMEG_2582	conserved hypothetical protein		2.13	3.65E-03	0.69	2.73E-03	1.27	7.46E-01
MSMEG_2583	putative transcriptional regulator		1.31	1.63E-01	0.97	8.19E-01	1.13	1.32E-01
MSMEG_2584	penicillin-binding protein, putative		1.16	4.46E-01	1.14	4.32E-01	3.47	6.04E-02
MSMEG_2585	conserved hypothetical protein		0.88	7.14E-02	0.82	1.45E-02	1.82	2.84E-02
MSMEG_2586	conserved hypothetical protein, putative		0.78	3.87E-02	1.13	1.08E-01	0.79	1.97E-01
MSMEG_2587	methionine aminopeptidase, type I	*map*	1.31	2.37E-04	0.76	4.54E-02	0.94	3.00E-01
MSMEG_2588	cobyric acid synthase CobQ	*cobQ*	0.62	1.05E-04	0.87	2.61E-01	2.27	7.16E-02
MSMEG_2589	conserved hypothetical protein		0.95	7.71E-01	1.24	1.20E-01	0.97	6.92E-01
MSMEG_2590	AraC-family protein transcriptional regulator		2.54	6.04E-04	1.03	8.26E-01	1.66	7.22E-03
MSMEG_2591	hypothetical protein		2.80	3.84E-04	0.80	1.24E-01	1.60	4.12E-02
MSMEG_2592	conserved hypothetical protein		3.28	1.73E-02	0.77	1.42E-01	0.20	2.97E-08
MSMEG_2593	gnat-family protein acetyltransferase		1.48	5.95E-02	1.19	2.54E-01	1.08	5.58E-01
MSMEG_2594	asparagine synthase (glutamine-hydrolyzing)	*asnB*	2.07	1.34E-02	0.89	3.95E-01	0.68	3.54E-03

MSMEG_2595	gamma-glutamylisopropylamide synthetase		2.49	6.71E-02	1.06	3.65E-01	2.12	8.26E-02
MSMEG_2596	peptidase C26		1.96	1.00E-03	0.91	2.57E-01	3.44	9.99E-03
MSMEG_2597	aldehyde dehydrogenase		1.48	1.02E-02	0.83	3.54E-02	2.52	6.69E-02
MSMEG_2598	short chain dehydrogenase		1.93	1.59E-03	0.77	3.85E-02	2.34	1.37E-02
MSMEG_2599	GntR-family protein transcriptional regulator		1.29	3.31E-02	0.80	1.38E-01	2.96	1.89E-03
MSMEG_2600	regulatory protein		1.37	8.40E-04	0.94	2.31E-01	1.70	8.18E-04
MSMEG_2601	protocatechuate 3,4-dioxygenase, beta subunit	*pcaH*	6.68	1.44E-03	0.69	1.06E-03	0.10	1.20E-05
MSMEG_2602	protocatechuate 3,4-dioxygenase, alpha subunit	*pcaG*	6.22	3.29E-02	0.75	4.82E-04	0.19	5.04E-05
MSMEG_2603	3-carboxy-cis,cis-muconate cycloisomerase	*pcaB*	4.07	4.94E-03	0.79	8.77E-02	0.20	1.59E-04
MSMEG_2604	4-carboxymuconolactone decarboxylase	*pcaC*	3.92	2.35E-03	0.75	4.05E-02	0.42	4.58E-03
MSMEG_2605	transcriptional regulator, GntR family protein		1.00	9.72E-01	0.97	7.17E-01	1.19	3.21E-01
MSMEG_2606	ArsR-family protein transcriptional regulator		1.33	7.83E-02	0.89	1.73E-01	0.96	3.40E-01
MSMEG_2607	cobalamin biosynthesis protein CbiM	*cbiM*	0.70	1.22E-02	1.50	1.69E-01	0.91	6.61E-01
MSMEG_2608	cobalt transport protein CbiM		0.86	1.17E-01	1.68	6.32E-02	0.75	1.35E-01
MSMEG_2609	cobalt ABC transporter, permease protein CbiQ	*cbiQ*	0.89	2.13E-01	1.87	1.29E-02	1.35	2.93E-03
MSMEG_2610	cobalt transport protein ATP-binding subunit		0.86	6.98E-02	1.59	3.71E-02	1.55	3.47E-02
MSMEG_2611	pyridine nucleotide-disulphide oxidoreductase family protein		1.12	1.52E-01	0.91	2.74E-01	1.67	9.32E-03
MSMEG_2612	conserved hypothetical protein		0.79	3.68E-03	0.96	3.33E-01	2.84	5.74E-03
MSMEG_2613	malate:quinone-oxidoreductase	*mqo*	0.77	1.58E-03	0.92	1.32E-02	3.67	1.73E-04
MSMEG_2614	ElaA protein		0.43	1.08E-06	1.01	8.72E-01	6.50	4.22E-04
MSMEG_2615	chelatase		0.43	3.74E-06	1.06	4.78E-01	7.27	5.78E-04
MSMEG_2616	cob(I)alamin adenosyltransferase	*cobO*	0.51	4.41E-04	1.10	4.52E-01	7.26	1.40E-02
MSMEG_2617	cobyrinic Acid a,c-diamide synthase	*cobB*	0.41	4.96E-06	1.19	7.98E-03	12.77	3.05E-02
MSMEG_2618	uroporphyrin-III C-methyltransferase	*cobA*	0.47	2.27E-04	1.00	9.00E-01	2.32	1.47E-01
MSMEG_2619	efflux protein		1.74	8.85E-03	0.57	4.22E-03	1.15	2.57E-01
MSMEG_2620	hypothetical protein		1.01	8.66E-01	0.75	2.78E-02	0.95	6.13E-01
MSMEG_2621	prolyl-tRNA synthetase	*proS*	0.60	1.21E-04	0.95	4.80E-01	1.35	1.36E-01
MSMEG_2622	conserved hypothetical alanine rich protein		0.78	2.96E-02	0.94	7.47E-01	0.92	2.92E-01
MSMEG_2623	Tat (twin-arginine translocation) pathway signal sequence domain protein		0.64	2.88E-02	1.00	9.72E-01	1.23	8.13E-02
MSMEG_2624	conserved hypothetical protein		1.20	1.50E-02	0.99	9.27E-01	1.74	1.21E-04
MSMEG_2625	transcription termination factor NusA	*nusA*	1.10	6.87E-02	0.94	2.37E-01	2.02	1.23E-02
MSMEG_2626	conserved hypothetical protein		1.53	1.30E-03	0.80	2.83E-02	0.55	1.51E-02
MSMEG_2627	hypothetical protein		1.55	1.49E-01	1.05	7.10E-01	1.40	1.55E-01
MSMEG_2628	translation initiation factor IF-2	*infB*	1.15	1.39E-02	0.67	1.52E-03	1.23	6.30E-03
MSMEG_2629	ribosome-binding factor A	*rbfA*	0.83	1.00E-02	0.67	2.31E-03	1.51	2.06E-02
MSMEG_2630	DHH family protein		0.74	7.35E-03	0.84	2.29E-01	2.23	1.59E-02

MSMEG_2631	MATE efflux family protein		0.83	7.69E-02	0.94	5.47E-01	1.77	1.60E-03
MSMEG_2632	SAM-dependent methyltransferase		2.22	2.20E-03	0.72	5.29E-02	1.21	1.51E-01
MSMEG_2633	ABC transporter ATP-binding protein		3.54	1.30E-02	0.72	4.21E-02	0.76	1.88E-01
MSMEG_2634	conserved hypothetical protein		2.97	7.16E-02	0.73	3.10E-02	0.13	8.32E-05
MSMEG_2635	ABC transporter permease protein		4.11	1.89E-02	0.72	7.39E-02	0.42	2.34E-02
MSMEG_2636	regulatory protein, LacI		0.93	5.26E-01	1.16	4.98E-01	1.71	3.14E-01
MSMEG_2637	oxidoreductase, Gfo/Idh/MocA family protein		1.14	2.27E-01	1.04	7.37E-01	0.95	5.51E-01
MSMEG_2638	conserved hypothetical protein		1.41	2.08E-01	1.28	5.99E-01	1.31	1.32E-01
MSMEG_2639	integral membrane protein		0.42	7.24E-05	0.81	1.40E-01	1.05	1.72E-01
MSMEG_2640	3-dehydroshikimate dehydratase		1.23	2.52E-01	0.90	1.38E-01	1.11	4.79E-01
MSMEG_2641	enoyl-CoA hydratase		0.42	1.88E-04	0.91	3.20E-01	1.48	3.18E-02
MSMEG_2642	conserved hypothetical protein		2.11	1.56E-03	0.96	6.29E-01	1.29	3.28E-01
MSMEG_2643	conserved hypothetical protein		1.25	1.40E-01	0.75	5.95E-02	0.91	2.18E-01
MSMEG_2644	putative hydrolase		0.91	3.30E-01	0.74	1.75E-02	2.41	7.58E-02
MSMEG_2645	conserved hypothetical protein		0.98	3.79E-01	1.18	2.95E-02	0.41	6.31E-05
MSMEG_2647	metallophosphoesterase		0.53	6.56E-06	1.16	7.74E-03	1.80	8.77E-02
MSMEG_2648	Sfp-type phosphopantetheinyl transferase		0.46	1.72E-05	0.83	1.28E-02	2.63	1.57E-02
MSMEG_2649	tRNA pseudouridine synthase B	*truB*	0.37	2.73E-07	1.20	4.17E-02	3.01	7.48E-03
MSMEG_2650	acyl-CoA dehydrogenase		0.34	8.22E-04	1.96	2.98E-01	14.22	5.30E-02
MSMEG_2651	alkanesulfonate monooxygenase family protein		0.42	4.14E-04	3.74	3.32E-01	10.56	1.24E-02
MSMEG_2652	iron repressor protein		1.07	2.29E-02	0.92	3.21E-01	1.11	3.54E-01
MSMEG_2653	riboflavin biosynthesis protein RibF	*ribF*	0.70	2.11E-04	1.06	3.25E-01	1.30	3.93E-03
MSMEG_2654	ribosomal protein S15	*rpsO*	0.94	2.84E-01	0.79	2.23E-01	0.48	8.74E-04
MSMEG_2655	LppU protein		0.47	2.39E-03	0.94	2.94E-01	1.03	8.79E-01
MSMEG_2656	guanosine pentaphosphate synthetase I/polyribonucleotide nucleotidyltransferase	*gpsI*	0.86	9.61E-03	0.93	5.35E-01	1.17	1.89E-01
MSMEG_2657	peptidase, M16 family protein		0.18	2.05E-07	1.13	2.02E-01	2.46	1.03E-02
MSMEG_2658	beta-lactamase		0.30	7.95E-05	0.92	7.34E-01	1.26	2.44E-01
MSMEG_2659	alanine dehydrogenase	*ald*	3.97	1.88E-02	0.79	9.60E-02	9.49	1.88E-01
MSMEG_2660	transcriptional regulator, AsnC family protein		1.20	2.07E-01	0.72	1.94E-02	1.97	1.35E-01
MSMEG_2661	domain of unknown function (306) family protein		0.85	6.84E-03	1.11	2.27E-01	1.19	1.57E-02
MSMEG_2662	hypothetical protein		1.50	4.60E-02	1.02	7.23E-01	1.12	1.86E-01
MSMEG_2663	conserved hypothetical protein		3.21	1.92E-01	0.78	1.58E-01	1.87	2.88E-01
MSMEG_2664	dihydrodipicolinate reductase	*dapB*	0.67	3.52E-04	1.12	2.05E-01	1.09	4.72E-01
MSMEG_2665	conserved hypothetical protein		0.39	4.18E-03	1.02	8.64E-01	7.17	3.08E-01
MSMEG_2666	multimeric flavodoxin WrbA		0.46	8.55E-05	1.14	1.39E-01	2.19	2.79E-02
MSMEG_2667	HpcH/HpaI aldolase/citrate lyase family protein, putative		0.64	4.37E-05	1.05	4.48E-01	1.65	3.11E-03

MSMEG_2668	conserved hypothetical protein		0.70	2.48E-03	0.94	5.43E-01	1.50	3.72E-02
MSMEG_2669	hydrolase		1.08	2.34E-01	1.06	5.03E-01	1.27	2.10E-01
MSMEG_2670	thymidylate synthase	*thyA*	0.97	2.86E-01	1.05	5.06E-01	0.95	1.03E-01
MSMEG_2671	dihydrofolate reductase	*folA*	0.55	3.85E-04	1.01	9.40E-01	1.10	7.24E-01
MSMEG_2672	acyl-CoA synthase		1.04	4.32E-01	1.19	2.72E-02	0.85	6.02E-02
MSMEG_2673	similar at the nucleotide level to IS1137 in the IS Finder database. This gene is disrupted by an IS element.; IS1137, transposase orfB, interruption-C		0.75	2.38E-02	1.21	1.75E-01	1.86	1.80E-01
MSMEG_2674	similar at the nucleotide level to IS1137 in the IS Finder database. This gene is disrupted by an IS element.; IS1137, transposase orfB, interruption-N		0.60	1.20E-01	1.21	4.72E-02	0.94	2.18E-01
MSMEG_2675	ISMsm8, transposase		0.89	6.07E-01	1.14	4.10E-01	0.99	9.43E-01
MSMEG_2676	IS1137, transposase orfA		0.24	8.05E-05	5.01	1.05E-01	1.44	2.07E-01
MSMEG_2676	IS1137, transposase orfA		0.30	9.39E-05	3.82	1.10E-01	3.70	2.71E-01
MSMEG_2677	conserved large membrane protein		0.99	9.50E-01	1.26	4.60E-01	1.15	3.16E-01
MSMEG_2678	hypothetical protein		0.91	2.86E-01	1.56	6.00E-02	0.95	5.35E-01
MSMEG_2679	conserved hypothetical protein		0.56	5.38E-04	0.96	7.70E-01	1.74	1.80E-01
MSMEG_2680	amino acid transporter		0.78	1.48E-01	0.96	6.93E-01	0.96	5.12E-01
MSMEG_2681	proline imino-peptidase		0.83	4.08E-01	0.91	4.09E-01	0.90	3.51E-01
MSMEG_2682	transcriptional regulator		0.84	3.26E-01	1.01	9.43E-01	1.11	4.37E-01
MSMEG_2683	thymidylate synthase, flavin-dependent	*thyX*	0.45	8.44E-05	1.02	7.62E-01	1.89	2.80E-01
MSMEG_2684	dihydrodipicolinate synthase	*dapA*	0.71	2.68E-04	0.91	1.79E-01	2.43	1.07E-02
MSMEG_2685	metallo-beta-lactamase superfamily protein		0.51	9.92E-05	1.07	3.49E-01	3.37	3.67E-04
MSMEG_2686	conserved hypothetical protein		0.90	1.61E-01	0.95	5.64E-01	2.32	1.98E-01
MSMEG_2687	carveol dehydrogenase		0.96	4.24E-01	1.01	9.35E-01	1.91	3.49E-02
MSMEG_2688	antibiotic biosynthesis monooxygenase domain protein		0.97	7.74E-01	0.77	7.09E-02	1.41	2.22E-01
MSMEG_2689	hypothetical protein		1.04	8.17E-01	1.01	9.09E-01	1.01	7.47E-01
MSMEG_2690	DNA translocase FtsK		0.93	7.66E-01	1.18	3.37E-01	1.45	4.10E-01
MSMEG_2691	acetyltransferase, gnat family protein		0.69	1.16E-02	0.97	7.97E-01	1.26	1.37E-01
MSMEG_2692	CDP-diacylglycerol--glycerol-3-phosphate 3-phosphatidyltransferase	*pgsA*	0.73	1.12E-02	1.02	6.89E-01	1.21	2.75E-01
MSMEG_2693	competence-damaged protein		0.53	4.08E-04	0.90	1.50E-01	1.58	5.14E-02
MSMEG_2694	transcriptional regulator, XRE family protein		2.60	1.45E-03	1.10	1.68E-01	0.83	1.20E-01
MSMEG_2695	35 kDa protein		0.62	2.77E-03	1.29	9.78E-02	0.85	6.69E-02
MSMEG_2696	putative conserved membrane alanine rich protein		0.96	6.15E-01	1.03	8.56E-01	0.73	1.03E-02

MSMEG_2697	conserved hypothetical protein		0.84	5.46E-01	1.21	1.84E-01	0.71	2.46E-02
MSMEG_2698	conserved hypothetical protein		1.17	1.15E-01	1.07	1.22E-01	0.56	4.06E-04
MSMEG_2699	conserved hypothetical protein		0.43	1.12E-03	2.30	7.59E-02	1.09	6.93E-01
MSMEG_2700	conserved hypothetical protein		1.09	1.65E-01	1.32	1.52E-01	1.30	1.24E-01
MSMEG_2701	hypothetical protein		3.08	1.76E-02	0.89	5.41E-01	1.70	1.46E-03
MSMEG_2702	hydrogenase expression/formation protein HypD	*hypD*	3.59	5.12E-03	1.41	2.68E-01	2.38	1.43E-02
MSMEG_2703	hydrogenase assembly chaperone HypC/HupF	*hypC*	6.99	3.83E-02	1.47	1.01E-01	3.48	2.84E-02
MSMEG_2704	transposase; this gene contains a frame shift which is not the result of sequencing error; identified by match to protein family HMM PF01548		2.40	9.31E-04	0.94	5.62E-01	1.01	7.35E-01
MSMEG_2705	hydrogenase expression/formation protein HypE	*hypE*	4.91	2.10E-02	1.22	3.04E-01	3.73	1.40E-01
MSMEG_2706	phosphoheptose isomerase	*gmhA*	13.42	3.07E-02	1.50	2.04E-01	5.59	1.23E-04
MSMEG_2707	hypothetical protein		1.50	1.26E-03	0.93	6.94E-02	0.98	9.05E-01
MSMEG_2708	hypothetical protein		6.75	2.08E-02	1.19	4.93E-01	3.06	5.54E-02
MSMEG_2709	hypothetical protein		1.26	1.39E-01	1.17	2.55E-01	0.97	5.57E-01
MSMEG_2710	hypothetical protein		3.95	9.14E-02	1.66	1.56E-01	5.15	4.46E-02
MSMEG_2711	[NiFe] hydrogenase maturation protein HypF	*hypF*	7.02	5.85E-02	1.57	2.22E-01	11.23	1.37E-01
MSMEG_2712	hydrogenase assembly chaperone HypC/HupF	*hypC*	15.08	3.82E-02	1.55	2.04E-01	12.09	1.56E-02
MSMEG_2713	peptidase M52, hydrogen uptake protein		14.46	1.06E-03	1.36	3.74E-01	8.87	2.94E-02
MSMEG_2714	hypothetical protein		13.04	2.54E-02	1.34	2.38E-01	5.78	1.77E-02
MSMEG_2715	conserved hypothetical protein		3.77	1.21E-01	1.40	3.75E-02	1.17	3.38E-01
MSMEG_2716	conserved hypothetical protein		6.19	9.73E-02	1.23	3.01E-01	1.89	2.08E-02
MSMEG_2717	conserved hypothetical protein		3.98	3.06E-02	1.17	3.93E-01	1.43	2.77E-01
MSMEG_2718	iron-sulfur cluster-binding protein, Rieske family protein, putative		15.35	1.22E-02	0.92	5.32E-01	3.47	1.43E-02
MSMEG_2719	hydrogen:quinone oxidoreductase		18.22	7.22E-04	1.09	7.42E-01	6.61	1.07E-02
MSMEG_2720	NADH ubiquinone oxidoreductase, 20 kda subunit		27.79	2.53E-03	1.24	3.31E-01	3.09	6.98E-02
MSMEG_2721	hydrogenase accessory protein HypB	*hypB*	7.94	4.32E-02	0.96	8.08E-01	1.01	8.96E-01
MSMEG_2722	hydrogenase nickel insertion protein HypA	*hypA*	1.45	4.57E-03	1.19	2.43E-01	0.82	1.08E-01
MSMEG_2723	protein RecA	*recA*	1.18	3.39E-01	0.66	1.18E-02	1.95	1.58E-01
MSMEG_2724	regulatory protein RecX		0.71	5.42E-02	0.75	1.13E-01	3.29	2.05E-02
MSMEG_2725	glutamate transporter permease protein GluD		0.44	3.49E-06	0.95	5.42E-01	0.45	4.12E-03
MSMEG_2726	glutamate permease		0.60	2.25E-03	1.03	8.02E-01	0.32	3.91E-04
MSMEG_2727	glutamate binding protein		0.62	3.10E-04	1.11	2.31E-01	0.27	2.62E-04
MSMEG_2728	glutamate transport ATP-binding protein GluA		0.56	2.74E-04	1.15	4.37E-03	0.34	2.03E-04
MSMEG_2729	tRNA-I(6)A37 thiotransferase enzyme MiaB	*miaB*	0.43	4.43E-05	1.05	2.39E-01	1.82	2.18E-02

MSMEG_2730	conserved hypothetical protein; this gene contains a frame shift which is not the result of sequencing error		0.48	3.27E-04	0.83	1.39E-01	2.05	2.99E-02
MSMEG_2730	conserved hypothetical protein; this gene contains a frame shift which is not the result of sequencing error		0.63	3.93E-03	1.12	3.81E-01	1.23	6.24E-02
MSMEG_2731	ATPase involved in DNA repair		1.13	1.76E-01	1.02	7.79E-01	0.65	3.57E-02
MSMEG_2732	conserved hypothetical protein		0.59	1.12E-03	0.91	4.50E-01	1.04	6.43E-01
MSMEG_2733	conserved hypothetical protein		0.62	7.32E-03	0.93	3.94E-01	1.18	1.26E-01
MSMEG_2734	tRNA delta(2)-isopentenylpyrophosphate transferase	*miaA*	0.56	6.02E-03	1.04	5.57E-01	1.47	4.19E-01
MSMEG_2735	diaminopimelate epimerase	*dapF*	1.16	1.53E-01	0.77	8.01E-02	1.25	3.13E-01
MSMEG_2736	GTP-binding protein		1.40	2.33E-02	0.88	1.80E-01	1.52	4.86E-02
MSMEG_2737	ppe family protein		2.55	1.60E-02	1.08	4.53E-01	0.47	6.13E-02
MSMEG_2738	biotin sulfoxide reductase		1.08	4.06E-01	1.12	1.82E-01	1.43	2.54E-02
MSMEG_2739	conserved hypothetical protein		1.05	5.89E-01	1.10	2.74E-01	0.78	1.50E-02
MSMEG_2740	LexA repressor	*lexA*	2.00	7.40E-05	1.00	8.94E-01	2.39	5.14E-02
MSMEG_2741	hypothetical protein		1.69	2.71E-01	1.20	5.12E-01	3.36	2.52E-01
MSMEG_2742	DNA-damage-inducible protein		1.65	1.50E-01	0.88	6.16E-03	3.60	1.18E-01
MSMEG_2743	transcriptional regulator, NrdR family protein	*nrdR*	1.13	7.81E-02	0.77	1.07E-02	2.88	9.25E-02
MSMEG_2744	thymidylate synthase		3.16	3.36E-04	0.93	1.91E-01	0.87	4.35E-02
MSMEG_2745	hydrolase; this gene contains a frame shift which is not the result of sequencing error; identified by match to protein family HMM PF00561		0.87	2.78E-02	1.10	2.49E-01	1.66	2.60E-01
MSMEG_2746	conserved hypothetical alanine and leucine rich protein		1.32	1.25E-01	1.21	1.44E-01	1.13	4.56E-01
MSMEG_2747	conserved hypothetical protein; this gene contains a frame shift which is not the result of sequencing error		2.09	6.79E-03	0.65	2.45E-02	0.73	4.40E-02
MSMEG_2747	conserved hypothetical protein; this gene contains a frame shift which is not the result of sequencing error		1.32	4.86E-02	1.20	1.52E-01	1.15	1.01E-01
MSMEG_2748	soluble pyridine nucleotide transhydrogenase	*sthA*	1.00	9.87E-01	2.23	1.15E-02	0.72	7.48E-02
MSMEG_2749	conserved hypothetical protein		1.16	2.82E-01	1.17	3.27E-01	0.97	5.95E-01
MSMEG_2750	iron-dependent repressor IdeR		2.10	2.38E-04	1.20	8.48E-02	1.19	7.96E-03
MSMEG_2751	hypothetical protein		0.50	6.61E-03	3.85	1.18E-01	1.23	3.58E-01
MSMEG_2752	sigma factor SigB		1.81	1.22E-03	1.43	1.21E-01	1.72	2.14E-02
MSMEG_2753	conserved hypothetical protein		2.23	8.21E-05	1.02	8.52E-01	1.13	5.34E-01

MSMEG_2754	conserved hypothetical protein		2.10	1.35E-02	0.87	2.18E-01	0.91	6.59E-01
MSMEG_2755	conserved hypothetical protein		0.84	4.93E-02	1.53	1.89E-02	2.42	4.77E-03
MSMEG_2756	conserved hypothetical protein		1.30	1.14E-01	1.06	6.68E-01	3.52	1.36E-03
MSMEG_2757	glutathione S-transferase domain protein		2.98	5.85E-03	0.61	5.23E-03	6.87	4.28E-03
MSMEG_2758	sigma factor MysA		1.37	2.97E-02	0.70	2.22E-02	1.30	3.36E-01
MSMEG_2759	hypothetical protein		1.17	5.07E-01	0.77	2.39E-01	5.63	1.36E-01
MSMEG_2760	polyphosphate glucokinase		0.86	1.64E-01	0.89	2.77E-01	0.63	5.19E-03
MSMEG_2761	putative secreted alanine rich protein		0.88	1.15E-01	0.87	1.87E-01	0.83	1.42E-01
MSMEG_2762	inositol-1-monophosphatase		0.80	5.12E-02	0.93	6.41E-01	0.91	5.01E-01
MSMEG_2763	conserved hypothetical protein		2.10	1.10E-02	0.99	9.53E-01	1.10	3.80E-01
MSMEG_2764	probable conserved alanine rich transmembrane protein		0.69	1.05E-03	0.95	6.97E-01	1.02	7.61E-01
MSMEG_2765	deoxyuridine 5'-triphosphate nucleotidohydrolase		0.80	3.11E-02	1.40	5.37E-02	2.41	5.12E-03
MSMEG_2766	conserved hypothetical protein		0.68	1.74E-03	1.16	1.70E-01	2.36	1.20E-02
MSMEG_2767	conserved hypothetical protein		1.48	8.63E-04	0.99	8.64E-01	1.65	2.49E-01
MSMEG_2768	OB-fold nucleic acid binding domain protein		1.62	3.25E-03	0.80	7.64E-05	3.26	4.50E-02
MSMEG_2769	TrkB protein		0.95	2.93E-01	0.94	5.74E-01	1.30	1.75E-01
MSMEG_2770	probable conserved integral membrane alanine and leucine rich protein		1.02	7.89E-01	0.93	3.69E-01	3.76	2.28E-03
MSMEG_2771	TrkA protein		0.86	2.02E-01	0.90	2.02E-01	1.58	9.81E-03
MSMEG_2772	amino acid permease		1.83	5.94E-04	0.79	2.24E-02	1.46	2.30E-02
MSMEG_2773	hypothetical RNA methyltransferase		1.08	1.87E-01	0.80	1.23E-02	2.97	4.80E-03
MSMEG_2774	diguanylate cyclase (ggdef) domain protein		2.43	2.42E-03	0.73	2.12E-02	0.92	2.47E-02
MSMEG_2775	Na+/H+ antiporter NhaA	*nhaA*	1.24	3.58E-03	0.84	5.20E-02	1.48	1.08E-01
MSMEG_2776	1-deoxy-D-xylulose-5-phosphate synthase	*dxs*	1.16	8.13E-02	0.86	6.47E-02	1.13	6.62E-02
MSMEG_2777	alpha/beta hydrolase fold		0.67	7.90E-03	0.85	7.19E-02	1.34	6.87E-02
MSMEG_2778	ribonuclease D		0.61	6.90E-03	1.20	3.19E-01	5.10	8.45E-03
MSMEG_2778	ribonuclease D		0.82	1.81E-02	0.94	4.32E-01	2.78	6.79E-02
MSMEG_2779	conserved hypothetical protein		0.81	1.01E-01	1.06	5.44E-01	2.06	4.63E-02
MSMEG_2780	uroporphyrinogen decarboxylase	*hemE*	0.82	1.02E-02	1.03	5.81E-01	1.37	4.62E-02
MSMEG_2781	protoporphyrinogen oxidase	*hemG*	0.79	5.63E-01	1.01	9.30E-01	1.30	1.40E-01
MSMEG_2782	conserved hypothetical protein		0.66	4.45E-04	1.01	9.10E-01	2.55	2.24E-03
MSMEG_2783	hypothetical protein		1.24	1.05E-01	0.98	8.46E-01	1.27	2.17E-01
MSMEG_2784	methionine-R-sulfoxide reductase	*msrB*	2.20	3.55E-03	0.86	1.96E-01	0.85	1.86E-01
MSMEG_2785	putative conserved integral membrane protein		0.47	5.51E-04	0.91	3.45E-01	0.80	3.03E-01
MSMEG_2786	hydrolase, alpha/beta fold family protein		0.67	1.29E-04	1.06	7.21E-01	0.96	8.07E-01
MSMEG_2787	putative riboflavin biosynthesis protein RibD		1.18	5.16E-01	0.81	2.15E-01	0.98	6.12E-01
MSMEG_2788	ATP/GTP-binding integral membrane protein		0.42	1.93E-04	0.94	3.32E-01	2.99	2.35E-02

MSMEG_2789	acetyltransferase, GNAT family protein	0.87	2.73E-03	0.87	5.05E-01	1.00	9.94E-01
MSMEG_2790	conserved hypothetical protein	1.11	3.70E-01	0.89	2.76E-01	0.29	4.77E-04
MSMEG_2791	pyridoxamine 5'-phosphate oxidase family	2.82	1.04E-02	0.65	1.08E-01	0.69	1.64E-01
MSMEG_2792	Clp amino terminal domain protein	0.88	4.01E-03	1.14	3.62E-01	0.97	3.23E-01
MSMEG_2793	sensor-type histidine kinase PrrB	0.49	3.50E-03	1.04	4.85E-01	1.43	2.50E-01
MSMEG_2794	transcriptional regulator, GntR family protein	1.58	4.64E-01	0.96	4.43E-01	1.00	9.42E-01
MSMEG_2795	transmembrane transport protein	0.76	4.89E-02	0.91	4.38E-01	1.09	3.60E-01
MSMEG_2796	integral membrane protein	1.35	1.71E-01	0.92	1.73E-01	1.84	1.80E-02
MSMEG_2797	LysR-family protein transcriptional regulator	1.01	9.30E-01	0.84	3.72E-01	1.15	4.68E-01
MSMEG_2798	hypothetical protein	0.80	4.42E-01	0.60	7.38E-03	1.05	7.37E-01
MSMEG_2799	phospho-2-dehydro-3-deoxyheptonate aldolase	0.27	2.02E-04	0.89	3.30E-01	1.61	2.61E-01
MSMEG_2800	NADPH-dependent fmn reductase	0.52	1.46E-04	1.06	7.70E-01	0.84	4.96E-01
MSMEG_2801	hypothetical protein	1.19	8.14E-02	1.07	6.23E-01	1.11	2.88E-01
MSMEG_2802	hypothetical protein	0.82	1.82E-01	0.93	5.73E-01	0.66	4.82E-03
MSMEG_2802	hypothetical protein	1.07	6.09E-01	0.97	8.07E-01	1.37	5.68E-01
MSMEG_2803	hypothetical protein	0.83	8.23E-02	0.86	1.41E-01	0.77	1.60E-01
MSMEG_2804	two-component system sensor kinase	0.87	1.24E-01	1.15	4.76E-01	0.79	1.01E-01
MSMEG_2805	ISMsm5, transposase	1.10	3.24E-02	1.08	4.99E-02	0.47	2.51E-05
MSMEG_2805	ISMsm5, transposase	0.93	2.89E-01	0.95	2.11E-01	0.57	3.66E-04
MSMEG_2805	ISMsm5, transposase	1.04	3.81E-01	0.98	5.41E-01	0.66	5.69E-04
MSMEG_2806	two-component system response regulator	1.01	8.81E-01	0.98	6.90E-01	1.04	8.35E-01
MSMEG_2807	two-component system response regulator	0.97	5.52E-01	1.13	2.81E-01	1.47	2.87E-01
MSMEG_2808	short-chain dehydrogenase/reductase SDR	0.85	6.04E-01	1.28	3.49E-01	1.02	5.97E-01
MSMEG_2809	conserved hypothetical protein	1.05	8.23E-01	1.34	3.50E-01	1.08	4.75E-01
MSMEG_2810	major facilitator superfamily protein	0.95	6.38E-01	1.02	8.62E-01	1.31	3.63E-01
MSMEG_2811	hydride transferase 1	2.13	2.69E-02	1.00	9.64E-01	1.06	7.75E-01
MSMEG_2812	C-5 sterol desaturase	0.96	6.99E-01	1.05	7.63E-01	1.02	6.24E-01
MSMEG_2813	sensor kinase	1.23	2.80E-01	0.88	1.97E-01	1.22	3.07E-01
MSMEG_2814	two component system response regulator; this gene contains a frame shift which is not the result of sequencing error; identified by match to protein family HMM PF00072	1.98	1.47E-02	1.06	5.66E-01	1.51	6.00E-02
MSMEG_2815	putative tape-measure protein; this region contains one or more premature stops and/or frameshifts which are not the result of sequencing error	0.39	1.58E-04	2.01	4.41E-04	2.00	1.15E-02

MSMEG_2815	putative tape-measure protein; this region contains one or more premature stops and/or frameshifts which are not the result of sequencing error	0.91	4.18E-01	1.40	1.56E-01	1.27	9.41E-02
MSMEG_2816	conserved hypothetical protein; this gene contains a frame shift which is not the result of sequencing error	0.81	8.23E-02	1.06	5.82E-01	1.04	7.56E-01
MSMEG_2817	ABC efflux pump, fused inner membrane and ATPase subunits	0.94	6.29E-01	1.09	2.37E-01	1.37	3.87E-02
MSMEG_2818	ISMsm1, transposase orfB	0.58	6.81E-04	1.58	1.25E-01	1.17	3.34E-01
MSMEG_2818	ISMsm1, transposase orfB	0.90	3.05E-01	1.14	4.34E-01	1.00	9.67E-01
MSMEG_2819	ISMsm1, transposase orfA	1.45	3.06E-01	1.39	2.28E-02	1.09	4.39E-01
MSMEG_2819	ISMsm1, transposase orfA	0.95	7.70E-01	1.12	1.88E-01	0.90	5.07E-01
MSMEG_2820	hypothetical protein	0.88	3.58E-02	1.41	2.31E-02	3.23	4.71E-01
MSMEG_2822	transposase, truncation	0.67	3.15E-03	0.69	1.73E-02	0.85	4.29E-01
MSMEG_2823	This gene is disrupted by an IS1549 element.; IS1549, transposase, interruption-N; identified by similarity to GP:2935133; match to protein family HMM PF01609	2.18	5.78E-02	1.50	4.03E-02	0.65	5.64E-02
MSMEG_2823	This gene is disrupted by an IS1549 element.; IS1549, transposase, interruption-N; identified by similarity to GP:2935133; match to protein family HMM PF01609	2.02	5.92E-02	1.35	6.49E-02	0.84	3.85E-01
MSMEG_2824	IS1549, transposase	0.75	1.62E-02	1.45	3.68E-02	1.69	6.26E-03
MSMEG_2824	IS1549, transposase	1.45	8.30E-02	0.69	6.10E-02	2.15	3.59E-02
MSMEG_2824	IS1549, transposase	0.91	1.34E-01	1.07	1.57E-01	1.07	3.42E-01
MSMEG_2824	IS1549, transposase	1.11	5.38E-01	0.88	2.48E-01	1.00	9.23E-01
MSMEG_2826	hypothetical protein	1.51	8.42E-02	1.57	1.64E-01	0.31	1.14E-05
MSMEG_2827	hypothetical protein	1.04	5.82E-01	1.06	7.72E-01	1.10	1.08E-01
MSMEG_2830	ISMsm4, transposase	0.82	2.57E-01	1.52	1.50E-02	0.68	2.77E-02
MSMEG_2830	ISMsm4, transposase	1.08	2.96E-01	1.11	2.37E-01	1.06	6.78E-01
MSMEG_2831	hypothetical protein	1.41	1.74E-02	0.93	3.17E-01	1.38	8.12E-02
MSMEG_2832	hypothetical protein	2.43	2.76E-03	0.90	2.79E-01	0.89	3.35E-01
MSMEG_2837	nitrate reductase NarB	1.40	7.06E-02	1.49	2.60E-02	0.95	2.29E-01
MSMEG_2838	conserved hypothetical protein	1.08	4.12E-01	1.50	3.71E-01	0.87	2.43E-01
MSMEG_2839	transcriptional accessory protein	0.33	2.13E-03	1.34	4.40E-01	1.31	4.41E-01
MSMEG_2840	conserved hypothetical protein	1.22	6.04E-01	0.92	3.80E-01	1.12	3.57E-01
MSMEG_2841	putative monooxygenase	1.11	4.40E-01	1.39	6.44E-02	1.75	3.59E-01

MSMEG_2842	nitrilotriacetate monooxygenase component A	*ssuD*	1.55	4.16E-01	1.06	2.58E-01	1.50	3.90E-01
MSMEG_2843	nitrilotriacetate monooxygenase component A	*ssuD*	1.33	1.81E-01	1.68	3.76E-01	1.09	3.55E-01
MSMEG_2844	ABC transporter, ATP-binding protein		1.34	2.99E-01	1.16	1.64E-01	1.37	3.40E-01
MSMEG_2845	ABC transporter permease protein		1.24	1.88E-01	1.12	4.99E-01	0.92	1.79E-01
MSMEG_2846	ABC transporter permease protein		0.94	5.78E-01	1.14	5.35E-01	1.03	8.48E-01
MSMEG_2847	bacterial extracellular solute-binding protein, family protein 5		2.63	1.33E-01	1.17	3.04E-01	1.00	9.67E-01
MSMEG_2848	transmembrane efflux protein		1.14	3.73E-01	0.93	1.03E-01	1.00	9.53E-01
MSMEG_2849	transcriptional regulatory protein		1.78	5.35E-02	0.84	2.38E-01	1.01	9.64E-01
MSMEG_2850	mycobacterial family protein 11 protein		2.25	2.09E-02	0.99	9.56E-01	0.88	2.87E-01
MSMEG_2851	conserved hypothetical protein		0.92	1.72E-01	1.28	1.20E-01	1.07	4.04E-01
MSMEG_2852	3-oxoacyl-[acyl-carrier-protein] reductase		0.98	8.97E-01	1.11	9.46E-02	1.26	4.69E-01
MSMEG_2853	conserved hypothetical protein		0.81	1.68E-02	1.51	2.00E-01	4.30	3.82E-01
MSMEG_2854	conserved hypothetical protein		0.97	6.51E-01	1.19	2.85E-01	1.01	8.93E-01
MSMEG_2855	virulence factor Mce family protein		1.28	2.38E-01	1.18	5.85E-01	1.01	7.86E-01
MSMEG_2856	virulence factor Mce family protein		1.11	4.59E-01	1.24	1.70E-01	1.65	1.82E-01
MSMEG_2857	virulence factor Mce family protein		1.31	2.43E-01	1.07	5.32E-01	1.17	3.13E-01
MSMEG_2858	virulence factor mce family protein		0.98	8.68E-01	1.32	6.95E-02	1.89	1.44E-01
MSMEG_2859	virulence factor Mce family protein		1.21	2.30E-01	1.16	4.20E-01	1.07	5.25E-01
MSMEG_2860	virulence factor mce family protein		1.46	1.95E-01	1.40	2.26E-01	0.88	1.61E-01
MSMEG_2861	conserved hypothetical protein		1.12	1.78E-01	1.15	3.73E-01	1.49	3.53E-01
MSMEG_2862	conserved hypothetical protein		1.02	6.04E-01	1.48	3.43E-01	2.21	3.65E-01
MSMEG_2863	conserved hypothetical protein		1.22	5.19E-02	0.87	7.42E-02	1.00	9.64E-01
MSMEG_2864	conserved hypothetical protein		1.12	5.33E-01	1.23	1.63E-01	1.09	5.17E-01
MSMEG_2865	conserved hypothetical protein		0.90	6.33E-01	1.17	6.07E-02	1.10	4.51E-01
MSMEG_2866	thioesterase family protein		0.77	2.00E-01	1.24	2.43E-01	1.27	2.70E-01
MSMEG_2867	3-ketosteroid dehydrogenase		1.11	5.79E-01	1.21	2.19E-01	1.02	7.57E-01
MSMEG_2868	transcriptional regulator, PadR family protein		0.65	1.57E-03	1.13	2.73E-01	1.07	1.79E-01
MSMEG_2869	3-ketosteroid-delta-1-dehydrogenase		1.74	3.06E-01	0.91	3.35E-01	1.03	5.10E-01
MSMEG_2870	oxygenase KshA		1.10	5.67E-01	1.01	9.65E-01	0.91	2.65E-01
MSMEG_2871	conserved hypothetical protein		1.05	7.39E-01	1.25	6.52E-02	1.76	4.00E-01
MSMEG_2872	short-chain dehydrogenase/reductase SDR		1.23	2.77E-01	1.92	7.67E-02	0.99	7.01E-01
MSMEG_2873	3-ketosteroid-delta4		1.39	3.90E-01	1.37	4.76E-01	1.32	4.77E-01
MSMEG_2874	conserved hypothetical protein		1.64	1.13E-01	0.86	4.39E-02	0.94	2.38E-01
MSMEG_2875	esterase		1.12	2.72E-01	1.14	3.14E-02	0.98	5.34E-01
MSMEG_2876	oxidoreductase, short chain dehydrogenase/reductase family protein		0.95	6.05E-01	0.96	7.73E-01	2.07	3.92E-01
MSMEG_2877	conserved hypothetical protein		1.07	3.97E-01	1.07	3.35E-01	0.93	5.12E-01

MSMEG_2878	putative acyl-CoA dehydrogenase	0.77	3.08E-02	1.07	4.52E-01	1.18	4.23E-01
MSMEG_2879	acyl-CoA dehydrogenase	0.83	2.27E-01	1.10	5.47E-01	1.04	5.56E-01
MSMEG_2880	enoyl-CoA hydratase	0.78	8.49E-02	0.94	1.92E-01	1.07	2.54E-01
MSMEG_2881	aldehyde dehydrogenase	0.75	4.03E-02	1.14	5.55E-01	1.09	4.16E-01
MSMEG_2882	5-exo-alcohol dehydrogenase	1.39	3.31E-01	1.28	4.43E-01	1.08	2.25E-01
MSMEG_2883	feruloyl-CoA synthetase	0.85	4.22E-01	1.27	2.53E-01	1.07	1.88E-01
MSMEG_2884	cyclohexanone monooxygenase	1.18	1.80E-01	1.01	9.41E-01	0.94	2.55E-01
MSMEG_2885	short-chain dehydrogenase/reductase SDR	1.52	4.62E-01	1.01	8.87E-01	1.11	5.10E-01
MSMEG_2886	stress responsive A/B Barrel Domain family protein	1.18	3.76E-01	0.98	8.68E-01	0.99	4.82E-01
MSMEG_2887	conserved hypothetical protein	0.94	6.07E-01	1.15	3.32E-01	1.09	3.66E-01
MSMEG_2888	lipase	1.18	1.69E-01	1.20	3.95E-01	1.47	1.31E-01
MSMEG_2889	NADH:flavin oxidoreductase/nadh oxidase	0.96	8.09E-01	1.13	6.30E-02	1.35	3.10E-01
MSMEG_2890	putative transcriptional regulator	1.01	9.00E-01	1.13	4.40E-01	1.07	3.56E-01
MSMEG_2891	biphenyl-2,3-diol 1,2-dioxygenase 1	1.10	2.06E-01	1.18	1.17E-01	1.22	2.78E-01
MSMEG_2892	pigment production hydroxylase	1.40	1.81E-01	1.02	7.96E-01	0.96	3.51E-01
MSMEG_2893	oxidoreductase, NAD/FAD-binding	1.40	1.27E-01	1.45	1.82E-01	0.96	3.83E-01
MSMEG_2894	steroid monooxygenase	0.85	1.68E-01	1.07	5.98E-01	1.06	6.26E-01
MSMEG_2895	short chain oxidoreductase	0.44	1.63E-04	1.01	8.11E-01	1.03	8.19E-01
MSMEG_2896	conserved hypothetical protein	1.06	2.83E-01	0.68	4.27E-02	3.23	3.03E-01
MSMEG_2897	conserved hypothetical protein	1.33	4.18E-02	1.04	8.26E-01	1.10	6.53E-01
MSMEG_2898	transcriptional regulator, TetR family protein	0.54	8.06E-03	1.04	6.47E-01	1.14	4.88E-01
MSMEG_2899	oxidoreductase, short chain dehydrogenase/reductase family protein	0.44	5.09E-05	1.13	1.19E-01	1.32	4.19E-01
MSMEG_2900	2-hydroxy-6-oxo-6-phenylhexa-2,4-dienoate hydrolase	0.31	1.07E-05	1.06	7.25E-01	1.27	3.04E-01
MSMEG_2901	conserved hypothetical protein	0.41	4.93E-06	1.14	4.28E-01	2.46	3.61E-01
MSMEG_2902	hydrolase, putative	0.73	3.58E-02	1.30	2.84E-01	1.67	7.26E-02
MSMEG_2903	transposase; this gene contains a frame shift which is not the result of sequencing error	1.47	5.84E-03	1.58	1.41E-01	1.23	1.31E-01
MSMEG_2903	transposase; this gene contains a frame shift which is not the result of sequencing error	1.32	4.44E-02	1.37	2.53E-01	1.65	1.46E-01
MSMEG_2903	transposase; this gene contains a frame shift which is not the result of sequencing error	1.01	7.62E-01	1.15	5.46E-01	1.09	3.75E-01
MSMEG_2904	MmcI protein	2.10	3.92E-02	0.82	1.31E-03	1.57	2.99E-01
MSMEG_2905	transcriptional regulator	1.84	2.25E-03	1.13	4.35E-01	1.30	1.62E-01
MSMEG_2906	conserved hypothetical protein	1.12	2.38E-01	2.03	1.60E-01	1.03	5.06E-01
MSMEG_2907	hypothetical oxidoreductase YeiQ	1.06	6.93E-01	0.99	8.69E-01	1.00	9.87E-01

MSMEG_2908	2-Keto-3-deoxy-gluconate kinase		1.06	7.31E-01	1.37	1.89E-01	0.99	8.03E-01
MSMEG_2909	starvation-sensing protein RspA		1.22	1.56E-01	0.96	7.95E-01	1.01	8.84E-01
MSMEG_2910	transcriptional regulator, GntR family protein		1.18	1.15E-01	0.99	6.12E-01	0.75	7.83E-02
MSMEG_2911	integral membrane transport protein		1.06	7.45E-01	1.20	3.10E-01	0.91	4.44E-01
MSMEG_2912	inner membrane metabolite transport protein YdfJ		0.95	4.36E-01	2.00	1.79E-01	1.27	5.34E-01
MSMEG_2913	hydrolase		1.00	9.88E-01	1.15	1.11E-01	0.79	1.18E-01
MSMEG_2914	L-idonate 5-dehydrogenase		1.03	8.69E-01	1.01	4.19E-01	1.39	2.01E-01
MSMEG_2915	sensor histidine kinase		1.81	2.30E-02	1.08	4.89E-01	0.92	3.96E-01
MSMEG_2916	DNA-binding response regulator, PhoP family protein		3.56	1.95E-01	0.96	5.77E-01	1.23	1.77E-01
MSMEG_2917	hypothetical protein		2.43	1.47E-02	0.85	1.54E-01	1.29	2.67E-02
MSMEG_2918	short-chain dehydrogenase/reductase SDR		1.25	4.13E-01	1.07	4.88E-01	1.12	5.38E-01
MSMEG_2919	2-dehydropantoate 2-reductase		1.40	1.21E-01	0.97	8.18E-01	1.10	1.06E-01
MSMEG_2920	aldo/keto reductase		1.44	2.74E-02	3.31	6.76E-03	1.64	3.62E-02
MSMEG_2921	NADPH-flavin oxidoreductase		1.24	2.97E-01	1.87	5.10E-02	1.18	1.71E-01
MSMEG_2922	conserved hypothetical protein		1.17	9.17E-02	1.04	3.85E-01	0.84	4.24E-02
MSMEG_2923	dehydrogenase/reductase SDR family protein member 1		0.44	1.88E-04	1.20	2.34E-01	1.14	5.17E-01
MSMEG_2924	permease binding-protein component		1.32	4.73E-01	2.34	2.58E-02	0.66	4.43E-02
MSMEG_2925	permease membrane component		0.90	6.71E-01	1.64	4.84E-02	1.13	6.18E-01
MSMEG_2926	glycine betaine/carnitine/choline transport ATP-binding protein opuCA		1.70	2.74E-01	2.57	2.01E-02	0.57	2.35E-02
MSMEG_2927	ABC transporter, permease protein OpuCB		1.39	3.98E-01	2.07	8.96E-02	0.57	1.60E-02
MSMEG_2928	conserved hypothetical protein		1.23	4.48E-01	0.97	8.58E-01	1.16	6.10E-01
MSMEG_2929	thioesterase family protein		0.95	6.34E-01	0.90	3.06E-01	1.44	1.17E-01
MSMEG_2930	conserved hypothetical protein TIGR02611		0.62	2.27E-02	1.15	1.66E-01	0.94	5.45E-01
MSMEG_2931	threonyl-tRNA synthetase	*thrS*	0.88	2.68E-01	1.06	2.54E-01	1.38	9.17E-03
MSMEG_2932	HIT family protein hydrolase		0.57	2.81E-04	0.97	6.08E-01	1.85	1.66E-02
MSMEG_2933	phosphatidylinositol synthase		0.70	8.72E-04	0.79	5.77E-03	1.67	1.71E-01
MSMEG_2934	lipid A biosynthesis lauroyl acyltransferase		0.90	1.34E-01	0.99	9.07E-01	1.79	9.82E-03
MSMEG_2935	phosphatidylinositol alpha-mannosyltransferase		0.76	2.29E-02	1.04	7.46E-01	3.04	2.44E-01
MSMEG_2936	hydrolase, nudix family protein		0.60	5.19E-04	1.12	4.64E-01	2.40	1.38E-01
MSMEG_2937	pyridoxine biosynthesis protein		0.48	1.17E-05	0.97	5.83E-01	1.28	9.59E-03
MSMEG_2938	acyl-CoA thioesterase II	*tesB*	0.26	7.10E-06	1.16	3.44E-01	1.30	1.31E-01
MSMEG_2939	glutamine amidotransferase subunit PdxT		0.33	9.41E-04	1.02	8.94E-01	1.22	5.27E-01
MSMEG_2940	conserved hypothetical protein		0.53	9.35E-04	0.88	4.42E-02	1.67	3.65E-03
MSMEG_2941	NAD dependent epimerase/dehydratase family protein		2.56	4.94E-02	1.00	9.90E-01	1.15	1.14E-01

MSMEG_2942	glyoxalase/bleomycin resistance protein/dioxygenase superfamily protein		1.53	1.57E-02	1.10	5.05E-01	0.99	8.98E-01
MSMEG_2943	crossover junction endodeoxyribonuclease RuvC	*ruvC*	0.91	5.78E-01	0.98	8.74E-01	3.71	2.31E-02
MSMEG_2944	holliday junction DNA helicase RuvA	*ruvA*	0.66	1.49E-02	0.90	2.79E-01	4.10	2.51E-02
MSMEG_2945	holliday junction DNA helicase RuvB	*ruvB*	0.97	5.73E-01	0.92	4.72E-01	1.87	1.26E-01
MSMEG_2946	transmembrane protein		0.88	2.15E-01	0.90	1.72E-01	1.92	9.10E-03
MSMEG_2947	polyketide cyclase		2.47	7.87E-03	0.84	1.77E-01	0.62	5.21E-02
MSMEG_2948	6-oxocamphor hydrolase		1.99	6.74E-03	0.91	4.49E-01	1.09	5.97E-01
MSMEG_2949	carbon-monoxide dehydrogenase		1.66	4.95E-03	1.06	7.18E-01	1.05	3.03E-01
MSMEG_2950	carbon monoxide dehydrogenase, medium chain		1.92	6.19E-02	1.20	3.58E-01	1.34	4.38E-01
MSMEG_2951	[2Fe-2S] binding domain protein		1.07	7.86E-01	2.60	2.68E-01	0.94	2.54E-01
MSMEG_2952	transport gene		0.87	4.44E-01	1.27	3.24E-01	1.05	2.03E-01
MSMEG_2953	ethyl tert-butyl ether degradation EthD		1.28	9.17E-02	1.25	2.16E-01	0.80	4.58E-02
MSMEG_2954	LacI-family protein transcriptional regulator		1.01	9.55E-01	1.10	5.04E-01	0.82	4.14E-04
MSMEG_2955	conserved hypothetical protein		0.70	9.73E-04	1.35	1.58E-01	1.55	1.52E-01
MSMEG_2956	NAD dependent epimerase/dehydratase family protein		0.84	2.89E-01	3.74	1.31E-01	1.04	8.22E-01
MSMEG_2957	conserved hypothetical protein		1.34	1.47E-02	1.40	1.15E-01	0.95	7.15E-01
MSMEG_2958	conserved hypothetical protein		2.48	1.60E-01	2.57	7.65E-02	0.51	5.80E-03
MSMEG_2959	4-aminobutyrate transaminase	*gabT*	1.99	3.72E-03	1.25	6.11E-02	0.32	7.41E-07
MSMEG_2960	preprotein translocase, YajC subunit	*yajC*	1.04	3.74E-01	0.95	4.75E-01	1.16	1.97E-02
MSMEG_2961	protein-export membrane protein SecD	*secD*	0.63	6.15E-04	0.81	4.21E-02	0.96	4.90E-01
MSMEG_2962	protein-export membrane protein SecF		0.55	6.88E-05	0.70	1.04E-02	1.55	6.49E-02
MSMEG_2963	bacterial extracellular solute-binding protein, family protein 5		0.45	3.96E-04	1.13	5.02E-01	2.46	1.09E-01
MSMEG_2964	adenine phosphoribosyltransferase		0.43	3.45E-04	1.01	8.93E-01	1.92	4.13E-02
MSMEG_2965	GTP pyrophosphokinase	*relA*	1.63	2.15E-02	1.07	3.66E-01	2.77	1.92E-02
MSMEG_2966	major facilitator family protein transporter		0.99	9.12E-01	3.03	1.39E-01	1.16	4.47E-01
MSMEG_2967	conserved hypothetical protein		1.01	8.85E-01	4.27	7.76E-02	1.06	7.39E-01
MSMEG_2968	conserved hypothetical protein		0.94	4.26E-01	1.39	1.94E-01	1.12	1.59E-01
MSMEG_2969	NADH:flavin oxidoreductase/nadh oxidase		1.56	1.92E-01	1.28	2.37E-02	1.14	3.90E-01
MSMEG_2970	polysaccharide deacetylase		1.23	1.04E-01	1.41	2.42E-01	1.07	3.75E-01
MSMEG_2971	regulatory protein		1.06	7.77E-01	6.04	9.81E-02	0.94	3.97E-01
MSMEG_2972	permease, cytosine/purines, uracil, thiamine, allantoin family protein		1.21	2.91E-01	1.89	2.79E-02	1.06	2.84E-01
MSMEG_2972	permease, cytosine/purines, uracil, thiamine, allantoin family protein		0.86	3.80E-01	1.18	2.19E-01	0.96	2.94E-01
MSMEG_2973	peptidyl-prolyl cis-trans isomerase		0.42	1.27E-03	1.20	1.02E-01	1.77	1.14E-02

MSMEG_2974	peptidyl-prolyl cis-trans isomerase, cyclophilin-type		0.50	1.09E-03	0.95	4.86E-01	0.68	3.93E-02
MSMEG_2975	metallo-beta-lactamase family protein		1.22	8.56E-02	0.90	7.26E-02	2.07	1.10E-02
MSMEG_2976	histidyl-tRNA synthetase	*hisS*	0.94	7.51E-01	0.79	1.01E-02	2.45	1.54E-03
MSMEG_2977	hypothetical protein		0.31	1.53E-04	1.19	1.63E-01	1.03	5.16E-01
MSMEG_2978	ABC transporter ATP-binding protein		0.18	1.78E-04	2.06	3.21E-01	0.83	1.95E-01
MSMEG_2979	ABC transporter ATP-binding protein		0.17	1.45E-04	2.39	2.68E-01	0.82	1.47E-01
MSMEG_2980	putative membrane protein		0.15	8.35E-05	4.13	3.44E-01	0.90	7.36E-01
MSMEG_2981	branched-chain amino acid ABC-type transport system, permease component		0.12	8.45E-06	1.58	1.78E-01	0.95	7.60E-01
MSMEG_2982	putative periplasmic binding protein		0.11	3.83E-06	2.67	2.96E-01	0.59	8.26E-02
MSMEG_2983	conserved hypothetical protein		0.75	9.12E-03	0.77	1.88E-02	1.07	5.32E-01
MSMEG_2984	putative hydrolase		0.75	5.16E-02	1.31	3.66E-01	1.21	5.77E-01
MSMEG_2985	fumarate hydratase class I, anaerobic		1.95	5.57E-02	1.41	2.92E-01	1.00	9.43E-01
MSMEG_2986	amidohydrolase, AtzE family protein		0.89	4.58E-01	1.11	6.23E-01	1.05	2.90E-01
MSMEG_2987	conserved hypothetical protein; this gene contains a frame shift which is not the result of sequencing error		1.37	6.74E-02	1.57	5.04E-02	1.09	6.67E-01
MSMEG_2988	carbon-nitrogen hydrolase family protein		1.16	3.03E-01	1.64	1.84E-01	1.52	2.76E-01
MSMEG_2989	LacI family protein transcriptional regulator		1.02	7.55E-01	0.77	6.68E-03	0.88	2.30E-01
MSMEG_2990	Fe-S protein, radical SAM family protein		1.08	4.97E-01	0.93	2.51E-01	1.07	6.50E-02
MSMEG_2991	permease of the major facilitator superfamily protein		0.93	7.96E-01	1.38	3.75E-01	1.02	8.76E-01
MSMEG_2992	putative acyl-CoA dehydrogenase		2.05	1.19E-01	2.35	1.12E-01	1.00	9.71E-01
MSMEG_2993	acyl-CoA dehydrogenase, C- domain protein		1.13	1.59E-01	1.83	1.20E-01	1.00	7.53E-01
MSMEG_2994	amidohydrolase family protein		1.57	8.86E-02	1.80	1.88E-01	0.95	3.29E-01
MSMEG_2995	transcriptional regulator, TetR family protein		5.29	1.22E-01	1.47	1.60E-01	0.68	1.18E-01
MSMEG_2996	conserved hypothetical protein		3.56	3.02E-01	1.04	6.82E-01	0.87	1.15E-01
MSMEG_2997	conserved hypothetical protein		1.94	8.47E-03	1.03	8.77E-01	1.47	3.87E-01
MSMEG_2998	conserved hypothetical protein		1.51	3.16E-02	1.29	2.35E-01	1.60	1.23E-01
MSMEG_2998	conserved hypothetical protein		1.25	2.21E-01	0.94	7.04E-01	0.92	3.56E-01
MSMEG_2999	conserved protein		0.77	2.22E-02	1.67	1.19E-01	1.65	6.08E-03
MSMEG_3000	metallopeptidase, zinc binding		1.13	1.03E-01	1.34	6.38E-02	1.27	1.32E-02
MSMEG_3001	conserved hypothetical protein		0.87	9.59E-02	1.94	1.64E-01	1.95	1.45E-03
MSMEG_3002	para-nitrobenzyl esterase		1.43	9.08E-02	1.05	6.07E-01	1.16	2.89E-01
MSMEG_3003	aspartyl-tRNA synthetase	*aspS*	0.51	3.92E-05	0.86	8.82E-02	1.38	2.31E-02
MSMEG_3004	conserved hypothetical protein TIGR00026		1.95	3.03E-03	0.80	2.22E-01	1.81	2.21E-02
MSMEG_3005	hydroxyacyl-CoA dehydrogenase		4.41	1.58E-02	2.27	1.12E-01	1.39	9.94E-02

MSMEG_3006	Fe-dependent alcohol dehydrogenase		6.29	7.64E-03	2.05	1.32E-01	1.21	2.29E-01
MSMEG_3007	succinate-semialdehyde dehydrogenase		5.40	1.20E-03	2.82	9.87E-02	3.33	1.61E-02
MSMEG_3008	putative sigma 54 type regulator		4.51	3.30E-03	1.17	1.05E-01	0.67	4.22E-03
MSMEG_3009	2,5-dichloro-2,5-cyclohexadiene-1,4-diol dehydrogenase		1.04	7.05E-01	7.32	1.45E-01	1.23	5.34E-01
MSMEG_3010	acetyl-hydrolase		1.43	4.94E-01	1.06	3.55E-01	1.05	6.20E-01
MSMEG_3011	steroid monooxygenase		1.25	3.51E-02	1.14	3.97E-01	1.31	2.81E-01
MSMEG_3012	acetyl-CoA acetyltransferases		1.33	3.43E-01	20.96	8.21E-02	1.84	3.83E-01
MSMEG_3013	putative transcriptional regulator family protein		1.26	1.36E-01	0.92	2.58E-01	1.09	2.61E-01
MSMEG_3014	conserved hypothetical protein		6.17	8.52E-04	0.95	6.03E-01	1.02	8.48E-01
MSMEG_3015	conserved hypothetical protein		0.61	1.59E-02	0.96	8.35E-01	3.93	1.96E-02
MSMEG_3016	conserved hypothetical protein		1.15	7.37E-01	1.35	2.03E-01	1.05	7.85E-01
MSMEG_3017	conserved hypothetical protein		0.74	8.88E-03	1.08	3.83E-01	3.73	1.21E-01
MSMEG_3018	transglutaminase domain protein		0.75	5.21E-03	0.76	6.86E-02	1.77	1.73E-01
MSMEG_3019	conserved hypothetical protein		0.72	3.40E-02	1.02	8.47E-01	1.40	1.32E-01
MSMEG_3020	conserved hypothetical protein		0.57	1.26E-02	0.76	4.60E-02	1.88	2.29E-01
MSMEG_3021	AAA ATPase, central region		0.81	1.24E-01	1.03	5.49E-01	1.69	1.18E-02
MSMEG_3022	transglycosylase associated protein		2.66	2.84E-01	2.53	4.05E-02	0.48	9.62E-04
MSMEG_3023	conserved hypothetical protein; this gene contains a frame shift which is not the result of sequencing error		4.52	1.55E-03	0.98	8.90E-01	0.78	1.37E-01
MSMEG_3024	conserved hypothetical protein		1.05	7.31E-01	0.84	1.70E-01	2.22	2.93E-02
MSMEG_3025	alanyl-tRNA synthetase	*alaS*	0.60	2.03E-04	1.12	7.68E-03	1.82	7.78E-03
MSMEG_3026	conserved hypothetical protein		0.70	4.75E-04	0.93	4.73E-02	2.58	1.60E-02
MSMEG_3027	conserved hypothetical protein		0.50	6.69E-05	0.93	3.78E-01	2.67	1.04E-02
MSMEG_3028	shikimate-5-dehydrogenase	*aroE*	0.41	5.49E-06	0.85	5.08E-03	3.29	8.69E-02
MSMEG_3029	peptidase, A24 (type IV prepilin peptidase) family protein		0.82	2.94E-01	1.07	5.07E-01	1.16	5.67E-01
MSMEG_3030	chorismate synthase	*aroC*	0.74	3.07E-04	1.01	9.05E-01	2.03	1.31E-02
MSMEG_3031	shikimate kinase		0.94	1.16E-01	0.91	1.68E-02	1.86	2.43E-02
MSMEG_3032	conserved hypothetical protein		0.83	3.59E-03	1.03	7.73E-01	0.20	6.63E-06
MSMEG_3033	3-dehydroquinate synthase	*aroB*	0.68	1.80E-01	1.05	7.78E-01	2.08	1.67E-01
MSMEG_3034	metallopeptidase, M24 family protein		0.71	1.25E-02	1.08	9.06E-02	1.52	2.73E-02
MSMEG_3035	translation elongation factor P	*efp*	0.52	1.32E-04	1.01	8.42E-01	0.80	1.99E-02
MSMEG_3036	transcription antitermination factor NusB	*nusB*	0.31	1.32E-05	0.96	5.03E-01	1.28	2.36E-02

MSMEG_3037	regulatory protein; this gene contains a frame shift which is not the result of sequencing error; identified by match to protein family HMM PF00196		2.13	9.30E-03	1.24	3.57E-02	1.47	1.20E-01
MSMEG_3038	hypothetical protein		0.85	4.04E-03	0.82	9.88E-02	0.91	3.30E-01
MSMEG_3039	monooxygenase		1.20	1.90E-01	1.22	2.09E-01	0.51	1.08E-02
MSMEG_3040	beta-lactamase		0.56	3.27E-03	0.87	3.56E-02	0.89	4.82E-01
MSMEG_3041	thiopurine S-methyltransferase (tpmt) superfamily protein		2.55	2.22E-02	0.99	9.48E-01	1.20	3.67E-01
MSMEG_3042	PyrR bifunctional protein		0.68	9.40E-03	0.97	5.94E-01	1.96	3.75E-02
MSMEG_3043	aspartate carbamoyltransferase	*pyrB*	0.51	6.58E-05	1.03	8.35E-01	2.76	2.69E-03
MSMEG_3044	dihydroorotase		0.45	3.70E-04	0.99	9.75E-01	3.13	8.88E-04
MSMEG_3045	integral membrane protein		0.44	7.13E-05	1.22	3.19E-01	2.67	1.78E-01
MSMEG_3046	carbamoyl-phosphate synthase, small subunit	*carA*	0.42	6.53E-05	1.31	2.01E-01	1.56	1.60E-03
MSMEG_3047	carbamoyl-phosphate synthase, large subunit	*carB*	0.33	4.52E-05	1.47	2.82E-01	1.75	2.73E-02
MSMEG_3048	orotidine 5'-phosphate decarboxylase	*pyrF*	0.26	2.96E-05	0.82	1.23E-01	2.74	2.03E-02
MSMEG_3049	hypothetical protein		1.18	5.49E-01	1.06	6.62E-01	1.22	4.35E-01
MSMEG_3050	integration host factor		1.06	5.24E-01	0.72	1.24E-01	0.91	6.56E-01
MSMEG_3051	guanylate kinase		1.03	6.70E-01	0.90	2.56E-01	0.67	1.11E-02
MSMEG_3052	hypothetical protein		1.11	3.64E-01	0.98	9.11E-01	1.24	1.12E-01
MSMEG_3053	DNA-directed RNA polymerase, omega subunit	*rpoZ*	1.32	6.30E-04	0.80	2.41E-03	1.51	5.33E-04
MSMEG_3054	phosphopantothenoylcysteine decarboxylase/phosphopantothenate--cysteine ligase	*coaBC*	0.96	5.24E-01	0.92	1.85E-01	1.62	5.38E-02
MSMEG_3055	S-adenosylmethionine synthetase	*metK*	0.57	2.99E-05	1.25	1.55E-01	1.69	3.50E-02
MSMEG_3056	ABC transporter ATP-binding protein		0.58	2.52E-03	1.78	1.81E-01	2.64	8.24E-03
MSMEG_3057	ABC-type metal ion transport system, permease component		0.35	2.46E-04	1.39	3.49E-01	1.91	3.78E-02
MSMEG_3058	lipoprotein, nlpa family protein		0.33	8.71E-05	1.28	3.24E-01	1.49	2.16E-01
MSMEG_3058	lipoprotein, nlpa family protein		0.36	1.68E-04	1.13	5.61E-01	1.16	4.54E-01
MSMEG_3059	esterase		0.63	6.16E-04	0.98	8.45E-01	1.33	1.12E-01
MSMEG_3060	conserved hypothetical protein		1.01	8.61E-01	0.87	5.71E-02	1.17	2.53E-01
MSMEG_3061	primosomal protein N'	*priA*	0.67	5.87E-04	0.92	1.61E-02	1.42	3.71E-01
MSMEG_3062	conserved hypothetical protein		1.00	9.69E-01	1.09	6.16E-01	1.29	2.10E-01
MSMEG_3063	LemA protein		1.25	1.06E-01	0.87	1.15E-01	0.98	8.80E-01
MSMEG_3064	methionyl-tRNA formyltransferase	*fmt*	1.33	6.36E-01	0.97	1.25E-01	2.18	1.11E-01
MSMEG_3065	ribosomal RNA small subunit methyltransferase B	*sun*	0.54	4.47E-04	0.88	3.13E-02	2.00	7.00E-04

MSMEG_3066	ribulose-phosphate 3-epimerase	*rpe*	0.73	1.35E-02	0.83	1.79E-03	1.23	1.25E-01
MSMEG_3067	riboflavin biosynthesis protein RibD	*ribD*	1.75	4.33E-01	0.87	7.47E-03	1.96	1.02E-01
MSMEG_3068	hypothetical protein		1.84	1.42E-02	0.56	4.43E-03	3.27	1.53E-03
MSMEG_3069	aminoglycosides/tetracycline-transport integral membrane protein		0.49	7.09E-05	1.04	3.52E-01	1.55	2.15E-01
MSMEG_3070	LprG protein		0.70	5.78E-05	1.05	1.89E-01	1.56	5.03E-02
MSMEG_3071	riboflavin synthase, alpha subunit	*ribE*	0.42	1.32E-05	0.97	7.01E-01	3.88	4.30E-02
MSMEG_3072	riboflavin biosynthesis protein ribAB		0.73	6.12E-06	0.89	1.32E-02	2.04	7.47E-03
MSMEG_3073	6,7-dimethyl-8-ribityllumazine synthase	*ribE*	0.64	1.39E-05	0.88	2.33E-01	4.09	1.47E-02
MSMEG_3074	conserved hypothetical protein		0.47	5.89E-05	0.90	1.32E-01	4.51	8.43E-03
MSMEG_3075	acetyltransferase, GNAT family protein		1.27	3.02E-02	1.04	6.64E-01	1.56	4.37E-02
MSMEG_3076	gamma-glutamyltransferase	*ggt*	1.61	1.20E-03	1.20	1.09E-01	1.22	2.02E-01
MSMEG_3077	hypothetical protein		1.05	7.80E-01	1.44	3.67E-01	0.97	3.98E-01
MSMEG_3078	excinuclease ABC, C subunit	*uvrC*	1.24	2.81E-02	0.81	2.64E-02	6.52	2.58E-02
MSMEG_3079	conserved hypothetical protein		1.26	1.50E-02	0.85	2.43E-01	3.61	2.26E-02
MSMEG_3080	conserved hypothetical protein		1.04	3.69E-01	0.75	5.86E-03	3.67	8.27E-03
MSMEG_3081	conserved hypothetical protein		0.75	2.59E-01	0.86	1.08E-02	2.65	1.66E-02
MSMEG_3082	soul heme-binding protein		0.89	3.63E-01	1.14	6.52E-02	1.69	9.14E-02
MSMEG_3083	nucleoside-diphosphate sugar epimerase		1.12	1.75E-01	1.28	5.30E-02	1.11	3.64E-01
MSMEG_3084	glyceraldehyde-3-phosphate dehydrogenase, type	*gap*	0.38	4.56E-05	0.98	8.04E-01	1.14	5.87E-01
MSMEG_3085	phosphoglycerate kinase	*pgk*	0.32	9.62E-06	1.04	8.13E-01	1.58	6.47E-02
MSMEG_3086	triosephosphate isomerase	*tpiA*	0.35	1.08E-05	0.99	9.31E-01	1.93	3.70E-02
MSMEG_3087	preprotein translocase, SecG subunit	*secG*	0.50	1.16E-04	0.83	5.70E-02	2.27	9.10E-03
MSMEG_3088	O-methyltransferase; this gene contains a premature stop which is not the result of sequencing error		1.74	1.06E-02	1.09	1.12E-01	1.17	1.37E-01
MSMEG_3088	O-methyltransferase; this gene contains a premature stop which is not the result of sequencing error		1.10	5.98E-01	0.89	3.19E-01	1.09	2.61E-01
MSMEG_3089	deoxyribose-phosphate aldolase	*deoC*	1.34	3.09E-01	0.86	1.08E-01	0.98	6.09E-01
MSMEG_3090	ribose transport system permease protein RbsC		1.00	9.80E-01	0.97	7.44E-01	0.75	1.67E-01
MSMEG_3091	ribose transport ATP-binding protein RbsA		1.24	2.27E-01	0.97	6.85E-01	0.98	6.34E-01
MSMEG_3092	transcriptional regulator, sugar-binding family protein		0.97	4.01E-01	1.34	9.23E-02	1.04	1.66E-01
MSMEG_3093	putative sugar kinase protein		1.64	3.88E-01	0.98	8.58E-01	0.95	6.96E-01
MSMEG_3094	oxidoreductase, zinc-binding dehydrogenase family protein		1.07	8.66E-02	1.05	5.87E-01	0.55	3.20E-02
MSMEG_3095	D-ribose-binding periplasmic protein		1.63	2.53E-03	0.87	2.68E-01	0.78	2.67E-01

MSMEG_3096	hypothetical protein		1.48	7.30E-02	1.06	8.14E-01	1.57	2.67E-01
MSMEG_3097	phosphoenolpyruvate carboxylase	*ppc*	0.61	1.16E-03	0.95	6.08E-01	1.49	2.96E-03
MSMEG_3098	conserved hypothetical protein		0.74	8.93E-02	1.21	2.95E-02	0.85	1.74E-01
MSMEG_3099	6-phosphogluconolactonase	*pgl*	0.71	1.32E-03	1.04	6.80E-01	0.89	8.21E-02
MSMEG_3100	OpcA protein	*opcA*	0.68	5.25E-04	1.13	2.50E-01	1.04	7.47E-01
MSMEG_3101	glucose-6-phosphate 1-dehydrogenase	*zwf*	0.59	1.35E-04	1.12	1.71E-01	1.10	1.90E-01
MSMEG_3102	transaldolase	*tal*	0.93	3.83E-01	1.16	4.05E-02	0.89	3.14E-01
MSMEG_3103	transketolase	*tkt*	0.88	1.39E-01	0.81	1.61E-02	0.64	6.75E-04
MSMEG_3104	hypothetical protein		0.83	3.30E-01	1.20	5.22E-02	1.51	1.65E-01
MSMEG_3105	protoheme IX farnesyltransferase	*cyoE*	0.44	5.58E-06	0.80	7.02E-02	3.69	2.73E-02
MSMEG_3106	quinone oxidoreductase		1.01	8.94E-01	0.77	2.39E-02	1.39	2.92E-02
MSMEG_3107	conserved hypothetical protein		0.62	1.22E-03	0.90	2.19E-01	1.28	3.73E-01
MSMEG_3108	ABC transporter, ATPase subunit		0.86	6.28E-02	1.00	9.84E-01	1.03	7.07E-01
MSMEG_3109	binding-protein-dependent transport systems inner membrane component		0.94	6.70E-01	1.17	7.79E-02	1.28	1.78E-01
MSMEG_3110	binding-protein-dependent transport systems inner membrane component		1.09	4.79E-01	1.11	5.03E-01	0.99	8.05E-01
MSMEG_3111	extracellular solute-binding protein, family protein 1		0.89	2.61E-01	0.94	2.88E-01	0.84	1.98E-01
MSMEG_3112	estradiol 17-beta-dehydrogenase 8		0.70	5.43E-03	0.87	3.84E-02	1.30	1.81E-01
MSMEG_3113	carbohydrate kinase, fggy		1.44	1.91E-02	1.09	2.36E-01	1.01	6.73E-01
MSMEG_3114	conserved hypothetical protein		0.71	5.15E-02	1.17	3.88E-01	0.93	2.53E-01
MSMEG_3115	regulatory protein, DeoR		0.78	8.35E-02	1.20	3.20E-01	0.86	3.44E-01
MSMEG_3116	inositol-1-monophosphatase		1.42	3.33E-01	1.03	8.68E-01	1.52	3.31E-01
MSMEG_3117	cytochrome aa3 controlling protein		0.33	1.39E-04	1.03	8.69E-01	11.96	9.60E-03
MSMEG_3118	ABC transporter efflux protein, DrrB family protein		0.59	9.56E-03	1.36	4.82E-01	1.36	4.55E-02
MSMEG_3119	ABC transporter, ATP-binding subunit		0.50	3.61E-04	1.01	8.42E-01	1.77	4.61E-02
MSMEG_3120	conserved hypothetical protein		0.63	1.97E-03	0.94	5.67E-01	1.95	3.15E-01
MSMEG_3121	DNA-binding protein		1.38	1.47E-03	0.55	1.74E-04	0.77	2.49E-02
MSMEG_3122	FeS assembly protein SufB	*sufB*	1.18	1.05E-01	0.70	7.20E-03	0.79	1.67E-02
MSMEG_3123	FeS assembly protein SufD	*sufD*	1.23	8.89E-02	0.60	1.87E-03	0.82	8.76E-03
MSMEG_3124	FeS assembly ATPase SufC	*sufC*	1.03	6.80E-01	0.52	4.30E-04	0.77	9.48E-03
MSMEG_3125	cysteine desulfurase		0.89	3.52E-01	0.58	2.96E-04	1.40	6.84E-03
MSMEG_3126	SUF system FeS assembly protein, NifU family protein		0.85	5.34E-02	0.61	3.42E-04	1.17	1.36E-01
MSMEG_3127	conserved protein, DUF59		0.93	1.12E-01	0.66	4.44E-04	0.93	2.29E-01
MSMEG_3128	hypothetical protein		0.76	2.99E-02	1.15	3.60E-01	1.08	4.38E-01

MSMEG_3129	putative HTH-type transcriptional regulator		1.10	1.73E-01	1.08	2.30E-01	0.96	8.60E-01
MSMEG_3130	hypothetical protein		0.95	7.16E-01	1.21	2.98E-01	1.05	8.40E-01
MSMEG_3131	AMP-binding protein		0.87	2.41E-01	1.30	1.08E-01	1.02	1.86E-01
MSMEG_3132	DNA-binding protein		1.54	2.24E-02	1.09	3.03E-01	1.19	1.63E-01
MSMEG_3133	hypothetical protein		1.29	3.42E-01	1.20	2.94E-01	0.94	3.69E-01
MSMEG_3134	cytochrome P450 107B1		1.72	2.50E-02	1.19	5.46E-01	0.81	3.74E-01
MSMEG_3135	hypothetical protein		1.29	2.10E-01	1.02	7.80E-01	1.05	5.90E-01
MSMEG_3136	heme peroxidase superfamily protein		1.35	2.31E-01	1.00	9.81E-01	0.87	3.45E-01
MSMEG_3137	oxidoreductase		2.70	2.34E-03	2.01	2.09E-01	0.90	2.18E-01
MSMEG_3138	thioredoxin	*trx*	2.50	5.59E-03	1.02	8.95E-01	0.57	6.86E-05
MSMEG_3139	enoyl-CoA hydratase/isomerase		0.52	2.92E-03	7.49	7.68E-02	1.55	9.83E-02
MSMEG_3140	ABC transporter ATP-binding protein, possibly in EF-3 subfamily protein		0.56	3.00E-04	3.05	1.37E-01	4.43	7.33E-03
MSMEG_3141	conserved domain protein		3.72	3.04E-02	3.38	8.48E-02	0.46	2.03E-02
MSMEG_3142	HTH-type transcriptional repressor AcnR		0.66	9.55E-04	1.14	3.29E-01	1.90	4.58E-04
MSMEG_3143	aconitate hydratase 1	*acnA*	0.90	2.06E-02	1.46	1.55E-01	1.48	1.01E-03
MSMEG_3144	putative membrane protein		0.66	9.01E-02	1.42	2.28E-01	1.00	9.91E-01
MSMEG_3145	secreted cell wall-associated hydrolase		0.94	6.54E-01	1.03	8.31E-01	0.70	4.60E-02
MSMEG_3146	invasin 1		0.68	2.00E-02	1.19	5.13E-01	1.11	6.89E-01
MSMEG_3147	ATPase, MoxR family protein	*moxR*	1.00	9.96E-01	0.85	9.99E-02	1.28	3.18E-01
MSMEG_3148	conserved hypothetical protein		0.65	5.76E-04	1.00	9.63E-01	1.70	3.00E-03
MSMEG_3149	conserved hypothetical protein		0.46	2.85E-06	1.12	2.76E-01	1.42	3.73E-03
MSMEG_3150	3-oxoacyl-(acyl-carrier-protein) reductase	*fabG*	0.38	2.06E-07	1.11	2.78E-01	1.66	6.22E-02
MSMEG_3151	[NADH] enoyl-[acyl-carrier-protein] reductase		0.36	2.14E-05	0.95	5.32E-01	2.29	1.45E-03
MSMEG_3152	ferrochelatase	*hemH*	0.25	3.38E-07	1.02	8.72E-01	2.46	1.21E-02
MSMEG_3153	conserved hypothetical protein		1.35	5.02E-03	0.93	4.40E-01	0.92	6.51E-01
MSMEG_3154	membrane protein implicated in regulation of membrane protease activity		1.58	3.87E-03	1.01	9.35E-01	0.76	9.17E-02
MSMEG_3155	band 7 protein		1.21	1.94E-03	1.04	5.35E-01	0.49	3.54E-04
MSMEG_3156	conserved hypothetical protein		0.95	6.11E-01	1.10	3.33E-01	1.10	3.37E-01
MSMEG_3157	conserved hypothetical protein		0.82	5.83E-03	0.83	5.99E-02	1.42	1.33E-01
MSMEG_3158	methylmalonyl-CoA mutase, small subunit	*mutA*	0.49	5.15E-03	1.10	3.41E-01	0.99	9.59E-01
MSMEG_3159	methylmalonyl-CoA mutase large subunit		0.40	1.35E-04	1.07	4.54E-01	0.81	1.53E-01
MSMEG_3160	LAO/AO transport system ATPase		0.39	6.77E-06	1.17	2.75E-01	1.12	6.69E-01
MSMEG_3161	conserved hypothetical protein		0.19	1.57E-05	1.18	1.06E-01	0.99	8.92E-01
MSMEG_3162	beta-lactamase		4.60	9.01E-04	0.81	8.93E-03	0.22	7.08E-04
MSMEG_3163	gp55 protein		1.17	3.03E-01	0.92	4.38E-01	0.32	2.49E-04
MSMEG_3164	hypothetical protein		1.76	2.26E-02	1.29	9.85E-03	0.22	2.54E-05

MSMEG_3168	phenolpthIocerol synthesis type-i polyketide synthase ppse		0.97	6.81E-01	1.10	4.85E-01	0.90	2.50E-01
MSMEG_3169	isoleucyl-tRNA synthetase	*ileS*	0.33	4.36E-06	1.12	1.58E-01	1.74	7.84E-03
MSMEG_3170	conserved domain protein		1.07	4.53E-01	1.09	6.34E-01	1.99	8.06E-02
MSMEG_3171	conserved hypothetical protein		7.90	1.15E-04	0.75	9.09E-02	0.75	4.49E-02
MSMEG_3172	DNA polymerase IV 1		0.87	2.34E-02	0.98	8.46E-01	1.77	9.94E-02
MSMEG_3173	L-asparaginase		0.75	4.12E-02	1.00	9.50E-01	2.05	2.27E-02
MSMEG_3174	lipoprotein signal peptidase		0.94	3.10E-01	0.81	3.79E-02	1.74	2.56E-02
MSMEG_3175	ribosomal large subunit pseudouridine synthase D		0.62	5.30E-04	1.01	7.76E-01	2.56	5.32E-02
MSMEG_3176	RarD protein	*rarD*	0.48	1.17E-05	0.93	5.17E-01	2.43	1.40E-02
MSMEG_3177	probable transcriptional regulatory protein, putative		0.89	4.33E-01	1.34	1.33E-01	1.34	3.23E-01
MSMEG_3178	DNA polymerase III alpha subunit		0.91	1.59E-01	0.99	8.54E-01	1.59	1.07E-01
MSMEG_3179	pyridoxamine 5'-phosphate oxidase family		1.15	4.05E-01	1.26	1.54E-02	0.97	4.49E-01
MSMEG_3180	transcriptional regulator, MerR family protein		1.92	3.90E-06	1.18	1.19E-01	0.36	2.16E-04
MSMEG_3181	conserved hypothetical protein		2.18	1.86E-04	0.87	5.11E-02	1.23	1.08E-01
MSMEG_3182	hypothetical protein		1.09	6.41E-01	1.23	3.21E-01	1.15	2.22E-01
MSMEG_3183	threonine dehydratase	*ilvA*	1.23	8.92E-02	1.15	6.15E-02	1.53	8.93E-02
MSMEG_3184	malto-oligosyltrehalose trehalohydrolase	*treZ*	0.88	3.15E-01	1.31	4.92E-02	1.03	8.41E-01
MSMEG_3185	putative maltooligosyl trehalose synthase; this gene contains a frame shift which is not the result of sequencing error; identified by match to protein family HMM PF00128		0.65	1.11E-02	1.55	1.83E-02	1.39	3.75E-01
MSMEG_3185	putative maltooligosyl trehalose synthase; this gene contains a frame shift which is not the result of sequencing error; identified by match to protein family HMM PF00128		0.74	5.48E-02	1.33	6.22E-02	1.05	8.40E-01
MSMEG_3186	glycogen debranching enzyme GlgX	*glgX*	0.73	9.64E-03	1.54	6.86E-02	0.84	5.13E-01
MSMEG_3187	putative acyltransferase domain protein		0.88	7.74E-02	0.92	3.38E-01	1.41	1.05E-03
MSMEG_3188	adenosylmethionine-8-amino-7-oxononanoate transaminase	*bioA*	0.47	2.59E-05	1.12	5.90E-01	1.23	2.17E-01
MSMEG_3189	8-amino-7-oxononanoate synthase	*bioF*	0.41	8.56E-04	1.19	3.46E-01	1.10	3.45E-01
MSMEG_3190	dethiobiotin synthase	*bioD*	0.87	4.09E-01	1.30	9.56E-02	1.36	1.23E-01
MSMEG_3191	conserved hypothetical protein		1.45	1.75E-01	1.13	1.72E-01	1.37	3.37E-01
MSMEG_3192	conserved hypothetical protein, putative		2.05	1.96E-03	0.95	1.18E-01	1.01	9.37E-01
MSMEG_3193	transcriptional regulator, TetR family protein		0.66	4.45E-04	0.94	6.44E-01	1.06	7.19E-01
MSMEG_3194	biotin synthase	*bioB*	0.49	6.00E-03	1.16	2.27E-01	1.23	3.38E-01

MSMEG_3195	conserved hypothetical protein		0.76	5.13E-04	0.83	6.40E-02	1.49	1.33E-01
MSMEG_3196	conserved hypothetical protein		0.91	1.51E-01	0.88	2.57E-01	1.50	2.50E-02
MSMEG_3197	lipase		1.21	8.32E-02	0.60	8.44E-03	0.71	8.19E-02
MSMEG_3198	hydrolase, NUDIX family protein		0.62	4.94E-03	1.01	9.02E-01	1.30	1.16E-01
MSMEG_3199	quinolinate synthetase complex, A subunit	*nadA*	0.66	1.19E-02	1.11	3.29E-01	0.71	8.84E-03
MSMEG_3200	L-aspartate oxidase	*nadB*	0.71	2.13E-03	1.09	5.65E-01	1.11	2.54E-01
MSMEG_3201	nicotinate-nucleotide pyrophosphorylase	*nadC*	0.94	5.89E-01	1.10	4.09E-01	1.06	7.98E-01
MSMEG_3202	hypothetical protein		1.24	5.11E-01	1.04	6.70E-01	1.03	7.94E-01
MSMEG_3203	transporter, LysE family protein		0.81	4.75E-02	1.06	6.64E-01	0.98	5.79E-01
MSMEG_3204	conserved hypothetical protein		1.84	9.98E-06	0.73	2.25E-02	0.71	1.24E-01
MSMEG_3205	histidinol dehydrogenase	*hisD*	1.00	9.85E-01	0.99	8.45E-01	1.15	4.68E-01
MSMEG_3206	histidinol-phosphate aminotransferase	*hisC*	0.94	7.84E-01	0.80	2.89E-03	1.35	7.18E-03
MSMEG_3207	imidazoleglycerol-phosphate dehydratase	*hisB*	0.96	5.28E-01	0.89	2.98E-01	1.72	1.36E-01
MSMEG_3208	imidazole glycerol phosphate synthase, glutamine amidotransferase subunit	*hisH*	0.73	1.17E-02	0.98	5.72E-01	2.61	2.46E-04
MSMEG_3209	bifunctional HisA/TrpF protein		0.71	3.88E-02	0.74	4.29E-02	1.92	1.01E-02
MSMEG_3210	inositol monophosphate phosphatase		0.46	8.33E-05	0.94	2.49E-01	2.16	2.22E-02
MSMEG_3211	imidazoleglycerol phosphate synthase, cyclase subunit	*hisF*	0.58	5.10E-04	0.93	3.82E-01	2.11	1.03E-01
MSMEG_3212	phosphoribosyl-AMP pyrophosphatase/phosphoribosyl-ATP cyclohydrolase	*hisIE*	0.52	1.05E-04	1.00	9.36E-01	1.83	5.07E-03
MSMEG_3213	conserved hypothetical protein		0.65	1.37E-02	1.00	9.85E-01	1.18	7.37E-02
MSMEG_3214	hypothetical protein		0.62	3.63E-03	0.92	5.87E-01	1.99	2.74E-01
MSMEG_3215	ABC-type molybdenum transport system, ATPase component/photorepair protein PhrA		0.84	3.33E-02	0.83	1.38E-01	1.03	8.71E-01
MSMEG_3216	peroxiredoxin Q		0.64	1.56E-03	1.06	6.24E-01	1.52	9.95E-02
MSMEG_3217	anthranilate synthase component I	*trpE*	0.62	5.67E-04	0.86	4.44E-02	1.61	3.70E-04
MSMEG_3218	trp region conserved hypothetical membrane protein		0.47	8.76E-05	1.06	6.51E-01	1.26	1.87E-01
MSMEG_3219	indole-3-glycerol phosphate synthase	*trpC*	0.85	1.38E-01	0.74	3.14E-03	1.34	3.54E-03
MSMEG_3220	tryptophan synthase, beta subunit	*trpB*	0.58	2.81E-03	0.92	3.90E-01	1.98	9.70E-04
MSMEG_3221	tryptophan synthase, alpha subunit	*trpA*	0.55	2.26E-04	0.84	3.86E-02	2.63	7.90E-03
MSMEG_3222	prolipoprotein diacylglyceryl transferase		0.39	2.40E-06	1.13	2.78E-01	2.34	6.52E-03
MSMEG_3223	TM2 domain protein		1.71	6.60E-03	0.85	9.24E-02	0.97	9.07E-01
MSMEG_3224	conserved hypothetical protein		1.40	7.45E-02	0.69	2.02E-02	1.23	5.68E-01
MSMEG_3225	ferredoxin-dependent glutamate synthase 1		0.50	1.65E-02	1.15	4.67E-01	1.06	8.48E-01
MSMEG_3226	glutamate synthase, NADH/nadph, small subunit		0.87	4.40E-01	1.01	9.45E-01	1.87	1.90E-01

MSMEG_3227	pyruvate kinase	*pyk*	0.87	6.40E-02	1.08	5.21E-01	0.86	2.46E-01
MSMEG_3228	acyl-CoA thioesterase II		0.44	1.65E-04	1.25	3.39E-02	2.07	8.44E-02
MSMEG_3229	conserved hypothetical protein		2.12	6.95E-03	0.91	4.04E-01	5.20	1.41E-03
MSMEG_3230	ABC transporter, CydDC cysteine exporter (CydDC-E) family, permease/ATP-binding protein CydC; this gene contains a frame shift which is not the result of sequencing error; identified by match to protein family HMM PF00005; match to protein family HMM TIGR02868		0.52	1.12E-02	1.01	9.29E-01	93.33	2.36E-02
MSMEG_3231	ABC transporter, CydDC cysteine exporter (CydDC-E) family protein, permease/ATP-binding protein CydD	*cydD*	0.52	2.20E-02	1.08	5.85E-01	62.22	5.79E-03
MSMEG_3232	cytochrome D ubiquinol oxidase, subunit II	*cydB*	0.23	4.15E-06	1.03	6.99E-01	159.29	2.89E-02
MSMEG_3233	cytochrome D ubiquinol oxidase subunit 1		0.34	2.26E-05	0.90	2.51E-01	48.86	6.01E-02
MSMEG_3234	integral membrane protein		1.80	3.26E-03	0.84	6.18E-02	0.59	1.68E-03
MSMEG_3235	ABC-type amino acid transport system, secreted component		0.57	1.71E-05	1.10	2.83E-01	0.31	3.55E-04
MSMEG_3236	ABC-type amino acid transport system, permease component		0.67	4.48E-04	1.03	2.31E-01	0.26	8.89E-06
MSMEG_3237	ATP-binding protein		0.63	3.38E-03	0.89	2.27E-01	0.41	1.26E-04
MSMEG_3238	3-mercaptopyruvate sulfurtransferase		1.04	6.96E-01	0.65	1.24E-02	1.30	2.29E-02
MSMEG_3239	two-component system sensor kinase		1.26	2.07E-02	0.96	5.29E-01	1.63	1.63E-03
MSMEG_3240	DNA-binding response regulator, LuxR family protein		1.17	2.94E-01	0.93	4.66E-01	1.34	7.96E-03
MSMEG_3241	conserved hypothetical protein		0.96	4.27E-01	1.02	7.43E-01	1.17	3.80E-01
MSMEG_3242	starvation-inducible DNA-binding protein or fine tangled pili major subunit		1.39	1.86E-01	20.89	1.25E-01	0.64	3.58E-02
MSMEG_3243	adenylate cyclase; this gene contains a frame shift which is not the result of sequencing error; identified by match to protein family HMM PF00211		1.24	2.98E-01	1.12	4.90E-01	1.27	4.42E-01
MSMEG_3244	hypothetical protein		2.28	3.15E-04	4.74	8.27E-02	1.57	5.45E-02
MSMEG_3246	response regulator		1.11	2.14E-01	1.35	9.36E-02	1.43	8.95E-02
MSMEG_3247	branched-chain amino acid ABC transporter substrate-binding protein		0.43	1.66E-05	1.43	5.13E-02	0.52	7.68E-03
MSMEG_3248	ABC transporter branched chain amino acid transport permease		0.25	1.23E-06	1.18	5.17E-01	0.65	1.39E-01

MSMEG_3249	branched-chain amino acid ABC transporter, permease protein		0.24	6.63E-07	1.39	2.09E-01	0.76	4.07E-01
MSMEG_3250	ABC transporter, ATP-binding protein		0.34	1.90E-06	1.25	3.03E-01	0.73	2.87E-01
MSMEG_3251	branched-chain amino acid ABC transporter ATP-binding protein		0.31	1.05E-07	1.23	2.64E-01	0.56	3.56E-02
MSMEG_3252	membrane protein		0.83	1.86E-01	1.24	2.36E-01	1.43	4.25E-01
MSMEG_3253	conserved hypothetical protein		0.74	1.11E-03	1.09	7.06E-01	3.29	5.68E-02
MSMEG_3254	RDD family protein, putative		2.36	6.22E-02	1.81	8.23E-02	0.64	9.76E-03
MSMEG_3255	DoxX subfamily protein, putative		2.67	2.00E-01	3.57	2.54E-02	0.34	2.18E-04
MSMEG_3256	mucin-associated surface protein		0.51	4.70E-03	1.27	1.86E-01	1.26	2.54E-01
MSMEG_3257	xylulokinase	*xylB*	0.81	1.54E-02	0.91	4.50E-02	1.01	9.27E-01
MSMEG_3258	hypothetical protein		0.72	1.39E-03	0.83	1.95E-02	3.77	1.97E-01
MSMEG_3259	acyl-CoA dehydrogenase		0.67	1.56E-04	0.96	7.54E-01	2.55	9.32E-03
MSMEG_3260	LysR-family protein transcriptional regulator		1.71	4.34E-02	1.13	8.22E-02	1.17	3.91E-01
MSMEG_3261	conserved hypothetical protein		0.84	5.18E-02	0.87	1.17E-01	1.18	1.85E-01
MSMEG_3262	L-xylulose reductase		1.53	3.88E-02	0.77	2.86E-02	1.22	2.95E-01
MSMEG_3263	xylulose kinase		0.91	3.40E-01	0.99	9.08E-01	1.76	3.09E-03
MSMEG_3264	transcriptional regulator		1.84	2.70E-02	1.00	9.70E-01	1.08	7.07E-01
MSMEG_3265	arabitol-phosphate dehydrogenase		1.21	1.97E-02	1.44	2.87E-01	0.88	4.95E-01
MSMEG_3266	maltose/maltodextrin-binding protein		1.63	5.21E-02	1.30	5.61E-02	1.06	7.10E-01
MSMEG_3267	transporter		1.30	4.29E-02	1.19	3.89E-01	1.20	2.94E-01
MSMEG_3268	ABC transporter, permease protein		1.17	4.15E-01	1.08	4.13E-01	1.11	7.74E-01
MSMEG_3269	putative sugar ABC transporter ATP-binding protein		1.19	2.07E-01	1.31	2.41E-01	1.21	1.70E-01
MSMEG_3270	SN-glycerol-3-phosphate ABC transporter, ATP-binding protein		1.27	1.76E-01	1.15	3.14E-01	0.98	8.04E-01
MSMEG_3271	dihydroxyacetone kinase		1.15	3.77E-01	1.17	3.11E-01	1.38	8.89E-02
MSMEG_3272	ribose 5-phosphate isomerase		0.83	2.51E-03	0.94	4.21E-01	1.30	1.92E-02
MSMEG_3273	glutamyl aminopeptidase, M42 family protein		1.03	8.47E-01	1.50	1.49E-01	1.07	6.94E-01
MSMEG_3274	MerR-family protein transcriptional regulator		1.74	1.39E-01	1.09	4.45E-01	1.29	2.13E-01
MSMEG_3275	RNA polymerase sigma factor, sigma-70 family protein		2.04	3.28E-01	6.35	1.53E-01	0.99	4.35E-01
MSMEG_3276	integral membrane protein		0.58	5.70E-03	2.42	1.67E-01	1.01	2.78E-01
MSMEG_3277	polyamine ABC transporter integral membrane protein		0.47	5.29E-06	1.22	3.17E-01	0.38	6.57E-04
MSMEG_3278	conserved hypothetical protein		1.68	1.23E-02	0.88	1.40E-01	0.75	1.82E-02
MSMEG_3279	polyamine ABC transporter permease protein		0.52	2.91E-04	1.83	2.18E-01	0.67	5.59E-02
MSMEG_3280	polyamine-binding lipoprotein		0.55	1.71E-04	1.12	2.00E-01	0.28	4.78E-05

MSMEG_3281	spermidine/putrescine ABC transporter ATP-binding subunit	0.76	5.93E-02	1.07	4.15E-01	0.32	2.18E-05
MSMEG_3282	hypothetical protein	1.17	2.13E-01	1.86	4.27E-01	1.00	9.73E-01
MSMEG_3283	membrane transport protein	0.99	9.12E-01	0.89	3.00E-01	1.48	7.77E-02
MSMEG_3284	transcriptional regulator, MarR family protein	1.03	4.92E-01	0.89	3.18E-01	0.93	3.29E-01
MSMEG_3285	3-isopropylmalate dehydrogenase	0.85	2.39E-01	0.89	3.30E-02	1.60	1.02E-01
MSMEG_3286	methyltransferase	0.93	3.80E-01	0.97	6.17E-01	1.79	8.10E-03
MSMEG_3287	hydrolase, alpha/beta fold family protein	1.07	2.95E-01	1.09	5.33E-01	1.08	1.49E-01
MSMEG_3288	LysM domain protein	12.40	2.76E-02	0.69	4.23E-01	1.13	7.41E-01
MSMEG_3289	gp61 protein	1.17	2.54E-01	1.67	7.01E-02	0.60	1.23E-01
MSMEG_3290	regulatory protein	1.50	5.26E-02	1.37	9.50E-02	1.56	2.78E-01
MSMEG_3291	hypothetical protein	1.05	6.29E-01	1.20	2.32E-01	1.01	8.55E-01
MSMEG_3292	DNA-binding protein	0.87	4.95E-01	1.23	2.28E-01	0.88	3.73E-01
MSMEG_3293	hypothetical protein	1.29	1.29E-02	0.87	2.53E-01	0.91	3.10E-01
MSMEG_3294	hypothetical protein	1.05	6.26E-01	1.28	3.20E-01	12.45	3.78E-01
MSMEG_3295	hypothetical protein	0.89	4.11E-01	1.10	7.56E-02	1.36	1.74E-01
MSMEG_3296	ECF-family protein sigma factor H	1.01	8.98E-01	0.99	8.41E-01	1.04	2.29E-01
MSMEG_3297	transcriptional regulator, CadC	0.73	2.46E-01	0.97	7.99E-01	0.38	7.86E-03
MSMEG_3298	response regulator receiver domain protein	0.37	1.98E-02	1.31	2.14E-01	0.69	7.93E-02
MSMEG_3299	putative oxidoreductase; this gene contains a frame shift which is not the result of sequencing error	0.35	5.08E-05	1.65	5.79E-02	0.77	2.66E-01
MSMEG_3300	oxidoreductase, Gfo/Idh/MocA family protein	0.53	1.39E-02	1.19	9.10E-02	0.80	1.39E-01
MSMEG_3301	conserved hypothetical protein	0.55	2.15E-02	0.96	7.63E-01	0.84	1.47E-01
MSMEG_3302	short-chain dehydrogenase/reductase SDR	0.64	2.48E-02	1.05	8.19E-01	0.85	3.15E-01
MSMEG_3303	carboxylic ester hydrolase	0.82	1.59E-01	1.02	8.41E-01	1.10	5.33E-01
MSMEG_3304	succinate semialdehyde dehydrogenase	1.15	6.17E-01	2.54	5.84E-02	2.21	3.59E-01
MSMEG_3305	integral membrane protein DUF6	1.10	2.81E-01	1.41	3.13E-01	1.07	2.66E-01
MSMEG_3306	zinc-binding alcohol dehydrogenase	0.81	1.63E-01	1.21	3.64E-01	1.28	3.73E-01
MSMEG_3307	methyltransferase FkbM; this gene contains a frame shift which is not the result of sequencing error	1.83	6.76E-02	0.85	1.81E-01	1.53	1.41E-02
MSMEG_3307	methyltransferase FkbM; this gene contains a frame shift which is not the result of sequencing error	1.30	2.50E-01	1.09	1.92E-01	0.98	7.51E-01
MSMEG_3308	universal stress protein family protein, putative	0.66	8.31E-03	1.26	3.24E-02	1.13	8.09E-01
MSMEG_3309	conserved hypothetical protein	0.60	1.63E-04	1.10	2.02E-01	0.80	3.80E-01
MSMEG_3310	integral membrane protein	1.42	2.87E-01	1.18	1.02E-01	1.12	1.28E-01

MSMEG_3311	acyl carrier protein		1.71	1.80E-01	2.30	2.15E-02	0.52	6.15E-02
MSMEG_3312	hemerythrin HHE cation binding domain subfamily protein, putative		1.17	3.50E-01	1.26	7.81E-02	1.19	1.74E-01
MSMEG_3313	xylose repressor, putative		1.07	3.74E-01	1.02	6.18E-01	1.49	5.38E-02
MSMEG_3314	transport protein		1.06	5.90E-01	1.17	3.88E-01	1.02	6.21E-01
MSMEG_3315	hypothetical protein		3.24	2.97E-03	0.80	1.02E-01	1.14	5.16E-02
MSMEG_3316	transporter, major facilitator family protein		0.88	5.92E-01	1.13	1.37E-01	0.93	2.69E-01
MSMEG_3317	dihydrodipicolinate reductase, N-terminus domain protein	*dapB*	2.74	4.06E-02	0.86	1.95E-01	0.87	4.50E-01
MSMEG_3318	oxidoreductase		0.85	1.15E-01	1.03	6.63E-01	1.12	1.62E-01
MSMEG_3319	repressor protein		1.14	5.21E-01	0.87	1.71E-01	1.12	1.46E-01
MSMEG_3320	LysR-family transcriptional regulator; this gene contains a frame shift which is not the result of sequencing error; identified by match to protein family HMM PF00126		0.57	6.31E-03	1.09	5.10E-01	2.28	5.87E-02
MSMEG_3321	ATP/GTP-binding protein		2.78	5.77E-03	0.84	2.47E-01	1.47	2.99E-01
MSMEG_3322	hypothetical protein		1.47	3.24E-01	0.94	5.04E-01	1.35	3.30E-01
MSMEG_3323	conserved hypothetical protein		0.96	5.62E-01	0.99	7.57E-01	1.78	4.94E-02
MSMEG_3324	hypothetical protein		1.67	6.13E-02	1.22	4.45E-02	1.04	5.80E-01
MSMEG_3325	conserved hypothetical protein		4.36	1.50E-02	0.76	6.74E-02	1.09	2.02E-01
MSMEG_3326	carboxylate-amine ligase		4.35	5.72E-03	0.82	3.68E-01	3.07	1.49E-03
MSMEG_3327	hypothetical protein		5.70	5.36E-03	0.74	5.95E-02	1.81	4.72E-02
MSMEG_3328	hypothetical protein		3.35	3.86E-03	0.69	3.12E-02	1.56	4.24E-02
MSMEG_3329	hypothetical protein		3.83	1.05E-02	0.73	1.28E-01	1.22	2.43E-01
MSMEG_3330	conserved domain protein		17.48	8.27E-03	0.83	3.88E-01	1.47	1.10E-01
MSMEG_3331	hypothetical protein		22.44	1.01E-02	0.69	5.59E-02	1.48	2.01E-01
MSMEG_3332	transcriptional regulator, TetR family protein		1.37	9.55E-02	0.83	8.43E-02	0.98	9.38E-01
MSMEG_3333	putative fatty acid desaturase, authentic frameshift; this gene contains a frame shift which is not the result of sequencing error		1.18	5.87E-03	1.08	5.20E-01	2.34	5.81E-02
MSMEG_3334	hypothetical protein		1.55	9.53E-02	0.99	8.73E-01	1.11	4.79E-01
MSMEG_3335	transcriptional regulator, IclR family protein, putative		1.56	5.57E-02	0.83	2.03E-02	0.97	9.04E-01
MSMEG_3336	hydrolase		0.67	6.06E-03	0.91	6.03E-02	1.00	9.86E-01
MSMEG_3337	hypothetical protein		0.92	3.11E-01	1.02	8.20E-01	0.92	3.09E-01
MSMEG_3338	oxidoreductase, FAD/FMN-binding		1.32	4.06E-02	1.04	7.84E-01	1.07	8.03E-01
MSMEG_3339	hypothetical protein		1.16	4.63E-01	0.97	7.94E-01	1.05	6.26E-01
MSMEG_3340	hypothetical protein		1.87	1.02E-01	0.90	7.11E-02	1.00	6.56E-01

MSMEG_3341	Transposase IS116/IS110/IS902 family protein	0.84	1.27E-01	1.55	1.61E-01	0.96	7.85E-01
MSMEG_3342	hypothetical protein	1.58	7.61E-02	1.04	8.17E-01	0.59	1.93E-02
MSMEG_3343	hypothetical protein	1.45	8.03E-02	0.83	2.87E-01	1.04	8.02E-01
MSMEG_3344	hypothetical protein	2.21	2.77E-02	0.92	6.36E-01	0.87	1.63E-01
MSMEG_3345	transcriptional regulator, GntR family protein	1.01	9.34E-01	0.96	5.99E-01	1.03	7.95E-01
MSMEG_3346	putative hydroxyacid aldolase	0.97	7.14E-01	1.03	4.17E-01	0.99	9.30E-01
MSMEG_3347	nitrilase/cyanide hydratase and apolipoprotein N-acyltransferase	0.65	1.26E-01	1.07	3.13E-01	0.88	1.23E-01
MSMEG_3348	bacterial extracellular solute-binding protein, family protein 7	0.56	3.11E-02	1.18	2.35E-01	4.17	3.72E-01
MSMEG_3349	trap dicarboxylate transporter, dctq subunit, putative	0.66	8.54E-03	1.02	9.08E-01	1.07	6.91E-01
MSMEG_3350	TRAP transporter, DctM-like membrane protein	0.50	7.27E-04	1.11	5.08E-01	1.04	2.37E-01
MSMEG_3351	hypothetical protein	0.81	3.55E-01	1.82	1.52E-01	1.01	7.86E-01
MSMEG_3352	hypothetical protein	1.86	3.91E-03	0.69	3.21E-03	1.30	2.98E-01
MSMEG_3353	hypothetical protein	1.53	2.35E-03	0.83	1.18E-01	0.93	4.22E-01
MSMEG_3354	TetR family protein transcriptional regulatory protein	1.24	1.33E-01	1.24	7.70E-02	1.33	2.28E-01
MSMEG_3355	conserved hypothetical protein	0.92	5.99E-01	1.13	3.76E-01	0.93	2.41E-01
MSMEG_3356	conserved hypothetical protein TIGR00026	1.69	8.21E-02	1.01	9.62E-01	0.84	1.78E-01
MSMEG_3357	metal-dependent phosphohydrolase, HD subdomain	0.44	2.36E-03	1.17	7.32E-02	0.84	4.06E-01
MSMEG_3358	YaeQ protein	0.48	5.29E-05	1.04	2.51E-01	1.13	4.70E-01
MSMEG_3359	cis-3-chloroacrylic acid dehalogenase, putative	0.60	1.79E-02	0.92	2.01E-01	1.46	2.35E-01
MSMEG_3360	transcriptional regulator, putative	0.83	6.93E-02	1.04	5.73E-01	0.98	9.27E-01
MSMEG_3361	conserved hypothetical protein	1.33	2.20E-01	1.16	4.37E-01	0.74	1.74E-01
MSMEG_3362	enoyl-CoA hydratase	1.53	4.13E-02	1.46	1.34E-01	0.98	7.74E-01
MSMEG_3363	regulatory protein, TetR	0.46	6.27E-04	1.00	9.99E-01	1.18	4.72E-01
MSMEG_3364	RhtB family protein transporter	0.28	1.26E-05	0.91	1.70E-01	1.22	5.44E-02
MSMEG_3365	transcriptional regulator, AraC family protein	0.56	4.50E-02	1.55	2.69E-01	1.11	4.16E-01
MSMEG_3366	isonitrile hydratase, putative	1.02	8.32E-01	0.89	1.91E-01	1.11	4.41E-01
MSMEG_3367	short-chain dehydrogenase/reductase SDR	0.95	1.94E-01	1.05	3.90E-01	1.15	9.71E-02
MSMEG_3368	hypothetical protein	1.08	5.52E-01	1.60	1.74E-02	1.42	1.81E-01
MSMEG_3369	putative transport protein	1.09	7.16E-01	1.48	2.54E-01	1.04	7.48E-01
MSMEG_3370	hypothetical protein	1.09	2.21E-01	1.05	5.46E-01	1.04	5.13E-01

MSMEG_3371	short-chain dehydrogenase/reductase SDR; this region contains one or more premature stops and/or frameshifts which are not the result of sequencing error; identified by match to protein family HMM PF00106	0.86	6.73E-02	1.06	4.01E-01	0.83	5.66E-02
MSMEG_3372	transcriptional regulator, ArsR family protein	2.46	2.44E-04	0.96	5.99E-01	0.94	5.62E-01
MSMEG_3373	major facilitator superfamily protein MFS_1	0.87	3.56E-01	1.26	5.14E-01	1.15	3.06E-01
MSMEG_3374	conserved hypothetical protein	2.18	6.42E-02	1.19	3.68E-01	0.74	2.07E-01
MSMEG_3375	alcohol dehydrogenase	0.54	1.80E-03	1.19	2.13E-01	1.18	4.20E-01
MSMEG_3376	beta-lactamase	1.54	2.51E-03	0.96	6.24E-01	1.08	2.69E-01
MSMEG_3377	hypothetical protein	0.86	7.21E-02	1.34	5.13E-01	1.36	2.86E-01
MSMEG_3378	beta-lactamase	0.83	1.06E-01	2.34	9.41E-02	1.03	7.52E-02
MSMEG_3379	2-deoxy-D-gluconate 3-dehydrogenase	0.64	4.62E-02	1.20	2.86E-01	1.01	9.17E-01
MSMEG_3380	pyridoxamine 5'-phosphate oxidase-related, FMN-binding	1.44	2.84E-02	1.18	3.50E-01	0.73	5.06E-02
MSMEG_3381	acyl carrier protein phosphodiesterase	0.52	9.51E-05	1.68	6.62E-02	0.86	4.04E-01
MSMEG_3382	transcriptional regulator, ArsR family protein	0.84	2.08E-01	1.00	9.86E-01	1.13	4.73E-01
MSMEG_3383	hypothetical protein	1.49	6.57E-04	1.56	1.66E-01	1.24	4.61E-01
MSMEG_3384	isoflavone reductase	1.50	1.52E-02	2.35	1.66E-01	0.92	1.87E-01
MSMEG_3385	hypothetical protein	1.14	6.69E-01	2.02	2.46E-01	0.84	1.79E-01
MSMEG_3386	shikimate transporter	1.72	5.93E-02	4.16	1.32E-01	1.11	3.51E-01
MSMEG_3387	aldehyde dehydrogenase (NAD) family protein	1.59	1.39E-01	5.46	1.84E-01	0.95	5.00E-01
MSMEG_3388	S-(hydroxymethyl)glutathione dehydrogenase/class III alcohol dehydrogenase	1.16	5.16E-01	4.54	1.75E-01	0.81	1.21E-01
MSMEG_3389	putative acetolactate synthase	2.16	2.51E-02	1.00	9.89E-01	0.95	2.78E-01
MSMEG_3390	enoyl-CoA hydratase	1.42	7.50E-02	0.89	1.81E-01	0.79	4.00E-02
MSMEG_3391	L-carnitine dehydratase/bile acid-inducible protein F	1.50	4.79E-02	1.42	4.06E-02	0.87	1.47E-01
MSMEG_3392	acyl-CoA dehydrogenase domain protein	1.50	4.40E-04	1.03	7.38E-01	1.07	4.73E-01
MSMEG_3393	putative acyl-CoA dehydrogenase	1.82	4.59E-03	0.91	1.86E-01	0.37	3.25E-03
MSMEG_3394	cupin domain protein	2.20	2.77E-04	1.10	6.12E-02	0.32	1.31E-04
MSMEG_3395	short chain dehydrogenase	2.26	1.10E-03	0.90	1.43E-01	0.38	1.34E-05
MSMEG_3396	transcriptional regulator, IclR family protein	0.84	2.13E-02	1.15	2.07E-01	1.38	1.85E-01
MSMEG_3397	acetylornithine deacetylase	0.98	9.06E-01	1.30	1.65E-01	0.88	2.20E-01
MSMEG_3398	integral membrane transport protein	0.94	7.18E-01	1.39	9.20E-02	1.06	2.66E-01
MSMEG_3399	conserved hypothetical protein; this gene contains a frame shift which is not the result of sequencing error	0.41	1.81E-03	1.28	2.53E-02	0.60	2.04E-02

MSMEG_3399	conserved hypothetical protein; this gene contains a frame shift which is not the result of sequencing error	0.65	7.41E-03	0.99	9.62E-01	2.26	4.47E-01
MSMEG_3400	glutamyl-tRNA(Gln) amidotransferase subunit A	0.43	8.84E-03	1.04	7.16E-01	1.07	6.80E-01
MSMEG_3401	LamB/YcsF family protein	0.26	2.78E-04	0.96	8.19E-01	0.89	4.58E-01
MSMEG_3402	cytosine permease, putative	0.23	4.89E-05	1.15	5.96E-01	0.90	3.12E-01
MSMEG_3403	formamidase	0.43	6.38E-03	0.95	7.71E-01	1.01	7.36E-01
MSMEG_3404	HNH endonuclease domain protein	0.51	1.41E-03	0.91	1.40E-01	0.96	2.40E-01
MSMEG_3405	methylenomycin A resistance protein	0.69	9.57E-03	5.30	1.56E-01	1.15	4.32E-01
MSMEG_3406	putative transcriptional regulator, TetR family protein	1.15	6.40E-01	1.08	6.55E-01	1.15	4.89E-01
MSMEG_3407	epoxide hydrolase	1.35	9.45E-02	2.45	1.09E-01	0.98	7.61E-01
MSMEG_3408	conserved hypothetical protein	1.43	2.95E-02	0.92	4.86E-01	1.74	4.51E-03
MSMEG_3409	oxidoreductase	1.03	7.17E-01	4.47	1.05E-01	1.19	3.79E-01
MSMEG_3410	transcriptional regulator, TetR family protein	1.43	1.41E-01	7.71	1.21E-01	0.96	4.56E-01
MSMEG_3411	MOSC domain protein	1.09	5.41E-02	0.76	5.13E-02	1.08	7.52E-02
MSMEG_3412	polysaccharide deacetylase family protein	0.96	3.96E-01	1.60	1.10E-01	1.07	4.67E-01
MSMEG_3413	MerR-family transcriptional regulator; this gene contains a frame shift which is not the result of sequencing error; identified by match to protein family HMM PF00376	2.89	1.00E-03	0.75	1.21E-01	0.84	1.20E-03
MSMEG_3413	MerR-family transcriptional regulator; this gene contains a frame shift which is not the result of sequencing error; identified by match to protein family HMM PF00376	1.81	1.19E-02	0.81	2.36E-01	1.17	1.83E-01
MSMEG_3414	conserved hypothetical protein	0.92	7.90E-01	2.50	2.20E-01	1.28	4.13E-01
MSMEG_3415	MarR-family protein regulatory protein	0.84	1.09E-01	1.19	8.10E-02	0.90	3.11E-01
MSMEG_3416	protein of unknown function DUF6, transmembrane, putative	1.19	3.12E-01	1.29	3.47E-01	1.32	2.64E-01
MSMEG_3417	conserved hypothetical protein	1.09	6.21E-01	1.33	2.15E-02	0.79	1.73E-01
MSMEG_3418	conserved hypothetical protein	1.44	2.84E-01	2.01	1.20E-01	0.89	6.03E-01
MSMEG_3419	hypothetical protein	2.47	2.06E-01	1.75	4.70E-02	1.02	9.26E-01
MSMEG_3420	gluconate 5-dehydrogenase	1.00	9.77E-01	1.47	9.88E-02	0.85	2.33E-01
MSMEG_3421	inner membrane metabolite transport protein YdfJ	1.19	1.04E-01	1.33	2.34E-01	0.79	1.60E-01
MSMEG_3422	D-isomer specific 2-hydroxyacid dehydrogenase, NAD-binding	1.09	4.16E-01	0.94	2.90E-01	0.83	9.89E-02
MSMEG_3423	probable IclR family protein regulator	1.90	4.33E-01	0.86	3.45E-02	1.16	4.93E-01
MSMEG_3424	transcriptional regulator, MerR family protein	0.82	3.22E-01	1.11	3.74E-01	8.03	3.09E-01

MSMEG_3425	TetR-family protein transcriptional regulator		1.62	3.01E-02	1.45	2.81E-01	1.18	1.97E-01
MSMEG_3426	short chain dehydrogenase		1.82	1.02E-01	1.25	3.71E-01	0.83	3.94E-01
MSMEG_3427	metal dependent hydrolase		1.40	1.50E-01	1.00	9.70E-01	1.20	3.96E-01
MSMEG_3428	conserved hypothetical protein		1.32	5.16E-02	1.11	4.05E-01	1.49	2.20E-01
MSMEG_3429	O-acyl transferase		2.04	9.92E-04	1.05	6.24E-01	0.27	3.87E-06
MSMEG_3430	SAM-dependent methyltransferase		0.50	9.74E-04	0.85	2.31E-01	1.07	8.13E-01
MSMEG_3431	choline dehydrogenase		1.09	3.31E-01	2.80	8.13E-02	1.15	4.97E-01
MSMEG_3432	choline dehydrogenase		1.16	3.60E-01	7.38	1.19E-01	1.03	5.03E-01
MSMEG_3434	peptidyl-prolyl cis-trans isomerase, fkbp-type domain protein		0.35	8.20E-06	1.02	8.34E-01	1.72	1.99E-01
MSMEG_3435	conserved hypothetical protein		0.91	5.13E-02	0.80	5.90E-02	0.68	1.95E-02
MSMEG_3436	conserved hypothetical protein		0.76	1.72E-03	0.80	2.36E-03	0.98	8.09E-01
MSMEG_3437	putative esterase family protein		1.63	5.27E-02	2.11	1.08E-01	0.62	1.67E-02
MSMEG_3438	hypothetical protein		1.19	2.35E-01	3.84	1.16E-01	0.92	4.77E-01
MSMEG_3439	hypothetical protein		1.91	3.69E-01	2.63	1.02E-01	0.62	2.71E-02
MSMEG_3440	L-serine ammonia-lyase	*sdaA*	1.41	1.43E-02	0.91	3.48E-01	0.89	3.38E-01
MSMEG_3441	hypothetical protein		1.48	4.94E-02	1.27	1.65E-01	1.06	8.73E-01
MSMEG_3442	cyclohexadienyl dehydratase		1.33	5.46E-01	1.17	2.91E-01	1.16	4.50E-01
MSMEG_3443	hypothetical protein		1.33	1.08E-02	1.72	3.11E-02	0.76	1.09E-01
MSMEG_3444	choline dehydrogenase		1.09	2.23E-01	12.62	1.36E-01	1.01	9.34E-01
MSMEG_3445	5,10-methylenetetrahydromethanopterin		2.00	3.63E-01	1.37	2.76E-01	0.87	4.39E-01
MSMEG_3446	hypothetical protein		1.76	2.41E-01	1.29	5.13E-01	1.58	4.08E-01
MSMEG_3447	two-component system response regulator		1.76	5.53E-02	6.74	9.69E-02	1.29	4.10E-01
MSMEG_3448	two-component system sensor kinase		1.19	1.30E-01	1.31	1.86E-01	0.99	9.21E-01
MSMEG_3449	DNA-binding protein		1.93	1.90E-02	1.02	8.33E-01	0.84	9.86E-02
MSMEG_3450	haloacid dehalogenase, type II	*dehII*	2.40	1.18E-02	1.50	1.12E-01	2.95	4.10E-01
MSMEG_3451	short-chain dehydrogenase/reductase		1.10	5.00E-01	0.82	9.27E-02	0.83	5.79E-01
MSMEG_3452	TetR family protein transcriptional regulator		1.33	1.48E-01	2.32	9.24E-02	0.92	4.52E-02
MSMEG_3453	hypothetical protein		1.19	4.07E-01	0.63	1.68E-02	1.21	3.18E-01
MSMEG_3454	hypothetical protein		1.73	2.15E-03	0.66	8.04E-03	0.72	2.69E-03
MSMEG_3455	heat shock protein 15	*hslR*	1.59	8.45E-03	0.70	1.51E-03	0.90	5.47E-02
MSMEG_3456	hydroxylaminobenzene mutase		1.37	2.08E-01	1.03	7.97E-01	1.03	8.66E-01
MSMEG_3457	DoxD family protein/pyridine nucleotide-disulfide oxidoreductase		1.02	7.94E-01	0.96	6.18E-01	0.87	2.41E-01
MSMEG_3458	conserved hypothetical protein		1.01	9.57E-01	0.96	5.22E-01	0.95	2.84E-01
MSMEG_3459	conserved hypothetical protein		1.01	8.89E-01	1.07	1.83E-01	2.27	1.17E-01
MSMEG_3460	ferric uptake regulation protein		8.12	1.01E-03	1.62	2.83E-02	0.47	7.69E-04
MSMEG_3461	catalase/peroxidase HPI	*katG*	5.89	2.70E-03	1.14	3.80E-01	0.69	1.56E-01

MSMEG_3462	oxidoreductase		1.77	2.75E-02	1.16	3.06E-01	1.35	4.17E-01
MSMEG_3463	putative esterase		1.65	3.02E-02	1.05	7.12E-01	0.79	1.93E-01
MSMEG_3464	alcohol dehydrogenase		2.22	3.43E-02	0.92	6.38E-01	0.47	4.57E-03
MSMEG_3465	acyl-CoA synthase		0.60	7.23E-04	0.77	6.83E-02	5.13	1.20E-02
MSMEG_3466	hypothetical protein		1.09	4.01E-01	1.44	2.73E-01	0.97	1.25E-01
MSMEG_3467	conserved hypothetical protein		0.89	3.76E-02	0.90	2.51E-01	1.21	4.87E-02
MSMEG_3468	AMP-binding enzyme		1.27	2.80E-01	1.00	9.25E-01	1.10	4.55E-01
MSMEG_3469	TetR family protein transcriptional regulator		0.64	2.33E-03	0.99	9.03E-01	1.25	4.18E-01
MSMEG_3470	NAD-dependent epimerase/dehydratase		1.41	1.92E-01	36.64	1.26E-01	1.02	8.03E-01
MSMEG_3471	GTP cyclohydrolase		2.07	1.09E-03	8.96	1.19E-01	1.06	8.66E-01
MSMEG_3472	conserved hypothetical protein		1.50	1.61E-02	9.02	1.23E-01	1.01	7.12E-01
MSMEG_3473	uracil phosphoribosyltransferase	*upp*	1.58	2.21E-03	7.25	9.48E-02	1.11	4.88E-01
MSMEG_3474	probable conserved integral membrane protein		1.35	1.66E-01	9.94	7.81E-02	1.03	5.88E-01
MSMEG_3475	conserved hypothetical protein		0.94	5.31E-01	0.94	4.99E-01	1.07	6.29E-01
MSMEG_3476	peptidase, M48 family protein		1.15	2.26E-01	0.93	6.55E-01	1.26	1.58E-01
MSMEG_3477	possible inv protein		1.96	2.60E-03	0.69	6.28E-02	2.27	1.68E-01
MSMEG_3478	tena/thi-4 family protein		0.83	1.28E-01	2.14	1.19E-01	0.85	1.28E-01
MSMEG_3479	thiol peroxidase		1.20	4.40E-02	0.93	4.95E-01	0.71	1.21E-01
MSMEG_3480	streptomycin 6-kinase (Streptidine kinase) (Streptomycin6-phosphotransferase) (APH(6))		0.76	5.56E-02	3.26	1.15E-01	2.38	1.61E-01
MSMEG_3481	O-succinylbenzoate synthase		1.18	4.13E-01	1.00	9.88E-01	1.13	9.64E-02
MSMEG_3482	hypothetical protein		1.64	8.54E-04	1.10	4.15E-01	0.89	5.50E-01
MSMEG_3483	mosc domain protein		0.58	5.54E-04	1.59	1.25E-01	0.83	2.54E-01
MSMEG_3484	cupin domain protein		0.93	3.86E-01	1.38	2.94E-01	1.18	2.53E-01
MSMEG_3485	putative ECF sigma factor RpoE1		1.49	1.94E-01	1.02	9.06E-01	0.90	3.70E-01
MSMEG_3486	catalase KatA	*katA*	1.16	5.33E-01	0.94	8.09E-02	1.09	3.61E-01
MSMEG_3487	metal-dependent phosphohydrolase		1.82	1.89E-01	0.92	2.79E-01	1.28	3.64E-01
MSMEG_3488	transcriptional regulator, AraC family protein		0.93	1.36E-01	1.71	1.27E-01	1.15	9.39E-02
MSMEG_3489	conserved hypothetical protein, putative		1.74	1.61E-02	0.72	1.71E-01	1.08	7.13E-01
MSMEG_3490	putative membrane acyltransferase		0.70	7.16E-04	0.83	1.18E-01	4.42	4.61E-02
MSMEG_3491	conserved hypothetical protein		1.45	1.00E-02	0.79	1.98E-02	0.70	9.31E-03
MSMEG_3492	phosphodiesterase		0.83	1.21E-01	2.59	1.04E-01	1.96	9.27E-02
MSMEG_3493	putative secreted protein		1.05	4.46E-01	0.84	1.65E-01	0.62	1.63E-02
MSMEG_3494	putative secreted protein		1.79	1.85E-03	0.79	5.13E-02	0.38	2.70E-07
MSMEG_3495	MmpS5 protein		0.90	1.07E-01	0.83	7.76E-03	1.37	3.30E-01
MSMEG_3496	MmpL4 protein		0.66	6.32E-04	0.92	5.83E-01	2.17	1.11E-03
MSMEG_3497	transcriptional regulator, ArsR family protein		1.20	3.46E-02	1.21	1.97E-01	0.86	4.70E-01
MSMEG_3498	hypothetical protein		6.14	1.51E-02	0.87	2.81E-01	0.99	7.82E-01

MSMEG_3499	conserved hypothetical protein	0.49	3.98E-03	0.70	2.98E-03	0.75	1.50E-03
MSMEG_3500	conserved hypothetical protein	0.43	6.54E-05	0.94	5.23E-01	1.91	1.53E-01
MSMEG_3501	hypothetical protein	1.70	5.08E-05	0.89	4.05E-01	0.72	2.90E-04
MSMEG_3502	hypothetical protein	1.81	4.63E-02	1.09	3.93E-01	2.58	1.97E-01
MSMEG_3503	conserved hypothetical protein	0.61	7.50E-04	0.91	5.74E-01	1.06	8.59E-01
MSMEG_3504	probable membrane protein	0.19	2.41E-07	1.64	9.04E-03	0.39	1.18E-03
MSMEG_3505	6-aminohexanoate-cyclic-dimer hydrolase	0.49	1.89E-04	1.13	6.20E-02	0.95	5.42E-01
MSMEG_3506	putative amino acid decarboxylase, Pyridoxal-dependent protein	0.68	4.16E-02	1.10	5.87E-02	1.39	1.11E-02
MSMEG_3507	fructose-bisphosphate aldolase class-I	0.83	1.65E-02	0.78	2.36E-02	0.43	1.21E-04
MSMEG_3508	hydrolase, alpha/beta hydrolase fold family protein	0.86	4.36E-02	0.91	5.92E-01	0.96	8.54E-01
MSMEG_3509	conserved hypothetical protein	1.16	2.72E-01	0.99	8.85E-01	0.96	6.49E-01
MSMEG_3511	hydroxylase	5.99	2.78E-02	0.42	3.22E-02	0.86	5.47E-01
MSMEG_3512	competence damage-inducible protein A	0.55	3.85E-03	0.88	1.20E-01	1.72	3.54E-01
MSMEG_3513	enhanced intracellular survival protein	0.74	2.30E-02	0.92	4.54E-01	1.82	1.17E-01
MSMEG_3514	hypothetical protein	1.16	9.02E-02	1.14	3.43E-01	1.16	8.42E-02
MSMEG_3515	3-alpha-(or 20-beta)-hydroxysteroid dehydrogenase	1.20	2.94E-01	1.09	4.24E-01	0.77	1.35E-01
MSMEG_3516	conserved hypothetical protein; this gene contains a frame shift which is not the result of sequencing error; identified by match to protein family HMM PF01113	1.48	3.26E-02	1.38	7.53E-02	0.82	1.44E-01
MSMEG_3517	HAD-superfamily protein subfamily protein IB hydrolase	1.05	2.51E-01	0.85	1.34E-02	1.06	4.28E-01
MSMEG_3518	conserved hypothetical protein	1.92	7.32E-02	1.49	7.72E-02	1.20	4.60E-01
MSMEG_3519	oxidoreductase, 2-nitropropane dioxygenase family protein	1.03	5.90E-01	1.10	4.41E-01	0.86	5.20E-01
MSMEG_3520	TetR-family protein transcriptional regulator	2.14	5.91E-04	0.82	1.06E-01	0.69	2.06E-02
MSMEG_3521	molybdopterin oxidoreductase	1.63	4.66E-03	0.86	1.41E-02	0.80	1.48E-01
MSMEG_3522	dopamine receptor D4	1.91	9.37E-03	1.15	1.06E-01	0.49	5.05E-04
MSMEG_3523	probable membrane protein	1.60	4.36E-02	1.09	3.98E-01	1.16	5.00E-01
MSMEG_3524	linalool 8-monooxygenase	3.56	1.58E-02	1.25	4.51E-01	0.84	9.25E-02
MSMEG_3525	transcriptional regulator, XRE family protein	1.98	1.50E-03	0.83	1.06E-01	1.11	5.05E-01
MSMEG_3526	conserved hypothetical protein	0.97	8.68E-01	11.30	2.56E-01	1.08	5.98E-01
MSMEG_3527	putative HTH-type transcriptional regulator	1.21	2.44E-02	1.56	1.69E-01	0.85	1.20E-01
MSMEG_3528	ErfK/YbiS/YcfS/YnhG family protein	1.21	1.12E-01	0.57	3.55E-03	1.34	2.54E-01
MSMEG_3529	fatty acid hydroxylase	0.74	5.59E-02	0.90	7.98E-02	1.19	6.71E-01

MSMEG_3530	conserved hypothetical protein		1.27	2.99E-01	1.19	4.64E-01	1.13	1.34E-01
MSMEG_3531	conserved hypothetical protein		0.37	3.64E-07	0.89	9.95E-02	4.89	1.72E-02
MSMEG_3532	serine/threonine dehydratase family protein		0.66	6.43E-03	1.15	1.38E-01	1.26	1.98E-01
MSMEG_3533	Di-/tripeptide transporter		1.10	1.47E-01	1.70	3.70E-01	2.02	3.65E-01
MSMEG_3534	4-hydroxybenzoyl-CoA thioesterase		0.73	8.48E-03	0.98	6.72E-01	1.04	8.61E-01
MSMEG_3535	agmatinase	*speB*	1.72	4.78E-02	1.13	2.12E-01	0.74	1.47E-01
MSMEG_3536	sugar transport protein		1.32	3.28E-01	1.78	5.19E-02	0.62	3.56E-02
MSMEG_3537	hypothetical protein		1.00	9.75E-01	1.13	5.07E-01	0.84	1.88E-01
MSMEG_3538	cyclopropane-fatty-acyl-phospholipid synthase 1		1.46	1.22E-02	0.78	1.65E-02	0.62	2.19E-03
MSMEG_3539	hypothetical protein		1.07	6.40E-02	0.61	4.03E-02	2.23	3.56E-02
MSMEG_3540	conserved hypothetical protein		5.68	5.34E-04	1.07	6.93E-01	1.07	6.56E-01
MSMEG_3541	cytochrome C biogenesis protein transmembrane region		1.11	5.95E-01	1.87	6.68E-02	0.98	7.12E-01
MSMEG_3542	conserved hypothetical protein		4.91	3.12E-03	0.94	7.39E-01	1.25	7.30E-02
MSMEG_3543	soluble secreted antigen MPT53		2.60	1.65E-01	1.60	7.14E-02	0.75	3.12E-01
MSMEG_3544	conserved hypothetical protein		2.06	5.02E-03	0.48	1.52E-03	2.46	1.06E-02
MSMEG_3545	hypothetical protein		1.71	4.11E-03	0.45	1.15E-03	3.96	8.48E-03
MSMEG_3546	conserved hypothetical protein		0.90	3.12E-01	1.01	8.33E-01	1.47	2.31E-01
MSMEG_3547	ectoine/hydroxyectoine ABC transporter solute-binding protein	*ehuB*	1.33	1.26E-01	1.04	7.58E-01	1.04	3.83E-01
MSMEG_3548	ectoine/hydroxyectoine ABC transporter, permease protein EhuC	*ehuC*	1.41	1.66E-01	0.98	5.95E-01	0.94	2.39E-01
MSMEG_3549	ectoine/hydroxyectoine ABC transporter, permease protein EhuD	*ehuD*	1.50	1.57E-01	1.23	3.28E-01	1.10	2.71E-02
MSMEG_3550	ectoine/hydroxyectoine ABC transporter, ATP-binding protein	*ehuA*	1.52	3.20E-01	1.05	5.81E-01	1.01	1.64E-01
MSMEG_3551	linalool 8-monooxygenase		1.36	5.13E-02	0.96	6.12E-01	1.44	5.80E-02
MSMEG_3552	conserved hypothetical protein		0.81	2.26E-02	1.00	9.64E-01	1.03	8.45E-01
MSMEG_3553	dihydropyrimidinase	*hydA*	1.70	1.04E-01	0.90	1.06E-01	1.04	6.96E-01
MSMEG_3554	N5,N10-methylene- tetrahydromethanopterin reductase		2.87	1.32E-01	0.75	1.30E-01	1.18	6.01E-01
MSMEG_3555	hydrolase		3.32	2.10E-02	0.69	3.18E-02	0.95	3.38E-01
MSMEG_3556	integral membrane transporter		2.02	1.50E-01	0.69	3.29E-02	0.98	5.52E-01
MSMEG_3557	amino acid permease-associated region		1.09	5.07E-01	1.10	4.21E-01	1.27	2.16E-02
MSMEG_3558	carboxymethylenebutenolidase		3.86	2.47E-03	1.40	1.30E-01	0.60	6.16E-04
MSMEG_3559	short chain dehydrogenase		3.45	1.80E-02	1.17	2.32E-01	0.72	7.51E-02
MSMEG_3560	conserved hypothetical protein		1.89	1.29E-02	1.19	3.46E-01	0.89	3.56E-01
MSMEG_3561	glutamine synthetase, catalytic domain	*glnA*	2.54	1.01E-02	0.80	1.33E-01	0.88	1.84E-01

MSMEG_3562	4-carboxymuconolactone decarboxylase domain protein	*pcaC*	4.42	5.13E-04	0.97	7.22E-01	0.44	3.07E-04
MSMEG_3563	drug transporter		0.89	3.64E-01	1.12	4.82E-01	0.98	8.74E-01
MSMEG_3564	bacterioferritin	*bfr*	3.15	1.47E-03	1.19	3.53E-01	1.09	7.89E-01
MSMEG_3565	hypothetical protein		2.10	7.00E-02	0.90	8.53E-02	2.03	3.43E-01
MSMEG_3566	FadD16 protein		0.71	1.70E-03	1.03	7.67E-01	0.46	4.61E-04
MSMEG_3567	enoyl-CoA hydratase		0.66	7.06E-03	1.02	8.58E-01	1.72	8.80E-02
MSMEG_3568	conserved hypothetical protein		0.75	5.24E-02	0.96	7.06E-01	0.85	1.48E-01
MSMEG_3569	conserved hypothetical protein		0.39	1.79E-04	0.90	3.68E-01	1.52	1.69E-01
MSMEG_3570	conserved hypothetical protein		1.08	2.91E-01	0.98	8.74E-01	2.15	3.38E-02
MSMEG_3571	thioesterase family protein		1.57	2.16E-02	1.05	4.82E-01	0.96	5.95E-01
MSMEG_3572	transcriptional regulator, TetR family protein		0.72	1.39E-02	0.94	6.95E-01	3.42	2.51E-01
MSMEG_3573	integral membrane protein, putative		0.82	9.61E-02	1.15	5.34E-01	1.04	5.55E-01
MSMEG_3574	TPR domain protein		1.83	1.90E-03	0.80	4.61E-02	0.68	4.67E-03
MSMEG_3575	cytidine/deoxycytidylate deaminase, zinc-binding region		1.24	3.60E-02	1.04	4.47E-01	1.52	5.08E-02
MSMEG_3576	alpha-amylase 3		0.98	6.66E-01	1.01	8.88E-01	1.40	7.48E-03
MSMEG_3577	calpastatin		3.70	3.65E-03	1.27	5.64E-03	0.63	3.57E-02
MSMEG_3578	putative cyclase		1.16	2.03E-01	1.24	3.46E-01	0.73	3.00E-01
MSMEG_3579	probable conserved transmembrane protein		1.28	3.34E-04	0.80	5.14E-02	0.92	6.89E-01
MSMEG_3580	antigen 85-C		0.96	6.47E-01	0.60	2.45E-02	1.06	8.89E-01
MSMEG_3581	FabG protein		2.83	6.18E-02	1.43	2.12E-01	0.85	7.77E-02
MSMEG_3582	ATP-dependent protease La	*lon*	7.33	1.30E-03	3.83	1.23E-01	0.93	3.37E-01
MSMEG_3583	monooxygenase		3.88	3.38E-03	5.87	8.15E-02	1.07	7.47E-01
MSMEG_3584	membrane protein, MmpL family protein		1.15	4.18E-02	1.15	1.16E-01	0.76	2.88E-01
MSMEG_3585	MmpS2 protein		1.06	6.55E-01	1.42	1.20E-01	1.04	6.10E-01
MSMEG_3586	major facilitator family protein transporter		1.45	1.29E-01	1.14	6.15E-01	3.98	2.37E-01
MSMEG_3587	D-serine/D-alanine/glycine transporter		0.96	7.96E-01	3.22	1.92E-01	1.03	7.19E-01
MSMEG_3588	hypothetical protein		1.11	5.68E-01	2.35	1.19E-01	1.01	9.24E-01
MSMEG_3589	conserved hypothetical protein		1.45	1.22E-01	2.69	1.16E-01	0.92	2.46E-01
MSMEG_3590	conserved hypothetical protein		1.15	4.24E-01	2.20	2.38E-01	0.89	3.13E-01
MSMEG_3591	conserved hypothetical protein		1.15	3.74E-01	1.96	1.41E-01	0.92	5.44E-01
MSMEG_3592	hypothetical protein		0.54	2.57E-03	1.98	1.13E-01	1.66	3.86E-01
MSMEG_3593	conserved hypothetical protein		0.49	2.75E-03	2.80	2.83E-02	1.19	7.54E-01
MSMEG_3594	anthranilate dioxygenase reductase		2.83	2.84E-05	1.02	8.38E-01	1.37	6.18E-02
MSMEG_3595	conserved hypothetical protein		1.13	7.31E-02	1.00	9.73E-01	1.42	7.33E-02
MSMEG_3596	ATPase		0.69	3.90E-03	1.45	1.33E-01	1.61	6.17E-03
MSMEG_3596	ATPase		1.13	1.63E-01	1.11	3.72E-01	2.19	1.31E-02

MSMEG_3598	periplasmic sugar-binding proteins		1.38	2.84E-04	0.98	8.89E-01	2.61	4.61E-01
MSMEG_3599	sugar-binding transcriptional regulator, LacI family protein		1.74	9.61E-04	0.96	6.86E-01	0.48	3.96E-05
MSMEG_3600	hypothetical protein		0.81	5.04E-04	0.89	1.36E-02	0.89	1.84E-01
MSMEG_3601	ribose/xylose/arabinose/galactoside ABC-type transport systems, permease components		1.33	5.81E-02	0.94	6.52E-01	1.26	5.44E-01
MSMEG_3602	ribose transport ATP-binding protein RbsA		1.17	1.86E-01	1.03	7.19E-01	1.04	7.07E-01
MSMEG_3603	oxidoreductase, zinc-binding dehydrogenase family protein		1.25	2.00E-02	0.98	8.79E-01	1.35	3.53E-01
MSMEG_3604	sorbitol utilization protein SOU2		1.72	6.04E-03	0.89	3.41E-01	0.92	2.89E-01
MSMEG_3605	sorbitol dehydrogenase		1.78	4.91E-04	0.88	2.40E-01	0.63	5.99E-03
MSMEG_3606	transcriptional regulator		1.62	4.66E-02	1.08	2.14E-01	0.76	1.08E-01
MSMEG_3607	short chain dehydrogenase		1.10	5.62E-01	1.13	2.88E-01	0.92	5.00E-01
MSMEG_3608	acetyl-CoA acetyltransferase		0.51	1.81E-03	1.06	2.67E-01	2.11	1.37E-01
MSMEG_3609	hydride transferase 1		1.03	7.15E-01	0.84	1.33E-02	0.80	1.32E-01
MSMEG_3610	conserved hypothetical protein		0.96	7.15E-01	1.35	9.49E-02	1.47	1.80E-01
MSMEG_3611	D-xylulose 5-phosphate/D-fructose 6-phosphate phosphoketolase	*xfp*	1.23	1.94E-01	1.34	3.45E-02	0.56	1.06E-01
MSMEG_3612	probable conserved integral membrane protein		1.38	2.27E-02	0.63	1.31E-02	0.97	8.65E-01
MSMEG_3613	conserved hypothetical protein		0.85	9.95E-02	0.92	4.90E-01	0.98	8.46E-01
MSMEG_3614	hypothetical protein		0.99	7.61E-01	1.05	4.33E-01	0.89	5.79E-02
MSMEG_3615	zinc-binding alcohol dehydrogenase family protein		5.28	2.80E-04	0.55	5.28E-03	2.17	1.77E-01
MSMEG_3616	integral membrane protein		6.65	4.31E-05	0.66	2.58E-02	2.29	1.63E-01
MSMEG_3617	hypothetical protein		9.46	2.75E-03	0.97	8.71E-01	2.26	1.27E-01
MSMEG_3618	alanine and proline-rich secreted protein apa		1.11	3.31E-01	0.92	4.17E-01	0.72	1.26E-01
MSMEG_3619	short chain dehydrogenase		0.98	8.80E-01	1.10	4.26E-01	1.43	3.09E-03
MSMEG_3620	conserved hypothetical protein		1.02	6.78E-01	1.05	4.32E-01	0.89	2.21E-01
MSMEG_3621	NADH dehydrogenase	*ndh*	0.89	2.24E-01	2.02	1.24E-01	2.23	3.08E-03
MSMEG_3622	conserved hypothetical protein		0.47	1.65E-03	0.98	6.34E-01	1.37	1.62E-01
MSMEG_3623	urease accessory protein UreG	*ureG*	0.48	5.57E-04	0.96	4.14E-01	0.83	1.68E-01
MSMEG_3624	urease accessory protein uref		0.34	1.83E-04	1.00	9.78E-01	1.02	3.26E-01
MSMEG_3625	urease, alpha subunit	*ureC*	0.53	1.02E-04	0.92	3.28E-01	0.74	3.12E-02
MSMEG_3626	urease, beta subunit	*ureB*	0.40	6.33E-05	1.04	7.57E-01	1.26	3.20E-01
MSMEG_3627	urease, gamma subunit	*ureA*	0.43	2.75E-04	0.99	9.65E-01	0.99	9.60E-01
MSMEG_3628	ComA operon protein 2		0.52	6.24E-04	0.96	6.02E-01	2.82	1.32E-03
MSMEG_3629	conserved hypothetical protein		0.60	1.43E-02	1.34	1.25E-01	1.84	2.18E-01
MSMEG_3630	transcriptional repressor, CopY family protein		0.81	4.82E-03	0.93	2.16E-01	4.82	1.67E-03

MSMEG_3631	integral membrane protein		1.00	9.72E-01	1.03	7.49E-01	4.37	2.58E-03
MSMEG_3632	6-phosphogluconate dehydrogenase, decarboxylating	*gnd*	1.12	2.50E-02	0.99	9.36E-01	2.28	1.68E-03
MSMEG_3633	ferric cations import ATP-binding protein FbpC		0.52	5.12E-03	1.07	5.04E-02	1.83	8.22E-02
MSMEG_3634	IMP dehydrogenase family protein		0.95	4.38E-01	0.78	4.80E-02	3.31	1.37E-03
MSMEG_3635	iron(III)-transport system permease protein SfuB		0.89	2.00E-03	0.91	1.40E-01	1.74	4.59E-03
MSMEG_3636	ferric iron-binding periplasmic protein of ABC transporter		0.92	4.72E-01	0.92	2.94E-01	0.98	7.23E-01
MSMEG_3637	CBS domain protein		2.14	5.55E-02	0.68	1.61E-02	2.35	4.89E-04
MSMEG_3638	CBS domain protein		2.38	4.39E-03	0.90	2.27E-01	3.93	3.27E-04
MSMEG_3639	conserved hypothetical protein		0.88	1.38E-02	0.86	1.88E-01	2.04	1.75E-02
MSMEG_3640	malate synthase G	*glcB*	0.92	1.23E-01	1.01	8.82E-01	2.33	2.48E-03
MSMEG_3641	conserved hypothetical protein		0.68	5.54E-03	1.08	2.28E-02	3.59	1.55E-04
MSMEG_3642	glycine dehydrogenase	*gcvP*	0.46	7.87E-05	0.97	5.25E-01	5.65	1.28E-03
MSMEG_3643	hypothetical protein		1.06	8.70E-01	0.99	8.77E-01	1.68	3.82E-01
MSMEG_3644	transcriptional regulator, MerR family protein		2.14	1.83E-03	0.83	1.39E-01	1.18	4.95E-01
MSMEG_3645	conserved hypothetical protein		2.20	5.29E-04	1.10	3.36E-01	1.61	1.50E-02
MSMEG_3646	transcriptional regulator, MerR family protein		0.47	3.12E-05	0.97	4.13E-01	0.93	3.56E-01
MSMEG_3647	forkhead-associated protein		0.72	1.81E-04	0.90	3.30E-01	0.71	9.09E-03
MSMEG_3648	glycine cleavage system H protein	*gcvH*	0.76	1.34E-06	0.82	3.23E-02	0.60	2.51E-04
MSMEG_3649	conserved hypothetical protein		0.77	1.84E-03	0.94	1.82E-01	1.33	1.22E-02
MSMEG_3650	integral membrane protein		1.03	9.33E-01	1.19	2.18E-02	1.34	1.68E-02
MSMEG_3651	membrane associated protein		1.00	9.41E-01	1.07	4.08E-01	1.63	1.14E-02
MSMEG_3652	CDP-diacylglycerol-glycerol-3-phosphate		0.92	1.08E-02	1.23	1.93E-01	1.14	2.51E-01
MSMEG_3653	hypothetical protein		0.95	8.35E-01	1.14	3.25E-01	1.03	1.27E-02
MSMEG_3654	ATPase SecA2		0.80	2.89E-02	0.94	1.89E-01	1.21	1.23E-01
MSMEG_3655	ABC transporter, permease/ATP-binding protein		0.80	3.05E-02	1.17	1.76E-01	0.69	1.71E-03
MSMEG_3656	ABC transporter, permease/ATP-binding protein		0.64	1.96E-03	1.09	4.62E-01	1.14	5.00E-01
MSMEG_3657	succinate dehydrogenase		1.10	5.52E-02	0.84	2.17E-02	1.10	4.66E-01
MSMEG_3658	conserved hypothetical protein		1.88	2.67E-03	0.98	7.92E-01	3.60	2.56E-01
MSMEG_3659	TetR-family protein transcriptional regulator		0.99	9.65E-01	1.15	1.11E-01	0.97	4.17E-01
MSMEG_3660	conserved hypothetical protein TIGR00026		0.77	1.05E-02	1.20	3.05E-01	1.15	4.78E-01
MSMEG_3661	conserved hypothetical protein		0.92	5.31E-01	0.98	8.38E-01	0.50	5.96E-03
MSMEG_3662	mannose-binding lectin		0.33	8.08E-05	1.45	2.01E-01	0.64	3.54E-02
MSMEG_3663	oxidoreductase		0.44	1.47E-04	1.40	1.29E-01	1.11	4.11E-01
MSMEG_3664	transporter, monovalent cation:proton antiporter-2 (CPA2) family protein		1.72	8.29E-02	1.05	5.72E-01	0.93	9.05E-02
MSMEG_3665	TrkA domain protein		1.41	9.34E-02	0.83	1.71E-01	0.78	4.49E-02

MSMEG_3666	C-5 sterol desaturase		0.90	1.13E-01	1.20	1.09E-01	1.67	7.27E-02
MSMEG_3667	para-nitrobenzyl esterase		1.54	3.18E-02	0.88	1.31E-02	1.28	1.70E-01
MSMEG_3668	acyl-CoA dehydrogenase		0.99	3.69E-01	5.14	1.87E-01	1.12	5.96E-01
MSMEG_3669	conserved hypothetical protein		1.33	3.91E-01	3.61	1.46E-01	1.05	2.25E-01
MSMEG_3670	transporter, small multidrug resistance (SMR) family protein		0.78	8.40E-02	1.48	3.72E-01	0.91	1.47E-01
MSMEG_3671	integral membrane protein		0.91	1.92E-01	0.90	3.86E-01	3.18	3.06E-04
MSMEG_3672	transporter, small multidrug resistance (SMR) family protein		0.77	2.60E-02	0.98	7.74E-01	2.95	1.62E-01
MSMEG_3673	4-alpha-glucanotransferase	*malQ*	0.77	1.27E-01	1.30	4.52E-02	0.87	2.41E-01
MSMEG_3674	hypothetical protein		1.58	7.94E-03	0.85	2.83E-01	0.67	3.80E-03
MSMEG_3675	metal-dependent hydrolase		3.00	1.12E-03	0.73	2.39E-02	1.68	5.67E-02
MSMEG_3676	phenoxybenzoate dioxygenase beta subunit		1.60	2.02E-04	0.85	1.17E-01	1.75	9.65E-02
MSMEG_3677	serine/threonine protein kinase		1.31	3.09E-02	0.87	3.66E-01	0.51	5.12E-04
MSMEG_3678	hypothetical protein		1.07	3.99E-01	0.72	1.45E-02	3.42	5.17E-03
MSMEG_3679	phosphohydrolase		1.24	3.86E-01	1.04	7.31E-01	1.83	2.85E-01
MSMEG_3680	hypothetical protein		1.63	7.58E-02	1.23	2.28E-01	2.04	1.95E-01
MSMEG_3681	conserved hypothetical protein		0.85	9.63E-03	0.71	8.77E-03	1.33	1.07E-01
MSMEG_3682	conserved hypothetical protein		0.81	1.20E-01	1.28	2.27E-01	2.84	1.59E-01
MSMEG_3683	conserved hypothetical protein		0.93	3.49E-01	0.79	5.52E-02	1.00	9.88E-01
MSMEG_3684	nuclear transport factor 2 (NTF2) domain family protein		1.27	6.49E-02	0.88	8.13E-02	1.00	9.69E-01
MSMEG_3685	conserved hypothetical protein		5.37	1.19E-03	0.84	2.38E-01	1.07	4.22E-01
MSMEG_3686	conserved hypothetical protein		2.16	1.22E-03	0.79	6.46E-02	0.75	1.20E-01
MSMEG_3687	medium chain acyl-CoA synthetase		1.20	4.83E-02	0.74	4.07E-02	0.97	6.68E-01
MSMEG_3688	conserved hypothetical protein		1.21	2.08E-01	1.13	3.64E-01	0.49	5.40E-04
MSMEG_3689	sodium:solute symporter		0.67	6.46E-03	1.28	8.56E-02	0.42	3.58E-04
MSMEG_3690	transcriptional regulatory protein; this gene contains a frame shift which is not the result of sequencing error; identified by match to protein family HMM PF08220		0.69	1.50E-02	0.88	2.38E-01	0.56	8.14E-03
MSMEG_3691	galactose-1-phosphate uridylyltransferase	*galT*	0.87	2.04E-01	1.10	6.66E-01	0.93	1.28E-01
MSMEG_3692	galactokinase	*galK*	0.74	2.83E-01	1.08	2.84E-01	1.10	1.49E-01
MSMEG_3693	cytochrome P450 monooxygenase		1.31	1.02E-01	1.01	8.77E-01	1.01	6.34E-01
MSMEG_3694	feruloyl-CoA synthetase		1.15	1.85E-01	1.02	8.19E-01	1.44	2.29E-01
MSMEG_3695	conserved hypothetical protein		1.92	5.82E-02	0.95	7.54E-01	1.14	4.38E-01
MSMEG_3698	hypothetical protein		1.32	2.10E-01	1.03	6.72E-01	1.37	3.35E-01
MSMEG_3699	conserved hypothetical protein		1.34	9.00E-02	1.00	9.47E-01	0.96	5.96E-01

MSMEG_3700	peroxidase		0.97	4.84E-01	1.23	2.32E-01	1.12	1.42E-01
MSMEG_3701	conserved hypothetical protein		0.97	7.27E-01	1.09	6.14E-01	0.97	5.25E-01
MSMEG_3703	hypothetical protein		0.68	3.01E-03	0.61	3.63E-03	0.89	2.13E-02
MSMEG_3703	hypothetical protein		1.19	4.81E-01	1.25	3.82E-01	2.06	3.53E-01
MSMEG_3704	3-alpha-hydroxysteroid dehydrogenase		0.39	1.97E-04	1.00	9.92E-01	1.01	9.53E-01
MSMEG_3705	major facilitator superfamily protein MFS_1		0.25	3.18E-06	0.86	1.90E-01	1.12	4.88E-01
MSMEG_3706	isocitrate lyase	*aceA*	3.75	1.03E-02	1.31	2.61E-01	0.20	9.44E-05
MSMEG_3707	conserved hypothetical protein		3.71	3.82E-02	1.11	4.15E-01	0.93	8.17E-01
MSMEG_3708	catalase		0.60	4.44E-03	0.80	2.90E-01	0.41	2.03E-02
MSMEG_3709	conserved hypothetical protein		0.82	6.16E-02	1.69	4.20E-01	1.13	5.50E-01
MSMEG_3710	cytochrome b561 family protein		0.83	6.71E-02	0.88	3.02E-01	0.57	8.27E-03
MSMEG_3711	hypothetical protein		6.49	3.44E-01	1.34	6.49E-02	0.95	2.98E-01
MSMEG_3712	hypothetical protein		1.10	5.71E-01	1.01	9.02E-01	1.14	4.98E-01
MSMEG_3713	hypothetical protein		0.69	1.51E-02	1.16	2.37E-01	0.99	5.55E-01
MSMEG_3714	conserved hypothetical protein		1.34	4.31E-02	0.94	5.86E-01	0.87	2.59E-01
MSMEG_3715	linear gramicidin synthetase subunit C		1.07	4.57E-02	1.10	1.24E-01	0.84	4.75E-02
MSMEG_3716	hypothetical protein		0.86	4.60E-02	1.10	3.00E-01	1.15	3.38E-01
MSMEG_3717	short-chain dehydrogenase/reductase SDR; this gene contains a frame shift which is not the result of sequencing error		2.44	8.61E-03	1.44	1.42E-02	0.70	5.90E-02
MSMEG_3717	short-chain dehydrogenase/reductase SDR; this gene contains a frame shift which is not the result of sequencing error		1.18	2.56E-01	1.06	6.14E-01	1.53	3.84E-01
MSMEG_3718	conserved hypothetical protein		2.23	4.45E-03	0.80	5.10E-03	1.47	5.98E-02
MSMEG_3719	sodium/calcium exchanger protein		1.21	2.65E-01	0.75	8.65E-03	0.88	6.63E-01
MSMEG_3720	recombination activating protein 1		1.31	3.18E-01	1.00	1.00E+00	1.07	1.15E-01
MSMEG_3721	coenzyme PQQ biosynthesis protein E	*pqqE*	1.14	5.80E-01	0.88	1.11E-01	1.24	2.04E-01
MSMEG_3722	bifunctional coenzyme PQQ synthesis protein C/D		1.10	5.88E-01	1.04	6.93E-01	0.95	2.05E-01
MSMEG_3723	coenzyme PQQ biosynthesis protein C	*pqqC*	1.26	1.85E-01	0.82	9.26E-02	1.30	1.75E-01
MSMEG_3724	coenzyme PQQ biosynthesis protein B	*pqqB*	1.15	1.39E-01	3.06	3.55E-01	1.42	3.56E-01
MSMEG_3725	coenzyme PQQ biosynthesis protein A	*pqqA*	1.46	1.05E-02	0.62	4.36E-02	1.06	1.35E-01
MSMEG_3726	alcohol dehydrogenase		1.82	9.04E-03	0.81	7.83E-02	2.19	2.69E-01
MSMEG_3727	hypothetical protein		1.73	9.18E-02	0.91	3.59E-01	1.18	1.17E-01
MSMEG_3728	hypothetical protein		1.09	3.14E-01	1.05	6.58E-01	0.98	7.68E-01
MSMEG_3729	catalase/peroxidase HPI	*katG*	2.69	4.70E-02	0.90	1.74E-01	1.09	2.79E-01
MSMEG_3730	helix-turn-helix, Fis-type, putative		5.03	2.54E-03	0.92	5.44E-01	0.71	8.70E-02
MSMEG_3731	dipeptidase 2		1.71	3.04E-01	1.25	1.88E-01	9.56	3.19E-01

MSMEG_3732	hypothetical protein		0.99	9.07E-01	1.11	4.81E-01	1.00	9.48E-01
MSMEG_3733	conserved hypothetical protein		1.12	9.00E-02	0.79	3.42E-02	2.62	8.18E-04
MSMEG_3735	YtnM protein		0.53	4.00E-03	1.08	6.80E-01	6.23	6.69E-03
MSMEG_3736	hypothetical protein		0.84	1.85E-03	1.06	8.13E-01	4.66	2.17E-03
MSMEG_3737	integral membrane protein		0.44	3.85E-04	0.85	1.32E-01	1.70	2.72E-02
MSMEG_3738	GTP-binding protein EngA		1.02	7.05E-01	0.74	3.80E-03	4.33	2.35E-02
MSMEG_3739	cytidylate kinase	*cmk*	1.20	2.70E-02	0.82	4.56E-02	3.84	1.08E-02
MSMEG_3740	ribosomal large subunit pseudouridine synthase B	*rluB*	1.33	1.20E-02	0.83	3.55E-02	3.90	7.85E-04
MSMEG_3741	transcriptional regulator		1.25	5.29E-03	0.74	3.35E-02	3.00	4.44E-03
MSMEG_3742	segregation and condensation protein A	*scpA*	0.58	1.01E-02	1.20	6.83E-02	2.61	1.53E-02
MSMEG_3743	SpoOJ regulator protein	*soj*	0.92	2.30E-01	0.92	3.51E-01	5.55	2.06E-01
MSMEG_3744	tyrosine recombinase XerD	*xerD*	0.60	2.22E-03	1.33	2.82E-01	4.64	1.39E-01
MSMEG_3745	MutT/nudix family protein		0.57	7.74E-04	1.06	6.46E-01	4.96	5.50E-03
MSMEG_3746	CTP synthase	*pyrG*	0.74	2.24E-03	0.93	3.19E-01	1.94	1.69E-02
MSMEG_3747	conserved hypothetical protein; this gene contains a frame shift which is not the result of sequencing error		0.92	7.87E-01	1.00	9.77E-01	1.55	5.62E-01
MSMEG_3748	thiamin pyrophosphokinase, catalytic domain protein		1.04	8.66E-01	0.80	2.45E-01	1.57	1.86E-01
MSMEG_3749	DNA repair protein RecN; this gene contains a frame shift which is not the result of sequencing error		0.73	4.16E-03	0.86	1.42E-01	3.17	5.13E-04
MSMEG_3749	DNA repair protein RecN; this gene contains a frame shift which is not the result of sequencing error		0.91	3.55E-02	0.89	2.46E-01	4.64	5.26E-03
MSMEG_3750	inorganic polyphosphate/ATP-NAD kinase (Poly(P)/ATP NADkinase)		0.74	6.48E-03	0.85	4.15E-02	3.25	3.94E-04
MSMEG_3751	hemolysin A		0.76	1.05E-03	0.96	5.84E-01	2.36	5.13E-03
MSMEG_3752	conserved hypothetical protein		1.12	2.71E-02	0.81	1.50E-01	1.77	6.29E-02
MSMEG_3753	hydrolase		0.57	4.16E-05	0.70	1.32E-02	1.10	4.56E-01
MSMEG_3754	TPR-repeat-containing protein		0.39	5.72E-03	1.03	8.37E-01	0.98	6.71E-01
MSMEG_3758	tyrosyl-tRNA synthetase	*tyrS*	0.53	3.13E-05	1.09	1.15E-01	1.24	1.09E-01
MSMEG_3759	3-methyladenine DNA glycosylase		0.67	1.06E-01	1.17	3.69E-01	1.18	3.20E-01
MSMEG_3760	conserved hypothetical protein		1.13	1.43E-01	1.05	4.89E-01	1.19	4.52E-01
MSMEG_3761	putative Clp protease subunit		1.02	9.19E-01	0.83	4.38E-02	0.91	4.58E-01
MSMEG_3762	ABC transporter ATP-binding protein		2.04	2.15E-02	0.70	1.44E-01	1.21	2.25E-01

MSMEG_3763	ABC transporter efflux protein, DrrB family protein		2.03	6.68E-03	0.50	2.64E-02	1.58	2.40E-01
MSMEG_3764	conserved hypothetical protein		0.92	2.09E-01	0.95	4.85E-01	1.06	3.87E-01
MSMEG_3765	transcriptional regulator		1.10	3.78E-01	0.62	1.37E-02	1.04	6.53E-01
MSMEG_3766	hypothetical protein		1.24	4.00E-03	0.85	1.67E-01	0.67	1.86E-05
MSMEG_3767	acyl-CoA synthase		0.77	1.52E-02	0.85	1.98E-01	1.27	1.85E-01
MSMEG_3768	macrolide-transport ATP-binding protein abc transporter		0.68	2.27E-02	0.95	5.23E-01	1.71	6.06E-02
MSMEG_3769	argininosuccinate lyase	*argH*	0.54	1.40E-03	0.89	5.71E-01	1.06	7.22E-01
MSMEG_3770	argininosuccinate synthase	*argG*	0.80	2.14E-01	0.80	2.96E-01	0.88	1.73E-01
MSMEG_3771	arginine repressor	*argR*	0.62	3.98E-03	0.86	4.07E-01	2.01	4.68E-02
MSMEG_3772	ornithine carbamoyltransferase	*argF*	0.62	9.73E-03	0.86	4.71E-01	1.39	6.23E-02
MSMEG_3773	acetylornithine aminotransferase	*argD*	0.78	8.29E-02	0.85	4.11E-01	1.34	1.77E-01
MSMEG_3774	acetylglutamate kinase	*argB*	0.84	1.95E-01	0.83	4.15E-01	0.98	8.74E-01
MSMEG_3775	arginine biosynthesis bifunctional protein ArgJ	*argJ*	1.04	6.50E-01	0.77	2.15E-01	1.15	3.95E-01
MSMEG_3776	N-acetyl-gamma-glutamyl-phosphate reductase	*argC*	1.16	2.63E-01	0.66	7.74E-02	1.21	5.36E-01
MSMEG_3777	phenylalanyl-tRNA synthetase, beta subunit	*pheT*	0.38	7.57E-05	1.04	5.61E-01	1.11	6.07E-01
MSMEG_3778	phenylalanyl-tRNA synthetase, alpha subunit	*pheS*	0.42	2.97E-05	0.97	8.06E-01	1.45	3.52E-01
MSMEG_3779	conserved hypothetical protein; this gene contains a frame shift which is not the result of sequencing error		0.58	6.89E-04	0.92	2.90E-01	1.28	4.37E-01
MSMEG_3780	adenylate and Guanylate cyclase catalytic domain protein		0.93	2.68E-01	0.94	5.63E-01	8.16	3.60E-01
MSMEG_3781	conserved hypothetical protein		5.78	6.48E-03	0.69	2.07E-02	0.71	1.83E-01
MSMEG_3782	endoribonuclease L-PSP, putative		0.92	2.72E-01	1.04	5.98E-01	0.95	5.76E-01
MSMEG_3783	acyl-CoA dehydrogenase		1.06	4.84E-01	0.90	3.80E-01	0.94	5.02E-01
MSMEG_3784	transcriptional regulator, IclR family protein		0.82	3.43E-02	1.01	8.65E-01	1.01	7.27E-01
MSMEG_3785	PfkB-family protein carbohydrate kinase		1.43	4.54E-02	0.97	5.56E-01	1.18	4.29E-01
MSMEG_3786	D-amino acid deaminase		1.22	2.29E-01	1.02	4.63E-01	0.92	5.63E-01
MSMEG_3787	D-aminoacylase		0.95	5.35E-01	1.15	2.80E-01	0.99	8.41E-01
MSMEG_3788	2-dehydro-3-deoxyphosphogluconate aldolase/4-hydroxy-2-oxoglutarate aldolase	*eda*	0.83	9.54E-03	1.05	6.92E-01	1.29	1.33E-01
MSMEG_3789	inner membrane permease YgbN		0.92	1.34E-01	1.55	3.09E-01	1.19	5.00E-01
MSMEG_3790	RNA methyltransferase, TrmH family protein		0.34	2.02E-06	1.18	3.44E-01	2.05	7.12E-02
MSMEG_3791	ribosomal protein L20	*rplT*	0.68	4.68E-05	0.66	1.18E-02	0.76	1.74E-02
MSMEG_3792	ribosomal protein L35	*rpmI*	1.09	1.31E-01	1.02	7.40E-01	1.07	5.01E-01
MSMEG_3793	translation initiation factor IF-3	*infC*	1.90	4.51E-03	0.82	9.66E-02	0.88	2.86E-01
MSMEG_3794	hypothetical protein		1.86	3.86E-03	0.74	5.02E-02	1.05	5.50E-01

MSMEG_3795	conserved hypothetical protein		0.83	3.93E-02	0.94	4.42E-01	1.03	7.32E-01
MSMEG_3796	lysyl-tRNA synthetase		0.59	1.39E-02	0.93	4.05E-01	0.84	1.61E-01
MSMEG_3797	putative esterase family protein		1.37	2.19E-02	0.78	4.90E-02	0.67	7.23E-02
MSMEG_3798	conserved hypothetical protein		1.11	3.94E-01	0.89	4.08E-02	1.37	1.56E-02
MSMEG_3799	conserved hypothetical protein		2.02	4.58E-03	0.88	2.13E-01	1.08	6.93E-01
MSMEG_3800	conserved hypothetical protein		1.23	3.23E-02	1.19	2.51E-01	0.98	8.93E-01
MSMEG_3801	conserved hypothetical protein		0.88	3.83E-01	0.82	2.55E-01	1.13	3.75E-01
MSMEG_3802	tetratricopeptide repeat family protein		1.12	2.56E-01	0.94	2.29E-01	1.23	8.37E-02
MSMEG_3803	lipoprotein LpqH		0.86	2.02E-02	1.15	3.53E-01	1.30	1.39E-01
MSMEG_3804	conserved hypothetical protein		1.60	3.31E-02	0.79	9.36E-02	1.15	4.83E-01
MSMEG_3805	oxidoreductase, short chain dehydrogenase/reductase family protein		1.14	9.00E-02	1.09	4.34E-01	1.03	7.36E-01
MSMEG_3806	conserved hypothetical protein		1.30	1.47E-01	1.01	9.47E-01	1.89	1.11E-01
MSMEG_3807	TetR-type regulator		1.46	7.36E-02	0.99	8.94E-01	1.05	5.70E-01
MSMEG_3808	excinuclease ABC, A subunit	*uvrA*	1.12	9.10E-02	0.88	8.08E-02	2.03	7.98E-03
MSMEG_3809	conserved hypothetical protein		1.39	3.68E-01	0.91	2.52E-01	1.07	8.13E-01
MSMEG_3810	hydrolase		0.55	2.29E-04	1.09	2.26E-01	1.31	9.48E-03
MSMEG_3811	universal stress protein family protein, putative		0.38	5.28E-05	0.86	1.70E-01	6.45	8.39E-04
MSMEG_3812	acyl-CoA thioesterase		0.38	6.87E-03	0.98	8.15E-01	1.13	1.24E-01
MSMEG_3813	NAD-dependent epimerase/dehydratase		1.31	2.97E-01	2.63	4.33E-01	1.12	4.52E-01
MSMEG_3815	possible drug efflux membrane protein		0.81	1.24E-03	1.05	6.24E-01	1.81	4.74E-02
MSMEG_3816	excinuclease ABC, B subunit	*uvrB*	2.11	3.19E-03	0.89	8.83E-02	0.87	1.94E-01
MSMEG_3817	conserved hypothetical protein		1.45	1.45E-01	0.90	1.66E-01	1.01	9.37E-01
MSMEG_3818	thermolabile glutaminase		1.06	4.99E-01	1.20	3.51E-01	0.97	7.59E-01
MSMEG_3819	conserved hypothetical protein		2.16	1.31E-03	1.05	7.78E-01	0.72	3.67E-03
MSMEG_3820	hypothetical protein		1.08	2.31E-01	1.08	5.58E-01	1.00	9.64E-01
MSMEG_3821	amino acid permease		1.85	1.42E-01	0.83	3.37E-02	1.22	3.73E-01
MSMEG_3822	regulatory protein GntR, HTH		0.88	4.73E-01	0.91	3.58E-01	0.97	6.11E-01
MSMEG_3823	3-oxoacyl-[acyl-carrier-protein] reductase		1.37	3.50E-01	1.12	4.94E-01	0.95	1.95E-01
MSMEG_3824	conserved hypothetical protein		0.89	5.87E-02	1.00	9.80E-01	1.01	8.66E-01
MSMEG_3825	3-oxoacyl-[acyl-carrier-protein] reductase		0.96	7.52E-01	0.92	4.68E-01	1.17	4.69E-01
MSMEG_3826	hypothetical protein		0.88	4.79E-01	0.94	2.72E-01	1.18	2.84E-01
MSMEG_3827	glutamine synthetase		1.81	2.37E-03	1.08	5.22E-01	0.52	8.93E-03
MSMEG_3828	glutamine synthetase		1.25	8.11E-02	0.89	1.00E-01	0.96	2.69E-01
MSMEG_3829	esterase		0.92	6.26E-01	1.17	5.38E-01	0.93	1.30E-01
MSMEG_3830	3-oxoacyl-[acyl-carrier-protein] reductase		0.93	2.56E-01	0.98	7.93E-01	0.90	3.03E-01

MSMEG_3831	dephospho-CoA kinase; this gene contains a frame shift which is not the result of sequencing error; identified by match to protein family HMM PF01121; match to protein family HMM TIGR00152	0.25	4.19E-06	1.07	7.26E-01	0.78	2.46E-01
MSMEG_3832	4-hydroxyacetophenone monooxygenase	1.20	5.01E-01	1.18	3.21E-01	1.04	3.57E-01
MSMEG_3833	30S ribosomal protein S1	0.87	2.80E-02	0.79	1.35E-02	0.49	1.99E-06
MSMEG_3834	putative TetR-family protein transcriptional regulator	1.23	1.42E-01	0.88	2.67E-01	1.12	7.61E-02
MSMEG_3835	YesF protein	1.34	1.43E-01	0.72	2.93E-03	1.41	1.93E-03
MSMEG_3836	conserved hypothetical protein	1.29	6.25E-02	0.73	1.31E-02	1.27	6.35E-02
MSMEG_3837	conserved hypothetical protein	0.58	3.57E-04	0.74	4.31E-02	2.10	1.00E-01
MSMEG_3838	integral membrane protein	1.20	2.99E-01	0.91	1.98E-01	0.77	7.25E-02
MSMEG_3839	DNA polymerase I	0.61	1.63E-03	0.95	4.50E-01	1.52	1.96E-02
MSMEG_3840	LysR-family protein transcriptional regulator	1.12	1.73E-01	0.92	3.08E-01	2.81	2.38E-02
MSMEG_3841	monooxygenase, FAD-binding	1.89	1.10E-01	0.92	4.99E-01	1.02	1.44E-02
MSMEG_3842	putative esterase	1.06	6.57E-01	1.17	4.26E-01	1.63	4.00E-01
MSMEG_3843	conserved hypothetical protein	0.77	4.33E-03	0.91	3.68E-01	1.00	9.71E-01
MSMEG_3844	lipid-transfer protein	0.79	7.76E-02	0.92	1.86E-01	0.94	6.80E-01
MSMEG_3845	conserved domain protein	0.52	1.04E-03	1.12	4.42E-01	0.98	8.78E-01
MSMEG_3846	phosphotransferase enzyme family protein	1.86	6.19E-02	1.12	5.57E-01	0.31	2.42E-03
MSMEG_3847	copper methylamine oxidase	2.05	9.39E-02	1.11	5.08E-01	0.86	5.74E-01
MSMEG_3848	para-nitrobenzyl esterase	1.06	5.12E-01	0.81	7.10E-02	0.82	2.84E-01
MSMEG_3849	hypothetical protein	0.90	1.60E-02	1.06	7.43E-01	0.98	2.35E-01
MSMEG_3850	alkanesulfonate monooxygenase family protein	0.99	9.18E-01	2.68	2.98E-01	2.01	1.85E-02
MSMEG_3851	LppI protein	1.02	8.73E-01	0.67	3.53E-02	1.48	3.88E-02
MSMEG_3852	aliphatic sulfonate binding protein	1.70	3.03E-03	1.86	1.18E-01	1.05	8.98E-01
MSMEG_3853	putative aliphatic sulfonates transport ATP-binding protein SsuB	1.24	3.31E-01	2.93	2.66E-01	1.86	3.06E-01
MSMEG_3854	putative aliphatic sulfonates transport permease protein SsuC	1.23	1.75E-01	1.64	2.81E-01	1.38	4.17E-02
MSMEG_3855	hypothetical protein	1.18	1.18E-01	1.08	4.67E-01	0.48	3.05E-03
MSMEG_3856	transcriptional regulator, CadC	1.48	8.55E-02	0.97	7.65E-01	2.88	3.24E-02
MSMEG_3857	conserved hypothetical protein	0.95	4.24E-01	1.48	6.74E-02	0.82	2.66E-01
MSMEG_3858	conserved hypothetical protein	1.51	5.38E-04	1.26	1.02E-01	1.40	2.85E-02
MSMEG_3859	glycosyl transferase, group 2 family protein	0.91	3.48E-02	0.71	9.13E-03	1.99	1.27E-03
MSMEG_3860	polyprenol-monophosphomannose synthase Ppm1	0.63	1.87E-03	1.12	2.63E-01	4.84	1.64E-02

MSMEG_3861	amidohydrolase 3		1.09	1.37E-01	1.22	3.30E-01	2.17	3.40E-03
MSMEG_3862	FxsA cytoplasmic membrane protein		2.27	4.07E-03	0.83	2.15E-02	0.79	1.07E-01
MSMEG_3863	pyridoxamine 5'-phosphate oxidase family		1.05	3.38E-01	0.89	9.03E-02	1.07	3.00E-01
MSMEG_3864	cobaltochelatase, CobN subunit	*cobN*	0.83	2.71E-01	0.96	2.68E-01	2.53	3.57E-02
MSMEG_3865	hypothetical protein		1.41	9.42E-02	0.81	5.53E-02	1.38	3.11E-01
MSMEG_3866	conserved hypothetical protein		1.62	6.08E-02	0.93	6.55E-01	1.45	1.07E-01
MSMEG_3867	ATPase family protein associated with various cellular activities (AAA)		2.26	3.73E-02	0.96	7.69E-01	1.49	9.91E-02
MSMEG_3868	conserved hypothetical protein		2.71	3.98E-04	1.02	8.56E-01	3.05	1.28E-01
MSMEG_3869	conserved hypothetical protein		2.27	2.81E-02	1.35	1.42E-01	1.29	3.06E-01
MSMEG_3870	alpha-ketoglutarate-dependent taurine		1.01	7.86E-01	7.97	2.44E-01	1.74	1.79E-01
MSMEG_3871	precorrin-3B synthase	*cobG*	1.65	1.78E-03	0.75	8.21E-03	4.40	1.93E-01
MSMEG_3872	precorrin-8X methylmutase		0.47	1.74E-04	0.94	5.37E-01	1.58	1.99E-02
MSMEG_3873	cobalamin biosynthesis protein cobIJ		0.54	1.45E-05	1.19	5.67E-01	1.70	1.28E-01
MSMEG_3874	transcriptional regulator, TetR family protein		2.21	4.37E-03	0.95	7.96E-01	0.80	4.67E-01
MSMEG_3875	precorrin-6x reductase	*cobK*	0.59	8.55E-03	0.94	7.31E-01	1.34	8.61E-02
MSMEG_3876	phosphotransferase enzyme family protein, putative		2.26	1.42E-02	0.72	9.58E-02	0.97	8.06E-01
MSMEG_3877	precorrin-4 C11-methyltransferase	*cobM*	0.53	2.05E-04	0.86	2.38E-01	1.36	4.83E-02
MSMEG_3878	precorrin-6Y C5,15-methyltransferase (decarboxylating)	*cobL*	0.75	2.96E-02	0.85	1.16E-02	1.66	8.29E-02
MSMEG_3879	short chain dehydrogenase		0.67	2.26E-03	0.73	2.92E-03	1.10	6.61E-01
MSMEG_3880	nitrilase/cyanide hydratase and apolipoprotein N-acyltransferase		0.83	2.33E-02	0.98	8.41E-01	1.41	4.88E-03
MSMEG_3881	proline dipeptidase		1.20	3.67E-02	0.85	1.70E-01	0.93	4.72E-01
MSMEG_3882	glyoxalase family protein		2.24	2.18E-03	0.79	5.54E-02	0.79	7.09E-03
MSMEG_3883	5'-3' exonuclease		0.82	4.94E-03	0.83	1.56E-01	1.13	5.63E-01
MSMEG_3884	conserved hypothetical protein		1.15	1.68E-02	0.81	1.18E-01	1.28	9.82E-02
MSMEG_3885	DEAD/DEAH box helicase		0.60	5.72E-03	1.03	5.38E-01	6.26	1.69E-02
MSMEG_3886	twin arginine-targeting protein translocase TatC	*tatC*	0.91	2.22E-01	0.81	8.68E-02	2.56	6.88E-04
MSMEG_3887	twin arginine-targeting protein translocase, TatA/E family protein	*tatA*	2.93	1.05E-03	0.75	6.77E-02	1.14	5.47E-01
MSMEG_3888	conserved hypothetical protein		0.76	1.53E-02	0.86	2.52E-01	1.92	8.89E-04
MSMEG_3889	conserved hypothetical protein; this gene contains a frame shift which is not the result of sequencing error		0.73	3.69E-02	0.90	3.77E-01	2.12	8.62E-04

MSMEG_3889	conserved hypothetical protein; this gene contains a frame shift which is not the result of sequencing error		0.94	2.67E-01	1.05	5.97E-01	1.72	3.22E-03
MSMEG_3890	proteasome component		1.65	1.91E-02	0.91	2.44E-01	0.66	1.47E-02
MSMEG_3891	hydrolase		1.76	4.58E-02	1.15	1.47E-01	0.33	1.36E-03
MSMEG_3892	hypothetical protein		0.65	2.36E-03	1.21	9.09E-02	1.01	2.66E-01
MSMEG_3893	hypothetical protein		0.72	4.96E-02	1.10	4.71E-01	0.96	4.67E-01
MSMEG_3894	proteasome alpha subunit		0.80	7.80E-03	1.04	4.74E-01	0.98	9.08E-01
MSMEG_3895	proteasome beta subunit		0.96	6.52E-01	0.84	1.27E-01	1.13	5.39E-01
MSMEG_3896	conserved hypothetical protein		1.93	4.51E-04	0.77	1.34E-01	0.85	4.24E-01
MSMEG_3897	proteasome component		1.80	3.68E-01	1.89	3.98E-01	1.90	4.13E-01
MSMEG_3898	ectoine hydroxylase	*thpD*	3.19	5.48E-02	0.64	2.75E-01	1.74	1.41E-01
MSMEG_3899	ectoine synthase	*ectC*	5.86	5.84E-02	0.82	6.66E-01	1.80	3.91E-02
MSMEG_3900	diaminobutyrate--2-oxoglutarate aminotransferase	*ectB*	8.96	3.80E-02	0.71	4.64E-01	1.44	2.68E-01
MSMEG_3901	L-2,4-diaminobutyric acid acetyltransferase	*ectA*	11.20	1.10E-02	0.64	2.66E-01	1.57	1.67E-02
MSMEG_3902	ATPase, AAA family protein		1.29	1.81E-02	0.63	1.44E-02	2.02	8.48E-04
MSMEG_3903	Low molecular weight antigen MTB12		0.61	3.37E-03	0.75	3.38E-01	0.93	6.85E-01
MSMEG_3904	LppK protein		0.41	2.98E-04	0.90	3.64E-01	1.18	1.83E-01
MSMEG_3905	conserved hypothetical protein		0.53	4.13E-05	0.88	1.70E-01	1.23	1.29E-01
MSMEG_3906	tRNA (adenine-N(1)-)-methyltransferase		1.06	7.61E-01	1.18	6.30E-01	2.39	1.36E-01
MSMEG_3907	RecB family protein exonuclease		0.93	4.45E-01	0.92	4.09E-01	2.12	3.35E-02
MSMEG_3908	conserved hypothetical protein		0.48	1.46E-03	1.02	8.21E-01	2.39	3.00E-03
MSMEG_3909	conserved hypothetical protein		1.23	2.14E-01	0.99	8.60E-01	1.41	9.40E-02
MSMEG_3910	conserved hypothetical protein		0.92	4.42E-02	0.98	8.74E-01	0.82	1.68E-01
MSMEG_3911	oxidoreductase		1.18	8.96E-02	1.18	2.12E-01	1.08	5.55E-01
MSMEG_3912	acetoacetyl-CoA reductase		1.18	4.85E-01	1.18	1.57E-01	1.06	8.51E-02
MSMEG_3913	short chain dehydrogenase; this gene contains a frame shift which is not the result of sequencing error		1.55	1.42E-01	1.00	9.77E-01	1.07	5.75E-01
MSMEG_3914	beta-lactamase		2.60	1.10E-01	1.26	4.39E-01	0.93	4.23E-01
MSMEG_3915	NAD-dependent alcohol dehydrogenase		1.27	1.11E-01	1.15	4.66E-01	3.36	2.38E-01
MSMEG_3916	hypothetical protein		2.15	8.04E-03	0.91	6.40E-01	1.69	3.84E-02
MSMEG_3917	hypothetical protein		0.98	8.45E-01	1.11	4.35E-02	1.42	2.99E-02
MSMEG_3918	hypothetical protein		2.12	9.94E-04	0.90	2.68E-01	0.65	9.85E-03
MSMEG_3919	hypothetical protein		2.17	1.40E-03	1.04	4.71E-01	1.01	9.78E-01
MSMEG_3920	hypothetical protein		1.88	2.65E-03	1.04	7.36E-01	1.00	9.84E-01
MSMEG_3921	hypothetical protein		1.67	2.77E-01	1.20	7.44E-02	1.23	5.44E-01
MSMEG_3922	hypothetical protein		1.88	1.02E-03	1.03	5.80E-01	0.99	5.87E-01

MSMEG_3923	conserved hypothetical protein	0.61	1.99E-03	0.93	4.60E-01	1.14	5.66E-01
MSMEG_3924	peptide synthetase ScpsB, putative	0.93	5.79E-01	0.91	1.76E-01	1.11	3.11E-01
MSMEG_3925	AttM/AiiB family protein	1.67	4.01E-01	0.92	3.05E-01	0.97	3.67E-01
MSMEG_3926	cation-transporting ATPase Pma1	1.11	7.98E-02	4.70	1.51E-02	15.13	3.29E-02
MSMEG_3927	peptidase M52, hydrogen uptake protein	1.44	7.40E-02	5.19	4.29E-03	18.65	1.34E-02
MSMEG_3928	[NiFe] hydrogenase, alpha subunit, putative	2.57	1.35E-03	2.20	3.68E-02	7.58	3.33E-02
MSMEG_3929	[NiFe] hydrogenase, delta subunit, putative	1.01	9.38E-01	8.20	8.61E-03	26.95	6.51E-03
MSMEG_3930	[NiFe] hydrogenase, gamma subunit, putative	1.19	8.42E-02	10.00	2.23E-02	28.04	2.05E-02
MSMEG_3931	[NiFe] hydrogenase, beta subunit, putative	1.03	8.16E-01	16.74	1.27E-02	67.26	7.44E-02
MSMEG_3932	14 kDa antigen	2.04	8.89E-02	48.74	1.24E-02	57.70	3.78E-02
MSMEG_3933	conserved hypothetical protein	1.60	8.70E-02	9.88	1.51E-03	10.07	2.07E-01
MSMEG_3934	phosphoenolpyruvate synthase	1.60	3.13E-01	13.83	9.20E-02	22.71	8.38E-02
MSMEG_3935	conserved hypothetical protein	1.53	8.53E-02	11.26	4.19E-03	8.08	4.13E-03
MSMEG_3936	universal stress protein family protein	1.17	4.42E-01	3.58	5.99E-02	9.85	1.08E-02
MSMEG_3937	conserved hypothetical protein	1.67	1.95E-01	8.83	3.46E-02	18.94	3.75E-04
MSMEG_3938	hypothetical protein	1.46	3.09E-01	1.37	1.83E-01	0.94	3.08E-01
MSMEG_3939	universal stress protein family protein	1.36	3.48E-02	10.86	2.31E-02	9.21	4.21E-03
MSMEG_3940	universal stress protein family protein	1.18	2.41E-01	12.02	2.34E-02	24.01	6.46E-03
MSMEG_3941	GAF family protein	2.47	3.00E-02	0.99	9.24E-01	1.51	8.04E-02
MSMEG_3942	conserved hypothetical protein	2.70	3.47E-02	11.94	4.78E-02	11.87	8.93E-02
MSMEG_3943	conserved hypothetical protein	1.87	1.55E-01	8.09	6.27E-02	10.04	3.22E-02
MSMEG_3944	two component transcriptional regulatory protein devr	1.04	7.62E-01	4.18	3.89E-02	3.41	2.26E-03
MSMEG_3945	universal stress protein family protein	1.76	5.43E-02	8.79	2.78E-04	17.76	1.50E-03
MSMEG_3946	probable conserved transmembrane protein	1.50	6.79E-02	11.97	1.37E-02	15.34	2.11E-03
MSMEG_3947	6-phosphofructokinase isozyme 2	1.50	4.40E-02	1.90	3.80E-02	3.06	2.05E-02
MSMEG_3948	acyltransferase, ws/dgat/mgat subfamily protein	1.28	1.06E-01	3.17	3.77E-03	4.85	6.29E-02
MSMEG_3948	acyltransferase, ws/dgat/mgat subfamily protein	1.50	4.34E-01	6.19	2.03E-02	2.17	2.53E-01
MSMEG_3949	hypothetical protein	2.04	4.05E-01	25.78	5.38E-03	25.30	3.98E-02
MSMEG_3950	universal stress protein family protein	1.91	9.40E-02	11.62	2.39E-03	4.65	2.04E-02
MSMEG_3951	conserved hypothetical protein	1.18	2.07E-01	4.31	2.53E-02	4.73	1.44E-01
MSMEG_3952	conserved hypothetical protein	1.36	1.71E-01	16.15	1.35E-02	10.94	6.69E-02
MSMEG_3953	conserved hypothetical protein	1.19	4.05E-01	5.76	5.14E-02	7.29	2.67E-02
MSMEG_3954	trehalose 6-phosphate phosphorylase	1.27	1.07E-01	2.28	7.42E-02	1.77	1.67E-01
MSMEG_3955	conserved hypothetical protein	1.61	9.80E-02	5.68	7.77E-03	3.37	3.77E-02
MSMEG_3956	alkylhydroperoxidase AhpD	0.44	2.33E-03	1.70	5.66E-02	1.65	3.24E-02
MSMEG_3957	conserved 13e12 repeat family protein	1.34	2.15E-01	0.94	5.47E-01	1.02	9.01E-01

MSMEG_3958	glyoxalase/bleomycin resistance protein/dioxygenase	3.94	7.79E-04	0.68	1.14E-02	0.83	2.62E-01
MSMEG_3959	transcriptional regulator	1.22	2.90E-01	1.69	2.56E-01	1.06	3.64E-01
MSMEG_3960	transcriptional regulator	0.99	8.90E-01	1.28	1.97E-01	1.06	5.31E-01
MSMEG_3961	transcriptional regulator	1.06	6.48E-01	0.73	5.33E-02	0.94	5.28E-01
MSMEG_3962	lactate 2-monooxygenase	1.44	1.81E-01	0.69	7.04E-02	0.16	3.11E-05
MSMEG_3963	hypothetical protein	1.06	2.45E-01	1.13	5.11E-01	1.07	6.29E-01
MSMEG_3964	pyruvate dehydrogenase	0.97	8.83E-01	0.93	5.47E-01	0.60	2.59E-03
MSMEG_3965	hypothetical protein	1.16	2.29E-01	1.13	2.76E-01	0.26	9.60E-06
MSMEG_3966	hypothetical protein	1.37	1.39E-01	0.89	1.96E-01	0.23	1.38E-05
MSMEG_3967	TonB-dependent receptor	1.25	1.15E-01	1.02	8.57E-01	1.08	4.24E-01
MSMEG_3968	hypothetical protein	1.70	1.64E-01	1.29	2.11E-01	1.34	2.26E-01
MSMEG_3969	hypothetical protein	2.40	2.00E-01	0.84	3.70E-01	1.18	1.96E-01
MSMEG_3970	glutamyl-tRNA(Gln) amidotransferase subunit A	1.11	3.90E-01	0.94	5.40E-01	0.93	2.15E-01
MSMEG_3971	hypothetical protein	1.04	7.06E-01	2.39	1.23E-01	0.97	5.15E-02
MSMEG_3972	transcriptional regulator, LysR family protein	1.48	4.64E-02	1.46	1.61E-01	1.07	4.35E-01
MSMEG_3973	N-methylhydantoinase	3.17	1.64E-02	0.98	9.05E-01	0.54	5.78E-03
MSMEG_3974	hydantoin utilization protein A; this gene contains a frame shift which is not the result of sequencing error; identified by match to protein family HMM PF05378	3.65	3.94E-02	1.23	3.48E-01	0.25	4.37E-04
MSMEG_3975	regulatory protein, putative	1.07	3.97E-01	1.68	9.73E-02	0.94	3.44E-01
MSMEG_3976	DNA-binding protein	0.91	3.63E-01	1.04	5.93E-01	0.93	1.64E-01
MSMEG_3977	conserved hypothetical protein	0.98	8.87E-01	8.76	9.23E-02	1.50	4.79E-01
MSMEG_3978	beta-lactamase	0.57	1.45E-03	7.43	7.96E-02	0.90	5.43E-01
MSMEG_3979	alpha/beta hydrolase; this gene contains a frame shift which is not the result of sequencing error	0.65	2.15E-01	1.12	3.77E-01	1.27	5.19E-02
MSMEG_3980	transcriptional regulator, GntR family protein	1.22	1.84E-01	0.93	2.36E-01	0.70	4.67E-02
MSMEG_3981	L-carnitine dehydratase/bile acid-inducible protein F	1.80	1.14E-02	0.96	9.99E-04	1.03	8.24E-01
MSMEG_3982	acyl-CoA dehydrogenase	2.02	2.10E-02	1.03	7.84E-01	0.87	3.33E-01
MSMEG_3983	L-carnitine dehydratase/bile acid-inducible protein F; this gene contains a frame shift which is not the result of sequencing error; identified by match to protein family HMM PF02515	2.13	2.12E-03	0.93	3.32E-01	0.48	8.47E-04

MSMEG_3983	L-carnitine dehydratase/bile acid-inducible protein F; this gene contains a frame shift which is not the result of sequencing error; identified by match to protein family HMM PF02515		1.59	3.89E-02	1.00	9.58E-01	0.80	4.32E-02
MSMEG_3984	Transposase IS116/IS110/IS902 family protein		0.79	2.31E-03	1.43	8.95E-02	1.41	2.42E-02
MSMEG_3984	Transposase IS116/IS110/IS902 family protein		0.98	6.68E-01	1.17	2.87E-01	0.91	5.92E-01
MSMEG_3985	integral membrane transport protein		1.48	2.20E-02	1.53	9.58E-02	0.97	4.44E-01
MSMEG_3986	acetyl-coenzyme A synthetase		1.77	1.06E-02	1.88	1.03E-01	1.07	5.77E-01
MSMEG_3987	FAD dependent oxidoreductase		1.25	2.22E-01	1.40	2.69E-01	1.09	4.94E-01
MSMEG_3988	transcriptional regulator, AsnC family protein		1.01	9.35E-01	1.29	4.31E-01	1.24	3.73E-01
MSMEG_3989	peptidase M20D, amidohydrolase		0.89	3.56E-01	1.40	2.08E-01	1.03	6.13E-01
MSMEG_3990	putative transporter		1.07	5.62E-01	1.55	2.15E-01	1.80	3.80E-01
MSMEG_3991	cyclase		1.35	4.54E-02	1.06	4.61E-01	0.83	2.20E-01
MSMEG_3992	integral membrane transport protein		1.07	5.18E-01	1.03	7.72E-01	1.33	2.19E-01
MSMEG_3993	Asp/Glu racemase		0.59	2.24E-02	1.08	6.80E-01	1.08	2.77E-01
MSMEG_3994	short chain dehydrogenase		0.54	7.52E-04	1.27	5.98E-01	0.94	1.74E-01
MSMEG_3995	N-carbamoyl-L-amino acid amidohydrolase		0.67	1.46E-02	1.04	7.49E-01	1.08	5.90E-01
MSMEG_3996	dihydropyrimidinase	*hydA*	0.65	1.02E-01	1.10	4.56E-01	1.41	3.49E-01
MSMEG_3997	regulatory protein		0.42	4.46E-04	0.92	3.96E-01	0.89	3.13E-01
MSMEG_3998	MmsAB operon regulatory protein		0.71	4.12E-02	1.30	1.84E-01	0.95	5.96E-01
MSMEG_3999	ABC transporter periplasmic-binding protein YphF		1.41	7.02E-02	1.46	1.08E-01	7.36	3.75E-01
MSMEG_4000	hypothetical ABC transporter ATP-binding protein YphE		0.88	9.90E-02	1.18	1.94E-01	1.33	2.48E-01
MSMEG_4001	ribose transport system permease protein RbsC		0.94	5.14E-01	1.17	3.05E-01	1.27	2.06E-01
MSMEG_4002	oxidoreductase, zinc-binding dehydrogenase family protein		1.16	1.62E-01	1.08	5.06E-01	0.64	9.81E-02
MSMEG_4003	conserved hypothetical protein		1.10	1.64E-01	1.32	2.76E-03	1.07	5.34E-01
MSMEG_4004	3-oxoacyl-[acyl-carrier-protein] reductase		0.83	1.52E-01	1.38	2.40E-01	1.07	5.02E-01
MSMEG_4005	calcium-binding protein		0.83	1.53E-01	1.28	7.11E-02	1.04	4.24E-01
MSMEG_4006	transcriptional regulator, CdaR, putative		0.80	2.74E-01	1.36	2.91E-01	1.14	4.24E-01
MSMEG_4007	oxidoreductase, 2OG-Fe(II) oxygenase family protein		0.94	4.97E-01	1.01	9.36E-01	1.00	9.89E-01
MSMEG_4008	oxidoreductase, 2OG-Fe(II) oxygenase family protein		0.94	8.63E-01	1.36	6.53E-02	0.90	1.72E-01
MSMEG_4009	vanillate O-demethylase oxidoreductase		0.73	7.23E-02	1.07	4.21E-01	0.90	2.32E-01
MSMEG_4010	glyoxalase family protein		0.74	8.94E-02	1.19	1.80E-01	1.05	4.42E-01
MSMEG_4011	putative pyrimidine permease RutG		0.75	1.13E-01	1.27	2.17E-01	0.97	4.78E-01

MSMEG_4012	dihydropyrimidinase	*hydA*	1.08	6.57E-01	1.11	4.44E-01	0.93	2.60E-01
MSMEG_4013	oxidoreductase		2.46	3.79E-01	1.28	1.29E-01	1.07	3.65E-01
MSMEG_4014	N-carbamoyl-L-amino acid amidohydrolase		1.56	1.87E-02	1.01	8.70E-01	0.71	2.29E-02
MSMEG_4015	hypothetical protein		1.08	7.85E-02	1.13	3.02E-01	1.05	2.10E-01
MSMEG_4016	conserved hypothetical protein		1.58	8.08E-02	0.96	6.73E-01	0.81	1.65E-01
MSMEG_4017	BmyD protein		0.81	9.70E-02	1.09	2.78E-01	1.13	5.47E-01
MSMEG_4018	nucleotidyltransferase domain protein		1.09	2.43E-01	1.10	1.23E-01	1.42	2.13E-01
MSMEG_4019	AMP-dependent synthetase and ligase		1.10	6.40E-01	0.99	8.43E-01	0.90	3.55E-01
MSMEG_4020	enoyl-CoA hydratase/isomerase family protein		1.15	3.46E-01	1.12	1.52E-01	1.12	3.26E-01
MSMEG_4021	hypothetical protein		0.96	6.19E-01	1.31	1.03E-01	1.94	5.59E-01
MSMEG_4022	TetR-family protein transcriptional regulator		0.72	2.95E-02	1.05	3.23E-01	3.33	2.55E-01
MSMEG_4023	oxidoreductase		3.28	5.27E-02	0.77	1.91E-02	0.87	2.32E-01
MSMEG_4024	transcriptional regulator, TetR family protein		1.27	2.55E-01	0.98	8.96E-01	1.24	6.25E-03
MSMEG_4025	transcriptional regulator, LysR family protein		0.95	7.71E-01	1.27	2.57E-01	1.04	7.90E-01
MSMEG_4026	hypothetical protein		2.01	1.58E-02	0.83	7.56E-02	1.30	2.90E-01
MSMEG_4027	zinc-containing alcohol dehydrogenase superfamily protein		3.56	1.67E-02	0.88	5.95E-01	1.32	2.22E-01
MSMEG_4028	hypothetical protein		0.90	4.72E-01	1.24	3.18E-01	1.01	8.18E-01
MSMEG_4029	conserved hypothetical protein		0.95	5.66E-01	1.15	3.24E-01	0.95	3.44E-01
MSMEG_4030	conserved hypothetical protein		2.09	1.08E-02	2.35	9.86E-02	1.01	7.60E-01
MSMEG_4031	hypothetical protein		1.44	5.40E-02	1.06	8.30E-01	0.91	2.97E-01
MSMEG_4032	zinc-binding alcohol dehydrogenase		1.81	3.07E-03	2.35	1.74E-01	1.09	2.61E-01
MSMEG_4033	TetR-family protein transcriptional regulator		0.61	1.13E-02	1.56	4.34E-02	1.04	4.29E-01
MSMEG_4034	NAD dependent epimerase/dehydratase family protein		0.57	5.27E-04	1.29	1.89E-01	1.20	3.61E-01
MSMEG_4035	citrate synthase		0.89	3.91E-01	1.29	1.51E-01	1.03	4.75E-01
MSMEG_4036	crotonobetaine/carnitine-CoA ligase		1.08	6.69E-01	0.91	6.02E-01	1.08	3.80E-01
MSMEG_4037	hypothetical protein		1.19	4.06E-01	1.38	4.63E-02	0.80	1.57E-01
MSMEG_4038	vanillin dehydrogenase		1.25	1.44E-01	0.91	6.18E-03	1.63	1.67E-01
MSMEG_4039	aryl-alcohol dehydrogenase		1.08	6.20E-01	1.09	6.94E-02	1.34	2.35E-01
MSMEG_4040	nitroreductase		1.78	2.98E-01	0.97	6.79E-01	1.30	4.31E-01
MSMEG_4041	hydroxyquinol 1,2-dioxygenase		0.83	4.83E-02	1.60	1.30E-01	1.08	6.86E-01
MSMEG_4042	transcriptional regulator, GntR family protein		0.58	1.59E-04	0.94	5.27E-01	2.10	1.40E-01
MSMEG_4043	amidohydrolase 2		0.64	8.03E-02	0.83	1.20E-01	1.46	2.62E-01
MSMEG_4044	GAF domain protein		1.08	7.25E-01	1.27	4.49E-01	2.94	1.91E-01
MSMEG_4045	muconolactone delta-isomerase		0.90	5.67E-01	1.13	2.57E-01	0.96	3.15E-01
MSMEG_4046	L-carnitine dehydratase/bile acid-inducible protein F		1.19	2.25E-01	1.22	1.28E-01	1.13	3.48E-01

MSMEG_4047	dihydropteroate synthase	1.34	8.60E-02	1.34	2.39E-01	1.06	2.68E-01
MSMEG_4048	cyclase	1.55	1.67E-01	1.06	6.66E-01	1.44	4.43E-01
MSMEG_4049	sugar transporter, permease protein	1.41	6.37E-03	1.76	8.30E-02	1.42	4.64E-01
MSMEG_4050	hypothetical protein	0.71	2.82E-03	1.23	2.90E-01	1.05	1.38E-01
MSMEG_4051	hypothetical protein	1.41	1.34E-01	1.32	2.16E-01	1.00	8.91E-01
MSMEG_4052	hypothetical protein	0.99	9.40E-01	2.13	1.99E-01	1.34	2.77E-01
MSMEG_4053	transcriptional regulatory protein	0.57	1.54E-03	1.97	1.25E-01	2.42	1.36E-01
MSMEG_4054	hypothetical protein	0.86	3.11E-01	1.26	2.04E-01	1.89	8.31E-02
MSMEG_4055	short-chain dehydrogenase/reductase SDR	0.85	8.21E-03	1.01	8.81E-01	1.16	3.69E-01
MSMEG_4056	conserved secreted protein	0.87	3.72E-01	1.06	7.08E-01	1.17	3.89E-01
MSMEG_4057	transcriptional regulator, GntR family protein	1.53	1.02E-01	0.82	3.00E-02	1.39	4.36E-01
MSMEG_4058	major facilitator superfamily protein	1.07	6.66E-01	1.13	2.38E-01	1.12	9.21E-02
MSMEG_4059	haloacid dehalogenase, type II	0.77	1.99E-01	0.96	8.10E-01	1.23	4.79E-01
MSMEG_4060	aspartate aminotransferase; this gene contains a frame shift which is not the result of sequencing error; identified by match to protein family HMM PF00155	0.66	2.37E-02	0.85	2.56E-02	0.95	4.31E-01
MSMEG_4060	aspartate aminotransferase; this gene contains a frame shift which is not the result of sequencing error; identified by match to protein family HMM PF00155	0.90	4.96E-01	1.35	1.24E-01	1.14	4.54E-01
MSMEG_4061	translation initiation inhibitor	1.11	4.81E-01	0.99	9.68E-01	1.00	8.91E-01
MSMEG_4062	glutamyl-tRNA(Gln) amidotransferase subunit A	0.83	8.78E-02	1.00	9.86E-01	1.06	5.35E-01
MSMEG_4063	amidohydrolase family protein	2.41	7.89E-02	1.35	3.97E-01	0.87	5.35E-01
MSMEG_4064	zinc-binding alcohol dehydrogenase	1.13	9.49E-02	1.42	3.81E-01	1.05	4.36E-01
MSMEG_4065	feruloyl-CoA synthetase	0.93	6.40E-01	1.11	2.24E-01	1.17	3.57E-01
MSMEG_4066	conserved hypothetical protein	0.89	5.27E-01	1.02	7.12E-01	1.02	6.65E-01
MSMEG_4067	zinc-containing alcohol dehydrogenase superfamily protein	1.12	1.12E-01	0.89	1.11E-01	0.71	3.45E-02
MSMEG_4068	hypothetical protein	1.09	6.95E-01	0.92	3.61E-01	0.96	6.24E-01
MSMEG_4069	hypothetical protein	1.28	2.66E-01	1.15	3.02E-01	1.10	5.99E-01
MSMEG_4070	transcriptional regulator, TetR family protein, putative	1.03	6.70E-01	1.05	6.56E-01	3.80	3.38E-01
MSMEG_4071	conserved hypothetical protein; this region contains one or more premature stops and/or frameshifts which are not the result of sequencing error	0.73	6.78E-02	1.30	1.35E-01	1.06	2.42E-01
MSMEG_4072	ISMsm5, transposase	0.81	4.16E-02	0.98	8.29E-01	0.81	5.16E-03

MSMEG_4073	DNA-binding protein; this gene contains a frame shift which is not the result of sequencing error		0.98	7.90E-01	1.18	3.98E-01	1.38	8.83E-02
MSMEG_4074	peroxisomal trans-2-enoyl-CoA reductase		1.54	5.02E-02	1.10	5.82E-01	1.40	2.92E-01
MSMEG_4075	CoA-binding protein		1.02	8.98E-01	1.19	1.14E-01	0.91	6.46E-01
MSMEG_4076	butyryl-CoA dehydrogenase		1.83	1.85E-02	1.26	2.02E-01	0.77	7.59E-02
MSMEG_4077	enoyl-CoA hydratase		1.41	3.39E-01	1.76	2.14E-01	1.01	4.99E-01
MSMEG_4078	hypothetical metabolite transport protein YaaU		1.41	1.94E-01	1.10	6.57E-01	0.96	8.40E-01
MSMEG_4079	putative hydrolase		0.66	4.72E-03	1.06	6.12E-01	1.94	2.10E-02
MSMEG_4080	fmnh2-utilizing oxygenase; this gene contains a frame shift which is not the result of sequencing error		0.84	7.14E-02	1.05	6.30E-01	1.18	2.51E-01
MSMEG_4082	monoxygenase		0.35	4.24E-05	5.03	2.34E-01	1.47	3.11E-01
MSMEG_4083	putative monooxygenase		0.37	2.64E-04	3.76	2.27E-01	1.37	2.57E-01
MSMEG_4084	putative acyl-CoA dehydrogenase		0.54	1.96E-03	4.85	2.29E-01	1.02	3.82E-01
MSMEG_4085	nitrilotriacetate monooxygenase component A		0.22	4.44E-06	7.69	2.72E-01	0.76	2.52E-01
MSMEG_4086	nitrilotriacetate monooxygenase component A	*ssuD*	1.29	7.84E-01	6.15	2.39E-01	0.94	6.65E-01
MSMEG_4087	major facilitator superfamily protein		0.24	5.50E-06	8.13	1.98E-01	1.10	4.66E-01
MSMEG_4090	putative IclR family protein transcriptional regulator		0.98	9.29E-01	0.93	5.90E-01	0.83	4.49E-01
MSMEG_4091	nitrilotriacetate monooxygenase component A	*ssuD*	1.12	2.40E-01	0.96	6.48E-01	1.03	6.39E-01
MSMEG_4092	alkanal monooxygenase		1.36	4.12E-01	0.80	4.84E-02	1.06	6.10E-01
MSMEG_4093	oxidoreductase, aldo/keto reductase family		1.48	6.35E-02	0.75	6.95E-02	0.99	4.24E-01
MSMEG_4094	acyl-CoA dehydrogenase		1.05	2.72E-01	0.94	6.99E-01	6.00	3.80E-01
MSMEG_4095	putative monooxygenase		2.31	2.61E-01	1.14	6.05E-02	1.48	3.34E-01
MSMEG_4096	nitrilotriacetate monooxygenase component A	*ssuD*	1.00	9.94E-01	1.13	1.54E-01	1.02	5.33E-01
MSMEG_4097	acyl-CoA dehydrogenase domain protein		0.97	7.43E-01	1.22	1.52E-01	1.03	1.38E-01
MSMEG_4098	ABC transporter ATP-binding protein		1.15	5.95E-02	1.12	5.20E-01	1.07	1.23E-01
MSMEG_4099	ABC transporter permease protein		1.06	5.84E-01	1.06	6.37E-01	1.03	4.50E-01
MSMEG_4100	ABC transporter permease protein		0.68	6.59E-02	1.28	1.10E-01	1.16	3.81E-01
MSMEG_4101	ABC-type transporter, substrate binding protein		0.93	3.83E-01	1.13	4.93E-01	1.00	9.64E-01
MSMEG_4102	conserved hypothetical protein		1.08	7.12E-01	1.02	7.47E-01	1.00	9.82E-01
MSMEG_4103	alkanesulfonate monooxygenase		1.24	2.08E-01	1.07	6.84E-01	0.97	6.27E-01
MSMEG_4104	transporter, major facilitator family protein		1.23	1.96E-02	1.38	1.30E-01	0.97	4.28E-01
MSMEG_4105	transposase subunit		0.51	6.31E-04	1.11	3.24E-01	1.49	6.71E-02
MSMEG_4105	transposase subunit		0.89	3.47E-01	0.95	6.88E-01	0.92	3.22E-01
MSMEG_4106	transposase		0.69	3.84E-04	1.35	2.86E-02	0.74	6.65E-02
MSMEG_4106	transposase		0.98	9.09E-01	1.02	8.26E-01	1.03	7.78E-01
MSMEG_4107	phosphoglycerate mutase, putative		0.66	3.93E-03	1.13	1.30E-01	0.99	6.73E-01

MSMEG_4108	NAD(P) transhydrogenase, beta subunit	*pntB*	0.89	3.93E-01	0.95	5.25E-01	1.03	6.24E-02
MSMEG_4109	NAD(P) transhydrogenase, alpha subunit	*pntA*	0.90	2.89E-01	1.03	7.00E-01	1.13	7.09E-01
MSMEG_4110	3-hydroxyacyl-CoA dehydrogenase		1.01	9.07E-01	0.88	7.89E-02	0.92	8.97E-02
MSMEG_4111	alpha-methylacyl-CoA racemase		1.08	1.46E-01	1.03	8.48E-01	0.67	8.17E-02
MSMEG_4112	cyclohexanecarboxylate-CoA ligase		1.62	2.71E-03	0.90	2.02E-01	0.74	1.21E-01
MSMEG_4113	acyl-CoA dehydrogenase		1.34	3.18E-02	0.81	1.26E-01	0.91	7.62E-01
MSMEG_4114	naphthoate synthase	*menB*	1.38	1.12E-01	0.96	7.34E-01	0.65	7.03E-02
MSMEG_4115	3-hydroxybutyryl-CoA dehydrogenase		1.38	1.55E-02	0.77	6.55E-02	0.60	2.68E-02
MSMEG_4116	3-hydroxybutyryl-CoA dehydrogenase		1.73	5.18E-03	0.71	2.02E-02	0.53	6.54E-02
MSMEG_4117	2-deoxy-D-gluconate 3-dehydrogenase		1.76	1.48E-02	0.87	2.26E-01	0.72	1.06E-01
MSMEG_4118	acyl-CoA dehydrogenase		1.08	5.17E-01	1.07	7.13E-01	0.80	1.31E-01
MSMEG_4119	3-hydroxybutyryl-CoA dehydratase		1.36	2.15E-02	0.90	3.98E-01	0.88	9.57E-02
MSMEG_4120	CAIB/BAIF family protein		2.17	5.83E-03	0.87	2.41E-01	0.92	1.58E-01
MSMEG_4121	GntR-family protein transcriptional regulator		2.07	1.50E-04	0.68	3.82E-03	2.24	3.65E-01
MSMEG_4122	major facilitator family protein transporter		3.81	1.80E-03	0.73	1.21E-02	0.41	8.89E-03
MSMEG_4123	3-hydroxyisobutyrate dehydrogenase		2.56	5.04E-03	0.70	2.54E-03	1.02	8.97E-01
MSMEG_4124	conserved hypothetical protein		1.86	3.89E-03	0.61	1.38E-03	1.12	5.03E-02
MSMEG_4125	oxidoreductase, short chain dehydrogenase/reductase family protein		1.94	6.84E-03	0.55	7.25E-03	1.05	7.74E-01
MSMEG_4126	hydrolase, isochorismatase family protein		4.46	3.12E-04	0.39	1.89E-03	0.59	3.98E-02
MSMEG_4127	TnpC protein		0.36	2.06E-05	1.08	4.28E-01	1.72	3.42E-01
MSMEG_4128	conserved hypothetical protein		0.63	1.23E-02	0.78	1.04E-01	0.73	2.10E-01
MSMEG_4129	hypothetical protein		0.50	3.52E-04	0.94	5.01E-01	0.89	3.79E-01
MSMEG_4130	polysaccharide deacetylase family protein		0.84	1.82E-01	1.08	6.32E-01	0.99	3.10E-01
MSMEG_4131	hypothetical protein		0.74	3.83E-01	1.08	4.09E-01	1.22	4.79E-01
MSMEG_4133	transposase		0.54	2.35E-04	0.98	7.21E-01	1.03	7.24E-01
MSMEG_4134	TnpC protein; this gene contains a frame shift which is not the result of sequencing error		0.61	4.24E-03	0.75	1.82E-02	0.88	1.09E-01
MSMEG_4134	TnpC protein; this gene contains a frame shift which is not the result of sequencing error		1.18	4.15E-01	1.08	5.04E-01	1.06	2.29E-01
MSMEG_4135	possible lysine decarboxylase superfamily protein		1.21	2.04E-01	1.03	4.12E-01	1.18	1.99E-01
MSMEG_4136	dTDP-glucose 4,6-dehydratase		1.03	7.57E-01	1.01	8.62E-01	2.06	4.30E-01
MSMEG_4137	hypothetical protein		1.19	3.01E-01	0.99	9.65E-01	1.05	7.29E-01
MSMEG_4138	aliphatic nitrilase		1.47	5.07E-02	1.46	2.43E-03	1.04	3.88E-01
MSMEG_4139	major facilitator superfamily MFS_1; this gene contains a frame shift which is not the result of sequencing error		1.47	4.66E-01	1.47	1.50E-01	1.34	3.22E-01
MSMEG_4140	GntR-family protein transcriptional regulator		0.77	3.91E-02	1.32	3.27E-01	1.19	2.16E-01

MSMEG_4141	conserved hypothetical protein	2.71	3.58E-02	1.12	6.86E-01	0.72	1.20E-01
MSMEG_4142	transcriptional repressor, TetR family protein, putative	0.55	1.23E-04	0.97	5.30E-01	1.19	3.47E-01
MSMEG_4143	conserved hypothetical protein	0.93	5.54E-01	1.55	1.68E-01	0.72	3.47E-01
MSMEG_4144	transcriptional regulator, LysR family protein	0.93	4.49E-01	1.61	5.46E-02	0.50	1.05E-05
MSMEG_4145	cupin 2 protein	0.76	3.62E-02	0.66	7.59E-03	1.11	5.84E-01
MSMEG_4146	quinone oxidoreductase	1.13	4.09E-01	1.21	2.71E-01	1.05	1.40E-01
MSMEG_4147	major facilitator superfamily protein MFS_1, putative	0.89	3.85E-02	1.29	4.04E-01	1.33	2.39E-01
MSMEG_4148	lipase	4.49	1.12E-01	0.62	4.86E-02	1.16	3.55E-01
MSMEG_4149	acetaldehyde dehydrogenase	1.94	3.93E-02	1.00	9.86E-01	1.01	9.51E-01
MSMEG_4150	4-hydroxy-2-oxovalerate aldolase	2.58	1.54E-02	0.90	2.79E-01	1.07	1.08E-01
MSMEG_4151	conserved domain protein	1.43	2.16E-01	1.00	9.96E-01	1.09	5.66E-01
MSMEG_4152	phenoxybenzoate dioxygenase beta subunit	1.07	3.07E-01	1.23	2.59E-01	1.35	9.35E-02
MSMEG_4153	cytochrome P450	1.16	3.31E-02	1.12	2.36E-01	1.16	7.00E-02
MSMEG_4154	transposase, Mutator family protein	1.36	4.10E-01	1.11	9.69E-02	1.03	1.86E-01
MSMEG_4155	transposase A	0.83	7.89E-02	1.57	2.68E-01	1.30	2.98E-01
MSMEG_4156	putative transposase	1.18	5.30E-01	1.25	1.07E-01	0.98	1.51E-01
MSMEG_4157	putative transposase	0.83	4.31E-02	1.47	1.22E-01	1.00	9.03E-01
MSMEG_4158	putative transposase	0.91	1.47E-01	1.19	1.54E-01	0.98	8.19E-01
MSMEG_4160	hypothetical protein	1.23	3.25E-01	1.04	6.40E-01	0.95	7.14E-01
MSMEG_4161	hypothetical protein	1.45	1.00E-01	0.89	2.88E-01	1.07	2.93E-01
MSMEG_4162	conserved hypothetical protein	0.89	4.20E-01	0.89	1.52E-01	1.02	8.78E-01
MSMEG_4163	3-oxoacyl-[acyl-carrier-protein] reductase	1.17	4.49E-01	1.13	9.47E-02	1.35	1.58E-01
MSMEG_4164	3-hydroxybutyryl-CoA dehydratase	0.96	5.94E-01	0.97	7.73E-01	0.80	5.51E-01
MSMEG_4165	[NAD+] benzaldehyde dehydrogenase	1.05	6.16E-01	1.02	8.95E-01	1.44	4.60E-01
MSMEG_4166	acyl-CoA dehydrogenase	1.36	5.08E-02	0.96	6.61E-01	0.65	2.88E-02
MSMEG_4167	alcohol dehydrogenase B	1.13	1.72E-01	1.09	7.88E-02	0.80	8.71E-02
MSMEG_4168	propionate CoA-transferase	1.09	3.65E-01	1.06	4.55E-01	0.77	1.93E-01
MSMEG_4169	3-oxoacyl-[acyl-carrier-protein] reductase	1.32	3.97E-01	1.20	4.57E-02	1.08	3.26E-01
MSMEG_4170	ribose transport ATP-binding protein RbsA	1.63	1.19E-01	2.33	3.07E-01	0.83	1.65E-01
MSMEG_4171	ribose transport system permease protein RbsC	1.14	2.94E-01	1.03	8.10E-01	0.79	2.37E-01
MSMEG_4172	D-ribose-binding periplasmic protein RbsB	2.07	4.46E-02	1.03	4.75E-01	0.63	8.88E-02
MSMEG_4173	MaoC family protein	1.26	1.28E-01	1.22	3.65E-01	0.68	1.75E-01
MSMEG_4174	transcriptional regulator, IclR family protein	2.09	5.47E-02	0.81	1.46E-02	0.79	1.75E-01
MSMEG_4175	transcriptional regulator, ArsR family protein	0.95	5.88E-01	0.95	2.28E-01	0.92	2.57E-01
MSMEG_4176	conserved hypothetical protein	1.26	5.15E-03	1.18	3.26E-01	0.78	1.55E-01

MSMEG_4177	mercuric reductase; this gene contains a frame shift which is not the result of sequencing error; identified by match to protein family HMM PF00070; match to protein family HMM PF02852; match to protein family HMM PF07992		1.28	3.95E-03	1.21	1.76E-01	0.66	1.16E-02
MSMEG_4178	flavodoxin/nitric oxide synthase		1.58	2.22E-03	0.80	1.33E-02	0.51	1.15E-03
MSMEG_4179	probable conserved integral membrane protein		1.12	3.96E-01	1.07	5.90E-01	0.65	8.18E-03
MSMEG_4180	ATP phosphoribosyltransferase	*hisG*	0.85	2.57E-01	1.11	8.64E-02	1.60	9.87E-03
MSMEG_4181	phosphoribosyl-ATP pyrophosphohydrolase		0.57	1.33E-04	1.07	1.37E-02	2.24	2.33E-02
MSMEG_4182	arabinose-proton symporter		1.17	4.16E-01	1.29	2.41E-02	0.94	2.96E-01
MSMEG_4183	phosphoglycolate phosphatase, chromosomal		0.58	9.00E-05	0.82	2.19E-02	3.65	6.16E-03
MSMEG_4184	dtdp-glucose-46-dehydratase		1.69	4.94E-02	0.88	6.56E-01	0.81	4.54E-01
MSMEG_4185	methionine synthase	*metH*	0.65	1.30E-03	2.07	2.40E-01	3.11	1.79E-04
MSMEG_4186	conserved hypothetical protein		1.43	4.89E-03	0.97	6.82E-01	0.86	1.85E-01
MSMEG_4187	conserved hypothetical protein		1.74	3.14E-04	0.76	4.27E-03	0.76	7.14E-02
MSMEG_4188	short chain dehydrogenase		2.15	6.03E-03	0.81	5.97E-03	0.69	6.16E-02
MSMEG_4189	cysteinyl-tRNA synthetase	*cysS*	1.10	3.04E-01	1.07	6.57E-01	1.24	3.69E-02
MSMEG_4190	inositol monophosphatase family protein		0.96	7.87E-01	1.04	3.43E-01	2.40	1.12E-03
MSMEG_4191	conserved hypothetical protein		0.81	2.91E-02	0.88	2.31E-01	1.84	1.70E-02
MSMEG_4192	conserved hypothetical protein		1.32	3.07E-02	0.65	7.65E-03	1.18	3.92E-02
MSMEG_4193	phosphoglycerate mutase family protein		0.66	4.59E-04	1.03	7.28E-01	1.39	7.90E-02
MSMEG_4194	undecaprenol kinase, putative		0.65	2.68E-04	1.09	5.32E-01	2.02	6.72E-02
MSMEG_4195	conserved hypothetical protein		2.21	1.84E-03	1.31	1.38E-01	0.73	5.60E-02
MSMEG_4196	LppL protein		1.04	1.71E-02	0.99	9.48E-01	1.62	6.80E-02
MSMEG_4197	conserved hypothetical protein		1.46	4.47E-03	0.89	2.69E-01	1.48	1.13E-02
MSMEG_4198	dihydroorotate oxidase	*pyrD*	0.56	1.30E-04	1.07	4.47E-01	1.22	4.70E-02
MSMEG_4199	conserved hypothetical protein		1.00	9.12E-01	0.74	6.07E-02	0.65	7.07E-03
MSMEG_4200	peptidase M20		0.87	6.34E-02	0.81	3.35E-02	1.28	5.68E-02
MSMEG_4202	conserved hypothetical protein		1.35	1.01E-01	1.21	5.98E-02	0.59	5.22E-02
MSMEG_4203	peptidase S8 and S53, subtilisin, kexin, sedolisin		1.40	2.29E-01	0.98	7.92E-01	0.64	1.86E-01
MSMEG_4204	catalase		8.69	2.53E-02	0.35	1.88E-02	0.67	1.49E-01
MSMEG_4205	putative transcriptional regulator		9.37	2.19E-02	0.49	3.42E-02	0.70	1.65E-01
MSMEG_4206	Molybdopterin oxidoreductase; this gene contains a frame shift which is not the result of sequencing error; identified by match to protein family HMM PF00384		4.16	1.14E-02	1.12	5.20E-01	0.54	3.60E-02

MSMEG_4206	Molybdopterin oxidoreductase; this gene contains a frame shift which is not the result of sequencing error; identified by match to protein family HMM PF00384		2.26	7.51E-02	0.89	5.56E-01	0.98	5.47E-01
MSMEG_4207	universal stress protein family protein		21.74	9.84E-03	0.95	7.49E-01	0.04	1.60E-07
MSMEG_4208	integral membrane protein		15.01	6.84E-03	1.18	4.03E-01	0.06	2.62E-07
MSMEG_4209	integral membrane protein		20.51	1.51E-02	0.75	1.12E-01	0.06	2.26E-07
MSMEG_4210	secreted protein		23.34	4.37E-02	1.19	1.94E-01	0.02	3.64E-09
MSMEG_4211	two component system histidine kinase		6.89	2.43E-03	0.96	6.82E-01	0.08	1.75E-06
MSMEG_4212	transcriptional regulatory protein CitT		3.56	1.10E-02	0.65	2.27E-02	0.71	1.85E-01
MSMEG_4213	cytochrome P450 hydroxylase		3.24	2.52E-03	0.49	6.42E-03	1.14	6.98E-01
MSMEG_4214	glutamine amidotransferase		0.61	5.17E-05	0.96	2.36E-01	3.30	9.90E-03
MSMEG_4215	phosphoribosyltransferase		2.83	3.88E-03	0.91	3.30E-01	2.03	4.57E-03
MSMEG_4216	conserved hypothetical protein		0.39	6.95E-07	1.05	1.18E-02	0.50	1.27E-03
MSMEG_4217	DivIVA protein		0.74	1.10E-02	1.15	2.37E-01	0.35	1.28E-03
MSMEG_4218	possible conserved transmembrane protein		0.48	1.98E-04	1.08	4.92E-01	1.40	3.82E-01
MSMEG_4219	conserved hypothetical protein		0.74	1.20E-01	1.10	3.96E-01	0.86	4.87E-01
MSMEG_4220	conserved hypothetical protein		0.71	4.28E-02	0.89	4.00E-01	1.90	7.59E-02
MSMEG_4221	conserved hypothetical protein		0.96	7.69E-01	0.81	5.91E-03	1.44	1.28E-01
MSMEG_4222	cell division protein FtsZ	*ftsZ*	1.09	2.80E-01	0.76	5.13E-02	0.99	9.37E-01
MSMEG_4223	hypothetical protein		0.70	9.28E-02	0.81	3.30E-01	1.20	4.03E-01
MSMEG_4224	hypothetical protein		0.86	3.40E-01	0.92	7.70E-01	1.05	7.26E-01
MSMEG_4225	putative Cell division protein FtsQ		0.61	1.86E-02	1.07	6.36E-01	1.65	3.42E-01
MSMEG_4226	UDP-N-acetylmuramate--alanine ligase	*murC*	0.48	1.94E-03	0.93	1.66E-01	4.37	7.55E-02
MSMEG_4227	undecaprenyldiphospho-muramoylpentapeptide beta-N-acetylglucosaminyltransferase	*murG*	0.54	5.10E-03	0.90	4.30E-01	5.45	6.37E-02
MSMEG_4228	cell division protein FtsW	*ftsW*	0.85	4.25E-01	1.10	7.52E-01	2.73	1.04E-01
MSMEG_4229	UDP-N-acetylmuramoylalanine--D-glutamate ligase	*murD*	0.71	1.46E-01	0.87	5.33E-01	2.72	1.28E-01
MSMEG_4230	phospho-N-acetylmuramoyl-pentapeptide-transferase	*mraY*	0.43	8.12E-04	0.83	1.05E-01	2.99	3.28E-02
MSMEG_4231	UDP-N-acetylmuramoyl-tripeptide--D-alanyl-D-alanine ligase		0.47	6.68E-04	0.81	1.87E-02	2.27	3.03E-03
MSMEG_4232	UDP-N-acetylmuramoylalanyl-D-glutamate-2, 6-diaminopimelate ligase		0.53	5.56E-04	0.77	1.56E-02	4.90	9.83E-02
MSMEG_4233	Penicillin binding protein transpeptidase domain protein		0.53	2.03E-02	0.76	1.59E-01	2.31	1.23E-01
MSMEG_4234	conserved hypothetical protein		0.75	2.27E-01	1.03	9.31E-01	1.67	2.83E-01

MSMEG_4235	S-adenosyl-methyltransferase MraW	*mraW*	0.76	2.20E-01	0.91	4.65E-01	1.68	3.02E-01
MSMEG_4236	MraZ protein	*mraZ*	0.82	2.28E-01	0.93	8.00E-01	2.15	1.87E-01
MSMEG_4237	probable conserved transmembrane protein		2.52	2.86E-04	1.02	8.85E-01	1.08	5.46E-01
MSMEG_4238	conserved hypothetical protein		0.79	1.57E-02	0.86	5.43E-02	1.06	6.82E-01
MSMEG_4239	LppM protein		0.90	6.57E-01	1.05	7.11E-01	1.23	2.63E-01
MSMEG_4240	polyprenyl synthetase		0.67	4.72E-04	0.92	3.96E-01	1.22	4.41E-01
MSMEG_4241	possible conserved integral membrane protein		0.64	1.82E-05	0.85	2.19E-01	1.73	1.19E-01
MSMEG_4242	transcriptional regulatory protein		1.55	4.39E-03	0.55	1.42E-02	1.53	1.39E-01
MSMEG_4243	serine/threonine-protein kinase PK-1		1.11	1.99E-01	0.88	5.03E-01	2.52	1.20E-03
MSMEG_4244	3-deoxy-7-phosphoheptulonate synthase		0.76	3.18E-04	1.03	4.25E-01	0.93	4.46E-01
MSMEG_4245	conserved hypothetical protein		0.58	2.11E-02	0.88	3.05E-01	1.11	1.04E-01
MSMEG_4246	probable conserved integral membrane protein		1.34	4.91E-01	0.91	4.51E-01	1.71	2.91E-02
MSMEG_4247	probable conserved integral membrane protein		1.11	3.86E-01	1.04	6.78E-01	1.72	3.04E-02
MSMEG_4248	1-acylglycerol-3-phosphate O-acyltransferase		1.18	5.99E-02	0.91	1.49E-01	0.90	6.17E-01
MSMEG_4249	conserved hypothetical protein		0.76	2.55E-03	0.87	1.24E-01	2.35	1.50E-03
MSMEG_4250	putative membrane transport ATPase		0.76	6.01E-05	0.68	1.35E-03	4.09	4.69E-02
MSMEG_4251	cyclase/dehydrase		0.82	1.88E-02	0.87	2.31E-01	2.23	4.17E-02
MSMEG_4252	conserved hypothetical protein		1.06	3.31E-01	0.76	5.18E-04	0.87	1.35E-01
MSMEG_4253	glycosyl transferase, group 1 family protein		0.94	6.11E-01	0.78	6.04E-02	2.62	1.16E-02
MSMEG_4254	AMP-binding enzyme		0.71	3.31E-04	0.87	1.66E-01	0.84	6.04E-02
MSMEG_4255	conserved hypothetical protein		0.55	9.05E-03	1.23	2.34E-01	1.42	4.23E-01
MSMEG_4256	NLP/P60 family protein		0.82	1.95E-02	0.87	9.15E-02	0.93	5.91E-01
MSMEG_4257	conserved hypothetical protein		1.31	4.21E-01	0.81	1.78E-01	1.63	1.67E-01
MSMEG_4258	anthranilate phosphoribosyltransferase	*trpD*	0.47	9.40E-05	0.98	7.39E-01	1.72	6.30E-02
MSMEG_4259	DNA polymerase III, epsilon subunit		0.94	3.99E-01	0.97	7.81E-01	1.29	1.17E-01
MSMEG_4260	cytochrome c oxidase subunit 3		0.40	4.20E-07	1.18	1.42E-02	5.74	3.22E-03
MSMEG_4261	ubiquinol-cytochrome c reductase cytochrome c subunit		0.42	5.04E-06	0.84	1.13E-03	2.79	8.29E-03
MSMEG_4262	ubiquinol-cytochrome c reductase iron-sulfur subunit		0.35	1.05E-05	0.94	3.14E-01	5.98	5.86E-04
MSMEG_4263	ubiquinol-cytochrome c reductase cytochrome b subunit		0.38	1.45E-05	0.94	4.75E-01	6.13	2.63E-02
MSMEG_4264	conserved hypothetical protein		0.53	4.11E-04	0.91	1.49E-01	1.55	1.59E-02
MSMEG_4265	MmpS3 protein		0.99	8.53E-01	0.93	5.30E-01	1.41	7.78E-02
MSMEG_4266	hypothetical protein		0.81	5.51E-02	0.71	8.05E-02	4.52	3.04E-01
MSMEG_4267	probable cytochrome c oxidase polypeptide 4		0.34	9.95E-06	0.92	2.21E-01	2.55	1.81E-02
MSMEG_4268	cytochrome c oxidase subunit 2		0.32	2.96E-06	0.93	2.92E-01	2.66	1.44E-02
MSMEG_4269	asparagine synthase (glutamine-hydrolyzing)	*asnB*	0.30	2.54E-04	0.92	6.47E-01	1.44	2.07E-01

MSMEG_4270	adenosine kinase		0.63	3.54E-04	1.18	2.02E-02	1.28	2.42E-01
MSMEG_4271	conserved hypothetical protein		1.21	4.03E-03	0.67	3.63E-03	0.64	8.39E-02
MSMEG_4272	HesB/YadR/YfhF family protein		3.19	3.56E-04	0.67	1.94E-03	0.51	1.10E-03
MSMEG_4273	integral membrane protein		0.76	2.32E-03	0.86	8.14E-04	1.20	2.07E-01
MSMEG_4274	Cobinamide kinase	*cobU*	0.31	2.13E-05	1.32	7.13E-02	1.78	2.81E-02
MSMEG_4275	nicotinate-nucleotide--dimethylbenzimidazole phosphoribosyltransferase		0.48	2.04E-03	0.94	5.85E-01	1.77	5.33E-03
MSMEG_4276	branched-chain amino acid aminotransferase	*ilvE*	1.83	1.85E-03	1.33	8.08E-02	1.12	1.47E-01
MSMEG_4277	cobalamin 5'-phosphate synthase	*cobS*	0.68	4.02E-02	1.09	5.53E-01	1.19	3.90E-01
MSMEG_4278	glycine cleavage system T protein	*gcvT*	0.88	1.65E-01	0.88	1.17E-01	0.42	2.05E-05
MSMEG_4279	adenylate and Guanylate cyclase catalytic domain protein		2.14	2.11E-03	1.04	5.62E-01	1.76	4.29E-01
MSMEG_4280	short chain dehydrogenase		1.93	7.60E-03	0.65	3.49E-03	1.72	1.73E-01
MSMEG_4281	cytosol aminopeptidase		0.49	6.55E-05	0.80	2.54E-04	0.74	4.81E-02
MSMEG_4282	conserved hypothetical protein		1.37	2.05E-03	0.66	3.27E-03	1.06	1.07E-01
MSMEG_4283	2-oxoglutarate dehydrogenase, E2 component, dihydrolipoamide succinyltransferase	*sucB*	0.76	1.42E-02	0.95	4.03E-01	1.37	1.01E-01
MSMEG_4284	conserved hypothetical protein		0.51	1.77E-04	0.92	2.37E-01	2.98	1.36E-03
MSMEG_4285	lipoyltransferase	*lipB*	1.00	9.46E-01	0.90	2.40E-01	1.95	1.39E-03
MSMEG_4286	lipoic acid synthetase	*lipA*	1.22	7.85E-04	0.90	1.71E-01	1.52	4.12E-03
MSMEG_4287	integral membrane protein		1.14	6.97E-03	0.94	3.87E-01	1.83	2.58E-03
MSMEG_4288	integral membrane protein		1.23	7.54E-02	0.73	1.30E-02	1.30	1.05E-02
MSMEG_4289	amidohydrolase		0.68	3.67E-02	1.01	9.51E-01	1.61	1.36E-01
MSMEG_4290	glutamine synthetase, type I	*glnA*	0.59	2.97E-02	1.15	5.65E-01	0.42	7.59E-04
MSMEG_4291	D-tyrosyl-tRNA(Tyr) deacylase	*dtd*	0.43	3.28E-03	0.88	3.26E-01	1.26	3.44E-01
MSMEG_4292	integral membrane protein		0.55	1.15E-03	0.99	9.56E-01	1.03	8.87E-01
MSMEG_4293	glutamate-ammonia-ligase adenylyltransferase		0.47	1.04E-03	0.95	4.17E-01	1.83	4.13E-02
MSMEG_4294	glutamine synthetase, type I	*glnA*	0.71	1.07E-01	0.81	3.63E-02	0.54	2.06E-05
MSMEG_4295	hydrolase, alpha/beta fold family protein		0.50	3.90E-03	1.05	7.45E-01	0.88	4.88E-01
MSMEG_4296	protease		0.50	2.36E-04	0.89	1.78E-01	1.60	1.73E-01
MSMEG_4297	acyltransferase, ws/dgat/mgat subfamily protein		1.39	2.15E-01	0.82	1.15E-01	0.47	2.35E-02
MSMEG_4298	3-methyl-2-oxobutanoate hydroxymethyltransferase	*panB*	0.08	9.05E-07	2.69	1.94E-01	8.79	6.40E-02
MSMEG_4299	enoyl-CoA hydratase/isomerase		0.70	1.88E-02	1.08	5.72E-01	1.18	1.04E-01
MSMEG_4300	transcription regulator AmtR		0.59	8.34E-04	1.06	6.86E-01	2.87	5.53E-02
MSMEG_4301	acyl-CoA synthase		1.04	4.40E-01	0.65	1.98E-03	3.32	7.43E-04
MSMEG_4302	adenylate cyclase, putative		0.93	6.11E-01	0.88	4.77E-01	1.40	1.04E-01
MSMEG_4303	methyltransferase		1.51	8.43E-02	0.96	7.03E-01	0.92	3.27E-01

MSMEG_4304	regulatory protein		0.71	1.43E-02	1.45	9.51E-02	0.69	9.00E-02
MSMEG_4305	phosphoglycerate mutase		0.71	3.39E-03	0.97	5.13E-01	1.06	7.26E-01
MSMEG_4306	conserved hypothetical protein		0.56	1.07E-03	0.84	7.36E-03	1.50	1.26E-01
MSMEG_4307	conserved hypothetical protein		0.63	3.22E-04	0.82	1.30E-01	1.14	5.14E-01
MSMEG_4308	5'-nucleotidase		0.99	9.55E-01	0.83	1.40E-01	2.29	4.82E-03
MSMEG_4309	Low molecular weight protein-tyrosine-phosphatase		1.08	5.08E-01	0.66	3.73E-02	2.26	1.43E-02
MSMEG_4310	cobalamin biosynthesis protein CobD	*cobD*	0.53	2.65E-03	1.10	3.82E-01	1.34	3.63E-01
MSMEG_4311	conserved hypothetical protein		0.99	9.07E-01	0.82	1.15E-01	2.72	1.02E-02
MSMEG_4312	conserved hypothetical protein; this gene contains a frame shift which is not the result of sequencing error		0.50	3.53E-04	0.81	3.92E-02	1.67	6.62E-02
MSMEG_4313	glyoxalase/bleomycin resistance protein/dioxygenase		0.64	1.59E-04	0.80	7.28E-02	1.41	4.74E-02
MSMEG_4314	hypothetical protein		0.64	4.63E-04	0.83	1.56E-02	1.05	8.02E-01
MSMEG_4315	RNA polymerase sigma factor, sigma-70 family protein		1.25	1.10E-01	0.87	2.15E-01	1.02	8.27E-01
MSMEG_4316	methylated-DNA--protein-cysteine methyltransferase		1.58	2.73E-02	0.87	4.42E-01	1.24	2.76E-01
MSMEG_4317	conserved hypothetical protein		1.53	9.33E-02	0.85	2.38E-01	1.24	1.30E-01
MSMEG_4318	hypothetical protein		0.94	5.75E-01	0.85	3.47E-02	1.02	8.10E-01
MSMEG_4320	alkyl hydroperoxide reductase/ Thiol specific antioxidant/ Mal allergen		0.42	3.31E-04	0.92	3.77E-01	1.29	2.93E-01
MSMEG_4321	conserved hypothetical protein		1.06	6.03E-01	0.99	9.40E-01	0.80	1.72E-01
MSMEG_4322	conserved hypothetical protein		0.70	1.77E-04	0.92	4.91E-01	1.40	4.67E-01
MSMEG_4323	pyruvate dehydrogenase E1 component		0.69	5.82E-04	1.01	6.72E-01	0.88	9.23E-04
MSMEG_4324	conserved hypothetical protein		1.57	3.50E-02	0.70	1.11E-02	1.02	8.67E-01
MSMEG_4325	malonyl CoA-acyl carrier protein transacylase		0.80	2.80E-02	1.03	6.38E-01	2.56	1.03E-02
MSMEG_4326	acyl carrier protein	*acpP*	0.43	6.97E-05	0.80	3.18E-02	3.80	1.52E-02
MSMEG_4327	3-oxoacyl-[acyl-carrier-protein] synthase 1		0.64	1.15E-02	0.94	3.59E-01	2.96	8.10E-02
MSMEG_4328	3-oxoacyl-[acyl-carrier-protein] synthase 2		0.48	1.46E-04	1.28	1.75E-01	3.61	3.39E-04
MSMEG_4329	propionyl-CoA carboxylase beta chain		0.75	1.58E-02	0.98	7.49E-01	2.44	2.80E-02
MSMEG_4330	short chain dehydrogenase		3.93	1.88E-04	0.89	4.75E-01	0.46	8.23E-04
MSMEG_4331	putative transcriptional regulator		1.15	2.47E-01	1.00	9.79E-01	1.36	3.10E-01
MSMEG_4332	glycerol-3-phosphate dehydrogenase 1		0.94	7.23E-01	1.11	1.23E-01	1.43	2.14E-01
MSMEG_4333	transcriptional regulator, TetR family protein		1.48	5.04E-02	1.17	5.04E-01	0.98	4.98E-01
MSMEG_4334	flavoprotein		1.17	2.62E-01	1.52	5.27E-01	1.20	5.29E-01
MSMEG_4335	conserved hypothetical protein		1.10	5.38E-01	1.20	1.57E-01	0.98	5.50E-01

MSMEG_4336	conserved hypothetical protein		1.08	7.37E-01	0.98	9.58E-01	1.66	3.09E-01
MSMEG_4337	beta-lactamase		0.55	1.41E-04	0.92	2.71E-01	1.11	5.64E-01
MSMEG_4338	possible transcriptional regulatory protein		1.59	1.15E-04	0.98	6.67E-01	1.18	1.98E-01
MSMEG_4339	tetratricopeptide repeat family protein		1.92	1.42E-01	0.97	6.80E-01	1.00	9.68E-01
MSMEG_4340	NAD/mycothiol-dependent formaldehyde dehydrogenase		0.93	2.21E-01	0.83	3.29E-02	0.57	1.58E-05
MSMEG_4341	hypothetical protein		1.09	3.16E-01	1.13	1.10E-01	3.44	3.65E-01
MSMEG_4342	metallo-beta-lactamase family protein		0.65	3.46E-03	0.92	4.55E-01	0.71	1.46E-02
MSMEG_4343	hypothetical protein		1.07	3.87E-01	1.00	9.95E-01	0.84	1.57E-01
MSMEG_4344	putative monooxygenase		0.43	8.96E-03	1.82	1.77E-01	0.90	4.69E-01
MSMEG_4345	conserved hypothetical protein		0.83	2.38E-01	1.19	3.67E-01	1.15	3.80E-01
MSMEG_4346	conserved hypothetical protein		1.09	2.60E-01	0.88	4.28E-01	1.24	8.55E-02
MSMEG_4347	conserved hypothetical protein		0.96	6.70E-01	0.93	2.37E-01	1.03	4.96E-01
MSMEG_4348	acetolactate synthase large subunit		1.88	2.21E-02	0.68	5.01E-03	0.93	7.27E-01
MSMEG_4349	conserved hypothetical proline rich protein		1.10	3.19E-01	0.90	1.18E-01	0.89	2.05E-01
MSMEG_4350	dihydrodipicolinate reductase	*dapB*	1.58	3.64E-02	0.89	5.15E-01	0.76	1.56E-01
MSMEG_4351	hypothetical oxidoreductase YjgI		3.62	1.15E-02	0.55	2.30E-02	0.90	2.94E-01
MSMEG_4352	putative secreted protein		1.05	6.67E-01	1.57	1.40E-02	0.42	1.09E-02
MSMEG_4353	conserved hypothetical protein		0.96	5.74E-01	0.90	4.02E-02	0.45	6.52E-06
MSMEG_4354	dipeptide-binding protein of ABC transport system		1.03	7.21E-01	1.25	1.45E-01	0.59	2.89E-02
MSMEG_4355	peptide ABC transporter, permease protein		0.82	1.75E-01	1.19	1.91E-01	1.10	7.41E-01
MSMEG_4356	inner membrane ABC transporter permease protein YddQ		0.97	7.37E-01	1.23	9.59E-02	1.07	3.63E-01
MSMEG_4357	ABC transporter, ATP-binding protein		0.72	1.95E-02	1.53	6.49E-02	0.94	6.21E-01
MSMEG_4358	D-beta-hydroxybutyrate dehydrogenase		0.57	1.59E-05	1.03	5.66E-01	1.50	4.53E-02
MSMEG_4359	hypothetical protein		1.05	7.40E-01	1.08	5.88E-01	1.01	7.95E-01
MSMEG_4360	conserved hypothetical protein		1.29	2.60E-01	0.67	8.13E-02	1.00	9.21E-01
MSMEG_4361	conserved hypothetical protein		4.84	1.25E-03	1.03	8.51E-01	0.89	3.09E-01
MSMEG_4362	universal stress protein family protein		1.37	1.68E-02	0.83	8.69E-02	1.09	3.69E-01
MSMEG_4363	conserved hypothetical protein		3.22	3.40E-03	0.73	3.81E-02	1.20	2.24E-01
MSMEG_4364	hypothetical protein		1.19	5.21E-01	1.00	9.58E-01	2.07	2.49E-01
MSMEG_4365	conserved hypothetical protein		0.77	4.91E-02	1.42	1.05E-01	0.96	6.62E-01
MSMEG_4366	serine/threonine-protein kinase PknD		0.93	3.60E-01	0.91	3.24E-02	1.20	1.64E-01
MSMEG_4367	formamidase		1.61	3.89E-02	0.88	7.10E-01	0.25	4.30E-05
MSMEG_4368	putative regulatory protein, FmdB family protein		1.92	3.01E-03	0.88	6.01E-01	0.21	9.59E-06
MSMEG_4369	hypothetical protein		0.94	3.48E-01	0.94	1.33E-01	1.11	2.71E-01
MSMEG_4370	regulatory protein		0.89	2.51E-01	0.95	4.41E-01	1.81	4.26E-02

MSMEG_4371	vanillate O-demethylase oxidoreductase		1.09	6.85E-01	1.02	9.29E-01	0.98	5.76E-01
MSMEG_4372	capreomycidine hydroxylase		1.20	2.46E-01	1.04	6.79E-01	1.25	3.16E-01
MSMEG_4373	polysaccharide deacetylase, putative		1.15	6.30E-01	0.98	8.84E-01	0.89	5.25E-01
MSMEG_4374	agmatinase	*speB*	1.63	3.18E-01	0.94	1.23E-01	1.13	4.45E-01
MSMEG_4375	3-oxoacyl-[acyl carrier protein] reductase		1.05	6.13E-01	1.10	3.95E-01	1.28	3.10E-01
MSMEG_4376	3-oxoacyl-[acyl-carrier-protein] reductase		1.08	2.07E-01	1.09	2.61E-01	0.90	1.06E-01
MSMEG_4377	amino acid permease family protein, putative		1.12	3.40E-01	1.09	4.12E-01	1.04	7.05E-01
MSMEG_4378	two-component system response regulator		1.16	1.61E-01	0.93	5.37E-01	1.13	5.12E-01
MSMEG_4379	isochorismatase hydrolase		2.16	4.45E-02	0.73	9.88E-03	1.17	6.72E-01
MSMEG_4380	ABC transporter, permease/ATP-binding protein		1.35	3.01E-01	0.95	4.84E-01	1.28	4.19E-01
MSMEG_4381	amidase		0.75	1.75E-02	1.25	3.22E-01	0.55	9.08E-04
MSMEG_4382	dehydrogenase/reductase SDR family protein member 10		0.95	6.56E-01	1.18	4.17E-01	1.13	8.68E-01
MSMEG_4383	MmpL5 protein		0.60	1.31E-03	0.89	2.62E-01	1.20	3.48E-01
MSMEG_4384	Penicillin binding protein transpeptidase domain protein		3.49	6.50E-04	0.88	9.11E-02	1.20	1.60E-01
MSMEG_4385	ABC transporter oligopeptide binding protein		1.05	3.06E-01	2.57	8.26E-02	1.36	1.31E-01
MSMEG_4386	ABC transporter permease protein		1.20	1.59E-01	1.32	2.52E-01	1.19	4.34E-01
MSMEG_4387	ABC transporter permease protein		0.98	8.48E-01	1.25	5.50E-01	0.78	3.65E-01
MSMEG_4388	ABC transporter ATP-binding protein		1.35	3.50E-01	1.51	2.18E-01	1.05	3.02E-01
MSMEG_4389	monooxygenase, NtaA/SnaA/SoxA family		0.92	4.82E-01	1.77	1.67E-01	0.82	1.19E-01
MSMEG_4390	hypothetical protein		1.01	9.25E-01	1.38	1.27E-01	1.03	4.86E-01
MSMEG_4391	acyl-CoA dehydrogenase-family protein		1.18	2.60E-01	3.36	3.08E-01	1.03	6.08E-01
MSMEG_4392	organophopsphate acid anhydrase		1.91	4.57E-02	0.77	3.23E-02	1.88	9.94E-02
MSMEG_4393	carboxyvinyl-carboxyphosphonate phosphorylmutase		1.44	8.51E-02	0.85	2.88E-01	1.13	4.17E-01
MSMEG_4394	LysR family protein transcriptional regulatory protein		0.75	4.40E-02	0.96	4.85E-01	1.70	1.41E-01
MSMEG_4395	excalibur domain family protein		1.13	3.66E-01	0.99	8.91E-01	1.04	6.73E-01
MSMEG_4396	isochorismatase hydrolase		1.03	8.28E-01	1.97	1.35E-01	4.95	3.50E-02
MSMEG_4397	transcriptional regulator, MerR family protein		3.28	8.17E-02	1.02	9.05E-01	0.85	2.07E-01
MSMEG_4398	conserved hypothetical protein		1.10	6.65E-01	0.90	2.42E-01	5.04	1.74E-01
MSMEG_4399	conserved hypothetical protein		0.48	1.36E-03	1.00	9.82E-01	1.96	1.88E-01
MSMEG_4400	alcohol dehydrogenase, zinc-containing		0.75	4.10E-04	0.80	4.03E-03	1.82	2.27E-03
MSMEG_4401	phosphonoacetaldehyde hydrolase		0.84	6.62E-03	0.96	1.96E-01	1.58	4.96E-02
MSMEG_4404	hypothetical protein		0.88	5.00E-01	0.88	1.58E-01	1.97	6.04E-02
MSMEG_4405	putative ECF sigma factor RpoE1		1.72	2.77E-02	1.21	2.23E-01	1.93	3.34E-01
MSMEG_4406	UsfY protein		1.11	2.48E-01	1.25	3.67E-01	1.03	5.89E-01

MSMEG_4407	conserved hypothetical protein		1.31	2.79E-01	1.07	6.28E-01	1.05	3.96E-01
MSMEG_4408	integral membrane protein		1.16	2.47E-01	0.88	1.40E-01	0.88	3.58E-01
MSMEG_4409	hypothetical protein		1.19	3.21E-01	0.93	1.22E-01	1.16	2.25E-01
MSMEG_4410	hypothetical protein		1.03	6.50E-01	0.95	6.71E-01	1.76	4.86E-01
MSMEG_4411	oxidoreductase FAD-binding region		4.44	6.10E-02	1.40	1.01E-01	0.33	6.02E-03
MSMEG_4412	oxidoreductase, molybdopterin binding		0.96	5.72E-01	0.93	5.42E-01	1.16	3.32E-01
MSMEG_4413	hypothetical protein		0.76	3.68E-02	1.20	3.35E-01	1.12	3.64E-01
MSMEG_4414	conserved hypothetical protein		1.04	8.07E-01	1.26	3.68E-01	1.19	4.82E-01
MSMEG_4415	transaldolase	*tal*	1.06	3.48E-01	0.94	5.67E-01	1.01	7.64E-01
MSMEG_4416	hypothetical protein		2.25	7.80E-02	1.05	8.32E-01	2.46	3.36E-01
MSMEG_4417	methionine-S-sulfoxide reductase	*msrA*	1.87	6.95E-02	0.93	5.20E-01	1.01	8.59E-01
MSMEG_4418	methionine-R-sulfoxide reductase	*msrB*	1.05	7.91E-02	1.04	2.70E-01	0.94	2.82E-01
MSMEG_4419	glucose-1-dehydrogenase		1.06	4.68E-01	1.17	9.22E-02	1.48	3.07E-01
MSMEG_4420	cupin 2, conserved barrel		1.17	2.51E-01	1.40	2.66E-01	0.80	2.59E-01
MSMEG_4421	conserved hypothetical protein		1.36	1.80E-01	1.42	1.68E-01	1.24	8.94E-02
MSMEG_4422	oxidoreductase		0.81	2.54E-01	1.04	7.74E-01	1.10	5.38E-01
MSMEG_4423	oxidoreductase		1.54	1.96E-01	1.14	3.12E-01	0.90	4.49E-01
MSMEG_4424	endoribonuclease L-PSP		0.90	3.04E-01	1.08	7.14E-01	1.00	9.57E-01
MSMEG_4425	putative oxidoreductase		1.05	4.60E-01	1.12	3.39E-01	0.96	4.98E-01
MSMEG_4426	secreted protein		1.05	2.42E-01	1.06	3.61E-01	1.02	5.52E-01
MSMEG_4427	transmembrane efflux pump		1.19	2.04E-01	1.05	4.94E-01	1.07	3.36E-01
MSMEG_4428	conserved hypothetical protein		1.58	1.85E-01	1.12	2.11E-01	1.16	1.57E-01
MSMEG_4429	hypothetical protein		0.94	7.61E-01	1.31	4.18E-01	0.98	2.69E-01
MSMEG_4430	transcriptional regulator		1.34	1.08E-01	1.10	3.81E-01	1.00	9.70E-01
MSMEG_4431	putative regulatory protein		4.46	1.85E-03	0.87	3.10E-01	4.44	8.42E-03
MSMEG_4432	hypothetical protein		1.11	4.83E-01	1.04	7.60E-01	0.94	1.64E-01
MSMEG_4433	dehydrogenase		1.20	2.50E-01	1.26	2.43E-01	0.95	1.67E-01
MSMEG_4434	Cof protein		1.20	3.07E-01	1.07	5.98E-01	1.12	4.31E-01
MSMEG_4435	dimethylmenaquinone methyltransferase		1.01	9.20E-01	1.82	1.18E-01	0.90	4.30E-01
MSMEG_4436	transketolase		1.17	2.85E-01	2.22	1.19E-01	1.02	7.85E-01
MSMEG_4437	cytochrome c oxidase, subunit I	*ctaD*	1.28	4.38E-01	1.16	5.31E-01	0.87	4.98E-01
MSMEG_4438	hypothetical protein		1.22	2.44E-01	1.09	5.61E-01	1.75	3.50E-01
MSMEG_4439	acetyltransferase		1.00	9.95E-01	0.93	6.59E-01	0.83	2.82E-01
MSMEG_4440	glucose-1-dehydrogenase		1.08	6.59E-01	1.30	9.79E-02	1.03	5.23E-01
MSMEG_4441	cupin domain protein		1.52	3.67E-01	1.21	2.27E-01	0.98	3.46E-01
MSMEG_4442	zinc-binding oxidoreductase		1.98	8.44E-02	1.17	4.11E-01	0.88	5.02E-01
MSMEG_4443	hypothetical protein		1.12	1.92E-01	0.96	4.56E-01	1.56	2.84E-01
MSMEG_4444	hypothetical protein		1.04	6.05E-01	1.17	9.24E-02	1.03	6.27E-01

MSMEG_4445	hypothetical protein		1.36	6.80E-02	1.38	4.09E-02	1.15	5.02E-01
MSMEG_4446	dihydrolipoamide dehydrogenase; this gene contains a premature stop which is not the result of sequencing error; identified by match to protein family HMM PF00070; match to protein family HMM PF02852; match to protein family HMM PF07992; match to protein family HMM TIGR01350	*lpdA*	3.20	4.12E-02	0.70	2.21E-01	1.22	3.49E-01
MSMEG_4447	conserved hypothetical protein		1.03	8.29E-02	0.86	6.23E-02	1.07	6.02E-01
MSMEG_4448	transcriptional modulator of MazE		0.66	8.45E-03	0.90	3.11E-01	1.14	1.87E-01
MSMEG_4449	putative transcriptional regulator		0.82	7.95E-02	0.91	3.31E-01	1.31	8.01E-02
MSMEG_4450	alpha/beta hydrolase		0.67	2.24E-02	1.10	6.89E-01	0.95	2.52E-01
MSMEG_4451	probable monooxygenase		0.59	1.03E-02	0.83	8.71E-03	1.01	9.36E-01
MSMEG_4453	conserved hypothetical protein		0.99	9.49E-01	1.43	3.77E-01	0.95	6.63E-01
MSMEG_4454	L-threonine aldolase, low-specificity	*ltaE*	1.29	2.49E-02	0.82	6.69E-02	0.88	5.55E-01
MSMEG_4455	beta-lactamase		1.89	3.93E-03	1.03	7.72E-01	1.22	9.01E-02
MSMEG_4456	conserved hypothetical protein		3.70	2.43E-04	1.19	1.38E-01	0.59	3.04E-04
MSMEG_4457	regulatory protein, AsnC/Lrp		1.92	4.39E-01	1.42	1.57E-01	1.54	1.50E-01
MSMEG_4458	transmembrane transport protein		1.39	1.70E-01	0.99	8.39E-01	0.97	2.23E-01
MSMEG_4459	agmatinase	*speB*	1.66	5.04E-03	0.94	4.47E-01	0.89	4.56E-01
MSMEG_4460	acetolactate synthase		1.34	2.50E-02	1.12	3.05E-01	1.15	7.63E-02
MSMEG_4460	acetolactate synthase		1.21	9.61E-02	0.95	6.45E-01	0.91	1.51E-01
MSMEG_4461	conserved hypothetical integral membrane protein		0.75	5.70E-02	1.52	1.62E-01	1.06	8.02E-01
MSMEG_4462	sodium:solute symporter		1.11	3.20E-01	1.11	4.63E-01	0.92	1.29E-01
MSMEG_4463	CalR5 protein		0.67	3.91E-04	1.04	7.36E-01	1.05	2.74E-01
MSMEG_4464	modulator of DNA gyrase		0.86	7.26E-02	1.35	1.14E-01	0.91	4.03E-01
MSMEG_4465	cutinase		3.53	4.43E-03	1.79	1.10E-01	1.18	3.36E-01
MSMEG_4466	ABC transporter, permease protein UspA		1.04	4.52E-01	1.33	5.10E-01	0.94	1.30E-01
MSMEG_4467	ABC transporter, permease protein UspE		2.29	3.47E-01	1.00	9.73E-01	1.03	8.64E-01
MSMEG_4468	Bacterial extracellular solute-binding protein UspC		1.02	8.47E-01	1.13	3.98E-01	1.63	2.33E-01
MSMEG_4469	cobalt transport protein		1.16	2.10E-01	0.70	1.37E-03	9.00	2.99E-01
MSMEG_4470	ABC transporter		0.91	1.83E-01	0.80	1.46E-01	3.96	5.48E-02
MSMEG_4471	MarR-family protein transcriptional regulator		0.97	8.01E-01	0.81	3.96E-02	1.18	1.16E-01
MSMEG_4472	multidrug-efflux transporter protein		0.78	1.10E-02	0.82	1.88E-01	1.51	2.64E-02
MSMEG_4473	Integral membrane protein		1.06	5.27E-01	1.00	9.86E-01	1.30	9.48E-02
MSMEG_4474	acyl-CoA oxidase		1.23	1.57E-02	0.97	7.02E-01	0.78	5.94E-02
MSMEG_4475	LppP protein		0.70	1.38E-03	0.90	2.07E-01	2.17	9.78E-03

MSMEG_4476	hypothetical protein		1.58	1.06E-02	0.84	6.64E-02	0.87	1.34E-02
MSMEG_4477	hydrolase, alpha/beta hydrolase fold family protein		3.12	3.01E-02	0.98	9.44E-01	2.96	1.58E-03
MSMEG_4479	conserved hypothetical protein		1.48	1.20E-02	0.70	2.80E-02	1.21	3.89E-01
MSMEG_4480	conserved hypothetical protein		1.94	1.61E-02	0.73	1.95E-02	0.53	1.05E-04
MSMEG_4481	hypothetical protein		0.94	6.64E-01	1.06	7.28E-01	1.69	3.10E-01
MSMEG_4482	DNA primase		0.68	1.70E-03	1.10	1.90E-01	2.71	8.84E-03
MSMEG_4483	deoxyguanosinetriphosphate triphosphohydrolase		0.63	3.38E-04	1.29	5.66E-03	1.61	1.68E-01
MSMEG_4484	putative conserved transmembrane protein		0.95	3.97E-01	1.06	2.40E-01	0.78	1.87E-01
MSMEG_4485	glycyl-tRNA synthetase	*glyS*	0.44	6.38E-06	1.09	4.24E-01	1.20	3.64E-01
MSMEG_4486	transcriptional regulator, ArsR family protein		1.69	4.97E-03	0.80	1.26E-01	0.67	2.02E-02
MSMEG_4487	ferric uptake regulation protein		1.46	1.29E-02	0.74	3.11E-02	0.68	4.73E-02
MSMEG_4488	hydrolase, nudix family protein		0.53	2.58E-03	1.21	3.15E-01	1.42	2.85E-01
MSMEG_4489	conserved hypothetical protein		0.47	9.98E-05	1.32	1.11E-01	1.75	1.04E-01
MSMEG_4490	undecaprenyl diphosphate synthase	*uppS*	0.64	1.76E-02	1.22	2.26E-01	1.40	1.81E-02
MSMEG_4491	DNA repair protein RecO	*recO*	0.68	2.09E-02	1.08	3.64E-01	1.37	6.83E-02
MSMEG_4492	amidase		1.07	4.74E-01	0.80	1.18E-01	2.65	1.14E-02
MSMEG_4493	GTP-binding protein Era	*era*	0.57	6.82E-04	1.81	1.20E-01	3.05	8.16E-02
MSMEG_4494	conserved hypothetical protein		0.50	1.58E-04	1.51	7.88E-03	2.19	2.65E-03
MSMEG_4495	CBS domain protein		0.51	2.33E-04	2.67	1.12E-01	3.42	2.53E-03
MSMEG_4496	conserved hypothetical protein		0.86	2.11E-01	1.30	1.99E-01	2.67	4.33E-04
MSMEG_4497	PhoH family protein		1.00	9.95E-01	1.38	1.78E-01	1.67	1.13E-03
MSMEG_4498	hypothetical protein		1.15	3.85E-01	0.82	2.20E-01	1.24	2.80E-01
MSMEG_4499	hypothetical protein		1.11	6.81E-01	4.76	1.19E-01	2.98	1.61E-01
MSMEG_4500	hypothetical protein		0.86	3.62E-01	1.20	1.56E-01	1.47	1.79E-01
MSMEG_4501	sodium:dicarboxylate symporter		0.20	2.00E-03	1.08	7.40E-01	0.78	3.03E-01
MSMEG_4502	conserved hypothetical protein		1.07	5.33E-01	1.09	6.29E-02	9.68	3.65E-01
MSMEG_4503	hypothetical protein		1.29	1.41E-01	1.12	4.42E-01	1.07	6.07E-01
MSMEG_4504	chaperone protein DnaJ	*dnaJ*	0.89	2.65E-01	0.80	1.31E-02	1.51	1.12E-01
MSMEG_4505	heat-inducible transcription repressor HrcA	*hrcA*	1.47	2.20E-02	0.99	8.98E-01	1.17	2.18E-01
MSMEG_4506	hypothetical protein		0.58	2.40E-02	0.95	1.33E-01	1.71	3.27E-01
MSMEG_4507	conserved hypothetical protein		2.36	4.29E-04	0.69	7.73E-02	0.63	6.66E-03
MSMEG_4508	conserved domain protein		0.68	9.71E-02	1.20	4.24E-01	1.00	9.45E-01
MSMEG_4509	MbtG protein		0.22	4.43E-05	1.33	4.87E-02	2.82	1.95E-01
MSMEG_4510	peptide synthetase mbtf		0.39	1.60E-03	1.27	3.37E-01	2.26	1.72E-01
MSMEG_4511	linear gramicidin synthetase subunit B		0.35	1.04E-02	0.99	9.45E-01	3.31	2.05E-01
MSMEG_4512	polyketide synthetase mbtd		1.15	2.94E-02	1.12	5.01E-01	1.43	2.11E-01
MSMEG_4513	polyketide synthase		0.49	2.10E-02	1.16	1.81E-01	1.85	2.75E-01

MSMEG_4514	Thioesterase domain protein		0.55	1.23E-03	1.19	3.79E-02	1.71	3.60E-01
MSMEG_4515	putative non-ribosomal peptide synthetase		0.46	1.51E-02	1.43	3.66E-01	1.78	2.64E-01
MSMEG_4516	2,3-dihydroxybenzoate-AMP ligase		0.47	9.06E-04	0.94	6.39E-01	1.39	3.51E-01
MSMEG_4517	TetR-type transcriptional regulator of sulfur metabolism		1.28	4.52E-01	0.97	8.27E-01	2.56	3.82E-01
MSMEG_4518	conserved hypothetical protein		1.34	1.47E-01	2.02	2.88E-01	1.15	6.29E-01
MSMEG_4519	NrtC protein		1.51	1.87E-01	1.29	2.36E-01	1.33	4.81E-01
MSMEG_4520	sulfate/thiosulfate import ATP-binding protein CysA		1.09	4.72E-01	1.56	2.09E-01	1.27	5.03E-01
MSMEG_4521	Tat (twin-arginine translocation) pathway signal sequence		1.30	1.74E-01	1.04	7.33E-01	1.02	1.18E-01
MSMEG_4522	ISMsm2, transposase		1.10	1.46E-01	0.88	5.31E-02	0.62	2.14E-02
MSMEG_4522	ISMsm2, transposase		0.93	3.77E-01	0.98	5.22E-01	0.78	7.20E-02
MSMEG_4523	binding-protein-dependent transport systems inner membrane component		1.07	3.63E-01	1.13	5.28E-01	1.09	3.26E-01
MSMEG_4524	anthranilate synthase component I		0.32	2.26E-04	1.26	1.66E-01	1.97	3.76E-01
MSMEG_4525	putative oxygen-independent coproporphyrinogen III oxidase		1.70	2.41E-01	0.94	5.95E-01	1.04	7.49E-01
MSMEG_4526	hypothetical protein		0.92	1.08E-01	1.00	9.74E-01	0.96	4.27E-01
MSMEG_4527	ferredoxin sulfite reductase		0.82	3.03E-03	1.02	9.17E-01	5.39	4.95E-03
MSMEG_4528	phosphoadenosine phosphosulfate reductase		0.77	2.80E-04	1.19	3.52E-01	5.74	3.61E-03
MSMEG_4529	secreted protein		0.53	8.99E-05	1.07	5.82E-01	5.86	3.71E-03
MSMEG_4530	sulfate ABC transporter, ATP-binding protein	*cysA*	0.73	3.41E-03	1.27	2.51E-02	1.16	2.77E-03
MSMEG_4531	sulfate ABC transporter, permease protein CysW	*cysW*	0.33	1.05E-05	1.65	2.80E-01	3.81	8.72E-03
MSMEG_4532	sulfate ABC transporter, permease protein CysT	*cysT*	0.24	1.48E-05	1.36	1.99E-01	4.02	5.02E-03
MSMEG_4533	sulfate-binding protein		0.28	1.05E-05	1.81	1.55E-01	3.05	1.65E-02
MSMEG_4534	putative conserved membrane protein		0.60	3.80E-04	0.76	2.69E-02	1.03	5.12E-01
MSMEG_4535	glycoside hydrolase		1.00	8.49E-01	0.80	8.54E-02	0.76	2.67E-01
MSMEG_4536	conserved hypothetical protein		0.73	3.28E-02	2.46	1.56E-01	11.82	1.26E-02
MSMEG_4537	major membrane protein I		1.34	2.26E-03	1.02	8.25E-01	0.28	1.71E-05
MSMEG_4538	cysteine desulphurase, SufS		1.24	3.44E-02	1.14	2.23E-01	0.57	1.66E-02
MSMEG_4539	alkanesulfonate monooxygenase		1.04	8.82E-01	1.69	3.76E-02	1.11	8.69E-02
MSMEG_4540	acetyltransferase, gnat family protein		1.03	9.33E-01	1.88	1.72E-01	0.97	2.07E-01
MSMEG_4541	ABC transporter, ATP-binding protein		1.04	9.15E-01	1.25	3.29E-01	1.45	3.35E-01
MSMEG_4542	oligopeptide transport integral membrane protein		0.92	5.48E-01	1.12	6.31E-01	1.27	4.19E-01
MSMEG_4543	ABC transporter system integral membrane protein		1.14	5.41E-01	1.38	1.14E-01	0.99	6.37E-01

MSMEG_4544	methyltransferase small domain superfamily protein		0.79	3.15E-01	0.95	6.39E-01	1.19	1.14E-01
MSMEG_4545	bacterial extracellular solute-binding protein, family protein 5		1.29	1.07E-01	1.15	2.28E-01	0.95	3.24E-01
MSMEG_4546	oxidoreductase; this gene contains a frame shift which is not the result of sequencing error		2.52	2.83E-02	2.10	2.10E-01	0.90	1.77E-01
MSMEG_4547	acyl-CoA dehydrogenase family protein		1.03	9.02E-01	1.04	7.48E-01	1.12	1.86E-01
MSMEG_4548	acetyltransferase, gnat family protein		0.92	5.48E-01	1.45	2.61E-01	1.01	1.62E-01
MSMEG_4549	amino acid permease-associated region, putative		1.28	2.28E-01	1.46	1.21E-01	2.36	3.49E-01
MSMEG_4551	monoxygenase		1.03	6.49E-01	1.17	1.67E-01	1.01	7.81E-01
MSMEG_4552	nitrilotriacetate monooxygenase component A	*ssuD*	1.00	9.56E-01	1.53	7.70E-02	1.24	4.20E-01
MSMEG_4553	GAF domain, putative		1.22	1.05E-01	1.00	9.83E-01	0.53	5.82E-03
MSMEG_4554	probable serine/threonine-protein kinase PknH, putative		2.07	1.95E-02	1.09	4.56E-01	1.05	4.03E-01
MSMEG_4555	LppR protein		1.11	5.87E-01	1.42	3.74E-01	0.94	2.43E-01
MSMEG_4556	GTP-binding protein LepA	*lepA*	0.67	1.23E-03	0.83	2.38E-01	1.19	3.56E-02
MSMEG_4557	ABC transporter, ATP-binding protein		0.27	1.94E-05	0.93	5.71E-01	3.14	1.68E-01
MSMEG_4558	conserved hypothetical protein		2.39	7.38E-04	0.82	9.30E-02	1.00	9.82E-01
MSMEG_4559	ABC transporter, membrane spanning protein		0.33	4.05E-05	0.85	9.76E-02	1.76	1.13E-01
MSMEG_4560	periplasmic binding protein		0.31	1.30E-05	1.01	9.08E-01	2.26	9.21E-02
MSMEG_4561	ABC Fe3+-siderophores transporter, periplasmic binding protein		0.35	4.65E-05	0.85	1.62E-01	2.32	2.30E-01
MSMEG_4562	conserved hypothetical protein; this gene contains a frame shift which is not the result of sequencing error		1.54	1.23E-01	1.47	3.01E-01	0.93	4.74E-01
MSMEG_4563	puromycin N-acetyltransferase		0.75	1.56E-02	1.04	5.35E-01	1.02	8.85E-01
MSMEG_4564	CBS domain protein		1.14	1.48E-01	0.92	3.96E-01	0.51	9.04E-04
MSMEG_4565	oxidoreductase		1.56	6.98E-02	1.12	3.51E-01	0.93	1.71E-01
MSMEG_4566	conserved 13e12 repeat family protein; this gene contains a frame shift which is not the result of sequencing error		1.41	2.79E-02	1.27	1.93E-01	1.86	1.72E-01
MSMEG_4566	conserved 13e12 repeat family protein; this gene contains a frame shift which is not the result of sequencing error		1.21	1.98E-01	1.05	6.82E-01	1.16	4.83E-01
MSMEG_4567	conserved hypothetical protein		0.57	4.42E-06	1.12	3.76E-01	0.82	1.59E-01
MSMEG_4568	ribonuclease Z		2.68	7.80E-02	1.05	5.11E-01	0.23	3.92E-04
MSMEG_4569	conserved hypothetical protein		0.68	7.14E-03	0.90	1.08E-02	1.01	9.40E-01
MSMEG_4570	conserved hypothetical protein		0.55	2.02E-03	0.96	7.67E-01	0.60	6.01E-03

MSMEG_4571	ribosomal protein S20	*rpsT*	1.47	1.41E-03	0.76	1.94E-02	0.76	1.21E-03
MSMEG_4572	DNA polymerase III, delta subunit		0.82	1.97E-01	1.01	8.57E-01	1.23	3.52E-01
MSMEG_4573	competence protein		1.06	6.26E-01	1.38	2.05E-01	1.06	6.86E-01
MSMEG_4574	DNA-binding protein		1.01	9.09E-01	1.01	9.58E-01	0.89	4.09E-01
MSMEG_4575	hypothetical protein		2.10	8.88E-03	0.88	4.82E-01	2.33	1.05E-01
MSMEG_4576	SpfH domain protein		7.95	1.17E-02	0.84	5.41E-01	2.15	2.19E-01
MSMEG_4577	DegV family protein		0.50	2.16E-02	1.47	4.54E-02	1.32	3.96E-01
MSMEG_4578	conserved hypothetical protein		0.45	1.33E-04	1.20	3.28E-01	1.56	1.54E-01
MSMEG_4579	phosphoglycerate mutase family protein		0.60	1.21E-03	0.91	3.43E-01	2.30	1.94E-02
MSMEG_4580	conserved domain protein		0.59	7.69E-04	0.99	8.49E-01	2.11	1.91E-03
MSMEG_4581	nicotinate (nicotinamide) nucleotide adenylyltransferase	*nadD*	0.66	1.20E-03	1.05	3.67E-01	1.35	1.36E-03
MSMEG_4582	conserved hypothetical protein		1.31	9.42E-02	0.84	9.44E-02	0.93	2.82E-01
MSMEG_4582	conserved hypothetical protein		0.89	2.00E-01	0.99	8.78E-01	1.11	2.89E-01
MSMEG_4583	ATPase associated with various cellular activities		0.97	7.41E-01	0.82	9.21E-03	0.84	9.02E-02
MSMEG_4584	gamma-glutamyl phosphate reductase	*proA*	1.14	5.80E-02	0.93	5.69E-01	0.84	1.07E-01
MSMEG_4585	ribokinase	*rbsK*	1.31	5.71E-02	0.95	7.54E-01	1.24	2.39E-01
MSMEG_4586	ABC nitrate/sulfonate/bicarbonate family protein transporter, periplasmic ligand binding protein		1.46	6.65E-02	2.67	2.80E-01	2.97	9.44E-02
MSMEG_4587	putative aliphatic sulfonates transport ATP-binding protein SsuB		1.62	9.39E-02	2.46	2.16E-01	1.29	3.44E-01
MSMEG_4588	ABC nitrate/sulfonate/bicarbonate family protein transporter, inner membrane subunit		1.43	4.44E-03	2.38	3.23E-01	1.82	1.88E-01
MSMEG_4589	rhodanese domain protein/cystathionine beta-lyase		1.45	1.04E-01	2.80	2.67E-01	2.06	6.84E-02
MSMEG_4590	nitrilotriacetate monooxygenase component A	*ssuD*	1.83	6.67E-02	2.42	2.96E-01	1.44	1.75E-01
MSMEG_4591	hypothetical protein		1.68	2.45E-02	1.08	4.95E-01	3.59	2.28E-01
MSMEG_4592	acyl-CoA dehydrogenase family protein		1.53	6.83E-02	2.92	1.88E-01	1.32	1.45E-01
MSMEG_4593	acyl-CoA dehydrogenase		0.73	1.60E-02	2.97	3.04E-01	1.05	2.09E-01
MSMEG_4594	hypothetical protein		1.92	5.24E-02	0.91	4.84E-01	1.01	9.09E-01
MSMEG_4595	probable serine/threonine-protein kinase PknH, putative		0.71	3.28E-03	1.34	2.59E-02	0.71	6.66E-02
MSMEG_4596	hypothetical protein		0.72	2.38E-03	1.23	1.26E-01	0.70	6.56E-02
MSMEG_4597	putative conserved lipoprotein lpph	*lppH*	0.68	1.07E-02	1.25	5.53E-03	1.67	9.55E-02
MSMEG_4598	putative conserved lipoprotein lpph	*lppH*	0.92	1.03E-01	1.14	1.44E-01	0.75	2.82E-02
MSMEG_4599	hypothetical protein		1.24	6.37E-02	1.32	1.89E-01	1.19	2.12E-01
MSMEG_4600	hypothetical protein		1.19	3.07E-01	0.84	8.76E-02	0.93	6.62E-01
MSMEG_4601	transcriptional regulator, AraC family protein		4.63	3.17E-01	1.06	5.19E-01	0.95	7.02E-01

MSMEG_4602	hypothetical protein		1.39	5.59E-02	0.98	8.41E-01	1.14	2.52E-01
MSMEG_4603	integral membrane protein; this gene contains a frame shift which is not the result of sequencing error; identified by match to protein family HMM PF01925		0.74	3.30E-02	1.10	3.17E-01	1.48	1.11E-01
MSMEG_4604	hypothetical protein		0.57	5.71E-02	1.10	1.12E-01	3.80	2.26E-01
MSMEG_4605	ankyrin; this gene contains a premature stop which is not the result of sequencing error		0.62	5.32E-03	1.20	5.34E-02	3.22	7.44E-03
MSMEG_4605	ankyrin; this gene contains a premature stop which is not the result of sequencing error		0.95	4.73E-01	0.99	9.13E-01	2.21	1.35E-02
MSMEG_4606	hypothetical protein		1.67	8.71E-02	1.11	1.94E-01	2.00	1.77E-01
MSMEG_4606	hypothetical protein		1.11	3.42E-01	0.92	3.66E-01	1.01	9.13E-01
MSMEG_4607	hypothetical protein		1.30	9.43E-02	1.19	1.58E-01	0.79	2.20E-01
MSMEG_4609	transcriptional regulator LysR family protein		0.98	9.05E-01	1.08	1.45E-01	0.94	3.51E-01
MSMEG_4610	IgiC, putative		1.86	3.68E-02	0.97	6.75E-01	0.70	3.95E-02
MSMEG_4611	carrier protein		1.54	7.14E-03	0.94	6.89E-01	0.99	9.55E-01
MSMEG_4612	conserved hypothetical protein; this gene contains a frame shift which is not the result of sequencing error; identified by match to protein family HMM PF04978		0.58	2.45E-03	1.24	1.21E-02	0.97	2.80E-01
MSMEG_4612	conserved hypothetical protein; this gene contains a frame shift which is not the result of sequencing error; identified by match to protein family HMM PF04978		0.72	8.42E-03	1.01	8.41E-01	1.50	3.61E-01
MSMEG_4613	hypothetical protein		1.34	3.13E-02	1.12	1.95E-01	1.32	2.10E-01
MSMEG_4614	GAF family protein		0.74	1.78E-03	1.09	4.58E-01	0.94	7.65E-01
MSMEG_4615	replicative DNA helicase; this region contains one or more premature stops and/or frameshifts which are not the result of sequencing error		0.88	1.01E-01	1.38	6.25E-03	1.46	1.24E-01
MSMEG_4615	replicative DNA helicase; this region contains one or more premature stops and/or frameshifts which are not the result of sequencing error		2.37	1.85E-01	1.09	8.51E-02	0.98	9.41E-01
MSMEG_4616	acyl-CoA dehydrogenase family protein; this gene contains a frame shift which is not the result of sequencing error		1.20	1.86E-01	1.21	4.59E-03	1.00	9.07E-01
MSMEG_4617	glutamine-dependent NAD+ synthetase	*nadE*	1.04	6.48E-01	0.66	2.77E-03	2.19	1.46E-01
MSMEG_4618	isochorismatase family protein		1.34	3.52E-01	2.00	5.67E-02	0.87	5.40E-01
MSMEG_4619	putative cytochrome P450 126		1.51	2.92E-02	1.02	8.98E-01	1.17	4.51E-01

MSMEG_4620	NAD-dependent deacetylase 1		1.91	5.69E-03	0.82	1.13E-01	1.36	2.56E-02
MSMEG_4621	glutamate 5-kinase	*proB*	0.75	6.71E-03	0.76	7.05E-02	0.92	5.02E-01
MSMEG_4622	transcriptional regulator, TetR family protein		1.27	3.64E-02	1.58	4.29E-01	2.14	3.60E-01
MSMEG_4623	GTP-binding protein Obg/CgtA		0.95	6.97E-01	0.73	3.77E-03	1.01	9.21E-01
MSMEG_4624	ribosomal protein L27	*rpmA*	1.02	3.04E-01	0.83	2.42E-01	0.62	2.62E-02
MSMEG_4625	ribosomal protein L21	*rplU*	0.97	6.36E-01	0.77	7.99E-02	0.72	1.01E-02
MSMEG_4626	ribonuclease, Rne/Rng family protein		0.82	6.38E-04	0.86	5.05E-02	1.24	1.90E-01
MSMEG_4627	nucleoside diphosphate kinase		0.41	1.14E-04	0.81	7.97E-02	0.57	2.48E-03
MSMEG_4628	conserved hypothetical protein		0.39	2.85E-04	0.92	3.35E-01	2.06	2.90E-03
MSMEG_4629	FolC bifunctional protein; this gene contains a frame shift which is not the result of sequencing error; identified by match to protein family HMM PF02875; match to protein family HMM TIGR01499	*folC*	0.85	9.57E-02	1.30	4.00E-01	1.54	5.12E-02
MSMEG_4630	valyl-tRNA synthetase	*valS*	0.53	1.17E-04	1.13	1.55E-01	2.42	1.32E-04
MSMEG_4631	conserved hypothetical protein		1.27	1.23E-02	0.79	9.31E-02	1.28	8.52E-02
MSMEG_4632	saccharopine dehydrogenase		0.85	1.10E-01	0.86	1.47E-02	1.50	7.94E-02
MSMEG_4633	peptidase S9, prolyl oligopeptidase		0.97	5.29E-01	0.99	8.91E-01	0.95	1.76E-01
MSMEG_4634	hypothetical protein		0.85	2.24E-01	1.34	3.16E-01	1.22	1.77E-01
MSMEG_4635	ammonium transporter family protein		0.10	1.03E-05	2.52	2.96E-01	0.60	1.25E-01
MSMEG_4636	hypothetical protein		0.48	3.24E-02	1.59	3.05E-01	1.04	8.20E-01
MSMEG_4637	conserved hypothetical protein		0.35	1.07E-02	1.73	3.63E-02	1.02	7.27E-01
MSMEG_4638	vanillate O-demethylase oxidoreductase		0.24	2.37E-03	1.33	5.29E-02	1.02	6.72E-01
MSMEG_4639	conserved hypothetical protein		0.29	7.67E-04	1.30	4.23E-01	1.67	4.41E-01
MSMEG_4640	secreted protein		1.46	2.12E-02	0.93	6.11E-01	0.74	1.18E-01
MSMEG_4641	salicylate hydroxylase		0.81	1.08E-01	0.83	1.67E-01	1.03	6.83E-01
MSMEG_4642	hypothetical protein		1.12	7.26E-01	1.11	2.26E-01	1.17	5.46E-01
MSMEG_4643	resuscitation-promoting factor		0.79	1.96E-02	1.26	2.86E-01	1.19	2.66E-01
MSMEG_4644	molybdopterin-guanine dinucleotide biosynthesis protein A		1.46	4.62E-03	1.18	2.84E-01	0.83	1.83E-01
MSMEG_4645	alpha oxoglutarate ferredoxin oxidoreductase, beta subunit	*orB*	1.35	3.11E-02	0.91	1.53E-01	4.13	2.05E-02
MSMEG_4646	pyruvate synthase		1.69	4.36E-03	0.76	5.30E-02	3.57	2.86E-03
MSMEG_4647	carbohydrate kinase, PfkB, putative		0.58	6.51E-04	0.81	1.50E-01	2.83	6.10E-03
MSMEG_4648	DNA-binding protein		1.31	4.33E-01	1.41	1.01E-01	0.95	5.85E-01
MSMEG_4649	phytanoyl-CoA dioxygenase		2.23	7.90E-03	1.32	2.61E-01	0.88	1.89E-01
MSMEG_4650	hypothetical oxidoreductase YisS		2.70	6.68E-04	1.06	6.60E-01	0.62	1.08E-02
MSMEG_4651	AP endonuclease, family protein 2		2.51	1.07E-02	1.38	2.40E-01	0.92	3.18E-01

MSMEG_4652	D-oliose 4-ketoreductase		2.23	3.66E-03	0.74	7.44E-02	0.92	1.58E-01
MSMEG_4653	AP endonuclease, family protein 2 superfamily protein		2.32	1.47E-02	0.73	6.38E-02	1.48	1.85E-01
MSMEG 4654	aldo/keto reductase		4.47	1.81E-02	0.60	1.29E-02	0.95	6.61E-01
MSMEG 4655	transcriptional regulator, AraC family protein		1.36	3.10E-02	1.16	4.02E-01	0.86	1.63E-01
MSMEG_4656	sugar ABC transporter ATP-binding protein		3.61	4.14E-03	1.10	5.13E-01	0.17	3.83E-05
MSMEG_4657	ABC transporter membrane protein		4.53	2.76E-03	0.80	1.45E-01	0.09	8.59E-08
MSMEG 4658	sugar ABC transporter substrate-binding protein		3.49	1.71E-03	0.97	7.78E-01	0.11	4.57E-06
MSMEG 4659	GntR-family protein transcriptional regulator		2.27	1.47E-03	0.77	1.58E-02	0.79	2.96E-02
MSMEG_4660	hypothetical protein		1.33	5.73E-02	1.06	6.28E-01	1.11	3.65E-01
MSMEG_4661	sugar kinase, ribokinase family protein		3.35	2.12E-03	0.87	3.38E-01	0.87	3.86E-01
MSMEG_4662	deoxyribose-phosphate aldolase superfamily protein		4.50	1.79E-03	0.81	1.61E-01	1.93	4.83E-01
MSMEG 4663	protein IolB		5.97	2.29E-03	0.76	9.01E-02	0.84	2.42E-01
MSMEG_4664	IolD protein		4.24	2.05E-03	0.68	2.16E-02	0.73	7.42E-03
MSMEG 4665	IolE protein		4.19	1.53E-03	0.76	6.90E-02	0.76	6.74E-02
MSMEG 4666	myo-inositol 2-dehydrogenase		2.85	2.71E-02	0.71	6.60E-02	1.15	3.79E-01
MSMEG_4667	transcriptional regulator, LysR family protein		1.77	3.38E-03	0.96	4.65E-01	1.20	5.08E-01
MSMEG_4668	oxidoreductase alpha (molybdopterin) subunit		1.93	8.32E-03	0.80	6.87E-02	0.74	4.11E-02
MSMEG_4669	formate dehydrogenase family protein accessory protein FdhD	*fdhD*	1.08	3.70E-01	1.11	4.32E-01	1.08	6.40E-01
MSMEG 4670	N-glycosylation		1.26	3.53E-01	1.22	1.64E-01	0.89	2.66E-01
MSMEG_4671	ATP-dependent Clp protease, ATP-binding subunit ClpX	*clpX*	2.43	4.15E-05	0.92	5.53E-01	1.03	4.91E-01
MSMEG_4672	Clp protease	*clpP*	1.17	8.32E-02	0.73	1.23E-01	0.82	1.15E-02
MSMEG 4673	Clp protease	*clpP*	1.20	5.68E-02	0.88	3.87E-01	0.87	1.45E-01
MSMEG 4674	trigger factor	*tig*	0.63	5.21E-04	0.82	3.27E-02	1.18	3.77E-01
MSMEG_4677	hypothetical protein		0.72	2.31E-01	1.21	2.69E-01	1.10	4.83E-01
MSMEG_4678	hypothetical protein		0.74	7.74E-03	1.12	1.10E-01	0.56	3.46E-04
MSMEG 4679	hypothetical protein		0.59	1.49E-03	1.12	1.84E-01	0.94	6.45E-01
MSMEG 4680	carboxylesterase, putative	*lipP*	1.22	2.56E-01	0.69	4.89E-02	0.94	6.65E-01
MSMEG_4681	conserved hypothetical protein		1.93	9.23E-03	0.82	1.94E-02	1.06	4.98E-01
MSMEG 4682	Na+/H+ antiporter		0.76	7.68E-04	0.96	6.30E-01	0.54	6.92E-04
MSMEG 4683	putative formamidopyrimidine-DNA glycosylase		0.82	1.49E-01	1.29	4.76E-02	1.49	1.71E-01
MSMEG 4684	ribose 5-phosphate isomerase		0.70	5.58E-04	1.06	2.26E-01	0.77	2.73E-03
MSMEG_4685	oxidoreductase		0.70	4.88E-03	0.91	3.06E-01	0.67	5.89E-02
MSMEG 4686	putative oxidoreductase YdbC		0.77	2.91E-02	0.69	6.68E-02	0.87	2.55E-01
MSMEG 4687	cytosine deaminase		0.89	1.82E-01	0.99	9.19E-01	0.36	8.94E-04

MSMEG_4688	conserved hypothetical protein, putative		2.28	3.01E-02	0.75	7.22E-02	0.41	9.10E-04
MSMEG_4689	conserved hypothetical protein		1.71	1.70E-02	0.68	6.57E-02	0.44	3.62E-03
MSMEG_4690	aminopeptidase N	*pepN*	0.41	1.25E-05	0.99	8.68E-01	0.87	2.94E-02
MSMEG_4691	HNH nuclease		0.54	1.33E-02	0.92	4.53E-01	1.22	2.25E-01
MSMEG_4692	conserved hypothetical protein		0.30	4.23E-07	0.89	3.68E-01	0.73	3.92E-02
MSMEG_4693	conserved hypothetical protein		0.39	1.29E-05	0.87	1.47E-02	0.64	2.24E-02
MSMEG_4694	HNH endonuclease family protein		3.20	2.98E-06	0.87	2.06E-01	0.53	9.37E-04
MSMEG_4695	protozoan/cyanobacterial globin family protein		1.70	8.38E-04	0.91	3.40E-01	0.90	6.28E-01
MSMEG_4696	alpha-amylase family protein		0.95	6.48E-01	0.90	7.24E-02	1.72	4.72E-02
MSMEG_4697	conserved hypothetical protein		0.28	9.10E-04	1.21	1.22E-01	1.44	2.04E-01
MSMEG_4698	conserved hypothetical protein		0.61	6.83E-03	0.81	1.02E-01	1.48	1.27E-01
MSMEG_4699	NAD-glutamate dehydrogenase		0.76	5.72E-03	0.91	2.12E-01	1.59	9.11E-02
MSMEG_4700	ABC-transporter protein, ATP binding component		0.72	7.18E-03	1.14	2.75E-01	1.19	5.06E-02
MSMEG_4701	single-strand binding protein		1.05	7.74E-01	6.42	1.39E-01	1.25	4.36E-01
MSMEG_4702	ABC-type transporter, permease components		0.67	2.97E-04	0.83	8.42E-03	3.55	1.09E-02
MSMEG_4703	glycerol-3-phosphate acyltransferase		0.80	2.77E-03	0.92	1.04E-01	1.02	4.99E-01
MSMEG_4704	acyltransferase family protein		0.98	8.21E-01	0.89	1.47E-01	1.45	3.02E-01
MSMEG_4705	acyltransferase, ws/dgat/mgat subfamily protein		1.27	1.51E-01	0.92	1.40E-01	1.77	1.38E-01
MSMEG_4707	non-heme bromoperoxidase BPO-A2		0.81	4.46E-01	1.19	1.19E-01	1.02	7.58E-01
MSMEG_4708	putative methyltransferase		1.01	9.17E-01	0.80	1.32E-02	1.85	1.69E-01
MSMEG_4709	enoyl-CoA hydratase		0.60	5.50E-05	0.85	8.55E-02	1.38	2.19E-02
MSMEG_4710	dihydrolipoamide acetyltransferase		8.15	5.62E-02	1.08	7.79E-01	1.39	2.23E-02
MSMEG_4711	pyruvate dehydrogenase E1 component subunit beta		11.36	2.73E-02	0.89	6.71E-01	1.06	5.88E-01
MSMEG_4712	pyruvate dehydrogenase E1 component, alpha subunit		5.39	2.72E-03	1.19	3.55E-01	0.58	1.00E-03
MSMEG_4713	HpcH/HpaI aldolase/citrate lyase family protein		1.25	2.79E-02	0.94	3.96E-01	1.65	7.24E-02
MSMEG_4714	MoaC domain protein		1.39	1.35E-02	1.02	7.07E-01	1.88	9.84E-02
MSMEG_4715	acyl-CoA dehydrogenase		1.64	9.96E-04	0.97	4.65E-01	1.61	2.15E-01
MSMEG_4716	acetyl-/propionyl-coenzyme A carboxylase alpha chain		1.44	3.92E-03	0.92	3.99E-01	2.04	1.80E-01
MSMEG_4717	Carboxyl transferase domain protein		1.05	4.81E-01	2.31	1.18E-01	1.06	5.62E-01
MSMEG_4718	transcriptional regulator, TetR family protein		1.58	1.31E-01	1.00	9.74E-01	1.11	7.37E-01
MSMEG_4719	peptidase S9, prolyl oligopeptidase		0.73	2.66E-02	0.86	1.39E-02	1.25	2.96E-01
MSMEG_4720	conserved hypothetical protein		1.51	7.23E-03	0.80	1.21E-01	1.61	2.45E-02
MSMEG_4721	permease of the major facilitator superfamily protein		0.66	2.15E-02	0.75	4.36E-02	1.14	5.59E-01

MSMEG_4722	short-chain dehydrogenase		0.56	3.81E-04	0.84	9.29E-02	1.02	8.88E-01
MSMEG_4723	conserved hypothetical protein		0.59	3.23E-04	0.69	4.63E-03	0.55	5.03E-03
MSMEG_4724	oligoribonuclease		0.72	4.05E-03	0.99	8.94E-01	0.84	9.10E-02
MSMEG_4726	carbamoyl-phosphate synthase small chain		1.13	3.56E-01	1.07	3.22E-01	1.04	5.84E-01
MSMEG_4727	mycocerosic acid synthase		0.66	1.53E-01	1.15	4.95E-01	1.00	9.77E-01
MSMEG_4728	condensation domain protein		0.57	3.70E-02	0.97	7.83E-01	1.12	7.05E-01
MSMEG_4729	conserved hypothetical protein		0.50	6.95E-04	1.05	6.96E-01	1.05	2.23E-01
MSMEG_4730	conserved hypothetical protein		0.77	7.16E-03	0.70	1.04E-03	0.87	7.30E-02
MSMEG_4730	conserved hypothetical protein		0.47	9.47E-03	1.04	5.59E-01	1.02	2.11E-01
MSMEG_4731	acyl-CoA synthase		0.65	1.44E-02	0.93	5.46E-01	2.74	5.79E-03
MSMEG_4732	glycosyl transferase, group 2 family protein, putative		1.11	3.48E-01	1.06	7.10E-01	1.13	5.83E-01
MSMEG_4733	putative membrane protein		0.58	1.03E-03	0.81	8.63E-02	1.75	8.64E-02
MSMEG_4734	hypothetical protein		0.43	2.04E-04	0.82	1.39E-01	1.73	2.77E-01
MSMEG_4735	conserved hypothetical protein		0.55	5.13E-03	0.85	3.20E-01	1.22	2.13E-01
MSMEG_4736	conserved hypothetical protein		0.74	1.71E-02	1.03	8.07E-01	3.46	3.49E-01
MSMEG_4737	conserved hypothetical protein		1.54	1.26E-02	0.99	8.83E-01	1.20	3.47E-01
MSMEG_4738	hypothetical protein		1.04	8.00E-01	0.98	7.50E-01	1.41	4.28E-01
MSMEG_4739	conserved hypothetical protein		1.17	5.04E-01	0.66	1.89E-02	1.16	2.48E-01
MSMEG_4740	Glycosyltransferase family protein 28		1.46	1.09E-01	0.80	1.98E-01	1.07	1.41E-01
MSMEG_4741	MmpL protein		1.47	1.76E-01	0.64	1.85E-02	1.49	1.58E-01
MSMEG_4742	clavaldehyde dehydrogenase		0.78	5.24E-03	0.81	1.04E-02	0.40	6.06E-04
MSMEG_4743	conserved hypothetical protein		2.75	4.05E-03	1.01	9.27E-01	0.38	5.39E-03
MSMEG_4744	conserved hypothetical protein		2.14	1.24E-02	1.13	3.43E-01	0.47	3.72E-03
MSMEG_4745	ErfK/YbiS/YcfS/YnhG family protein		1.01	8.51E-01	0.96	6.41E-01	1.61	1.15E-02
MSMEG_4747	hypothetical protein		1.05	8.18E-01	1.08	8.06E-01	2.18	4.67E-01
MSMEG_4748	hypothetical protein		4.76	3.94E-02	1.18	5.97E-01	0.66	8.29E-03
MSMEG_4749	hypothetical protein		0.60	2.56E-03	1.29	1.72E-01	1.06	7.14E-01
MSMEG_4750	ornithine carbamoyltransferase		0.55	9.47E-03	1.29	7.93E-02	0.95	4.27E-01
MSMEG_4751	hypothetical protein		2.03	2.17E-02	1.02	8.90E-01	1.23	3.79E-01
MSMEG_4752	conserved hypothetical protein		0.60	3.45E-06	0.97	8.11E-01	1.27	1.08E-01
MSMEG_4753	antioxidant, AhpC/TSA family protein		0.86	5.28E-02	0.82	1.07E-01	1.07	4.32E-01
MSMEG_4754	K+-dependent Na+/Ca+ exchanger related-		0.26	1.20E-05	1.15	3.54E-01	1.89	3.48E-02
MSMEG_4755	peptidase M20		0.53	2.09E-04	0.85	1.13E-01	1.23	1.62E-01
MSMEG_4756	holo-(acyl-carrier-protein) synthase	*acpS*	0.26	2.31E-06	1.08	6.24E-01	1.83	6.02E-02
MSMEG_4757	fatty acid synthase		0.34	2.91E-05	0.69	2.56E-02	1.51	1.00E-01
MSMEG_4759	hypothetical protein		1.47	8.84E-03	1.32	8.98E-02	1.34	5.75E-02
MSMEG_4759	hypothetical protein		1.19	4.76E-02	0.89	3.13E-01	1.04	8.49E-01

MSMEG_4760	conserved hypothetical protein; this region contains one or more premature stops and/or frameshifts which are not the result of sequencing error		1.39	2.43E-01	1.16	5.79E-01	1.11	4.02E-01
MSMEG_4761	ABC transporter ATP-binding protein		0.66	3.97E-05	0.96	6.43E-01	1.45	2.25E-01
MSMEG_4762	ABC transporter binding protein		0.60	5.06E-03	0.90	1.19E-01	0.89	2.74E-01
MSMEG_4763	ABC transporter permease protein		0.65	9.43E-03	0.91	1.20E-01	0.90	2.81E-01
MSMEG_4764	ABC transporter permease protein		2.55	1.64E-01	0.94	4.83E-01	0.92	2.38E-01
MSMEG_4765	transcriptional regulator, MerR family protein		1.20	3.12E-01	1.10	7.25E-01	1.35	1.06E-01
MSMEG_4766	low temperature requirement protein LtrA	*ltrA*	0.81	4.09E-02	0.91	2.09E-01	1.80	2.98E-02
MSMEG_4767	major facilitator superfamily protein permease		0.37	4.34E-04	0.98	7.90E-01	9.11	3.75E-02
MSMEG_4768	transcriptional regulator		6.10	1.64E-02	0.37	7.08E-03	0.92	7.55E-01
MSMEG_4769	conserved hypothetical protein		7.18	2.80E-01	0.85	5.12E-01	0.92	7.62E-01
MSMEG_4770	conserved hypothetical protein		0.96	5.88E-01	0.88	2.48E-01	1.43	5.29E-01
MSMEG_4771	conserved hypothetical protein		1.07	3.72E-01	0.90	2.00E-01	0.95	6.64E-01
MSMEG_4772	acyl-CoA synthase		1.71	8.59E-02	0.91	2.73E-01	1.51	1.40E-01
MSMEG_4773	amidohydrolase family protein		3.07	5.86E-03	0.67	9.11E-03	0.56	1.59E-02
MSMEG_4774	AMP-dependent synthetase and ligase		2.27	3.92E-02	0.86	1.11E-01	1.00	9.57E-01
MSMEG_4775	enoyl-CoA hydratase/isomerase family protein		0.95	5.48E-01	0.87	4.58E-01	0.89	2.25E-01
MSMEG_4776	glyoxalase/bleomycin resistance protein/dioxygenase		1.65	3.66E-02	1.27	2.60E-01	1.09	3.53E-01
MSMEG_4777	conserved hypothetical protein		1.97	1.25E-02	0.74	1.12E-02	0.72	1.63E-02
MSMEG_4778	putative thiolase		1.99	1.92E-01	0.86	6.23E-02	0.79	2.02E-01
MSMEG_4779	probable regulatory protein		0.71	2.32E-03	0.94	6.00E-01	0.93	2.44E-01
MSMEG_4780	cytochrome p450		17.52	1.98E-02	0.53	3.45E-02	0.17	2.49E-05
MSMEG_4781	conserved hypothetical protein		8.03	3.50E-04	0.48	1.62E-02	0.22	2.20E-05
MSMEG_4781	conserved hypothetical protein		9.18	9.21E-04	0.57	2.75E-02	0.75	8.90E-02
MSMEG_4782	conserved hypothetical protein		4.76	1.01E-02	0.57	2.19E-02	0.77	9.57E-02
MSMEG_4783	conserved hypothetical protein		4.43	8.82E-03	0.71	2.03E-02	0.94	3.64E-01
MSMEG_4784	conserved hypothetical protein		6.52	8.33E-03	0.52	2.93E-02	0.87	1.49E-01
MSMEG_4785	mce-family protein mce1f		1.36	3.64E-02	1.00	9.28E-01	0.94	2.75E-01
MSMEG_4786	virulence factor mce family protein		0.88	2.03E-01	1.49	1.77E-01	0.96	1.43E-01
MSMEG_4787	virulence factor mce family protein		1.17	5.96E-01	1.63	5.35E-02	1.06	3.45E-01
MSMEG_4788	virulence factor mce family protein; this region contains one or more premature stops and/or frameshifts which are not the result of sequencing error		0.71	5.72E-02	0.86	4.11E-01	0.86	1.05E-01
MSMEG_4789	hypothetical protein		1.09	1.74E-01	1.01	8.81E-01	0.91	7.44E-01

MSMEG_4791	IS1096, tnpR protein	1.47	8.75E-02	0.65	4.70E-02	0.64	5.83E-03
MSMEG_4791	IS1096, tnpR protein	0.86	2.10E-01	1.03	5.42E-01	1.63	1.42E-02
MSMEG_4792	virulence factor mce family protein	3.84	8.84E-03	0.93	7.55E-01	0.99	6.20E-01
MSMEG_4793	virulence factor Mce family protein	3.11	1.53E-02	0.62	1.05E-02	0.96	3.03E-01
MSMEG_4794	virulence factor	1.75	3.25E-02	0.96	5.84E-01	1.10	7.33E-01
MSMEG_4795	ABC-transporter integral membrane protein	1.10	5.95E-01	0.86	2.06E-01	0.89	5.13E-01
MSMEG_4796	conserved hypothetical protein	1.39	8.59E-02	0.77	5.71E-02	0.79	5.16E-01
MSMEG_4797	hypothetical protein	3.54	2.13E-02	0.89	4.03E-01	0.96	7.49E-01
MSMEG_4798	L-carnitine dehydratase/bile acid-inducible protein F	1.92	1.32E-01	1.10	6.28E-01	1.00	9.48E-01
MSMEG_4799	aldehyde dehydrogenase (NAD) family protein	3.27	8.84E-04	0.91	2.63E-01	1.18	1.30E-01
MSMEG_4800	3-oxoacyl-(acyl-carrier-protein) reductase,	1.18	4.30E-01	1.15	3.59E-01	1.03	6.12E-01
MSMEG_4801	carveol dehydrogenase	2.77	2.66E-02	0.91	3.79E-01	1.14	6.04E-01
MSMEG_4802	conserved domain protein	2.59	1.79E-02	0.72	5.31E-02	1.28	5.46E-01
MSMEG_4803	cytochrome P450 superfamily protein	1.89	9.10E-02	0.79	3.21E-03	1.13	6.04E-01
MSMEG_4804	conserved hypothetical protein	2.35	2.02E-02	0.88	6.04E-01	0.62	1.39E-02
MSMEG_4805	acyl-CoA dehydrogenase	1.85	1.03E-01	1.01	8.45E-01	1.18	3.21E-01
MSMEG_4806	putative acyl-CoA dehydrogenase	1.29	3.06E-01	1.08	2.92E-01	0.80	4.21E-02
MSMEG_4807	amidohydrolase family protein	1.56	1.26E-01	0.81	1.27E-02	2.72	3.88E-01
MSMEG_4808	amidohydrolase family protein	2.20	1.42E-02	1.06	3.67E-01	0.94	4.48E-01
MSMEG_4809	Rieske [2Fe-2S] domain, putative	1.38	3.42E-02	0.95	5.35E-01	0.99	6.82E-01
MSMEG_4810	conserved hypothetical protein	1.80	2.64E-02	0.88	1.88E-01	1.77	3.26E-01
MSMEG_4811	conserved hypothetical protein	1.44	1.61E-01	0.88	4.16E-01	1.41	2.10E-01
MSMEG_4812	respiratory-chain NADH dehydrogenase domain, 51 kda subunit	1.35	1.27E-01	0.96	4.38E-01	1.39	2.26E-01
MSMEG_4813	conserved domain protein	1.52	5.73E-02	0.89	5.44E-01	1.12	2.12E-01
MSMEG_4814	conserved hypothetical protein	1.94	1.04E-01	0.96	5.38E-01	1.14	1.91E-01
MSMEG_4815	conserved hypothetical protein	1.67	8.50E-02	0.91	4.81E-01	1.00	9.90E-01
MSMEG_4816	6-phosphogluconate dehydrogenase, NAD-binding	1.17	2.13E-01	1.05	2.92E-01	1.33	1.82E-01
MSMEG_4817	6-phosphogluconate dehydrogenase, NAD-binding	1.24	3.73E-01	1.25	5.48E-01	1.18	3.10E-01
MSMEG_4818	putative cytochrome P450 123	1.59	1.59E-01	0.89	2.83E-01	1.13	5.87E-01
MSMEG_4819	conserved domain protein	2.02	1.55E-02	1.09	6.29E-01	1.54	4.15E-01
MSMEG_4820	conserved hypothetical protein	2.53	2.76E-01	1.13	1.61E-01	1.18	1.61E-01
MSMEG_4821	conserved hypothetical protein	3.19	1.33E-02	0.95	8.89E-01	0.97	2.38E-01
MSMEG_4822	conserved domain protein	2.41	5.15E-02	1.01	8.70E-01	0.93	3.73E-01
MSMEG_4823	cytochrome p450	1.89	3.28E-04	1.06	7.39E-01	1.01	9.56E-01

MSMEG_4824	hypothetical protein	1.35	1.08E-01	1.04	7.42E-01	0.85	2.55E-01
MSMEG_4825	transcriptional repressor, TetR family protein, putative	1.41	1.44E-01	0.96	7.82E-01	1.02	8.96E-01
MSMEG_4826	putative acyl-CoA dehydrogenase	1.31	4.61E-02	0.93	4.41E-01	0.66	2.55E-02
MSMEG_4827	aminoglycoside phosphotransferase	1.45	1.53E-02	0.72	2.45E-02	1.09	8.63E-01
MSMEG_4828	alcohol dehydrogenase, zinc-binding	0.81	1.55E-01	1.05	7.87E-01	1.14	4.26E-01
MSMEG_4829	cytochrome P450	2.50	2.70E-02	0.77	2.41E-02	0.67	4.52E-02
MSMEG_4830	conserved hypothetical protein	3.14	8.17E-03	1.07	6.31E-01	0.24	6.59E-06
MSMEG_4831	transcriptional regulator, TetR family protein	2.02	3.18E-02	0.91	6.05E-01	0.31	1.14E-03
MSMEG_4832	acyl-CoA dehydrogenase	1.41	7.47E-03	0.93	6.53E-01	1.09	3.66E-01
MSMEG_4833	putative acyl-CoA dehydrogenase	1.49	1.01E-03	0.80	5.70E-02	1.16	1.81E-01
MSMEG_4834	L-carnitine dehydratase/bile acid-inducible protein F	1.05	9.69E-02	0.83	1.92E-03	1.64	1.06E-01
MSMEG_4835	3-oxoacyl-[acyl-carrier-protein] reductase	1.37	1.68E-02	0.95	5.89E-01	1.04	4.19E-01
MSMEG_4836	FadE12_1	2.33	6.73E-03	0.60	1.99E-03	1.15	2.31E-01
MSMEG_4837	amidohydrolase 2	2.00	1.58E-01	0.77	7.08E-02	0.87	3.31E-01
MSMEG_4838	hypothetical protein	1.04	6.23E-01	1.17	4.95E-02	1.72	2.71E-01
MSMEG_4839	carboxymuconolactone decarboxylase	1.44	1.50E-01	0.90	3.95E-01	1.05	2.04E-01
MSMEG_4840	Rieske [2Fe-2S] domain protein	2.01	3.43E-02	0.68	2.12E-04	0.86	3.09E-01
MSMEG_4841	conserved hypothetical protein	1.50	1.79E-02	1.12	1.55E-01	0.95	2.82E-01
MSMEG_4842	amidohydrolase	2.39	2.75E-02	0.87	1.96E-01	1.02	8.16E-01
MSMEG_4843	conserved hypothetical protein	1.77	2.68E-02	1.05	6.86E-01	1.42	4.05E-01
MSMEG_4844	acyl-CoA dehydrogenase	2.60	2.45E-01	1.03	7.85E-01	1.00	9.80E-01
MSMEG_4845	putative acyl-CoA dehydrogenase	1.16	5.22E-01	0.85	4.44E-02	1.37	3.60E-01
MSMEG_4846	probable enoyl-CoA hydratase	1.08	3.46E-01	0.90	3.02E-01	1.03	6.15E-01
MSMEG_4847	transcriptional regulator, TetR family protein	1.17	2.08E-01	0.87	9.87E-02	0.81	1.90E-01
MSMEG_4848	hypothetical protein	0.70	3.81E-02	1.25	1.58E-01	1.38	1.81E-01
MSMEG_4849	carveol dehydrogenase	4.37	2.83E-03	0.87	1.90E-01	0.31	2.87E-05
MSMEG_4850	short-chain dehydrogenase/reductase SDR	2.73	5.62E-02	0.83	1.24E-01	1.02	7.77E-01
MSMEG_4851	caib/baif family protein	0.84	2.21E-01	1.04	6.39E-01	1.03	3.02E-01
MSMEG_4852	enoyl-CoA hydratase, putative	0.70	6.37E-03	0.95	1.80E-01	1.08	5.66E-01
MSMEG_4853	peptidase, M24 family protein	2.09	2.91E-02	1.47	5.15E-02	0.35	8.85E-03
MSMEG_4854	peptidase, M24 family protein	1.09	5.68E-01	0.92	5.04E-01	0.82	1.31E-01
MSMEG_4855	amidohydrolase family protein	1.29	3.19E-02	1.03	6.09E-01	1.23	6.84E-01
MSMEG_4856	cytochrome P450	1.41	6.37E-02	0.98	7.56E-01	0.86	4.66E-01
MSMEG_4857	conserved domain protein	1.15	1.74E-01	1.31	4.72E-01	0.92	2.79E-01
MSMEG_4858	transcriptional regulator, TetR family protein	0.94	3.00E-01	1.02	8.55E-01	1.12	4.06E-01

MSMEG_4859	oxidoreductase, short chain dehydrogenase/reductase family protein		1.41	2.98E-03	0.95	4.90E-01	0.98	6.60E-01
MSMEG_4860	hydrolase, alpha/beta fold family protein, putative		3.64	3.42E-01	1.18	4.41E-01	1.08	4.32E-01
MSMEG_4861	cyclase		3.04	3.64E-01	1.10	6.27E-01	0.98	4.42E-01
MSMEG_4862	3-alpha-hydroxysteroid dehydrogenase		1.42	3.19E-01	1.08	2.27E-01	1.47	3.56E-01
MSMEG_4863	conserved hypothetical protein		1.43	2.81E-03	1.00	9.76E-01	1.22	5.58E-01
MSMEG_4864	3-ketosteroid dehydrogenase		1.33	1.53E-01	0.91	3.74E-01	0.96	3.55E-01
MSMEG_4865	metapyrocatechase 1		0.85	9.49E-02	0.94	1.32E-01	1.14	1.61E-01
MSMEG_4866	3-(3-hydroxy-phenyl)propionate hydroxylase		1.29	1.32E-01	0.94	4.38E-01	1.13	9.51E-02
MSMEG_4867	2-hydroxy-6-ketonona-2,4-dienedoic acid hydrolase		1.26	1.51E-01	0.97	5.45E-01	1.06	5.31E-01
MSMEG_4868	transcriptional regulator, IclR family protein, C-domain protein		1.85	1.68E-01	1.28	1.02E-01	0.94	3.15E-01
MSMEG_4869	LAO/AO transport system ATPase		1.39	4.88E-02	0.96	7.99E-01	0.79	2.10E-01
MSMEG_4870	FAD binding domain, putative		0.95	4.75E-01	1.56	3.77E-01	1.14	3.57E-01
MSMEG_4871	3-hydroxyacyl-CoA dehydrogenase type-2		2.04	1.03E-01	1.25	4.98E-01	1.02	4.86E-01
MSMEG_4872	short chain dehydrogenase		0.90	3.01E-01	1.99	2.08E-01	0.98	8.89E-01
MSMEG_4873	enoyl-CoA hydratase/isomerase		1.38	2.80E-02	0.99	9.37E-01	0.95	6.32E-01
MSMEG_4874	putative acyl-CoA dehydrogenase		1.19	1.41E-01	1.00	1.00E+00	1.38	4.03E-01
MSMEG_4875	putative acyl-CoA dehydrogenase		1.14	4.40E-01	1.11	7.20E-01	0.99	8.97E-01
MSMEG_4876	acyl-CoA synthase		1.03	8.19E-01	0.99	8.68E-01	1.09	3.65E-01
MSMEG_4877	CaiB/BaiF family protein		1.88	9.91E-02	0.94	2.76E-01	1.02	8.46E-01
MSMEG_4878	2Fe-2S iron-sulfur cluster binding domain protein		1.29	2.71E-01	1.10	2.14E-01	0.99	9.55E-01
MSMEG_4879	conserved hypothetical protein		1.66	2.91E-02	0.88	2.52E-01	1.10	1.90E-01
MSMEG_4880	methylmalonyl-CoA mutase C-terminal domain protein		0.87	9.48E-02	1.16	7.01E-02	1.09	4.92E-01
MSMEG_4881	methylmalonyl-CoA mutase, N-terminus of large subunit		2.44	2.02E-01	2.08	2.76E-01	1.12	5.01E-01
MSMEG_4882	short chain dehydrogenase		1.59	2.17E-01	1.02	7.46E-01	0.92	1.69E-01
MSMEG_4883	AMP-dependent synthetase and ligase		1.13	4.81E-01	1.62	3.06E-01	1.79	3.84E-01
MSMEG_4884	conserved hypothetical protein		1.56	5.42E-02	1.03	7.33E-01	1.04	2.18E-01
MSMEG_4885	conserved hypothetical protein		1.04	8.99E-01	1.13	1.73E-01	0.98	3.17E-01
MSMEG_4886	major facilitator family protein transporter		1.21	2.72E-01	1.08	5.42E-01	0.95	1.88E-01
MSMEG_4887	cytochrome P450		1.36	4.36E-01	0.98	8.06E-01	0.99	7.78E-01
MSMEG_4888	periplasmic binding proteins and sugar binding domain of the LacI family protein, putative		1.54	1.37E-02	0.93	5.08E-01	1.18	4.73E-01
MSMEG_4889	integral membrane transport protein		1.01	9.56E-01	1.22	2.38E-01	0.95	5.02E-01
MSMEG_4890	alkylhydroperoxidase, AhpD family protein	*ahpD*	0.16	1.06E-07	0.80	3.01E-01	16.32	3.21E-02

MSMEG_4891	alkylhydroperoxide reductase		0.10	1.93E-07	0.63	2.27E-02	26.67	1.40E-02
MSMEG_4892	conserved hypothetical protein		0.79	3.06E-01	0.81	6.17E-02	2.39	3.70E-01
MSMEG_4893	Putative neutral zinc metallopeptidase		0.99	9.21E-01	0.86	1.92E-02	1.37	6.73E-02
MSMEG_4894	conserved hypothetical protein		0.91	2.71E-01	1.03	8.83E-01	1.69	2.53E-01
MSMEG_4895	hypothetical protein		1.11	6.05E-01	0.99	9.45E-01	1.09	3.74E-01
MSMEG_4896	non-ribosomal peptide synthetase		0.73	4.16E-02	0.81	1.33E-01	3.67	1.58E-02
MSMEG_4898	conserved hypothetical protein		0.50	2.33E-04	1.03	6.29E-01	9.08	6.25E-03
MSMEG_4899	non-canonical purine NTP pyrophosphatase, RdgB/HAM1 family protein	*rdgB*	0.34	3.48E-06	0.98	8.21E-01	1.66	1.20E-01
MSMEG_4900	Pks14 protein		0.67	4.19E-03	0.81	7.53E-02	6.77	2.29E-03
MSMEG_4901	ribonuclease PH	*rph*	0.74	2.75E-03	0.85	7.76E-02	1.86	5.26E-03
MSMEG_4902	metal-dependent hydrolase of the beta-lactamase superfamily protein III		1.20	5.93E-02	0.79	1.74E-02	2.27	5.94E-03
MSMEG_4903	glutamate racemase	*murI*	1.01	9.18E-01	0.88	1.53E-01	4.08	7.75E-02
MSMEG_4904	rhomboid family protein		1.27	1.50E-01	0.72	1.02E-02	1.13	1.78E-01
MSMEG_4905	cysteine synthase B	*cysM*	1.00	9.32E-01	0.85	9.16E-03	1.92	1.42E-03
MSMEG_4906	ThiS family protein		1.05	3.13E-01	0.77	1.21E-02	1.32	2.02E-03
MSMEG_4907	Mov34/MPN/PAD-1 family protein		0.98	6.74E-01	1.10	2.31E-01	2.35	1.36E-02
MSMEG_4908	endo-type 6-aminohexanoate oligomer hydrolase		1.01	6.96E-01	0.86	4.33E-02	2.70	2.53E-02
MSMEG_4909	conserved hypothetical protein		0.98	8.70E-01	0.80	6.27E-04	1.57	1.30E-01
MSMEG_4910	ATP-dependent Clp protease adaptor protein ClpS		1.60	2.40E-02	0.80	4.66E-03	1.44	1.57E-02
MSMEG_4911	putative nicotinate phosphoribosyltransferase	*pncB*	0.79	2.30E-03	0.87	1.44E-02	2.45	4.65E-04
MSMEG_4912	putative helicase		1.04	5.29E-01	0.98	9.07E-01	1.30	2.00E-01
MSMEG_4913	LpqM protein		1.76	2.05E-02	0.94	2.55E-01	0.80	1.83E-01
MSMEG_4914	virginiamycin B hydrolase		0.59	4.04E-04	0.90	2.99E-01	0.99	9.53E-01
MSMEG_4915	alpha-glucan phosphorylase family protein		1.02	7.20E-01	0.82	4.07E-02	0.55	5.12E-04
MSMEG_4916	alpha-amylase family protein		1.04	4.72E-01	1.35	8.57E-02	0.75	1.85E-02
MSMEG_4917	tetratricopeptide repeat domain protein		0.79	3.09E-02	0.87	8.31E-02	2.62	1.51E-03
MSMEG_4918	1,4-alpha-glucan branching enzyme	*glgB*	0.93	5.34E-01	1.16	3.01E-01	0.62	3.33E-03
MSMEG_4919	conserved hypothetical protein		0.22	2.54E-06	0.96	8.33E-01	25.98	1.19E-02
MSMEG_4920	acetyl-CoA acetyltransferase		0.40	7.04E-04	0.90	1.81E-01	3.61	1.59E-03
MSMEG_4921	methylmalonyl-CoA epimerase	*mce*	0.60	1.71E-02	1.21	2.46E-01	3.53	5.28E-03
MSMEG_4922	conserved hypothetical protein		0.58	1.11E-02	0.94	3.69E-01	0.93	3.41E-01
MSMEG_4923	conserved hypothetical protein		0.58	5.39E-04	0.81	8.56E-02	1.18	4.30E-01
MSMEG_4924	adenylate cyclase, family protein 3		0.75	1.90E-02	0.82	1.59E-01	1.03	8.86E-01
MSMEG_4925	transcriptional regulator, Ada family protein/DNA-3-methyladenine glycosylase II		2.10	9.72E-04	0.96	5.59E-01	0.80	9.42E-02

MSMEG_4926	IS1096, tnpA protein		1.16	4.33E-01	0.78	2.18E-02	1.87	5.82E-02
MSMEG_4926	IS1096, tnpA protein		0.99	9.26E-01	0.73	2.27E-02	1.32	1.55E-01
MSMEG_4927	IS1096, tnpR protein		1.02	8.58E-01	0.92	2.15E-01	1.41	1.39E-02
MSMEG_4928	methylated-DNA--protein-cysteine methyltransferase		2.33	3.95E-01	1.17	1.66E-01	0.92	3.33E-01
MSMEG_4932	UDP-N-acetylglucosamine 1-carboxyvinyltransferase	*murA*	0.96	8.40E-01	0.89	3.33E-04	0.87	2.10E-01
MSMEG_4933	conserved hypothetical protein		0.36	1.24E-04	0.88	1.13E-01	2.78	5.98E-02
MSMEG_4934	ATP:cob(I)alamin adenosyltransferase		1.23	3.27E-01	0.93	4.32E-01	0.91	2.35E-02
MSMEG_4935	ATP synthase F1, epsilon subunit	*atpC*	0.49	4.05E-03	0.93	1.59E-01	2.85	9.59E-03
MSMEG_4936	ATP synthase F1, beta subunit	*atpD*	0.40	7.20E-04	0.92	2.94E-01	3.04	1.49E-02
MSMEG_4937	ATP synthase F1, gamma subunit	*atpG*	0.37	8.65E-04	1.16	3.08E-01	2.52	6.71E-02
MSMEG_4938	ATP synthase F1, alpha subunit	*atpA*	0.71	2.89E-02	0.83	3.48E-02	1.58	5.83E-02
MSMEG_4939	ATP synthase delta chain		0.63	1.77E-03	1.01	7.91E-01	1.55	1.99E-01
MSMEG_4940	bacteriophage lysis protein		1.02	9.24E-01	0.90	1.54E-01	1.90	3.68E-02
MSMEG_4941	ATP synthase F0, C subunit	*atpE*	0.56	7.73E-03	1.09	2.36E-01	3.05	2.84E-02
MSMEG_4942	ATP synthase F0, A subunit	*atpB*	0.52	5.58E-03	0.96	5.49E-01	1.77	7.49E-02
MSMEG_4943	conserved hypothetical protein		0.59	1.17E-02	0.89	4.21E-01	2.40	1.88E-02
MSMEG_4944	hypothetical protein		0.63	2.61E-02	0.95	5.42E-01	2.50	1.27E-02
MSMEG_4947	glycosyl trasferase		0.36	6.82E-05	1.30	2.82E-01	1.36	1.64E-01
MSMEG_4948	Sua5/YciO/YrdC/YwlC family protein		0.61	3.81E-05	1.21	2.13E-01	2.00	1.64E-02
MSMEG_4949	modification methylase, HemK family protein		0.70	4.12E-02	1.37	2.20E-01	3.07	1.88E-01
MSMEG_4950	peptide chain release factor 1	*prfA*	0.56	8.16E-04	1.18	4.17E-01	1.49	1.72E-01
MSMEG_4951	ribosomal protein L31	*rpmE*	1.72	1.84E-02	1.07	6.58E-01	0.85	3.99E-01
MSMEG_4952	acyl-CoA synthase		1.19	4.19E-01	2.19	1.34E-01	0.97	5.92E-01
MSMEG_4953	putative transcriptional regulator		1.33	4.31E-02	0.88	2.17E-01	1.10	7.02E-01
MSMEG_4954	transcription termination factor Rho	*rho*	0.57	1.83E-04	1.43	1.70E-01	0.88	1.10E-01
MSMEG_4955	homoserine kinase	*thrB*	0.31	4.95E-06	1.37	1.96E-01	2.28	6.05E-04
MSMEG_4956	threonine synthase	*thrC*	0.29	7.02E-06	1.65	1.65E-01	0.90	5.54E-01
MSMEG_4957	homoserine dehydrogenase		0.34	2.64E-05	1.85	1.81E-01	1.43	1.38E-01
MSMEG_4958	diaminopimelate decarboxylase	*lysA*	0.24	4.53E-06	3.55	8.91E-02	1.93	2.24E-02
MSMEG_4959	arginyl-tRNA synthetase	*argS*	0.34	2.85E-05	3.69	9.66E-02	1.31	4.35E-03
MSMEG_4961	hypothetical protein		4.50	1.19E-02	2.84	1.04E-01	1.57	9.11E-02
MSMEG_4962	RemO protein		0.98	8.41E-01	1.08	2.49E-01	0.96	4.60E-01
MSMEG_4963	peptide synthetase ScpsB, putative		1.01	7.97E-01	1.09	1.12E-01	1.47	3.36E-01
MSMEG_4964	transcriptional regulator, TetR family protein		0.84	1.00E-01	0.96	3.39E-01	1.44	1.60E-01
MSMEG_4965	hypothetical protein		1.73	4.42E-03	0.94	5.32E-01	0.57	1.22E-03
MSMEG_4966	CalU12 protein		0.62	6.18E-04	1.02	8.57E-01	1.33	1.47E-01

MSMEG_4967	conserved hypothetical protein		0.57	4.13E-03	0.92	3.95E-01	2.19	5.61E-02
MSMEG_4968	two-component system sensor kinase		3.01	2.40E-03	0.54	1.50E-02	2.73	2.33E-01
MSMEG_4969	two-component system response regulator		2.26	1.31E-02	0.62	7.02E-03	2.37	2.23E-01
MSMEG_4970	hypothetical protein		3.18	3.80E-03	0.61	4.81E-02	1.77	3.31E-01
MSMEG_4971	oxidoreductase		1.14	7.02E-03	1.06	3.84E-01	1.20	1.08E-01
MSMEG_4972	acetyltransferase		1.32	1.71E-02	1.17	4.40E-01	1.26	2.79E-02
MSMEG_4973	glyoxalase/bleomycin resistance protein/dioxygenase		3.61	3.30E-03	0.81	6.77E-02	0.82	1.23E-01
MSMEG_4974	rrf2 family protein (putative transcriptional regulator)		0.86	3.75E-02	0.78	1.83E-02	1.23	9.31E-02
MSMEG_4975	flavin-nucleotide-binding protein		0.66	2.40E-03	1.00	9.61E-01	0.46	6.71E-03
MSMEG_4976	isochorismatase hydrolase		1.18	2.46E-01	0.95	3.24E-01	0.29	1.83E-04
MSMEG_4977	3'(2'),5'-bisphosphate nucleotidase, putative		0.59	4.31E-05	0.97	3.08E-01	1.40	5.85E-02
MSMEG_4978	sulfate adenylytransferase, large subunit/adenylylsulfate kinase	*cysNC*	0.44	3.02E-03	0.86	7.05E-02	1.09	2.16E-01
MSMEG_4979	sulfate adenylyltransferase, small subunit	*cysD*	0.57	8.86E-04	0.90	1.60E-01	1.61	8.83E-02
MSMEG_4980	major facilitator superfamily protein MFS_1		0.71	5.02E-02	0.91	3.38E-01	1.13	3.22E-01
MSMEG_4981	phosphotransferase enzyme family protein, putative		1.22	4.81E-02	1.44	2.34E-01	1.09	1.23E-01
MSMEG_4982	conserved hypothetical protein		1.45	2.00E-01	1.05	5.04E-01	1.01	7.89E-01
MSMEG_4983	transcriptional regulator, TetR family protein		0.87	1.77E-01	1.04	5.59E-01	1.05	2.95E-01
MSMEG_4984	conserved hypothetical protein, putative		1.38	5.51E-02	1.11	2.44E-01	0.90	1.82E-01
MSMEG_4985	carbonic anhydrase		1.46	8.80E-03	1.45	2.58E-01	1.88	2.19E-01
MSMEG_4986	glycosyl transferase, family protein 39		1.26	2.83E-01	1.15	3.26E-01	1.05	1.66E-01
MSMEG_4987	glycosyl transferase		0.98	7.82E-01	1.31	2.56E-01	1.28	5.28E-01
MSMEG_4988	integral membrane protein		1.05	7.30E-01	1.10	5.11E-01	0.96	2.65E-01
MSMEG_4989	sensor histidine kinase		1.10	1.61E-01	1.02	7.16E-01	3.32	1.03E-01
MSMEG_4990	DNA-binding response regulator		2.26	1.34E-03	0.73	2.09E-03	80.88	3.62E-01
MSMEG_4991	hypothetical protein		4.52	2.77E-04	0.75	1.54E-01	0.50	1.41E-03
MSMEG_4992	hypothetical protein		1.56	1.99E-03	0.54	4.67E-03	1.70	1.36E-01
MSMEG_4993	hypothetical protein		3.98	3.65E-03	1.04	7.95E-01	0.24	3.90E-05
MSMEG_4994	flagella membrane glycoprotein 1B, putative		1.00	9.62E-01	1.31	6.21E-01	0.91	4.60E-01
MSMEG_4995	oligopeptide transport system permease protein AppB		0.76	1.67E-02	0.86	1.36E-01	2.30	3.79E-02
MSMEG_4996	ABC transporter, permease protein OppC	*oppC*	0.44	7.32E-04	0.96	7.67E-01	3.18	4.22E-02
MSMEG_4997	ABC transporter, ATP-binding protein OppD	*oppD*	0.59	1.30E-02	1.12	1.24E-01	1.32	1.67E-01
MSMEG_4998	hydrolase, alpha/beta fold family protein		1.31	5.29E-02	1.00	9.97E-01	1.83	5.94E-02

MSMEG_4999	bacterial extracellular solute-binding protein, family protein 5	1.03	4.93E-01	0.87	2.06E-01	0.81	9.64E-02
MSMEG_5000	transcriptional regulator, LysR family protein	1.29	1.18E-01	0.92	5.98E-01	1.14	4.48E-01
MSMEG_5001	pesticide degrading monooxygenase	0.99	8.75E-01	1.59	1.63E-01	2.26	1.68E-02
MSMEG_5002	conserved hypothetical protein	0.84	8.92E-02	1.01	9.00E-01	1.97	8.90E-02
MSMEG_5003	O-methyltransferase, family protein 3	1.34	3.23E-03	0.88	2.37E-01	1.59	1.07E-01
MSMEG_5004	DNA repair exonuclease	0.72	2.23E-01	1.04	7.60E-01	2.74	3.38E-02
MSMEG_5005	LprC protein	0.83	9.16E-03	0.81	7.02E-02	1.20	4.73E-01
MSMEG_5006	phosphohistidine phosphatase	1.09	5.44E-01	0.83	7.54E-02	1.23	1.06E-01
MSMEG_5007	LprB protein	0.98	7.21E-01	0.96	4.35E-01	1.02	9.24E-01
MSMEG_5008	ABC transporter, ATP-binding protein	0.80	6.17E-02	0.90	1.33E-01	1.22	3.27E-01
MSMEG_5009	ABC transporter	0.72	3.78E-02	0.98	8.77E-01	1.03	8.25E-01
MSMEG_5010	conserved hypothetical protein	3.61	9.76E-03	0.66	1.48E-02	1.94	3.41E-03
MSMEG_5011	hypothetical protein	2.49	1.78E-03	0.90	9.21E-02	1.36	5.63E-02
MSMEG_5012	trans-sialidase, putative	0.97	7.23E-01	1.13	4.10E-01	1.00	9.99E-01
MSMEG_5013	conserved hypothetical protein	1.10	2.29E-01	0.87	2.45E-01	1.17	6.18E-02
MSMEG_5014	copper-translocating P-type ATPase	0.86	1.38E-01	0.79	1.55E-01	2.15	6.42E-02
MSMEG_5015	secreted protein	1.14	3.32E-01	0.97	8.54E-01	1.40	5.23E-01
MSMEG_5016	conserved domain protein	1.44	1.15E-03	0.77	7.56E-03	0.89	3.18E-01
MSMEG_5017	lipoprotein	0.85	7.12E-02	0.90	1.16E-01	0.84	4.61E-02
MSMEG_5018	Adenylate and Guanylate cyclase catalytic domain protein	1.00	9.41E-01	1.22	2.45E-01	2.19	9.70E-02
MSMEG_5019	regulatory protein	2.24	2.82E-02	1.32	9.62E-02	7.25	3.94E-01
MSMEG_5020	acetoin(diacetyl) reductase	0.88	1.61E-01	1.66	7.58E-02	2.26	3.46E-01
MSMEG_5021	alcohol dehydrogenase, zinc-containing	0.94	4.66E-01	1.77	1.02E-01	1.32	6.22E-01
MSMEG_5022	flavin-containing monooxygenase FMO	1.08	2.69E-01	1.45	6.84E-02	0.43	4.63E-02
MSMEG_5023	chitooligosaccharide deacetylase	0.82	7.27E-03	0.86	1.12E-02	1.05	8.76E-01
MSMEG_5024	T/U mismatch-specific DNA glycosylase	0.81	2.56E-02	1.38	2.91E-01	1.19	3.49E-01
MSMEG_5025	putative transcriptional regulator	1.19	1.14E-01	1.55	2.23E-01	1.51	1.64E-01
MSMEG_5026	conserved hypothetical protein	0.94	6.20E-01	1.06	4.55E-01	2.07	1.56E-01
MSMEG_5027	glyoxalase family protein	1.66	2.63E-02	0.86	3.63E-01	1.34	1.07E-01
MSMEG_5028	HIT family protein	1.81	5.75E-02	0.88	5.20E-01	1.19	2.84E-02
MSMEG_5029	alkanal monooxygenase alpha chain	3.40	1.65E-02	1.01	9.47E-01	0.71	1.98E-02
MSMEG_5030	conserved hypothetical protein TIGR00026	0.95	5.77E-01	0.87	1.26E-01	0.86	1.29E-01
MSMEG_5031	uracil-DNA glycosylase superfamily protein	2.06	7.38E-03	0.82	2.05E-01	1.07	6.02E-01
MSMEG_5032	putative transcriptional regulator	1.25	6.16E-02	1.37	8.51E-02	1.37	2.31E-01
MSMEG_5033	Tap protein; this gene contains a frame shift which is not the result of sequencing error	1.49	1.22E-01	1.22	1.29E-02	0.90	1.66E-01

MSMEG_5033	Tap protein; this gene contains a frame shift which is not the result of sequencing error		0.98	8.54E-01	0.93	6.51E-01	1.19	1.72E-01
MSMEG_5034	hypothetical protein		1.72	7.59E-02	0.86	2.61E-01	0.89	3.22E-01
MSMEG_5035	conserved hypothetical protein		1.38	2.62E-01	1.14	3.20E-01	1.14	4.05E-01
MSMEG_5036	rhomboid family protein		0.44	6.38E-04	3.68	3.33E-01	1.50	3.05E-01
MSMEG_5037	oxidoreductase, FAD-binding		0.80	5.57E-03	1.05	3.53E-01	1.13	1.69E-02
MSMEG_5038	putative cytochrome P450 123		1.26	6.53E-03	0.91	2.48E-01	0.98	9.44E-01
MSMEG_5039	Siderophore-interacting protein		1.11	4.49E-01	1.29	2.95E-01	16.74	3.43E-01
MSMEG_5040	transcriptional regulator, TetR family protein		1.05	9.04E-01	1.08	7.01E-01	0.96	5.44E-01
MSMEG_5041	probable acyltransferase		0.92	7.18E-01	1.04	6.80E-01	1.84	4.55E-02
MSMEG_5042	ATP-dependent rna helicase, dead/deah box family protein		1.07	1.47E-01	1.05	4.26E-01	2.05	7.54E-02
MSMEG_5043	LprE protein		0.95	8.28E-01	0.99	8.92E-01	1.51	1.77E-01
MSMEG_5044	ATPase		0.79	1.83E-01	0.88	1.72E-01	1.42	1.46E-01
MSMEG_5045	D-2-hydroxyglutarate dehydrogenase		0.84	1.97E-02	1.07	7.74E-01	1.36	1.22E-01
MSMEG_5046	drug transporter		1.15	7.75E-02	1.10	6.31E-01	1.87	2.61E-02
MSMEG_5047	drug resistance transporter Bcr/CflA subfamily protein		0.84	2.08E-01	1.57	1.33E-01	1.08	7.52E-01
MSMEG_5048	conserved hypothetical protein		2.10	4.40E-03	1.41	1.25E-01	6.34	2.89E-02
MSMEG_5049	2-oxoglutarate dehydrogenase, E1 component	*sucA*	0.76	6.41E-03	1.18	7.17E-02	1.51	3.40E-04
MSMEG_5050	methionine aminopeptidase, type I	*map*	2.34	5.34E-03	0.95	4.72E-01	0.22	2.67E-06
MSMEG_5051	major facilitator superfamily protein		0.41	1.26E-03	6.32	2.05E-01	11.65	8.07E-04
MSMEG_5052	DNA-binding protein		1.26	3.76E-02	0.64	5.02E-03	0.67	1.67E-03
MSMEG_5053	short chain alcohol dehydrogenase		1.24	1.14E-02	0.72	2.70E-02	1.09	3.17E-01
MSMEG_5054	ABC transporter, quaternary amine uptake transporter (QAT) family protein, substrate-binding protein		0.55	1.01E-03	1.95	9.40E-02	1.07	5.18E-01
MSMEG_5055	NAD-dependent malic enzyme		0.67	6.43E-03	1.19	4.65E-01	0.48	1.46E-03
MSMEG_5056	magnesium and cobalt transport protein CorA	*corA*	1.60	9.60E-02	0.93	6.62E-01	0.97	2.30E-01
MSMEG_5057	conserved hypothetical protein		0.69	1.14E-02	1.90	6.03E-02	0.67	5.41E-02
MSMEG_5058	ABC transporter, ATP-binding protein SugC		1.37	9.26E-03	1.24	3.45E-02	0.48	6.98E-03
MSMEG_5059	ABC transporter, permease protein SugB		1.90	2.46E-03	1.03	4.86E-01	0.42	5.87E-03
MSMEG_5060	ABC transporter, permease protein SugA		1.41	4.55E-04	1.13	1.15E-01	0.74	4.02E-01
MSMEG_5061	Bacterial extracellular solute-binding protein		2.06	2.65E-05	0.84	3.88E-02	0.39	8.75E-04
MSMEG_5062	conserved hypothetical protein		1.06	3.49E-01	1.86	1.16E-01	0.52	3.00E-04
MSMEG_5063	integral membrane protein		0.71	1.17E-02	0.88	3.25E-01	1.69	4.60E-02
MSMEG_5064	malyl-CoA lyase		0.55	4.62E-03	3.30	9.37E-02	1.39	3.58E-01
MSMEG_5065	Mg/Co/Ni transporter MgtE		0.74	3.42E-03	1.22	3.04E-01	1.66	1.70E-02

MSMEG_5066	integral membrane protein		0.70	2.89E-03	1.34	1.48E-01	1.01	9.20E-01
MSMEG_5067	secreted protein		0.89	2.85E-02	1.21	4.37E-01	0.34	3.12E-04
MSMEG_5068	Mrp protein		0.86	5.99E-03	1.12	3.03E-01	0.92	2.57E-01
MSMEG_5069	sec-independent translocase		0.90	6.40E-02	3.19	1.12E-01	2.58	3.71E-03
MSMEG_5070	Trypsin		1.10	1.84E-01	3.16	1.33E-01	2.86	1.41E-02
MSMEG_5071	conserved hypothetical protein		1.91	1.13E-03	2.12	1.58E-01	3.27	9.40E-02
MSMEG_5072	extracytoplasmic function alternative sigma factor		3.20	4.73E-03	0.59	2.88E-03	1.74	2.55E-03
MSMEG_5073	O-methyltransferase, family protein 3		0.89	1.70E-01	0.82	7.22E-02	2.41	1.08E-01
MSMEG_5074	probable transcriptional regulatory protein		0.98	7.85E-01	1.09	2.51E-01	1.21	6.47E-02
MSMEG_5075	ABC transporter ATP-binding protein		1.18	4.89E-02	1.29	1.90E-01	1.50	3.03E-02
MSMEG_5076	ABC transporter membrane-spanning protein		1.12	1.44E-01	1.09	2.53E-01	1.39	4.14E-03
MSMEG_5077	probable conserved integral membrane protein		1.27	9.45E-03	0.90	4.56E-01	2.15	1.61E-02
MSMEG_5078	glucose-1-phosphate adenylyltransferase	*glgC*	1.13	4.48E-01	1.32	1.61E-01	0.38	1.54E-05
MSMEG_5079	conserved hypothetical protein		0.79	1.62E-02	1.05	6.19E-01	1.41	2.48E-01
MSMEG_5080	glycogen synthase		1.01	8.15E-01	0.88	6.66E-02	1.26	3.27E-01
MSMEG_5081	conserved hypothetical protein		1.44	1.73E-02	0.69	2.61E-02	1.21	2.15E-01
MSMEG_5082	DNA-3-methyladenine glycosylase I	*tag*	0.68	1.60E-02	0.94	4.35E-03	1.48	5.29E-02
MSMEG_5083	conserved hypothetical protein		0.69	8.47E-03	1.05	5.70E-01	1.18	2.66E-01
MSMEG_5084	glycosyl transferase, group 2 family protein		0.47	2.10E-02	1.23	5.33E-01	0.74	1.64E-01
MSMEG_5085	dihydropteroate synthase	*folP*	1.05	6.63E-01	1.05	6.53E-01	1.42	9.74E-02
MSMEG_5086	very-long-chain acyl-CoA synthetase		0.56	3.52E-05	1.12	1.56E-01	1.95	1.25E-02
MSMEG_5087	conserved hypothetical protein		0.77	1.60E-02	0.82	1.09E-02	1.58	1.42E-01
MSMEG_5088	tetratricopeptide repeat family protein		0.90	5.67E-01	1.14	5.18E-01	1.00	9.98E-01
MSMEG_5089	conserved hypothetical protein		1.05	7.46E-01	1.51	1.72E-01	0.65	2.81E-03
MSMEG_5090	conserved hypothetical protein		0.84	6.36E-02	1.26	2.16E-01	0.67	9.25E-03
MSMEG_5091	hypothetical protein		0.80	3.46E-01	0.80	3.06E-02	1.16	9.24E-02
MSMEG_5092	hypothetical protein		0.80	1.25E-02	1.10	3.09E-01	0.85	1.96E-01
MSMEG_5095	This gene is disrupted by an IS1096 element.; ISMsm1, transposase orfB, truncation; identified by similarity to GP:16551202; match to protein family HMM PF00665		0.78	1.19E-01	1.07	6.82E-01	1.13	5.40E-01
MSMEG_5097	hypothetical protein		1.87	4.18E-01	0.81	9.67E-02	2.42	1.27E-01
MSMEG_5098	hypothetical protein		1.31	3.69E-01	1.25	1.50E-01	1.30	4.19E-01
MSMEG_5099	conserved hypothetical protein		0.64	8.52E-03	0.93	2.34E-01	1.18	4.66E-01
MSMEG_5100	pyruvate ferredoxin/flavodoxin oxidoreductase family protein		0.64	1.18E-03	1.34	5.49E-02	0.88	1.10E-01
MSMEG_5100	pyruvate ferredoxin/flavodoxin oxidoreductase family protein		0.82	8.80E-02	0.96	5.74E-01	0.98	8.34E-01

MSMEG_5101	hypothetical protein		1.29	8.05E-02	1.12	4.86E-01	0.85	3.83E-01
MSMEG_5102	ABC transporter ATP-binding protein		1.17	5.49E-01	1.44	4.98E-02	1.09	5.23E-01
MSMEG_5103	succinyl-diaminopimelate desuccinylase	*dapE*	0.98	8.47E-01	0.88	1.79E-01	0.90	1.98E-01
MSMEG_5104	tetrahydropicolinate succinylase		0.37	1.72E-05	1.11	5.72E-01	1.10	6.93E-01
MSMEG_5105	hypothetical protein		1.29	1.99E-01	1.30	6.71E-02	0.78	2.43E-01
MSMEG_5106	HNH endonuclease		1.29	1.06E-01	1.69	1.44E-01	1.44	4.59E-01
MSMEG_5107	secreted protein		3.33	1.72E-02	0.87	2.05E-01	1.29	1.24E-01
MSMEG_5108	conserved hypothetical protein		2.23	3.08E-03	1.27	4.15E-02	0.80	1.32E-01
MSMEG_5109	conserved hypothetical protein		5.23	7.38E-03	0.92	2.45E-01	1.00	9.64E-01
MSMEG_5110	sulfatase; this region contains one or more premature stops and/or frameshifts which are not the result of sequencing error		1.11	3.92E-01	1.24	2.02E-01	1.44	1.85E-01
MSMEG_5111	conserved hypothetical protein		2.19	1.55E-03	1.01	8.84E-01	0.31	4.50E-04
MSMEG_5112	hypothetical protein		1.59	3.14E-03	0.99	8.65E-01	0.94	8.86E-01
MSMEG_5113	conserved hypothetical protein; this region contains one or more premature stops and/or frameshifts which are not the result of sequencing error		0.65	1.59E-05	1.13	1.83E-01	1.86	1.65E-02
MSMEG_5114	acyl-CoA synthase		0.70	2.67E-03	1.06	6.10E-01	1.42	3.53E-02
MSMEG_5115	hypothetical protein		1.95	1.32E-02	0.78	5.52E-02	0.81	2.62E-01
MSMEG_5116	conserved hypothetical protein		2.16	1.95E-02	0.86	1.70E-01	1.11	9.92E-02
MSMEG_5117	proline dehydrogenase		4.15	2.82E-02	1.36	3.11E-01	3.47	2.48E-01
MSMEG_5118	nudix hydrolase		1.25	1.88E-02	0.83	5.16E-02	0.73	3.82E-03
MSMEG_5119	1-pyrroline-5-carboxylate dehydrogenase	*pruA*	8.69	1.38E-02	1.61	1.87E-01	3.19	2.64E-01
MSMEG_5120	conserved hypothetical protein		1.20	6.08E-02	1.03	7.31E-01	1.26	3.04E-01
MSMEG_5121	aminotransferase, classes I and II		0.59	3.37E-03	1.18	2.13E-01	3.56	4.57E-02
MSMEG_5122	ferredoxin		0.71	2.91E-03	1.13	1.30E-01	2.56	1.26E-04
MSMEG_5123	acyl-CoA dehydrogenase, C- domain protein; this gene contains a frame shift which is not the result of sequencing error; identified by match to protein family HMM PF08028		1.09	4.29E-01	1.51	1.92E-01	0.81	1.03E-01
MSMEG_5124	2,4-dienoyl-coA reductase		1.40	5.12E-01	0.91	3.80E-01	1.42	5.26E-02
MSMEG_5125	YceI like family protein		2.04	6.53E-02	4.89	1.13E-01	1.13	3.24E-01
MSMEG_5126	FO synthase		0.65	2.48E-03	1.24	2.81E-01	1.40	1.20E-02
MSMEG_5127	hypothetical protein		1.07	2.34E-01	1.19	3.03E-01	7.32	3.99E-01
MSMEG_5128	conserved hypothetical protein		0.54	2.53E-03	0.73	2.50E-02	0.92	3.47E-01
MSMEG_5129	N-Acetyl-1-D-myo-Inositÿl-2-Amino-2-Deoxy-alpha- D-Glucopyranoside Deacetylase MshB		0.47	2.88E-04	0.82	1.54E-01	1.69	3.79E-02

MSMEG_5130	bacterial extracellular solute-binding protein, family protein 5		0.60	4.22E-03	1.15	4.13E-01	2.65	4.37E-02
MSMEG_5131	hypothetical protein		4.17	2.50E-04	0.97	8.90E-01	0.61	7.40E-02
MSMEG_5132	GTP-binding protein TypA/BipA	*typA*	0.52	3.01E-05	0.85	4.27E-02	1.78	1.39E-03
MSMEG_5133	hypothetical protein		0.83	9.00E-02	0.80	4.50E-03	1.05	5.02E-01
MSMEG_5134	hypothetical protein		0.86	2.90E-01	0.81	3.61E-02	1.05	6.25E-01
MSMEG_5135	hypothetical protein		5.06	3.36E-04	0.89	2.15E-01	1.38	5.62E-02
MSMEG_5136	helix-turn-helix motif		3.24	1.09E-02	5.81	1.86E-03	2.98	2.25E-02
MSMEG_5137	respiratory nitrate reductase, gamma subunit	*narI*	1.82	4.19E-03	0.94	3.84E-01	0.74	1.83E-01
MSMEG_5138	nitrate reductase molybdenum cofactor assembly chaperone	*narJ*	1.39	2.27E-01	1.59	1.88E-01	0.86	1.85E-01
MSMEG_5139	nitrate reductase, beta subunit	*narH*	1.48	1.84E-01	1.22	4.88E-02	0.86	2.92E-01
MSMEG_5140	nitrate reductase, alpha subunit		1.87	8.47E-02	1.14	1.89E-01	0.88	1.66E-01
MSMEG_5141	nitrate/nitrite transporter protein		3.99	2.52E-01	1.25	2.46E-01	0.88	9.03E-02
MSMEG_5142	beta-glucosidase A		2.30	2.00E-02	0.87	1.85E-01	0.97	7.37E-01
MSMEG_5143	HTH-type transcriptional regulator DegA		6.87	3.51E-01	0.85	6.55E-02	0.87	1.84E-01
MSMEG_5144	xylosidase/arabinosidase		1.36	1.47E-02	0.99	9.64E-01	3.73	3.69E-01
MSMEG_5145	extracellular solute-binding protein, family protein 1		0.89	3.22E-01	1.03	8.24E-01	0.44	6.64E-03
MSMEG_5146	binding protein dependent transport protein		0.96	8.67E-01	0.81	1.34E-02	0.85	4.47E-02
MSMEG_5147	sugar ABC-transporter integral membrane protein		0.74	9.63E-02	0.95	5.05E-01	0.85	8.16E-02
MSMEG_5148	CTP pyrophosphohydrolase		0.57	4.13E-02	1.19	5.09E-01	1.00	8.49E-01
MSMEG_5149	conserved transmembrane protein		0.90	2.03E-02	0.94	3.80E-01	3.32	4.41E-02
MSMEG_5150	putative pterin-4-alpha-carbinolamine		1.22	1.41E-02	1.05	4.83E-01	0.63	5.08E-02
MSMEG_5152	hypothetical protein		2.54	3.34E-02	0.50	3.13E-04	1.84	2.90E-01
MSMEG_5152	hypothetical protein		1.60	1.21E-01	0.62	3.62E-03	0.80	5.17E-01
MSMEG_5153	conserved hypothetical protein		1.74	8.38E-02	0.68	8.62E-03	1.09	7.49E-01
MSMEG_5154	conserved hypothetical protein		1.86	1.33E-03	1.24	1.77E-01	0.99	9.51E-01
MSMEG_5155	nitroreductase		2.06	9.19E-03	0.86	1.92E-01	0.83	1.71E-01
MSMEG_5156	base excision DNA repair protein, HhH-GPD family protein		1.04	6.35E-01	1.06	5.41E-01	1.47	1.31E-02
MSMEG_5157	hypothetical protein		1.30	1.78E-01	1.15	2.69E-01	0.72	6.50E-02
MSMEG_5158	sensor histidine kinase		0.93	2.67E-01	1.12	1.42E-01	1.07	2.59E-01
MSMEG_5159	DNA-binding response regulator, putative		0.81	5.42E-02	0.97	7.70E-01	1.13	4.00E-01
MSMEG_5160	putative integral membrane protein		0.84	2.81E-01	0.89	4.13E-01	0.99	8.47E-01
MSMEG_5161	transmembrane transport protein		1.11	2.28E-01	1.19	2.72E-01	1.39	3.85E-02
MSMEG_5162	cinnamoyl ester hydrolase		1.17	3.80E-01	0.76	4.65E-02	0.99	3.54E-01

MSMEG_5163	ABC proline/glycine betaine family protein transporter, periplasmic ligand binding protein	1.08	2.60E-02	1.83	4.17E-02	0.99	8.77E-01
MSMEG_5164	zinc-binding alcohol dehydrogenase family protein	4.20	2.78E-05	0.64	1.83E-02	1.04	7.70E-01
MSMEG_5165	integral membrane protein	4.98	4.16E-03	0.75	2.08E-01	0.49	1.53E-02
MSMEG_5166	Na+/solute symporter	4.65	1.77E-03	0.92	7.57E-01	0.38	9.30E-04
MSMEG_5167	major facilitator superfamily protein MFS_1	2.60	9.87E-04	0.92	5.80E-01	0.92	2.62E-01
MSMEG_5168	conserved hypothetical protein	1.07	4.58E-01	0.99	6.21E-01	1.11	2.17E-01
MSMEG_5169	conserved hypothetical protein	1.88	2.37E-03	0.78	3.13E-02	0.81	3.50E-02
MSMEG_5170	pyridoxamine 5'-phosphate oxidase family	2.37	8.59E-05	0.72	4.32E-02	0.52	1.78E-05
MSMEG_5171	conserved hypothetical protein	0.97	5.14E-01	0.81	4.13E-02	0.99	9.32E-01
MSMEG_5172	conserved hypothetical protein	1.75	8.26E-04	0.90	2.10E-01	0.79	2.17E-01
MSMEG_5173	O-methyltransferase, putative	1.33	3.68E-02	0.75	2.74E-02	0.94	8.02E-01
MSMEG_5174	regulatory protein GntR, HTH	3.51	5.71E-04	0.92	3.65E-01	1.35	1.79E-01
MSMEG_5175	NAD-dependent deacetylase	1.35	4.78E-02	0.68	9.73E-03	1.19	3.54E-01
MSMEG_5176	methyltransferase type 11	2.18	5.53E-03	0.82	8.54E-02	0.62	2.01E-02
MSMEG_5177	regulatory protein, TetR	1.35	4.47E-02	0.98	8.60E-01	0.94	6.93E-01
MSMEG_5178	integral membrane protein	1.39	1.13E-01	0.93	6.90E-01	1.04	2.09E-01
MSMEG_5179	DoxX subfamily protein, putative	2.20	4.17E-02	1.29	6.63E-04	1.24	4.66E-01
MSMEG_5180	conserved hypothetical protein	1.82	1.90E-01	1.28	1.04E-02	0.63	1.89E-02
MSMEG_5182	2-Nitropropane dioxygenase	1.03	7.91E-01	1.02	7.18E-01	1.59	7.14E-03
MSMEG_5183	3-Hydroxyacyl-CoA dehydrogenase	1.22	9.47E-02	0.78	4.53E-02	1.37	1.32E-02
MSMEG_5184	alpha-methylacyl-CoA racemase, putative	1.40	5.67E-02	0.88	5.89E-01	1.80	1.70E-02
MSMEG_5185	enoyl-CoA hydratase PaaB	1.81	8.44E-03	0.81	1.55E-01	1.06	3.55E-01
MSMEG_5186	HicB family protein	1.38	1.15E-01	0.78	1.02E-01	1.84	3.12E-01
MSMEG_5187	tetracycline-resistance determinant TetV	1.19	2.40E-01	1.13	6.61E-01	1.11	4.18E-01
MSMEG_5188	caax amino protease family protein	1.21	1.76E-01	1.07	4.97E-01	1.34	3.06E-01
MSMEG_5189	oxidoreductase	1.28	2.76E-01	1.02	8.77E-01	1.38	9.32E-02
MSMEG_5190	TetR-family protein transcriptional regulator	1.31	7.17E-02	0.92	4.42E-01	1.04	6.51E-01
MSMEG_5191	siderophore binding protein	0.43	1.97E-05	0.91	3.22E-01	1.65	1.64E-01
MSMEG_5192	aldo/keto reductase	0.89	4.98E-01	1.06	6.24E-01	1.03	2.93E-01
MSMEG_5193	transcriptional regulator, AraC family protein	0.66	5.68E-04	0.91	4.05E-01	1.03	3.49E-01
MSMEG_5194	integral membrane protein	0.89	2.33E-01	0.98	6.30E-01	1.27	3.23E-01
MSMEG_5195	hypothetical protein	0.79	9.50E-02	0.93	5.61E-01	0.97	5.84E-01
MSMEG_5196	fasciclin domain protein	0.44	5.10E-05	0.99	9.48E-01	0.61	1.46E-02
MSMEG_5197	long-chain specific acyl-CoA dehydrogenase	1.67	1.53E-03	1.02	7.21E-01	0.97	7.98E-01
MSMEG_5198	carnitinyl-CoA dehydratase	1.69	2.08E-03	0.95	5.18E-01	1.01	9.45E-01
MSMEG_5199	putative acyl-CoA dehydrogenase	1.87	2.76E-03	0.98	8.59E-01	1.18	3.33E-01

MSMEG_5200	hypothetical protein		0.95	4.72E-01	1.01	8.50E-01	1.00	9.20E-01
MSMEG_5201	regulatory protein GntR, HTH		1.00	9.94E-01	0.79	1.16E-02	1.38	1.53E-01
MSMEG_5202	conserved membrane protein		1.17	5.49E-01	0.97	8.24E-01	0.76	1.34E-01
MSMEG_5203	DoxX subfamily protein, putative		1.13	5.30E-02	0.94	6.53E-01	0.97	7.68E-01
MSMEG_5204	oxidoreductase, short chain dehydrogenase/reductase family protein		1.77	8.22E-03	1.11	4.89E-01	0.98	2.33E-01
MSMEG_5205	conserved hypothetical protein		0.69	9.46E-02	1.26	7.23E-02	0.98	1.52E-01
MSMEG_5206	regulatory protein		1.21	1.52E-01	1.25	1.13E-01	1.46	3.25E-01
MSMEG_5207	conserved hypothetical protein		0.52	1.32E-03	0.98	8.22E-01	0.88	1.14E-01
MSMEG_5208	conserved hypothetical protein		0.67	6.49E-04	1.11	1.65E-01	0.97	3.35E-01
MSMEG_5209	hydrolase, alpha/beta fold family protein		0.50	4.23E-03	1.13	1.27E-01	3.87	1.81E-02
MSMEG_5210	hydrolase		0.90	2.92E-01	1.02	8.45E-01	0.94	6.31E-01
MSMEG_5211	aminotransferase class-III		0.42	2.84E-05	0.91	7.93E-02	2.26	7.39E-02
MSMEG_5212	conserved hypothetical protein		1.64	1.68E-02	1.21	4.09E-01	0.96	4.65E-01
MSMEG_5213	conserved hypothetical protein		0.78	1.83E-02	1.17	3.65E-01	1.03	8.22E-01
MSMEG_5214	RNA polymerase sigma-70 factor		0.81	1.73E-01	0.92	5.81E-01	1.13	3.22E-01
MSMEG_5215	conserved hypothetical protein		1.17	3.41E-03	0.85	1.18E-01	1.20	6.32E-02
MSMEG_5216	glyoxalase family protein		2.28	3.86E-02	0.79	1.68E-01	1.52	2.31E-01
MSMEG_5217	hypothetical protein		3.30	5.56E-03	0.93	4.67E-01	2.00	1.65E-02
MSMEG_5218	conserved hypothetical protein		2.35	6.90E-03	1.00	9.75E-01	0.87	1.10E-01
MSMEG_5219	riboflavin biosynthesis protein RibD C- domain protein		1.16	6.99E-02	1.02	8.62E-01	1.34	9.08E-02
MSMEG_5220	esterase/lipase/thioesterase		0.80	4.94E-03	0.86	4.23E-03	0.97	7.37E-01
MSMEG_5221	GCN5-related N-acetyltransferase		0.46	4.09E-04	0.95	5.40E-01	1.27	1.27E-01
MSMEG_5222	GTP-binding protein YchF	*ychF*	0.46	2.37E-04	0.93	1.25E-01	1.80	1.16E-01
MSMEG_5223	conserved hypothetical protein		0.59	3.03E-03	1.06	3.33E-01	1.17	2.88E-01
MSMEG_5224	4-hydroxy-3-methylbut-2-enyl diphosphate reductase	*ispH*	1.50	4.24E-03	0.84	6.50E-02	1.25	1.67E-01
MSMEG_5225	conserved hypothetical protein		0.82	4.02E-02	0.92	2.63E-01	0.81	3.14E-01
MSMEG_5226	exodeoxyribonuclease VII, large subunit	*xseA*	0.44	4.70E-05	0.97	6.08E-01	1.40	2.91E-02
MSMEG_5227	exodeoxyribonuclease VII, small subunit	*xseB*	0.50	7.14E-06	1.00	9.77E-01	1.07	5.92E-01
MSMEG_5228	3-beta hydroxysteroid dehydrogenase/isomerase family protein		0.26	1.42E-05	1.52	4.24E-02	1.13	4.45E-01
MSMEG_5228	3-beta hydroxysteroid dehydrogenase/isomerase family protein		0.39	6.85E-05	1.18	1.94E-01	0.99	9.59E-01
MSMEG_5229	hypothetical protein		3.41	4.43E-02	0.66	1.93E-02	1.97	3.28E-02
MSMEG_5230	conserved hypothetical protein		8.48	1.24E-03	0.76	2.07E-01	0.93	6.27E-01
MSMEG_5231	hypothetical protein		9.50	1.93E-03	0.63	5.19E-02	0.41	9.67E-03

MSMEG_5232	hypothetical protein		1.09	4.21E-01	1.42	7.31E-02	1.29	5.35E-01
MSMEG_5233	RmlD substrate binding domain superfamily protein		0.72	1.57E-01	1.12	2.52E-01	0.70	8.46E-02
MSMEG_5234	short-chain dehydrogenase/reductase SDR		0.84	4.07E-01	1.58	4.48E-02	0.48	5.04E-03
MSMEG_5235	short chain dehydrogenase family protein		1.00	9.82E-01	1.05	5.24E-01	1.21	6.67E-01
MSMEG_5236	dienelactone hydrolase family protein		1.00	9.69E-01	0.82	1.23E-02	1.64	2.46E-02
MSMEG_5237	conserved hypothetical protein		1.27	1.82E-01	1.26	2.65E-01	1.17	5.87E-01
MSMEG_5238	conserved hypothetical protein		0.98	6.02E-01	0.89	8.58E-02	1.30	2.73E-01
MSMEG_5239	fructose-1,6-bisphosphatase, class II	*glpX*	0.97	6.86E-01	0.97	4.79E-01	0.70	1.57E-02
MSMEG_5240	fumarate hydratase class II		0.92	2.10E-01	0.74	1.96E-02	0.56	8.25E-03
MSMEG_5241	GAF family protein		1.49	1.42E-02	3.21	4.04E-03	4.10	2.27E-03
MSMEG_5242	acyltransferase, ws/dgat/mgat subfamily protein		1.41	1.52E-01	17.47	2.73E-02	24.46	1.23E-02
MSMEG_5243	helix-turn-helix motif		1.06	7.06E-01	19.21	1.83E-02	12.08	1.59E-03
MSMEG_5244	two component transcriptional regulatory protein devr		1.95	3.46E-03	3.33	5.41E-04	7.78	4.35E-04
MSMEG_5245	universal stress protein family protein		1.92	4.21E-02	8.81	1.06E-04	13.20	4.91E-03
MSMEG_5246	conserved hypothetical protein		1.39	3.57E-01	10.25	7.30E-03	17.70	1.10E-02
MSMEG_5247	PhoH family protein		1.05	6.92E-01	1.04	4.03E-01	2.19	5.74E-03
MSMEG_5248	acyl-[ACP] desaturase		0.31	2.63E-06	1.38	2.13E-02	19.56	1.24E-02
MSMEG_5249	serine hydroxymethyltransferase	*glyA*	0.58	9.55E-04	1.05	3.23E-01	1.40	9.23E-02
MSMEG_5250	CalR9 protein		0.81	6.55E-03	1.08	4.28E-01	1.08	7.61E-01
MSMEG_5251	hypothetical protein		1.88	1.16E-02	1.29	1.55E-01	1.10	7.46E-01
MSMEG_5252	pantothenate kinase	*coaA*	0.51	2.13E-03	0.94	5.58E-01	1.83	9.84E-02
MSMEG_5253	conserved hypothetical protein		2.43	7.01E-02	1.45	9.29E-02	1.47	3.80E-01
MSMEG_5254	conserved hypothetical protein		1.30	2.29E-01	1.30	4.89E-02	1.15	4.51E-01
MSMEG_5255	conserved hypothetical protein		2.56	9.45E-02	0.90	2.57E-01	1.72	1.06E-01
MSMEG_5256	undecaprenyl diphosphate synthase	*uppS*	1.19	2.74E-03	1.18	4.96E-02	2.29	1.57E-01
MSMEG_5257	channel protein, hemolysin III family protein		1.36	3.79E-04	0.79	3.70E-02	2.24	4.59E-03
MSMEG_5258	steroid delta-isomerase		0.77	1.55E-02	1.04	4.09E-01	0.96	8.08E-01
MSMEG_5259	conserved hypothetical protein		0.82	2.34E-02	1.09	5.93E-01	1.22	2.56E-01
MSMEG_5260	conserved hypothetical protein		0.53	5.58E-05	1.19	1.68E-01	1.61	3.40E-02
MSMEG_5261	mycothiol conjugate amidase Mca		0.46	7.09E-05	1.33	1.56E-01	1.31	6.72E-04
MSMEG_5262	conserved hypothetical protein		0.60	5.80E-04	0.83	6.15E-02	1.32	4.55E-01
MSMEG_5263	transcription elongation factor GreA		1.34	1.55E-02	0.64	1.36E-02	0.61	3.88E-03
MSMEG_5264	conserved hypothetical protein		0.43	5.28E-06	0.79	2.84E-01	1.28	6.44E-02
MSMEG_5264	conserved hypothetical protein		0.36	6.46E-05	1.00	9.89E-01	1.02	8.53E-01
MSMEG_5265	putative cystathionine gamma-synthase		0.40	2.70E-06	0.89	2.00E-01	1.45	1.24E-01
MSMEG_5266	RDD family protein		1.23	5.17E-02	1.03	6.73E-01	0.59	3.23E-03

MSMEG_5267	glutaryl-CoA dehydrogenase		2.29	8.99E-03	0.88	1.41E-01	1.19	2.28E-01
MSMEG_5268	conserved hypothetical protein		0.73	5.26E-03	1.17	2.58E-01	0.66	2.69E-02
MSMEG_5269	hypothetical protein		1.10	3.03E-01	0.90	2.63E-01	1.04	7.25E-01
MSMEG_5270	cystathionine beta-synthase		1.01	9.28E-01	1.79	1.61E-01	1.69	5.89E-02
MSMEG_5271	esterase		6.23	1.01E-03	1.15	2.55E-01	0.26	2.17E-04
MSMEG_5272	conserved exported protein		2.51	2.76E-03	0.74	9.39E-03	1.74	2.50E-02
MSMEG_5273	beta-ketoadipyl CoA thiolase		0.48	1.67E-04	0.78	4.95E-02	1.67	4.87E-02
MSMEG_5274	phosphoribosylglycinamide formyltransferase 2	*purT*	0.52	2.73E-04	0.93	5.96E-01	1.13	4.66E-01
MSMEG_5275	permease of the major facilitator superfamily protein		1.88	5.85E-03	1.17	9.21E-03	0.71	1.30E-04
MSMEG_5276	enoyl-CoA hydratase		0.38	7.97E-04	1.18	1.31E-01	1.37	1.65E-01
MSMEG_5277	probable enoyl-CoA hydratase		0.74	6.09E-02	1.25	2.25E-01	1.59	5.46E-02
MSMEG_5278	conserved membrane-spanning protein		1.00	9.83E-01	1.02	4.84E-01	1.02	3.20E-01
MSMEG_5279	conserved hypothetical protein		1.66	4.87E-01	1.42	3.52E-02	2.42	8.30E-02
MSMEG_5280	cysteine dioxygenase type I superfamily protein		0.75	7.31E-02	1.71	1.05E-01	5.43	9.51E-03
MSMEG_5281	LpqV protein		0.88	8.51E-02	0.91	8.15E-02	0.73	3.51E-03
MSMEG_5282	transcriptional regulator		1.55	3.40E-02	1.16	2.84E-01	1.15	5.15E-01
MSMEG_5283	hydrolase, CocE/NonD family protein		0.82	5.63E-02	1.33	4.10E-01	1.11	5.12E-01
MSMEG_5284	patatin		1.11	2.56E-01	1.08	2.92E-01	1.11	3.13E-01
MSMEG_5285	phospholipase, patatin family protein		0.73	2.01E-03	1.11	2.86E-01	1.63	3.97E-02
MSMEG_5286	dihydrodipicolinate reductase, N-terminus domain protein	*dapB*	1.24	1.24E-01	1.12	3.67E-01	0.98	6.63E-01
MSMEG_5287	dehydrogenase		1.36	1.21E-01	0.80	4.82E-05	1.37	3.29E-02
MSMEG_5288	putative serine/threonine protein kinase		1.18	2.37E-01	2.53	2.04E-01	1.01	5.76E-01
MSMEG_5289	hypothetical protein		0.96	6.68E-01	0.90	1.20E-01	1.16	2.03E-01
MSMEG_5290	hypothetical protein		1.63	2.66E-01	0.99	8.66E-01	1.27	3.88E-01
MSMEG_5291	acyl-CoA synthase		0.94	4.04E-01	1.66	9.21E-02	0.49	2.48E-03
MSMEG_5292	hypothetical protein		1.29	4.32E-04	1.41	4.15E-02	1.02	8.93E-01
MSMEG_5293	hypothetical protein		1.15	2.26E-02	1.38	1.02E-01	1.13	2.72E-01
MSMEG_5294	hypothetical protein		0.78	2.95E-01	2.38	4.37E-01	1.11	3.15E-01
MSMEG_5295	peroxisomal-coenzyme A synthetase		1.37	7.33E-02	6.75	1.03E-01	1.01	9.51E-01
MSMEG_5296	hypothetical protein		1.07	5.20E-01	2.45	2.41E-01	1.46	2.00E-01
MSMEG_5297	oxalyl-CoA decarboxylase		1.21	1.91E-01	2.04	1.21E-01	2.24	2.18E-01
MSMEG_5298	hypothetical protein		7.00	4.21E-03	1.26	5.34E-01	2.86	3.84E-03
MSMEG_5299	hypothetical protein		2.84	3.58E-01	1.20	1.59E-01	1.03	7.13E-01
MSMEG_5300	short-chain type dehydrogenase/reductase		1.78	1.63E-02	6.40	1.32E-01	1.13	5.34E-01
MSMEG_5301	transcriptional regulator		1.05	4.35E-01	1.27	1.57E-01	1.06	6.01E-01
MSMEG_5302	aerobic C4-dicarboxylate transport protein		11.50	5.64E-03	1.03	8.26E-01	0.13	4.17E-07

MSMEG_5303	sodium/proline symporter	*putP*	1.88	5.68E-03	1.23	1.21E-01	0.21	4.09E-05
MSMEG_5304	two-component sensor kinase		1.97	1.13E-02	1.21	1.03E-01	0.89	2.10E-01
MSMEG_5305	choline dehydrogenase		1.10	4.27E-01	1.03	7.69E-01	1.34	4.57E-01
MSMEG_5306	two-component system response regulator		1.53	6.01E-02	1.00	9.27E-01	1.04	4.33E-01
MSMEG_5307	TetR-family protein transcriptional regulator		1.36	5.61E-02	0.96	7.08E-01	1.08	6.09E-01
MSMEG_5308	conserved hypothetical protein		1.77	1.17E-02	0.67	6.54E-03	9.30	3.39E-02
MSMEG_5309	conserved hypothetical protein		1.13	5.00E-01	1.16	1.71E-01	2.88	2.28E-01
MSMEG_5310	SAM-dependent methyltransferase		2.30	7.63E-03	0.89	1.31E-01	1.35	1.73E-02
MSMEG_5311	conserved hypothetical protein		0.92	5.28E-01	1.46	1.24E-01	1.02	9.17E-01
MSMEG_5312	ABC-type multidrug transport system ATPase component		0.56	3.33E-04	1.20	2.85E-01	1.23	3.11E-01
MSMEG_5313	hypothetical protein		1.13	3.23E-01	1.36	4.88E-02	1.44	4.99E-01
MSMEG_5314	conserved hypothetical protein		1.01	9.61E-01	1.36	1.01E-01	1.10	4.71E-01
MSMEG_5315	gp35 protein		2.87	1.62E-02	0.68	1.46E-02	1.41	6.71E-02
MSMEG_5316	glutamine ABC transporter, ATP-binding protein		1.19	3.07E-01	1.01	8.89E-01	1.20	1.82E-01
MSMEG_5317	conserved hypothetical protein		1.25	1.21E-01	0.94	1.21E-01	0.84	5.37E-01
MSMEG_5318	glutamine ABC transporter, permease/substrate-binding protein		1.10	6.07E-01	1.03	8.71E-01	0.69	1.05E-01
MSMEG_5319	alkylhydroperoxidase AhpD core		2.70	2.50E-02	1.19	3.19E-01	1.07	2.48E-01
MSMEG_5320	hypothetical protein		0.94	9.77E-02	0.96	5.57E-01	0.93	2.49E-02
MSMEG_5321	hypothetical protein		1.14	4.72E-01	1.42	1.63E-01	1.13	4.16E-01
MSMEG_5322	hypothetical protein		2.14	5.70E-02	0.88	4.16E-01	1.02	3.59E-01
MSMEG_5323	conserved hypothetical protein		5.19	3.82E-02	2.95	1.26E-01	0.31	3.99E-03
MSMEG_5324	conserved hypothetical protein		1.08	6.12E-01	1.08	5.05E-01	0.92	3.57E-01
MSMEG_5325	hypothetical protein		0.57	1.57E-04	0.85	8.54E-02	1.06	5.70E-01
MSMEG_5326	hypothetical protein		1.11	4.49E-01	1.11	4.20E-01	1.37	2.28E-01
MSMEG_5327	hypothetical protein		25.37	6.18E-02	0.27	6.21E-03	0.87	5.86E-01
MSMEG_5328	conserved hypothetical protein		1.95	8.36E-04	0.97	7.88E-01	1.15	7.62E-01
MSMEG_5329	conserved hypothetical protein		0.75	4.77E-02	1.30	8.30E-03	1.14	4.17E-01
MSMEG_5330	SIS domain protein		0.70	1.16E-02	1.17	2.64E-01	0.90	1.49E-01
MSMEG_5331	UDP-glucoronosyl and UDP-glucosyl transferase family; this gene contains a frame shift which is not the result of sequencing error; identified by match to protein family HMM PF00201; match to protein family HMM PF04101		0.51	1.89E-02	1.13	6.25E-02	0.71	6.22E-02

MSMEG_5331	UDP-glucoronosyl and UDP-glucosyl transferase family; this gene contains a frame shift which is not the result of sequencing error; identified by match to protein family HMM PF00201; match to protein family HMM PF04101		0.65	3.52E-02	1.03	4.91E-01	0.93	2.14E-01
MSMEG_5332	transcriptional regulator, TetR family protein		0.70	1.57E-01	0.95	5.37E-01	1.36	3.62E-01
MSMEG_5333	hypothetical protein		1.17	4.56E-01	0.98	5.09E-01	0.74	6.16E-02
MSMEG_5334	conserved hypothetical protein		1.55	9.18E-02	0.95	7.39E-01	1.22	4.02E-01
MSMEG_5335	formamidase		1.04	7.49E-01	1.18	9.97E-02	0.81	1.38E-01
MSMEG_5336	amidate substrates transporter protein		1.22	3.05E-01	1.43	1.95E-01	0.93	2.48E-01
MSMEG_5337	putative regulatory protein, FmdB family; this gene contains a frame shift which is not the result of sequencing error; identified by match to protein family HMM TIGR02605		1.03	7.97E-01	1.25	1.82E-01	1.04	4.58E-01
MSMEG_5338	regulatory protein, MarR		1.38	1.85E-05	0.83	2.20E-02	0.97	7.23E-01
MSMEG_5339	nitrile hydratase regulator1		10.07	1.17E-03	0.92	4.00E-01	0.92	6.38E-01
MSMEG_5340	TetR-family protein transcriptional regulator		0.89	2.33E-02	1.23	1.50E-02	1.49	1.89E-01
MSMEG_5341	dipeptidyl aminopeptidase/acylaminoacyl peptidase		1.43	4.42E-03	1.17	1.44E-01	0.90	3.27E-01
MSMEG_5342	conserved hypothetical protein		1.68	8.77E-03	1.65	8.85E-03	0.76	8.76E-02
MSMEG_5343	conserved hypothetical protein		1.25	3.52E-01	1.53	7.30E-02	2.87	3.03E-01
MSMEG_5344	hypothetical protein		4.70	1.94E-03	0.75	4.79E-02	19.60	5.45E-02
MSMEG_5345	glycosyl hydrolases family protein 16		0.78	4.62E-02	0.90	7.50E-02	1.01	9.44E-01
MSMEG_5346	TetR-family protein transcriptional regulator		1.36	1.56E-03	1.69	3.76E-02	0.87	1.80E-01
MSMEG_5347	transporter, major facilitator family protein		1.44	4.26E-01	1.19	2.65E-01	1.62	2.22E-01
MSMEG_5348	medium-chain fatty acid-CoA ligase		1.32	8.89E-02	1.06	6.74E-01	1.00	9.86E-01
MSMEG_5349	conserved hypothetical protein, putative		0.78	2.99E-02	1.10	5.80E-01	0.88	1.78E-01
MSMEG_5350	PPE family protein, putative		1.10	6.02E-01	1.24	1.73E-01	1.01	8.67E-01
MSMEG_5351	hypothetical protein		1.35	4.78E-02	1.12	5.93E-02	0.99	8.59E-01
MSMEG_5352	conserved hypothetical protein		2.95	1.28E-03	0.95	6.02E-01	1.51	7.65E-02
MSMEG_5353	putative methyltransferase		1.00	9.93E-01	1.30	1.44E-01	1.23	2.30E-01
MSMEG_5354	O-methyltransferase, family protein 2		0.69	3.30E-02	1.00	9.45E-01	0.95	6.21E-01
MSMEG_5355	hypothetical protein		1.80	7.94E-02	0.70	8.09E-02	5.95	2.38E-02
MSMEG_5356	hypothetical protein		0.98	8.62E-01	0.98	1.99E-01	1.22	3.61E-01
MSMEG_5357	conserved hypothetical protein		1.23	2.74E-01	1.39	1.53E-01	1.03	1.27E-01
MSMEG_5358	acetamidase/Formamidase family protein		0.29	1.37E-03	1.74	2.83E-01	0.57	4.57E-02
MSMEG_5359	cyanate hydratase	*cynS*	1.30	7.37E-02	0.92	1.21E-01	0.36	1.40E-03
MSMEG_5360	formate/nitrate transporter		0.79	1.61E-01	1.90	1.86E-01	1.08	3.33E-01

MSMEG_5361	hypothetical protein		1.48	2.65E-01	1.47	1.56E-01	0.98	6.43E-01
MSMEG_5362	hypothetical protein		0.98	9.24E-01	1.24	3.35E-01	1.85	2.24E-01
MSMEG_5363	hypothetical protein		0.75	1.68E-01	0.89	4.28E-01	1.90	2.13E-01
MSMEG_5364	amidohydrolase 2		1.30	5.22E-02	1.37	4.17E-01	1.85	3.41E-01
MSMEG_5365	RNA polymerase sigma-70 factor		1.18	5.34E-01	1.44	9.91E-02	1.00	9.84E-01
MSMEG_5366	conserved hypothetical protein		0.98	8.75E-01	1.07	3.82E-01	1.17	4.69E-01
MSMEG_5367	conserved hypothetical protein; this gene contains a frame shift which is not the result of sequencing error		1.24	8.82E-02	0.99	9.14E-01	0.88	1.92E-01
MSMEG_5368	ectoine/hydroxyectoine ABC transporter solute-binding protein	*ehuB*	2.44	7.67E-04	0.54	3.04E-02	0.46	1.77E-02
MSMEG_5369	ectoine/hydroxyectoine ABC transporter, permease protein EhuC	*ehuC*	1.28	8.22E-02	0.73	4.27E-02	0.98	9.55E-01
MSMEG_5370	ectoine/hydroxyectoine ABC transporter, permease protein EhuD	*ehuD*	1.02	8.74E-01	0.73	4.44E-02	0.78	3.27E-01
MSMEG_5371	ectoine/hydroxyectoine ABC transporter, ATP-binding protein	*ehuA*	1.24	9.66E-02	0.71	9.38E-02	0.71	1.80E-01
MSMEG_5372	sensor protein KdpD		1.89	6.80E-03	1.07	2.69E-01	0.94	8.72E-01
MSMEG_5373	nitrilase 2		0.78	5.83E-02	1.03	9.00E-01	0.99	7.75E-01
MSMEG_5374	glutamate--ammonia ligase		0.65	8.16E-02	1.01	9.22E-01	1.05	6.18E-01
MSMEG_5375	GntR-family protein transcriptional regulator		1.33	2.57E-02	0.86	1.65E-02	1.35	2.63E-01
MSMEG_5376	conserved hypothetical protein		0.89	8.83E-02	1.18	4.84E-01	1.99	3.77E-01
MSMEG_5377	hypothetical protein		2.78	5.94E-02	0.56	6.24E-02	0.64	1.07E-01
MSMEG_5381	hypothetical protein		0.94	3.85E-01	1.71	9.60E-02	0.92	3.29E-01
MSMEG_5382	hypothetical protein; this gene contains a frame shift which is not the result of sequencing error; identified by Glimmer2; putative		1.56	2.35E-03	1.32	1.33E-01	1.12	3.52E-01
MSMEG_5383	dehydrogenase/reductase SDR family protein member 4		1.06	7.07E-01	1.95	5.50E-02	1.31	3.23E-01
MSMEG_5384	hypothetical protein		1.13	3.98E-01	1.66	1.96E-01	1.31	1.93E-01
MSMEG_5385	metallo-beta-lactamase family protein		1.13	4.73E-01	3.15	8.65E-02	1.02	3.33E-01
MSMEG_5386	rhodanese-like domain protein		1.08	2.35E-01	1.75	3.64E-01	1.15	4.25E-01
MSMEG_5387	conserved hypothetical protein		1.17	2.18E-01	1.13	1.65E-01	1.21	3.83E-01
MSMEG_5388	conserved hypothetical protein		1.52	1.16E-02	1.29	1.08E-01	0.95	2.69E-01
MSMEG_5389	LysA protein		1.46	9.91E-02	0.88	7.25E-02	1.06	3.15E-01
MSMEG_5390	conserved hypothetical protein		1.81	2.32E-03	1.63	1.09E-01	0.64	1.82E-04
MSMEG_5391	hypothetical protein		1.25	1.58E-01	2.05	1.93E-01	1.16	5.18E-01
MSMEG_5392	K+-transporting ATPase, A subunit	*kdpA*	1.41	1.36E-02	3.20	1.13E-01	0.98	6.10E-01

MSMEG_5393	K+-transporting ATPase, B subunit	*kdpB*	1.26	2.84E-01	1.35	6.10E-02	1.28	3.74E-01
MSMEG_5394	potassium-transporting ATPase C chain		0.96	1.29E-01	1.13	4.56E-01	1.14	4.91E-01
MSMEG_5395	sensor protein KdpD		1.87	7.88E-03	1.10	5.95E-01	1.89	6.92E-02
MSMEG_5396	KDP operon transcriptional regulatory protein KdpE		1.21	3.48E-01	0.98	8.45E-01	1.09	2.82E-01
MSMEG_5397	ATP-dependent DNA helicase RecQ	*recQ*	2.09	2.66E-01	4.31	3.97E-01	1.13	2.91E-01
MSMEG_5399	ATP-dependent DNA helicase RecQ		0.85	1.89E-01	1.00	9.72E-01	1.37	1.98E-01
MSMEG_5400	dehydrogenase		1.19	3.79E-01	1.24	1.14E-01	1.00	9.78E-01
MSMEG_5401	conserved hypothetical protein		2.51	3.22E-01	3.03	1.92E-01	0.83	5.35E-01
MSMEG_5402	dehydrogenase DhgA		1.69	1.03E-01	1.38	1.39E-01	0.70	4.66E-02
MSMEG_5403	cadmium-translocating P-type ATPase	*cadA*	1.35	2.07E-01	1.19	1.82E-01	1.00	9.52E-01
MSMEG_5404	propionate--CoA ligase		1.49	1.96E-02	0.87	1.21E-01	0.32	3.11E-05
MSMEG_5405	transcriptional regulator, ArsR family protein		1.14	9.82E-02	0.92	1.30E-01	0.82	1.74E-01
MSMEG_5406	hypothetical protein		2.85	7.85E-04	0.85	2.55E-01	0.92	6.91E-01
MSMEG_5407	integral membrane protein		2.30	6.13E-03	0.69	4.64E-02	1.06	6.59E-01
MSMEG_5408	prolipoprotein diacylglyceryl transferase	*lgt*	1.31	2.44E-01	1.39	2.95E-01	1.52	6.06E-01
MSMEG_5409	conserved hypothetical protein		1.99	9.67E-02	0.78	1.63E-02	0.85	3.15E-01
MSMEG_5410	radical SAM domain protein		1.13	1.40E-01	0.90	3.21E-02	2.12	4.27E-01
MSMEG_5411	conserved hypothetical protein		0.74	1.50E-02	1.22	4.75E-01	1.22	3.21E-01
MSMEG_5412	immunogenic protein MPT63		1.59	1.07E-02	0.55	7.23E-03	0.30	9.46E-06
MSMEG_5413	exopolyphosphatase		0.45	1.37E-04	1.03	7.96E-01	1.00	9.62E-01
MSMEG_5414	septum formation initiator subfamily protein, putative		0.42	3.19E-05	1.05	5.28E-01	1.41	1.98E-01
MSMEG_5415	enolase	*eno*	0.35	4.99E-06	0.76	1.93E-02	0.66	3.63E-03
MSMEG_5416	LpqU protein		0.88	2.69E-01	1.05	4.64E-01	1.82	5.34E-03
MSMEG_5417	hypothetical protein		0.82	8.89E-02	1.27	4.34E-01	0.98	7.40E-01
MSMEG_5418	iron permease FTR1		0.31	9.15E-05	0.96	6.96E-01	0.65	1.78E-02
MSMEG_5419	lipoprotein; this gene contains a frame shift which is not the result of sequencing error; identified by match to protein family HMM PF04302		0.29	5.88E-05	1.06	6.23E-01	0.46	1.43E-03
MSMEG_5420	Tat-translocated enzyme		0.58	1.29E-03	1.09	5.29E-01	0.60	1.36E-02
MSMEG_5421	hypothetical protein		1.01	9.71E-01	0.94	5.51E-01	1.36	1.39E-01
MSMEG_5422	transcriptional regulator, MazG family protein		0.47	7.11E-04	0.89	4.20E-01	3.07	6.34E-03
MSMEG_5423	transcription-repair coupling factor	*mfd*	0.46	7.71E-05	1.02	8.78E-01	2.78	3.29E-02
MSMEG_5424	transcriptional regulator, TetR family protein		0.86	3.62E-02	0.91	4.87E-02	1.46	1.42E-01
MSMEG_5426	UDP-N-acetylglucosamine pyrophosphorylase	*glmU*	0.48	5.97E-04	0.99	9.00E-01	1.03	7.73E-01
MSMEG_5427	ribose-phosphate pyrophosphokinase		0.58	3.14E-04	0.94	2.81E-01	2.00	1.88E-02
MSMEG_5428	arsenate reductase	*arsC*	0.41	3.36E-06	0.88	7.23E-02	2.25	9.14E-03

MSMEG_5429	LpqT protein		0.34	3.66E-05	0.66	1.46E-02	3.25	5.17E-04
MSMEG_5429	LpqT protein		0.81	2.64E-02	1.14	3.13E-01	0.75	2.90E-01
MSMEG_5430	retinol dehydrogenase 13		0.76	2.37E-03	1.00	9.74E-01	0.76	1.02E-01
MSMEG_5431	ribosomal protein L25, Ctc-form		0.60	6.63E-04	0.85	7.69E-03	0.68	1.90E-02
MSMEG_5432	peptidyl-tRNA hydrolase	*pth*	0.46	6.64E-05	0.85	6.17E-02	0.92	4.71E-01
MSMEG_5433	4Fe-4S binding domain protein		0.96	6.72E-01	1.14	1.79E-01	1.00	9.44E-01
MSMEG_5434	hypothetical protein		1.44	1.57E-02	0.88	5.03E-01	1.82	8.00E-02
MSMEG_5435	acyl-CoA synthase		1.23	2.54E-02	0.71	6.34E-02	2.58	9.11E-03
MSMEG_5436	4-diphosphocytidyl-2C-methyl-D-erythritol kinase	*ispE*	0.37	9.13E-05	0.97	7.95E-01	1.43	3.10E-01
MSMEG_5437	probable serine/threonine-protein kinase PknB		0.93	5.54E-01	0.98	7.86E-01	1.04	6.52E-01
MSMEG_5438	dimethyladenosine transferase	*ksgA*	2.05	1.88E-03	1.04	4.77E-01	0.27	3.32E-05
MSMEG_5439	resuscitation-promoting factor RpfB		0.46	4.87E-04	1.15	3.02E-01	0.70	1.30E-01
MSMEG_5440	deoxyribonuclease		0.50	1.32E-03	1.33	3.80E-01	1.05	1.18E-01
MSMEG_5441	methionyl-tRNA synthetase	*metG*	0.40	1.07E-06	0.83	3.07E-02	1.40	1.10E-01
MSMEG_5442	glutamate dehydrogenase		0.55	7.18E-04	0.75	1.57E-02	1.35	1.45E-01
MSMEG_5443	dehydrogenase		4.69	2.24E-03	1.22	2.31E-01	0.41	3.82E-03
MSMEG_5444	RNA polymerase sigma-70 factor, TIGR02957 family protein		3.64	6.54E-03	0.67	1.25E-02	0.92	8.81E-01
MSMEG_5445	conserved hypothetical protein		0.98	8.92E-01	1.14	4.07E-01	1.50	2.33E-01
MSMEG_5446	para-aminobenzoate synthase, component I	*pabB*	2.00	1.65E-02	0.80	2.67E-01	1.59	2.68E-01
MSMEG_5447	dolichyl-phosphate-mannose-protein mannosyltransferase		0.86	1.22E-01	1.02	7.69E-01	1.17	3.42E-01
MSMEG_5448	arginine deiminase	*arcA*	1.26	9.32E-03	1.02	7.16E-01	1.59	1.12E-01
MSMEG_5449	conserved hypothetical protein		1.16	5.38E-01	0.94	4.89E-01	1.20	3.52E-01
MSMEG_5450	redox-sensitive transcriptional activator SoxR	*soxR*	1.11	2.37E-01	0.93	3.83E-01	1.20	2.51E-01
MSMEG_5451	alkylated DNA repair protein		1.17	2.60E-01	0.81	2.15E-01	1.31	4.22E-01
MSMEG_5452	conserved hypothetical protein		0.67	7.94E-03	0.92	5.51E-01	0.99	9.81E-01
MSMEG_5453	conserved hypothetical protein		1.00	9.93E-01	1.01	9.31E-01	1.14	2.39E-01
MSMEG_5454	choloylglycine hydrolase, putative		0.49	1.60E-03	1.71	5.46E-02	1.33	2.68E-01
MSMEG_5455	pe family protein		1.73	3.83E-02	1.34	4.77E-02	0.98	1.80E-01
MSMEG_5456	MK35 lipoprotein		1.24	2.21E-01	1.08	3.75E-01	0.86	1.12E-01
MSMEG_5457	shikimate 5-dehydrogenase		2.42	3.34E-04	0.81	2.11E-02	1.91	1.08E-02
MSMEG_5458	cyclic nucleotide-binding protein		2.01	6.76E-03	1.11	2.89E-01	2.18	2.98E-02
MSMEG_5459	hypothetical protein		1.05	8.41E-01	1.16	1.08E-01	1.53	1.99E-01
MSMEG_5460	conserved hypothetical protein		1.94	3.15E-02	0.82	5.87E-02	0.96	2.05E-01
MSMEG_5461	hypothetical protein		2.14	9.48E-04	0.82	1.01E-01	0.83	4.23E-01
MSMEG_5462	RarD protein	*rarD*	0.91	1.99E-01	1.00	9.75E-01	1.65	3.33E-01

MSMEG_5463	6-phosphogluconate dehydrogenase, NAD-binding		2.37	1.37E-02	0.96	8.24E-01	1.10	2.36E-01
MSMEG_5464	integral membrane transport protein		1.53	1.54E-02	0.94	6.41E-01	1.35	4.09E-01
MSMEG_5465	transcriptional regulator, AraC family protein		1.82	2.78E-03	0.78	2.41E-02	1.02	2.73E-01
MSMEG_5466	oxidoreductase		1.53	2.73E-02	1.69	4.19E-01	1.17	2.99E-01
MSMEG_5468	conserved hypothetical protein		1.54	2.54E-01	0.96	6.97E-01	1.03	9.00E-01
MSMEG_5469	acetyltransferase, GNAT family protein		0.47	5.63E-04	1.07	5.22E-01	1.37	4.15E-01
MSMEG_5470	molybdopterin biosynthesis protein MoeA 1		0.71	5.17E-03	0.85	3.91E-03	15.51	3.53E-01
MSMEG_5471	UTP-glucose-1-phosphate uridylyltransferase	*galU*	0.88	1.62E-03	1.05	1.99E-01	0.73	1.06E-02
MSMEG_5472	5,10-methenyltetrahydrofolate synthetase		1.50	3.85E-04	0.90	8.89E-02	1.13	3.27E-01
MSMEG_5473	transporter		1.60	8.15E-02	1.03	8.20E-01	0.89	3.45E-01
MSMEG_5474	universal stress protein family protein		1.29	2.76E-01	1.28	4.23E-01	0.87	1.63E-01
MSMEG_5475	acetate operon repressor		1.11	1.25E-01	0.86	2.12E-01	0.55	8.46E-05
MSMEG_5476	glyoxylate carboligase	*gcl*	2.15	2.36E-03	0.67	3.03E-03	0.44	1.27E-02
MSMEG_5477	2-hydroxy-3-oxopropionate reductase		3.23	4.87E-02	0.67	4.51E-02	0.16	1.52E-05
MSMEG_5478	hydroxypyruvate isomerase, putative		3.36	1.09E-03	0.52	6.70E-04	0.34	2.55E-02
MSMEG_5479	type I antifreeze protein		0.89	9.15E-02	0.80	4.17E-02	1.19	8.21E-02
MSMEG_5480	hypothetical protein		1.25	2.99E-02	0.85	3.87E-01	0.64	3.46E-02
MSMEG_5481	conserved hypothetical protein		0.68	3.07E-02	0.82	9.59E-03	1.31	2.45E-01
MSMEG_5482	large conductance mechanosensitive channel protein	*mscL*	0.94	3.73E-01	1.99	3.09E-01	0.52	1.14E-02
MSMEG_5483	porin		2.04	1.26E-03	1.33	1.95E-01	0.18	7.58E-05
MSMEG_5484	conserved hypothetical protein		1.00	9.82E-01	0.87	1.50E-01	2.06	1.31E-02
MSMEG_5485	molybdopterin biosynthesis protein		1.28	2.73E-03	0.60	3.23E-03	3.57	4.81E-03
MSMEG_5486	peptidase S1 and S6, chymotrypsin/Hap		1.98	6.45E-03	0.78	8.93E-02	15.01	8.68E-03
MSMEG_5487	sensor histidine kinase		1.20	1.17E-01	0.65	1.53E-03	3.96	1.04E-03
MSMEG_5488	DNA-binding response regulator		1.47	1.01E-02	0.79	2.59E-02	3.73	1.35E-04
MSMEG_5489	ribosomal protein L32	*rpmF*	0.83	1.85E-02	0.75	9.35E-02	0.97	3.97E-01
MSMEG_5490	conserved hypothetical protein		3.19	1.92E-03	0.78	3.86E-02	1.17	3.45E-01
MSMEG_5491	putative acyl-CoA dehydrogenase		2.92	5.11E-03	1.15	7.34E-01	0.98	9.37E-01
MSMEG_5492	acetyl-CoA carboxylase carboxyltransferase		2.76	3.25E-03	0.84	1.61E-01	0.80	1.90E-01
MSMEG_5493	acetyl-/propionyl-coenzyme A carboxylase alpha chain		2.13	5.07E-03	0.73	1.13E-02	0.90	4.44E-01
MSMEG_5494	acyl-CoA dehydrogenase fadE12		2.23	9.27E-03	0.89	2.90E-01	0.83	1.12E-01
MSMEG_5495	enoyl-CoA hydratase		1.59	5.68E-02	0.86	5.44E-02	1.08	4.75E-01
MSMEG_5496	MscS Mechanosensitive ion channel		1.44	1.00E-01	0.94	3.43E-01	1.06	4.10E-01
MSMEG_5497	conserved hypothetical protein		1.02	8.72E-01	1.22	1.54E-01	0.99	2.86E-01
MSMEG_5498	conserved hypothetical protein		1.40	5.54E-02	1.32	2.30E-01	0.97	1.17E-01

MSMEG_5499	conserved hypothetical protein		2.02	2.60E-01	0.91	5.20E-01	0.92	2.98E-01
MSMEG_5500	hypothetical protein		1.05	5.52E-01	1.35	2.14E-01	1.86	2.51E-01
MSMEG_5501	hypothetical protein		2.46	2.69E-01	0.87	3.97E-01	0.97	4.22E-01
MSMEG_5502	hypothetical protein		0.58	2.26E-02	1.06	6.05E-01	1.04	5.85E-01
MSMEG_5503	hypothetical protein		0.75	7.37E-02	0.97	4.72E-01	1.56	3.96E-01
MSMEG_5504	conserved secreted protein		1.92	3.83E-03	1.02	8.50E-01	1.25	9.76E-04
MSMEG_5505	conserved hypothetical protein		1.62	4.80E-03	0.74	2.33E-02	6.92	1.56E-03
MSMEG_5506	hypothetical protein		4.98	3.78E-02	0.92	6.59E-01	0.86	2.64E-01
MSMEG_5507	hypothetical protein		0.96	8.31E-01	1.11	3.01E-01	1.03	3.18E-01
MSMEG_5508	alkaline phosphatase		1.58	2.88E-01	1.17	1.38E-01	1.08	1.81E-01
MSMEG_5509	conserved hypothetical protein		0.98	8.58E-01	1.11	2.02E-02	0.70	1.34E-01
MSMEG_5510	conserved hypothetical protein; this gene contains a frame shift which is not the result of sequencing error		1.06	7.32E-01	1.25	2.43E-01	1.07	6.12E-01
MSMEG_5511	von Willebrand factor, type A		1.30	1.06E-03	0.99	8.37E-01	1.69	2.56E-03
MSMEG_5512	magnesium chelatase		1.39	5.85E-03	1.02	7.65E-01	0.89	2.58E-01
MSMEG_5513	serine/threonine-protein kinase PknE		0.65	6.13E-03	0.95	3.08E-01	0.87	5.83E-01
MSMEG_5514	hypothetical protein		0.45	1.24E-05	1.24	2.18E-01	1.29	1.06E-01
MSMEG_5515	bifunctional purine biosynthesis protein PurH	*purH*	0.42	6.64E-05	0.95	3.41E-01	1.14	4.66E-02
MSMEG_5516	phosphoribosylglycinamide formyltransferase	*purN*	0.51	6.08E-04	0.88	4.08E-01	1.66	7.22E-03
MSMEG_5517	conserved hypothetical protein		0.58	3.14E-03	0.73	3.97E-02	2.37	8.99E-02
MSMEG_5518	antigen 34 kDa		1.56	4.97E-03	0.59	2.02E-02	1.28	2.67E-01
MSMEG_5519	monooxygenase		1.39	4.14E-01	3.12	2.02E-01	0.93	4.54E-01
MSMEG_5520	conserved hypothetical protein		1.10	2.81E-01	1.09	4.04E-01	0.37	3.63E-03
MSMEG_5521	acetyl-CoA acetyltransferase		1.07	4.92E-01	1.07	2.63E-01	1.16	2.47E-01
MSMEG_5522	transcriptional Regulator, TetR family protein		2.72	8.09E-04	0.71	8.05E-03	0.80	1.63E-01
MSMEG_5523	peptidase		0.80	3.24E-01	1.12	3.55E-01	1.05	2.20E-01
MSMEG_5524	succinyl-CoA synthetase, alpha subunit	*sucD*	0.52	1.34E-03	0.91	3.18E-01	0.68	1.04E-01
MSMEG_5525	succinyl-CoA synthetase, beta subunit	*sucC*	0.50	9.16E-04	0.91	3.92E-01	1.02	9.32E-01
MSMEG_5526	peptidoglycan-binding LysM		1.45	1.03E-02	1.21	8.01E-02	0.47	1.09E-03
MSMEG_5527	conserved hypothetical protein		0.81	8.20E-02	0.83	1.77E-01	0.84	1.87E-02
MSMEG_5528	hypothetical protein		1.07	2.09E-01	1.26	2.21E-01	1.19	2.53E-01
MSMEG_5529	hypothetical protein		0.98	9.08E-01	7.31	3.03E-01	1.03	1.91E-01
MSMEG_5530	putative aliphatic sulfonates transport permease protein SsuC		1.04	4.62E-01	2.20	8.61E-02	1.00	9.97E-01
MSMEG_5531	ABC transporter ATP-binding protein		1.00	9.89E-01	1.85	2.83E-01	0.83	3.75E-01
MSMEG_5532	FAD dependent oxidoreductase		1.16	7.89E-02	3.01	2.03E-01	1.49	3.48E-01
MSMEG_5533	4Fe-4S ferredoxin, iron-sulfur binding		0.97	6.10E-01	5.15	1.63E-01	1.06	2.93E-01

MSMEG_5534	ATP-dependent DNA helicase PcrA	*pcrA*	0.61	8.27E-05	0.93	3.36E-01	1.63	1.71E-02
MSMEG_5535	hypothetical protein		2.96	1.23E-02	0.60	9.81E-03	1.11	2.18E-01
MSMEG_5536	chorismate mutase		0.74	6.21E-03	0.72	4.80E-02	1.40	3.89E-02
MSMEG_5537	conserved integral membrane protein		0.82	2.17E-01	0.79	6.87E-02	1.35	2.47E-01
MSMEG_5538	[NADP+] succinate-semialdehyde dehydrogenase		1.33	7.87E-03	0.78	1.46E-02	0.84	1.81E-03
MSMEG_5539	hypothetical protein		3.84	8.14E-03	0.82	7.96E-02	0.68	7.02E-03
MSMEG_5540	conserved hypothetical protein		0.49	1.84E-03	1.03	7.47E-01	1.29	7.84E-01
MSMEG_5541	glucose-6-phosphate isomerase	*pgi*	0.50	4.94E-04	1.03	7.88E-01	0.58	3.14E-03
MSMEG_5542	transcriptional regulator, HTH_3 family protein		0.65	6.31E-02	2.05	1.21E-02	0.32	1.14E-04
MSMEG_5543	hypothetical protein		2.30	2.18E-01	2.63	1.00E-02	0.30	3.04E-04
MSMEG_5544	oxidoreductase, short chain dehydrogenase/reductase family protein		0.67	2.53E-04	1.15	3.67E-01	1.15	3.64E-01
MSMEG_5545	formamidopyrimidine-DNA glycosylase		2.09	2.84E-03	0.82	1.00E-01	1.01	9.18E-01
MSMEG_5546	membrane spanning protein		0.70	7.36E-03	1.03	6.53E-01	1.18	3.71E-01
MSMEG_5547	conserved hypothetical protein		1.19	1.19E-01	1.05	5.17E-01	0.92	4.44E-01
MSMEG_5548	precorrin-6A synthase (deacetylating)	*cobF*	0.42	4.94E-04	1.03	8.78E-01	0.98	5.40E-01
MSMEG_5549	conserved hypothetical protein		17.35	8.33E-04	0.56	2.92E-02	3.00	3.16E-02
MSMEG_5550	protein-glutamate methylesterase; this gene contains a frame shift which is not the result of sequencing error; identified by match to protein family HMM PF01339		2.50	9.17E-03	1.11	5.91E-01	0.76	6.08E-02
MSMEG_5550	protein-glutamate methylesterase; this gene contains a frame shift which is not the result of sequencing error; identified by match to protein family HMM PF01339		3.87	1.00E-02	0.90	6.70E-01	1.14	1.03E-01
MSMEG_5551	stas domain protein		2.56	9.14E-04	0.82	4.60E-02	1.41	3.74E-01
MSMEG_5552	conserved hypothetical protein		2.01	8.85E-05	1.04	4.29E-01	1.08	4.51E-01
MSMEG_5553	CheR methyltransferase, SAM binding domain protein	*cheR*	0.66	9.25E-04	1.08	9.31E-02	1.35	2.80E-02
MSMEG_5554	antar domain protein		3.13	4.00E-04	0.96	5.48E-01	0.66	1.78E-02
MSMEG_5555	conserved hypothetical protein; this gene contains a frame shift which is not the result of sequencing error		0.80	9.74E-03	1.19	1.03E-01	0.63	1.11E-02
MSMEG_5556	MFS permease		0.20	2.31E-05	4.72	9.56E-02	4.58	3.08E-02
MSMEG_5557	conserved hypothetical protein		1.14	3.28E-01	1.25	1.37E-01	0.92	7.18E-01
MSMEG_5558	hypothetical protein		3.04	5.40E-03	1.85	2.03E-01	1.04	6.12E-01
MSMEG_5559	metabolite/sugar transport protein		1.06	7.90E-01	2.80	4.80E-02	1.06	6.02E-01
MSMEG_5560	hypothetical protein		0.73	1.63E-02	1.68	1.14E-01	1.28	3.78E-01

MSMEG_5561	HPP family protein		1.94	5.36E-02	1.35	2.63E-01	1.04	5.65E-01
MSMEG_5562	RNase H	*rnhA*	0.63	2.85E-02	1.33	1.57E-01	1.05	7.36E-01
MSMEG_5563	AraC-family protein transcriptional regulator		0.98	9.19E-01	1.35	2.90E-01	1.08	2.78E-01
MSMEG_5564	NAD(P)H dehydrogenase, quinone family protein		1.22	6.17E-02	1.15	4.73E-01	1.32	8.63E-02
MSMEG_5565	conserved hypothetical protein		3.61	2.08E-02	0.64	6.56E-02	0.99	9.49E-01
MSMEG_5566	transcriptional regulator		0.86	8.49E-02	0.95	3.36E-01	1.40	1.61E-01
MSMEG_5567	transcriptional regulator, TetR family protein		1.72	6.59E-03	0.84	1.39E-01	0.79	2.43E-01
MSMEG_5568	clavaldehyde dehydrogenase		12.30	1.17E-02	0.45	5.74E-03	1.80	2.66E-01
MSMEG_5569	clavaldehyde dehydrogenase		14.46	2.85E-02	0.46	2.79E-03	1.44	4.83E-01
MSMEG_5570	DNA ligase		1.50	7.08E-02	1.11	2.80E-02	0.99	5.90E-01
MSMEG_5571	ABC transporter ATP-binding protein		1.57	6.24E-02	1.15	3.06E-01	0.13	5.41E-07
MSMEG_5572	sugar ABC transporter permease protein		1.60	1.11E-01	1.42	1.97E-01	0.25	8.84E-05
MSMEG_5573	sugar ABC transporter permease protein		1.75	2.11E-03	1.38	1.00E-01	0.21	1.61E-04
MSMEG_5574	substrate binding protein		1.94	1.09E-03	1.57	6.63E-02	0.24	7.99E-04
MSMEG_5575	repressor		2.01	4.96E-03	1.37	1.84E-01	0.34	2.12E-03
MSMEG_5576	D-mannonate oxidoreductase		1.36	4.78E-02	1.17	2.91E-01	0.46	4.71E-02
MSMEG_5577	fructokinase		1.58	1.30E-02	1.21	1.13E-01	0.74	9.01E-02
MSMEG_5578	hypothetical protein		1.05	3.14E-01	1.35	5.52E-02	1.00	9.41E-01
MSMEG_5579	MarR-family protein transcriptional regulator		1.05	7.34E-01	1.29	3.62E-01	0.96	3.88E-01
MSMEG_5580	Ku protein		1.64	4.73E-03	0.84	2.15E-02	0.68	1.83E-02
MSMEG_5581	conserved hypothetical integral membrane protein		1.54	1.69E-02	1.04	4.60E-01	1.64	6.94E-02
MSMEG_5582	hypothetical protein		16.68	2.44E-04	1.04	8.13E-01	0.91	1.38E-01
MSMEG_5583	HNH endonuclease		0.84	2.04E-01	1.30	4.44E-02	2.88	1.56E-01
MSMEG_5584	short chain dehydrogenase		1.33	1.50E-01	1.11	1.01E-01	1.14	5.68E-01
MSMEG_5585	hypothetical protein		1.03	8.21E-01	0.83	3.96E-01	1.36	5.24E-01
MSMEG_5586	conserved hypothetical protein		1.53	2.28E-02	1.02	7.94E-01	0.80	4.27E-02
MSMEG_5587	hypothetical protein		0.92	3.09E-01	0.59	3.12E-02	0.64	6.64E-02
MSMEG_5588	hypothetical protein		0.96	2.48E-01	0.76	1.41E-01	0.50	5.00E-04
MSMEG_5589	manganese transport protein MntH		1.39	4.51E-02	0.80	2.17E-02	0.38	1.04E-03
MSMEG_5590	carboxylate-amine ligase Nfa27300		2.17	2.21E-02	0.98	7.16E-01	0.22	7.21E-05
MSMEG_5591	conserved hypothetical protein		1.06	5.28E-01	0.92	1.75E-01	0.38	2.48E-04
MSMEG_5592	conserved hypothetical protein		0.97	5.63E-01	0.76	6.45E-02	0.58	3.51E-02
MSMEG_5593	pyruvate dehydrogenase		0.82	3.80E-01	1.04	8.01E-01	0.91	2.96E-01
MSMEG_5594	ferredoxin-dependent glutamate synthase		0.66	3.47E-03	1.20	3.53E-01	2.11	1.96E-01
MSMEG_5595	MarR-family protein transcriptional regulator		0.91	2.12E-01	0.63	1.18E-04	0.96	7.98E-01
MSMEG_5596	oxidoreductase		1.16	4.51E-01	1.27	1.39E-01	0.93	2.83E-01
MSMEG_5597	transcriptional regulator, TetR family protein		1.49	5.21E-01	0.82	2.75E-01	1.71	3.81E-01
MSMEG_5599	hypothetical protein		0.92	4.04E-01	1.07	5.46E-01	1.23	2.31E-01

MSMEG_5600	conserved hypothetical protein	0.98	8.79E-01	0.92	1.84E-01	1.03	6.12E-01
MSMEG_5601	hypothetical protein	0.87	2.08E-02	1.18	3.86E-01	1.00	6.55E-01
MSMEG_5602	conserved hypothetical protein	0.81	4.92E-02	0.94	4.51E-01	2.46	2.57E-01
MSMEG_5603	transcriptional regulator, ArsR family protein	1.22	3.64E-01	1.05	7.31E-01	1.02	8.32E-01
MSMEG_5604	integral membrane protein	1.01	9.44E-01	1.48	1.61E-01	1.13	5.13E-01
MSMEG_5605	cytochrome bd ubiquinol oxidase, subunit I	1.24	3.22E-01	1.41	9.36E-02	1.52	2.52E-01
MSMEG_5606	cytochrome bd-I oxidase subunit II	1.17	4.45E-01	2.55	2.15E-01	1.16	6.48E-01
MSMEG_5607	hypothetical protein	1.42	7.16E-03	1.86	1.26E-01	0.82	3.32E-02
MSMEG_5608	acetyl-CoA acetyltransferase	1.23	3.61E-01	1.80	2.46E-01	1.00	9.85E-01
MSMEG_5609	carotenoid oxygenase	2.48	3.91E-03	0.92	2.56E-01	1.22	1.89E-02
MSMEG_5610	putative transcriptional regulator family protein	1.23	1.36E-01	1.27	2.47E-01	1.23	1.57E-01
MSMEG_5611	spore protein	4.42	3.05E-04	0.76	3.71E-02	1.52	1.94E-01
MSMEG_5612	amino-acid acetyltransferase	0.87	4.68E-01	1.00	9.85E-01	0.89	3.39E-01
MSMEG_5613	probable conserved transmembrane protein	0.48	3.78E-04	1.04	6.24E-01	2.27	3.61E-01
MSMEG_5614	conserved hypothetical protein	1.42	1.24E-01	0.94	5.16E-01	0.68	3.20E-02
MSMEG_5615	conserved hypothetical protein	1.08	3.94E-01	1.06	3.12E-01	0.91	7.03E-01
MSMEG_5616	glyoxalase/bleomycin resistance protein/dioxygenase	1.70	1.28E-02	1.01	9.15E-01	0.77	1.89E-01
MSMEG_5617	immunogenic protein MPT63	0.69	3.45E-01	1.34	1.15E-01	0.80	2.52E-01
MSMEG_5618	acyl-CoA dehydrogenase	1.65	1.43E-01	1.99	4.05E-01	1.00	9.81E-01
MSMEG_5619	acyl-CoA dehydrogenase domain protein	1.21	3.90E-01	8.80	3.16E-01	1.00	9.47E-01
MSMEG_5620	enoyl-CoA hydratase	1.13	2.96E-01	1.36	1.73E-02	1.03	5.18E-01
MSMEG_5621	putative acyl-CoA dehydrogenase	1.43	1.27E-01	1.04	8.50E-01	1.01	9.60E-01
MSMEG_5622	putative acyl-CoA dehydrogenase	1.44	2.25E-01	1.52	2.43E-01	1.01	5.95E-01
MSMEG_5623	L-carnitine dehydratase/bile acid-inducible protein F	1.19	3.07E-01	1.14	4.41E-01	0.95	5.95E-01
MSMEG_5624	oxidoreductase, short chain dehydrogenase/reductase family protein	1.09	2.13E-01	12.95	3.90E-01	1.18	5.10E-01
MSMEG_5625	cyclododecanone monooxygenase	1.17	1.71E-01	0.96	6.49E-01	0.97	8.14E-01
MSMEG_5626	luciferase	1.82	2.04E-01	1.24	8.27E-02	0.93	2.22E-01
MSMEG_5627	Rieske [2Fe-2S] domain protein	1.05	2.71E-01	1.77	7.73E-02	1.16	5.89E-01
MSMEG_5628	oxidoreductase, short chain dehydrogenase/reductase family protein	1.08	3.82E-01	1.95	1.48E-01	1.19	2.92E-01
MSMEG_5629	conserved hypothetical protein	0.94	4.70E-01	1.01	9.10E-01	1.11	4.93E-01
MSMEG_5630	transcriptional regulator, GntR family protein	0.92	4.10E-01	1.30	4.76E-02	0.89	3.14E-01
MSMEG_5631	AMP-dependent synthetase and ligase	1.04	6.08E-01	1.28	5.24E-01	1.18	5.54E-01
MSMEG_5632	oxidoreductase, short chain dehydrogenase/reductase family protein	0.92	3.16E-01	1.14	3.43E-01	0.92	1.40E-01

MSMEG_5633	feruloyl-CoA synthetase		1.08	4.97E-01	0.99	8.09E-01	1.57	7.79E-02
MSMEG_5634	conserved hypothetical protein		0.72	2.16E-02	1.07	1.15E-01	1.06	6.28E-01
MSMEG_5635	conserved hypothetical protein		0.97	7.43E-01	1.11	1.57E-01	1.70	2.13E-02
MSMEG_5636	P-type ATPase - metal cation transport		0.45	4.84E-04	1.26	6.87E-02	1.11	3.94E-01
MSMEG_5637	penicillin-binding protein 4		0.88	3.32E-01	0.99	8.06E-01	1.44	5.74E-02
MSMEG_5638	metallo-beta-lactamase family protein		0.59	2.62E-04	0.95	5.09E-01	1.78	1.80E-01
MSMEG_5639	enoyl-CoA hydratase		0.88	1.90E-01	0.70	1.04E-02	1.16	5.73E-02
MSMEG_5640	amylo-alpha-1,6-glucosidase		1.51	1.20E-02	1.06	4.91E-01	1.11	2.77E-01
MSMEG_5641	glycosyl transferase, group 1 family protein		1.25	1.85E-02	0.80	1.20E-02	0.98	5.63E-01
MSMEG_5642	acetyl-coenzyme a carboxylase carboxyl transferase		0.97	6.08E-01	0.93	8.08E-02	1.37	4.55E-02
MSMEG_5643	conserved hypothetical protein		1.05	6.46E-01	1.10	3.34E-01	1.10	2.37E-01
MSMEG_5644	membrane protein		0.59	1.70E-03	1.32	6.19E-02	1.14	1.75E-01
MSMEG_5645	conserved hypothetical protein		0.55	4.64E-05	1.52	8.09E-03	1.08	3.87E-01
MSMEG_5646	conserved hypothetical protein		1.14	7.34E-01	0.96	7.89E-01	1.54	1.77E-01
MSMEG_5647	conserved hypothetical protein; this gene contains a frame shift which is not the result of sequencing error		1.62	7.78E-03	0.76	2.93E-04	1.67	1.19E-01
MSMEG_5648	hypothetical protein		1.21	6.19E-02	0.90	2.09E-01	0.85	6.14E-02
MSMEG_5649	acyl-CoA synthase		3.75	3.78E-03	1.50	2.85E-02	0.13	9.06E-05
MSMEG_5650	AMP-dependent synthetase and ligase		5.21	3.04E-03	1.50	6.47E-02	0.12	9.30E-06
MSMEG_5651	transcriptional regulator, LuxR family protein		3.73	8.25E-04	0.89	2.83E-01	0.10	1.04E-05
MSMEG_5652	alpha/beta hydrolase fold		0.97	7.30E-01	1.53	1.10E-01	1.10	2.18E-01
MSMEG_5653	cytosine/purine/uracil/thiamine/allantoin permease family protein		1.05	7.15E-01	1.55	2.85E-01	3.32	1.80E-01
MSMEG_5654	transcriptional regulator, LysR family protein		1.09	2.60E-01	1.24	3.65E-01	1.02	6.92E-01
MSMEG_5655	hydrolase, carbon-nitrogen family protein		0.98	3.82E-01	1.56	1.16E-01	20.57	1.08E-01
MSMEG_5656	histidinol dehydrogenase	*hisD*	0.89	3.48E-01	1.31	3.55E-01	1.79	1.18E-01
MSMEG_5657	transcriptional regulator, LysR family protein		1.29	1.78E-01	1.49	2.95E-01	1.01	8.56E-01
MSMEG_5658	epoxide hydrolase		0.90	4.80E-01	0.86	1.59E-02	0.76	7.09E-02
MSMEG_5659	ABC transporter, ATP-binding protein		0.81	1.36E-01	1.34	2.58E-01	1.12	3.64E-01
MSMEG_5660	ABC transporter ATP-binding protein		0.87	2.11E-01	1.95	1.30E-01	1.87	7.68E-02
MSMEG_5661	ABC transporter ATP-binding protein		0.83	4.79E-02	4.74	1.27E-01	0.95	7.37E-01
MSMEG_5662	DNA-binding response regulator PrrA	*prrA*	0.74	1.82E-03	0.89	5.49E-02	1.21	9.12E-02
MSMEG_5663	sensor-type histidine kinase PrrB		1.06	3.16E-01	0.86	9.61E-02	1.61	2.53E-01
MSMEG_5664	binding protein		0.70	1.91E-04	0.84	1.85E-01	0.60	3.50E-05
MSMEG_5665	hypothetical protein		1.36	2.06E-01	1.44	3.30E-01	0.86	2.90E-01
MSMEG_5666	conserved hypothetical protein		3.12	3.41E-03	0.79	1.64E-02	5.93	4.03E-01

MSMEG_5667	phytoene dehydrogenase		1.81	3.09E-02	0.86	1.35E-01	1.13	7.77E-01
MSMEG_5668	putative transcriptional regulator		1.45	2.01E-01	1.02	8.64E-01	1.16	3.14E-01
MSMEG_5670	efflux membrane protein		0.99	9.52E-01	1.45	5.93E-02	1.39	4.47E-01
MSMEG_5671	YbaK/prolyl-tRNA synthetase associated region		0.82	1.47E-01	1.09	6.39E-01	0.98	6.17E-01
MSMEG_5672	citrate synthase I	*gltA*	0.40	7.50E-06	0.77	1.58E-01	1.37	2.45E-01
MSMEG_5673	transcriptional regulator		2.71	8.69E-05	1.28	1.27E-01	1.09	7.57E-01
MSMEG_5674	membrane transport protein		0.83	5.40E-01	1.74	2.03E-01	2.79	2.54E-04
MSMEG_5675	pyridoxamine 5'-phosphate oxidase	*pdxH*	0.87	1.58E-02	1.20	8.82E-02	1.42	5.50E-02
MSMEG_5676	citrate synthase		0.98	5.40E-01	0.91	1.33E-01	1.42	4.21E-03
MSMEG_5677	conserved hypothetical protein		0.61	5.54E-04	0.87	4.87E-02	2.37	5.70E-03
MSMEG_5678	glyoxalase/bleomycin resistance protein/dioxygenase		0.98	7.79E-01	0.84	5.95E-02	0.82	2.73E-02
MSMEG_5679	putative DNA-binding protein		1.03	5.97E-01	0.89	8.07E-03	0.59	4.44E-03
MSMEG_5680	glyoxalase family protein		1.91	2.50E-02	0.77	4.85E-02	1.27	7.38E-02
MSMEG_5681	probable ferredoxin/ferredoxin--NADP reductase		3.35	1.76E-03	0.61	1.39E-02	3.88	8.84E-02
MSMEG_5682	conserved hypothetical protein		6.08	8.24E-05	0.65	2.03E-02	2.86	3.17E-02
MSMEG_5683	methionine aminopeptidase, type I	*map*	1.61	1.01E-02	0.80	7.99E-02	1.24	1.99E-01
MSMEG_5684	phosphoserine aminotransferase, putative		0.43	5.76E-05	1.04	5.89E-01	0.93	1.99E-01
MSMEG_5685	DNA-binding protein		1.39	1.79E-02	1.02	8.25E-01	1.31	1.55E-01
MSMEG_5686	conserved hypothetical protein		1.24	5.80E-02	0.85	2.99E-01	1.26	2.00E-01
MSMEG_5687	23s ribosomal RNA methyltransferase		0.39	1.66E-05	1.12	3.08E-01	4.14	8.47E-02
MSMEG_5688	regulatory protein, MarR		0.61	1.28E-01	1.22	2.71E-01	1.06	1.57E-01
MSMEG_5689	conserved hypothetical protein		1.21	3.55E-02	0.82	1.98E-01	1.32	6.57E-01
MSMEG_5690	conserved hypothetical protein		1.42	1.81E-03	0.75	1.63E-02	0.69	4.47E-02
MSMEG_5691	conserved hypothetical protein		1.20	6.07E-02	0.69	6.30E-02	1.72	3.94E-01
MSMEG_5692	conserved hypothetical protein		1.12	3.36E-01	1.71	1.02E-01	0.99	5.59E-01
MSMEG_5693	transporter, major facilitator family protein		2.67	3.38E-01	0.86	3.47E-02	6.44	2.67E-01
MSMEG_5694	conserved hypothetical protein		0.77	9.57E-03	0.87	2.14E-01	1.37	2.25E-02
MSMEG_5695	glutathione S-transferase		1.74	1.69E-03	1.21	7.93E-02	0.66	4.80E-03
MSMEG_5696	'Cold-shock' DNA-binding domain protein		0.45	5.58E-05	1.08	6.08E-01	0.69	2.53E-02
MSMEG_5697	integral membrane protein		1.48	3.61E-01	1.07	8.41E-01	1.84	5.14E-01
MSMEG_5698	molybdenum cofactor biosynthesis protein A	*moaA*	1.91	6.82E-03	0.72	2.58E-02	1.53	2.70E-02
MSMEG_5699	ThiS family protein		2.59	9.09E-04	0.90	2.94E-01	0.88	3.98E-01
MSMEG_5700	secreted protein		1.05	7.59E-01	1.09	2.06E-01	1.99	2.65E-01
MSMEG_5701	molybdopterin converting factor, subunit 2	*moaE*	0.93	5.81E-01	0.93	2.85E-01	1.72	5.50E-02
MSMEG_5702	molybdenum cofactor synthesis domain protein		0.63	1.06E-03	0.93	2.70E-01	1.04	5.11E-01
MSMEG_5703	molybdenum cofactor biosynthesis protein C	*moaC*	0.65	9.85E-03	1.30	1.83E-01	1.99	1.30E-02
MSMEG_5704	conserved hypothetical protein		0.84	1.30E-02	0.75	3.36E-02	0.78	1.61E-04

MSMEG_5705	conserved hypothetical protein		0.95	4.53E-01	0.88	2.38E-01	1.16	3.18E-01
MSMEG_5706	DNA or RNA helicase of superfamily protein II		0.88	1.95E-02	0.80	6.33E-02	1.14	1.06E-01
MSMEG_5707	cupin domain protein		0.77	1.02E-03	0.85	5.29E-02	1.23	1.48E-01
MSMEG_5708	hypothetical protein		0.85	9.16E-03	0.99	7.74E-01	0.86	4.76E-02
MSMEG_5709	conserved hypothetical protein		0.75	1.65E-02	1.26	1.20E-01	0.69	3.25E-02
MSMEG_5710	hypothetical protein		0.73	8.53E-03	1.35	9.11E-02	0.38	1.30E-03
MSMEG_5711	oxidoreductase, short chain dehydrogenase/reductase family protein		1.22	2.74E-01	1.33	3.11E-01	1.60	1.92E-01
MSMEG_5712	LysR-family protein transcriptional regulator		1.26	1.66E-01	0.94	2.83E-01	0.99	9.56E-01
MSMEG_5713	major facilitator family protein transporter		1.37	5.30E-02	0.88	1.20E-01	2.60	4.99E-02
MSMEG_5714	short-chain dehydrogenase/reductase SDR		1.63	1.70E-05	0.67	3.26E-02	4.05	3.69E-01
MSMEG_5715	conserved hypothetical protein		1.46	8.38E-03	0.75	9.76E-03	0.64	3.64E-02
MSMEG_5716	hypothetical protein		1.75	4.55E-02	0.92	1.74E-01	1.80	2.01E-01
MSMEG_5717	pyridoxamine 5'-phosphate oxidase family		1.56	1.06E-02	0.79	9.23E-03	0.15	4.44E-06
MSMEG_5718	transcriptional regulator, TetR family protein		1.18	1.42E-01	1.05	4.95E-01	1.00	9.77E-01
MSMEG_5719	oxidoreductase		0.91	7.93E-01	0.84	1.46E-01	1.02	4.99E-01
MSMEG_5720	putative 3-hydroxyacyl-CoA dehydrogenase		2.14	2.61E-03	0.83	1.53E-01	2.37	6.00E-02
MSMEG_5721	acetyl-CoA acetyltransferase		1.65	1.45E-02	1.04	6.91E-01	1.93	9.39E-03
MSMEG_5722	conserved hypothetical protein		1.67	3.58E-01	4.92	1.56E-02	0.62	7.00E-02
MSMEG_5723	endoribonuclease L-psp family protein		0.82	1.51E-01	4.30	1.38E-01	0.96	7.82E-01
MSMEG_5724	bacterial regulatory protein, MarR family protein		1.28	1.89E-02	0.96	6.06E-01	1.72	4.86E-02
MSMEG_5725	PLP-dependent aminotransferases		0.75	1.01E-02	0.82	1.25E-01	1.77	3.19E-02
MSMEG_5726	conserved hypothetical protein		0.69	1.76E-02	0.96	7.86E-01	1.42	5.01E-01
MSMEG_5727	allantoicase	*alc*	0.72	1.85E-02	0.85	3.33E-01	1.15	3.60E-01
MSMEG_5728	polysaccharide deacetylase family protein		0.80	4.06E-02	0.83	3.45E-01	1.34	5.90E-02
MSMEG_5729	hydantoin racemase		0.57	5.88E-03	1.19	4.84E-01	0.75	5.05E-02
MSMEG_5730	permease for cytosine/purines, uracil, thiamine, allantoin transporter		0.21	1.19E-04	1.15	2.63E-01	0.81	2.99E-01
MSMEG_5731	transcriptional regulator, GntR family protein		0.76	2.59E-02	1.22	1.66E-01	0.80	2.11E-01
MSMEG_5732	monooxygenase		1.95	4.04E-03	1.21	3.55E-01	1.26	3.34E-01
MSMEG_5733	universal stress protein family protein		1.51	2.91E-02	5.66	1.10E-03	5.35	1.73E-02
MSMEG_5734	alpha-methylacyl-CoA racemase		0.57	2.08E-05	0.87	2.45E-01	0.98	9.19E-01
MSMEG_5735	indole-3-pyruvate decarboxylase		2.01	7.43E-02	0.93	5.70E-01	0.95	7.36E-01
MSMEG_5736	cyclase/dehydrase		1.63	2.67E-04	1.10	3.04E-01	0.40	4.82E-04
MSMEG_5737	hypothetical protein		1.18	3.91E-02	1.25	2.82E-01	1.22	3.40E-01
MSMEG_5738	conserved hypothetical protein		1.92	4.70E-03	0.83	8.04E-02	0.93	2.91E-01
MSMEG_5739	putative long-chain fatty-acid--CoA ligase		1.34	1.21E-01	0.98	5.96E-01	1.11	1.50E-01
MSMEG_5740	hypothetical protein		0.85	8.17E-03	1.06	6.29E-01	0.92	3.46E-01

MSMEG_5741	conserved hypothetical protein		0.76	1.38E-03	0.91	5.05E-01	0.75	7.35E-02
MSMEG_5742	acyltransferase domain protein		1.29	2.13E-02	0.82	1.49E-02	1.14	7.04E-01
MSMEG_5743	patatin		1.67	8.77E-03	1.15	2.90E-01	1.06	5.00E-01
MSMEG_5744	gas vesicle protein K	*gvpK*	2.57	1.60E-02	1.05	7.57E-01	0.65	1.08E-01
MSMEG_5745	gas vesicle synthesis protein		0.95	4.72E-01	0.98	8.93E-01	1.30	4.32E-01
MSMEG_5746	gas vesicle protein		0.90	2.53E-01	1.40	1.82E-01	0.96	3.03E-01
MSMEG_5747	hypothetical protein		0.79	5.93E-02	1.16	4.84E-01	1.24	4.10E-03
MSMEG_5748	hypothetical protein		0.86	1.55E-01	1.06	3.54E-01	2.16	1.15E-02
MSMEG_5749	hypothetical protein		1.16	6.26E-02	0.91	2.24E-01	0.83	1.79E-01
MSMEG_5750	hypothetical protein		1.24	1.53E-02	0.91	1.98E-01	1.40	5.93E-02
MSMEG_5751	putative adhesin/hemolysin		1.33	9.17E-03	0.81	2.60E-03	1.34	8.58E-02
MSMEG_5752	conserved hypothetical protein		0.84	1.90E-01	0.83	1.40E-01	0.84	2.23E-01
MSMEG_5753	serine/threonine specific protein phosphatase		0.59	6.83E-03	1.21	4.08E-01	1.07	7.61E-01
MSMEG_5754	gp41 protein		1.41	6.09E-02	0.95	6.80E-01	0.96	3.55E-01
MSMEG_5759	pe family protein		1.01	8.26E-01	0.95	7.08E-01	1.08	4.53E-01
MSMEG_5760	GntR-family protein regulator		1.79	9.67E-02	1.10	3.98E-01	1.07	5.98E-01
MSMEG_5761	cupin domain protein		4.03	1.44E-01	1.00	9.93E-01	1.06	6.27E-01
MSMEG_5762	zinc-binding oxidoreductase		2.35	1.72E-01	0.84	3.12E-01	1.09	1.76E-01
MSMEG_5763	conserved hypothetical protein		3.84	1.44E-01	0.99	9.72E-01	1.06	5.51E-01
MSMEG_5764	putative cyanamide hydratase		1.64	2.63E-01	0.88	2.90E-01	1.25	4.79E-01
MSMEG_5765	globin		0.45	1.54E-02	1.32	6.55E-02	1.13	5.86E-01
MSMEG_5766	conserved hypothetical protein		0.62	4.51E-03	1.12	3.10E-01	0.77	8.01E-02
MSMEG_5767	conserved hypothetical protein		1.56	2.13E-02	0.58	5.04E-03	0.83	2.08E-01
MSMEG_5768	conserved hypothetical protein		0.91	1.17E-01	0.89	2.92E-01	0.98	8.06E-01
MSMEG_5769	hypothetical protein		0.68	1.15E-03	0.89	1.69E-01	1.54	9.52E-03
MSMEG_5770	hypothetical protein		1.06	5.27E-01	0.76	8.27E-02	0.34	9.04E-07
MSMEG_5771	hypothetical protein		0.73	9.19E-04	0.97	8.59E-01	0.45	4.65E-03
MSMEG_5772	cytospin-A, putative		1.21	6.22E-01	1.10	3.47E-01	0.94	5.58E-01
MSMEG_5773	fatty acid desaturase		0.78	3.86E-03	2.01	7.14E-03	7.33	1.12E-03
MSMEG_5774	tRNA-dihydrouridine synthase, putative		1.09	3.99E-01	1.36	2.67E-02	4.88	6.20E-03
MSMEG_5775	LytR/CpsA/Psr family protein		1.53	4.32E-03	0.62	1.53E-03	0.96	8.82E-01
MSMEG_5776	phosphate transport system regulatory protein PhoU	*phoU*	2.40	1.02E-03	0.97	7.29E-01	0.58	3.93E-04
MSMEG_5777	transcription regulator		1.37	1.64E-01	0.99	9.22E-01	1.07	6.09E-01
MSMEG_5778	alcohol dehydrogenase, zinc-binding		1.16	7.10E-03	0.98	6.33E-01	1.14	3.52E-01
MSMEG_5779	phosphate ABC transporter, ATP-binding protein	*pstB*	2.08	1.75E-01	0.82	1.29E-01	1.74	2.32E-02
MSMEG_5780	phosphate ABC transporter, permease protein PstA	*pstA*	1.17	4.95E-02	0.70	1.10E-02	2.20	5.03E-02

MSMEG_5781	phosphate ABC transporter, permease protein PstC	*pstC*	1.25	1.13E-02	0.83	9.52E-03	1.43	1.78E-02
MSMEG_5782	phosphate ABC transporter, phosphate-binding protein PstS	*pstS*	0.90	2.70E-01	0.96	7.30E-01	1.01	9.28E-01
MSMEG_5783	acetyltransferase, GNAT family; this gene contains a frame shift which is not the result of sequencing error; identified by match to protein family HMM PF00583		0.62	5.93E-04	2.54	1.06E-01	1.56	2.21E-01
MSMEG_5784	transcriptional regulatory protein		1.31	2.68E-03	0.95	5.39E-01	0.84	1.61E-01
MSMEG_5785	probable exported protein		0.43	4.65E-04	1.05	5.11E-01	1.65	7.86E-02
MSMEG_5786	thiredoxin		0.70	9.68E-04	0.96	5.58E-01	0.98	8.05E-01
MSMEG_5787	hypothetical protein		0.87	3.52E-01	0.98	7.73E-01	1.33	3.37E-01
MSMEG_5788	integral membrane protein		0.76	2.25E-02	1.84	1.46E-01	2.34	1.44E-01
MSMEG_5789	putative thiosulfate sulfurtransferase		0.62	5.34E-04	1.57	1.15E-01	1.85	8.02E-03
MSMEG_5790	SseC protein		0.61	4.13E-03	1.38	2.64E-01	2.87	1.59E-02
MSMEG_5791	conserved hypothetical protein		0.57	8.10E-05	0.97	8.13E-01	1.73	5.35E-03
MSMEG_5792	conserved hypothetical protein		0.50	6.53E-05	1.05	4.99E-01	1.61	8.86E-03
MSMEG_5793	hypothetical protein		0.46	6.74E-07	0.92	1.09E-02	2.21	2.40E-02
MSMEG_5794	hypothetical protein		1.21	6.88E-01	0.64	9.17E-02	1.21	4.68E-01
MSMEG_5795	4-amino-4-deoxychorismate lyase		1.16	3.88E-02	0.84	1.58E-01	1.10	5.89E-01
MSMEG_5796	Glycine cleavage T-protein (aminomethyl transferase)		1.77	1.12E-03	0.69	2.05E-03	0.80	5.61E-02
MSMEG_5797	conserved hypothetical protein		1.74	9.82E-05	0.71	1.83E-02	1.89	6.39E-02
MSMEG_5798	phosphoribosylformylglycinamidine cyclo-ligase	*purM*	0.67	6.40E-03	0.87	7.53E-02	0.81	3.87E-03
MSMEG_5799	nucleoside-diphosphate-sugar epimerase		1.62	4.15E-01	1.03	6.06E-01	1.00	9.49E-01
MSMEG_5800	amidophosphoribosyltransferase	*purF*	2.04	1.28E-03	0.70	1.70E-02	1.49	1.05E-01
MSMEG_5801	hydroxylase		1.20	1.70E-01	1.04	7.99E-01	0.99	7.72E-01
MSMEG_5802	oxidoreductase, short chain dehydrogenase/reductase family protein		0.88	3.93E-01	1.06	3.66E-01	1.55	8.35E-02
MSMEG_5803	hypothetical protein		1.01	9.13E-01	0.98	7.97E-01	0.95	5.66E-01
MSMEG_5804	conserved hypothetical protein		0.69	1.98E-02	0.74	5.29E-02	1.49	1.31E-01
MSMEG_5805	hypothetical protein		0.65	3.16E-03	1.13	1.07E-01	1.57	2.50E-01
MSMEG_5806	hypothetical protein		2.46	9.28E-02	1.11	3.63E-01	5.18	3.72E-01
MSMEG_5807	D-methionine transport ATP-binding protein MetN		0.94	7.53E-01	2.28	2.44E-01	1.09	1.53E-01
MSMEG_5808	binding-protein-dependent transport systems inner membrane component		1.20	3.02E-01	1.67	4.57E-02	1.00	9.74E-01
MSMEG_5809	lipoprotein YaeC, putative		0.98	8.19E-01	1.97	2.52E-01	1.27	1.73E-01

MSMEG_5810	putative monooxygenase		1.13	5.52E-01	1.98	3.33E-01	1.17	1.44E-01
MSMEG_5811	transcriptional regulator, TetR family protein		0.85	1.25E-01	1.20	2.87E-01	0.98	2.39E-01
MSMEG_5812	carveol dehydrogenase		1.35	6.77E-02	0.76	5.84E-02	1.05	3.70E-01
MSMEG_5813	carveol dehydrogenase		1.41	3.85E-01	0.98	7.42E-01	1.12	4.90E-01
MSMEG_5814	4-carboxymuconolactone decarboxylase domain protein	*pcaC*	0.88	3.16E-01	0.87	1.22E-02	1.01	8.28E-01
MSMEG_5815	betaine aldehyde dehydrogenase		0.74	1.07E-02	1.18	4.35E-01	1.15	5.62E-01
MSMEG_5816	conserved hypothetical protein		0.64	1.70E-04	0.67	2.32E-02	0.96	7.35E-01
MSMEG_5817	conserved hypothetical protein		1.04	7.09E-01	1.04	5.36E-01	1.36	5.92E-02
MSMEG_5818	virulence factor Mce family protein		1.01	9.38E-01	0.98	5.63E-01	1.04	2.14E-01
MSMEG_5819	pyridoxamine 5'-phosphate oxidase family		1.52	2.71E-01	1.14	2.41E-01	0.87	3.20E-01
MSMEG_5820	hypothetical protein		0.99	9.49E-01	1.00	9.78E-01	2.00	5.34E-01
MSMEG_5821	caax amino protease family protein		0.62	7.58E-03	1.05	5.05E-01	1.66	6.77E-02
MSMEG_5822	conserved hypothetical protein		1.25	2.14E-01	0.99	9.50E-01	1.03	8.09E-01
MSMEG_5823	conserved membrane-spanning protein		0.82	4.99E-01	1.09	1.88E-01	2.16	7.70E-02
MSMEG_5824	phosphoribosylformylglycinamidine synthase II	*purL*	0.64	2.31E-04	1.05	5.38E-01	1.64	3.16E-03
MSMEG_5825	Na+/H+ antiporter		0.80	6.74E-02	0.94	7.23E-01	0.94	6.06E-01
MSMEG_5826	pyruvate decarboxylase		1.14	5.19E-01	1.32	3.94E-02	0.69	1.10E-01
MSMEG_5827	glyoxalase family protein		0.59	2.25E-03	0.77	9.45E-02	1.17	1.66E-01
MSMEG_5828	aspartyl aminopeptidase		0.49	4.12E-04	0.86	3.88E-01	1.69	1.41E-02
MSMEG_5829	Dyp-type peroxidase		0.96	6.56E-01	0.96	3.08E-01	0.56	1.37E-02
MSMEG_5830	29 kda antigen Cfp29		0.88	7.74E-02	1.03	8.35E-01	0.53	2.72E-05
MSMEG_5831	phosphoribosylformylglycinamidine synthase I	*purQ*	0.51	1.82E-04	0.95	3.63E-01	0.87	2.53E-01
MSMEG_5832	phosphoribosylformylglycinamidine synthase, PurS protein	*purS*	0.67	3.50E-04	0.97	5.74E-01	1.40	8.76E-02
MSMEG_5833	conserved hypothetical protein; this gene contains a frame shift which is not the result of sequencing error		1.40	9.39E-02	1.12	3.18E-01	0.95	6.38E-01
MSMEG_5834	metallo-beta-lactamase superfamily protein		0.99	8.88E-01	1.11	1.70E-01	0.65	7.04E-04
MSMEG_5835	fumarate reductase/succinate dehydrogenase flavoprotein		1.02	7.12E-01	0.87	2.19E-01	0.57	4.42E-02
MSMEG_5836	conserved hypothetical protein		0.50	3.53E-02	1.36	2.37E-01	1.15	5.82E-01
MSMEG_5837	glutathione peroxidase family protein		0.66	3.01E-04	0.95	5.95E-01	0.92	6.32E-01
MSMEG_5838	TetR-family protein transcriptional regulator		0.94	2.60E-01	0.90	9.88E-03	1.89	4.13E-01
MSMEG_5839	protease 2		0.52	3.42E-05	0.90	3.74E-02	1.44	1.51E-01
MSMEG_5840	conserved hypothetical protein		0.95	3.54E-01	1.62	1.67E-01	0.92	2.53E-01
MSMEG_5841	phosphoribosylaminoimidazole-succinocarboxamide synthase	*purC*	0.54	3.60E-05	1.00	9.70E-01	0.88	3.57E-01

MSMEG_5842	conserved hypothetical protein		1.75	2.93E-02	1.12	8.58E-02	1.19	3.33E-01
MSMEG_5843	conserved hypothetical protein		0.76	5.57E-02	0.73	4.29E-02	0.93	1.63E-01
MSMEG_5844	conserved hypothetical protein		0.94	6.23E-01	1.22	1.41E-01	0.87	3.48E-01
MSMEG_5845	putative C4-dicarboxylate transporter		0.62	2.50E-02	1.12	2.34E-01	1.12	6.02E-01
MSMEG_5846	NikQ protein		0.78	6.24E-02	0.90	2.92E-01	1.10	5.14E-01
MSMEG_5847	adenylosuccinate lyase	*purB*	0.50	3.89E-05	0.86	4.73E-02	1.34	4.17E-02
MSMEG_5848	amino acid permease-associated region		1.17	3.55E-01	1.12	1.56E-01	0.67	2.36E-02
MSMEG_5849	conserved hypothetical protein		1.64	3.28E-03	0.89	7.95E-02	1.19	1.78E-01
MSMEG_5850	transcriptional regulator, TetR family protein		6.68	1.34E-02	1.58	2.83E-02	0.08	4.72E-08
MSMEG_5851	putative esterase superfamily protein		1.13	2.02E-01	0.70	4.16E-03	1.45	2.33E-01
MSMEG_5852	phosphoribosylamine--glycine ligase	*purD*	0.38	2.54E-05	0.79	5.82E-02	2.75	1.40E-02
MSMEG_5853	cytochrome P450		1.45	8.93E-02	0.94	6.78E-01	0.94	5.72E-01
MSMEG_5854	transcriptional regulator, TetR family protein		0.79	1.54E-01	1.04	5.36E-01	1.27	2.46E-01
MSMEG_5855	conserved hypothetical protein		0.70	2.74E-02	1.02	8.98E-01	0.92	4.64E-01
MSMEG_5856	4-carboxymuconolactone decarboxylase domain protein	*pcaC*	0.83	5.21E-02	1.05	6.35E-01	1.03	8.19E-01
MSMEG_5857	hypothetical oxidoreductase		0.94	7.57E-01	1.01	8.42E-01	1.26	4.39E-01
MSMEG_5858	3-oxoacyl-[acyl-carrier-protein] reductase		0.83	1.01E-01	1.00	9.96E-01	0.82	1.79E-01
MSMEG_5859	aldehyde dehydrogenase (NAD) family protein		0.84	1.28E-01	1.15	2.04E-01	0.93	4.56E-01
MSMEG_5860	transcriptional regulator, TetR family protein		1.74	2.93E-01	1.24	8.77E-02	1.01	8.62E-01
MSMEG_5861	cytochrome P450 109		0.84	1.18E-01	0.94	4.79E-01	1.00	9.11E-01
MSMEG_5862	short chain dehydrogenase		1.27	4.40E-01	1.02	6.83E-01	1.00	8.64E-01
MSMEG_5863	cytochrome P450 51		0.93	5.34E-01	1.04	8.40E-01	0.99	6.06E-01
MSMEG_5864	cytochrome P450 51		0.71	7.07E-02	1.09	4.13E-01	1.79	4.17E-01
MSMEG_5865	conserved hypothetical protein		1.08	1.62E-01	1.11	3.87E-01	1.02	5.67E-01
MSMEG_5866	alcohol dehydrogenase B		3.82	5.30E-03	0.97	7.82E-01	0.08	8.12E-07
MSMEG_5867	nuclear transport factor 2 (NTF2) domain superfamily protein		3.09	7.73E-02	1.26	4.25E-01	0.24	1.17E-04
MSMEG_5868	hypothetical protein		0.98	8.63E-01	1.03	5.68E-01	8.04	9.91E-03
MSMEG_5869	putative short chain dehydrogenase		1.19	1.94E-01	1.15	1.74E-01	0.72	1.04E-01
MSMEG_5870	sensor histidine kinase PhoR		0.73	1.95E-05	0.79	4.84E-02	0.98	8.88E-01
MSMEG_5871	HIT family protein		0.70	7.32E-05	1.04	6.14E-01	0.80	6.24E-02
MSMEG_5872	DNA-binding response regulator PhoP	*phoP*	2.38	1.89E-03	0.84	1.52E-03	0.22	3.70E-05
MSMEG_5873	conserved hypothetical protein		1.46	1.35E-03	0.90	2.21E-02	0.61	4.25E-04
MSMEG_5875	hypothetical protein		1.02	8.87E-01	1.09	5.57E-01	0.55	8.14E-03
MSMEG_5876	H-N-H endonuclease F-TflIV		0.77	1.72E-01	0.95	6.33E-01	1.20	4.27E-01
MSMEG_5877	conserved hypothetical protein		1.49	9.30E-02	0.72	2.23E-01	0.92	7.97E-01
MSMEG_5878	serine esterase, cutinase family protein		6.13	2.99E-05	0.79	3.94E-02	1.46	5.64E-03

MSMEG_5879	D-alanyl-D-alanine dipeptidase	0.77	2.75E-03	0.93	3.54E-01	0.85	1.16E-01
MSMEG_5880	nicotine dehydrogenase	0.95	7.51E-01	1.09	5.44E-01	0.97	5.09E-01
MSMEG_5881	putative carbon monoxide dehydrogenase subunit G	1.42	4.53E-01	1.16	2.77E-01	0.89	4.31E-01
MSMEG_5882	carbon monoxide dehydrogenase medium chain	0.89	1.39E-01	1.18	3.04E-01	1.10	6.52E-01
MSMEG_5883	[2Fe-2S] binding domain protein	0.97	7.67E-01	1.17	3.09E-01	1.34	3.71E-01
MSMEG_5884	3-hydroxyisobutyrate dehydrogenase family protein	1.99	1.64E-03	0.68	4.06E-03	1.02	8.81E-01
MSMEG_5885	short chain dehydrogenase	1.87	7.98E-03	0.58	2.88E-02	0.95	8.12E-01
MSMEG_5886	enoyl-CoA hydratase	0.80	1.25E-01	1.09	3.15E-01	1.47	4.07E-01
MSMEG_5887	intersectin-EH binding protein Ibp1	3.59	9.24E-04	0.53	1.84E-02	0.21	1.11E-04
MSMEG_5888	conserved hypothetical protein	0.68	9.23E-04	1.36	8.11E-03	1.20	4.52E-01
MSMEG_5889	conserved hypothetical protein	0.93	4.85E-01	0.92	6.34E-01	0.37	1.63E-05
MSMEG_5890	conserved hypothetical protein	1.21	1.29E-01	0.92	3.52E-01	1.44	3.03E-02
MSMEG_5892	alpha,alpha-trehalose-phosphate synthase (UDP-forming)	0.94	3.52E-01	0.89	1.07E-01	1.62	3.84E-04
MSMEG_5893	conserved hypothetical protein	0.52	9.31E-05	1.11	3.09E-01	1.04	8.66E-01
MSMEG_5894	conserved hypothetical protein	0.45	5.00E-06	1.16	3.20E-01	0.80	2.32E-01
MSMEG_5895	virulence factor mce family protein	0.55	1.13E-04	1.12	2.41E-01	0.37	1.14E-03
MSMEG_5896	virulence factor Mce family protein	0.54	1.11E-03	1.15	1.78E-01	0.57	6.04E-03
MSMEG_5897	virulence factor mce family protein	0.52	1.42E-04	1.29	5.05E-02	0.49	1.94E-02
MSMEG_5898	virulence factor Mce family protein	0.55	2.04E-04	1.18	5.62E-02	0.64	6.07E-02
MSMEG_5899	virulence factor Mce family protein	0.47	9.32E-05	1.14	2.56E-01	0.78	8.74E-02
MSMEG_5900	virulence factor Mce family protein	0.56	3.34E-04	1.05	5.19E-01	0.66	1.05E-01
MSMEG_5901	TrnB2 protein	0.50	4.08E-04	1.19	1.65E-01	0.86	6.08E-01
MSMEG_5902	conserved hypothetical protein	0.70	2.93E-02	0.94	5.92E-01	0.61	1.22E-03
MSMEG_5903	oxidoreductase, short chain dehydrogenase/reductase family protein	0.95	1.53E-01	0.80	7.29E-06	1.02	9.26E-01
MSMEG_5904	conserved domain protein	0.86	4.88E-02	1.25	5.63E-02	1.57	1.50E-02
MSMEG_5905	hypothetical protein	1.38	1.82E-01	1.13	5.49E-01	0.95	3.48E-01
MSMEG_5906	putative acyl-CoA dehydrogenase	3.26	2.98E-02	0.90	6.86E-01	0.77	1.75E-01
MSMEG_5907	acyl-CoA dehydrogenase	2.62	4.77E-02	0.99	9.67E-01	1.05	7.36E-01
MSMEG_5908	acyl-CoA synthase	1.50	4.71E-02	1.14	5.55E-01	1.42	1.70E-01
MSMEG_5909	oxidoreductase	2.02	3.48E-02	1.10	3.46E-01	1.17	2.88E-01
MSMEG_5910	quinone binding protein	1.38	2.97E-03	0.65	6.55E-03	0.61	2.65E-03
MSMEG_5911	AraC family protein transcriptional regulator	2.44	1.38E-03	0.73	1.92E-02	2.67	1.93E-02
MSMEG_5912	succinic semialdehyde dehydrogenase	2.23	8.37E-03	0.95	4.63E-01	1.26	4.58E-02
MSMEG_5913	dioxygenase	1.58	9.95E-02	0.80	1.91E-01	0.91	6.79E-01

MSMEG_5914	acyl-CoA synthase		1.30	2.58E-01	0.94	7.10E-01	0.86	3.48E-01
MSMEG_5915	enoyl-CoA hydratase		1.76	2.32E-02	0.77	2.06E-02	1.12	1.60E-01
MSMEG_5916	hypothetical protein		2.35	5.22E-02	0.60	1.24E-03	2.02	3.88E-01
MSMEG_5917	conserved hypothetical protein		1.44	2.00E-01	0.95	5.44E-01	1.06	3.47E-01
MSMEG_5918	P450 heme-thiolate protein		1.63	3.74E-02	0.95	5.10E-01	0.89	2.33E-01
MSMEG_5919	conserved hypothetical protein		1.24	2.86E-01	0.88	3.17E-01	1.13	2.31E-01
MSMEG_5920	FMN-dependent monooxygenase		1.16	3.60E-01	0.94	1.57E-01	1.49	1.79E-01
MSMEG_5921	conserved hypothetical protein		1.32	1.18E-01	0.64	7.46E-03	0.58	5.55E-02
MSMEG_5922	lipid-transfer protein		1.19	2.19E-01	0.74	4.16E-03	0.86	8.85E-02
MSMEG_5923	acetyl-CoA acetyltransferase		1.29	2.99E-01	0.68	3.89E-03	1.19	5.29E-01
MSMEG_5924	conserved hypothetical protein		1.51	2.11E-02	0.89	2.05E-01	1.89	4.64E-04
MSMEG_5925	Rieske [2Fe-2S] domain protein		0.94	6.40E-01	0.73	8.92E-02	1.14	5.31E-01
MSMEG_5926	putative secreted protein		1.13	6.39E-01	1.16	4.04E-01	0.96	5.93E-01
MSMEG_5927	conserved hypothetical protein		0.84	1.79E-01	0.77	2.23E-02	2.25	1.37E-01
MSMEG_5928	conserved hypothetical protein		1.06	8.67E-01	0.97	7.60E-01	1.05	5.52E-01
MSMEG_5929	conserved hypothetical protein		0.67	2.24E-02	0.82	3.10E-02	1.12	1.33E-01
MSMEG_5930	conserved hypothetical protein		0.66	4.53E-03	1.07	4.58E-01	0.95	5.45E-01
MSMEG_5931	short chain dehydrogenase		0.78	4.37E-02	1.01	9.32E-01	1.04	8.75E-01
MSMEG_5932	conserved hypothetical protein		0.90	3.74E-02	1.08	6.25E-01	1.06	3.37E-01
MSMEG_5933	conserved hypothetical protein		0.88	2.37E-01	1.47	7.03E-02	1.23	4.66E-01
MSMEG_5934	conserved hypothetical protein		3.06	1.76E-02	0.95	4.93E-01	0.63	8.10E-02
MSMEG_5935	ATP-dependent DNA helicase		0.59	6.45E-03	0.96	6.98E-01	2.07	4.68E-02
MSMEG_5936	conserved hypothetical protein		0.59	5.17E-03	1.58	7.87E-02	0.52	2.70E-02
MSMEG_5937	4-hydroxy-2-oxovalerate aldolase		1.39	1.44E-02	0.82	3.51E-03	0.51	9.42E-03
MSMEG_5938	hypothetical protein		0.89	5.36E-01	0.96	6.20E-01	0.98	7.50E-01
MSMEG_5939	acetaldehyde dehydrogenase		1.09	1.04E-01	0.72	4.34E-03	0.44	3.70E-03
MSMEG_5940	2-keto-4-pentenoate hydratase		0.74	2.02E-03	0.91	1.81E-01	0.50	1.25E-02
MSMEG_5941	3-ketosteroid dehydrogenase		0.30	1.89E-05	1.06	6.63E-01	0.75	2.00E-01
MSMEG_5942	AMP-dependent synthetase and ligase		1.00	9.95E-01	1.21	3.14E-01	0.93	2.10E-01
MSMEG_5943	putative peroxisomal multifunctional enzyme type 2		0.43	1.32E-04	0.82	8.19E-02	0.67	2.24E-01
MSMEG_5944	glucose-methanol-choline oxidoreductase		1.64	1.54E-01	0.96	2.54E-01	0.93	7.19E-01
MSMEG_5945	conserved hypothetical protein		3.72	2.65E-02	0.51	1.03E-03	0.82	1.54E-01
MSMEG_5946	hypothetical protein		1.29	2.20E-01	1.00	9.53E-01	1.03	2.54E-03
MSMEG_5947	serine O-acetyltransferase	*cysE*	1.31	2.63E-01	0.83	7.85E-02	0.67	8.40E-02
MSMEG_5948	glycosyl transferase		2.77	1.51E-01	0.97	7.48E-01	0.98	6.35E-01
MSMEG_5949	conserved hypothetical protein		1.90	6.95E-02	1.09	2.37E-01	0.84	1.36E-01

MSMEG_5950	putative colanic acid biosynthesis acetyltransferase WcaF		1.77	4.47E-02	0.88	3.08E-01	0.87	2.08E-01
MSMEG_5951	glycosyl transferase, group 1, putative		1.70	1.68E-01	1.17	2.98E-01	0.97	6.36E-01
MSMEG_5952	conserved hypothetical protein, putative		1.44	2.64E-01	0.97	6.54E-01	0.61	3.90E-02
MSMEG_5953	hypothetical protein		1.12	5.15E-01	1.14	4.43E-02	0.89	2.85E-01
MSMEG_5954	cell surface polysaccharide biosynthesis, putative		1.45	7.64E-02	1.24	1.78E-01	0.82	2.18E-01
MSMEG_5955	protein-tyrosine kinase, putative		1.66	9.68E-02	0.88	2.76E-02	0.64	8.18E-02
MSMEG_5956	NAD dependent epimerase/dehydratase family protein		1.24	1.44E-01	0.79	1.58E-01	0.64	1.24E-01
MSMEG_5957	GDP-mannose 6-dehydrogenase AlgD	*algD*	2.43	2.07E-01	0.99	8.92E-01	0.58	1.79E-01
MSMEG_5958	hypothetical protein		1.15	2.36E-01	1.05	6.45E-01	0.61	1.23E-01
MSMEG_5959	conserved hypothetical protein		0.86	9.75E-02	1.09	4.00E-01	0.60	8.38E-02
MSMEG_5960	O-antigen polymerase, putative		1.58	1.73E-01	1.15	5.47E-01	0.86	4.75E-01
MSMEG_5961	glycosyl transferase, group 2 family protein, putative		0.75	6.42E-03	1.14	2.80E-01	0.68	1.89E-02
MSMEG_5962	glycosyl transferase, family protein 2		0.91	1.94E-01	1.20	5.64E-02	0.74	1.19E-01
MSMEG_5963	glycosyl transferase, family protein 2		1.06	6.49E-01	1.00	9.76E-01	0.52	6.17E-02
MSMEG_5964	glycosyl transferase, group 1, putative		0.99	6.99E-01	0.90	2.80E-01	0.81	2.26E-01
MSMEG_5965	hypothetical protein		2.23	6.61E-03	1.12	4.69E-01	0.66	8.46E-02
MSMEG_5966	oxidoreductase, short chain dehydrogenase/reductase family protein		1.06	6.31E-01	1.00	9.99E-01	0.46	3.12E-02
MSMEG_5967	glucose-methanol-choline oxidoreductase		1.36	1.57E-01	1.18	2.09E-01	0.57	6.83E-02
MSMEG_5968	polysaccharide biosynthesis protein		1.59	2.67E-01	0.79	1.13E-01	1.53	9.91E-02
MSMEG_5969	conserved hypothetical protein		2.03	5.79E-03	0.77	2.07E-01	1.17	4.62E-01
MSMEG_5970	probable glutamate-1-semialdehyde 2,1-aminomutase		5.79	8.61E-02	0.72	9.50E-02	0.86	1.30E-01
MSMEG_5971	conserved hypothetical protein		2.04	2.86E-02	0.75	1.63E-01	0.68	5.13E-02
MSMEG_5972	hypothetical protein		1.74	1.14E-02	0.94	5.44E-01	0.82	1.75E-01
MSMEG_5973	hypothetical protein		1.38	3.71E-01	0.92	5.64E-01	0.93	4.28E-01
MSMEG_5974	hypothetical protein		2.51	4.13E-01	0.99	9.48E-01	1.42	2.93E-01
MSMEG_5975	methyltransferase type 12, putative		1.41	3.58E-02	0.98	8.83E-01	1.22	2.20E-01
MSMEG_5976	epimerase/dehydratase		1.38	2.60E-01	0.81	8.59E-02	0.94	2.64E-01
MSMEG_5977	dTDP-4-dehydrorhamnose 3,5-epimerase	*rfbC*	1.14	2.10E-01	1.13	3.15E-01	0.96	2.51E-01
MSMEG_5978	GlcNAc-PI de-N-acetylase family protein		1.36	2.68E-01	0.91	2.09E-02	1.07	6.64E-01
MSMEG_5979	transferase		1.63	1.37E-01	0.93	3.04E-01	1.16	3.81E-01
MSMEG_5980	methyltransferase		0.90	4.93E-01	0.90	2.05E-01	1.15	5.18E-01
MSMEG_5981	glycosyl transferase, putative		0.95	4.23E-01	1.09	6.73E-01	1.07	2.96E-01
MSMEG_5982	UDP-glucose 6-dehydrogenase		1.55	7.22E-02	1.10	4.57E-01	0.92	2.80E-01

MSMEG_5983	glucose-1-phosphate thymidylyltransferase	*rfbA*	1.68	5.74E-02	0.66	1.19E-02	1.03	4.91E-02
MSMEG_5984	undecaprenyl-phosphate galactosephosphotransferase		2.02	6.85E-02	1.61	3.02E-01	0.91	3.61E-01
MSMEG_5985	NAD dependent epimerase/dehydratase family protein		1.41	3.03E-01	1.18	9.54E-02	1.40	2.51E-01
MSMEG_5986	glucosamine--fructose-6-phosphate aminotransferase, isomerizing	*glmS*	0.96	7.57E-01	1.41	1.16E-01	1.14	1.94E-01
MSMEG_5987	two-component regulator		1.31	1.21E-02	1.33	3.00E-01	0.91	2.51E-01
MSMEG_5988	hypothetical protein		1.58	1.07E-02	0.99	9.53E-01	0.53	4.84E-03
MSMEG_5989	hypothetical protein		1.84	4.82E-03	0.97	8.64E-01	0.50	5.53E-03
MSMEG_5990	lipid-transfer protein		2.67	8.35E-04	0.72	1.61E-02	1.32	1.66E-01
MSMEG_5991	MaoC like domain, putative		1.82	1.61E-04	0.92	4.68E-01	1.31	1.27E-01
MSMEG_5992	conserved hypothetical protein		3.00	7.36E-02	0.87	3.43E-01	1.02	3.35E-01
MSMEG_5993	putative acyl-CoA dehydrogenase		3.61	2.94E-02	0.67	6.80E-02	0.95	6.78E-01
MSMEG_5994	putative acyl-CoA dehydrogenase		3.24	2.37E-02	0.83	5.58E-01	1.19	2.38E-01
MSMEG_5995	P450 heme-thiolate protein		5.82	1.38E-02	0.84	4.19E-01	0.84	3.29E-01
MSMEG_5996	acetyl-CoA acetyltransferase		1.58	6.33E-02	0.86	2.73E-01	0.75	1.13E-01
MSMEG_5997	conserved hypothetical protein		1.91	8.34E-02	1.01	8.67E-01	1.06	5.71E-01
MSMEG_5998	conserved hypothetical protein		2.00	1.91E-03	0.98	4.64E-01	1.08	5.42E-01
MSMEG_5999	short chain dehydrogenase		0.90	9.21E-04	0.95	4.65E-02	0.98	8.19E-01
MSMEG_6000	short chain dehydrogenase		1.25	2.14E-01	1.02	8.05E-01	1.07	7.60E-01
MSMEG_6001	enoyl-CoA hydratase		0.75	8.67E-02	0.99	8.74E-01	0.88	1.47E-01
MSMEG_6002	coenzyme A transferase, subunit A		0.80	5.05E-02	0.79	6.70E-02	0.97	3.39E-01
MSMEG_6003	coenzyme A transferase, subunit B		0.98	8.73E-01	1.06	8.23E-01	1.02	9.22E-01
MSMEG_6004	oxidoreductase, 2-nitropropane dioxygenase family protein		1.16	1.58E-01	0.91	3.30E-01	1.33	2.06E-01
MSMEG_6005	conserved hypothetical protein		1.03	7.54E-01	0.97	3.77E-01	1.14	6.08E-01
MSMEG_6006	conserved hypothetical protein		0.42	6.94E-06	0.97	7.64E-01	1.36	2.84E-01
MSMEG_6007	cation diffusion facilitator family transporter; this gene contains a frame shift which is not the result of sequencing error		1.33	3.67E-02	0.82	2.95E-02	1.13	1.23E-01
MSMEG_6007	cation diffusion facilitator family transporter; this gene contains a frame shift which is not the result of sequencing error		0.96	7.51E-01	1.24	2.52E-01	0.94	6.98E-01
MSMEG_6008	acetyl-CoA acetyltransferase		1.07	3.84E-01	0.86	4.56E-02	1.19	3.12E-01
MSMEG_6009	transcriptional regulator, TetR family protein		0.85	2.48E-02	1.07	3.83E-03	1.81	1.07E-01
MSMEG_6010	hypothetical protein		1.17	1.14E-01	0.88	7.08E-02	2.24	3.72E-01
MSMEG_6011	short chain dehydrogenase		0.80	3.11E-02	0.88	1.41E-01	1.02	8.33E-01

MSMEG_6012	putative acyl-CoA dehydrogenase		0.96	6.44E-01	1.05	6.63E-01	1.06	8.37E-01
MSMEG_6013	probable fatty-acid-coa ligase fadd3 (fatty-acid-coa synthetase); this gene contains a frame shift which is not the result of sequencing error; identified by match to protein family HMM	*fadD*	1.69	1.35E-02	0.83	4.87E-02	0.86	2.05E-01
MSMEG_6013	probable fatty-acid-coa ligase fadd3 (fatty-acid-coa synthetase); this gene contains a frame shift which is not the result of sequencing error; identified by match to protein family HMM	*fadD*	1.28	3.86E-02	1.06	4.40E-01	1.01	6.97E-01
MSMEG_6014	putative acyl-CoA dehydrogenase		1.98	4.92E-03	0.90	5.21E-01	0.98	7.38E-01
MSMEG_6015	putative acyl-CoA dehydrogenase		1.74	1.48E-02	0.90	2.35E-01	1.67	3.18E-01
MSMEG_6016	putative acyl-CoA dehydrogenase		2.67	3.03E-02	0.95	6.15E-01	0.85	1.29E-01
MSMEG_6017	aminotransferase, class I		0.93	3.39E-01	0.92	1.17E-01	2.24	1.26E-02
MSMEG_6018	xylose transport system permease protein XylH		2.92	7.40E-04	0.99	9.04E-01	0.13	2.85E-06
MSMEG_6019	ABC-type sugar transport system ATPase component		2.50	4.88E-04	0.88	1.81E-02	0.10	7.88E-07
MSMEG_6020	D-xylose-binding periplasmic protein		2.43	5.08E-05	0.80	7.85E-04	0.16	2.12E-06
MSMEG_6021	xylose isomerase	*xylA*	3.17	2.67E-03	0.73	1.04E-03	0.32	3.97E-02
MSMEG_6022	xylose repressor, ROK-family protein transcriptional regulator		1.42	3.29E-01	1.14	2.24E-01	2.09	3.24E-01
MSMEG_6023	transposase		1.54	1.01E-01	1.10	6.13E-01	1.01	7.98E-01
MSMEG_6024	acetoacetyl-CoA synthase		1.91	2.18E-03	0.70	2.14E-03	0.73	2.42E-01
MSMEG_6025	conserved hypothetical protein		1.23	7.87E-02	1.42	1.19E-01	2.01	1.87E-01
MSMEG_6026	3-hydroxybutyrate dehydrogenase		3.13	1.48E-03	0.80	1.67E-01	0.27	1.61E-04
MSMEG_6027	major facilitator superfamily protein MFS_1		5.32	6.56E-03	0.52	1.55E-02	0.61	3.65E-02
MSMEG_6028	transcriptional regulator, LysR family protein		1.01	9.28E-01	1.13	4.24E-01	1.87	1.09E-01
MSMEG_6029	conserved hypothetical protein		1.75	1.36E-02	0.81	3.92E-03	1.88	5.08E-01
MSMEG_6030	cytochrome p450		4.04	1.40E-02	0.64	2.41E-02	2.94	3.84E-01
MSMEG_6031	carveol dehydrogenase		0.91	4.62E-01	0.99	8.94E-01	1.02	5.70E-01
MSMEG_6032	transcriptional regulator, TetR family protein		1.42	3.84E-02	0.89	2.97E-01	1.13	2.49E-01
MSMEG_6033	hypothetical protein		0.15	1.03E-05	1.01	9.37E-01	0.50	2.93E-02
MSMEG_6034	hypothetical protein		1.16	3.80E-01	1.32	1.59E-01	1.01	6.01E-01
MSMEG_6035	nitrilotriacetate monooxygenase component B		1.59	1.32E-01	0.69	7.54E-02	1.03	8.80E-01
MSMEG_6036	biphenyl-2,3-diol 1,2-dioxygenase		0.98	8.61E-01	0.90	2.56E-01	1.22	1.74E-01
MSMEG_6037	2-hydroxy-6-ketonona-2,4-dienedioic acid hydrolase		0.91	4.64E-01	0.98	8.35E-01	0.88	3.95E-01
MSMEG_6038	pigment production hydroxylase		1.36	5.54E-02	0.94	5.68E-01	0.65	3.34E-02
MSMEG_6039	oxidoreductase, electron transfer component		0.70	2.36E-02	0.85	6.75E-02	1.85	1.59E-01

MSMEG_6040	conserved hypothetical protein		0.94	4.89E-01	1.10	2.95E-01	0.72	1.21E-01
MSMEG_6041	putative acyl-CoA dehydrogenase		2.17	3.96E-03	0.78	1.27E-02	0.87	2.87E-02
MSMEG_6042	transcriptional regulator, TetR family protein		1.71	5.73E-02	1.66	1.80E-01	0.84	5.46E-02
MSMEG_6043	trehalose-phosphatase	*otsB*	0.81	7.38E-02	1.23	3.83E-01	0.92	5.08E-01
MSMEG_6044	periplasmic binding proteins and sugar binding domain of the LacI family protein, putative		0.63	2.10E-03	0.99	8.46E-01	1.88	4.72E-02
MSMEG_6045	ABC heavy metal transporter, inner membrane subunit		1.13	3.86E-01	0.91	5.36E-01	1.18	5.22E-01
MSMEG_6046	cation ABC transporter, ATP-binding protein, putative		1.11	3.61E-01	1.27	4.30E-01	0.88	1.81E-01
MSMEG_6047	cation ABC transporter, periplasmic cation-binding protein, putative		0.59	5.60E-03	0.92	2.09E-01	2.78	1.97E-01
MSMEG_6048	cobalamin synthesis protein/P47K		1.10	6.49E-01	2.23	5.59E-02	1.44	4.85E-01
MSMEG_6049	secreted protein		0.54	1.06E-02	1.09	1.16E-01	1.04	6.02E-01
MSMEG_6050	solute-binding lipoprotein		1.14	1.52E-01	0.98	8.86E-01	0.99	8.65E-01
MSMEG_6051	ABC transporter protein, integral membrane subunit; this gene contains a frame shift which is not the result of sequencing error; identified by match to protein family HMM PF00950		0.73	6.67E-02	1.54	1.86E-02	1.70	1.77E-01
MSMEG_6051	ABC transporter protein, integral membrane subunit; this gene contains a frame shift which is not the result of sequencing error; identified by match to protein family HMM PF00950		1.02	8.78E-01	1.01	8.92E-01	1.02	8.87E-01
MSMEG_6052	ABC transport protein, ATP-binding subunit		0.94	5.29E-01	1.16	1.75E-01	1.19	2.88E-01
MSMEG_6053	cob(II)yrinic acid a,c-diamide reductase	*bluB*	1.01	8.66E-01	0.89	2.25E-01	1.15	2.41E-01
MSMEG_6054	LamB/YcsF		1.46	2.52E-02	0.83	8.68E-02	1.22	4.39E-02
MSMEG_6055	hypothetical protein		3.41	2.16E-01	1.04	3.83E-01	0.90	1.50E-01
MSMEG_6056	transposase; this gene contains a frame shift which is not the result of sequencing error		1.75	2.16E-03	0.90	1.00E-01	4.34	3.58E-01
MSMEG_6056	transposase; this gene contains a frame shift which is not the result of sequencing error		1.47	1.10E-02	1.44	1.66E-01	1.03	6.72E-01
MSMEG_6057	MspD protein		1.23	4.21E-01	1.23	3.24E-01	0.96	6.67E-01
MSMEG_6058	cadmium transporting P-type ATPase		0.75	1.54E-03	1.04	5.86E-01	2.29	8.55E-02
MSMEG_6059	conserved hypothetical protein		0.93	3.94E-02	0.91	1.33E-01	1.39	3.28E-01
MSMEG_6060	permease		1.01	7.63E-01	0.84	8.63E-02	1.63	1.11E-01
MSMEG_6061	conserved hypothetical protein		1.08	6.14E-01	0.97	6.92E-01	1.19	2.36E-01
MSMEG_6062	Fe uptake system permease		0.56	3.17E-04	1.16	2.27E-01	1.52	1.36E-01
MSMEG_6063	Fe uptake system integral membrane protein		0.45	5.26E-04	0.99	5.95E-01	2.65	1.95E-02

MSMEG_6064	lipoprotein		0.75	4.39E-02	0.81	1.20E-01	1.10	6.16E-01
MSMEG_6065	ribosomal protein S18	*rpsR*	1.04	7.55E-01	1.13	1.55E-01	1.03	2.79E-01
MSMEG_6066	30S ribosomal protein S14		1.00	9.91E-01	1.07	4.84E-01	0.92	4.22E-01
MSMEG_6067	ribosomal protein L33	*rpmG*	1.14	4.19E-01	1.07	1.08E-01	0.95	5.18E-01
MSMEG_6068	ribosomal protein L28	*rpmB*	0.96	6.21E-01	1.10	3.08E-01	1.08	3.82E-01
MSMEG_6069	CobW/P47K C- domain protein		1.06	5.76E-01	1.17	2.37E-01	0.94	3.28E-01
MSMEG_6070	ribosomal protein L31	*rpmE*	1.57	1.35E-01	1.18	3.68E-01	0.97	2.55E-01
MSMEG_6071	metallo-beta-lactamase superfamily protein		1.04	6.28E-01	1.00	9.70E-01	0.93	4.97E-01
MSMEG_6072	Citrate transporter		0.56	5.88E-03	0.96	7.90E-01	1.03	8.52E-01
MSMEG_6073	RNA methyltransferase, TrmH family protein, group 3		0.60	1.38E-04	0.81	3.60E-02	1.50	1.61E-02
MSMEG_6074	cysteinyl-tRNA synthetase	*cysS*	0.60	2.25E-04	0.95	2.47E-01	1.36	1.49E-02
MSMEG_6075	2C-methyl-D-erythritol 2,4-cyclodiphosphate synthase	*ispF*	0.22	2.28E-05	1.08	6.95E-01	1.96	5.82E-02
MSMEG_6076	2-C-methyl-D-erythritol 4-phosphate cytidylyltransferase	*ispD*	0.35	7.85E-06	0.83	2.88E-02	1.13	1.26E-01
MSMEG_6077	transcriptional regulator, CarD family protein		0.78	3.27E-02	1.12	2.68E-02	0.58	7.27E-03
MSMEG_6078	LpqE protein		0.39	1.85E-05	0.92	3.25E-01	2.21	2.82E-03
MSMEG_6079	DNA repair protein RadA	*radA*	0.80	1.14E-01	1.25	4.87E-01	1.12	3.17E-01
MSMEG_6080	conserved hypothetical protein		1.16	1.84E-02	0.92	3.09E-01	0.70	6.45E-03
MSMEG_6081	conserved hypothetical protein		1.26	2.32E-01	1.00	9.69E-01	1.46	1.48E-01
MSMEG_6082	carbonic anhydrase		1.26	1.38E-01	1.12	3.91E-01	1.63	2.69E-04
MSMEG_6083	base excision DNA repair protein, HhH-GPD family protein		1.34	5.10E-01	0.69	1.85E-01	1.00	9.52E-01
MSMEG_6084	AraC-family transcriptional regulator; this gene contains a frame shift which is not the result of sequencing error; identified by match to protein family HMM PF00165		1.73	5.49E-04	0.87	2.17E-01	0.79	9.33E-02
MSMEG_6085	hydrolase, alpha/beta hydrolase fold family protein		0.79	3.18E-01	0.99	9.25E-01	1.25	3.46E-01
MSMEG_6086	antibiotic biosynthesis monooxygenase domain protein		1.01	8.53E-01	0.90	3.20E-02	0.97	3.19E-01
MSMEG_6087	beta-lactamase		0.46	1.40E-05	0.95	6.49E-01	1.28	1.66E-01
MSMEG_6088	phosphoglycerate mutase		0.33	2.11E-05	1.33	8.17E-02	0.80	3.53E-03
MSMEG_6089	conserved hypothetical protein		0.72	7.39E-03	1.17	1.16E-01	0.90	6.25E-01
MSMEG_6090	conserved hypothetical protein		1.13	2.58E-01	0.86	1.10E-01	0.56	6.08E-03
MSMEG_6091	negative regulator of genetic competence ClpC/mecB		0.73	4.18E-02	0.95	5.32E-01	1.93	1.43E-03

MSMEG_6092	Lsr2 protein		1.27	1.21E-01	0.96	5.74E-01	1.25	1.47E-01
MSMEG_6093	hypothetical protein		0.99	9.87E-01	0.46	1.51E-02	0.90	3.42E-01
MSMEG_6094	lysyl-tRNA synthetase	*lysS*	0.44	4.78E-06	1.01	8.90E-01	1.47	1.19E-02
MSMEG_6095	D-amino acid deaminase		0.88	3.03E-01	1.05	6.27E-01	0.98	4.57E-01
MSMEG_6096	Bvg accessory factor family protein		0.43	8.46E-05	0.67	4.41E-04	1.35	1.43E-02
MSMEG_6097	pantoate--beta-alanine ligase	*panC*	0.50	7.40E-04	0.69	2.15E-03	0.96	8.56E-01
MSMEG_6098	chalcone/stilbene synthase family protein		0.47	2.19E-04	0.58	3.53E-03	2.95	2.30E-01
MSMEG_6099	probable conserved transmembrane protein rich in alanine		1.59	6.61E-04	0.81	3.06E-02	0.71	2.88E-02
MSMEG_6100	conserved hypothetical protein		0.43	1.64E-03	0.90	3.28E-01	1.13	2.82E-01
MSMEG_6101	2-amino-4-hydroxy-6-hydroxymethyldihydropteridine pyrophosphokinase	*folK*	0.69	9.73E-04	0.85	1.45E-01	1.51	3.71E-02
MSMEG_6102	dihydroneopterin aldolase	*folB*	0.63	1.75E-03	0.88	6.75E-02	2.10	7.15E-02
MSMEG_6103	dihydropteroate synthase	*folP*	0.69	5.09E-05	0.93	2.53E-01	2.04	6.10E-02
MSMEG_6104	GTP cyclohydrolase I	*folE*	0.92	9.35E-02	0.86	1.52E-01	2.01	1.47E-03
MSMEG_6105	cell division protein		1.08	1.53E-01	0.89	3.73E-01	1.06	1.98E-01
MSMEG_6106	epoxide hydrolase		2.06	8.63E-02	0.91	3.17E-01	0.21	4.58E-05
MSMEG_6107	limonene 1,2-monooxygenase		1.56	5.74E-03	0.98	6.62E-01	0.29	6.17E-04
MSMEG_6108	zinc-binding dehydrogenase		1.04	1.54E-01	0.87	9.64E-03	0.57	2.36E-03
MSMEG_6109	LpqG protein		0.75	8.54E-02	0.78	1.73E-01	0.63	4.19E-03
MSMEG_6110	hypoxanthine phosphoribosyltransferase	*hpt*	1.15	7.28E-02	0.99	7.35E-01	0.75	6.28E-02
MSMEG_6111	tRNA(Ile)-lysidine synthetase	*tilS*	0.59	1.33E-03	1.05	7.13E-01	1.57	1.70E-01
MSMEG_6112	conserved hypothetical protein		0.84	2.10E-02	0.89	1.00E-01	1.82	1.00E-01
MSMEG_6113	D-alanyl-D-alanine carboxypeptidase/D-alanyl-D-alanine-endopeptidase	*dacB*	0.62	1.02E-04	0.89	4.30E-01	2.94	1.50E-01
MSMEG_6114	inorganic pyrophosphatase	*ppa*	0.34	3.62E-05	1.14	4.00E-01	0.64	3.57E-02
MSMEG_6115	phosphoglycerate dehydrogenase		0.96	7.52E-01	10.70	3.71E-01	1.03	8.93E-01
MSMEG_6116	conserved hypothetical protein		0.19	2.54E-04	2.65	3.43E-01	0.34	1.01E-03
MSMEG_6117	glucarate dehydratase		1.02	8.56E-01	1.26	4.35E-01	1.14	2.27E-01
MSMEG_6118	IclR-family protein transcriptional regulator		1.45	1.26E-01	1.20	4.24E-01	1.03	6.33E-01
MSMEG_6119	conserved hypothetical protein		0.97	7.48E-01	1.02	8.73E-01	1.01	7.12E-01
MSMEG_6120	peptide transport system ATP-binding protein		1.75	1.27E-01	0.90	3.36E-01	1.01	9.16E-01
MSMEG_6121	dipeptide transport ATP-binding protein DppD		1.94	1.22E-01	0.86	2.33E-01	1.07	5.06E-01
MSMEG_6122	ABC transporter, membrane spanning protein		3.29	3.27E-02	0.83	2.32E-01	1.27	4.97E-01
MSMEG_6123	ABC transporter, membrane spanning protein		2.73	2.79E-02	0.81	1.15E-02	1.02	2.05E-01
MSMEG_6124	putative ABC transporter periplasmic oligopeptide-binding protein		2.74	4.08E-02	0.98	7.56E-01	0.93	3.84E-01

MSMEG_6125	transcriptional regulatory protein		1.07	7.82E-01	0.86	2.56E-01	1.24	3.43E-01
MSMEG_6126	D-isomer specific 2-hydroxyacid dehydrogenase		0.99	9.09E-01	0.97	8.61E-01	1.49	4.68E-02
MSMEG_6127	anti-anti-sigma factor		0.89	3.15E-02	1.06	4.08E-01	1.23	1.31E-01
MSMEG_6128	two-component system response regulator		0.93	2.01E-01	0.82	4.89E-02	1.23	1.56E-01
MSMEG_6129	conserved hypothetical protein		0.60	1.17E-05	1.19	6.70E-02	1.06	2.02E-01
MSMEG_6130	two-component sensor protein		1.03	7.31E-01	0.84	5.64E-02	1.13	5.54E-02
MSMEG_6131	magnesium or manganese-dependent protein phosphatase		1.21	3.40E-02	0.81	1.63E-01	1.33	1.29E-01
MSMEG_6132	glucarate dehydratase		0.99	9.33E-01	1.08	2.27E-01	1.16	4.82E-01
MSMEG_6133	5-dehydro-4-deoxyglucarate dehydratase		1.37	3.12E-01	0.95	3.56E-01	1.03	7.77E-01
MSMEG_6134	NADP-dependent fatty aldehyde dehydrogenase		0.94	6.66E-01	0.99	8.45E-01	1.66	4.20E-01
MSMEG_6135	hypothetical protein		0.74	9.72E-02	2.55	2.75E-01	1.35	1.50E-01
MSMEG_6136	membrane protein, TerC family protein		1.12	3.31E-01	0.85	8.96E-02	1.09	5.95E-01
MSMEG_6137	non-ribosomal peptide synthetase		0.60	1.80E-02	0.96	7.62E-01	3.48	9.55E-02
MSMEG_6138	metallopeptidase		0.41	1.25E-03	1.07	3.04E-01	3.11	6.81E-03
MSMEG_6139	putative HTH-type transcriptional regulator		4.21	7.09E-02	0.90	1.71E-01	1.77	3.90E-01
MSMEG_6140	membrane protein		0.76	1.31E-01	0.95	6.37E-01	0.93	6.76E-01
MSMEG_6141	N-acyl-D-glutamate amidohydrolase		3.35	2.55E-03	0.79	1.49E-01	1.01	9.72E-01
MSMEG_6142	nucleoside-diphosphate-sugar epimerase		0.44	3.64E-05	0.82	4.84E-03	0.89	5.02E-01
MSMEG_6143	conserved hypothetical protein		0.82	9.64E-02	0.93	3.89E-01	1.00	9.68E-01
MSMEG_6144	pe family protein		1.24	1.14E-01	1.38	8.35E-02	0.61	5.04E-02
MSMEG_6145	hypothetical protein		1.77	2.11E-02	1.23	2.38E-01	0.87	2.70E-01
MSMEG_6146	ISMsm3, transposase		3.29	4.84E-05	0.46	7.88E-03	1.48	1.89E-01
MSMEG_6146	ISMsm3, transposase		2.41	1.03E-02	0.53	2.54E-02	2.13	2.08E-01
MSMEG_6147	conserved hypothetical protein		1.49	2.75E-02	0.88	1.15E-01	6.31	3.60E-01
MSMEG_6150	4-carboxymuconolactone decarboxylase		0.98	9.03E-01	1.03	7.98E-01	0.73	6.80E-02
MSMEG_6151	alpha/beta hydrolase fold-1		0.64	5.54E-02	1.22	1.20E-01	0.69	9.95E-03
MSMEG_6153	DNA polymerase III subunit delta		0.56	1.03E-03	0.94	5.22E-01	1.49	1.61E-01
MSMEG_6154	adenylate cyclase, family protein 3		0.68	1.49E-04	0.84	5.45E-03	1.19	3.48E-01
MSMEG_6157	DNA topoisomerase I	*topA*	0.81	2.64E-02	0.93	2.31E-01	0.68	2.45E-03
MSMEG_6158	conserved hypothetical protein		0.57	1.82E-05	0.83	1.64E-01	0.79	1.63E-02
MSMEG_6159	conserved domain protein		1.64	4.49E-02	0.75	2.83E-02	0.76	9.18E-02
MSMEG_6160	ATP-dependent rna helicase, dead/deah box family protein		0.70	1.63E-02	1.04	6.63E-01	1.48	1.67E-02
MSMEG_6163	conserved hypothetical protein		1.32	1.07E-01	0.86	4.75E-02	2.51	2.69E-03
MSMEG_6164	conserved hypothetical protein		1.04	7.94E-01	0.95	5.29E-01	1.09	4.17E-01
MSMEG_6165	conserved hypothetical protein		1.81	1.12E-02	0.83	6.53E-03	1.23	1.48E-01
MSMEG_6166	hypothetical protein		1.49	2.88E-01	1.49	9.81E-02	0.85	2.72E-01

MSMEG_6167	bacterial type II secretion system protein F domain		1.23	1.87E-01	1.08	2.88E-01	1.36	4.15E-01
MSMEG_6168	bacterial type II secretion system protein F domain		1.21	1.40E-02	1.13	5.43E-01	1.12	4.48E-01
MSMEG_6169	type II/IV secretion system protein		1.17	4.22E-01	0.96	6.44E-01	1.01	8.84E-01
MSMEG_6170	hypothetical protein		0.85	2.51E-01	1.19	2.75E-01	1.17	4.12E-01
MSMEG_6171	conserved hypothetical protein		2.17	1.89E-01	1.11	3.94E-01	0.94	5.40E-01
MSMEG_6172	hypothetical protein		0.92	5.22E-01	1.01	9.68E-01	1.03	6.82E-01
MSMEG_6173	morphological differentiation-associated protein		1.60	2.23E-03	0.70	5.97E-02	1.45	7.62E-02
MSMEG_6174	transcriptional regulator, IclR family protein		1.21	7.57E-02	0.98	8.65E-01	1.17	2.74E-01
MSMEG_6175	2-keto-3-deoxy-galactonokinase		1.13	6.46E-01	0.89	2.90E-01	1.00	8.34E-01
MSMEG_6176	2-dehydro-3-deoxy-6-phosphogalactonate		1.64	3.66E-02	1.03	8.28E-01	1.06	6.09E-01
MSMEG_6177	galactonate dehydratase		1.86	2.29E-01	1.05	5.53E-01	1.11	4.15E-01
MSMEG_6178	D-galactonate transporter		1.24	2.17E-01	0.86	8.32E-02	0.93	3.06E-01
MSMEG_6179	acetyl-coenzyme A synthetase		1.15	6.06E-02	1.04	5.04E-01	0.34	1.89E-03
MSMEG_6180	secreted protein		0.98	7.94E-01	0.82	1.64E-01	1.18	1.40E-01
MSMEG_6181	conserved hypothetical protein		0.69	6.13E-02	0.88	6.00E-01	1.09	2.70E-01
MSMEG_6182	conserved hypothetical protein		1.20	7.54E-01	0.94	5.41E-01	0.81	1.99E-01
MSMEG_6183	serine protease		0.85	1.86E-01	0.93	2.81E-01	1.46	1.31E-01
MSMEG_6184	hydrolase, alpha/beta fold family protein		0.63	1.33E-02	1.06	5.19E-01	1.20	3.88E-01
MSMEG_6185	NTP pyrophosphohydrolase		0.39	5.11E-05	0.95	3.33E-01	1.66	7.63E-02
MSMEG_6186	conserved hypothetical protein		0.56	3.19E-05	1.13	4.38E-01	1.97	8.84E-02
MSMEG_6187	endonuclease III	*nth*	0.86	5.54E-02	0.99	8.51E-01	0.91	3.50E-01
MSMEG_6188	conserved hypothetical protein		1.79	1.80E-02	0.83	1.97E-01	0.94	8.63E-01
MSMEG_6189	transcriptional regulator, Crp/Fnr family protein		1.02	8.77E-01	0.82	1.29E-01	0.80	2.49E-02
MSMEG_6190	metallo-beta-lactamase family protein		0.27	1.40E-05	1.05	4.27E-01	1.62	3.50E-02
MSMEG_6191	translation initiation inhibitor		0.53	9.04E-05	0.87	2.08E-01	1.00	8.77E-01
MSMEG_6192	conserved hypothetical protein		0.60	5.68E-04	0.94	4.70E-01	0.96	6.68E-01
MSMEG_6193	anion-transporting ATPase		0.55	2.72E-04	0.98	7.76E-01	1.41	9.22E-02
MSMEG_6194	beta-lactamase related protein		0.90	6.45E-01	1.89	2.42E-01	1.22	4.11E-01
MSMEG_6195	ion-transporting ATPase		0.51	2.03E-04	0.91	8.25E-02	1.10	5.61E-01
MSMEG_6196	gaba permease		0.98	8.22E-01	1.32	1.23E-01	1.75	3.77E-01
MSMEG_6197	diaminopimelate decarboxylase		0.89	4.54E-01	1.12	3.91E-01	0.96	6.27E-01
MSMEG_6198	DNA-binding protein, putative		0.55	2.83E-02	1.31	2.49E-01	1.88	1.36E-01
MSMEG_6199	Transcription factor WhiB		4.36	4.93E-02	1.12	6.25E-01	0.05	2.90E-07
MSMEG_6200	hypothetical protein		1.22	2.34E-01	1.46	9.78E-02	3.07	3.06E-01
MSMEG_6201	Transglycosylase		0.98	6.39E-01	0.87	2.81E-01	0.94	6.91E-01
MSMEG_6202	secreted protein		0.70	7.32E-05	0.93	2.24E-01	1.18	4.28E-02

MSMEG_6203	cysteine synthase/cystathionine beta-synthase family protein		0.51	2.78E-03	1.19	2.91E-02	2.09	1.64E-02
MSMEG_6205	hypothetical protein		11.03	1.23E-01	0.23	6.47E-03	1.11	7.62E-01
MSMEG_6206	serine/threonine protein kinase PksC		1.14	5.26E-01	1.07	5.22E-01	2.39	1.55E-01
MSMEG_6207	conserved hypothetical protein		1.72	2.77E-03	0.76	1.12E-02	0.85	9.61E-02
MSMEG_6208	palmitoyl-CoA hydrolase		0.37	4.55E-04	0.95	6.94E-01	2.77	1.43E-01
MSMEG_6209	conserved hypothetical protein		1.41	2.53E-01	0.70	1.48E-02	33.40	3.57E-03
MSMEG_6210	conserved hypothetical protein		0.88	5.42E-01	1.40	1.39E-01	7.52	3.52E-02
MSMEG_6211	hypothetical protein		1.41	1.76E-01	1.99	2.00E-02	0.64	7.04E-02
MSMEG_6212	hemerythrin HHE cation binding domain subfamily protein, putative		2.73	1.69E-01	2.92	2.15E-02	0.36	3.63E-04
MSMEG_6213	Manganese containing catalase		1.39	3.35E-01	2.85	4.02E-02	0.44	2.83E-03
MSMEG_6214	GatB/Yqey domain protein		1.32	6.51E-03	1.02	7.31E-01	1.28	4.32E-01
MSMEG_6215	conserved hypothetical protein		0.73	1.06E-02	1.10	2.90E-01	13.07	3.78E-01
MSMEG_6216	integral membrane protein		1.91	1.21E-02	0.75	1.62E-02	1.57	1.81E-02
MSMEG_6217	integral membrane protein		1.20	6.03E-02	0.76	1.45E-02	2.24	1.01E-03
MSMEG_6218	secreted protein		0.87	2.51E-02	1.07	6.94E-01	3.28	1.09E-03
MSMEG_6219	ATPase family protein associated with various cellular activities (AAA)		0.93	4.76E-02	0.81	3.35E-02	1.29	1.77E-02
MSMEG_6220	lipoprotein		0.69	1.30E-02	0.85	1.34E-02	1.57	1.48E-01
MSMEG_6221	integral membrane protein		0.64	5.74E-04	0.91	4.53E-01	1.48	4.70E-02
MSMEG_6222	integral membrane protein		1.29	4.24E-02	0.87	1.03E-01	0.95	6.67E-01
MSMEG_6223	TetR family protein transcriptional repressor LfrR		0.69	5.69E-03	1.09	6.94E-01	1.51	8.31E-02
MSMEG_6224	Retinal pigment epithelial membrane protein		1.47	3.84E-02	1.16	2.09E-01	0.29	1.15E-04
MSMEG_6225	proton antiporter efflux pump		0.57	1.79E-03	1.30	1.13E-01	1.09	6.62E-01
MSMEG_6226	transcriptional regulator, PadR family protein domain protein		1.74	5.01E-01	1.10	5.33E-01	1.06	5.58E-01
MSMEG_6227	transcriptional regulator, PadR family protein		1.02	8.86E-01	0.79	1.34E-01	4.83	1.02E-01
MSMEG_6228	hypothetical protein		0.45	1.25E-04	0.99	8.82E-01	4.42	6.12E-02
MSMEG_6229	glycerol kinase	*glpK*	2.97	3.90E-01	1.29	5.75E-01	0.84	4.34E-01
MSMEG_6230	probable acyltransferase		0.73	6.54E-02	1.24	3.04E-01	1.04	2.88E-01
MSMEG_6231	conserved hypothetical protein		2.28	2.31E-04	0.84	4.01E-01	1.02	8.00E-01
MSMEG_6232	catalase KatA	*katA*	0.96	8.93E-01	4.15	1.57E-01	0.59	9.27E-02
MSMEG_6233	conserved hypothetical protein		1.02	8.37E-01	1.12	3.89E-01	0.93	6.35E-01
MSMEG_6234	CBS domain pair protein		3.40	6.95E-04	1.10	3.94E-01	0.34	1.27E-05
MSMEG_6235	thiopurine S-methyltransferase (tpmt) superfamily protein		1.84	2.04E-02	0.73	7.75E-02	1.25	1.21E-01
MSMEG_6236	two-component system, regulatory protein		2.41	7.57E-03	1.05	7.10E-01	0.06	1.36E-07

MSMEG_6237	conserved hypothetical protein		2.65	1.75E-01	2.03	2.40E-01	0.72	2.44E-01
MSMEG_6238	putative two-component system sensor kinase		3.07	7.66E-03	1.26	9.68E-02	0.05	7.38E-09
MSMEG_6239	1,3-propanediol dehydrogenase		3.50	4.15E-03	1.04	6.55E-01	0.20	4.60E-03
MSMEG_6240	conserved hypothetical protein		2.04	2.56E-01	1.91	1.54E-01	0.25	1.61E-02
MSMEG_6241	ATPase associated with various cellular activities, AAA-5		2.84	1.39E-01	1.67	1.35E-01	0.09	2.11E-05
MSMEG_6242	alcohol dehydrogenase, iron-containing		2.38	4.25E-02	2.05	7.40E-02	0.10	6.34E-06
MSMEG_6243	response regulator receiver domain protein		0.65	1.70E-02	1.32	2.74E-02	1.16	2.61E-01
MSMEG_6244	TetR-family protein transcriptional regulator		0.98	8.23E-01	1.13	3.02E-01	1.05	7.77E-01
MSMEG_6245	chloramphenicol resistance protein		1.51	1.55E-01	4.07	3.46E-01	1.14	1.64E-01
MSMEG_6246	pyridoxal-phosphate-dependent transferase		1.14	5.91E-02	1.89	1.46E-01	2.48	3.10E-01
MSMEG_6247	conserved hypothetical protein		1.31	6.65E-02	0.90	5.19E-02	2.11	3.04E-02
MSMEG_6248	conserved hypothetical protein		1.62	2.44E-03	0.92	3.87E-01	1.53	1.04E-01
MSMEG_6249	conserved hypothetical protein		1.49	7.29E-04	0.87	3.19E-02	1.37	6.42E-02
MSMEG_6250	glutamate--cysteine ligase		1.73	1.86E-04	0.70	2.29E-02	1.24	6.95E-02
MSMEG_6251	conserved hypothetical protein		0.77	4.71E-02	0.80	2.07E-03	2.24	1.42E-02
MSMEG_6252	conserved hypothetical protein		2.85	2.66E-03	0.80	2.04E-01	0.43	1.44E-03
MSMEG_6253	fur family protein transcriptional regulator		0.43	8.17E-05	1.61	2.71E-02	6.29	1.66E-01
MSMEG_6254	hypothetical protein		2.24	5.43E-04	0.87	3.03E-01	2.86	5.68E-02
MSMEG_6255	conserved hypothetical protein		0.37	2.46E-04	1.01	9.19E-01	1.62	3.49E-01
MSMEG_6256	aspartate-semialdehyde dehydrogenase	*asd*	0.56	1.12E-05	0.85	1.10E-01	1.08	5.16E-01
MSMEG_6257	asparate kinase, monofunctional class		0.38	3.64E-05	0.97	7.53E-01	1.13	2.81E-01
MSMEG_6258	nitroreductase family protein		0.62	4.95E-04	0.97	7.66E-01	2.04	1.29E-02
MSMEG_6259	ammonium transporter	*amt*	0.19	8.79E-05	1.35	1.68E-01	0.96	7.90E-01
MSMEG_6260	glutamine synthetase, type III	*glnT*	0.60	6.48E-02	1.08	5.87E-01	1.02	8.21E-01
MSMEG_6261	glutamine amidotransferase, class II		0.60	1.41E-01	1.10	4.92E-01	0.96	3.55E-01
MSMEG_6262	FwdC/FmdC family protein		0.58	5.20E-05	1.11	2.04E-01	0.95	2.35E-01
MSMEG_6263	glutamate synthase family protein		0.56	5.54E-03	0.94	4.59E-01	1.01	8.21E-01
MSMEG_6264	putative oxidoreductase		0.79	2.25E-01	0.92	5.81E-01	1.02	7.64E-01
MSMEG_6265	transcriptional regulator		0.76	1.46E-02	1.18	1.40E-01	0.97	7.80E-01
MSMEG_6266	thiocyanate hydrolase beta subunit		1.94	1.46E-02	1.06	6.15E-01	0.10	4.58E-06
MSMEG_6267	thiocyanate hydrolase gamma subunit		2.05	1.86E-02	1.19	3.05E-01	0.14	5.40E-06
MSMEG_6268	putative ScnB protein		2.10	6.22E-02	1.14	3.91E-01	0.19	1.28E-05
MSMEG_6269	magnesium transporter	*mgtE*	0.87	4.12E-01	0.83	1.83E-01	0.94	2.20E-01
MSMEG_6270	hypothetical protein		1.00	9.90E-01	0.68	1.75E-01	1.15	2.21E-01
MSMEG_6271	2-isopropylmalate synthase	*leuA*	0.81	1.84E-02	0.87	1.01E-01	1.42	5.24E-02
MSMEG_6272	NAD-glutamate dehydrogenase		1.36	1.13E-03	0.98	8.84E-01	1.59	2.19E-01
MSMEG_6273	integral membrane protein		0.80	1.20E-01	0.96	6.68E-01	1.06	2.29E-01

MSMEG_6274	conserved hypothetical protein		0.98	8.73E-01	1.09	4.70E-01	1.06	6.33E-01
MSMEG_6275	DNA polymerase III subunit epsilon		1.25	3.19E-01	1.01	8.98E-01	2.48	1.21E-01
MSMEG_6276	mur ligase family protein		0.45	4.08E-04	0.78	3.50E-02	1.60	1.77E-02
MSMEG_6277	cobyric acid synthase		0.60	6.16E-04	0.81	5.59E-02	2.30	9.61E-02
MSMEG_6278	metallo-beta-lactamase superfamily protein, putative		2.01	6.37E-03	0.75	8.32E-03	1.23	8.74E-02
MSMEG_6279	recombination protein RecR	*recR*	0.66	1.72E-03	0.83	1.55E-02	1.00	9.81E-01
MSMEG_6280	conserved hypothetical protein		0.76	5.85E-04	0.95	5.29E-01	0.92	3.72E-01
MSMEG_6281	N-acetylmuramoyl-L-alanine amidase		1.74	6.83E-02	0.78	3.04E-01	1.21	6.17E-01
MSMEG_6282	KanY protein		0.55	1.26E-04	0.95	4.54E-02	1.28	1.15E-02
MSMEG_6283	FAD binding domain protein		1.06	4.83E-01	0.95	4.49E-01	1.18	1.07E-01
MSMEG_6284	cyclopropane-fatty-acyl-phospholipid synthase		0.94	2.74E-01	0.81	1.07E-01	2.62	1.38E-03
MSMEG_6285	DNA polymerase III gamma/tau subunit		0.47	4.06E-05	1.17	6.07E-01	0.79	1.29E-01
MSMEG_6286	aspartate transaminase		0.37	3.56E-05	0.85	1.43E-01	1.99	8.74E-02
MSMEG_6288	conserved hypothetical protein		0.53	1.17E-03	0.90	1.40E-01	1.13	3.10E-01
MSMEG_6289	Trypsin		1.16	3.95E-01	0.81	1.42E-01	1.58	6.82E-03
MSMEG_6290	putative DNA-binding protein		0.98	6.40E-01	0.97	1.92E-01	3.06	1.54E-01
MSMEG_6291	D-amino-acid dehydrogenase		4.50	6.57E-02	0.80	4.73E-02	3.93	6.97E-02
MSMEG_6292	transcription elongation factor GreA		1.09	6.73E-01	0.96	6.81E-01	2.60	9.05E-02
MSMEG_6293	transcriptional regulatory protein		0.65	5.47E-03	0.90	4.40E-02	1.02	8.15E-01
MSMEG_6294	caib/baif family protein		0.96	7.35E-01	0.88	6.93E-02	1.00	9.92E-01
MSMEG_6295	shikimate transporter		0.98	8.65E-01	0.86	1.78E-01	0.97	2.40E-01
MSMEG_6296	5-exo-alcohol dehydrogenase		1.22	4.25E-01	0.94	5.31E-01	0.62	7.84E-02
MSMEG_6297	aldehyde dehydrogenase		1.26	9.01E-02	0.91	4.02E-01	1.04	6.36E-01
MSMEG_6298	malyl-CoA lyase		0.98	9.05E-01	0.97	7.06E-01	0.94	3.13E-01
MSMEG_6299	glyoxylate reductase		1.49	3.67E-01	0.91	4.32E-01	0.98	6.52E-01
MSMEG_6300	transcriptional regulator, GntR family protein		1.32	1.22E-03	0.87	9.59E-02	1.34	4.58E-01
MSMEG_6301	DNA polymerase LigD, polymerase domain	*ligD*	1.61	4.15E-03	0.92	3.81E-01	1.36	7.80E-02
MSMEG_6302	DNA ligase		1.78	4.32E-02	0.91	1.59E-01	0.95	6.47E-01
MSMEG_6303	para-nitrobenzyl esterase		1.69	8.12E-02	0.89	4.74E-01	1.68	9.07E-02
MSMEG_6304	DNA ligase		1.00	9.89E-01	0.93	3.22E-01	1.06	6.85E-01
MSMEG_6305	conserved hypothetical protein		1.19	4.64E-01	1.49	1.29E-01	1.06	9.19E-01
MSMEG_6306	glutamyl-tRNA synthetase	*gltX*	0.77	5.32E-02	1.25	1.65E-02	1.13	2.96E-01
MSMEG_6307	glutamine-binding periplasmic protein/glutamine transport system permease protein		1.12	2.40E-02	1.25	2.48E-01	0.89	5.82E-01
MSMEG_6308	hypothetical protein		1.07	7.76E-01	1.09	6.80E-01	1.01	7.97E-01
MSMEG_6309	ABC transporter, ATP-binding protein		0.70	1.67E-02	1.25	3.92E-01	0.83	3.67E-01
MSMEG_6310	4-hydroxybutyrate coenzyme A transferase		0.93	5.54E-01	0.71	5.12E-02	1.44	2.34E-01

MSMEG_6311	saccharopine dehydrogenase	*lys1*	0.92	5.02E-01	0.88	2.45E-01	1.07	4.24E-01
MSMEG_6312	cytochrome P450 107B1		2.12	1.90E-02	0.64	3.52E-02	0.72	1.07E-01
MSMEG_6313	queuine tRNA-ribosyltransferase	*tgt*	0.58	3.73E-03	0.91	2.82E-01	1.18	7.15E-02
MSMEG_6314	haloalkane dehalogenase 1		2.32	5.16E-02	1.18	3.97E-02	0.69	7.37E-02
MSMEG_6315	lipoprotein LpqH		0.41	5.42E-05	0.77	1.20E-01	1.04	7.89E-01
MSMEG_6316	lipoprotein LpqH		3.53	2.86E-02	1.35	5.92E-02	0.40	1.16E-02
MSMEG_6317	lipolytic enzyme, G-D-S-L		1.06	5.95E-01	1.04	5.16E-01	0.46	3.49E-05
MSMEG_6318	putative diol dehydratase reactivation protein		1.54	4.04E-01	0.97	5.62E-01	0.83	1.14E-01
MSMEG_6319	penicillin-binding protein, transpeptidase		0.64	6.32E-04	0.96	4.13E-01	0.82	1.29E-01
MSMEG_6320	diol dehydrase gamma subunit		1.56	3.20E-01	0.91	2.53E-01	0.63	1.07E-01
MSMEG_6321	glycerol dehydratase large subunit		1.00	9.94E-01	1.13	2.15E-01	1.06	4.91E-01
MSMEG_6322	bifunctional wax ester synthase/acyl-CoA diacylglycerol acyltransferase		1.89	3.31E-03	1.16	2.30E-01	0.66	3.39E-01
MSMEG_6323	hypothetical protein		24.55	4.42E-02	0.40	9.50E-02	0.89	6.34E-01
MSMEG_6324	peroxidase		19.76	2.09E-02	0.30	2.66E-02	0.77	2.82E-01
MSMEG_6325	conserved hypothetical protein		18.72	3.46E-02	0.29	3.09E-02	1.13	7.29E-01
MSMEG_6327	cytidine and deoxycytidylate deaminase family protein		0.70	5.66E-02	1.20	3.33E-01	1.11	1.85E-01
MSMEG_6328	conserved hypothetical protein		1.16	4.90E-02	0.85	4.60E-02	0.61	9.03E-03
MSMEG_6329	conserved hypothetical protein		0.89	4.60E-02	0.82	2.84E-02	0.49	1.74E-04
MSMEG_6330	prephenate dehydrogenase		1.00	9.92E-01	0.70	7.85E-03	1.34	1.14E-02
MSMEG_6331	ABC transporter, permease protein ProZ		2.06	1.49E-01	1.04	6.64E-01	0.98	7.36E-01
MSMEG_6332	amino acid ABC transporter, permease protein		0.85	2.68E-02	0.96	5.79E-01	0.95	1.54E-01
MSMEG_6333	amino acid ABC transporter, ATP-binding protein		1.48	2.35E-01	1.40	4.44E-01	1.06	3.69E-01
MSMEG_6334	ABC transporter, quaternary amine uptake transporter (QAT) family protein, substrate-binding protein		1.07	6.89E-01	0.98	8.17E-01	1.01	7.78E-01
MSMEG_6335	hypothetical protein		1.18	2.26E-01	1.22	3.01E-01	2.11	4.02E-01
MSMEG_6336	conserved hypothetical protein		1.24	1.49E-02	0.91	4.32E-01	1.15	2.22E-01
MSMEG_6337	Phosphotransferase enzyme family protein		0.45	2.38E-04	1.05	7.00E-01	1.18	6.08E-01
MSMEG_6338	phosphoglycerate mutase family protein, putative		0.59	3.44E-03	0.92	1.73E-01	0.92	2.70E-01
MSMEG_6339	metallo-beta-lactamase family protein		1.52	2.64E-01	1.04	8.03E-01	1.27	2.78E-01
MSMEG_6340	short-chain dehydrogenase/reductase SDR		1.47	2.91E-02	0.89	1.70E-01	1.06	2.35E-01
MSMEG_6341	6-phosphogluconate dehydrogenase, NAD-binding		1.57	5.80E-02	0.92	3.33E-01	1.32	2.96E-01
MSMEG_6342	oxidoreductase		1.95	5.92E-02	0.95	4.58E-01	0.97	3.23E-01
MSMEG_6343	conserved hypothetical protein		0.88	8.71E-02	0.92	4.37E-01	1.44	3.27E-02

MSMEG_6344	transcriptional regulator		1.21	5.84E-02	1.04	6.42E-01	0.98	4.54E-01
MSMEG_6345	conserved hypothetical protein		0.26	2.20E-05	1.10	6.16E-01	8.18	5.75E-03
MSMEG_6346	hypothetical protein		0.40	4.19E-04	1.15	5.12E-01	3.02	1.54E-01
MSMEG_6347	hypothetical protein		1.27	4.10E-01	0.87	2.44E-02	1.15	3.26E-01
MSMEG_6348	hypothetical protein		0.67	2.36E-01	1.34	2.81E-01	0.74	1.13E-01
MSMEG_6351	histidinol-phosphate aminotransferase	*hisC*	0.69	3.18E-03	0.94	4.01E-01	1.42	1.64E-02
MSMEG_6352	conserved hypothetical protein		0.37	1.03E-04	0.82	1.12E-01	1.70	2.32E-02
MSMEG_6353	delta3,5-delta2,4-dienoyl-CoA isomerase		0.50	8.62E-05	0.99	9.01E-01	1.33	1.01E-01
MSMEG_6354	serine esterase, cutinase family protein		0.53	1.05E-02	1.97	3.71E-02	0.90	4.15E-01
MSMEG_6355	hypothetical protein		0.70	3.06E-01	1.80	1.79E-01	0.65	1.01E-01
MSMEG_6356	probable transcriptional regulator, AraC family protein, putative		1.52	2.55E-02	0.74	1.09E-02	0.63	9.24E-03
MSMEG_6357	hypothetical protein		0.97	8.16E-01	1.09	2.34E-01	1.44	1.89E-01
MSMEG_6358	mesocentin		0.73	1.42E-01	1.01	9.71E-01	1.21	3.12E-01
MSMEG_6359	trypsin domain protein		0.64	1.76E-02	1.04	2.74E-01	1.27	1.18E-01
MSMEG_6361	transcriptional regulator, MarR family protein		1.14	3.44E-01	1.08	7.19E-01	0.29	3.72E-05
MSMEG_6362	quinone oxidoreductase		0.61	3.11E-03	0.91	3.97E-01	1.01	2.37E-01
MSMEG_6363	cysteine desulfurase family protein		0.84	7.21E-02	0.87	1.82E-01	1.80	4.89E-01
MSMEG_6364	conserved hypothetical protein; this gene contains a frame shift which is not the result of sequencing error		0.57	9.82E-03	1.04	6.22E-01	1.31	1.81E-01
MSMEG_6365	conserved hypothetical protein		0.70	5.37E-04	1.01	8.58E-01	1.19	3.11E-01
MSMEG_6366	O-antigen export system, ATP-binding protein		0.61	7.09E-04	0.91	9.55E-02	1.46	4.95E-03
MSMEG_6367	glycosyl transferase, group 2 family protein		0.38	7.89E-06	0.93	4.83E-01	2.17	3.80E-02
MSMEG_6368	DNA-binding protein		1.86	3.12E-03	0.91	9.64E-02	0.56	3.09E-02
MSMEG_6368	DNA-binding protein		1.19	2.06E-01	1.03	7.16E-01	0.92	1.68E-01
MSMEG_6369	O-antigen export system, permease protein		0.34	3.78E-04	1.27	1.32E-01	2.29	3.10E-01
MSMEG_6370	4-carboxymuconolactone decarboxylase	*pcaC*	1.03	8.38E-01	1.06	5.88E-01	0.98	8.49E-01
MSMEG_6371	GntR-family protein regulator		0.71	4.46E-03	1.23	1.15E-01	1.04	4.28E-01
MSMEG_6372	membrane protein		0.63	7.13E-03	0.87	4.87E-02	2.00	1.93E-01
MSMEG_6373	conserved hypothetical protein		0.69	1.32E-02	1.06	5.45E-01	0.80	2.46E-01
MSMEG_6374	2-dehydropantoate 2-reductase	*panE*	0.96	8.41E-01	1.00	9.73E-01	1.46	3.26E-01
MSMEG_6375	transporter, major facilitator family protein		1.41	1.94E-01	1.07	5.64E-01	0.98	7.49E-01
MSMEG_6376	transcriptional regulator LacI family protein		1.32	3.83E-02	1.22	2.59E-01	0.94	1.11E-01
MSMEG_6377	hypothetical protein		1.45	5.62E-02	1.13	1.54E-01	1.26	3.66E-01
MSMEG_6378	senescence marker protein-30		1.70	2.34E-02	0.93	4.35E-01	0.90	6.36E-02
MSMEG_6379	transporter, major facilitator family protein		1.41	6.18E-03	0.87	3.28E-02	1.26	1.53E-01
MSMEG_6380	hypothetical protein		1.09	3.45E-01	1.10	3.27E-01	1.14	3.13E-01

MSMEG_6381	conserved hypothetical protein		0.94	5.85E-01	1.09	4.51E-01	1.18	2.03E-01
MSMEG_6382	oxidoreductase, FAD-binding		0.59	7.58E-04	1.06	5.72E-01	0.70	1.72E-02
MSMEG_6383	transcription regulator FurA		0.41	9.27E-04	2.43	4.50E-02	0.99	9.91E-01
MSMEG_6384	catalase/peroxidase HPI	*katG*	0.40	2.30E-04	2.46	3.36E-02	0.08	5.58E-07
MSMEG_6385	hypothetical oxidoreductase in MprA 5'region		0.36	2.93E-06	0.88	3.66E-02	1.78	2.47E-01
MSMEG_6386	membrane protein		0.29	1.25E-05	1.10	4.61E-01	1.29	1.62E-01
MSMEG_6387	probable arabinosyltransferase A		0.45	2.61E-05	1.09	5.07E-01	1.60	6.57E-02
MSMEG_6388	probable arabinosyltransferase B		0.48	9.09E-03	1.54	3.71E-01	1.10	6.87E-01
MSMEG_6389	probable arabinosyltransferase A		0.34	4.56E-04	1.50	4.07E-01	1.09	6.28E-01
MSMEG_6390	transporter, major facilitator family protein		0.95	2.15E-01	1.09	7.01E-01	1.31	2.17E-01
MSMEG_6391	propionyl-CoA carboxylase beta chain		0.30	3.94E-06	0.76	1.77E-02	3.81	1.25E-02
MSMEG_6392	polyketide synthase		0.33	4.56E-05	0.76	1.57E-03	3.25	8.81E-03
MSMEG_6393	acyl-CoA synthase		0.55	6.05E-05	0.84	6.97E-02	2.51	1.95E-02
MSMEG_6394	conserved hypothetical protein		0.71	8.09E-04	0.87	1.01E-01	1.72	1.95E-03
MSMEG_6395	conserved hypothetical protein		0.89	1.24E-01	0.91	2.76E-01	2.24	5.47E-03
MSMEG_6396	antigen 85-C		0.80	4.98E-03	0.85	8.56E-03	0.34	1.66E-05
MSMEG_6397	hypothetical protein		1.05	4.96E-01	0.89	6.96E-02	0.73	2.89E-03
MSMEG_6398	antigen 85-A		1.60	3.41E-02	0.64	2.29E-02	1.10	4.46E-01
MSMEG_6399	antigen 85-C		1.22	2.51E-01	0.66	9.71E-03	1.40	7.61E-02
MSMEG_6400	probable conserved transmembrane protein		0.56	1.27E-03	1.00	9.96E-01	1.62	2.25E-02
MSMEG_6401	prenyltransferase, UbiA family protein		0.36	1.24E-05	1.04	7.07E-01	2.82	4.55E-02
MSMEG_6402	PAP2 superfamily protein		0.54	2.63E-03	0.98	8.08E-01	1.77	2.71E-02
MSMEG_6403	bifunctional udp-galactofuranosyl transferase glft		0.49	1.46E-03	0.97	4.10E-01	1.33	1.26E-01
MSMEG_6404	UDP-galactopyranose mutase	*glf*	0.51	4.33E-04	1.29	7.97E-02	1.20	2.36E-01
MSMEG_6405	Erp protein		2.02	1.97E-02	0.72	9.05E-02	1.34	3.12E-01
MSMEG_6406	N-acetylmuramoyl-L-alanine amidase		1.10	7.65E-01	1.15	1.31E-01	1.29	3.86E-01
MSMEG_6407	Cof-like hydrolase		0.77	1.54E-02	0.92	1.31E-01	1.50	4.24E-02
MSMEG_6408	acyltransferase family protein		0.55	1.10E-04	1.21	2.44E-01	1.48	8.63E-03
MSMEG_6409	acyltransferase family protein		0.78	4.19E-03	0.93	2.98E-01	1.02	7.88E-01
MSMEG_6410	Rieske 2Fe-2S family protein		0.74	4.28E-02	1.01	9.49E-01	1.08	6.16E-01
MSMEG_6411	conserved hypothetical protein		0.42	3.77E-06	1.05	4.47E-01	1.20	1.14E-02
MSMEG_6412	conserved hypothetical protein		0.45	2.81E-05	0.92	1.76E-01	0.91	3.49E-01
MSMEG_6413	seryl-tRNA synthetase	*serS*	0.43	4.40E-05	0.99	9.42E-01	1.03	8.32E-01
MSMEG_6414	conserved hypothetical protein		0.89	8.89E-02	0.87	2.86E-01	0.88	3.05E-01
MSMEG_6415	conserved hypothetical protein		0.46	4.25E-05	1.01	8.71E-01	0.96	6.49E-01
MSMEG_6416	phosphoglycerate mutase family protein		1.25	7.91E-02	0.90	2.71E-01	1.06	5.08E-01
MSMEG_6417	conserved hypothetical protein		1.84	2.88E-02	0.83	9.75E-02	1.17	2.53E-01
MSMEG_6418	prephenate dehydratase		1.39	1.14E-02	1.03	7.42E-01	0.90	2.53E-01

MSMEG_6419	conserved hypothetical protein		0.66	2.92E-02	0.97	8.04E-01	1.16	4.28E-01
MSMEG_6420	abortive infection protein		0.62	2.10E-02	0.93	2.01E-01	1.35	3.97E-01
MSMEG_6421	transcriptional regulator		0.78	7.63E-02	0.88	1.56E-01	1.12	3.95E-01
MSMEG_6422	ferritin family protein		0.95	6.19E-01	1.06	7.48E-01	14.24	1.53E-01
MSMEG_6423	Glycerophosphoryl diester phosphodiesterase family protein		1.17	2.91E-02	0.77	1.77E-02	0.68	1.59E-02
MSMEG_6424	probable conserved transmembrane protein		1.20	5.25E-01	0.92	1.91E-01	0.99	9.18E-01
MSMEG_6425	rhodanese-like domain protein		1.14	2.59E-04	0.88	1.06E-01	1.07	6.38E-01
MSMEG_6426	conserved hypothetical protein		3.64	6.62E-04	0.97	8.00E-01	0.05	6.62E-08
MSMEG_6427	[Mn] superoxide dismutase		0.94	4.49E-01	1.17	1.15E-01	0.71	1.99E-01
MSMEG_6428	conserved hypothetical protein		2.96	4.72E-03	0.77	8.13E-02	0.93	6.65E-01
MSMEG_6429	hypothetical protein		1.09	4.40E-01	2.19	1.08E-01	1.55	2.22E-01
MSMEG_6430	hypothetical protein		0.92	7.20E-01	1.17	1.11E-01	0.98	4.75E-01
MSMEG_6431	conserved hypothetical protein		1.92	1.23E-03	0.53	2.73E-04	0.49	3.33E-04
MSMEG_6432	conserved hypothetical protein		1.00	9.80E-01	0.91	1.40E-01	1.18	2.47E-01
MSMEG_6433	hypothetical protein		1.32	2.96E-02	1.03	7.28E-01	2.17	1.86E-01
MSMEG_6434	conserved hypothetical protein		0.67	8.19E-03	0.82	2.03E-01	3.60	1.47E-01
MSMEG_6435	hypothetical protein		0.63	8.82E-04	0.92	2.27E-01	1.04	7.95E-01
MSMEG_6436	copper resistance protein CopC		0.59	1.65E-02	1.04	6.89E-01	0.98	6.95E-01
MSMEG_6437	copper resistance protein D		0.74	8.60E-02	0.96	4.58E-01	1.16	2.15E-01
MSMEG_6438	hypothetical protein		0.94	1.10E-01	0.77	2.28E-02	0.90	6.45E-01
MSMEG_6439	regulator of ribonuclease activity A		0.46	1.65E-05	0.84	1.54E-01	1.28	5.98E-02
MSMEG_6440	monooxygenase, flavin-binding family protein		1.83	1.97E-02	0.52	6.45E-03	4.05	3.23E-02
MSMEG_6441	transcriptional regulator, TetR family protein		1.75	5.53E-03	0.73	1.84E-03	5.54	5.20E-02
MSMEG_6442	integral membrane protein		1.21	1.33E-01	1.09	2.87E-01	1.00	9.78E-01
MSMEG_6443	DNA polymerase IV	*dinP*	2.35	2.06E-01	1.00	9.40E-01	1.41	3.67E-01
MSMEG_6444	YiaAB two helix domain protein		1.05	6.12E-01	1.17	7.65E-02	1.69	3.80E-01
MSMEG_6445	PHP domain protein		1.87	3.40E-02	0.99	9.28E-01	1.04	2.63E-01
MSMEG_6446	hypothetical protein		0.81	1.13E-01	1.10	1.55E-01	0.88	7.86E-01
MSMEG_6447	hypothetical protein		0.93	3.45E-01	1.26	1.38E-01	0.53	7.69E-02
MSMEG_6448	hypothetical protein		0.65	4.99E-02	1.19	4.99E-01	0.86	3.20E-01
MSMEG_6449	conserved hypothetical protein		0.49	9.03E-04	1.51	1.52E-02	0.65	6.42E-02
MSMEG_6450	hypothetical protein		0.44	3.76E-03	1.07	7.28E-01	0.94	2.81E-01
MSMEG_6451	transcriptional regulator, ArsR family protein		0.57	9.40E-03	1.03	8.35E-01	1.60	1.64E-01
MSMEG_6452	[NADP+] succinate-semialdehyde dehydrogenase		1.10	1.11E-01	1.05	5.51E-01	0.38	7.42E-06
MSMEG_6453	sulfate permease		1.12	5.06E-01	0.63	5.42E-02	1.21	4.03E-01
MSMEG_6454	conserved hypothetical protein		1.14	1.66E-01	0.88	3.76E-01	0.32	2.01E-04
MSMEG_6455	conserved hypothetical protein		0.90	4.32E-01	1.47	1.72E-01	1.23	2.68E-01

MSMEG_6456	conserved hypothetical protein		1.22	3.40E-02	1.40	2.27E-01	2.67	1.04E-01
MSMEG_6457	oxidoreductase molybdopterin binding domain, putative		1.36	3.20E-04	1.12	3.96E-02	0.74	1.33E-02
MSMEG_6458	glutamate synthase, small subunit		0.36	2.16E-04	0.83	1.41E-01	21.40	3.74E-01
MSMEG_6459	ferredoxin-dependent glutamate synthase 1		0.37	9.06E-05	0.80	9.01E-02	0.84	1.78E-01
MSMEG_6460	hypothetical protein		0.70	1.15E-02	1.13	1.01E-01	0.79	3.99E-01
MSMEG_6461	hypothetical protein		1.06	5.63E-01	1.04	5.67E-01	1.18	2.25E-01
MSMEG_6464	conserved hypothetical protein		0.67	1.43E-01	1.76	3.44E-01	1.26	1.45E-01
MSMEG_6465	chloride channel		1.25	2.24E-01	1.01	8.09E-01	1.16	1.76E-01
MSMEG_6466	aquaporin Z		1.62	9.61E-02	0.90	8.05E-03	0.80	1.19E-01
MSMEG_6467	starvation-induced DNA protecting protein		1.86	3.52E-01	3.13	1.14E-02	0.23	1.01E-05
MSMEG_6468	conserved hypothetical protein		0.99	9.49E-01	1.68	9.46E-02	1.20	4.28E-01
MSMEG_6469	conserved hypothetical protein		11.53	9.13E-04	0.79	1.04E-01	0.61	8.60E-02
MSMEG_6470	hypothetical protein		1.12	3.33E-01	2.01	1.21E-01	1.11	5.04E-01
MSMEG_6471	glycine/D-amino acid oxidase		0.73	2.56E-01	0.83	1.73E-03	0.76	5.83E-02
MSMEG_6472	hypothetical protein		7.59	4.97E-02	4.08	3.47E-02	1.14	4.92E-02
MSMEG_6473	tetracenomycin polyketide synthesis O-methyltransferase TcmP		0.98	8.48E-01	1.41	3.17E-02	1.10	6.72E-01
MSMEG_6474	nucleoside-diphosphate-sugar epimerase		0.91	6.61E-01	1.01	9.07E-01	3.11	3.63E-01
MSMEG_6475	conserved hypothetical protein		0.98	8.50E-01	1.05	1.97E-01	0.64	3.71E-02
MSMEG_6476	conserved hypothetical protein		1.01	9.04E-01	0.88	1.51E-01	1.07	6.97E-01
MSMEG_6477	methionine-S-sulfoxide reductase	*msrA*	1.97	3.12E-03	0.74	1.77E-02	0.90	1.35E-01
MSMEG_6478	putative cytochrome P450 135B1		1.25	3.42E-01	0.94	6.27E-01	7.02	5.33E-02
MSMEG_6479	putative transcriptional regulator		1.53	2.51E-01	0.86	2.40E-01	0.98	8.89E-01
MSMEG_6480	putative transcriptional regulatory protein		1.01	8.35E-01	0.98	6.46E-01	1.05	3.46E-01
MSMEG_6481	conserved hypothetical protein		0.42	4.06E-05	0.94	2.58E-01	1.09	6.46E-01
MSMEG_6482	secreted protein		0.81	4.39E-03	1.11	2.65E-01	0.90	3.06E-01
MSMEG_6483	methyltransferase type 11		0.51	3.98E-05	0.91	1.03E-01	0.70	4.61E-02
MSMEG_6484	glycosyl transferase		0.72	5.88E-03	1.03	5.68E-01	1.32	1.91E-01
MSMEG_6485	ATP binding protein		0.58	3.20E-04	1.07	1.31E-02	1.64	8.35E-03
MSMEG_6486	oxidoreductase, FAD/FMN-binding		0.59	3.75E-03	0.95	5.58E-01	0.98	9.42E-01
MSMEG_6487	2-hydroxy-3-carboxy-6-oxo-7-methylocta-2, 4-dienoate decarboxylase		1.00	9.95E-01	1.02	8.79E-01	1.02	8.75E-01
MSMEG_6488	conserved hypothetical protein		0.60	7.94E-03	0.96	7.00E-01	0.87	3.00E-01
MSMEG_6489	2,6-dihydroxypyridine hydroxylase		1.47	1.59E-01	1.16	5.54E-01	1.01	8.60E-01
MSMEG_6490	major facilitator superfamily protein		1.68	1.93E-02	1.19	1.16E-01	0.98	3.27E-01
MSMEG_6491	aldehyde dehydrogenase		1.03	8.30E-01	1.34	2.81E-02	1.04	4.00E-01
MSMEG_6492	GAF domain protein, putative		0.92	4.67E-01	1.40	2.29E-01	0.80	1.50E-01

MSMEG_6493	hypothetical protein		0.97	5.46E-01	1.07	6.12E-01	1.12	2.47E-01
MSMEG_6494	ABC nitrate/sulfonate/bicarbonate family protein transporter, inner membrane subunit, putative		1.44	2.80E-01	1.12	3.70E-01	0.99	9.32E-01
MSMEG_6495	ABC nitrate/sulfonate/bicarbonate family protein transporter, inner membrane subunit, putative		1.39	9.59E-02	0.95	6.92E-01	1.22	3.76E-01
MSMEG_6496	nitrate/sulfonate/bicarbonate ABC transporter ATPase subunit		1.48	1.04E-01	0.97	8.14E-01	1.07	5.69E-01
MSMEG_6497	hypothetical protein		0.74	9.76E-03	0.91	5.45E-01	1.47	3.15E-01
MSMEG_6498	hypothetical protein		2.07	1.54E-02	2.23	8.69E-02	2.27	2.13E-01
MSMEG_6499	conserved hypothetical protein		2.90	3.09E-03	1.06	1.86E-01	0.36	1.08E-05
MSMEG_6500	conserved hypothetical protein		1.22	2.48E-01	1.39	2.55E-01	0.46	1.62E-03
MSMEG_6501	hypothetical protein		1.13	2.06E-01	1.36	1.12E-01	0.82	2.98E-01
MSMEG_6502	conserved hypothetical protein		0.34	4.12E-04	1.02	8.89E-01	1.92	6.10E-02
MSMEG_6503	transcriptional regulator, TetR family protein		1.47	4.61E-01	1.32	5.18E-02	0.87	1.53E-01
MSMEG_6504	conserved hypothetical protein		0.31	1.40E-05	1.19	2.51E-01	1.05	1.75E-02
MSMEG_6505	NfnB protein		0.96	5.23E-01	1.11	2.29E-01	1.01	7.14E-01
MSMEG_6506	nicotinamidase/pyrazinamidase		1.29	8.55E-04	0.82	3.55E-02	0.87	5.13E-01
MSMEG_6507	glycogen debranching enzyme GlgX	*glgX*	0.79	2.42E-02	0.97	6.95E-01	1.21	4.67E-02
MSMEG_6508	MarR-family protein transcriptional regulator		0.85	1.83E-01	0.79	8.34E-02	3.32	1.02E-01
MSMEG_6509	ABC-type drug export system, ATP-binding protein		0.92	1.64E-01	1.01	8.94E-01	3.71	1.30E-02
MSMEG_6510	ABC-type drug export system, membrane protein		0.91	6.12E-01	0.92	3.67E-01	2.93	2.73E-02
MSMEG_6511	acyl-CoA dehydrogenase domain protein		1.02	8.38E-01	0.96	6.85E-01	4.04	2.18E-03
MSMEG_6512	acyl-CoA dehydrogenase domain protein		1.05	5.40E-01	1.02	8.99E-01	4.27	1.66E-02
MSMEG_6513	membrane transport protein		0.72	2.98E-01	1.29	4.85E-02	1.18	4.87E-02
MSMEG_6514	trehalose synthase-fused probable maltokinase		0.95	4.50E-01	0.94	5.34E-01	0.88	2.56E-01
MSMEG_6515	trehalose synthase	*treS*	0.76	1.59E-02	1.24	1.30E-01	0.79	4.73E-02
MSMEG_6516	hypothetical protein		0.90	4.82E-01	0.59	8.76E-04	0.99	6.38E-01
MSMEG_6517	hypothetical protein		1.16	1.72E-01	1.04	7.53E-01	1.08	2.19E-01
MSMEG_6518	conserved hypothetical protein		1.22	6.64E-02	0.85	2.54E-01	0.57	2.36E-02
MSMEG_6519	pyridoxamine 5'-phosphate oxidase family		0.58	4.91E-04	1.07	7.08E-01	1.04	6.50E-01
MSMEG_6520	orotate phosphoribosyltransferase		0.96	5.37E-01	0.70	1.99E-02	0.95	5.55E-01
MSMEG_6521	sulfate/thiosulfate import ATP-binding protein CysA 2		0.80	5.89E-02	1.02	7.72E-01	0.55	2.18E-02
MSMEG_6522	ABC transporter, inner membrane subunit		0.54	1.09E-06	1.17	9.05E-02	0.70	7.47E-02
MSMEG_6523	ABC transporter, membrane spanning protein		0.64	2.65E-02	1.05	6.01E-01	0.59	2.35E-02
MSMEG_6524	ABC Polyamine/Opine/Phosphonate transporter, periplasmic ligand binding protein		0.85	5.95E-02	1.14	1.79E-01	0.22	1.83E-05

MSMEG_6525	hypothetical protein	1.08	4.84E-01	0.87	1.09E-01	0.71	1.78E-02
MSMEG_6526	pyridoxamine 5'-phosphate oxidase family	1.53	1.22E-01	0.87	2.67E-01	1.07	3.60E-01
MSMEG_6527	transcriptional regulator, TetR family protein	1.18	1.91E-01	1.14	3.09E-01	1.05	4.23E-01
MSMEG_6528	conserved hypothetical protein	1.09	5.88E-01	0.99	9.57E-01	0.95	4.32E-01
MSMEG_6529	conserved hypothetical protein	1.45	1.36E-03	0.98	7.58E-01	0.91	1.03E-01
MSMEG_6530	cytochrome c oxidase subunit III family protein	2.31	3.56E-01	1.03	7.04E-01	0.96	5.97E-01
MSMEG_6531	conserved hypothetical protein	0.68	1.97E-02	1.63	1.37E-02	1.95	2.07E-03
MSMEG_6532	transcriptional regulatory protein	2.06	6.94E-03	0.81	5.35E-02	1.23	1.54E-01
MSMEG_6533	conserved hypothetical protein	1.19	4.13E-02	1.03	2.12E-01	1.16	3.18E-01
MSMEG_6534	hypothetical protein	1.19	2.96E-01	1.22	1.98E-01	3.24	3.76E-01
MSMEG_6535	translation elongation factor EF-G	25.45	2.31E-05	1.26	3.01E-01	0.08	2.31E-07
MSMEG_6536	carboxymuconolactone decarboxylase	0.75	1.39E-01	1.15	1.52E-01	1.03	8.53E-01
MSMEG_6537	transcriptional regulator, TetR family protein	0.81	4.95E-02	1.17	1.03E-01	1.14	3.69E-01
MSMEG_6538	3-oxoacyl-(acyl-carrier-protein) reductase,	0.49	6.53E-04	0.95	4.71E-01	0.98	6.65E-01
MSMEG_6539	conserved hypothetical protein	0.68	2.16E-02	0.95	5.84E-01	1.44	1.40E-01
MSMEG_6540	virulence factor Mce family protein	0.51	6.99E-03	0.89	3.30E-01	3.82	1.31E-01
MSMEG_6541	anti-sigma factor antagonist	0.82	2.23E-02	1.40	1.03E-02	0.76	8.59E-02
MSMEG_6542	B12 binding domain protein	0.90	1.51E-01	1.21	6.30E-02	1.24	4.42E-01
MSMEG_6543	GAF domain protein; this gene contains a frame shift which is not the result of sequencing error; identified by match to protein family HMM PF01590	0.67	5.21E-03	1.13	3.68E-02	3.35	8.57E-02
MSMEG_6543	GAF domain protein; this gene contains a frame shift which is not the result of sequencing error; identified by match to protein family HMM PF01590	0.92	1.20E-01	1.01	8.86E-01	6.51	1.97E-01
MSMEG_6544	transport-associated, putative	0.95	4.24E-01	0.74	8.42E-03	1.63	6.33E-02
MSMEG_6545	phosphoglycerate mutase family protein	1.11	5.25E-01	1.39	3.53E-02	1.29	1.49E-01
MSMEG_6546	transcriptional regulatory protein	0.86	1.49E-01	1.11	4.00E-01	1.13	8.21E-02
MSMEG_6547	amidohydrolase 2	1.82	3.10E-02	0.91	5.59E-01	1.07	6.10E-01
MSMEG_6548	Rieske iron-sulfur protein	1.39	7.64E-02	1.01	9.15E-01	0.96	3.37E-01
MSMEG_6549	conserved hypothetical protein	1.52	1.59E-01	1.37	1.16E-01	0.99	8.05E-01
MSMEG_6550	hypothetical protein	1.34	1.49E-01	1.46	9.03E-02	0.75	5.65E-02
MSMEG_6550	hypothetical protein	3.88	1.97E-01	1.24	2.40E-01	1.01	7.39E-01
MSMEG_6551	hypothetical protein	1.68	1.56E-01	1.14	2.05E-01	1.18	6.61E-01
MSMEG_6552	conserved hypothetical protein	1.19	2.08E-01	1.14	2.01E-01	1.16	3.29E-01
MSMEG_6553	ABC transporter ATP-binding protein	0.56	2.61E-03	1.25	1.90E-01	0.64	1.05E-01

MSMEG_6554	hypothetical ABC transporter ATP-binding protein	0.70	1.24E-01	1.53	2.24E-02	1.01	3.80E-01
MSMEG_6555	transcriptional regulator, TetR family protein, putative	0.68	1.33E-01	1.52	9.82E-02	0.80	2.96E-01
MSMEG_6556	putative transcriptional regulator	1.04	7.68E-01	1.01	9.45E-01	1.47	1.38E-01
MSMEG_6557	dehydrogenase	0.28	1.88E-05	1.40	4.97E-02	7.35	8.62E-02
MSMEG_6558	putative enoyl-CoA hydratase	1.32	3.25E-02	1.00	9.65E-01	1.36	2.04E-01
MSMEG_6559	hypothetical protein	2.01	5.40E-04	0.73	3.39E-02	2.81	2.40E-02
MSMEG_6560	acyl dehydratase	0.83	9.39E-02	1.15	2.21E-01	1.16	7.05E-01
MSMEG_6561	nonspecific lipid-transfer protein	0.89	5.11E-01	1.46	1.20E-01	1.00	9.72E-01
MSMEG_6562	putative acyl-CoA dehydrogenase	0.98	9.31E-01	1.81	1.56E-01	0.96	3.06E-01
MSMEG_6563	dihydrokaempferol 4-reductase	0.93	2.88E-01	2.49	1.29E-01	1.30	1.68E-01
MSMEG_6564	transcriptional regulator, TetR family protein	0.70	1.03E-02	1.00	9.74E-01	1.17	1.88E-01
MSMEG_6565	conserved hypothetical protein	0.68	2.76E-03	1.99	1.01E-01	0.90	1.17E-01
MSMEG_6566	hypothetical protein	2.49	3.81E-02	4.41	4.19E-02	4.63	1.64E-01
MSMEG_6567	iron-dependent peroxidase	0.88	4.13E-01	2.23	1.81E-01	1.16	3.02E-01
MSMEG_6568	regulatory protein, LuxR, putative	1.00	9.75E-01	1.14	1.17E-01	1.03	4.34E-01
MSMEG_6569	conserved hypothetical protein	1.23	1.91E-01	1.46	4.94E-02	1.01	8.69E-01
MSMEG_6570	integral membrane protein	1.31	4.88E-01	1.63	2.10E-01	1.33	1.28E-01
MSMEG_6571	conserved hypothetical protein	0.97	8.94E-01	1.17	4.74E-01	1.62	2.46E-01
MSMEG_6572	methyltransferase	0.93	3.02E-01	1.84	3.07E-01	1.00	7.09E-01
MSMEG_6573	conserved hypothetical protein	2.35	1.41E-01	1.08	6.74E-01	1.09	3.60E-01
MSMEG_6574	conserved hypothetical protein	0.74	1.61E-02	1.07	4.59E-01	1.65	3.99E-02
MSMEG_6575	beta-lactamase	0.74	1.08E-02	0.98	6.75E-01	1.48	1.34E-01
MSMEG_6576	pyridoxamine 5'-phosphate oxidase family	3.85	2.22E-02	1.66	1.25E-01	0.79	2.37E-01
MSMEG_6577	methylmalonyl-CoA carboxyltransferase 12S subunit	1.01	9.32E-01	0.99	7.10E-01	2.02	1.53E-01
MSMEG_6578	hypothetical protein	1.07	2.63E-01	1.29	3.46E-01	0.99	6.00E-01
MSMEG_6579	conserved hypothetical protein	2.45	6.44E-02	1.25	4.58E-02	0.38	2.24E-02
MSMEG_6580	transcriptional regulator family protein	1.29	5.39E-02	0.83	4.28E-03	5.81	3.56E-01
MSMEG_6581	oxidoreductase, short chain dehydrogenase/reductase family protein	0.90	1.33E-01	1.17	1.95E-01	0.73	3.74E-03
MSMEG_6582	pyridine nucleotide-disulphide oxidoreductase domain protein	1.20	4.28E-01	0.97	6.11E-01	1.01	8.44E-01
MSMEG_6583	antigen 85-C	1.06	8.63E-01	0.82	3.74E-01	1.12	7.59E-01
MSMEG_6584	acyl-CoA dehydrogenase	1.28	7.80E-02	0.85	2.06E-02	0.49	9.28E-03
MSMEG_6585	acyl-CoA dehydrogenase	1.34	1.57E-02	0.88	3.23E-02	0.24	2.96E-04
MSMEG_6586	alpha/beta hydrolase, putative	1.02	5.08E-01	1.22	1.33E-01	1.05	5.40E-01

MSMEG_6587	ATP-dependent helicase HrpA	*hrpA*	1.56	5.37E-01	1.02	9.02E-01	1.54	3.10E-01
MSMEG_6588	fumarylacetoacetate		0.71	1.06E-01	0.96	7.65E-01	0.96	8.51E-02
MSMEG_6590	conserved hypothetical protein		1.29	5.28E-02	0.97	9.20E-02	1.00	9.70E-01
MSMEG_6591	aminotransferase, class V family protein		0.47	2.39E-05	1.02	7.39E-01	0.98	9.25E-01
MSMEG_6592	short-chain dehydrogenase/reductase SDR		0.72	2.06E-02	0.91	2.05E-01	1.91	2.16E-02
MSMEG_6593	ATP-binding protein		0.82	4.21E-02	0.87	2.33E-01	0.68	2.50E-02
MSMEG_6594	amino acid ABC transporter, permease protein, 3-TM region, His/Glu/Gln/Arg/opine		0.83	1.69E-02	0.72	6.14E-02	0.52	1.66E-02
MSMEG_6595	secreted protein		1.08	5.61E-01	0.80	1.09E-02	0.40	1.14E-03
MSMEG_6596	conserved hypothetical protein		0.39	4.80E-05	0.77	1.47E-02	0.67	5.78E-02
MSMEG_6597	hydrolase of the alpha/beta superfamily protein		0.75	1.20E-02	0.98	7.85E-01	1.40	4.78E-01
MSMEG_6598	6,7-dimethyl-8-ribityllumazine synthase		0.52	4.26E-04	1.12	3.86E-01	1.08	3.58E-01
MSMEG_6599	regulatory protein, LuxR; this gene contains a frame shift which is not the result of sequencing error		2.72	1.09E-01	1.16	6.23E-02	1.26	2.88E-01
MSMEG_6599	regulatory protein, LuxR; this gene contains a frame shift which is not the result of sequencing error		1.24	1.94E-01	0.98	8.41E-01	1.01	8.22E-01
MSMEG_6600	transposase		1.56	2.73E-01	0.94	6.25E-01	1.27	1.29E-01
MSMEG_6601	hypothetical protein		0.87	7.19E-02	0.87	1.15E-01	1.25	1.07E-01
MSMEG_6602	oxidoreductase		0.93	3.83E-01	1.00	9.85E-01	2.44	2.42E-01
MSMEG_6603	NADH:flavin oxidoreductase/nadh oxidase		0.80	1.02E-01	0.82	4.22E-01	1.05	4.70E-01
MSMEG_6604	transcriptional regulator, TetR family protein		0.85	5.15E-02	0.94	1.35E-01	1.10	7.08E-01
MSMEG_6605	transcriptional regulatory protein		0.90	4.22E-01	0.86	1.37E-01	1.73	6.56E-02
MSMEG_6606	hypothetical protein		1.14	2.56E-01	1.11	3.95E-01	1.03	6.27E-01
MSMEG_6607	hypothetical protein		0.92	4.83E-01	1.36	1.07E-02	0.95	4.42E-01
MSMEG_6608	hypothetical protein		0.91	2.67E-02	1.39	2.13E-01	1.06	3.72E-01
MSMEG_6609	hypothetical protein		0.77	1.34E-01	1.26	1.66E-01	1.06	6.39E-01
MSMEG_6610	protein of unknown function DUF58, putative		1.82	4.51E-01	1.40	4.00E-02	1.45	5.42E-01
MSMEG_6611	hypothetical protein		1.84	1.33E-01	0.98	8.61E-01	0.90	2.00E-01
MSMEG_6612	ATPase, MoxR family protein	*moxR*	1.11	5.07E-01	1.45	6.87E-02	0.73	1.40E-01
MSMEG_6613	conserved hypothetical protein		1.58	3.10E-02	1.13	4.25E-01	1.55	2.51E-01
MSMEG_6614	integral membrane protein		1.08	3.82E-01	1.00	9.99E-01	1.18	3.42E-01
MSMEG_6615	hypothetical protein		3.04	1.36E-01	1.97	1.41E-01	0.51	7.20E-02
MSMEG_6616	S-(hydroxymethyl)glutathione dehydrogenase		1.99	2.27E-01	2.51	9.03E-02	0.42	1.26E-02
MSMEG_6617	nudix hydrolase		1.09	7.02E-01	1.10	3.63E-01	1.42	1.53E-01

MSMEG_6618	DEAD/DEAH box helicase; this gene contains a frame shift which is not the result of sequencing error; identified by match to protein family HMM PF00270; match to protein family HMM PF00271; match to protein family HMM PF04851		0.65	3.03E-04	0.98	7.46E-01	1.81	1.56E-01
MSMEG_6619	conserved hypothetical protein; this gene contains a frame shift which is not the result of sequencing error		0.47	1.36E-03	0.98	8.71E-01	2.12	1.85E-01
MSMEG_6620	conserved hypothetical protein		0.69	1.19E-02	0.84	1.48E-01	2.14	5.95E-03
MSMEG_6621	bacterial luciferase family protein		1.07	5.16E-01	1.18	4.43E-01	1.00	9.82E-01
MSMEG_6622	cytochrome P450 monooxygenase		1.47	1.20E-01	1.17	2.51E-01	1.08	5.96E-01
MSMEG_6623	dehydrogenase		1.13	5.35E-01	0.98	8.82E-01	1.15	3.99E-01
MSMEG_6624	conserved hypothetical protein		1.44	3.14E-03	0.97	9.23E-01	0.96	3.36E-01
MSMEG_6625	glyoxalase family protein		1.24	2.47E-01	1.05	6.36E-01	1.07	4.91E-01
MSMEG_6626	short-chain dehydrogenase/reductase SDR		1.13	3.36E-01	0.87	1.13E-01	1.60	8.04E-02
MSMEG_6627	nitroreductase family protein		2.24	8.43E-02	0.72	1.44E-01	0.86	1.69E-01
MSMEG_6628	transcriptional regulator, TetR family protein		1.27	2.81E-01	0.77	2.83E-01	0.80	2.56E-01
MSMEG_6629	malonate decarboxylase, epsilon subunit	*mdcH*	0.76	1.44E-01	1.07	4.58E-01	1.16	2.58E-01
MSMEG_6630	glyoxalase family protein		1.19	2.76E-01	0.90	4.86E-01	0.87	5.51E-01
MSMEG_6631	phosphoribosyl-dephospho-CoA transferase	*mdcG*	0.87	3.04E-01	1.55	2.17E-01	1.28	2.11E-01
MSMEG_6632	beta subunit of malonate decarboxylase		0.60	4.57E-03	1.38	1.66E-01	1.12	2.69E-01
MSMEG_6633	malonate decarboxylase acyl carrier protein	*mdcC*	0.83	6.58E-01	1.04	6.19E-01	2.38	3.68E-01
MSMEG_6634	triphosphoribosyl-dephospho-CoA synthase MdcB	*mdcB*	0.78	5.91E-02	1.09	4.13E-01	0.88	1.59E-01
MSMEG_6635	alpha subunit of malonate decarboxylase		0.40	1.19E-04	1.03	7.31E-01	1.18	4.15E-01
MSMEG_6636	[Mn] superoxide dismutase		0.70	3.37E-02	0.79	1.57E-01	0.38	2.69E-05
MSMEG_6637	hypothetical protein		1.66	5.23E-02	0.57	1.75E-02	1.10	7.13E-01
MSMEG_6638	5-methyltetrahydropteroyltriglutamate--homocysteine S-methyltransferase	*metE*	0.41	4.83E-05	1.61	3.78E-01	0.61	1.77E-01
MSMEG_6639	transcriptional regulator, GntR family protein, putative		0.64	3.24E-04	0.87	4.32E-02	0.69	3.24E-02
MSMEG_6640	hypothetical protein		0.95	5.52E-01	0.96	3.57E-01	0.85	3.33E-01
MSMEG_6641	nitrilotriacetate monooxygenase component A		0.84	9.82E-02	0.70	3.93E-02	0.94	3.94E-01
MSMEG_6642	caib/baif family protein		0.89	6.26E-02	0.97	8.08E-01	0.92	2.73E-01
MSMEG_6643	DNA-binding protein		0.98	8.43E-01	1.07	4.18E-01	1.26	2.27E-01
MSMEG_6644	malyl-CoA lyase		1.02	8.55E-01	1.11	6.93E-01	0.93	6.05E-01
MSMEG_6645	2-methylcitrate dehydratase 2		0.99	8.09E-01	1.05	6.68E-01	1.05	7.33E-01

MSMEG_6646	methylisocitrate lyase	*prpB*	0.85	3.30E-01	1.22	9.45E-02	0.95	1.84E-01
MSMEG_6647	citrate synthase 2		1.28	5.17E-01	1.19	8.56E-02	1.00	4.72E-01
MSMEG_6648	pyruvate carboxylase	*pyc*	1.10	6.65E-01	1.43	7.19E-02	1.01	8.52E-01
MSMEG_6649	conserved hypothetical protein		0.26	2.26E-05	1.18	2.82E-01	0.90	7.36E-01
MSMEG_6650	hypothetical protein		1.42	6.85E-02	0.99	9.34E-01	1.08	5.08E-01
MSMEG_6651	hypothetical protein		1.03	8.30E-01	0.94	5.96E-01	1.06	8.47E-01
MSMEG_6652	hypothetical protein		1.17	4.73E-02	1.17	5.41E-01	0.96	3.47E-01
MSMEG_6653	hypothetical protein; this gene contains a frame shift which is not the result of sequencing error; identified by Glimmer2; putative		1.70	9.77E-02	1.69	2.70E-01	1.10	3.04E-01
MSMEG_6653	hypothetical protein; this gene contains a frame shift which is not the result of sequencing error; identified by Glimmer2; putative		0.91	5.67E-01	0.87	3.43E-01	0.93	4.83E-01
MSMEG_6654	TnpC protein		1.07	6.26E-01	1.35	1.31E-01	2.12	1.68E-01
MSMEG_6655	conserved hypothetical protein		1.14	6.49E-01	1.34	1.63E-01	3.90	2.51E-01
MSMEG_6656	conserved hypothetical protein		1.44	2.82E-01	1.26	1.67E-01	1.25	4.44E-01
MSMEG_6657	alpha subunit of malonate decarboxylase		1.09	4.17E-01	1.05	3.74E-01	1.45	3.48E-01
MSMEG_6658	alpha/beta hydrolase fold		0.99	9.56E-01	1.72	5.25E-02	0.93	5.03E-01
MSMEG_6659	hypothetical protein		1.02	8.74E-01	1.02	3.01E-01	1.28	2.84E-01
MSMEG_6660	permease, cytosine/purines, uracil, thiamine, allantoin family protein		0.68	2.69E-01	0.91	4.29E-01	0.87	2.30E-01
MSMEG_6661	O-methyltransferase, putative		0.78	2.18E-03	0.94	2.24E-01	1.72	3.81E-02
MSMEG_6662	short chain dehydrogenase		1.93	3.41E-03	1.14	1.00E-01	1.17	1.85E-01
MSMEG_6663	C5-O-methyltransferase		1.65	1.31E-01	1.22	1.58E-01	1.52	1.37E-01
MSMEG_6664	methylenetetrahydrofolate reductase family protein, putative		1.43	2.94E-01	1.28	1.70E-01	1.14	2.38E-01
MSMEG_6665	integral membrane protein		1.21	4.69E-01	1.52	1.48E-01	0.67	9.20E-02
MSMEG_6666	conserved hypothetical protein		0.96	8.44E-01	1.04	3.67E-01	0.85	1.92E-01
MSMEG_6667	conserved hypothetical protein		1.01	9.51E-01	1.52	5.70E-03	1.05	4.45E-01
MSMEG_6668	ABC transporter, periplasmic substrate-binding protein, putative		1.18	6.07E-01	0.93	3.40E-01	0.91	3.83E-01
MSMEG_6669	ABC transporter, permease protein		1.22	4.05E-01	1.12	1.73E-01	1.00	6.69E-01
MSMEG_6670	ABC transporter, permease protein, putative		1.05	6.95E-01	1.51	3.03E-01	1.06	5.68E-01
MSMEG_6671	sulfate/thiosulfate import ATP-binding protein CysA		1.35	1.24E-01	1.04	5.39E-01	1.01	9.05E-01
MSMEG_6672	type I phosphodiesterase		1.05	3.49E-01	1.20	1.75E-01	0.97	5.66E-01
MSMEG_6673	6-aminohexanoate-cyclic-dimer hydrolase		0.73	8.41E-02	0.99	7.88E-01	0.97	6.51E-01
MSMEG_6674	transcriptional regulator, LysR family protein		1.57	3.49E-01	1.22	3.34E-01	0.84	1.28E-01

MSMEG_6675	hypothetical protein		1.00	9.52E-01	1.96	2.16E-01	1.02	5.55E-01
MSMEG_6676	probable conserved transmembrane protein		1.27	1.28E-01	1.07	5.84E-01	1.54	1.71E-01
MSMEG_6677	transcriptional regulator, LysR family protein		5.20	4.33E-03	1.27	2.96E-01	1.03	8.57E-01
MSMEG_6678	conserved hypothetical protein		1.18	2.44E-01	0.91	3.51E-01	0.33	4.96E-04
MSMEG_6679	metallo-beta-lactamase family protein		1.09	3.51E-01	1.00	9.76E-01	1.04	3.25E-01
MSMEG_6680	integral membrane protein		1.34	9.61E-02	0.76	1.56E-04	0.88	1.82E-01
MSMEG_6681	hypothetical protein		1.58	7.55E-02	0.84	1.59E-01	1.04	7.59E-01
MSMEG_6682	RNA polymerase sigma-70 factor, putative		1.50	2.23E-01	1.31	4.40E-01	0.98	2.84E-01
MSMEG_6683	probable peroxiredoxin, putative		1.15	1.08E-01	1.15	5.88E-01	0.98	8.58E-01
MSMEG_6684	agarase		1.05	7.86E-01	1.33	2.59E-02	0.98	1.82E-01
MSMEG_6685	4-aminobutyrate transaminase	*gabT*	0.79	7.57E-02	0.98	8.57E-01	0.39	4.99E-04
MSMEG_6686	glutaryl-CoA dehydrogenase		0.62	1.17E-02	0.95	5.84E-01	0.50	1.61E-03
MSMEG_6687	aldehyde dehydrogenase, thermostable		0.83	3.06E-02	0.93	2.55E-01	0.52	8.67E-04
MSMEG_6688	regulatory protein		1.20	2.25E-01	1.05	8.46E-01	1.67	5.01E-02
MSMEG_6689	CAIB/BAIF family protein		0.74	1.35E-01	0.95	4.91E-01	0.73	2.44E-02
MSMEG_6690	glutamine amidotransferase class-I		1.00	9.84E-01	0.91	2.05E-01	1.57	1.09E-01
MSMEG_6691	glutamine amidotransferase		1.15	4.33E-01	0.99	8.71E-01	1.08	7.00E-01
MSMEG_6692	putative glutamine-binding periplasmic protein		3.08	1.02E-02	0.79	9.50E-02	0.89	2.35E-01
MSMEG_6693	glutamine synthetase III		3.54	4.25E-03	0.71	1.83E-02	0.60	9.67E-03
MSMEG_6694	conserved domain protein		2.31	1.24E-02	0.78	1.02E-01	0.88	4.99E-01
MSMEG_6695	cytochrome P450		4.85	3.90E-02	0.67	2.95E-02	1.05	3.55E-01
MSMEG_6696	hypothetical protein		0.72	2.66E-02	0.72	2.09E-01	3.21	6.67E-02
MSMEG_6699	conserved hypothetical protein		0.74	1.45E-01	0.94	3.39E-01	0.94	4.21E-01
MSMEG_6700	regulatory protein		0.95	4.57E-01	0.89	2.13E-02	1.18	3.86E-01
MSMEG_6701	amino acid permease		3.13	8.01E-02	0.71	9.90E-03	0.96	8.87E-02
MSMEG_6702	[NADP+] succinate-semialdehyde dehydrogenase		3.33	5.94E-02	0.88	3.32E-01	1.05	6.74E-01
MSMEG_6703	N5,N10- methylenetetrahydromethanopterin reductase-related protein		1.74	6.85E-02	1.93	8.28E-02	0.99	7.82E-01
MSMEG_6704	shikimate 5-dehydrogenase		3.62	3.20E-01	1.12	2.57E-01	1.29	2.14E-01
MSMEG_6705	regulatory protein		2.10	3.12E-02	1.19	2.18E-01	1.24	4.13E-01
MSMEG_6706	hypothetical protein		1.78	4.21E-01	1.35	1.60E-01	1.17	2.69E-01
MSMEG_6707	hypothetical protein		0.95	7.67E-01	1.14	1.11E-01	1.61	1.95E-01
MSMEG_6708	epoxide hydrolase		1.23	1.60E-01	1.23	3.31E-01	1.01	8.96E-01
MSMEG_6709	cyclopentanol dehydrogenase		2.15	1.02E-01	1.34	1.42E-01	0.87	1.91E-01
MSMEG_6710	hydrolase, alpha/beta fold family protein		1.20	2.86E-02	1.33	9.46E-02	0.85	2.06E-01
MSMEG_6711	4-hydroxybenzoate transporter		1.26	1.27E-01	0.93	4.47E-01	0.92	3.91E-01
MSMEG_6712	maleylacetate reductase		1.39	1.65E-01	1.31	4.51E-02	0.94	3.44E-01
MSMEG_6713	hydroxyquinol 1,2-dioxygenase		1.01	8.13E-01	1.22	2.53E-01	1.48	3.41E-01

MSMEG_6714	conserved hypothetical protein	1.41	5.58E-02	1.16	6.18E-02	1.14	5.40E-01
MSMEG_6715	AP endonuclease, family protein 2	2.33	9.13E-02	1.32	1.96E-01	1.12	6.25E-01
MSMEG_6716	AP endonuclease, family protein 2	1.86	2.08E-01	1.07	4.98E-01	1.06	5.91E-01
MSMEG_6717	oxidoreductase, Gfo/Idh/MocA family protein	1.88	1.64E-01	1.15	2.05E-01	1.01	9.11E-01
MSMEG_6718	transporter, major facilitator family protein	2.19	2.86E-02	0.83	9.18E-02	0.76	3.36E-02
MSMEG_6719	epoxide hydrolase	1.24	2.32E-01	1.15	8.05E-02	0.99	9.36E-01
MSMEG_6720	epoxide hydrolase	1.48	1.02E-02	1.04	5.13E-01	1.03	6.56E-01
MSMEG_6721	hypothetical protein	1.49	4.50E-02	0.99	9.66E-01	1.11	3.30E-01
MSMEG_6722	conserved hypothetical protein	1.89	1.60E-02	0.73	2.13E-02	0.58	5.67E-02
MSMEG_6723	conserved hypothetical protein	0.54	1.02E-03	1.06	7.66E-01	1.21	7.89E-02
MSMEG_6724	ABC-type cobalt transport system	0.40	5.18E-05	1.03	6.57E-01	1.50	2.25E-01
MSMEG_6725	ABC transporter, ATP-binding protein	0.54	3.85E-04	0.95	7.38E-01	1.52	1.41E-01
MSMEG_6726	ABC transporter, permease protein	0.60	6.01E-04	0.70	2.85E-02	1.29	3.53E-01
MSMEG_6727	amino acid permease-associated region	1.13	6.34E-01	1.74	3.21E-02	0.72	6.86E-02
MSMEG_6728	conserved hypothetical protein	0.79	5.29E-01	1.48	8.02E-02	0.85	1.02E-01
MSMEG_6729	DNA-binding protein	1.28	1.90E-01	0.96	6.80E-01	0.89	5.63E-01
MSMEG_6730	putative oxidoreductase YdbC	0.86	5.52E-01	0.89	4.61E-01	1.22	7.69E-02
MSMEG_6731	transcriptional regulatory protein	0.96	7.27E-01	0.87	9.46E-02	0.71	1.05E-01
MSMEG_6732	integral membrane protein	1.05	9.12E-01	1.32	8.68E-02	1.10	6.15E-01
MSMEG_6733	hydrolase, carbon-nitrogen family protein	0.59	3.19E-04	0.79	3.34E-02	1.15	4.45E-01
MSMEG_6734	dibenzothiophene desulfurization enzyme A	0.21	2.70E-04	0.98	6.63E-01	0.76	1.51E-01
MSMEG_6735	amino acid permease, putative	0.30	8.05E-04	1.04	6.97E-01	0.92	7.18E-01
MSMEG_6736	LacI-family protein transcriptional regulator	0.92	1.46E-01	1.10	1.21E-01	0.94	1.88E-01
MSMEG_6737	putative membrane protein	1.19	2.60E-01	0.89	1.82E-01	1.22	2.53E-01
MSMEG_6738	probable transcriptional regulator YdhC	0.78	1.06E-01	1.18	2.49E-01	1.19	3.42E-01
MSMEG_6739	hypothetical protein	1.99	1.73E-02	0.73	2.74E-01	1.10	5.10E-01
MSMEG_6740	1-aminocyclopropane-1-carboxylate deaminase	0.80	2.46E-02	0.94	3.29E-01	0.56	1.63E-02
MSMEG_6741	antar domain protein	0.60	1.81E-03	0.95	6.84E-01	1.50	4.14E-01
MSMEG_6742	conserved hypothetical protein	1.01	8.99E-01	1.14	3.72E-01	1.68	1.91E-01
MSMEG_6743	lactoylglutathione lyase	0.41	5.32E-04	0.96	7.25E-01	0.98	6.63E-01
MSMEG_6744	oxidoreductase FAD/NAD	0.69	9.15E-05	1.10	4.07E-01	1.12	5.70E-02
MSMEG_6745	transcriptional regulator, GntR family protein	0.62	5.87E-03	0.92	1.12E-01	0.84	2.31E-01
MSMEG_6746	oxidoreductase, aldo/keto reductase family	0.91	2.19E-01	0.76	1.31E-02	0.80	2.25E-01
MSMEG_6747	oxidoreductase	3.95	2.87E-04	1.45	3.36E-01	0.81	2.19E-01
MSMEG_6748	ATPase component of ABC transporters with duplicated ATPase domains	0.78	6.44E-02	0.95	5.13E-01	1.90	1.02E-02
MSMEG_6749	conserved hypothetical protein	0.50	1.23E-04	1.08	4.56E-01	1.39	2.42E-01
MSMEG_6750	hypothetical protein	0.49	2.92E-03	1.03	8.12E-01	1.22	1.49E-01

MSMEG_6751	hypothetical protein		1.87	1.32E-01	1.56	8.70E-02	0.93	4.45E-01
MSMEG_6752	endoglucanase A		0.42	4.32E-05	0.87	2.70E-01	0.82	1.99E-01
MSMEG_6753	oxidoreductase, short chain dehydrogenase/reductase family protein		3.03	3.58E-02	1.00	9.78E-01	0.64	8.97E-02
MSMEG_6754	MaoC like domain protein		0.48	5.24E-04	1.04	8.57E-01	0.78	1.95E-01
MSMEG_6755	isoflavone reductase		2.42	6.89E-03	1.10	6.93E-01	0.55	1.43E-02
MSMEG_6756	glycerol kinase	*glpK*	0.84	2.43E-02	1.12	2.66E-01	1.50	9.44E-02
MSMEG_6757	glycerol operon regulatory protein		2.27	1.94E-03	0.84	1.85E-01	0.55	2.18E-03
MSMEG_6758	transport integral membrane protein		2.18	2.84E-03	0.71	2.09E-02	0.24	1.92E-03
MSMEG_6759	glycerol kinase	*glpK*	2.25	2.93E-02	0.99	9.14E-01	0.35	1.13E-02
MSMEG_6760	conserved hypothetical protein		0.81	1.74E-01	0.90	6.76E-01	1.02	7.69E-01
MSMEG_6761	glycerol-3-phosphate dehydrogenase 2		1.92	4.05E-03	0.86	4.34E-04	0.23	3.34E-03
MSMEG_6762	transcriptional regulator		0.79	1.15E-01	0.87	1.14E-01	6.07	1.03E-01
MSMEG_6763	oxidoreductase		1.25	5.91E-02	1.06	4.63E-01	1.71	1.80E-01
MSMEG_6764	transcriptional regulator, TetR family protein		0.94	9.03E-02	2.03	1.00E-01	1.26	3.29E-01
MSMEG_6765	ABC-2 type transporter superfamily protein		1.18	3.82E-01	1.24	4.59E-01	1.12	3.32E-01
MSMEG_6766	ABC transporter, ATP-binding protein, NodI family protein		0.69	1.60E-02	1.29	7.41E-02	1.13	2.05E-01
MSMEG_6767	mycocerosic acid synthase		1.02	7.15E-01	1.39	1.21E-01	0.75	9.73E-02
MSMEG_6768	halogenase		0.89	5.19E-01	1.79	6.31E-02	0.97	9.05E-01
MSMEG_6769	transporter, monovalent cation:proton antiporter-2 (CPA2) family protein		0.81	5.35E-02	1.10	5.76E-01	1.04	7.05E-01
MSMEG_6770	conserved hypothetical protein		1.65	1.39E-01	1.82	3.27E-01	0.90	3.39E-01
MSMEG_6771	conserved hypothetical protein		1.13	4.93E-01	1.67	3.10E-01	0.99	5.26E-01
MSMEG_6772	homoserine O-acetyltransferase		4.29	1.48E-04	0.63	5.15E-02	0.41	1.04E-02
MSMEG_6773	CaiB/BaiF family protein		2.29	5.16E-03	0.54	1.30E-02	0.90	6.23E-02
MSMEG_6774	enoyl-CoA hydratase		2.44	1.55E-02	0.94	4.64E-01	1.03	1.92E-01
MSMEG_6775	major facilitator superfamily protein transporter		5.95	2.85E-03	0.73	8.19E-03	1.28	4.80E-01
MSMEG_6776	transcriptional regulator, LysR family protein		4.36	2.55E-02	0.80	1.12E-01	2.05	4.52E-02
MSMEG_6777	probable enoyl-CoA hydratase		2.54	3.49E-03	0.67	1.78E-02	1.76	1.91E-01
MSMEG_6778	hypothetical protein		2.08	5.41E-04	0.59	3.73E-04	1.36	4.15E-02
MSMEG_6779	conserved hypothetical protein, putative		1.82	2.85E-04	1.02	7.19E-01	1.59	9.28E-04
MSMEG_6780	phenazine biosynthesis protein PhzF		1.50	1.09E-02	0.90	2.88E-01	1.44	1.26E-01
MSMEG_6781	hypothetical protein		1.36	2.25E-01	1.20	5.18E-01	0.99	8.65E-01
MSMEG_6782	hypothetical protein		1.15	2.50E-01	1.27	1.45E-01	1.17	4.11E-01
MSMEG_6783	integral membrane protein		0.82	3.99E-01	0.96	8.45E-01	1.92	5.13E-02
MSMEG_6784	beta-lactamase		1.05	7.90E-01	1.05	7.36E-01	1.25	1.42E-01
MSMEG_6785	triosephosphate isomerase	*tpiA*	1.04	7.73E-01	1.55	1.31E-01	1.62	6.78E-02

MSMEG_6786	conserved hypothetical protein		1.37	2.16E-01	1.12	3.58E-02	0.96	3.92E-01
MSMEG_6787	ribose 5-phosphate isomerase		1.30	7.92E-02	1.15	1.88E-01	1.18	4.02E-01
MSMEG_6788	dihydroxyacetone kinase		1.14	1.66E-01	1.41	3.40E-02	1.76	1.54E-01
MSMEG_6789	transcriptional regulator, GntR family protein		0.81	4.06E-02	1.12	3.71E-01	0.92	3.36E-01
MSMEG_6790	AP endonuclease, family protein 2		2.34	1.67E-02	1.17	3.00E-01	0.22	2.83E-03
MSMEG_6791	3-hydroxybutyryl-CoA dehydrogenase		2.68	5.49E-02	1.14	3.08E-01	0.12	4.79E-05
MSMEG_6792	inner membrane permease YgbN		2.03	1.10E-01	0.88	2.47E-01	1.88	5.92E-01
MSMEG_6793	hypothetical protein		1.09	3.54E-01	1.25	1.63E-01	0.80	4.25E-01
MSMEG_6794	DNA-binding protein		2.00	2.87E-02	1.50	2.40E-01	1.03	4.11E-01
MSMEG_6795	enoyl-CoA hydratase/isomerase family protein		1.24	3.94E-01	1.04	8.87E-01	1.12	5.28E-01
MSMEG_6796	hypothetical protein		0.76	3.83E-01	0.96	6.16E-01	1.08	1.23E-01
MSMEG_6797	carboxymuconolactone decarboxylase		1.68	6.86E-04	0.75	1.74E-02	0.70	7.11E-03
MSMEG_6798	ribose operon repressor	*rbsR*	0.94	5.01E-01	1.80	1.44E-01	0.89	8.95E-02
MSMEG_6799	aldo/keto reductase; this gene contains a frame shift which is not the result of sequencing error; identified by match to protein family HMM PF00248		1.24	2.47E-01	1.18	7.37E-02	0.93	6.57E-01
MSMEG_6800	phosphoribulokinase/uridine kinase		1.22	3.31E-02	1.56	1.99E-01	0.78	3.44E-02
MSMEG_6801	kinase, pfkB family protein		1.32	2.47E-02	1.52	2.02E-01	0.77	1.40E-01
MSMEG_6802	ABC transporter ATP-binding protein		1.33	9.77E-02	1.22	3.85E-01	0.55	4.05E-03
MSMEG_6803	ribose transport system permease protein RbsC		1.47	5.53E-02	1.02	8.90E-01	0.30	2.28E-04
MSMEG_6804	sugar ABC transporter substrate-binding protein		1.95	1.22E-02	1.17	3.20E-01	0.29	9.08E-05
MSMEG_6805	beta-lactamase		1.33	1.56E-01	0.81	2.51E-02	1.25	9.28E-02
MSMEG_6806	excinuclease ABC, A subunit	*uvrA*	1.17	2.55E-01	2.12	1.21E-01	1.06	6.53E-01
MSMEG_6807	transcriptional regulator, TetR family protein		1.49	8.32E-02	1.25	2.99E-01	0.90	6.72E-01
MSMEG_6808	hypothetical protein		1.52	3.57E-01	1.14	5.44E-01	0.96	5.99E-02
MSMEG_6809	CbbQ protein	*cbbQ*	1.13	2.04E-01	1.00	9.77E-01	1.04	7.92E-01
MSMEG_6810	nitric oxide reductase activase protein; this gene contains a frame shift which is not the result of sequencing error; identified by match to protein family HMM PF00092		0.96	8.45E-01	1.05	5.43E-01	1.06	3.12E-01
MSMEG_6811	conserved hypothetical protein		2.91	2.18E-03	2.09	1.64E-01	0.93	6.90E-01
MSMEG_6812	major facilitator superfamily; this gene contains a frame shift which is not the result of sequencing error; identified by match to protein family HMM PF00083		0.97	8.75E-01	8.55	1.32E-01	1.02	6.12E-01
MSMEG_6813	amidohydrolase 2		1.02	8.81E-01	4.31	1.10E-01	0.96	5.16E-01
MSMEG_6814	helix-turn-helix, Fis-type		1.03	4.10E-01	1.06	5.60E-01	1.30	1.86E-01

MSMEG_6815	secreted protein		1.20	3.75E-01	1.11	4.72E-01	1.28	4.54E-01
MSMEG_6816	molybdopterin oxidoreductase		0.34	1.77E-02	5.65	2.58E-01	0.65	2.95E-01
MSMEG_6817	RNA polymerase sigma factor, sigma-70 family protein		2.15	7.51E-04	3.70	1.05E-01	0.65	1.35E-01
MSMEG_6818	dehydrogenase		1.52	1.15E-02	3.55	1.21E-01	0.66	1.36E-01
MSMEG_6819	conserved domain protein; this gene contains a frame shift which is not the result of sequencing error		0.84	4.31E-02	0.88	7.18E-02	1.15	1.90E-01
MSMEG_6819	conserved domain protein; this gene contains a frame shift which is not the result of sequencing error		1.18	1.86E-01	1.52	1.37E-01	1.00	9.17E-01
MSMEG_6820	acid phosphatase SurE	*surE*	1.22	3.66E-01	1.11	2.80E-01	1.45	2.77E-01
MSMEG_6821	NLP/P60 family protein		0.66	8.96E-04	5.65	9.16E-02	17.26	3.61E-01
MSMEG_6822	beta-lactamase		0.84	2.31E-01	1.41	8.97E-02	1.56	4.34E-02
MSMEG_6823	short-chain dehydrogenase/reductase SDR		1.42	5.56E-03	4.66	8.17E-02	0.49	9.91E-04
MSMEG_6824	MarR-family protein regulatory protein		0.82	8.25E-02	0.95	6.03E-01	1.03	8.02E-01
MSMEG_6825	caax amino protease family protein, putative		1.61	1.52E-01	0.79	1.15E-01	1.01	3.75E-01
MSMEG_6826	L-lactate permease		7.97	1.82E-03	0.79	1.05E-01	0.18	6.96E-04
MSMEG_6827	hypothetical protein		1.10	4.15E-01	1.12	5.88E-01	1.11	2.74E-01
MSMEG_6828	transcriptional regulator		1.07	7.71E-01	0.93	6.22E-01	1.08	2.96E-01
MSMEG_6829	transcriptional regulatory protein		1.41	2.84E-03	0.80	8.57E-03	0.90	2.15E-01
MSMEG_6830	conserved hypothetical protein		0.91	2.03E-01	0.96	4.94E-01	1.21	3.90E-01
MSMEG_6831	hydrolase, alpha/beta hydrolase fold family protein		5.43	5.10E-02	0.90	2.33E-01	1.20	5.15E-01
MSMEG_6832	conserved hypothetical protein		1.45	1.34E-01	1.05	8.78E-01	0.85	3.28E-01
MSMEG_6833	alcohol dehydrogenase		1.59	1.11E-01	1.91	1.33E-01	1.21	2.28E-01
MSMEG_6834	alcohol dehydrogenase		2.33	4.67E-02	1.44	2.12E-01	1.92	3.68E-01
MSMEG_6835	Fatty acid desaturase		1.16	2.37E-01	1.00	9.50E-01	1.11	3.76E-01
MSMEG_6836	putative oxidoreductase		1.21	2.55E-01	1.06	5.60E-01	2.84	3.79E-01
MSMEG_6837	haloalkane dehalogenase		2.08	7.15E-03	0.79	1.44E-02	1.07	5.60E-01
MSMEG_6838	putative esterase		1.81	1.94E-01	1.64	1.58E-01	0.95	4.00E-01
MSMEG_6839	transcriptional regulator, AraC family protein		1.47	1.52E-01	1.40	2.94E-02	1.05	7.46E-01
MSMEG_6840	LysR-family protein transcriptional regulator		0.98	7.51E-01	0.98	7.84E-01	1.18	1.78E-01
MSMEG_6841	hypothetical protein		1.24	5.21E-01	1.22	4.80E-01	1.17	1.12E-01
MSMEG_6842	2,2-dialkylglycine decarboxylase		1.17	1.37E-01	1.11	3.58E-01	0.88	1.62E-01
MSMEG_6843	NAD-dependent epimerase/dehydratase		2.24	8.60E-03	0.73	9.87E-03	0.68	2.10E-01
MSMEG_6844	trap-t family protein transporter, dctp		1.12	1.55E-01	1.53	7.14E-02	1.02	7.57E-01
MSMEG_6845	trap dicarboxylate transporter, dctm subunit		1.91	5.54E-02	1.25	3.57E-01	0.96	7.92E-01

MSMEG_6846	putative C4 decarboxylate transport protein	0.78	3.87E-03	1.05	7.06E-01	1.13	4.20E-01
MSMEG_6847	conserved hypothetical protein	2.96	4.19E-02	1.13	5.15E-01	1.71	1.18E-01
MSMEG_6848	putative oxidoreductase	3.55	6.78E-04	0.91	4.89E-01	1.12	4.90E-01
MSMEG_6849	LysR-family protein transcriptional regulator	1.38	1.65E-01	0.94	2.76E-01	1.53	1.68E-01
MSMEG_6850	alpha/beta hydrolase	1.87	3.25E-02	0.81	1.19E-01	0.74	1.82E-01
MSMEG_6851	phosphatidylethanolamine-binding protein	1.25	3.93E-01	1.08	2.54E-01	1.03	3.86E-01
MSMEG_6852	putative carboxylesterase/lipase	1.52	1.74E-02	0.99	8.02E-01	1.00	9.25E-01
MSMEG_6853	3-isopropylmalate dehydratase small subunit	1.08	4.28E-01	11.98	3.70E-01	1.24	3.33E-01
MSMEG_6854	3-isopropylmalate dehydratase large subunit	1.23	1.93E-01	2.86	2.84E-01	1.12	4.63E-01
MSMEG_6855	carboxyvinyl-carboxyphosphonate phosphorylmutase	1.18	4.99E-01	1.56	2.61E-01	0.92	5.14E-01
MSMEG_6856	MmgE/PrpD family protein	1.03	6.19E-01	2.02	2.32E-01	0.96	6.07E-01
MSMEG_6857	putative transcription regulator	1.35	1.13E-01	1.12	4.97E-01	1.55	2.54E-01
MSMEG_6858	epoxide hydrolase 1	2.40	1.74E-01	1.62	3.53E-01	1.15	4.56E-01
MSMEG_6859	oxidoreductase	3.01	3.74E-03	1.59	2.26E-01	1.06	2.46E-01
MSMEG_6860	conserved hypothetical protein	3.88	1.18E-03	0.71	9.57E-04	1.55	3.69E-01
MSMEG_6861	cupin domain protein	1.23	9.79E-02	1.26	2.74E-01	0.97	7.17E-01
MSMEG_6862	putative transcription regulator	1.26	1.50E-02	0.96	6.33E-01	0.93	5.44E-01
MSMEG_6863	class II aldolase/adducin domain protein	1.42	3.19E-03	0.97	6.62E-01	0.83	1.83E-01
MSMEG_6864	putative transcription regulator	1.07	5.94E-01	0.91	1.21E-01	0.92	4.95E-01
MSMEG_6865	ABC transporter solute binding lipoprotein	1.71	5.04E-03	0.85	8.46E-02	1.06	4.65E-01
MSMEG_6866	dipeptide transport system permease protein DppB	1.52	1.28E-03	0.82	9.35E-02	1.02	8.59E-01
MSMEG_6867	oligopeptide ABC transporter integral membrane protein	1.53	7.25E-02	0.82	1.59E-03	1.00	9.63E-01
MSMEG_6868	oligopeptide ABC transporter ATP-binding protein; this gene contains a frame shift which is not the result of sequencing error; identified by match to protein family HMM PF00005	2.20	2.21E-02	0.74	2.07E-02	1.24	1.93E-01
MSMEG_6868	oligopeptide ABC transporter ATP-binding protein; this gene contains a frame shift which is not the result of sequencing error; identified by match to protein family HMM PF00005	1.27	1.24E-01	0.88	4.02E-02	0.99	9.29E-01
MSMEG_6869	regulatory protein	1.64	4.56E-02	0.87	1.67E-01	1.11	3.25E-01
MSMEG_6870	creatinine amidohydrolase	1.29	4.83E-02	0.86	6.34E-02	0.98	7.93E-01
MSMEG_6871	isomerase	1.12	2.48E-01	1.06	3.27E-01	1.15	5.00E-01
MSMEG_6872	beta-lactamase	1.06	6.03E-01	0.98	7.17E-01	1.02	5.60E-01

MSMEG_6873	enoyl-CoA hydratase/isomerase; this gene contains a frame shift which is not the result of sequencing error	0.40	4.91E-07	1.46	9.26E-02	0.93	1.36E-01
MSMEG_6873	enoyl-CoA hydratase/isomerase; this gene contains a frame shift which is not the result of sequencing error	0.69	2.92E-02	1.21	2.48E-01	1.17	4.01E-01
MSMEG_6874	aldehyde dehydrogenase	0.30	1.41E-04	0.94	5.81E-01	1.06	5.41E-01
MSMEG_6875	endoribonuclease L-PSP family protein	0.72	1.14E-02	0.93	4.83E-01	1.02	7.24E-01
MSMEG_6876	branched chain amino acid transport ATP-binding protein	0.65	5.12E-02	1.01	8.69E-01	0.78	1.53E-01
MSMEG_6877	branched-chain amino acid transport system ATP-binding protein	0.54	4.35E-02	1.14	1.86E-01	1.22	3.96E-01
MSMEG_6878	inner-membrane translocator	0.24	3.11E-05	1.17	5.93E-01	1.02	7.72E-01
MSMEG_6879	integral membrane protein of the ABC-type Nat permease for neutral amino acids NatD	0.34	3.14E-04	0.98	8.00E-01	0.90	2.19E-01
MSMEG_6880	hydrophobic amino acid ABC transporter, putative	0.15	1.96E-05	1.13	3.62E-01	0.79	1.39E-01
MSMEG_6881	transcriptional regulator, GntR family protein	0.47	4.03E-03	1.04	8.13E-01	0.75	1.35E-01
MSMEG_6882	LysR-family protein transcriptional regulator	1.69	8.09E-02	0.82	1.76E-01	1.34	1.51E-01
MSMEG_6883	hypothetical protein; this region contains one or more premature stops and/or frameshifts which are not the result of sequencing error; identified by Glimmer2; putative	1.53	1.79E-03	1.06	5.90E-01	1.21	3.12E-01
MSMEG_6884	NADP oxidoreductase, coenzyme f420-dependent:6-phosphogluconate dehydrogenase, nad-binding, putative	1.70	8.44E-02	1.11	4.39E-01	0.89	3.97E-01
MSMEG_6885	MmcI protein	1.37	4.49E-03	0.91	2.31E-01	0.45	4.03E-04
MSMEG_6886	transcriptional regulator	1.66	9.30E-03	0.89	8.60E-02	1.04	7.45E-01
MSMEG_6887	amidohydrolase family protein	1.10	3.15E-01	1.34	1.23E-01	1.08	4.01E-01
MSMEG_6888	transposase subunit	0.70	1.05E-03	0.80	4.23E-02	0.98	5.22E-01
MSMEG_6889	transposase	1.95	3.38E-01	1.05	6.06E-01	0.88	1.71E-01
MSMEG_6890	antibiotic biosynthesis monooxygenase domain protein	1.39	6.96E-02	1.14	3.76E-01	2.03	2.09E-01
MSMEG_6891	ribonuclease H; this region contains one or more premature stops and/or frameshifts which are not the result of sequencing error	1.17	3.26E-01	0.99	9.10E-01	1.10	5.55E-01
MSMEG_6892	replicative DNA helicase	0.71	2.39E-03	1.02	8.34E-01	1.67	2.35E-02
MSMEG_6893	hypothetical protein	0.95	8.58E-01	1.11	3.52E-01	1.18	5.37E-01

MSMEG_6894	ribosomal protein L9	*rplI*	0.27	5.53E-07	0.79	1.08E-01	0.88	2.93E-01
MSMEG_6895	ribosomal protein S18	*rpsR*	0.44	1.17E-05	0.82	3.39E-02	0.39	1.61E-05
MSMEG_6896	single-stranded DNA-binding protein		0.54	4.46E-04	0.91	2.38E-01	0.86	4.42E-02
MSMEG_6897	ribosomal protein S6	*rpsF*	0.40	2.65E-04	0.89	3.41E-01	0.80	4.93E-02
MSMEG_6898	hypothetical protein		1.04	8.38E-01	1.00	9.84E-01	1.57	1.50E-01
MSMEG_6899	conserved hypothetical protein		0.50	2.65E-03	1.02	5.92E-01	1.37	1.46E-01
MSMEG_6900	penicillin-binding protein 1		0.52	1.38E-04	0.88	1.90E-01	2.21	1.38E-02
MSMEG_6901	conserved hypothetical protein		1.28	1.00E-02	0.87	1.36E-01	1.31	2.40E-01
MSMEG_6902	putative membrane protein		0.60	7.75E-02	1.20	1.50E-01	0.86	1.62E-01
MSMEG_6903	transcriptional regulator, PadR family protein		1.30	6.59E-03	1.03	8.89E-01	0.54	6.08E-03
MSMEG_6904	myo-inositol-1-phosphate synthase		0.90	1.03E-01	0.84	4.53E-01	0.73	1.72E-02
MSMEG_6906	putative hydrolase		0.71	2.20E-03	0.80	1.45E-02	0.89	2.18E-01
MSMEG_6907	MmcI protein		1.85	6.64E-02	0.82	1.68E-01	0.71	2.17E-03
MSMEG_6908	putative HTH-type transcriptional regulator		1.16	1.41E-01	0.95	2.60E-01	0.97	5.80E-01
MSMEG_6909	amino acid ABC transporter, permease protein, 3-TM region, His/Glu/Gln/Arg/opine family protein		1.15	1.77E-01	1.01	9.09E-01	0.90	4.50E-01
MSMEG_6911	ABC transporter, ATP-binding protein GluA		1.13	1.95E-02	0.96	5.91E-01	2.46	3.62E-01
MSMEG_6913	putative transcriptional regulatory protein		1.50	6.10E-03	0.88	1.73E-01	0.51	1.13E-04
MSMEG_6914	conserved hypothetical protein		1.27	9.56E-03	1.00	9.33E-01	1.36	3.67E-01
MSMEG_6916	short chain dehydrogenase		0.61	1.35E-03	0.87	3.29E-02	2.58	1.39E-02
MSMEG_6917	leucyl-tRNA synthetase	*leuS*	0.43	2.80E-06	1.09	3.64E-01	2.57	8.22E-02
MSMEG_6919	proline-rich 28 kDa antigen		1.78	2.56E-02	0.70	6.89E-02	0.97	9.22E-01
MSMEG_6920	hypothetical protein		0.92	1.26E-01	0.72	3.55E-02	1.25	1.74E-01
MSMEG_6921	conserved hypothetical protein		2.13	1.42E-03	0.80	5.69E-02	0.96	7.54E-01
MSMEG_6922	transporter, major facilitator family protein		0.84	1.39E-02	0.86	1.34E-01	3.41	6.80E-02
MSMEG_6923	conserved hypothetical protein		0.90	2.02E-01	0.88	9.49E-02	1.53	7.71E-02
MSMEG_6925	conserved hypothetical protein		1.17	6.15E-01	1.16	3.25E-01	1.06	6.47E-01
MSMEG_6926	tRNA adenylyltransferase		0.27	2.27E-05	0.99	8.77E-01	1.83	1.25E-01
MSMEG_6927	MutT/nudix family protein		0.69	1.03E-02	0.80	2.73E-02	1.77	1.36E-01
MSMEG_6928	conserved hypothetical protein		0.68	5.57E-03	0.90	5.19E-01	1.49	2.05E-01
MSMEG_6929	integral membrane protein MviN, putative		0.56	2.51E-02	1.29	9.09E-02	1.09	4.57E-01
MSMEG_6930	hypothetical protein		1.26	3.51E-01	1.05	7.51E-01	1.31	4.77E-01
MSMEG_6931	RNA polymerase sigma-70 factor		1.07	6.25E-01	1.40	1.47E-01	0.81	2.67E-01
MSMEG_6932	conserved hypothetical protein		0.73	1.02E-02	0.93	3.01E-01	1.17	4.72E-01
MSMEG_6933	thioredoxin-disulfide reductase	*trxB*	1.06	3.28E-01	1.30	1.70E-01	1.50	3.63E-02
MSMEG_6934	thioredoxin	*trx*	2.39	1.74E-03	0.80	5.19E-03	0.90	1.66E-01
MSMEG_6935	N-acetylmuramoyl-L-alanine amidase		1.10	2.24E-01	0.87	1.56E-01	1.10	1.95E-01

MSMEG_6936	conserved hypothetical protein		1.71	3.06E-05	0.68	2.77E-02	1.47	1.61E-01
MSMEG_6938	ParB-like partition proteins		1.25	5.81E-03	0.84	1.57E-02	1.05	5.82E-01
MSMEG_6939	Soj family protein		0.74	2.47E-02	1.12	1.68E-01	1.39	1.11E-02
MSMEG_6940	methyltransferase GidB	*gidB*	1.10	1.96E-01	1.06	4.65E-01	1.62	5.55E-02
MSMEG_6941	R3H domain-containing protein		0.69	1.49E-03	0.93	4.26E-01	1.29	2.04E-02
MSMEG_6942	membrane protein OxaA		0.59	1.92E-04	0.85	4.94E-02	1.02	9.16E-01
MSMEG_6944	conserved hypothetical protein, putative		0.57	2.00E-04	0.86	2.17E-02	0.81	9.62E-02
MSMEG_6945	ribonuclease P protein component	*rnpA*	1.32	7.67E-03	0.91	3.13E-01	0.79	1.05E-01
MSMEG_6946	ribosomal protein L34	*rpmH*	1.08	1.84E-01	0.89	1.52E-01	0.69	1.22E-01
MSMEG_6947	chromosomal replication initiator protein DnaA	*dnaA*	0.98	6.98E-01	0.90	2.71E-01	0.52	3.02E-05
