# Supplementary material for: Unique Flexibility in Energy Metabolism Allows Mycobacteria to Combat Starvation and Hypoxia
Source: PLoS One. 2010 Jan 7;5(1):e8614. doi: 10.1371/journal.pone.0008614 (PMC2799521; doi:10.1371/journal.pone.0008614)
Supplement: Table S1 — Expression ratios of genes involved in energy metabolism. Expression ratios with a p-value<0.05 are indicated with bold numbers. (0.22 MB DOC) [file pone.0008614.s002.doc]

Table S1: Expression ratios of genes involved in energy metabolism.

Expression ratios with a p-value < 0.05 are indicated with bold numbers.

|  |  |  |  |  |  |
| --- | --- | --- | --- | --- | --- |
| **Gene ID** | **Gene name** | **Slow/fast^1^** | **Hypoxia^2^ 2.5% O_2_** | **Hypoxia^2^ 0.6% O_2_** | **Description** |
|  |  |  |  |  |  |
| **Putative succinate dehydrogenase** | | | |  |  |
| MSMEG_0417 |  | **4.0** | 0.9 | **0.14** | succinate dehydrogenase/fumarate reductase iron sulfur subunit |
| MSMEG_0418 |  | 4.4 | **0.7** | **0.09** | succinate dehydrogenase flavoprotein subunit |
| MSMEG_0419 |  | 4.4 | 1.1 | **0.03** | integral membrane protein/succinate dehydrogenase membrane anchor subunit |
| MSMEG_0420 |  | **9.9** | 1.3 | **0.02** | conserved hypothetical protein |
|  |  |  |  |  |  |
| **Carbon monoxide dehydrogenase** | | | |  |  |
| MSMEG_0744 |  | **8.5** | 2.4 | **0.2** | carbon monoxide dehydrogenase medium chain |
| MSMEG_0745 |  | **3.4** | 1.9 | **0.3** | [2Fe-2S] binding domain protein |
| MSMEG_0746 |  | **6.9** | 2.1 | **0.2** | carbon-monoxide dehydrogenase, large subunit |
| MSMEG_0747 |  | 5.8 | 1.9 | **0.5** | carbon monoxide dehydrogenase F protein |
| MSMEG_0749 |  | **2.0** | 1.6 | 0.9 | carbon monoxide dehydrogenase subunit G |
|  |  |  |  |  |  |
| **Succinate dehydrogenase** | | |  |  |  |
| MSMEG_1669 | *sdhB* | **0.3** | 0.9 | 2.5 | succinate dehydrogenase, iron-sulfur protein |
| MSMEG_1670 | *sdhA* | **0.3** | 1.0 | **2.0** | succinate dehydrogenase, flavoprotein subunit |
| MSMEG_1671 |  | **0.4** | 0.9 | **2.1** | succinate dehydrogenase hydrophobic membrane anchor protein SdhD |
| MSMEG_1672 | *sdhC* | **0.4** | 0.9 | 1.8 | succinate dehydrogenase, cytochrome b556 subunit |
|  |  |  |  |  |  |
| **Propane/methane monooxygenase** | | | | |  |
| MSMEG_1971 |  | **50.7** | 1.2 | **0.08** | propane monooxygenase hydroxylase large subunit |
| MSMEG_1972 |  | **30.9** | 1.9 | **0.07** | methane monooxygenase component C |
| MSMEG_1973 |  | **22.8** | 1.5 | **0.04** | propane monooxygenase hydroxylase small subunit (contains framshift) |
| MSMEG_1974 |  | **25.0** | 1.6 | **0.04** | propane monooxygenase coupling protein |
| MSMEG_1975 |  | **37.9** | 1.7 | **0.03** | amidohydrolase 2 |
| MSMEG_1976 |  | **20.5** | 1.2 | **0.07** | conserved hypothetical protein |
| MSMEG_1977 |  | **17.9** | 1.3 | **0.08** | alcohol dehydrogenase |
| MSMEG_1978 | *groL* | **20.6** | 1.2 | **0.1** | chaperonin GroL |
|  |  |  |  |  |  |
| **NADH-Menaquinone oxidoreductases** | | | | |  |
| MSMEG_2050 | *nuoN* | 3.4 | 1.0 | **0.4** | NADH-quinone oxidoreductase, N subunit |
| MSMEG_2051 | *nuoM* | **4.3** | 1.0 | **0.2** | NADH-quinone oxidoreductase, M subunit |
| MSMEG_2052 | *nuoL* | **3.5** | 0.8 | 0.5 | NADH-quinone oxidoreductase, L subunit |
| MSMEG_2053 | *nuoK* | **5.0** | 0.8 | **0.1** | NADH-quinone oxidoreductase, k subunit |
| MSMEG_2054 |  | 4.5 | 0.9 | **0.2** | NADH dehydrogenase subunit j |
| MSMEG_2055 | *nuoI* | 2.4 | 1.0 | 0.7 | NADH-quinone oxidoreductase, I subunit |
| MSMEG_2056 | *nuoH* | 6.1 | 1.0 | **0.2** | NADH-quinone oxidoreductase, H subunit |
| MSMEG_2057 | *nuoG* | **5.2** | 1.0 | **0.1** | NADH-quinone oxidoreductase, G subunit |
| MSMEG_2058 | *nuoF* | **19.1** | 0.8 | **0.1** | NADH-quinone oxidoreductase, F subunit |
| MSMEG_2059 |  | **4.2** | 1.1 | **0.2** | NADH-quinone oxidoreductase chain e |
| MSMEG_2060 | *nuoD* | 4.9 | 0.7 | 0.5 | NADH-quinone oxidoreducatase, D subunit |
| MSMEG_2061 |  | 3.1 | 1.1 | **0.2** | NADH-quinone oxidoreductase chain c |
| MSMEG_2062 | *nuoB* | **7.7** | 0.8 | **0.2** | NADH-quinone oxidoreductase, B subunit |
| MSMEG_2063 | *nuoA* | 6.6 | 0.9 | 0.7 | NADH-quinone oxidoreductase, a subunit |
| MSMEG_2064 |  | **4.0** | 0.8 | **0.6** | two-component system response regulator |
|  |  |  |  |  |  |
| MSMEG_4812 |  | 1.4 | 1.0 | 1.4 | respiratory-chain NADH dehydrogenase domain, 51 kda subunit |
|  |  |  |  |  |  |
| MSMEG_3621 | *ndh* | 0.9 | 2.0 | **2.2** | NADH dehydrogenase |
|  |  |  |  |  |  |
| **Nitrate reduction** | |  |  |  |  |
| MSMEG_5136 |  | **3.2** | **5.8** | **3.0** | helix-turn-helix motif |
| MSMEG_5137 | *narI* | **1.8** | 0.9 | 0.7 | respiratory nitrate reductase, gamma subunit |
| MSMEG_5138 | *narJ* | 1.4 | 1.6 | 0.9 | nitrate reductase molybdenum cofactor assembly chaperone |
| MSMEG_5139 | *narH* | 1.5 | **1.2** | 1.0 | nitrate reductase, beta subunit |
| MSMEG_5140 | *narG* | 1.9 | 1.1 | 0.9 | nitrate reductase, alpha subunit |
| MSMEG_5141 | *narK* | 4.0 | 1.2 | 0.8 | nitrate/nitrite transporter protein |
|  |  |  |  |  |  |
| MSMEG_2837 | *narB* | 1.4 | **1.5** | 1.0 | nitrate reductase NarB |
|  |  |  |  |  |  |
| **ATP-synthase** | |  |  |  |  |
| MSMEG_4935 | *atpC* | **0.5** | 0.9 | **2.9** | ATP synthase F1, epsilon subunit |
| MSMEG_4936 | *atpD* | **0.4** | 0.9 | **3.0** | ATP synthase F1, beta subunit |
| MSMEG_4937 | *atpG* | **0.4** | **1.2** | 2.5 | ATP synthase F1, gamma subunit |
| MSMEG_4938 | *atpA* | **0.7** | 0.8 | 1.6 | ATP synthase F1, alpha subunit |
| MSMEG_4939 |  | **0.6** | 1.0 | 1.5 | ATP synthase delta chain |
| MSMEG_4940 |  | 1.0 | 0.9 | **1.9** | ATP synthase F0, B subunit |
| MSMEG_4941 | *atpE* | **0.6** | 1.1 | **3.0** | ATP synthase F0, C subunit |
| MSMEG_4942 | *atpB* | **0.5** | 1.0 | 1.8 | ATP synthase F0, A subunit |
|  |  |  |  |  |  |
| **Cytochrome *bd*oxidase** | | |  |  |  |
| MSMEG_3230 | *cydC* | **0.5** | 1.0 | **93.3** | ABC transporter, CydDC cysteine exporter |
| MSMEG_3231 | *cydD* | **0.5** | 1.1 | **62.2** | ABC transporter, CydDC cysteine exporter |
| MSMEG_3232 | *cydB* | **0.2** | 1.0 | **159.3** | cytochrome D ubiquinol oxidase, subunit II |
| MSMEG_3233 | *cydA* | **0.3** | 0.9 | 48.9 | cytochrome D ubiquinol oxidase subunit 1 |
|  |  |  |  |  |  |
| MSMEG_5605 |  | 1.2 | 1.4 | 1.5 | cytochrome bd ubiquinol oxidase, subunit I |
| MSMEG_5606 |  | 1.2 | 2.5 | 1.2 | cytochrome bd-I oxidase subunit II |
|  |  |  |  |  |  |
| **Cytochrome *bc_1_* complex** | | |  |  |  |
| MSMEG_4261 |  | **0.4** | **0.8** | **2.8** | ubiquinol-cytochrome c reductase cytochrome c subunit |
| MSMEG_4262 |  | **0.4** | 0.9 | **6.0** | ubiquinol-cytochrome c reductase iron-sulfur subunit |
| MSMEG_4263 |  | **0.4** | 0.9 | **6.1** | ubiquinol-cytochrome c reductase cytochrome b subunit |
|  |  |  |  |  |  |
| **Cytochrome c Oxidase (*aa_3_*)** | | | |  |  |
| MSMEG_2320 | *ctaD* | **0.3** | 0.8 | **4.1** | cytochrome c oxidase, subunit I (contains a frameshift) |
| MSMEG_1040 | *ctaD* | 0.5 | 1.0 | **2.9** | cytochrome c oxidase, subunit I (contains a frameshift) |
| MSMEG_4437 | *ctaD* | 1.3 | 1.2 | 0.9 | cytochrome c oxidase, subunit I |
| MSMEG_4267 |  | **0.3** | 0.9 | **2.5** | probable cytochrome c oxidase polypeptide 4 |
| MSMEG_4268 |  | **0.3** | 0.9 | **2.7** | cytochrome c oxidase subunit 2 |
| MSMEG_4260 |  | **0.4** | **1.2** | **5.7** | cytochrome c oxidase subunit 3 |
| MSMEG_3105 | *cyoE* | **0.4** | 0.8 | **3.7** | protoheme IX farnesyltransferase |
| MSMEG_3117 |  | **0.3** | 1.0 | **12.0** | cytochrome aa3 controlling protein |
|  |  |  |  |  |  |
| **Cytochrome c biogenesis** | | |  |  |  |
| MSMEG_0971 |  | **0.3** | 1.1 | **5.7** | conserved hypothetical protein |
| MSMEG_0972 |  | **0.4** | 1.0 | **8.7** | cytochrome C biogenesis protein transmembrane region |
| MSMEG_0973 |  | **0.6** | 1.1 | **4.0** | conserved membrane protein |
| MSMEG_0974 | *ccsB* | **0.4** | 0.9 | **2.0** | cytochrome c-type biogenesis protein CcsB |
| MSMEG_3541 |  | 1.1 | 1.9 | 1.0 | cytochrome C biogenesis protein transmembrane region |
|  |  |  |  |  |  |
| **Menaquinone Pool** | |  |  |  |  |
| MSMEG_0988 | *menA* | 0.9 | 0.8 | 1.1 | 1,4-dihydroxy-2-naphthoate octaprenyltransferase |
| MSMEG_1075 | *menB* | 0.8 | 1.0 | 1.3 | naphthoate synthase |
| MSMEG_1103 | *menC* | 0.9 | 0.9 | 1.5 | O-succinylbenzoate synthase |
| MSMEG_1109 | *menD* | **0.8** | **0.8** | **1.5** | 2-oxoglutarate decarboxylase |
| MSMEG_1062 | *menE* | 0.9 | 0.9 | **2.1** | O-succinylbenzoic acid--CoA ligase |
| MSMEG_6439 | *menG* | **0.5** | 0.8 | 1.3 | regulator of ribonuclease activity A |
| MSMEG_1115 | *ubiE* | **0.5** | 1.2 | 1.2 | menaquinone biosynthesis methyltransferase UbiE |
| MSMEG_1925 | *entC* | 0.4 | 1.1 | 1.3 | isochorismate synthase DhbC |
| MSMEG_4524 | *mbtI* | 0.3 | 1.3 | 2.0 | anthranilate synthase component I |
|  |  |  |  |  |  |
| **Alanine dehydrogenase** | | |  |  |  |
| MSMEG_2659 | *ald* | **4.0** | 0.8 | 9.5 | alanine dehydrogenase |
|  |  |  |  |  |  |
| **Glycine dehydrogenase** | | |  |  |  |
| MSMEG_3642 |  | **0.5** | 1.0 | **5.7** | glycine dehydrogenase |
|  |  |  |  |  |  |
| **Proline dehydrogenase** | | |  |  |  |
| Msmeg_5117 |  | **4.2** | 1.4 | 3.5 | proline dehydrogenase |
| Msmeg_5119 | *pruA* | **8.7** | 1.6 | 3.2 | 1-pyrroline-5-carboxylate dehydrogenase |
|  |  |  |  |  |  |
| **D-amino acid dehydrogenase** | | | |  |  |
| MSMEG_1129 |  | 1.5 | 1.0 | 1.1 | D-amino-acid dehydrogenase |
| MSMEG_6291 |  | 4.5 | **0.8** | 3.9 | D-amino-acid dehydrogenase |
|  |  |  |  |  |  |
| **Malate dehydrogenase / Malic enzyme** | | | | |  |
| MSMEG_2613 | *mqo* | **0.8** | **0.9** | **3.7** | malate:quinone-oxidoreductase |
| MSMEG_5055 |  | **0.7** | 1.2 | **0.5** | NAD-dependent malic enzyme |
|  |  |  |  |  |  |
| **Pyruvate dehydrogenase complex** | | | |  |  |
| Msmeg_0903 |  | **0.4** | **1.1** | **0.7** | dihydrolipoamide dehydrogenase |
| MSMEG_2280 |  | 1.0 | 1.1 | **0.6** | pyruvate dehydrogenase |
| MSMEG_2471 |  | **3.3** | 0.9 | 1.3 | pyruvate dehydrogenase alpha subunit |
| MSMEG_3964 |  | 1.0 | 0.9 | **0.6** | pyruvate dehydrogenase |
| MSMEG_4283 | *sucB* | **0.8** | 1.0 | 1.4 | dihydrolipoamide succinyltransferase |
| MSMEG_4323 |  | **0.7** | 1.0 | **0.9** | pyruvate dehydrogenase E1 component |
| MSMEG_4710 |  | 8.2 | 1.1 | **1.4** | dihydrolipoamide acetyltransferase |
| MSMEG_4711 |  | **11.4** | 0.9 | 1.1 | pyruvate dehydrogenase E1 component subunit beta |
| MSMEG_4712 |  | **5.4** | 1.2 | **0.6** | pyruvate dehydrogenase E1 component, alpha subunit |
| MSMEG_5593 |  | 0.8 | 1.0 | 0.9 | pyruvate dehydrogenase |
|  |  |  |  |  |  |
| **D-lactate dehydrogenase** | | |  |  |  |
| MSMEG_2492 |  | **0.8** | 1.2 | 2.2 | D-lactate dehydrogenase |
|  |  |  |  |  |  |
| **Glycerol-3-phosphate dehydrogenase** | | | | |  |
| MSMEG_1140 |  | **2.2** | 1.1 | **0.4** | glycerol-3-phosphate dehydrogenase 2 [NAD(P)+] |
| MSMEG_1736 |  | 1.2 | 1.0 | 1.1 | glycerol-3-phosphate dehydrogenase 2 |
| MSMEG_2393 |  | **0.4** | 1.4 | **3.1** | NAD-dependent glycerol-3-phosphate dehydrogenase |
| MSMEG_4332 |  | 0.9 | 1.1 | 1.4 | glycerol-3-phosphate dehydrogenase 1 |
| MSMEG_6761 |  | **1.9** | **0.9** | **0.2** | glycerol-3-phosphate dehydrogenase 2 |
|  |  |  |  |  |  |
| MSMEG_2351 | *etfB* | **0.6** | 0.9 | **1.5** | electron transfer flavoprotein, beta subunit |
| MSMEG_2352 | *etfA* | **0.6** | **0.7** | 0.9 | electron transfer flavoprotein, alpha subunit |
|  |  |  |  |  |  |

^1^Gene expression ratio of slow growing (0.01 h^-1^) versus fast growing (0.15 h^-1^) chemostat cultures both running at 50% oxygen saturation.

^2^Gene expression ratio of slow growing (0.01h^-1^) chemostat culture at 50% oxygen saturation versus slow growing at 2.5% or 0.6% oxygen saturation.
