# Supplementary material for: Unique Flexibility in Energy Metabolism Allows Mycobacteria to Combat Starvation and Hypoxia
Source: PLoS One. 2010 Jan 7;5(1):e8614. doi: 10.1371/journal.pone.0008614 (PMC2799521; doi:10.1371/journal.pone.0008614)
Supplement: Table S2 — Primer sequences used for quantitative RT-PCR. (0.05 MB DOC) [file pone.0008614.s003.doc]

Table S2: Primer sequences used for quantitative RT-PCR

|  |  |  |  |  |  |
| --- | --- | --- | --- | --- | --- |
| Locus | Gene name | Primer sequence (5' to 3') | | | |
|  |  | forward | | reverse | |
| Msmeg_1669 | *sdhB* | GAACCCTCGCTCCAGTACAC | | GAGAAGGACCTGGTGGTGAA | |
| Msmeg_1971 |  | GTCCAGATGATCGACGAGGT | | GCCACCACCGTGAGATAGAT | |
| Msmeg_2058 | *nuoF* | AAGATCTGCAGTGCCTTGGT | | TTCACGCTGTATTCGCTGTC | |
| Msmeg_2262 | *hybA* | TGTACGCGTGTGCAGTTCTT | | GCTGACCTTCTGGGTCTTGA | |
| Msmeg_2719 |  | ATGTCGCGGTAGCTGAAGTT | | CCGACTACATGACACGGTTG | |
| Msmeg_2758 | *sigA* | GACTCTTCCTCGTCCCACAC | | GAAGACACCGACCTGGAACT | |
| Msmeg_3232 | *cydB* | GAACGACAGGTGGATCTGCT | | ATGTTCTCGGGCCTGTACCT | |
| Msmeg_4262 |  | GAGATGTCGCGTGAGGAACT | | CAAGGCCAGAAGAGGAACAC | |
| Msmeg_4891 | *ahpC* | TCGAAGTCCTCGTTCAGCTT | | GGTGACCAGTTTCCGGAGTA | |
| Msmeg_4937 | *atpG* | GAAGCTGAAGTAGCCCAACG | | ACCAACATGCTCACCGAACT | |
|  |  |  |  |  |  |
|  |  |  |  |  |  |
