# Supplementary material for: Unique Flexibility in Energy Metabolism Allows Mycobacteria to Combat Starvation and Hypoxia
Source: PLoS One. 2010 Jan 7;5(1):e8614. doi: 10.1371/journal.pone.0008614 (PMC2799521; doi:10.1371/journal.pone.0008614)
Supplement: Table S3 — Primer sequences used for deletion of msmeg_2719 and complementation. (0.03 MB DOC) [file pone.0008614.s004.doc]

Table S3: Primer sequences used for deletion of *msmeg_2719* and complementation.

|  |  |  |  |  |
| --- | --- | --- | --- | --- |
| Primer Name | Primer sequence (5' to 3') | | | |
| Hyd2.1 | AAATTTACTAGTGCACACCGTCACGCATCAG | | | |
| Hyd2.2 | CTTCGAGGAGGAGAACGATCGGGAGCA | | | |
| Hyd2.3 | TCTCCTCCTCGAAGTCGATCTTGGTGTA | | | |
| Hyd2.4 | AAATTTACTAGTTATTGGTGCGGGTTCGGTAA | | | |
| cHyd2f | AAATTTAAGCTTGCACTCGTCGGTCTTGGGA | | | |
| cHyd2r | AAATTTAAGCTTCATCGAGAATGTCGGAAACCT | | | |
|  |  |  |  |  |
|  |  |  |  |  |
