# Supplementary figures and images for: Unique Flexibility in Energy Metabolism Allows Mycobacteria to Combat Starvation and Hypoxia
Source: PLoS One. 2010 Jan 7;5(1):e8614. doi: 10.1371/journal.pone.0008614 (PMC2799521; doi:10.1371/journal.pone.0008614)

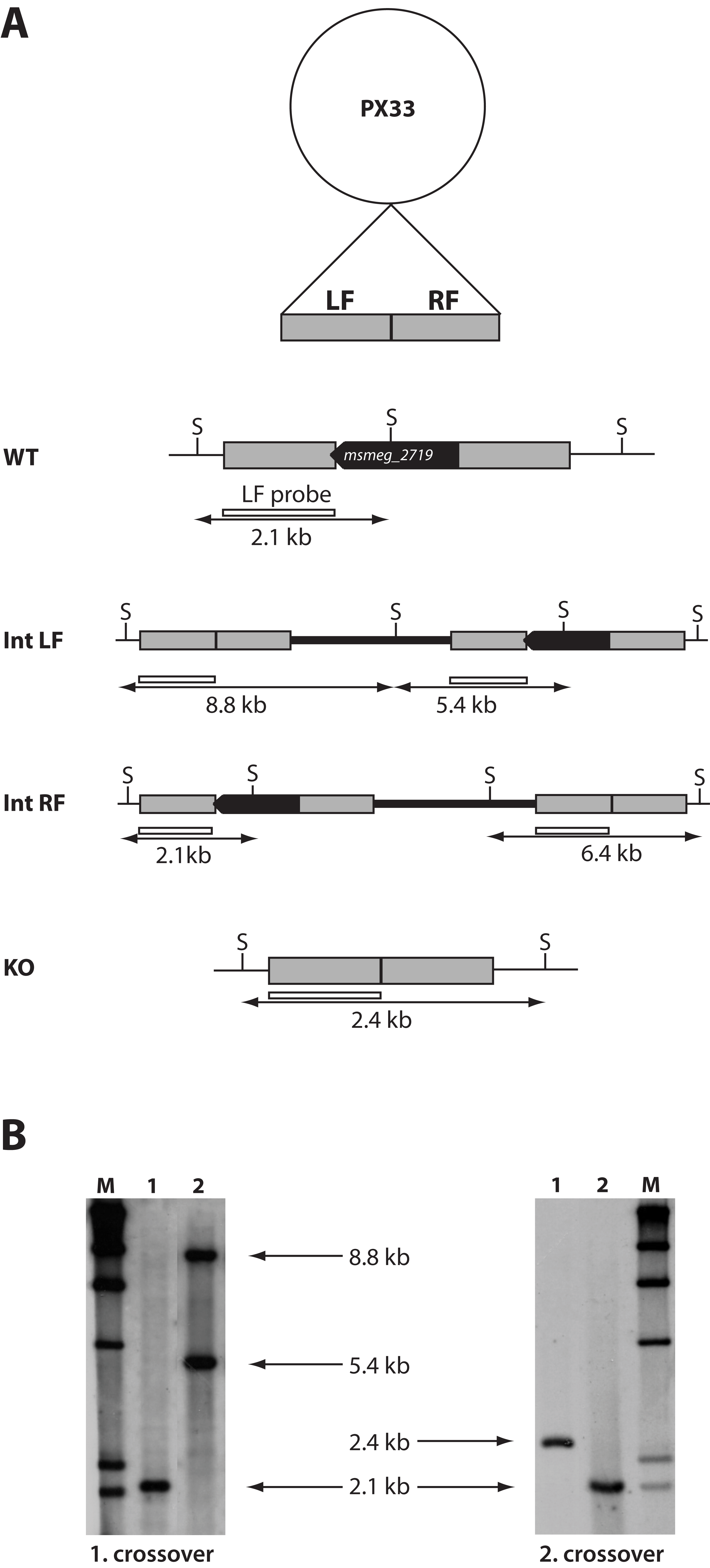

Supplement: Figure S2 — Construction of an unmarked msmeg_2719 deletion mutant of M. smegmatis mc2155. A. Schematic diagram of the two-step approach for deletion of msmeg_2719. The knockout construct consisted of two fragments flanking msmeg_2719 on the left (LF) and right (RF) in PX33. Integration of the vector (thick black line) into the chromosome (thin black line) via the left flank (Int LF) or right flank (Int RF) and subsequent deletion of msmeg_2719 (KO) are shown. Restriction sites of SmaI (S) and fragment sizes as detected in Southern hybridization are indicated. Drawing not to scale. WT, wild-type. B. Southern hybridization analysis of the integration event. Left panel, 1. crossover: SmaI-digests of genomic DNA of wild-type mc2155 (lane 1) and a candidate colony (lane 2) were probed with radiolabeled left flank PCR product of the deletion construct. Right panel, 2. crossover: Southern hybridization of msmeg_2719 deletion. Analysis of msmeg_2719 deletion strain (line 1) and wild-type mc2155 (lane 2) was performed as in the left panel. Molecular masses are indicated in kb. M, marker. (3.30 MB TIF) [file pone.0008614.s006.tif]

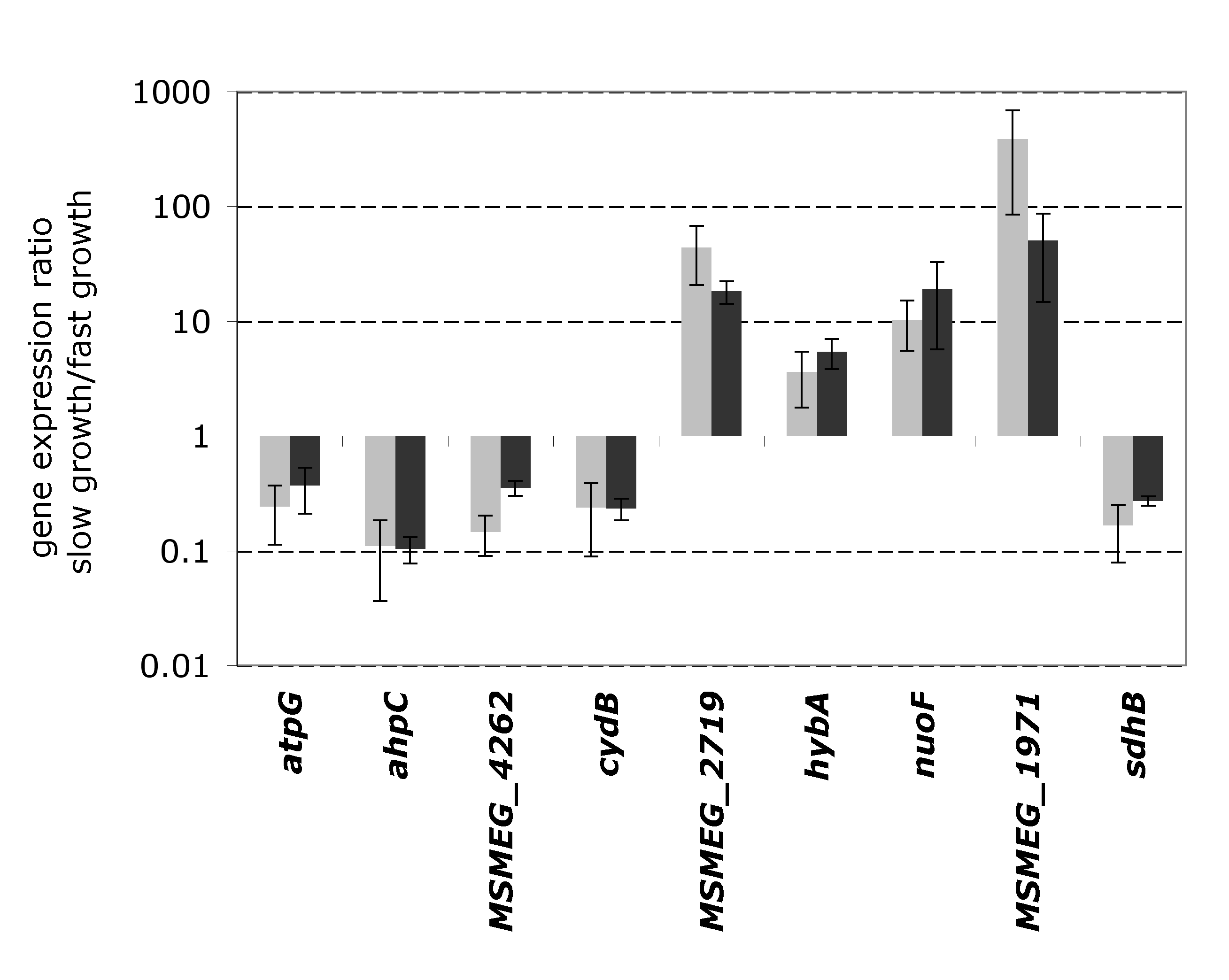

Supplement: Figure S3 — Validation of M. smegmatis gene expression ratios (slow growth rate at 50% oxygen saturation versus fast growth rate at 50% oxygen saturation) by quantitative RT-PCR (qPCR) (grey bars) compared to microarray results (black bars). For qPCR the expression of each gene was normalized to the expression of sigA. Error bars represent standard deviations of gene expression ratios from three biological replicates for each condition. (5.48 MB TIF) [file pone.0008614.s007.tif]
